# Supplementary material for: Perspectives of Dietary Assessment in Human Health and Disease
Source: Nutrients. 2022 Feb 16;14(4):830. doi: 10.3390/nu14040830 (PMC8877528; doi:10.3390/nu14040830)
Supplement: Supplementary file 1 [file nutrients-14-00830-s001.zip › Table S1.pdf]

Table S1 - PubMed search keywords "dietary assessment human health disease" filter 1 year
starting date 08/02/2022
Type of article: All
n = 1,998

1: Sutter DO, Bender N. Nutrient status and growth in vegan children. Nutr Res. 2021 Jul;91:13-25. doi: 10.1016/j.nutres.2021.04.005. Epub 2021 May 18. PMID: 34130207.

2: Schulz R, Slavin J. Perspective: Defining Carbohydrate Quality for Human Health and Environmental Sustainability. Adv Nutr. 2021 Jul 30;12(4):1108-1121. doi: 10.1093/advances/nmab050. PMID: 33951143.

3: English LK, Ard JD, Bailey RL, Bates M, Bazzano LA, Boushey CJ, Brown C, Butera G, Callahan EH, de Jesus J, Mattes RD, Mayer-Davis EJ, Novotny R, Obbagy JE, Rahavi EB, Sabate J, Snetselaar LG, Stoody EE, Van Horn LV, Venkatramanan S, Heymsfield SB. Evaluation of Dietary Patterns and All-Cause Mortality: A Systematic Review. JAMA Netw Open. 2021 Aug 2;4(8):e2122277. doi: 10.1001/jamanetworkopen.2021.22277. PMID: 34463743; PMCID: PMC8408672.

4: Petersen KS, Kris-Etherton PM. Diet Quality Assessment and the Relationship between Diet Quality and Cardiovascular Disease Risk. Nutrients. 2021 Nov 28;13(12):4305. doi: 10.3390/nu13124305. PMID: 34959857; PMCID: PMC8706326.

5: Marx W, Veronese N, Kelly JT, Smith L, Hockey M, Collins S, Trakman GL, Hoare E, Teasdale SB, Wade A, Lane M, Aslam H, Davis JA, O'Neil A, Shivappa N, Hebert JR, Blekkenhorst LC, Berk M, Segasby T, Jacka F. The Dietary Inflammatory Index and Human Health: An Umbrella Review of Meta-Analyses of Observational Studies. Adv Nutr. 2021 Oct 1;12(5):1681-1690. doi: 10.1093/advances/nmab037. PMID: 33873204; PMCID: PMC8483957.

6: Quintela BCSF, Carioca AAF, de Oliveira JGR, Fraser SDS, da Silva Junior GB. Dietary patterns and chronic kidney disease outcomes: A systematic review. Nephrology (Carlton). 2021 Jul;26(7):603-612. doi: 10.1111/nep.13883. Epub 2021 Apr 28. PMID: 33864650.

7: Metcalfe-Roach A, Yu AC, Golz E, Cirstea M, Sundvick K, Kliger D, Foulger LH, Mackenzie M, Finlay BB, Appel-Cresswell S. MIND and Mediterranean Diets Associated with Later Onset of Parkinson's Disease. Mov Disord. 2021 Apr;36(4):977-984. doi: 10.1002/mds.28464. Epub 2021 Jan 6. PMID: 33404118; PMCID: PMC8248352.

8: Narula N, Wong ECL, Dehghan M, Mente A, Rangarajan S, Lanas F, Lopez-Jaramillo P, Rohatgi P, Lakshmi PVM, Varma RP, Orlandini A, Avezum A, Wielgosz A, Poirier P, Almadi MA, Altuntas Y, Ng KK, Chifamba J, Yeates K, Puoane T, Khatib R, Yusuf R, Boström KB, Zatonska K, Iqbal R, Weida L, Yibing Z, Sidong L, Dans A, Yusufali A, Mohammadifard N, Marshall JK, Moayyedi P, Reinisch W, Yusuf S. Association of ultra-processed food intake with risk of inflammatory bowel disease: prospective cohort study. BMJ. 2021 Jul 14;374:n1554. doi:

10.1136/bmj.n1554. PMID: 34261638; PMCID: PMC8279036.

9: Marck CH, Probst Y, Chen J, Taylor B, van der Mei I. Dietary patterns and associations with health outcomes in Australian people with multiple sclerosis. Eur J Clin Nutr. 2021 Oct;75(10):1506-1514. doi: 10.1038/s41430-021-00864-y. Epub 2021 Feb 2. PMID: 33531638.

10: Alfawaz H, Yakout SM, Wani K, Aljumah GA, Ansari MGA, Khattak MNK, Hussain SD, Al-Daghri NM. Dietary Intake and Mental Health among Saudi Adults during COVID-19 Lockdown. Int J Environ Res Public Health. 2021 Feb 9;18(4):1653. doi: 10.3390/ijerph18041653. PMID: 33572328; PMCID: PMC7916162.

11: Baygi F, Mohammadi-Nasrabadi F, Zyriax BC, Jensen OC, Bygvraa DA, Oldenburg M, Nielsen JB. Global overview of dietary outcomes and dietary intake assessment methods in maritime settings: a systematic review. BMC Public Health. 2021 Aug 21;21(1):1579. doi: 10.1186/s12889-021-11593-z. PMID: 34419000; PMCID: PMC8379789.

12: Dahl IK, Dalgård C. Sami dietary habits and the risk of cardiometabolic disease: a systematic review. Int J Circumpolar Health. 2021 Dec;80(1):1873621. doi: 10.1080/22423982.2021.1873621. PMID: 33463398; PMCID: PMC7832988.

13: Gaona-Pineda EB, Martinez-Tapia B, Rodríguez-Ramírez S, Guerrero-Zúñiga S, Perez-Padilla R, Shamah-Levy T. Dietary patterns and sleep disorders in Mexican adults from a National Health and Nutrition Survey. J Nutr Sci. 2021 May 11;10:e34. doi: 10.1017/jns.2021.24. PMID: 34094514; PMCID: PMC8141679.

14: Ru Y, Wang N, Min Y, Wang X, McGurie V, Duan M, Xu X, Zhao X, Wu YH, Lu Y, Hsing AW, Zhu S. Characterization of dietary patterns and assessment of their relationships with metabolomic profiles: A community-based study. Clin Nutr. 2021 May;40(5):3531-3541. doi: 10.1016/j.clnu.2020.12.006. Epub 2020 Dec 10. PMID: 33349486.

15: Shahavandi M, Amini MR, Shahinfar H, Shab-Bidar S. Major dietary patterns and predicted cardiovascular disease risk in an Iranian adult population. Nutr Health. 2021 Mar;27(1):27-37. doi: 10.1177/0260106020952591. Epub 2020 Aug 31. PMID: 32867574.

16: Desmond MA, Sobiecki JG, Jaworski M, Płudowski P, Antoniewicz J, Shirley MK, Eaton S, Książyk J, Cortina-Borja M, De Stavola B, Fewtrell M, Wells JCK. Growth, body composition, and cardiovascular and nutritional risk of 5- to 10-y-old children consuming vegetarian, vegan, or omnivore diets. Am J Clin Nutr. 2021 Jun 1;113(6):1565-1577. doi: 10.1093/ajcn/nqaa445. PMID: 33740036; PMCID: PMC8176147.

17: Seo AR, Hwang TY. Relationship between Dietary Patterns and Cardiovascular Disease Risk in Korean Older Adults. Int J Environ Res Public Health. 2021 Apr 1;18(7):3703. doi: 10.3390/ijerph18073703. PMID: 33916265; PMCID: PMC8038041.

18: Little M, Hagar H, Zivot C, Dodd W, Skinner K, Kenny TA, Caughey A, Gaupholm J, Lemire M. Drivers and health implications of the dietary transition among Inuit in the Canadian Arctic: a scoping review. Public Health Nutr. 2021 Jun;24(9):2650-2668. doi: 10.1017/S1368980020002402. Epub 2020 Sep 11. PMID: 32914743.

19: Verger EO, Le Port A, Borderon A, Bourbon G, Moursi M, Savy M, Mariotti F, Martin-Prevel Y. Dietary Diversity Indicators and Their Associations with Dietary Adequacy and Health Outcomes: A Systematic Scoping Review. Adv Nutr. 2021 Oct 1;12(5):1659-1672. doi: 10.1093/advances/nmab009. PMID: 33684194; PMCID: PMC8483968.

20: Tisdel DM, Gadberry JJ, Burke SL, Carlini NA, Fleenor BS, Campbell MS. Dietary fat and alcohol in the prediction of indices of vascular health among young adults. Nutrition. 2021 Apr;84:111120. doi: 10.1016/j.nut.2020.111120. Epub 2020 Dec 15. PMID: 33515808.

21: Merino J, Joshi AD, Nguyen LH, Leeming ER, Mazidi M, Drew DA, Gibson R, Graham MS, Lo CH, Capdevila J, Murray B, Hu C, Selvachandran S, Hammers A, Bhupathiraju SN, Sharma SV, Sudre C, Astley CM, Chavarro JE, Kwon S, Ma W, Menni C, Willett WC, Ourselin S, Steves CJ, Wolf J, Franks PW, Spector TD, Berry S, Chan AT. Diet quality and risk and severity of COVID-19: a prospective cohort study. Gut. 2021 Nov;70(11):2096-2104. doi: 10.1136/gutjnl-2021-325353. Epub 2021 Sep 6. PMID: 34489306; PMCID: PMC8500931.

22: Charlot A, Hutt F, Sabatier E, Zoll J. Beneficial Effects of Early Time-Restricted Feeding on Metabolic Diseases: Importance of Aligning Food Habits with the Circadian Clock. Nutrients. 2021 Apr 22;13(5):1405. doi: 10.3390/nu13051405. PMID: 33921979; PMCID: PMC8143522.

23: Qin Y, Jiao C, Huang S, Li Y, Zhang Z, Bao W, Mao L. "Zuòyuèzi" dietary and behavioural associations with maternal health among puerperal women in South China. Asia Pac J Clin Nutr. 2021 Jun;30(2):291-302. doi: 10.6133/apjcn.202106_30(2).0014. PMID: 34191432.

24: Botelho RBA, Ginani VC, Cupertino AP. Health Conditions and Dietary Intake Among Brazilian Immigrants in the United States of America. J Immigr Minor Health. 2021 Dec;23(6):1259-1266. doi: 10.1007/s10903-021-01139-1. Epub 2021 Jan 23. PMID: 33486659.

25: Fenton S, Burrows TL, Skinner JA, Duncan MJ. The influence of sleep health on dietary intake: a systematic review and meta-analysis of intervention studies. J Hum Nutr Diet. 2021 Apr;34(2):273-285. doi: 10.1111/jhn.12813. Epub 2020 Oct 1. PMID: 33001515.

26: Hassani Zadeh S, Mansoori A, Hosseinzadeh M. Relationship between dietary patterns and non-alcoholic fatty liver disease: A systematic review and meta-analysis. J Gastroenterol Hepatol. 2021 Jun;36(6):1470-1478. doi: 10.1111/jgh.15363. Epub 2020 Dec 14. PMID: 33269500.

27: Iqbal R, Dehghan M, Mente A, Rangarajan S, Wielgosz A, Avezum A, Seron P, AlHabib KF, Lopez-Jaramillo P, Swaminathan S, Mohammadifard N, Zatońska K, Bo H, Varma RP, Rahman O, Yusufali A, Lu Y, Ismail N, Rosengren A, Imeryuz N, Yeates K, Chifamba J, Dans A, Kumar R, Xiaoyun L, Tsolekile L, Khatib R, Diaz R, Teo K, Yusuf S. Associations of unprocessed and processed meat intake with mortality and cardiovascular disease in 21 countries [Prospective Urban Rural Epidemiology (PURE) Study]: a prospective cohort study. Am J Clin Nutr. 2021 Sep 1;114(3):1049-1058. doi: 10.1093/ajcn/nqaa448. PMID: 33787869.

28: Pravst I, Lavriša Ž, Hribar M, Hristov H, Kvarantan N, Seljak BK, Gregorič M, Blaznik U, Gregorič N, Zaletel K, Oblak A, Osredkar J, Žmitek K, Kušar A. Dietary Intake of Folate and Assessment of the Folate Deficiency Prevalence in Slovenia Using Serum Biomarkers. Nutrients. 2021 Oct 28;13(11):3860. doi: 10.3390/nu13113860. PMID: 34836112; PMCID: PMC8620305.

29: García Mayor J, Moreno Llamas A, de la Cruz Sánchez E. Actividad física y estilo de vida relacionado con la salud en la población española con enfermedad musculoesquelética [Physical activity and health-related lifestyle in the Spanish population living with musculoskeletal disease]. Nutr Hosp. 2021 Feb 23;38(1):128-138. Spanish. doi: 10.20960/nh.02998. PMID: 33179513.

30: Gatto NM, Garcia-Cano J, Irani C, Jaceldo-Siegl K, Liu T, Chen Z, Paul J, Fraser G, Wang C, Lee GJ. Vegetarian Dietary Patterns and Cognitive Function among Older Adults: The Adventist Health Study-2. J Nutr Gerontol Geriatr. 2021 Oct-Dec;40(4):197-214. doi: 10.1080/21551197.2021.1965939. Epub 2021 Aug 19. PMID: 34412570.

31: Paknahad Z, Moosavian SP, Jervekani ZT, Hasanzadeh A, Hashemi M. Dietary total antioxidant capacity and severity of stenosis in patients with coronary artery disease. Int J Vitam Nutr Res. 2021 Jun;91(3-4):235-241. doi: 10.1024/0300-9831/a000622. Epub 2020 Jan 27. PMID: 31984875.

32: Whittaker J, Wu K. Low-fat diets and testosterone in men: Systematic review and meta-analysis of intervention studies. J Steroid Biochem Mol Biol. 2021 Jun;210:105878. doi: 10.1016/j.jsbmb.2021.105878. Epub 2021 Mar 16. PMID: 33741447.

33: Gao M, Jebb SA, Aveyard P, Ambrosini GL, Perez-Cornago A, Carter J, Sun X, Piernas C. Associations between dietary patterns and the incidence of total and fatal cardiovascular disease and all-cause mortality in 116,806 individuals from the UK Biobank: a prospective cohort study. BMC Med. 2021 Apr 22;19(1):83. doi: 10.1186/s12916-021-01958-x. PMID: 33882922; PMCID: PMC8061025.

34: Matsuyama S, Sawada N, Tomata Y, Zhang S, Goto A, Yamaji T, Iwasaki M, Inoue M, Tsuji I, Tsugane S; Japan Public Health Center-based Prospective Study Group. Association between adherence to the Japanese diet and all-cause and cause-specific mortality: the Japan Public Health Center-based Prospective Study. Eur J Nutr. 2021 Apr;60(3):1327-1336. doi: 10.1007/s00394-020-02330-0. Epub 2020 Jul

16. PMID: 32676701; PMCID: PMC7987617.

35: Zhu J, Tan Y, Lu W, He Y, Yu Z. Current Assessment of Weight, Dietary and Physical Activity Behaviors among Middle and High School Students in Shanghai, China-A 2019 Cross-Sectional Study. Nutrients. 2021 Nov 30;13(12):4331. doi: 10.3390/nu13124331. PMID: 34959883; PMCID: PMC8707717.

36: Kaiser J, van Daalen KR, Thayyil A, Cocco MTARR, Caputo D, Oliver-Williams C. A Systematic Review of the Association Between Vegan Diets and Risk of Cardiovascular Disease. J Nutr. 2021 Jun 1;151(6):1539-1552. doi: 10.1093/jn/nxab037. PMID: 33831953; PMCID: PMC8169813.

37: Filip R, Anchidin-Norocel L, Gheorghita R, Savage WK, Dimian M. Changes in Dietary Patterns and Clinical Health Outcomes in Different Countries during the SARS-CoV-2 Pandemic. Nutrients. 2021 Oct 15;13(10):3612. doi: 10.3390/nu13103612. PMID: 34684615; PMCID: PMC8539259.

38: Porykali B, Davies A, Brooks C, Melville H, Allman-Farinelli M, Coombes J. Effects of Nutritional Interventions on Cardiovascular Disease Health Outcomes in Aboriginal and Torres Strait Islander Australians: A Scoping Review. Nutrients. 2021 Nov 15;13(11):4084. doi: 10.3390/nu13114084. PMID: 34836337; PMCID: PMC8620344.

39: Ramírez-Mejía MM, Díaz-Orozco LE, Barranco-Fragoso B, Méndez-Sánchez N. A Review of the Increasing Prevalence of Metabolic-Associated Fatty Liver Disease (MAFLD) in Children and Adolescents Worldwide and in Mexico and the Implications for Public Health. Med Sci Monit. 2021 Aug 30;27:e934134. doi: 10.12659/MSM.934134. PMID: 34456329; PMCID: PMC8415038.

40: Ivey KL, Nguyen XT, Posner D, Rogers GB, Tobias DK, Song R, Ho YL, Li R, Wilson PWF, Cho K, Gaziano JM, Hu FB, Willett WC, Djoussé L. The Structure of Relationships between the Human Exposome and Cardiometabolic Health: The Million Veteran Program. Nutrients. 2021 Apr 19;13(4):1364. doi: 10.3390/nu13041364. PMID: 33921792; PMCID: PMC8073795.

41: Glenn AJ, Lo K, Jenkins DJA, Boucher BA, Hanley AJ, Kendall CWC, Manson JE, Vitolins MZ, Snetselaar LG, Liu S, Sievenpiper JL. Relationship Between a Plant-Based Dietary Portfolio and Risk of Cardiovascular Disease: Findings From the Women's Health Initiative Prospective Cohort Study. J Am Heart Assoc. 2021 Aug 17;10(16):e021515. doi: 10.1161/JAHA.121.021515. Epub 2021 Aug 4. PMID: 34346245; PMCID: PMC8475059.

42: Li Z, Gao Y, Byrd DA, Gibbs DC, Prizment AE, Lazovich D, Bostick RM. Novel Dietary and Lifestyle Inflammation Scores Directly Associated with All-Cause, All-Cancer, and All-Cardiovascular Disease Mortality Risks Among Women. J Nutr. 2021 Apr 8;151(4):930-939. doi: 10.1093/jn/nxaa388. PMID: 33693725; PMCID: PMC8030700.

43: Jimenez-Torres J, Alcalá-Diaz JF, Torres-Peña JD, Gutierrez-Mariscal FM,

Leon-Acuña A, Gómez-Luna P, Fernández-Gandara C, Quintana-Navarro GM, Fernandez-Garcia JC, Perez-Martinez P, Ordovas JM, Delgado-Lista J, Yubero-Serrano EM, Lopez-Miranda J. Mediterranean Diet Reduces Atherosclerosis Progression in Coronary Heart Disease: An Analysis of the CORDIOPREV Randomized Controlled Trial. Stroke. 2021 Nov;52(11):3440-3449. doi: 10.1161/STROKEAHA.120.033214. Epub 2021 Aug 10. Erratum in: Stroke. 2021 Nov;52(11):e754. PMID: 34372670.

44: Kim SM, Kim MH, Ryu DR, Oh HJ. The dietary intake of chronic kidney disease according to stages: Findings from the Korean National Health and Nutritional Examination Survey. PLoS One. 2021 Nov 29;16(11):e0260242. doi: 10.1371/journal.pone.0260242. PMID: 34843534; PMCID: PMC8629218.

45: Ramp D, Mols F, Ezendam N, Beijer S, Bours M, Winkels R, de Vries J, Seidell JC, Kampman E, Hoedjes M. Psychological distress and lower health-related quality of life are associated with need for dietary support among colorectal cancer survivors with overweight or obesity. Support Care Cancer. 2021 Dec;29(12):7659-7668. doi: 10.1007/s00520-021-06306-6. Epub 2021 Jun 17. PMID: 34142281; PMCID: PMC8550733.

46: Mumme KD, Conlon CA, von Hurst PR, Jones B, de Seymour J, Heath AM, Stonehouse W, Coad J, Haskell-Ramsay CF, Beck KL. Relative Validity and Reproducibility of a Food Frequency Questionnaire for Assessing Dietary Patterns and Food Group Intake in Older New Zealand Adults: The Researching Eating, Activity, and Cognitive Health Study. J Acad Nutr Diet. 2021 Dec;121(12):2389-2400.e10. doi: 10.1016/j.jand.2021.05.022. Epub 2021 Jul 17. PMID: 34281811.

47: Kim Y, Je Y, Giovannucci EL. Association between dietary fat intake and mortality from all-causes, cardiovascular disease, and cancer: A systematic review and meta-analysis of prospective cohort studies. Clin Nutr. 2021 Mar;40(3):1060-1070. doi: 10.1016/j.clnu.2020.07.007. Epub 2020 Jul 14. PMID: 32723506.

48: Lecorguillé M, Teo S, Phillips CM. Maternal Dietary Quality and Dietary Inflammation Associations with Offspring Growth, Placental Development, and DNA Methylation. Nutrients. 2021 Sep 8;13(9):3130. doi: 10.3390/nu13093130. PMID: 34579008; PMCID: PMC8468062.

49: Gotfredsen JL, Hoppe C, Andersen R, Andersen EW, Landberg R, Overvad K, Tetens I. Effects of substitution dietary guidelines targeted at prevention of IHD on dietary intake and risk factors in middle-aged Danish adults: the Diet and Prevention of Ischemic Heart Disease: a Translational Approach (DIPI) randomised controlled trial. Br J Nutr. 2021 Oct 28;126(8):1179-1193. doi: 10.1017/S0007114520005164. Epub 2020 Dec 28. PMID: 33357247.

50: Solbak NM, Robson PJ, Lo Siou G, Al Rajabi A, Paek S, Vena JE, Kirkpatrick SI. Administering a combination of online dietary assessment tools, the Automated Self-Administered 24-Hour Dietary Assessment Tool, and Diet History Questionnaire II, in a cohort of adults in Alberta's Tomorrow Project. J Acad

Nutr Diet. 2021 Jul;121(7):1312-1326. doi: 10.1016/j.jand.2021.01.014. Epub 2021 Feb 18. PMID: 33612438.

51: Ennis MA, Elango R. A discussion on the 'dispensable' amino acids. Curr Opin Clin Nutr Metab Care. 2021 Sep 1;24(5):395-401. doi: 10.1097/MCO.0000000000000784. PMID: 34387624.

52: Kim SA, Shin S. Dietary Patterns and the Risk of Dyslipidemia in Korean Adults: A Prospective Cohort Study Based on the Health Examinees (HEXA) Study. J Acad Nutr Diet. 2021 Jul;121(7):1242-1257.e2. doi: 10.1016/j.jand.2020.08.090. Epub 2020 Nov 3. PMID: 33158796.

53: Prentice RL, Howard BV, Van Horn L, Neuhouser ML, Anderson GL, Tinker LF, Lampe JW, Raftery D, Pettinger M, Aragaki AK, Thomson CA, Mossavar-Rahmani Y, Stefanick ML, Cauley JA, Rossouw JE, Manson JE, Chlebowski RT. Nutritional epidemiology and the Women's Health Initiative: a review. Am J Clin Nutr. 2021 May 8;113(5):1083-1092. doi: 10.1093/ajcn/nqab091. PMID: 33876183; PMCID: PMC8120331.

54: Sohouli MH, Sayyari AA, Lari A, Nameni G, Lotfi M, Fatahi S, Saneie S, Găman MA, Moodi F, Raee P, Aghamiri S, Rayi A, Shahriari A, Moodi V. Association of dietary insulinaemic potential and odds of non-alcoholic fatty liver disease among adults: A case-control study. J Hum Nutr Diet. 2021 Oct;34(5):901-909. doi: 10.1111/jhn.12865. Epub 2021 Feb 15. PMID: 33586811.

55: Mao Z, Prizment AE, Lazovich D, Bostick RM. Associations of dietary and lifestyle oxidative balance scores with mortality risk among older women: the Iowa Women's Health Study. Eur J Nutr. 2021 Oct;60(7):3873-3886. doi: 10.1007/s00394-021-02557-5. Epub 2021 Apr 21. PMID: 33881582.

56: Lucassen DA, Lasschuijt MP, Camps G, Van Loo EJ, Fischer ARH, de Vries RAJ, Haarman JAM, Simons M, de Vet E, Bos-de Vos M, Pan S, Ren X, de Graaf K, Lu Y, Feskens EJM, Brouwer-Brolsma EM. Short and Long-Term Innovations on Dietary Behavior Assessment and Coaching: Present Efforts and Vision of the Pride and Prejudice Consortium. Int J Environ Res Public Health. 2021 Jul 25;18(15):7877. doi: 10.3390/ijerph18157877. PMID: 34360170; PMCID: PMC8345591.

57: Liu X, Morris MC, Dhana K, Ventrelle J, Johnson K, Bishop L, Hollings CS, Boulin A, Laranjo N, Stubbs BJ, Reilly X, Carey VJ, Wang Y, Furtado JD, Marcovina SM, Tangney C, Aggarwal NT, Arfanakis K, Sacks FM, Barnes LL. Mediterranean-DASH Intervention for Neurodegenerative Delay (MIND) study: Rationale, design and baseline characteristics of a randomized control trial of the MIND diet on cognitive decline. Contemp Clin Trials. 2021 Mar;102:106270. doi: 10.1016/j.cct.2021.106270. Epub 2021 Jan 9. PMID: 33434704; PMCID: PMC8042655.

58: Zhou YF, Song XY, Wu J, Chen GC, Neelakantan N, van Dam RM, Feng L, Yuan JM, Pan A, Koh WP. Association Between Dietary Patterns in Midlife and Healthy Ageing in Chinese Adults: The Singapore Chinese Health Study. J Am Med Dir

Assoc. 2021 Jun;22(6):1279-1286. doi: 10.1016/j.jamda.2020.09.045. Epub 2020 Nov 18. PMID: 33218913.

59: Ma Z, Hummel SL, Sun N, Chen Y. From salt to hypertension, what is missed? J Clin Hypertens (Greenwich). 2021 Dec;23(12):2033-2041. doi: 10.1111/jch.14402. Epub 2021 Nov 30. PMID: 34846798; PMCID: PMC8696232.

60: Low JHM, Toh DWK, Ng MTT, Fam J, Kua EH, Kim JE. A Systematic Review and Meta-Analysis of the Impact of Different Intensity of Dietary Counselling on Cardiometabolic Health in Middle-Aged and Older Adults. Nutrients. 2021 Aug 25;13(9):2936. doi: 10.3390/nu13092936. PMID: 34578814; PMCID: PMC8469488.

61: Mendoza-Vasconez AS, Landry MJ, Crimarco A, Bladier C, Gardner CD. Sustainable Diets for Cardiovascular Disease Prevention and Management. Curr Atheroscler Rep. 2021 May 10;23(7):31. doi: 10.1007/s11883-021-00929-0. PMID: 33970349.

62: Nakashita C, Xi L, Inoue Y, Kabura R, Masuda S, Yamano Y, Katoh T. Impact of dietary compositions and patterns on the prevalence of nonalcoholic fatty liver disease in Japanese men: a cross-sectional study. BMC Gastroenterol. 2021 Sep 4;21(1):342. doi: 10.1186/s12876-021-01919-x. PMID: 34481454; PMCID: PMC8418738.

63: Cox S, Sandall A, Smith L, Rossi M, Whelan K. Food additive emulsifiers: a review of their role in foods, legislation and classifications, presence in food supply, dietary exposure, and safety assessment. Nutr Rev. 2021 May 12;79(6):726-741. doi: 10.1093/nutrit/nuaa038. PMID: 32626902.

64: Lukomskyj N, Allman-Farinelli M, Shi Y, Rangan A. Dietary exposures in childhood and adulthood and cardiometabolic outcomes: a systematic scoping review. J Hum Nutr Diet. 2021 Jun;34(3):511-523. doi: 10.1111/jhn.12841. Epub 2021 Jan 6. PMID: 33406314.

65: Das A, Cumming RG, Naganathan V, Blyth F, Le Couteur DG, Handelsman DJ, Waite LM, Ribeiro RVR, Simpson SJ, Hirani V. Dietary and supplemental antioxidant intake and risk of major adverse cardiovascular events in older men: The concord health and ageing in men project. Nutr Metab Cardiovasc Dis. 2021 Apr 9;31(4):1102-1112. doi: 10.1016/j.numecd.2020.11.032. Epub 2020 Dec 8. PMID: 33549432.

66: Boelens Keun JT, Arnoldussen IA, Vriend C, van de Rest O. Dietary Approaches to Improve Efficacy and Control Side Effects of Levodopa Therapy in Parkinson's Disease: A Systematic Review. Adv Nutr. 2021 Dec 1;12(6):2265-2287. doi: 10.1093/advances/nmab060. PMID: 34113965; PMCID: PMC8634393.

67: Chen G. Dietary N-epsilon-carboxymethyllysine as for a major glycotoxin in foods: A review. Compr Rev Food Sci Food Saf. 2021 Sep;20(5):4931-4949. doi: 10.1111/1541-4337.12817. Epub 2021 Aug 10. PMID: 34378329.

68: Wang X, Wang Y, Xu W, Lan L, Li Y, Wang L, Sun X, Yang C, Jiang Y, Feng R.

Dietary isoflavones intake is inversely associated with non-alcoholic fatty liver disease, hyperlipidaemia and hypertension. Int J Food Sci Nutr. 2022 Feb;73(1):60-70. doi: 10.1080/09637486.2021.1910630. Epub 2021 Apr 25. PMID: 33899670.

69: LeVatte M, Keshteli AH, Zarei P, Wishart DS. Applications of Metabolomics to Precision Nutrition. Lifestyle Genom. 2022;15(1):1-9. doi: 10.1159/000518489. Epub 2021 Sep 8. PMID: 34518463.

70: Kocanda L, Brain K, Frawley J, Schumacher TL, May J, Rollo ME, Brown LJ. The Effectiveness of Randomized Controlled Trials to Improve Dietary Intake in the Context of Cardiovascular Disease Prevention and Management in Rural Communities: A Systematic Review. J Acad Nutr Diet. 2021 Oct;121(10):2046-2070.e1. doi: 10.1016/j.jand.2021.05.025. Epub 2021 Jul 8. PMID: 34247977.

71: Baderol Allam FN, Ab Hamid MR, Buhari SS, Md Noor H. Web-Based Dietary and Physical Activity Intervention Programs for Patients With Hypertension: Scoping Review. J Med Internet Res. 2021 Mar 15;23(3):e22465. doi: 10.2196/22465. PMID: 33720036; PMCID: PMC8074856.

72: McKenzie BL, Coyle DH, Santos JA, Burrows T, Rosewarne E, Peters SAE, Carcel C, Jaacks LM, Norton R, Collins CE, Woodward M, Webster J. Investigating sex differences in the accuracy of dietary assessment methods to measure energy intake in adults: a systematic review and meta-analysis. Am J Clin Nutr. 2021 May 8;113(5):1241-1255. doi: 10.1093/ajcn/nqaa370. PMID: 33564834; PMCID: PMC8106762.

73: Kebbe M, Gao M, Perez-Cornago A, Jebb SA, Piernas C. Adherence to international dietary recommendations in association with all-cause mortality and fatal and non-fatal cardiovascular disease risk: a prospective analysis of UK Biobank participants. BMC Med. 2021 Jun 23;19(1):134. doi: 10.1186/s12916-021-02011-7. PMID: 34158032; PMCID: PMC8220774.

74: Garcêz LS, Avelar CR, Fonseca NSS, Costa PRF, Lyra AC, Cunha CM, Jesus RP, Oliveira LPM. Effect of dietary carbohydrate and lipid modification on clinical and anthropometric parameters in nonalcoholic fatty liver disease: a systematic review and meta-analysis. Nutr Rev. 2021 Nov 10;79(12):1321-1337. doi: 10.1093/nutrit/nuaa146. PMID: 33515021.

75: Volta M, Turrini E, Carnevale C, Valeri E, Gatta V, Polidori P, Maione M. Co-benefits of changing diet. A modelling assessment at the regional scale integrating social acceptability, environmental and health impacts. Sci Total Environ. 2021 Feb 20;756:143708. doi: 10.1016/j.scitotenv.2020.143708. Epub 2020 Nov 21. PMID: 33302065.

76: Potter T, Vieira R, de Roos B. Perspective: Application of N-of-1 Methods in Personalized Nutrition Research. Adv Nutr. 2021 Jun 1;12(3):579-589. doi: 10.1093/advances/nmaa173. PMID: 33460438; PMCID: PMC8166550.

77: Sharma J, Ludin H, Chauhan M, Zodpey S. Public health nutrition in Afghanistan-policies, strategies and capacity-building: current scenario and initiatives. East Mediterr Health J. 2021 Jul 29;27(7):728-737. doi: 10.26719/emhj.21.043. PMID: 34369588.

78: Rafiq T, Azab SM, Teo KK, Thabane L, Anand SS, Morrison KM, de Souza RJ, Britz-McKibbin P. Nutritional Metabolomics and the Classification of Dietary Biomarker Candidates: A Critical Review. Adv Nutr. 2021 Dec 1;12(6):2333-2357. doi: 10.1093/advances/nmab054. PMID: 34015815; PMCID: PMC8634495.

79: Ooi JY, Wolfenden L, Sutherland R, Nathan N, Oldmeadow C, Mclaughlin M, Barnes C, Hall A, Vanderlee L, Yoong SL. A Systematic Review of the Recent Consumption Levels of Sugar-Sweetened Beverages in Children and Adolescents From the World Health Organization Regions With High Dietary-Related Burden of Disease. Asia Pac J Public Health. 2022 Jan;34(1):11-24. doi: 10.1177/10105395211014642. Epub 2021 May 20. PMID: 34013784.

80: Aljada B, Zohni A, El-Matary W. The Gluten-Free Diet for Celiac Disease and Beyond. Nutrients. 2021 Nov 9;13(11):3993. doi: 10.3390/nu13113993. PMID: 34836247; PMCID: PMC8625243.

81: Ba DM, Gao X, Al-Shaar L, Muscat J, Chinchilli VM, Ssentongo P, Zhang X, Liu G, Beelman RB, Richie JP Jr. Prospective study of dietary mushroom intake and risk of mortality: results from continuous National Health and Nutrition Examination Survey (NHANES) 2003-2014 and a meta-analysis. Nutr J. 2021 Sep 21;20(1):80. doi: 10.1186/s12937-021-00738-w. PMID: 34548082; PMCID: PMC8454070.

82: Espinosa A. Health Behaviors, Self-Rated Health, and Health Consciousness Among Latinx in New York City. J Immigr Minor Health. 2021 Jun;23(3):591-596. doi: 10.1007/s10903-020-01053-y. PMID: 32683521.

83: de Araújo TP, de Moraes MM, Magalhães V, Afonso C, Santos C, Rodrigues SSP. Ultra-Processed Food Availability and Noncommunicable Diseases: A Systematic Review. Int J Environ Res Public Health. 2021 Jul 10;18(14):7382. doi: 10.3390/ijerph18147382. PMID: 34299832; PMCID: PMC8306957.

84: Noakes TD. Hiding unhealthy heart outcomes in a low-fat diet trial: the Women's Health Initiative Randomized Controlled Dietary Modification Trial finds that postmenopausal women with established coronary heart disease were at increased risk of an adverse outcome if they consumed a low-fat 'heart-healthy' diet. Open Heart. 2021 Jul;8(2):e001680. doi: 10.1136/openhrt-2021-001680. PMID: 34290045; PMCID: PMC8296783.

85: Scarallo L, Lionetti P. Dietary Management in Pediatric Patients with Crohn's Disease. Nutrients. 2021 May 11;13(5):1611. doi: 10.3390/nu13051611. PMID: 34064976; PMCID: PMC8150738.

86: Schönbach JK, Lhachimi SK. To what extent could cardiovascular diseases be

reduced if Germany applied fiscal policies to increase fruit and vegetable consumption? A quantitative health impact assessment. Public Health Nutr. 2021 Jun;24(9):2570-2576. doi: 10.1017/S1368980020000634. Epub 2020 Jul 14. PMID: 32662362; PMCID: PMC8145472.

87: Drake D, Hayden AM, Delkoski S. Love the Food That Loves You Back: A Planetary Health and Women's Heart Health Partnership. Creat Nurs. 2021 Nov 1;27(4):262-266. doi: 10.1891/cn-2021-0016. PMID: 34903630.

88: Aleksandrova K, Koelman L, Rodrigues CE. Dietary patterns and biomarkers of oxidative stress and inflammation: A systematic review of observational and intervention studies. Redox Biol. 2021 Jun;42:101869. doi: 10.1016/j.redox.2021.101869. Epub 2021 Jan 22. PMID: 33541846; PMCID: PMC8113044.

89: D'Amico E, Grosso G, Nieves JW, Zanghì A, Factor-Litvak P, Mitsumoto H. Metabolic Abnormalities, Dietary Risk Factors and Nutritional Management in Amyotrophic Lateral Sclerosis. Nutrients. 2021 Jun 30;13(7):2273. doi: 10.3390/nu13072273. PMID: 34209133; PMCID: PMC8308334.

90: Kilvington A, Barnaba C, Rajasekaran S, Laurens Leimanis ML, Medina-Meza IG. Lipid profiling and dietary assessment of infant formulas reveal high intakes of major cholesterol oxidative product (7-ketocholesterol). Food Chem. 2021 Aug 30;354:129529. doi: 10.1016/j.foodchem.2021.129529. Epub 2021 Mar 9. PMID: 33761334.

91: Al-Dashti YA, Holt RR, Keen CL, Hackman RM. Date Palm Fruit (<i>Phoenix dactylifera</i>): Effects on Vascular Health and Future Research Directions. Int J Mol Sci. 2021 Apr 28;22(9):4665. doi: 10.3390/ijms22094665. PMID: 33925062; PMCID: PMC8125345.

92: Duplantier SC, Gardner CD. A Critical Review of the Study of Neuroprotective Diets to Reduce Cognitive Decline. Nutrients. 2021 Jun 30;13(7):2264. doi: 10.3390/nu13072264. PMID: 34208980; PMCID: PMC8308213.

93: Zhong VW, Ning H, Van Horn L, Carnethon MR, Wilkins JT, Lloyd-Jones DM, Allen NB. Diet Quality and Long-Term Absolute Risks for Incident Cardiovascular Disease and Mortality. Am J Med. 2021 Apr;134(4):490-498.e24. doi: 10.1016/j.amjmed.2020.08.012. Epub 2020 Sep 14. PMID: 32941845; PMCID: PMC7956066.

94: Poulsen KO, Sundekilde UK. The Metabolomic Analysis of Human Milk Offers Unique Insights into Potential Child Health Benefits. Curr Nutr Rep. 2021 Mar;10(1):12-29. doi: 10.1007/s13668-020-00345-x. Epub 2021 Feb 8. PMID: 33555534.

95: Rossa-Roccor V, Richardson CG, Murphy RA, Gadermann AM. The association between diet and mental health and wellbeing in young adults within a biopsychosocial framework. PLoS One. 2021 Jun 3;16(6):e0252358. doi:

10.1371/journal.pone.0252358. PMID: 34081708; PMCID: PMC8174719.

96: Duncanson K, Burns G, Pryor J, Keely S, Talley NJ. Mechanisms of Food-Induced Symptom Induction and Dietary Management in Functional Dyspepsia. Nutrients. 2021 Mar 28;13(4):1109. doi: 10.3390/nu13041109. PMID: 33800668; PMCID: PMC8066021.

97: Ding G, Jing Y, Han Y, Sun P, Liang S, Liu J, Wang X, Lian Y, Fang Y, Jin Z, Li W. Monitoring of Aluminum content in food and assessment of dietary exposure of residents in North China. Food Addit Contam Part B Surveill. 2021 Sep;14(3):177-183. doi: 10.1080/19393210.2021.1912191. Epub 2021 Aug 6. PMID: 34362289.

98: Ramadas A, Tham SM, Lalani SA, Shyam S. Diet Quality of Malaysians across Lifespan: A Scoping Review of Evidence in a Multi-Ethnic Population. Nutrients. 2021 Apr 20;13(4):1380. doi: 10.3390/nu13041380. PMID: 33924050; PMCID: PMC8074191.

99: Russell C, Grimes C, Baker P, Sievert K, Lawrence MA. The drivers, trends and dietary impacts of non-nutritive sweeteners in the food supply: a narrative review. Nutr Res Rev. 2021 Dec;34(2):185-208. doi: 10.1017/S0954422420000268. Epub 2020 Nov 5. PMID: 33148371.

100: Tessitore M, Sorrentino E, Schiano Di Cola G, Colucci A, Vajro P, Mandato C. Malnutrition in Pediatric Chronic Cholestatic Disease: An Up-to-Date Overview. Nutrients. 2021 Aug 13;13(8):2785. doi: 10.3390/nu13082785. PMID: 34444944; PMCID: PMC8400766.

101: Parveen S. Impact of calorie restriction and intermittent fasting on periodontal health. Periodontol 2000. 2021 Oct;87(1):315-324. doi: 10.1111/prd.12400. PMID: 34463980.

102: Iguacel I, Schmidt JA, Perez-Cornago A, Van Puyvelde H, Travis R, Stepien M, Scalbert A, Casagrande C, Weiderpass E, Riboli E, Schulze MB, Skeie G, Bodén S, Boeing H, Cross AJ, Harlid S, Jensen TE, Huerta JM, Katzke V, Kühn T, Lujan-Barroso L, Masala G, Rodriguez-Barranco M, Rostgaard-Hansen AL, van der Schouw YT, Vermeulen R, Tagliabue G, Tjønneland A, Trevisan M, Ferrari P, Gunter MJ, Huybrechts I. Associations between dietary amino acid intakes and blood concentration levels. Clin Nutr. 2021 Jun;40(6):3772-3779. doi: 10.1016/j.clnu.2021.04.036. Epub 2021 Apr 27. PMID: 34130023.

103: Ding K, Zhou H, Gao T, Xu R, Chen L, Cai J, Zhang H, Zhong F, Ma A. Dietary patterns and cognitive function in older adults residing in rural China. Asia Pac J Clin Nutr. 2021 Jun;30(2):253-262. doi: 10.6133/apjcn.202106_30(2).0010. PMID: 34191428.

104: Bryant L, Rangan A, Grafenauer S. Lupins and Health Outcomes: A Systematic Literature Review. Nutrients. 2022 Jan 13;14(2):327. doi: 10.3390/nu14020327. PMID: 35057507; PMCID: PMC8777979.

105: Hakeem FF, Bernabé E, Sabbah W. Association Between Oral Health and Frailty Among American Older Adults. J Am Med Dir Assoc. 2021 Mar;22(3):559-563.e2. doi: 10.1016/j.jamda.2020.07.023. Epub 2020 Aug 25. PMID: 32859517.

106: Wu F, Pahkala K, Juonala M, Rovio SP, Sabin MA, Rönnemaa T, Smith KJ, Jula A, Lehtimäki T, Hutri-Kähönen N, Kähönen M, Laitinen T, Viikari JSA, Raitakari OT, Magnussen CG. Childhood and long-term dietary calcium intake and adult cardiovascular risk in a population with high calcium intake. Clin Nutr. 2021 Apr;40(4):1926-1931. doi: 10.1016/j.clnu.2020.09.007. Epub 2020 Sep 18. PMID: 32994068.

107: López-Cepero A, O'Neill J, Tamez M, Falcón LM, Tucker KL, Rodríguez-Orengo JF, Mattei J. Associations Between Perceived Stress and Dietary Intake in Adults in Puerto Rico. J Acad Nutr Diet. 2021 Apr;121(4):762-769. doi: 10.1016/j.jand.2020.09.035. Epub 2020 Oct 24. PMID: 33109502; PMCID: PMC7981238.

108: Lee SD, Kellow NJ, Choi TST, Huggins CE. Assessment of Dietary Acculturation in East Asian Populations: A Scoping Review. Adv Nutr. 2021 Jun 1;12(3):865-886. doi: 10.1093/advances/nmaa127. PMID: 33119743; PMCID: PMC8166541.

109: Eicher-Miller HA, Prapkree L, Palacios C. Expanding the Capabilities of Nutrition Research and Health Promotion Through Mobile-Based Applications. Adv Nutr. 2021 Jun 1;12(3):1032-1041. doi: 10.1093/advances/nmab022. PMID: 33734305; PMCID: PMC8166539.

110: Jakše B, Jakše B, Godnov U, Pinter S. Nutritional, Cardiovascular Health and Lifestyle Status of 'Health Conscious' Adult Vegans and Non-Vegans from Slovenia: A Cross-Sectional Self-Reported Survey. Int J Environ Res Public Health. 2021 Jun 2;18(11):5968. doi: 10.3390/ijerph18115968. PMID: 34199550; PMCID: PMC8199727.

111: Wang YY, Tian T, Pan D, Zhang JX, Xie W, Wang SK, Xia H, Dai Y, Sun G. The relationship between dietary patterns and overweight and obesity among adult in Jiangsu Province of China: a structural equation model. BMC Public Health. 2021 Jun 25;21(1):1225. doi: 10.1186/s12889-021-11341-3. PMID: 34172040; PMCID: PMC8229268.

112: Vassou C, Yannakoulia M, Georgousopoulou EN, Pitsavos C, Cropley M, Panagiotakos DB. Foods, Nutrients and Dietary Patterns in Relation to Irrational Beliefs and Related Psychological Disorders: The ATTICA Epidemiological Study. Nutrients. 2021 Apr 27;13(5):1472. doi: 10.3390/nu13051472. PMID: 33925406; PMCID: PMC8146573.

113: Sullivan VK, Johnston EA, Firestone MJ, Yi SS, Beasley JM. Self-Rated Diet Quality and Cardiometabolic Health Among U.S. Adults, 2011-2018. Am J Prev Med. 2021 Oct;61(4):563-575. doi: 10.1016/j.amepre.2021.04.033. Epub 2021 Jul 8. PMID: 34246527; PMCID: PMC8523030.

114: Therrien AS, Buffa G, Roome AB, Standard E, Pomer A, Obed J, Taleo G, Tarivonda L, Chan CW, Kaneko A, Olszowy KM, Dancause KN. Relationships between mental health and diet during pregnancy and birth outcomes in a lower-middle income country: "Healthy mothers, healthy communities" study in Vanuatu. Am J Hum Biol. 2021 May;33(3):e23500. doi: 10.1002/ajhb.23500. Epub 2020 Sep 11. PMID: 32918311.

115: Kisaakye S, Matovu N, Guwatudde D, Kajjura R. Using dietary serving scores to assess adequacy of dietary intake and associated factors among adult patients with type 2 diabetes in Kampala: a cross-sectional study. Eur J Clin Nutr. 2021 Mar;75(3):555-563. doi: 10.1038/s41430-020-00731-2. Epub 2020 Aug 28. PMID: 32859987.

116: Yeh KL, Kautz A, Lohse B, Groth SW. Associations between Dietary Patterns and Inflammatory Markers during Pregnancy: A Systematic Review. Nutrients. 2021 Mar 4;13(3):834. doi: 10.3390/nu13030834. PMID: 33806342; PMCID: PMC8000934.

117: Kingsnorth J, Cushen SJ, Janiszewska K, Avery A. Health professionals' knowledge, views and advice on diet and dental health: a survey of UK and Ireland dietitians and dentists. J Hum Nutr Diet. 2021 Aug;34(4):705-714. doi: 10.1111/jhn.12842. Epub 2021 Jan 7. PMID: 33411983.

118: Kotronia E, Brown H, Papacosta AO, Lennon LT, Weyant RJ, Whincup PH, Wannamethee SG, Ramsay SE. Poor oral health and the association with diet quality and intake in older people in two studies in the UK and USA. Br J Nutr. 2021 Jul 14;126(1):118-130. doi: 10.1017/S0007114521000180. Epub 2021 Jan 20. Erratum in: Br J Nutr. 2021 Jul 14;126(1):160. PMID: 33468264; PMCID: PMC8187263.

119: El Sabry MI, Stino FKR, El-Ghany WAA. Copper: benefits and risks for poultry, livestock, and fish production. Trop Anim Health Prod. 2021 Sep 29;53(5):487. doi: 10.1007/s11250-021-02915-9. PMID: 34590182.

120: Ko GJ, Kalantar-Zadeh K. How important is dietary management in chronic kidney disease progression? A role for low protein diets. Korean J Intern Med. 2021 Jul;36(4):795-806. doi: 10.3904/kjim.2021.197. Epub 2021 Jun 22. PMID: 34153180; PMCID: PMC8273814.

121: Qin C, Lv J, Yu C, Guo Y, Bian Z, Gao M, Du H, Yang L, Chen Y, Shen L, Zhou S, Chen J, Chen Z, Li L. Dietary patterns and cardiometabolic diseases in 0.5 million Chinese adults: a 10-year cohort study. Nutr J. 2021 Sep 3;20(1):74. doi: 10.1186/s12937-021-00730-4. PMID: 34479555; PMCID: PMC8418004.

122: Norde MM, Collese TS, Giovannucci E, Rogero MM. A posteriori dietary patterns and their association with systemic low-grade inflammation in adults: a systematic review and meta-analysis. Nutr Rev. 2021 Feb 11;79(3):331-350. doi: 10.1093/nutrit/nuaa010. PMID: 32417914.

123: Bye ZL, Keshavarz P, Lane GL, Vatanparast H. What Role Do Plant-Based Diets Play in Supporting the Optimal Health and Well-being of Canadians? A Scoping Review. Adv Nutr. 2021 Dec 1;12(6):2132-2146. doi: 10.1093/advances/nmab061. PMID: 34049398; PMCID: PMC8634516.

124: Azizi-Soleiman F, Khoshhali M, Heidari-Beni M, Qorbani M, Kelishadi R. Association between Dietary Antioxidant Quality Score and Anthropometric Measurements in Children and Adolescents: The Weight Disorders Survey of the CASPIAN-IV Study. J Trop Pediatr. 2021 Jul 2;67(3):fmaa065. doi: 10.1093/tropej/fmaa065. PMID: 32844219.

125: Hart MJ, Torres SJ, McNaughton SA, Milte CM. Dietary patterns and associations with biomarkers of inflammation in adults: a systematic review of observational studies. Nutr J. 2021 Mar 12;20(1):24. doi: 10.1186/s12937-021-00674-9. PMID: 33712009; PMCID: PMC7955619.

126: Klonizakis M, Bugg A, Hunt B, Theodoridis X, Bogdanos DP, Grammatikopoulou MG. Assessing the Physiological Effects of Traditional Regional Diets Targeting the Prevention of Cardiovascular Disease: A Systematic Review of Randomized Controlled Trials Implementing Mediterranean, New Nordic, Japanese, Atlantic, Persian and Mexican Dietary Interventions. Nutrients. 2021 Aug 30;13(9):3034. doi: 10.3390/nu13093034. PMID: 34578911; PMCID: PMC8466163.

127: Madzorera I, Ismail A, Hemler EC, Korte ML, Olufemi AA, Wang D, Assefa N, Workneh F, Lankoande B, Chukwu A, Ourohire M, Mattei J, Soura A, Berhane Y, Sie A, Oduola A, Fawzi WW. Impact of COVID-19 on Nutrition, Food Security, and Dietary Diversity and Quality in Burkina Faso, Ethiopia and Nigeria. Am J Trop Med Hyg. 2021 Jun 23;105(2):295-309. doi: 10.4269/ajtmh.20-1617. PMID: 34161300; PMCID: PMC8437159.

128: Kebbe M, Sparks JR, Flanagan EW, Redman LM. Beyond weight loss: current perspectives on the impact of calorie restriction on healthspan and lifespan. Expert Rev Endocrinol Metab. 2021 May;16(3):95-108. doi: 10.1080/17446651.2021.1922077. Epub 2021 May 7. PMID: 33957841.

129: Hu S, Lv Z, Xiang Q, Wang Y, Shen J, Ke Y. Dietary Factors of <i>bla</i>_{NDM} Carriage in Health Community Population: A Cross-Sectional Study. Int J Environ Res Public Health. 2021 Jun 2;18(11):5959. doi: 10.3390/ijerph18115959. PMID: 34199383; PMCID: PMC8199633.

130: Zhao X, Xu X, Li X, He X, Yang Y, Zhu S. Emerging trends of technology-based dietary assessment: a perspective study. Eur J Clin Nutr. 2021 Apr;75(4):582-587. doi: 10.1038/s41430-020-00779-0. Epub 2020 Oct 20. PMID: 33082535.

131: Zhao F, He L, Zhao L, Guo Q, Yu D, Ju L, Fang H. The Status of Dietary Energy and Nutrients Intakes among Chinese Elderly Aged 80 and Above: Data from the CACDNS 2015. Nutrients. 2021 May 12;13(5):1622. doi: 10.3390/nu13051622. PMID: 34066082; PMCID: PMC8150709.

132: Breuninger TA, Wawro N, Breuninger J, Reitmeier S, Clavel T, Six-Merker J, Pestoni G, Rohrmann S, Rathmann W, Peters A, Grallert H, Meisinger C, Haller D, Linseisen J. Associations between habitual diet, metabolic disease, and the gut microbiota using latent Dirichlet allocation. Microbiome. 2021 Mar 16;9(1):61. doi: 10.1186/s40168-020-00969-9. PMID: 33726846; PMCID: PMC7967986.

133: Penhaligan J, Poppitt SD, Miles-Chan JL. The Role of Bovine and Non-Bovine Milk in Cardiometabolic Health: Should We Raise the "Baa"? Nutrients. 2022 Jan 11;14(2):290. doi: 10.3390/nu14020290. PMID: 35057470; PMCID: PMC8780791.

134: Zhang Q, Wolf RL, Lee AR, Catassi C, Zybert P, Green PH, Lebwohl B. Navigating celiac disease and the gluten-free diet in China. Nutr Health. 2021 Dec;27(4):395-403. doi: 10.1177/0260106021990254. Epub 2021 Apr 11. PMID: 33843325.

135: Patterson E, Eustachio Colombo P, Milner J, Green R, Elinder LS. Potential health impact of increasing adoption of sustainable dietary practices in Sweden. BMC Public Health. 2021 Jul 6;21(1):1332. doi: 10.1186/s12889-021-11256-z. PMID: 34229654; PMCID: PMC8261973.

136: Altun E, Walther C, Borof K, Petersen E, Lieske B, Kasapoudis D, Jalilvand N, Beikler T, Jagemann B, Zyriax BC, Aarabi G. Association between Dietary Pattern and Periodontitis-A Cross-Sectional Study. Nutrients. 2021 Nov 21;13(11):4167. doi: 10.3390/nu13114167. PMID: 34836422; PMCID: PMC8621734.

137: Mozaffari H, Hosseini Z, Lafrenière J, Conklin AI. The role of dietary diversity in preventing metabolic-related outcomes: Findings from a systematic review. Obes Rev. 2021 Jun;22(6):e13174. doi: 10.1111/obr.13174. Epub 2021 Feb 21. PMID: 33615679.

138: Morrill KE, Bland VL, Klimentidis YC, Hingle MD, Thomson CA, Garcia DO. Assessing Interactions between <i>PNPLA3</i> and Dietary Intake on Liver Steatosis in Mexican-Origin Adults. Int J Environ Res Public Health. 2021 Jul 1;18(13):7055. doi: 10.3390/ijerph18137055. PMID: 34280991; PMCID: PMC8296936.

139: Guz M, Jeleniewicz W, Malm A, Korona-Glowniak I. A Crosstalk between Diet, Microbiome and microRNA in Epigenetic Regulation of Colorectal Cancer. Nutrients. 2021 Jul 15;13(7):2428. doi: 10.3390/nu13072428. PMID: 34371938; PMCID: PMC8308570.

140: Clancy AK, Lee C, Hamblin H, Gunaratne AW, LeBusque A, Beck EJ, Dawson MV, Borody TJ. Dietary Intakes of Recipients of Faecal Microbiota Transplantation: An Observational Pilot Study. Nutrients. 2021 Apr 28;13(5):1487. doi: 10.3390/nu13051487. PMID: 33924834; PMCID: PMC8147000.

141: Haeri F, Pourmasoumi M, Ghiasvand R, Feizi A, Salehi-Abargouei A, Marvast LD, Clark CCT, Mirzaei M. The relationship between major dietary patterns and fertility status in iranian men: a case-control study. Sci Rep. 2021 Sep

22;11(1):18861. doi: 10.1038/s41598-021-98355-4. PMID: 34552156; PMCID: PMC8458458.

142: Li A, Chen Y, Schuller AA, van der Sluis LWM, Tjakkes GE. Dietary inflammatory potential is associated with poor periodontal health: A population-based study. J Clin Periodontol. 2021 Jul;48(7):907-918. doi: 10.1111/jcpe.13472. Epub 2021 May 7. PMID: 33899265; PMCID: PMC8251843.

143: Matsunaga T, Nishikawa K, Adachi T, Yasuda K. Associations between dietary consumption and sleep quality in young Japanese males. Sleep Breath. 2021 Mar;25(1):199-206. doi: 10.1007/s11325-020-02077-2. Epub 2020 May 8. PMID: 32385731.

144: Stoica C, Cox G. Old problems and new solutions: antibiotic alternatives in food animal production. Can J Microbiol. 2021 Jun;67(6):427-444. doi: 10.1139/cjm-2020-0601. Epub 2021 Feb 19. PMID: 33606564.

145: Davis JA, Mohebbi M, Collier F, Loughman A, Staudacher H, Shivappa N, Hébert JR, Pasco JA, Jacka FN. The role of diet quality and dietary patterns in predicting muscle mass and function in men over a 15-year period. Osteoporos Int. 2021 Nov;32(11):2193-2203. doi: 10.1007/s00198-021-06012-3. Epub 2021 May 27. PMID: 34043032; PMCID: PMC8155648.

146: Wang Y, Nguyen LH, Mehta RS, Song M, Huttenhower C, Chan AT. Association Between the Sulfur Microbial Diet and Risk of Colorectal Cancer. JAMA Netw Open. 2021 Nov 1;4(11):e2134308. doi: 10.1001/jamanetworkopen.2021.34308. PMID: 34767023; PMCID: PMC8590167.

147: González N, Marquès M, Domingo JL. Respiratory viruses in foods and their potential transmission through the diet: A review of the literature. Environ Res. 2021 Apr;195:110826. doi: 10.1016/j.envres.2021.110826. Epub 2021 Jan 30. PMID: 33529649; PMCID: PMC7963685.

148: Wiedeman AM, Panagiotopoulos C, Devlin AM. Treatment-related weight gain and metabolic complications in children with mental health disorders: potential role for lifestyle interventions. Appl Physiol Nutr Metab. 2021 Mar;46(3):193-204. doi: 10.1139/apnm-2020-0259. Epub 2020 Nov 23. PMID: 33226841.

149: Kim HS, Lee H, Provido SMP, Kang M, Chung GH, Hong S, Yu SH, Lee CB, Lee JE. Association Between Diet Quality and Prevalence of Obesity, Dyslipidemia, and Insulin Resistance Among Filipino Immigrant Women in Korea: The Filipino Women's Diet and Health Study. Front Public Health. 2021 Jul 1;9:647661. doi: 10.3389/fpubh.2021.647661. PMID: 34277535; PMCID: PMC8281297.

150: Silva-Santos T, Moreira P, Rodrigues M, Padrão P, Pinho O, Norton P, Ndrio A, Gonçalves C. Interventions That Successfully Reduced Adults Salt Intake-A Systematic Review. Nutrients. 2021 Dec 21;14(1):6. doi: 10.3390/nu14010006. PMID: 35010883; PMCID: PMC8746410.

151: Chung MG, Li Y, Liu J. Global red and processed meat trade and non-communicable diseases. BMJ Glob Health. 2021 Nov;6(11):e006394. doi: 10.1136/bmjgh-2021-006394. PMID: 34782356; PMCID: PMC8559104.

152: Mokhtari E, Farhadnejad H, Salehi-Sahlabadi A, Najibi N, Azadi M, Teymoori F, Mirmiran P. Spinach consumption and nonalcoholic fatty liver disease among adults: a case-control study. BMC Gastroenterol. 2021 May 1;21(1):196. doi: 10.1186/s12876-021-01784-8. PMID: 33933019; PMCID: PMC8088717.

153: Zhang Y, Wang Y, Chen Y, Zhou J, Xu L, Xu K, Wang N, Fu C, Liu T. Associations of Dietary Patterns and Risk of Hypertension in Southwest China: A Prospective Cohort Study. Int J Environ Res Public Health. 2021 Nov 25;18(23):12378. doi: 10.3390/ijerph182312378. PMID: 34886102; PMCID: PMC8656527.

154: Koponen KK, Salosensaari A, Ruuskanen MO, Havulinna AS, Männistö S, Jousilahti P, Palmu J, Salido R, Sanders K, Brennan C, Humphrey GC, Sanders JG, Meric G, Cheng S, Inouye M, Jain M, Niiranen TJ, Valsta LM, Knight R, Salomaa VV. Associations of healthy food choices with gut microbiota profiles. Am J Clin Nutr. 2021 Aug 2;114(2):605-616. doi: 10.1093/ajcn/nqab077. PMID: 34020448; PMCID: PMC8326043.

155: Hu Y, Li Y, Sampson L, Wang M, Manson JE, Rimm E, Sun Q. Lignan Intake and Risk of Coronary Heart Disease. J Am Coll Cardiol. 2021 Aug 17;78(7):666-678. doi: 10.1016/j.jacc.2021.05.049. PMID: 34384548; PMCID: PMC8432598.

156: Jafri A, Mathe N, Aglago EK, Konyole SO, Ouedraogo M, Audain K, Zongo U, Laar AK, Johnson J, Sanou D. Food availability, accessibility and dietary practices during the COVID-19 pandemic: a multi-country survey. Public Health Nutr. 2021 May;24(7):1798-1805. doi: 10.1017/S1368980021000987. Epub 2021 Mar 5. PMID: 33663623; PMCID: PMC8007937.

157: Conradie C, Baumgartner J, Malan L, Symington EA, Cockeran M, Smuts CM, Faber M. A Priori and a Posteriori Dietary Patterns among Pregnant Women in Johannesburg, South Africa: The NuPED Study. Nutrients. 2021 Feb 9;13(2):565. doi: 10.3390/nu13020565. PMID: 33572105; PMCID: PMC7914963.

158: DeMayo F, Molinsky R, Tahir MJ, Roy S, Genkinger JM, Papapanou PN, Jacobs DR Jr, Demmer RT. Diet quality and periodontal disease: Results from the oral infections, glucose intolerance and insulin resistance study (ORIGINS). J Clin Periodontol. 2021 May;48(5):638-647. doi: 10.1111/jcpe.13450. Epub 2021 Mar 12. PMID: 33710636; PMCID: PMC8084984.

159: Mei Y, Wang S, Pang X, Xu J, Li R, Wang Y, Zhao W, Xu T. [Relationship between dietary behavior and malnutrition of children aged 3-5 years in five cities of China]. Wei Sheng Yan Jiu. 2021 Nov;50(6):909-913. Chinese. doi: 10.19813/j.cnki.weishengyanjiu.2021.06.006. PMID: 34949315.

160: Pruvost-Couvreur M, Béchaux C, Rivière G, Le Bizec B. Impact of sociodemographic profile, generation and bioaccumulation on lifetime dietary and internal exposures to PCBs. Sci Total Environ. 2021 Dec 15;800:149511. doi: 10.1016/j.scitotenv.2021.149511. Epub 2021 Aug 9. PMID: 34392223.

161: Poulain T, Spielau U, Vogel M, Dathan-Stumpf A, Körner A, Kiess W. Changes in diet from pregnancy to one year after birth: a longitudinal study. BMC Pregnancy Childbirth. 2021 Sep 4;21(1):600. doi: 10.1186/s12884-021-04038-3. PMID: 34481457; PMCID: PMC8418026.

162: Hochmayr C, Ndayisaba JP, Gande N, Staudt A, Bernar B, Stock K, Geiger R, Knoflach M, Kiechl-Kohlendorfer U; Early Vascular Ageing (EVA) Study Group. Prevalence and differences of ideal cardiovascular health in urban and rural adolescents in the Region of Tyrol: results from the EVA Tyrol study. BMC Cardiovasc Disord. 2021 Jul 13;21(1):338. doi: 10.1186/s12872-021-02156-6. PMID: 34256716; PMCID: PMC8276470.

163: LoBuono DL, Shea KS, Tovar A, Leedahl SN, Mahler L, Xu F, Lofgren IE. Diet Quality and Nutrition Concerns of People with Parkinson's Disease and Their Informal Caregivers: A Mixed Methods Study. J Nutr Gerontol Geriatr. 2022 Jan-Mar;41(1):1-21. doi: 10.1080/21551197.2021.2024478. Epub 2022 Jan 20. PMID: 35048783.

164: Vasmehjani AA, Darabi Z, Nadjarzadeh A, Mirzaei M, Hosseinzadeh M. The relation between dietary phytochemical index and metabolic syndrome and its components in a large sample of Iranian adults: a population-based study. BMC Public Health. 2021 Aug 24;21(1):1587. doi: 10.1186/s12889-021-11590-2. PMID: 34429094; PMCID: PMC8383421.

165: Shikany JM, Safford MM, Soroka O, Brown TM, Newby PK, Durant RW, Judd SE. Mediterranean Diet Score, Dietary Patterns, and Risk of Sudden Cardiac Death in the REGARDS Study. J Am Heart Assoc. 2021 Jul 6;10(13):e019158. doi: 10.1161/JAHA.120.019158. Epub 2021 Jun 30. PMID: 34189926; PMCID: PMC8403280.

166: Veisa V, Kalere I, Zake T, Strele I, Makrecka-Kuka M, Upmale-Engela S, Skesters A, Rezeberga D, Lejnieks A, Pudule I, Grinberga D, Velika B, Dambrova M, Konrade I. Assessment of Iodine and Selenium Nutritional Status in Women of Reproductive Age in Latvia. Medicina (Kaunas). 2021 Nov 5;57(11):1211. doi: 10.3390/medicina57111211. PMID: 34833429; PMCID: PMC8622847.

167: Van Puyvelde H, Papadimitriou N, Clasen J, Muller D, Biessy C, Ferrari P, Halkjær J, Overvad K, Tjønneland A, Fortner RT, Katzke V, Schulze MB, Chiodini P, Masala G, Pala V, Sacerdote C, Tumino R, Bakker MF, Agudo A, Ardanaz E, Chirlaque López MD, Sánchez MJ, Ericson U, Gylling B, Karlsson T, Manjer J, Schmidt JA, Nicolas G, Casagrande C, Weiderpass E, Heath AK, Godderis L, Van Herck K, De Bacquer D, Gunter MJ, Huybrechts I. Dietary Methyl-Group Donor Intake and Breast Cancer Risk in the European Prospective Investigation into Cancer and Nutrition (EPIC). Nutrients. 2021 May 28;13(6):1843. doi: 10.3390/nu13061843. PMID: 34071317; PMCID: PMC8228096.

168: Cortés-Valencia A, Monge A, Tamayo-Ortiz M, Lopez-Ridaura R, Rodriguez BL, Cantú-Brito C, Catzin-Kuhlmann A, Lajous M. Dairy consumption and subclinical atherosclerosis: A cross-sectional study among middle-aged Mexican women. Nutr Metab Cardiovasc Dis. 2021 Jun 7;31(6):1747-1755. doi: 10.1016/j.numecd.2021.02.024. Epub 2021 Mar 2. PMID: 33965300.

169: Nel JH, Steyn NP, Senekal M. Illustration of the Importance of Adjustment for within- and between-Person Variability in Dietary Intake Surveys for Assessment of Population Risk of Micronutrient Deficiency/Excess Using an Example Data Set. Nutrients. 2022 Jan 11;14(2):285. doi: 10.3390/nu14020285. PMID: 35057466; PMCID: PMC8781123.

170: Rollet M, Bohn T, Vahid F, On Behalf Of The Oriscav Working Group. Association between Dietary Factors and Constipation in Adults Living in Luxembourg and Taking Part in the ORISCAV-LUX 2 Survey. Nutrients. 2021 Dec 28;14(1):122. doi: 10.3390/nu14010122. PMID: 35010999; PMCID: PMC8746799.

171: Keaver L, Ruan M, Chen F, Du M, Ding C, Wang J, Shan Z, Liu J, Zhang FF. Plant- and animal-based diet quality and mortality among US adults: a cohort study. Br J Nutr. 2021 Jun 28;125(12):1405-1415. doi: 10.1017/S0007114520003670. Epub 2020 Sep 18. PMID: 32943123; PMCID: PMC8547553.

172: Zheng J, Zhou R, Li F, Chen L, Wu K, Huang J, Liu H, Huang Z, Xu L, Yuan Z, Mao C, Wu X. Association between dietary diversity and cognitive impairment among the oldest-old: Findings from a nationwide cohort study. Clin Nutr. 2021 Apr;40(4):1452-1462. doi: 10.1016/j.clnu.2021.02.041. Epub 2021 Mar 2. PMID: 33740515.

173: Gianfredi V, Koster A, Odone A, Amerio A, Signorelli C, Schaper NC, Bosma H, Köhler S, Dagnelie PC, Stehouwer CDA, Schram MT, Dongen MCJMV, Eussen SJPM. Associations of Dietary Patterns with Incident Depression: The Maastricht Study. Nutrients. 2021 Mar 23;13(3):1034. doi: 10.3390/nu13031034. PMID: 33806882; PMCID: PMC8004955.

174: Quan J, Panaccione N, Jeong J, Underwood FE, Coward S, Windsor JW, Ronksley PE, Gidrewicz D, deBruyn J, Turner JM, Lebwohl B, Kaplan GG, King JA. Association Between Celiac Disease and Autism Spectrum Disorder: A Systematic Review. J Pediatr Gastroenterol Nutr. 2021 May 1;72(5):704-711. doi: 10.1097/MPG.0000000000003051. PMID: 33847288.

175: Głąbska D, Kołota A, Lachowicz K, Skolmowska D, Stachoń M, Guzek D. Vitamin D Supplementation and Mental Health in Inflammatory Bowel Diseases and Irritable Bowel Syndrome Patients: A Systematic Review. Nutrients. 2021 Oct 19;13(10):3662. doi: 10.3390/nu13103662. PMID: 34684663; PMCID: PMC8540769.

176: McParland V, Wilck N. Ernährung und Hypertonie : Worauf man neben der medikamentösen Therapie achten sollte [Nutrition and hypertension : What one should pay attention to in addition to the pharmaceutical treatment]. Internist

(Berl). 2021 Mar;62(3):269-276. German. doi: 10.1007/s00108-021-00988-0. Epub 2021 Feb 16. PMID: 33590293.

177: Ko J, Wang J, Du Y, Jiwani R, Li C. Personalized Behavioral Nutrition Among Older Asian Americans: Study Protocol. Nurs Res. 2021 Jul-Aug 01;70(4):317-322. doi: 10.1097/NNR.0000000000000514. PMID: 34160184; PMCID: PMC8231758.

178: Castellanos-Gutiérrez A, Rodríguez-Ramírez S, Bromage S, Fung TT, Li Y, Bhupathiraju SN, Deitchler M, Willett W, Batis C. Performance of the Global Diet Quality Score with Nutrition and Health Outcomes in Mexico with 24-h Recall and FFQ Data. J Nutr. 2021 Oct 23;151(12 Suppl 2):143S-151S. doi: 10.1093/jn/nxab202. PMID: 34689195; PMCID: PMC8542100.

179: Jun L, Root M. Association of Carotenoid Intake with Pulmonary Function. J Am Coll Nutr. 2021 Nov-Dec;40(8):708-712. doi: 10.1080/07315724.2020.1815608. Epub 2020 Oct 8. PMID: 33030982.

180: Khatun T, Maqbool D, Ara F, Sarker MR, Anwar KS, Hoque A. Dietary habits of patients with coronary artery disease in a tertiary-care hospital of Bangladesh: a case-controlled study. J Health Popul Nutr. 2021 Mar 1;40(1):3. doi: 10.1186/s41043-021-00226-1. Erratum in: J Health Popul Nutr. 2021 Apr 13;40(1):18. PMID: 33648595; PMCID: PMC7919298.

181: Zhang JY, Zhang B, Tang J, Gao C, Dong J, Ren J, Guo X, Xu A. [Deaths and life expectancy losses attributed to high-salt diet in Shandong province]. Zhonghua Liu Xing Bing Xue Za Zhi. 2021 Mar 10;42(3):527-530. Chinese. doi: 10.3760/cma.j.cn112338-20200306-00268. PMID: 34814424.

182: Schüz B, Meyerhof H, Hilz LK, Mata J. Equity Effects of Dietary Nudging Field Experiments: Systematic Review. Front Public Health. 2021 Jul 23;9:668998. doi: 10.3389/fpubh.2021.668998. PMID: 34368049; PMCID: PMC8342848.

183: Engelmann G, Marsall M, Skoda EM, Knoll-Pientka N, Bäuerle L, Stroebele-Benschop N, Teufel M, Bäuerle A. Development and Validation of the General Dietary Behavior Inventory (GDBI) in Scope of International Nutrition Guidelines. Nutrients. 2021 Apr 17;13(4):1328. doi: 10.3390/nu13041328. PMID: 33920515; PMCID: PMC8073993.

184: Akter S, Mizoue T, Nanri A, Goto A, Noda M, Sawada N, Yamaji T, Iwasaki M, Inoue M, Tsugane S; Japan Public Health Center-based Prospective Study Group. Low carbohydrate diet and all cause and cause-specific mortality. Clin Nutr. 2021 Apr;40(4):2016-2024. doi: 10.1016/j.clnu.2020.09.022. Epub 2020 Sep 23. PMID: 33046262.

185: Shoesmith A, Hall A, Wolfenden L, Shelton RC, Powell BJ, Brown H, McCrabb S, Sutherland R, Yoong S, Lane C, Booth D, Nathan N. Barriers and facilitators influencing the sustainment of health behaviour interventions in schools and childcare services: a systematic review. Implement Sci. 2021 Jun 12;16(1):62. doi: 10.1186/s13012-021-01134-y. PMID: 34118955; PMCID: PMC8199827.

186: Babashahi M, Omidvar N, Yazdizadeh B, Heidari-Beni M, Joulaei H, Narmcheshm S, Zargaraan A, Kelishadi R. Systematic review and meta-analysis of the most common processed foods consumed by Iranian children. East Mediterr Health J. 2021 Sep 21;27(9):918-930. doi: 10.26719/emhj.21.032. PMID: 34569048.

187: Baid D, Hayles E, Finkelstein EA. Return on Investment of Workplace Wellness Programs for Chronic Disease Prevention: A Systematic Review. Am J Prev Med. 2021 Aug;61(2):256-266. doi: 10.1016/j.amepre.2021.02.002. Epub 2021 May 5. PMID: 33965267.

188: Landgraf-Rauf K, von Mutius E. Effective Ways to Prevent Allergic Diseases: Where Do We Stand? Handb Exp Pharmacol. 2022;268:437-448. doi: 10.1007/164_2021_497. PMID: 34196812.

189: Zhang YB, Chen JX, Jiang YW, Xia PF, Pan A. Association of sugar-sweetened beverage and artificially sweetened beverage intakes with mortality: an analysis of US National Health and Nutrition Examination Survey. Eur J Nutr. 2021 Jun;60(4):1945-1955. doi: 10.1007/s00394-020-02387-x. Epub 2020 Sep 18. PMID: 32945955.

190: Otsuka R, Nishita Y, Nakamura A, Kato T, Iwata K, Tange C, Tomida M, Kinoshita K, Nakagawa T, Ando F, Shimokata H, Arai H. Dietary diversity is associated with longitudinal changes in hippocampal volume among Japanese community dwellers. Eur J Clin Nutr. 2021 Jun;75(6):946-953. doi: 10.1038/s41430-020-00734-z. Epub 2020 Sep 2. PMID: 32879451; PMCID: PMC8189902.

191: Choi Y, Larson N, Steffen LM, Schreiner PJ, Gallaher DD, Duprez DA, Shikany JM, Rana JS, Jacobs DR Jr. Plant-Centered Diet and Risk of Incident Cardiovascular Disease During Young to Middle Adulthood. J Am Heart Assoc. 2021 Aug 17;10(16):e020718. doi: 10.1161/JAHA.120.020718. Epub 2021 Aug 4. PMID: 34344159; PMCID: PMC8475033.

192: Currenti W, Buscemi S, Cincione RI, Cernigliaro A, Godos J, Grosso G, Galvano F. Time-Restricted Feeding and Metabolic Outcomes in a Cohort of Italian Adults. Nutrients. 2021 May 13;13(5):1651. doi: 10.3390/nu13051651. PMID: 34068302; PMCID: PMC8153259.

193: Wang ZQ, Zhang L, Zheng H, Guo WB, Gao Y, Zhao YF, Liu DW, Zhou MG, Li M. Burden and trend of ischemic heart disease and colorectal cancer attributable to a diet low in fiber in China, 1990-2017: findings from the Global Burden of Disease Study 2017. Eur J Nutr. 2021 Oct;60(7):3819-3827. doi: 10.1007/s00394-021-02556-6. Epub 2021 Apr 14. PMID: 33852070.

194: Sezavar H, Yousefi R, Abbasi M, Safari S, Mottaghi A. Anthropometric and Biochemical Measures in Bariatric Surgery Candidates: What Is the Role of Inflammatory Potential of Diet? Obes Surg. 2021 Jul;31(7):3097-3108. doi: 10.1007/s11695-021-05345-6. Epub 2021 Mar 22. PMID: 33751340.

195: Costa de Miranda R, Rauber F, de Moraes MM, Afonso C, Santos C, Rodrigues S, Levy RB; UPPER Group. Consumption of ultra-processed foods and non-communicable disease-related nutrient profile in Portuguese adults and elderly (2015-2016): the UPPER project. Br J Nutr. 2021 May 28;125(10):1177-1187. doi: 10.1017/S000711452000344X. Epub 2020 Sep 3. PMID: 32878664.

196: Duan R, Qiao T, Chen Y, Chen M, Xue H, Zhou X, Yang M, Liu Y, Zhao L, Libuda L, Cheng G. The overall diet quality in childhood is prospectively associated with the timing of puberty. Eur J Nutr. 2021 Aug;60(5):2423-2434. doi: 10.1007/s00394-020-02425-8. Epub 2020 Nov 2. PMID: 33140158; PMCID: PMC8275527.

197: Auchincloss AH, Li J, Moore KA, Franco M, Mujahid MS, Moore LV. Are neighbourhood restaurants related to frequency of restaurant meals and dietary quality? Prevalence and changes over time in the Multi-Ethnic Study of Atherosclerosis. Public Health Nutr. 2021 Oct;24(14):4630-4641. doi: 10.1017/S1368980021002196. Epub 2021 May 25. PMID: 34030763; PMCID: PMC8688147.

198: Baraldi LG, Steele EM, Louzada MLC, Monteiro CA. Associations between ultraprocessed food consumption and total water intake in the US population. J Acad Nutr Diet. 2021 Sep;121(9):1695-1703. doi: 10.1016/j.jand.2021.02.011. Epub 2021 Mar 18. PMID: 33745880.

199: Abdullah MMH, Hughes J, Grafenauer S. Healthcare Cost Savings Associated with Increased Whole Grain Consumption among Australian Adults. Nutrients. 2021 May 29;13(6):1855. doi: 10.3390/nu13061855. PMID: 34072326; PMCID: PMC8228843.

200: Inanir D, Kaelin I, Pestoni G, Faeh D, Mueller N, Rohrmann S, Sych J. Daily and meal-based assessment of dairy and corresponding protein intake in Switzerland: results from the National Nutrition Survey menuCH. Eur J Nutr. 2021 Jun;60(4):2099-2109. doi: 10.1007/s00394-020-02399-7. Epub 2020 Oct 8. PMID: 33030578; PMCID: PMC8137467.

201: Adom T, De Villiers A, Puoane T, Kengne AP. A Scoping Review of Policies Related to the Prevention and Control of Overweight and Obesity in Africa. Nutrients. 2021 Nov 11;13(11):4028. doi: 10.3390/nu13114028. PMID: 34836281; PMCID: PMC8625107.

202: Mohsenian S, Shabbidar S, Siassi F, Qorbani M, Khosravi S, Abshirini M, Aslani Z, Sotoudeh G. Carbohydrate quality index: Its relationship to menopausal symptoms in postmenopausal women. Maturitas. 2021 Aug;150:42-48. doi: 10.1016/j.maturitas.2021.05.006. Epub 2021 May 29. PMID: 34274075.

203: Zhang H, Zeng Y, Yang H, Hu Y, Hu Y, Chen W, Ying Z, Sun Y, Qu Y, Li Q, Valdimarsdóttir UA, Song H. Familial factors, diet, and risk of cardiovascular disease: a cohort analysis of the UK Biobank. Am J Clin Nutr. 2021 Nov 8;114(5):1837-1846. doi: 10.1093/ajcn/nqab261. PMID: 34375391.

204: Hobbs-Grimmer DA, Givens DI, Lovegrove JA. Associations between red meat,

processed red meat and total red and processed red meat consumption, nutritional adequacy and markers of health and cardio-metabolic diseases in British adults: a cross-sectional analysis using data from UK National Diet and Nutrition Survey. Eur J Nutr. 2021 Sep;60(6):2979-2997. doi: 10.1007/s00394-021-02486-3. Epub 2021 Feb 7. PMID: 33554272; PMCID: PMC8354925.

205: Scarpa G, Berrang-Ford L, Bawajeeh AO, Twesigomwe S, Kakwangire P, Peters R, Beer S, Williams G, Zavaleta-Cortijo C, Namanya DB, Lwasa S, Nowembabazi E, Kesande C, Rippin H; IHACC Team, Cade JE. Developing an online food composition database for an Indigenous population in south-western Uganda. Public Health Nutr. 2021 Jun;24(9):2455-2464. doi: 10.1017/S1368980021001397. Epub 2021 Apr 12. PMID: 33843552; PMCID: PMC8145457.

206: Alzaben AS, Alnashwan NI, Alatr AA, Alneghamshi NA, Alhashem AM. Effectiveness of a nutrition education and intervention programme on nutrition knowledge and dietary practice among Princess Nourah Bint Abdulrahman University's population. Public Health Nutr. 2021 May;24(7):1854-1860. doi: 10.1017/S1368980021000604. Epub 2021 Feb 10. PMID: 33563363.

207: Wieser H, Ruiz-Carnicer Á, Segura V, Comino I, Sousa C. Challenges of Monitoring the Gluten-Free Diet Adherence in the Management and Follow-Up of Patients with Celiac Disease. Nutrients. 2021 Jun 30;13(7):2274. doi: 10.3390/nu13072274. PMID: 34209138; PMCID: PMC8308436.

208: Currenti W, Godos J, Castellano S, Caruso G, Ferri R, Caraci F, Grosso G, Galvano F. Time-restricted feeding is associated with mental health in elderly Italian adults. Chronobiol Int. 2021 Oct;38(10):1507-1516. doi: 10.1080/07420528.2021.1932998. Epub 2021 Jun 8. PMID: 34100325.

209: Stosovic D, Vasiljevic N, Jovanovic V, Cirkovic A, Paunovic K, Davidovic D. Dietary Habits of Older Adults in Serbia: Findings From the National Health Survey. Front Public Health. 2021 Aug 23;9:610873. doi: 10.3389/fpubh.2021.610873. PMID: 34497788; PMCID: PMC8419354.

210: Kavyani M, Saleh-Ghadimi S, Dehghan P, Abbasalizad Farhangi M, Khoshbaten M. Co-supplementation of camelina oil and a prebiotic is more effective for in improving cardiometabolic risk factors and mental health in patients with NAFLD: a randomized clinical trial. Food Funct. 2021 Sep 20;12(18):8594-8604. doi: 10.1039/d1fo00448d. PMID: 34338703.

211: Ludwig-Borycz E, Guyer HM, Aljahdali AA, Baylin A. Organic food consumption is associated with inflammatory biomarkers among older adults. Public Health Nutr. 2021 Oct;24(14):4603-4613. doi: 10.1017/S1368980020005236. Epub 2020 Dec 23. PMID: 33353578.

212: Kurnik-Łucka M, Pasieka P, Łączak P, Rząsa-Duran E, Gil K. Advancing European nutrition - are pharmacists eligible partners in the process? Eur J Clin Nutr. 2021 Sep;75(9):1349-1358. doi: 10.1038/s41430-020-00846-6. Epub 2021 Feb 2. PMID: 33531633; PMCID: PMC7851801.

213: Mazzucca CB, Raineri D, Cappellano G, Chiocchetti A. How to Tackle the Relationship between Autoimmune Diseases and Diet: Well Begun Is Half-Done. Nutrients. 2021 Nov 5;13(11):3956. doi: 10.3390/nu13113956. PMID: 34836210; PMCID: PMC8620243.

214: Mao Z, Aglago EK, Zhao Z, Schalkwijk C, Jiao L, Freisling H, Weiderpass E, Hughes DJ, Eriksen AK, Tjønneland A, Severi G, Rothwell J, Boutron-Ruault MC, Katzke V, Kaaks R, Schulze MB, Birukov A, Krogh V, Panico S, Tumino R, Ricceri F, Bueno-de-Mesquita HB, Vermeulen RCH, Gram IT, Skeie G, Sandanger TM, Quirós JR, Crous-Bou M, Sánchez MJ, Amiano P, Chirlaque MD, Barricarte Gurrea A, Manjer J, Johansson I, Perez-Cornago A, Jenab M, Fedirko V. Dietary Intake of Advanced Glycation End Products (AGEs) and Mortality among Individuals with Colorectal Cancer. Nutrients. 2021 Dec 10;13(12):4435. doi: 10.3390/nu13124435. PMID: 34959986; PMCID: PMC8704988.

215: Carubbi F, Alunno A, Mai F, Mercuri A, Centorame D, Cipolloni J, Mariani FM, Rossi M, Bartoloni E, Grassi D, Ferri C. Adherence to the Mediterranean diet and the impact on clinical features in primary Sjögren's syndrome. Clin Exp Rheumatol. 2021 Nov-Dec;39 Suppl 133(6):190-196. Epub 2021 Nov 29. PMID: 34874828.

216: Barcus GC, Papathakis PC, Schaffner A, Chimera B. Nutrition Screening, Reported Dietary Intake, Hospital Foods, and Malnutrition in Critical Care Patients in Malawi. Nutrients. 2021 Apr 1;13(4):1170. doi: 10.3390/nu13041170. PMID: 33916149; PMCID: PMC8066941.

217: Full KM, Berger AT, Erickson D, Berry KM, Laska MN, Lenk KM, Iber C, Redline S, Widome R. Assessing Changes in Adolescents' Sleep Characteristics and Dietary Quality in the START Study, a Natural Experiment on Delayed School Start Time Policies. J Nutr. 2021 Sep 4;151(9):2808-2815. doi: 10.1093/jn/nxab169. PMID: 34087941; PMCID: PMC8417921.

218: Crane MM, Halloway S, Walts ZL, Gavin KL, Moss A, Westrick JC, Appelhans BM. Behavioural interventions for CVD risk reduction for blue-collar workers: a systematic review. J Epidemiol Community Health. 2021 Dec;75(12):1236-1243. doi: 10.1136/jech-2021-216515. Epub 2021 Jul 28. PMID: 34321281; PMCID: PMC8595631.

219: McLaren-Hedwards T, Hickman IJ, Campbell KL, Macdonald GA, Mayr HL. A Qualitative Study of Clinician Barriers and Enablers to Implementing the Mediterranean Dietary Pattern with Kidney and Liver Transplant Recipients. Prog Transplant. 2021 Dec;31(4):337-344. doi: 10.1177/15269248211046001. Epub 2021 Nov 2. PMID: 34726088.

220: Gonzalez AE, Waldman HS, Abel MG, McCurdy KW, McAllister MJ. Impact of Time Restricted Feeding on Fitness Variables in Professional Resistance Trained Firefighters. J Occup Environ Med. 2021 Apr 1;63(4):343-349. doi: 10.1097/JOM.0000000000002144. PMID: 33769400.

221: Fraiz GM, da Conceição AR, de Souza Vilela DL, Rocha DMUP, Bressan J, Hermsdorff HHM. Can resveratrol modulate sirtuins in obesity and related diseases? A systematic review of randomized controlled trials. Eur J Nutr. 2021 Sep;60(6):2961-2977. doi: 10.1007/s00394-021-02623-y. Epub 2021 Jul 12. PMID: 34251517.

222: Huang Q, Jia X, Zhang J, Huang F, Wang H, Zhang B, Wang L, Jiang H, Wang Z. Diet-Cognition Associations Differ in Mild Cognitive Impairment Subtypes. Nutrients. 2021 Apr 17;13(4):1341. doi: 10.3390/nu13041341. PMID: 33920687; PMCID: PMC8073801.

223: Kaesler N, Baid-Agrawal S, Grams S, Nadal J, Schmid M, Schneider MP, Eckardt KU, Floege J, Bergmann MM, Schlieper G, Saritas T. Low adherence to CKD-specific dietary recommendations associates with impaired kidney function, dyslipidemia, and inflammation. Eur J Clin Nutr. 2021 Sep;75(9):1389-1397. doi: 10.1038/s41430-020-00849-3. Epub 2021 Feb 2. PMID: 33531632; PMCID: PMC8416654.

224: Dong Z, Gao X, Chinchilli VM, Sinha R, Muscat J, Winkels R, Richie JP Jr. Association of dietary sulfur amino acid intake with mortality from diabetes and other causes. Eur J Nutr. 2022 Feb;61(1):289-298. doi: 10.1007/s00394-021-02641-w. Epub 2021 Jul 29. PMID: 34327571.

225: Zhao Y, Naumova EN, Bobb JF, Claus Henn B, Singh GM. Joint Associations of Multiple Dietary Components With Cardiovascular Disease Risk: A Machine-Learning Approach. Am J Epidemiol. 2021 Jul 1;190(7):1353-1365. doi: 10.1093/aje/kwab004. PMID: 33521815; PMCID: PMC8245893.

226: Pinart M, Jeran S, Boeing H, Stelmach-Mardas M, Standl M, Schulz H, Harris C, von Berg A, Herberth G, Koletzko S, Linseisen J, Breuninger TA, Nöthlings U, Barbaresko J, Benda S, Lachat C, Yang C, Gasparini P, Robino A, Rojo-Martínez G, Castaño L, Guillaume M, Donneau AF, Hoge A, Gillain N, Avraam D, Burton PR, Bouwman J, Pischon T, Nimptsch K. Dietary Macronutrient Composition in Relation to Circulating HDL and Non-HDL Cholesterol: A Federated Individual-Level Analysis of Cross-Sectional Data from Adolescents and Adults in 8 European Studies. J Nutr. 2021 Aug 7;151(8):2317-2329. doi: 10.1093/jn/nxab077. PMID: 33847346.

227: Fabricius FA, Thomsen ST, Fagt S, Nauta M. The health impact of substituting unprocessed red meat by pulses in the Danish diet. Eur J Nutr. 2021 Sep;60(6):3107-3118. doi: 10.1007/s00394-021-02495-2. Epub 2021 Jan 30. PMID: 33515322.

228: Vilela DLS, Fonseca PG, Pinto SL, Bressan J. Influence of dietary patterns on the metabolically healthy obesity phenotype: A systematic review. Nutr Metab Cardiovasc Dis. 2021 Sep 22;31(10):2779-2791. doi: 10.1016/j.numecd.2021.05.007. Epub 2021 May 26. PMID: 34340900.

229: Bromage S, Batis C, Bhupathiraju SN, Fawzi WW, Fung TT, Li Y, Deitchler M, Angulo E, Birk N, Castellanos-Gutiérrez A, He Y, Fang Y, Matsuzaki M, Zhang Y,

Moursi M, Gicevic S, Holmes MD, Isanaka S, Kinra S, Sachs SE, Stampfer MJ, Stern D, Willett WC. Development and Validation of a Novel Food-Based Global Diet Quality Score (GDQS). J Nutr. 2021 Oct 23;151(12 Suppl 2):75S-92S. doi: 10.1093/jn/nxab244. PMID: 34689200; PMCID: PMC8542096.

230: Wu H, Yuan YQ, Wang YC, Zhou XF, Liu SJ, Cai MQ, He GS, Li SG, Zang JJ, Chen B. The development of a Chinese Healthy Eating Index for School-age Children and its Application in children from China Health and Nutrition Survey. Int J Food Sci Nutr. 2021 Mar;72(2):280-291. doi: 10.1080/09637486.2020.1796930. Epub 2020 Jul 27. PMID: 32718190.

231: Ramos Álvarez O, Arufe Giráldez V, Cantarero Prieto D, Ibáñez García A. Changes in Physical Fitness, Dietary Habits and Family Habits for Spanish Children during SARS-CoV-2 Lockdown. Int J Environ Res Public Health. 2021 Dec 16;18(24):13293. doi: 10.3390/ijerph182413293. PMID: 34948901; PMCID: PMC8701998.

232: Krijger JA, Nicolaou M, Nguyen AN, Voortman T, Hutten BA, Vrijkotte TG. Diet quality at age 5-6 and cardiovascular outcomes in preadolescents. Clin Nutr ESPEN. 2021 Jun;43:506-513. doi: 10.1016/j.clnesp.2021.02.011. Epub 2021 Feb 25. PMID: 34024563.

233: Stefler D, Brett D, Sarkadi-Nagy E, Kopczynska E, Detchev S, Bati A, Scrob M, Koenker D, Aleksov B, Douarin E, Simonova G, Malyutina S, Kubinova R, Pajak A, Ruiz M, Peasey A, Pikhart H, Bobak M. Traditional Eastern European diet and mortality: prospective evidence from the HAPIEE study. Eur J Nutr. 2021 Mar;60(2):1091-1100. doi: 10.1007/s00394-020-02319-9. Epub 2020 Jul 1. PMID: 32613328; PMCID: PMC7900332.

234: Fardet A, Aubrun K, Rock E. Nutrition transition and chronic diseases in China (1990-2019): industrially processed and animal calories rather than nutrients and total calories as potential determinants of the health impact. Public Health Nutr. 2021 Nov;24(16):5561-5575. doi: 10.1017/S1368980021003311. Epub 2021 Aug 11. PMID: 34376266.

235: Witkowska AM, Waśkiewicz A, Zujko ME, Mirończuk-Chodakowska I, Cicha-Mikołajczyk A, Drygas W. Assessment of Plant Sterols in the Diet of Adult Polish Population with the Use of a Newly Developed Database. Nutrients. 2021 Aug 7;13(8):2722. doi: 10.3390/nu13082722. PMID: 34444882; PMCID: PMC8398305.

236: Williams CM, Ashwell M, Prentice A, Hickson M, Stanner S; Academy of Nutrition Sciences. Nature of the evidence base and frameworks underpinning dietary recommendations for prevention of non-communicable diseases: a position paper from the Academy of Nutrition Sciences. Br J Nutr. 2021 Oct 14;126(7):1076-1090. doi: 10.1017/S0007114520005000. Epub 2020 Dec 10. PMID: 34515022.

237: Chen LW, Aubert AM, Shivappa N, Bernard JY, Mensink-Bout SM, Geraghty AA, Mehegan J, Suderman M, Polanska K, Hanke W, Jankowska A, Relton CL, Crozier SR,

Harvey NC, Cooper C, Hanson M, Godfrey KM, Gaillard R, Duijts L, Heude B, Hébert JR, McAuliffe FM, Kelleher CC, Phillips CM. Maternal dietary quality, inflammatory potential and childhood adiposity: an individual participant data pooled analysis of seven European cohorts in the ALPHABET consortium. BMC Med. 2021 Feb 22;19(1):33. doi: 10.1186/s12916-021-01908-7. PMID: 33612114; PMCID: PMC7898733.

238: Mondot S, Poirier P, Abou-Bacar A, Greigert V, Brunet J, Nourrisson C, Randrianarivelojosia M, Razafindrakoto JL, Morel E, Rakotomalala RS, Leclerc M, Le Roux K, Monot C, Lepage P, Candolfi E. Parasites and diet as main drivers of the Malagasy gut microbiome richness and function. Sci Rep. 2021 Sep 3;11(1):17630. doi: 10.1038/s41598-021-96967-4. PMID: 34480059; PMCID: PMC8417078.

239: Strikwerda AJ, Dommershuijsen LJ, Ikram MK, Voortman T. Diet Quality and Risk of Parkinson's Disease: The Rotterdam Study. Nutrients. 2021 Nov 7;13(11):3970. doi: 10.3390/nu13113970. PMID: 34836224; PMCID: PMC8618850.

240: Ali Z, Scheelbeek PFD, Sanin KI, Thomas TS, Ahmed T, Prentice AM, Green R. Characteristics of Distinct Dietary Patterns in Rural Bangladesh: Nutrient Adequacy and Vulnerability to Shocks. Nutrients. 2021 Jun 15;13(6):2049. doi: 10.3390/nu13062049. PMID: 34203961; PMCID: PMC8232582.

241: Cediel G, Reyes M, Corvalán C, Levy RB, Uauy R, Monteiro CA. Ultra-processed foods drive to unhealthy diets: evidence from Chile. Public Health Nutr. 2021 May;24(7):1698-1707. doi: 10.1017/S1368980019004737. Epub 2020 Apr 27. PMID: 32338229.

242: Sisa I, Abeyá-Gilardon E, Fisberg RM, Jackson MD, Mangialavori GL, Sichieri R, Cudhea F, Bannuru RR, Ruthazer R, Mozaffarian D, Singh GM. Impact of diet on CVD and diabetes mortality in Latin America and the Caribbean: a comparative risk assessment analysis. Public Health Nutr. 2021 Jun;24(9):2577-2591. doi: 10.1017/S1368980020000646. Epub 2020 Jun 3. PMID: 32489172; PMCID: PMC7710925.

243: Jennings C, Patterson E, Curtis RG, Mazzacano A, Maher CA. Effectiveness of a Lifestyle Modification Program Delivered under Real-World Conditions in a Rural Setting. Nutrients. 2021 Nov 12;13(11):4040. doi: 10.3390/nu13114040. PMID: 34836296; PMCID: PMC8620632.

244: Karimbeiki R, Alipoor E, Yaseri M, Shivappa N, Hebert JR, Hosseinzadeh-Attar MJ. Association between the dietary inflammatory index and obesity in otherwise healthy adults: Role of age and sex. Int J Clin Pract. 2021 Oct;75(10):e14567. doi: 10.1111/ijcp.14567. Epub 2021 Jul 9. PMID: 34165878.

245: LeCroy MN, Bryant M, Albrecht SS, Siega-Riz AM, Ward DS, Cai J, Stevens J. Obesogenic home food availability, diet, and BMI in Pakistani and White toddlers. Matern Child Nutr. 2021 Jul;17(3):e13138. doi: 10.1111/mcn.13138. Epub 2021 Jan 19. PMID: 33470030; PMCID: PMC8189220.

246: Emmert-Fees KMF, Karl FM, von Philipsborn P, Rehfuess EA, Laxy M. Simulation Modeling for the Economic Evaluation of Population-Based Dietary Policies: A Systematic Scoping Review. Adv Nutr. 2021 Oct 1;12(5):1957-1995. doi: 10.1093/advances/nmab028. PMID: 33873201; PMCID: PMC8483966.

247: Paris JMG, Falkenberg T, Nöthlings U, Heinzel C, Borgemeister C, Escobar N. Changing dietary patterns is necessary to improve the sustainability of Western diets from a One Health perspective. Sci Total Environ. 2022 Mar 10;811:151437. doi: 10.1016/j.scitotenv.2021.151437. Epub 2021 Nov 6. PMID: 34748829.

248: Liu N, Ma F, Feng Y, Ma X. The Association between the Dietary Inflammatory Index and Thyroid Function in U.S. Adult Males. Nutrients. 2021 Sep 23;13(10):3330. doi: 10.3390/nu13103330. PMID: 34684331; PMCID: PMC8540204.

249: Galekop MMJ, Uyl-de Groot CA, Ken Redekop W. A Systematic Review of Cost-Effectiveness Studies of Interventions With a Personalized Nutrition Component in Adults. Value Health. 2021 Mar;24(3):325-335. doi: 10.1016/j.jval.2020.12.006. Epub 2021 Feb 10. PMID: 33641765.

250: Ahmed S, Newton PD, Ojo O, Dibley L. Experiences of ethnic minority patients who are living with a primary chronic bowel condition: a systematic scoping review with narrative synthesis. BMC Gastroenterol. 2021 Aug 18;21(1):322. doi: 10.1186/s12876-021-01857-8. PMID: 34407752; PMCID: PMC8371833.

251: Aslani Z, Bahreynian M, Namazi N, Shivappa N, Hébert JR, Asayesh H, Motlagh ME, Pourmirzaei MA, Kasaeian A, Mahdavi-Gorabi A, Qorbani M, Kelishadi R. Association of dietary acid load with anthropometric indices in children and adolescents. Eat Weight Disord. 2021 Mar;26(2):555-567. doi: 10.1007/s40519-020-00883-x. Epub 2020 Mar 14. PMID: 32172506.

252: Choeda T, Jeyashree K, Kathirvel S, Dorji T, Dorjee K, Tenzin K, Thinley S, Tenzin T, Gurung MS. Dietary behavior of school-going adolescents in Bhutan: Findings from the global school-based student health survey in 2016. Nutrition. 2021 Oct;90:111290. doi: 10.1016/j.nut.2021.111290. Epub 2021 Apr 28. PMID: 34111832.

253: Liu W, Hu B, Dehghan M, Mente A, Wang C, Yan R, Rangarajan S, Tse LA, Yusuf S, Liu X, Wang Y, Qiang D, Hu L, Han A, Tang X, Liu L, Li W; PURE-China Investigators. Fruit, vegetable, and legume intake and the risk of all-cause, cardiovascular, and cancer mortality: A prospective study. Clin Nutr. 2021 Jun;40(6):4316-4323. doi: 10.1016/j.clnu.2021.01.016. Epub 2021 Jan 27. PMID: 33581953.

254: Qiao T, Chen Y, Duan R, Chen M, Xue H, Tian G, Liang Y, Zhang J, He F, Yang D, Gong Y, Zhou R, Cheng G. Beyond protein intake: does dietary fat intake in the year preceding pregnancy and during pregnancy have an impact on gestational diabetes mellitus? Eur J Nutr. 2021 Sep;60(6):3461-3472. doi: 10.1007/s00394-021-02525-z. Epub 2021 Mar 4. PMID: 33661377; PMCID: PMC8354989.

255: Draper CL, Morrissey E, Younginer N. Health Clinic Readiness to Implement Nutrition Supports in Partnership With SNAP-Ed. J Nutr Educ Behav. 2021 Oct;53(10):843-850. doi: 10.1016/j.jneb.2021.03.008. Epub 2021 May 21. PMID: 34030974.

256: Calvo-Lerma J, Boon M, Hulst J, Colombo C, Asseiceira I, Garriga M, Masip E, Claes I, Bulfamante A, Janssens HM, Roca M, Vicente S, Fornés V, Zazzeron L, van Schijndel B, Woodcock S, Pereira L, de Boeck K, Ribes-Koninckx C. Change in Nutrient and Dietary Intake in European Children with Cystic Fibrosis after a 6-Month Intervention with a Self-Management mHealth Tool. Nutrients. 2021 May 26;13(6):1801. doi: 10.3390/nu13061801. PMID: 34073260; PMCID: PMC8229611.

257: Pfledderer CD, Gren LH, Metos J, Brusseau TA, O'Toole K, Buys SS, Daly MB, Frost CJ. Mothers' Diet and Family Income Predict Daughters' Healthy Eating. Prev Chronic Dis. 2021 Mar 18;18:E24. doi: 10.5888/pcd18.200445. PMID: 33734964; PMCID: PMC7986974.

258: Sacks G, Kwon J, Vandevijvere S, Swinburn B. Benchmarking as a Public Health Strategy for Creating Healthy Food Environments: An Evaluation of the INFORMAS Initiative (2012-2020). Annu Rev Public Health. 2021 Apr 1;42:345-362. doi: 10.1146/annurev-publhealth-100919-114442. Epub 2021 Dec 22. PMID: 33351647.

259: de Winter J, Ezendam NPM, Bours MJL, Winkels RM, Weijenberg MP, Kampman E, Vissers PAJ, Mols F, Beijer S. Is sleep associated with BMI, waist circumference, and diet among long-term colorectal cancer survivors? Results from the population-based PROFILES registry. Support Care Cancer. 2021 Dec;29(12):7225-7235. doi: 10.1007/s00520-021-06393-5. Epub 2021 Jul 6. PMID: 34228174.

260: Della Valle PG, Mosconi G, Nucci D, Vigezzi GP, Gentile L, Gianfredi V, Bonaccio M, Gianfagna F, Signorelli C, Iacoviello L, Odone A. Adherence to the Mediterranean Diet during the COVID-19 national lockdowns: a systematic review of observational studies. Acta Biomed. 2021 Oct 19;92(S6):e2021440. doi: 10.23750/abm.v92iS6.12233. PMID: 34739464.

261: Yin X, Liu H, Webster J, Trieu K, Huffman MD, Miranda JJ, Marklund M, Wu JHY, Cobb LK, Li KC, Pearson SA, Neal B, Tian M. Availability, Formulation, Labeling, and Price of Low-sodium Salt Worldwide: Environmental Scan. JMIR Public Health Surveill. 2021 Jul 14;7(7):e27423. doi: 10.2196/27423. PMID: 33985938; PMCID: PMC8319774.

262: Heidari-Beni M, Riahi R, Massoudi S, Qorbani M, Kelishadi R. Association between dietary diversity score and anthropometric indices among children and adolescents: the weight disorders survey in the CASPIAN-IV study. J Sci Food Agric. 2021 Sep;101(12):5075-5081. doi: 10.1002/jsfa.11152. Epub 2021 Mar 3. PMID: 33570757.

263: Wang Z, Zhao S, Cui X, Song Q, Shi Z, Su J, Zang J. Effects of Dietary

Patterns during Pregnancy on Preterm Birth: A Birth Cohort Study in Shanghai. Nutrients. 2021 Jul 10;13(7):2367. doi: 10.3390/nu13072367. PMID: 34371874; PMCID: PMC8308829.

264: Mahjoub F, Ben Jemaa H, Ben Sabeh F, Ben Amor N, Gamoudi A, Jamoussi H. Impact of nutrients and Mediterranean diet on the occurrence of gestational diabetes. Libyan J Med. 2021 Dec;16(1):1930346. doi: 10.1080/19932820.2021.1930346. PMID: 34024269; PMCID: PMC8158182.

265: Soldavini J, Ammerman AS. Marginal, Low, and Very-Low Food Security among Children Are Associated with Intake of Select Dietary Factors during Summer. J Acad Nutr Diet. 2021 Apr;121(4):728-737. doi: 10.1016/j.jand.2020.11.010. Epub 2020 Dec 5. PMID: 33288496.

266: Wandt VK, Winkelbeiner N, Lossow K, Kopp JF, Schwarz M, Alker W, Nicolai MM, Simon L, Dietzel C, Hertel B, Pohl G, Ebert F, Schomburg L, Bornhorst J, Haase H, Kipp AP, Schwerdtle T. Ageing-associated effects of a long-term dietary modulation of four trace elements in mice. Redox Biol. 2021 Oct;46:102083. doi: 10.1016/j.redox.2021.102083. Epub 2021 Jul 27. PMID: 34371368; PMCID: PMC8358688.

267: O'Donoghue B. Addressing physical health in mental illness: the urgent need to translate evidence-based interventions into routine clinical practice. Ir J Psychol Med. 2021 Mar;38(1):1-5. doi: 10.1017/ipm.2021.4. PMID: 33715645.

268: Altomare A, Del Chierico F, Rocchi G, Emerenziani S, Nuglio C, Putignani L, Angeletti S, Lo Presti A, Ciccozzi M, Russo A, Cocca S, Ribolsi M, Muscaritoli M, Cicala M, Guarino MPL. Association between Dietary Habits and Fecal Microbiota Composition in Irritable Bowel Syndrome Patients: A Pilot Study. Nutrients. 2021 Apr 27;13(5):1479. doi: 10.3390/nu13051479. PMID: 33925672; PMCID: PMC8170891.

269: Zhao Y, Araki T. Evaluation of Disparities in Adults' Macronutrient Intake Status: Results from the China Health and Nutrition 2011 Survey. Nutrients. 2021 Aug 30;13(9):3044. doi: 10.3390/nu13093044. PMID: 34578923; PMCID: PMC8465727.

270: Hart MJ, Torres SJ, McNaughton SA, Milte CM. A Dietary Inflammatory Index and associations with C-reactive protein in a general adult population. Eur J Nutr. 2021 Oct;60(7):4093-4106. doi: 10.1007/s00394-021-02573-5. Epub 2021 May 15. PMID: 33991227.

271: Coelho-Júnior HJ, Trichopoulou A, Panza F. Cross-sectional and longitudinal associations between adherence to Mediterranean diet with physical performance and cognitive function in older adults: A systematic review and meta-analysis. Ageing Res Rev. 2021 Sep;70:101395. doi: 10.1016/j.arr.2021.101395. Epub 2021 Jun 19. PMID: 34153553.

272: Chiu THT, Chang CC, Lin CL, Lin MN. A Vegetarian Diet Is Associated with a Lower Risk of Cataract, Particularly Among Individuals with Overweight: A

Prospective Study. J Acad Nutr Diet. 2021 Apr;121(4):669-677.e1. doi: 10.1016/j.jand.2020.11.003. Epub 2020 Dec 11. PMID: 33309591.

273: Critselis E, Kontogianni MD, Georgousopoulou E, Chrysohoou C, Tousoulis D, Pitsavos C, Panagiotakos DB. Comparison of the Mediterranean diet and the Dietary Approach Stop Hypertension in reducing the risk of 10-year fatal and non-fatal CVD events in healthy adults: the ATTICA Study (2002-2012). Public Health Nutr. 2021 Jun;24(9):2746-2757. doi: 10.1017/S136898002000230X. Epub 2020 Aug 3. PMID: 32744196.

274: Mekal D, Czerw A, Deptala A. Dietary Behaviour and Nutrition in Patients with COPD Treated with Long-Term Oxygen Therapy. Int J Environ Res Public Health. 2021 Dec 4;18(23):12793. doi: 10.3390/ijerph182312793. PMID: 34886519; PMCID: PMC8657430.

275: Bonaccio M, Di Castelnuovo A, Costanzo S, De Curtis A, Persichillo M, Cerletti C, Donati MB, de Gaetano G, Iacoviello L; Moli-sani Study Investigators. Association of a traditional Mediterranean diet and non-Mediterranean dietary scores with all-cause and cause-specific mortality: prospective findings from the Moli-sani Study. Eur J Nutr. 2021 Mar;60(2):729-746. doi: 10.1007/s00394-020-02272-7. Epub 2020 May 21. PMID: 32440732.

276: Mangano KM, Noel SE, Lai CQ, Christensen JJ, Ordovas JM, Dawson-Hughes B, Tucker KL, Parnell LD. Diet-derived fruit and vegetable metabolites show sex-specific inverse relationships to osteoporosis status. Bone. 2021 Mar;144:115780. doi: 10.1016/j.bone.2020.115780. Epub 2020 Dec 2. PMID: 33278656; PMCID: PMC7856195.

277: Bogataj Jontez N, Novak K, Kenig S, Petelin A, Jenko Pražnikar Z, Mohorko N. The Impact of COVID-19-Related Lockdown on Diet and Serum Markers in Healthy Adults. Nutrients. 2021 Mar 26;13(4):1082. doi: 10.3390/nu13041082. PMID: 33810256; PMCID: PMC8066004.

278: Harris JR, Hammerback K, Brown M, Ryan DE, Coe NB, Pike KJ, Santiago PM, Hannon PA. Local Health Jurisdiction Staff Deliver Health Promotion to Small Worksites, Washington. J Public Health Manag Pract. 2021 Mar-Apr 01;27(2):117-124. doi: 10.1097/PHH.0000000000001105. PMID: 31738191; PMCID: PMC7220816.

279: Sloan RP, Wall M, Yeung LK, Feng T, Feng X, Provenzano F, Schroeter H, Lauriola V, Brickman AM, Small SA. Insights into the role of diet and dietary flavanols in cognitive aging: results of a randomized controlled trial. Sci Rep. 2021 Feb 15;11(1):3837. doi: 10.1038/s41598-021-83370-2. PMID: 33589674; PMCID: PMC7884710.

280: Kammholz G, Craven D, Boodoosingh R, Akeli Amaama S, Abraham J, Burkhart S. Exploring Food Literacy Domains in an Adult Samoan Population. Int J Environ Res Public Health. 2021 Mar 30;18(7):3587. doi: 10.3390/ijerph18073587. PMID:

33808339; PMCID: PMC8036961.

281: Janowska-Miasik E, Waśkiewicz A, Witkowska AM, Drygas W, Markhus MW, Zujko ME, Kjellevold M. Diet quality in the population of Norway and Poland: differences in the availability and consumption of food considering national nutrition guidelines and food market. BMC Public Health. 2021 Feb 9;21(1):319. doi: 10.1186/s12889-021-10361-3. PMID: 33563240; PMCID: PMC7871600.

282: Ivey KL, Nguyen XT, Quaden RM, Ho YL, Cho K, Gaziano JM, Djoussé L. Association of Nut Consumption with Risk of Stroke and Cardiovascular Disease: The Million Veteran Program. Nutrients. 2021 Aug 30;13(9):3031. doi: 10.3390/nu13093031. PMID: 34578913; PMCID: PMC8472092.

283: Al-Sunaid FF, Al-Homidi MM, Al-Qahtani RM, Al-Ashwal RA, Mudhish GA, Hanbazaza MA, Al-Zaben AS. The influence of a gluten-free diet on health-related quality of life in individuals with celiac disease. BMC Gastroenterol. 2021 Aug 25;21(1):330. doi: 10.1186/s12876-021-01908-0. PMID: 34433427; PMCID: PMC8390240.

284: Do WL, Whitsel EA, Costeira R, Masachs OM, Le Roy CI, Bell JT, Staimez LR, Stein AD, Smith AK, Horvath S, Assimes TL, Liu S, Manson JE, Shadyab AH, Li Y, Hou L, Bhatti P, Jordahl K, Narayan KMV, Conneely KN. Epigenome-wide association study of diet quality in the Women's Health Initiative and TwinsUK cohort. Int J Epidemiol. 2021 May 17;50(2):675-684. doi: 10.1093/ije/dyaa215. PMID: 33354722; PMCID: PMC8128469.

285: Fang Y, Zhu J, Fan J, Sun L, Cai S, Fan C, Zhong Y, Li Y. Dietary Inflammatory Index in relation to bone mineral density, osteoporosis risk and fracture risk: a systematic review and meta-analysis. Osteoporos Int. 2021 Apr;32(4):633-643. doi: 10.1007/s00198-020-05578-8. Epub 2020 Aug 1. PMID: 32740669.

286: Zhang J, Hayden K, Jackson R, Schutte R. Association of red and processed meat consumption with cardiovascular morbidity and mortality in participants with and without obesity: A prospective cohort study. Clin Nutr. 2021 May;40(5):3643-3649. doi: 10.1016/j.clnu.2020.12.030. Epub 2020 Dec 29. PMID: 33413912.

287: Shams-White MM, Korycinski RW, Dodd KW, Barrett B, Jacobs S, Subar AF, Park Y, Bowles HR. Examining the association between meal context and diet quality: an observational study of meal context in older adults. Int J Behav Nutr Phys Act. 2021 May 20;18(1):67. doi: 10.1186/s12966-021-01122-x. PMID: 34016140; PMCID: PMC8136192.

288: Hartmann AM, Dell'Oro M, Kessler CS, Schumann D, Steckhan N, Jeitler M, Fischer JM, Spoo M, Kriegel MA, Schneider JG, Häupl T, Kandil FI, Michalsen A, Koppold-Liebscher DA. Efficacy of therapeutic fasting and plant-based diet in patients with rheumatoid arthritis (NutriFast): study protocol for a randomised controlled clinical trial. BMJ Open. 2021 Aug 11;11(8):e047758. doi:

10.1136/bmjopen-2020-047758. PMID: 34380725; PMCID: PMC8359474.

289: Valerino-Perea S, Armstrong MEG, Papadaki A. Development of an index to assess adherence to the traditional Mexican diet using a modified Delphi method. Public Health Nutr. 2021 Oct;24(14):4387-4396. doi: 10.1017/S1368980020004565. Epub 2020 Nov 13. PMID: 33183382.

290: Grace-Farfaglia P. Self-Reported Diet and Health Outcomes of Participants of the CCSVI-Tracking Survey Study. Nutrients. 2021 May 31;13(6):1891. doi: 10.3390/nu13061891. PMID: 34072860; PMCID: PMC8230225.

291: van Dongen KCW, Linkens AMA, Wetzels SMW, Wouters K, Vanmierlo T, van de Waarenburg MPH, Scheijen JLJM, de Vos WM, Belzer C, Schalkwijk CG. Dietary advanced glycation endproducts (AGEs) increase their concentration in plasma and tissues, result in inflammation and modulate gut microbial composition in mice; evidence for reversibility. Food Res Int. 2021 Sep;147:110547. doi: 10.1016/j.foodres.2021.110547. Epub 2021 Jun 18. PMID: 34399524.

292: Oladeji O, Zhang C, Moradi T, Tarapore D, Stokes AC, Marivate V, Sengeh MD, Nsoesie EO. Monitoring Information-Seeking Patterns and Obesity Prevalence in Africa With Internet Search Data: Observational Study. JMIR Public Health Surveill. 2021 Apr 29;7(4):e24348. doi: 10.2196/24348. PMID: 33913815; PMCID: PMC8120431.

293: Baldeón ME, Felix C, Fornasini M, Zertuche F, Largo C, Paucar MJ, Ponce L, Rangarajan S, Yusuf S, López-Jaramillo P. Prevalence of metabolic syndrome and diabetes mellitus type-2 and their association with intake of dairy and legume in Andean communities of Ecuador. PLoS One. 2021 Jul 23;16(7):e0254812. doi: 10.1371/journal.pone.0254812. PMID: 34297755; PMCID: PMC8301611.

294: Wang Y, Dai Y, Tian T, Zhang J, Xie W, Pan D, Xu D, Lu Y, Wang S, Xia H, Sun G. The Effects of Dietary Pattern on Metabolic Syndrome in Jiangsu Province of China: Based on a Nutrition and Diet Investigation Project in Jiangsu Province. Nutrients. 2021 Dec 13;13(12):4451. doi: 10.3390/nu13124451. PMID: 34960003; PMCID: PMC8708757.

295: Aglago EK, Mayén AL, Knaze V, Freisling H, Fedirko V, Hughes DJ, Jiao L, Eriksen AK, Tjønneland A, Boutron-Ruault MC, Rothwell JA, Severi G, Kaaks R, Katzke V, Schulze MB, Birukov A, Palli D, Sieri S, Santucci de Magistris M, Tumino R, Ricceri F, Bueno-de-Mesquita B, Derksen JWG, Skeie G, Gram IT, Sandanger T, Quirós JR, Luján-Barroso L, Sánchez MJ, Amiano P, Chirlaque MD, Gurrea AB, Johansson I, Manjer J, Perez-Cornago A, Weiderpass E, Gunter MJ, Heath AK, Schalkwijk CG, Jenab M. Dietary Advanced Glycation End-Products and Colorectal Cancer Risk in the European Prospective Investigation into Cancer and Nutrition (EPIC) Study. Nutrients. 2021 Sep 8;13(9):3132. doi: 10.3390/nu13093132. PMID: 34579010; PMCID: PMC8470201.

296: Duan Y, Shang B, Liang W, Du G, Yang M, Rhodes RE. Effects of eHealth-Based Multiple Health Behavior Change Interventions on Physical Activity, Healthy

Diet, and Weight in People With Noncommunicable Diseases: Systematic Review and Meta-analysis. J Med Internet Res. 2021 Feb 22;23(2):e23786. doi: 10.2196/23786. PMID: 33616534; PMCID: PMC8074786.

297: Nardocci M, Polsky JY, Moubarac JC. Consumption of ultra-processed foods is associated with obesity, diabetes and hypertension in Canadian adults. Can J Public Health. 2021 Jun;112(3):421-429. doi: 10.17269/s41997-020-00429-9. Epub 2020 Nov 10. PMID: 33174128; PMCID: PMC8076355.

298: Lee YQ, Lumbers ER, Schumacher TL, Collins CE, Rae KM, Pringle KG, Gomeroi Gaaynggal Advisory Committee. Maternal Diet Influences Fetal Growth but Not Fetal Kidney Volume in an Australian Indigenous Pregnancy Cohort. Nutrients. 2021 Feb 9;13(2):569. doi: 10.3390/nu13020569. PMID: 33572217; PMCID: PMC7914647.

299: Neuhouser ML, Pettinger M, Lampe JW, Tinker LF, George SM, Reedy J, Song X, Thyagarajan B, Beresford SA, Prentice RL. Novel Application of Nutritional Biomarkers From a Controlled Feeding Study and an Observational Study to Characterization of Dietary Patterns in Postmenopausal Women. Am J Epidemiol. 2021 Nov 2;190(11):2461-2473. doi: 10.1093/aje/kwab171. PMID: 34142699; PMCID: PMC8799924.

300: Qin Y, Chen Y, Zhang Z, Huang S, Jiao C, Zhang Z, Mao L. Associations of dietary inflammatory potential with postpartum weight change and retention: Results from a cohort study. Obesity (Silver Spring). 2021 Oct;29(10):1689-1699. doi: 10.1002/oby.23238. Epub 2021 Sep 7. PMID: 34490743.

301: Tan KW, Quaye SED, Koo JR, Lim JT, Cook AR, Dickens BL. Assessing the Impact of Salt Reduction Initiatives on the Chronic Disease Burden of Singapore. Nutrients. 2021 Apr 1;13(4):1171. doi: 10.3390/nu13041171. PMID: 33916184; PMCID: PMC8065991.

302: Fan Y, Zhang Y, Li J, Liu Y, Zhou L, Yu Y. Association between Healthy Eating Index-2015 and physical frailty among the United States elderly adults: the National Health and Nutrition Examination Survey (NHANES) 2011-2014. Aging Clin Exp Res. 2021 Dec;33(12):3245-3255. doi: 10.1007/s40520-021-01874-3. Epub 2021 May 12. PMID: 33978925.

303: Wright CN, Jaceldo-Siegl K, Mashchak A, Singh PN, Fraser GE. Validation of estimated glycaemic index and glycaemic load, stratified by race, in the Adventist Health Study-2 (AHS-2). Public Health Nutr. 2021 Oct;24(14):4530-4536. doi: 10.1017/S1368980020003778. Epub 2021 Jan 8. PMID: 33413707.

304: Fiorindi C, Dinu M, Gavazzi E, Scaringi S, Ficari F, Nannoni A, Sofi F, Giudici F. Adherence to mediterranean diet in patients with inflammatory bowel disease. Clin Nutr ESPEN. 2021 Dec;46:416-423. doi: 10.1016/j.clnesp.2021.09.726. Epub 2021 Sep 29. PMID: 34857229.

305: Matsumoto Y, Shivappa N, Sugioka Y, Tada M, Okano T, Mamoto K, Inui K, Habu

D, Hebert JR, Koike T. Change in dietary inflammatory index score is associated with control of long-term rheumatoid arthritis disease activity in a Japanese cohort: the TOMORROW study. Arthritis Res Ther. 2021 Apr 8;23(1):105. doi: 10.1186/s13075-021-02478-y. PMID: 33832530; PMCID: PMC8028141.

306: Millar SR, Navarro P, Harrington JM, Shivappa N, Hébert JR, Perry IJ, Phillips CM. Comparing dietary score associations with lipoprotein particle subclass profiles: A cross-sectional analysis of a middle-to older-aged population. Clin Nutr. 2021 Jul;40(7):4720-4729. doi: 10.1016/j.clnu.2021.06.005. Epub 2021 Jun 16. PMID: 34237699.

307: Firat S, Nogay NH. Association of blood pressure with dietary intake, physical activity, and anthropometric measurements in Turkish adolescents. Niger J Clin Pract. 2021 Nov;24(11):1616-1623. doi: 10.4103/njcp.njcp_685_20. PMID: 34782499.

308: Abbate M, Mascaró CM, Montemayor S, Barbería-Latasa M, Casares M, Gómez C, Ugarriza L, Tejada S, Abete I, Zulet MÁ, Sureda A, Martínez JA, Tur JA. Animal Fat Intake Is Associated with Albuminuria in Patients with Non-Alcoholic Fatty Liver Disease and Metabolic Syndrome. Nutrients. 2021 May 4;13(5):1548. doi: 10.3390/nu13051548. PMID: 34064372; PMCID: PMC8147815.

309: Becerra-Tomás N, Paz-Graniel I, Hernández-Alonso P, Jenkins DJA, Kendall CWC, Sievenpiper JL, Salas-Salvadó J. Nut consumption and type 2 diabetes risk: a systematic review and meta-analysis of observational studies. Am J Clin Nutr. 2021 Apr 6;113(4):960-971. doi: 10.1093/ajcn/nqaa358. PMID: 33471083.

310: Amiri M, Karabegović I, van Westing AC, Verkaar AJCF, Beigrezaei S, Lara M, Bramer WM, Voortman T. Whole-diet interventions and cardiovascular risk factors in postmenopausal women: A systematic review of controlled clinical trials. Maturitas. 2022 Jan;155:40-53. doi: 10.1016/j.maturitas.2021.10.001. Epub 2021 Oct 9. PMID: 34876248.

311: Pedroni C, Castetbon K, Desbouys L, Rouche M, Vandevijvere S. The Cost of Diets According to Nutritional Quality and Sociodemographic Characteristics: A Population-Based Assessment in Belgium. J Acad Nutr Diet. 2021 Nov;121(11):2187-2200.e4. doi: 10.1016/j.jand.2021.05.024. Epub 2021 Jun 24. PMID: 34175255.

312: Prete M, Luzzetti A, Augustin LSA, Porciello G, Montagnese C, Calabrese I, Ballarin G, Coluccia S, Patel L, Vitale S, Palumbo E, Celentano E, La Vecchia C, Crispo A. Changes in Lifestyle and Dietary Habits during COVID-19 Lockdown in Italy: Results of an Online Survey. Nutrients. 2021 Jun 3;13(6):1923. doi: 10.3390/nu13061923. PMID: 34205057; PMCID: PMC8230113.

313: Baart AM, Brouwer-Brolsma EM, Perenboom CWM, de Vries JHM, Feskens EJM. Dietary Intake in the Lifelines Cohort Study: Baseline Results from the Flower Food Frequency Questionnaire among 59,982 Participants. Nutrients. 2021 Dec 23;14(1):48. doi: 10.3390/nu14010048. PMID: 35010922; PMCID: PMC8746534.

314: Marrero A, Tamez M, Rodríguez-Orengo JF, Mattei J. The association between purchasing locally produced food and diet quality among adults in Puerto Rico. Public Health Nutr. 2021 Sep;24(13):4177-4186. doi: 10.1017/S1368980020003134. Epub 2020 Sep 9. PMID: 32900415; PMCID: PMC7940455.

315: Weimann A, Shung-King M, McCreedy N, Tatah L, Mapa-Tassou C, Muzenda T, Govia I, Were V, Oni T. Intersectoral Action for Addressing NCDs through the Food Environment: An Analysis of NCD Framing in Global Policies and Its Relevance for the African Context. Int J Environ Res Public Health. 2021 Oct 26;18(21):11246. doi: 10.3390/ijerph182111246. PMID: 34769763; PMCID: PMC8582825.

316: Brown MC, Marciniak CM, Garrett AM, Gaebler-Spira DJ. Diet quality in adults with cerebral palsy: a modifiable risk factor for cardiovascular disease prevention. Dev Med Child Neurol. 2021 Oct;63(10):1221-1228. doi: 10.1111/dmcn.14913. Epub 2021 May 6. PMID: 33959958.

317: Malavolti M, Naska A, Fairweather-Tait SJ, Malagoli C, Vescovi L, Marchesi C, Vinceti M, Filippini T. Sodium and Potassium Content of Foods Consumed in an Italian Population and the Impact of Adherence to a Mediterranean Diet on Their Intake. Nutrients. 2021 Aug 1;13(8):2681. doi: 10.3390/nu13082681. PMID: 34444841; PMCID: PMC8401684.

318: Freak-Poli R, Brand M, Boelsen-Robinson T, Huse O, de Courten M, Peeters A. Development and piloting of a Checklist for healthy eating And Physical Activity in the Workplace (CEPAW). Health Promot Int. 2021 Mar 12;36(1):8-19. doi: 10.1093/heapro/daaa026. PMID: 32268355.

319: Dumas BL, Harris DM, McMahon JM, Daymude TJ, Warnock AL, Moore LV, Onufrak SJ. Prevalence of Municipal-Level Policies Dedicated to Transportation That Consider Food Access. Prev Chronic Dis. 2021 Nov 18;18:E97. doi: 10.5888/pcd18.210193. PMID: 34793691; PMCID: PMC8673943.

320: Brauer P, Royall D, Rodrigues A. Use of the Healthy Eating Index in Intervention Studies for Cardiometabolic Risk Conditions: A Systematic Review. Adv Nutr. 2021 Jul 30;12(4):1317-1331. doi: 10.1093/advances/nmaa167. PMID: 33460430; PMCID: PMC8321868.

321: Juton C, Lerin C, Homs C, Casas Esteve R, Berruezo P, Cárdenas-Fuentes G, Fíto M, Grau M, Estrada L, Gómez SF, Schröder H. Prospective Associations between Maternal and Child Diet Quality and Sedentary Behaviors. Nutrients. 2021 May 18;13(5):1713. doi: 10.3390/nu13051713. PMID: 34070137; PMCID: PMC8158334.

322: Kuhail M, Shab-Bidar S, Yaseri M, Djafarian K. Major Dietary Patterns Relationship with Severity of Coronary Artery Disease in Gaza-Strip, Palestine: A Cross-Sectional Study. Ethiop J Health Sci. 2021 May;31(3):599-610. doi: 10.4314/ejhs.v31i3.17. PMID: 34483617; PMCID: PMC8365497.

323: Choy MJY, Brownlee I, Murphy AM. Data-Driven Dietary Patterns, Nutrient Intake and Body Weight Status in a Cross-Section of Singaporean Children Aged 6-12 Years. Nutrients. 2021 Apr 17;13(4):1335. doi: 10.3390/nu13041335. PMID: 33920618; PMCID: PMC8074157.

324: Govindaraju T, McCaffrey TA, McNeil JJ, Reid CM, Smith BJ, Campbell DJ, Owen AJ. Mis-reporting of energy intake among older Australian adults: Prevalence, characteristics, and associations with quality of life. Nutrition. 2021 Oct;90:111259. doi: 10.1016/j.nut.2021.111259. Epub 2021 Apr 6. PMID: 33975061.

325: Riseberg E, Tamez M, Tucker KL, Rodriguez Orengo JF, Mattei J. Associations between diet quality scores and central obesity among adults in Puerto Rico. J Hum Nutr Diet. 2021 Dec;34(6):1014-1021. doi: 10.1111/jhn.12873. Epub 2021 May 14. PMID: 33988871; PMCID: PMC8590710.

326: Zhang X, Zhang J, Du W, Su C, Ouyang Y, Huang F, Jia X, Li L, Bai J, Zhang B, Wang Z, Du S, Wang H. Multi-Trajectories of Macronutrient Intake and Their Associations with Obesity among Chinese Adults from 1991 to 2018: A Prospective Study. Nutrients. 2021 Dec 21;14(1):13. doi: 10.3390/nu14010013. PMID: 35010888; PMCID: PMC8746800.

327: Lin Y, Zheng Y, Wang HL, Wu J. Global Patterns and Trends in Gastric Cancer Incidence Rates (1988-2012) and Predictions to 2030. Gastroenterology. 2021 Jul;161(1):116-127.e8. doi: 10.1053/j.gastro.2021.03.023. Epub 2021 Mar 18. PMID: 33744306.

328: Monge A, Silva Canella D, López-Olmedo N, Lajous M, Cortés-Valencia A, Stern D. Ultraprocessed beverages and processed meats increase the incidence of hypertension in Mexican women. Br J Nutr. 2021 Aug 28;126(4):600-611. doi: 10.1017/S0007114520004432. Epub 2020 Nov 5. PMID: 33148348.

329: Jung YJ, Lee SH, Chang JH, Lee HS, Kang EH, Lee SW. The Impact of Changes in the Intake of Fiber and Antioxidants on the Development of Chronic Obstructive Pulmonary Disease. Nutrients. 2021 Feb 10;13(2):580. doi: 10.3390/nu13020580. PMID: 33578669; PMCID: PMC7916350.

330: Assunção R, Boué G, Alvito P, Brazão R, Carmona P, Carvalho C, Correia D, Fernandes P, Lopes C, Martins C, Membré JM, Monteiro S, Nabais P, Thomsen ST, Torres D, Pires SM, Jakobsen LS. Risk-Benefit Assessment of Cereal-Based Foods Consumed by Portuguese Children Aged 6 to 36 Months-A Case Study under the RiskBenefit4EU Project. Nutrients. 2021 Sep 8;13(9):3127. doi: 10.3390/nu13093127. PMID: 34579004; PMCID: PMC8467172.

331: Boqué N, Tarro L, Rosi A, Torrell H, Saldaña G, Luengo E, Rachman Z, Pires A, Tavares NT, Pires AS, Botelho MF, Mena P, Scazzina F, Del Rio D, Caimari A. Study Protocol of a Multicenter Randomized Controlled Trial to Tackle Obesity through a Mediterranean Diet vs. a Traditional Low-Fat Diet in Adolescents: The MED4Youth Study. Int J Environ Res Public Health. 2021 May 1;18(9):4841. doi:

10.3390/ijerph18094841. PMID: 34062731; PMCID: PMC8125034.

332: Burch E, Williams LT, Thalib L, Ball L. What happens to diet quality in people newly diagnosed with type 2 diabetes? The 3D case-series study. J Hum Nutr Diet. 2022 Feb;35(1):191-201. doi: 10.1111/jhn.12953. Epub 2021 Oct 25. PMID: 34694048.

333: Ribó-Coll M, Lassale C, Sacanella E, Ros E, Toledo E, Sorlí JV, Babio N, Lapetra J, Gómez-Gracia E, Alonso-Gómez ÁM, Fiol M, Serra-Majem L, Pinto X, Castañer O, Díez-Espino J, González JI, Becerra-Tomás N, Cofán M, Díaz-López A, Estruch R, Hernáez Á. Mediterranean diet and antihypertensive drug use: a randomized controlled trial. J Hypertens. 2021 Jun 1;39(6):1230-1237. doi: 10.1097/HJH.0000000000002765. PMID: 33496530.

334: Naspolini NF, Machado PP, Moreira JC, Asmus CIRF, Meyer A. Maternal consumption of ultra-processed foods and newborn exposure to perfluoroalkyl substances (PFAS). Cad Saude Publica. 2021 Dec 1;37(11):e00152021. doi: 10.1590/0102-311X00152021. PMID: 34877989.

335: Amazouz H, Roda C, Beydon N, Lezmi G, Bourgoin-Heck M, Just J, Momas I, Rancière F. Mediterranean diet and lung function, sensitization, and asthma at school age: The PARIS cohort. Pediatr Allergy Immunol. 2021 Oct;32(7):1437-1444. doi: 10.1111/pai.13527. Epub 2021 May 21. PMID: 33914969.

336: Kirkham AA, King K, Joy AA, Pelletier AB, Mackey JR, Young K, Zhu X, Meza-Junco J, Basi SK, Hiller JP, Brkin T, Michalowski B, Pituskin E, Paterson DI, Courneya KS, Thompson RB, Prado CM. Rationale and design of the Diet Restriction and Exercise-induced Adaptations in Metastatic breast cancer (DREAM) study: a 2-arm, parallel-group, phase II, randomized control trial of a short-term, calorie-restricted, and ketogenic diet plus exercise during intravenous chemotherapy versus usual care. BMC Cancer. 2021 Oct 10;21(1):1093. doi: 10.1186/s12885-021-08808-2. PMID: 34629067; PMCID: PMC8504029.

337: Rennekamp R, Brandl B, Giesbertz P, Skurk T, Hauner H. Metabolic and satiating effects and consumer acceptance of a fibre-enriched Leberkas meal: a randomized cross-over trial. Eur J Nutr. 2021 Sep;60(6):3203-3210. doi: 10.1007/s00394-020-02472-1. Epub 2021 Feb 8. PMID: 33555374.

338: Dorling JL, Ravussin E, Redman LM, Bhapkar M, Huffman KM, Racette SB, Das SK, Apolzan JW, Kraus WE, Höchsmann C, Martin CK; CALERIE Phase 2 Study Group. Effect of 2 years of calorie restriction on liver biomarkers: results from the CALERIE phase 2 randomized controlled trial. Eur J Nutr. 2021 Apr;60(3):1633-1643. doi: 10.1007/s00394-020-02361-7. Epub 2020 Aug 14. PMID: 32803412; PMCID: PMC7882001.

339: Schorgg P, Bärnighausen T, Rohrmann S, Cassidy A, Karavasiloglou N, Kühn T. Vitamin B6 Status among Vegetarians: Findings from a Population-Based Survey. Nutrients. 2021 May 12;13(5):1627. doi: 10.3390/nu13051627. PMID: 34066199; PMCID: PMC8150266.

340: Bhattacharya R, Zekavat SM, Uddin MM, Pirruccello J, Niroula A, Gibson C, Griffin GK, Libby P, Ebert BL, Bick A, Natarajan P. Association of Diet Quality With Prevalence of Clonal Hematopoiesis and Adverse Cardiovascular Events. JAMA Cardiol. 2021 Sep 1;6(9):1069-1077. doi: 10.1001/jamacardio.2021.1678. PMID: 34106216; PMCID: PMC8190703.

341: Ribeiro FM, Lopes G, da Cunha Nascimento D, Pires L, Mulder AP, Franco OL, Petriz B. An overview of the level of dietary support in the gut microbiota at different stages of life: A systematic review. Clin Nutr ESPEN. 2021 Apr;42:41-52. doi: 10.1016/j.clnesp.2021.01.024. Epub 2021 Feb 9. PMID: 33745615.

342: Simpson-Yap S, Nag N, Jakaria M, Jelinek GA, Neate S. Sociodemographic and clinical characteristics of diet adherence and relationship with diet quality in an international cohort of people with multiple sclerosis. Mult Scler Relat Disord. 2021 Nov;56:103307. doi: 10.1016/j.msard.2021.103307. Epub 2021 Oct 3. PMID: 34627006.

343: Bzikowska-Jura A, Sobieraj P, Raciborski F. Low Comparability of Nutrition-Related Mobile Apps against the Polish Reference Method-A Validity Study. Nutrients. 2021 Aug 20;13(8):2868. doi: 10.3390/nu13082868. PMID: 34445026; PMCID: PMC8398064.

344: Arnolda G, Hiscock H, Moore D, Farrow G, Hibbert PD, Wiles LK, Ting HP, Molloy CJ, Warwick M, Braithwaite J. Assessing the appropriateness of the management of gastro-oesophageal reflux in Australian children: a population-based sample survey. Sci Rep. 2021 Apr 8;11(1):7744. doi: 10.1038/s41598-021-87369-7. PMID: 33833360; PMCID: PMC8032666.

345: Shemirani F, Fotouhi A, Djafarian K, Azadbakht L, Rezaei N, Mahmoudi M. Effects of modified-Paleo and moderate-carbohydrate diets on body composition, serum levels of hepatokines and adipocytokines, and flow cytometric analysis of endothelial microparticles in adults with metabolic syndrome: a study protocol for a randomized clinical trial. Trials. 2021 Sep 30;22(1):673. doi: 10.1186/s13063-021-05612-y. PMID: 34593030; PMCID: PMC8483422.

346: Carioca AAF, Gorgulho B, de Mello Fontanelli M, Fisberg RM, Marchioni DM. Cardiometabolic risk profile and diet quality among internal migrants in Brazil: a population-based study. Eur J Nutr. 2021 Mar;60(2):759-768. doi: 10.1007/s00394-020-02281-6. Epub 2020 May 21. PMID: 32440729.

347: Zuraikat FM, St-Onge MP, Makarem N, Boege HL, Xi H, Aggarwal B. Evening Chronotype Is Associated with Poorer Habitual Diet in US Women, with Dietary Energy Density Mediating a Relation of Chronotype with Cardiovascular Health. J Nutr. 2021 May 11;151(5):1150-1158. doi: 10.1093/jn/nxaa442. PMID: 33758908; PMCID: PMC8112764.

348: Alsulami S, Bodhini D, Sudha V, Shanthi Rani CS, Pradeepa R, Anjana RM,

Radha V, Lovegrove JA, Gayathri R, Mohan V, Vimaleswaran KS. Lower Dietary Intake of Plant Protein Is Associated with Genetic Risk of Diabetes-Related Traits in Urban Asian Indian Adults. Nutrients. 2021 Aug 31;13(9):3064. doi: 10.3390/nu13093064. PMID: 34578944; PMCID: PMC8466015.

349: Kamran R BSc (Hons), MD (c), Coletta G BSc (Hons), MSc (c), Pritchard JM PhD, BSc. Can Undergraduate Students Help Change Older Adults' Confidence for Making Nutrition-Related Decisions in a 45-Minute Nutrition Workshop? Can J Diet Pract Res. 2021 Sep 1;82(3):136-139. doi: 10.3148/cjdpr-2021-010. Epub 2021 Jul 21. PMID: 34286607.

350: Svop Jensen V, Fledelius C, Max Wulff E, Lykkesfeldt J, Hvid H. Temporal Development of Dyslipidemia and Nonalcoholic Fatty Liver Disease (NAFLD) in Syrian Hamsters Fed a High-Fat, High-Fructose, High-Cholesterol Diet. Nutrients. 2021 Feb 12;13(2):604. doi: 10.3390/nu13020604. PMID: 33673227; PMCID: PMC7917647.

351: Caspi CE, Davey C, Barsness CB, Wolfson J, Peterson H, Pratt RJ. Applying the Healthy Eating Index-2015 in a Sample of Choice-Based Minnesota Food Pantries to Test Associations Between Food Pantry Inventory, Client Food Selection, and Client Diet. J Acad Nutr Diet. 2021 Nov;121(11):2242-2250. doi: 10.1016/j.jand.2021.05.007. Epub 2021 Jun 5. PMID: 34103273; PMCID: PMC8530893.

352: Lassale C, Hernáez Á, Toledo E, Castañer O, Sorlí JV, Salas-Salvadó J, Estruch R, Ros E, Alonso-Gómez ÁM, Lapetra J, Cueto R, Fiol M, Serra-Majem L, Pinto X, Gea A, Corella D, Babio N, Fitó M, Schröder H. Energy Balance and Risk of Mortality in Spanish Older Adults. Nutrients. 2021 May 4;13(5):1545. doi: 10.3390/nu13051545. PMID: 34064328; PMCID: PMC8147789.

353: Hecht EM, Williams AP, Abrams GA, Passman RS. Cardiovascular Risk Factors in Young Adolescents: Results from the National Health and Nutrition Examination Survey 1988-2016. South Med J. 2021 May;114(5):261-265. doi: 10.14423/SMJ.0000000000001244. PMID: 33942107.

354: Franco S, Godinho C, Silva CS, Avelar-Rosa B, Santos R, Mendes R, Silva MN. Assessment of Good Practices in Community-Based Interventions for Physical Activity Promotion: Development of a User-Friendly Tool. Int J Environ Res Public Health. 2021 Apr 29;18(9):4734. doi: 10.3390/ijerph18094734. PMID: 33946746; PMCID: PMC8124131.

355: Tessari S, Casazza M, De Boni G, Bertoncello C, Fonzo M, Di Pieri M, Russo F. Promoting health and preventing non-communicable diseases: evaluation of the adherence of the Italian population to the Mediterranean Diet by using the PREDIMED questionnaire. Ann Ig. 2021 Jul-Aug;33(4):337-346. doi: 10.7416/ai.2020.2393. Epub 2020 Dec 4. PMID: 33270077.

356: Goodyear VA, Boardley I, Chiou SY, Fenton SAM, Makopoulou K, Stathi A, Wallis GA, Veldhuijzen van Zanten JJCS, Thompson JL. Social media use informing behaviours related to physical activity, diet and quality of life during

COVID-19: a mixed methods study. BMC Public Health. 2021 Jul 6;21(1):1333. doi: 10.1186/s12889-021-11398-0. PMID: 34229651; PMCID: PMC8259772.

357: Würtz AML, Jakobsen MU, Bertoia ML, Hou T, Schmidt EB, Willett WC, Overvad K, Sun Q, Manson JE, Hu FB, Rimm EB. Replacing the consumption of red meat with other major dietary protein sources and risk of type 2 diabetes mellitus: a prospective cohort study. Am J Clin Nutr. 2021 Mar 11;113(3):612-621. doi: 10.1093/ajcn/nqaa284. PMID: 33094800; PMCID: PMC7948828.

358: He Y, Fang Y, Bromage S, Fung TT, Bhupathiraju SN, Batis C, Deitchler M, Fawzi W, Stampfer MJ, Hu FB, Willett WC, Li Y. Application of the Global Diet Quality Score in Chinese Adults to Evaluate the Double Burden of Nutrient Inadequacy and Metabolic Syndrome. J Nutr. 2021 Oct 23;151(12 Suppl 2):93S-100S. doi: 10.1093/jn/nxab162. PMID: 34689199; PMCID: PMC8542094.

359: Raiten DJ, Combs GF, Steiber AL, Bremer AA. Perspective: Nutritional Status as a Biological Variable (NABV): Integrating Nutrition Science into Basic and Clinical Research and Care. Adv Nutr. 2021 Oct 1;12(5):1599-1609. doi: 10.1093/advances/nmab046. PMID: 34009250; PMCID: PMC8483963.

360: Naude CE, Brand A, Schoonees A, Nguyen KA, Chaplin M, Volmink J. Low-carbohydrate versus balanced-carbohydrate diets for reducing weight and cardiovascular risk. Cochrane Database Syst Rev. 2022 Jan 28;1(1):CD013334. doi: 10.1002/14651858.CD013334.pub2. PMID: 35088407; PMCID: PMC8795871.

361: Lauria F, Dello Russo M, Formisano A, De Henauw S, Hebestreit A, Hunsberger M, Krogh V, Intemann T, Lissner L, Molnar D, Moreno LA, Reisch LA, Tornaritis M, Veidebaum T, Williams G, Siani A, Russo P; I.Family consortium. Ultra-processed foods consumption and diet quality of European children, adolescents and adults: Results from the I.Family study. Nutr Metab Cardiovasc Dis. 2021 Oct 28;31(11):3031-3043. doi: 10.1016/j.numecd.2021.07.019. Epub 2021 Jul 27. PMID: 34518085.

362: Lutsiv T, Weir TL, McGinley JN, Neil ES, Wei Y, Thompson HJ. Compositional Changes of the High-Fat Diet-Induced Gut Microbiota upon Consumption of Common Pulses. Nutrients. 2021 Nov 9;13(11):3992. doi: 10.3390/nu13113992. PMID: 34836246; PMCID: PMC8625176.

363: Buzzetti E, Linden A, Best LM, Madden AM, Roberts D, Chase TJG, Freeman SC, Cooper NJ, Sutton AJ, Fritche D, Milne EJ, Wright K, Pavlov CS, Davidson BR, Tsochatzis E, Gurusamy KS. Lifestyle modifications for nonalcohol-related fatty liver disease: a network meta-analysis. Cochrane Database Syst Rev. 2021 Jun 11;6(6):CD013156. doi: 10.1002/14651858.CD013156.pub2. PMID: 34114650; PMCID: PMC8193812.

364: Zhuang P, Jiao J, Wu F, Mao L, Zhang Y. Associations of meat consumption and changes with all-cause mortality in hypertensive patients during 11.4-year follow-up: Findings from a population-based nationwide cohort. Clin Nutr. 2021 Mar;40(3):1077-1084. doi: 10.1016/j.clnu.2020.06.040. Epub 2020 Jul 14. PMID:

32741682.

365: Moursi M, Bromage S, Fung TT, Isanaka S, Matsuzaki M, Batis C, Castellanos-Gutiérrez A, Angulo E, Birk N, Bhupathiraju SN, He Y, Li Y, Fawzi W, Danielyan A, Thapa S, Ndiyoi L, Vossenaar M, Bellows A, Arsenault JE, Willett WC, Deitchler M. There's an App for That: Development of an Application to Operationalize the Global Diet Quality Score. J Nutr. 2021 Oct 23;151(12 Suppl 2):176S-184S. doi: 10.1093/jn/nxab196. PMID: 34689193; PMCID: PMC8542098.

366: Yu D, Nguyen SM, Yang Y, Xu W, Cai H, Wu J, Cai Q, Long J, Zheng W, Shu XO. Long-term diet quality is associated with gut microbiome diversity and composition among urban Chinese adults. Am J Clin Nutr. 2021 Mar 11;113(3):684-694. doi: 10.1093/ajcn/nqaa350. PMID: 33471054; PMCID: PMC7948864.

367: Tosetti C, Savarino E, Benedetto E, De Bastiani R; Study Group for the Evaluation of GERD Triggering Foods. Elimination of Dietary Triggers Is Successful in Treating Symptoms of Gastroesophageal Reflux Disease. Dig Dis Sci. 2021 May;66(5):1565-1571. doi: 10.1007/s10620-020-06414-z. Epub 2020 Jun 24. PMID: 32578044.

368: Mompeo O, Berry SE, Spector TD, Menni C, Mangino M, Gibson R. Differential associations between <i>a priori</i> diet quality scores and markers of cardiovascular health in women: cross-sectional analyses from TwinsUK. Br J Nutr. 2021 Oct 14;126(7):1017-1027. doi: 10.1017/S000711452000495X. Epub 2020 Dec 10. PMID: 33298202.

369: Wang S, Liu Y, Cai H, Li Y, Zhang X, Liu J, Sun R, Fang S, Yu B. Decreased risk of all-cause and heart-specific mortality is associated with low-fat or skimmed milk consumption compared with whole milk intake: A cohort study. Clin Nutr. 2021 Nov;40(11):5568-5575. doi: 10.1016/j.clnu.2021.09.012. Epub 2021 Sep 17. PMID: 34656953.

370: Nicoli C, Galbussera AA, Bosetti C, Franchi C, Gallus S, Mandelli S, Marcon G, Quadri P, Riso P, Riva E, Lucca U, Tettamanti M. The role of diet on the risk of dementia in the oldest old: The Monzino 80-plus population-based study. Clin Nutr. 2021 Jul;40(7):4783-4791. doi: 10.1016/j.clnu.2021.06.016. Epub 2021 Jun 18. PMID: 34242918.

371: Salwa M, Subaita F, Choudhury SR, Khalequzzaman M, Al Mamun MA, Bhuiyan MR, Haque MA. Fruit and vegetables consumption among school-going adolescents: Findings from the baseline survey of an intervention program in a semi-urban area of Dhaka, Bangladesh. PLoS One. 2021 Jun 8;16(6):e0252297. doi: 10.1371/journal.pone.0252297. PMID: 34101740; PMCID: PMC8186781.

372: Li H, Zeng X, Wang Y, Zhang Z, Zhu Y, Li X, Hu A, Zhao Q, Yang W. A prospective study of healthful and unhealthful plant-based diet and risk of overall and cause-specific mortality. Eur J Nutr. 2022 Feb;61(1):387-398. doi: 10.1007/s00394-021-02660-7. Epub 2021 Aug 11. PMID: 34379193.

373: Kyprianidou M, Panagiotakos D, Faka A, Kambanaros M, Makris KC, Christophi CA. Adherence to the Mediterranean diet in Cyprus and its relationship to multi-morbidity: an epidemiological study. Public Health Nutr. 2021 Oct;24(14):4546-4555. doi: 10.1017/S1368980020004267. Epub 2020 Oct 27. PMID: 33106201.

374: Esposito S, Gialluisi A, Costanzo S, Di Castelnuovo A, Ruggiero E, De Curtis A, Persichillo M, Cerletti C, Donati MB, de Gaetano G, Iacoviello L, Bonaccio M, On Behalf Of The Investigators For The Moli-Sani Study. Dietary Polyphenol Intake Is Associated with Biological Aging, a Novel Predictor of Cardiovascular Disease: Cross-Sectional Findings from the Moli-Sani Study. Nutrients. 2021 May 17;13(5):1701. doi: 10.3390/nu13051701. PMID: 34067821; PMCID: PMC8157169.

375: Toh DWK, Xia X, Sutanto CN, Low JHM, Poh KK, Wang JW, Foo RS, Kim JE. Enhancing the cardiovascular protective effects of a healthy dietary pattern with wolfberry (Lycium barbarum): A randomized controlled trial. Am J Clin Nutr. 2021 Jul 1;114(1):80-89. doi: 10.1093/ajcn/nqab062. Erratum in: Am J Clin Nutr. 2021 Jul 1;114(1):397. PMID: 33964853.

376: Sadat S, Salehi-Sahlabadi A, Pourmasoumi M, Feizi A, Clark CCT, Akkasheh G, Ghiasvand R. A healthy dietary pattern may be associated with primary insomnia among Iranian adults: A case-control study. Int J Vitam Nutr Res. 2021 Sep;91(5-6):479-490. doi: 10.1024/0300-9831/a000644. Epub 2020 Mar 11. PMID: 32156220.

377: Dorelli B, Gallè F, De Vito C, Duranti G, Iachini M, Zaccarin M, Preziosi Standoli J, Ceci R, Romano F, Liguori G, Romano Spica V, Sabatini S, Valeriani F, Cattaruzza MS. Can Physical Activity Influence Human Gut Microbiota Composition Independently of Diet? A Systematic Review. Nutrients. 2021 May 31;13(6):1890. doi: 10.3390/nu13061890. PMID: 34072834; PMCID: PMC8228232.

378: Nestares T, Martín-Masot R, de Teresa C, Bonillo R, Maldonado J, Flor-Alemany M, Aparicio VA. Influence of Mediterranean Diet Adherence and Physical Activity on Bone Health in Celiac Children on a Gluten-Free Diet. Nutrients. 2021 May 13;13(5):1636. doi: 10.3390/nu13051636. PMID: 34068001; PMCID: PMC8152289.

379: Agarwal P, Dhana K, Barnes LL, Holland TM, Zhang Y, Evans DA, Morris MC. Unhealthy foods may attenuate the beneficial relation of a Mediterranean diet to cognitive decline. Alzheimers Dement. 2021 Jul;17(7):1157-1165. doi: 10.1002/alz.12277. Epub 2021 Jan 7. PMID: 33410584.

380: Hasdell R, Poland B, Cole D, Sheppard F, Burton L, Mah CL. Retail Food Environment Intervention Planning: Interviews With Owners and Managers of Small- and Medium-Sized Rural Food Stores. Health Promot Pract. 2021 Mar;22(2):170-173. doi: 10.1177/1524839920910376. Epub 2020 Mar 14. PMID: 32174186.

381: Kim J, Kim H, Giovannucci EL. Quality of plant-based diets and risk of

hypertension: a Korean genome and examination study. Eur J Nutr. 2021 Oct;60(7):3841-3851. doi: 10.1007/s00394-021-02559-3. Epub 2021 Apr 17. PMID: 33864513.

382: Siritharan SS, Henry A, Gow ML, Roberts LM, Yao A, Ojurovic M, O'Sullivan AJ. Maternal macro- and micronutrient intake six months after hypertensive versus normotensive pregnancy: is poor diet quality contributing to future cardiometabolic disease risk? Pregnancy Hypertens. 2021 Mar;23:196-204. doi: 10.1016/j.preghy.2020.11.002. Epub 2020 Nov 13. PMID: 33515976.

383: Garcia S, Valencia C, Amaro H. Cognitive Factors Associated with Frequency of Eating Out and Eating Takeout among Latinas. J Acad Nutr Diet. 2021 Mar;121(3):520-528. doi: 10.1016/j.jand.2020.08.089. Epub 2020 Oct 4. PMID: 33028510.

384: Barchitta M, Maugeri A, Favara G, Magnano San Lio R, Riela PM, Guarnera L, Battiato S, Agodi A. Development of a Web-App for the Ecological Momentary Assessment of Dietary Habits among College Students: The HEALTHY-UNICT Project. Nutrients. 2022 Jan 13;14(2):330. doi: 10.3390/nu14020330. PMID: 35057511; PMCID: PMC8779738.

385: Marin-Alejandre BA, Cantero I, Perez-Diaz-Del-Campo N, Monreal JI, Elorz M, Herrero JI, Benito-Boillos A, Quiroga J, Martinez-Echeverria A, Uriz-Otano JI, Huarte-Muniesa MP, Tur JA, Martinez JA, Abete I, Zulet MA. Effects of two personalized dietary strategies during a 2-year intervention in subjects with nonalcoholic fatty liver disease: A randomized trial. Liver Int. 2021 Jul;41(7):1532-1544. doi: 10.1111/liv.14818. Epub 2021 Mar 1. PMID: 33550706.

386: Rijnaarts I, de Roos NM, Wang T, Zoetendal EG, Top J, Timmer M, Bouwman EP, Hogenelst K, Witteman B, de Wit N. Increasing dietary fibre intake in healthy adults using personalised dietary advice compared with general advice: a single-blind randomised controlled trial. Public Health Nutr. 2021 Apr;24(5):1117-1128. doi: 10.1017/S1368980020002980. Epub 2020 Sep 18. PMID: 32943128; PMCID: PMC8025104.

387: Parada A, Méndez C, Espino A, Reyes Á, Santibáñez H. Adherence to a gluten-free diet and quality of life in Chilean celiac patients. Rev Esp Enferm Dig. 2021 Jun;113(6):429-431. doi: 10.17235/reed.2020.7293/2020. PMID: 33267589.

388: Payandeh N, Shahinfar H, Jafari A, Babaei N, Djafarian K, Shab-Bidar S. Mediterranean diet quality index is associated with better cardiorespiratory fitness and reduced systolic blood pressure in adults: A cross-sectional study. Clin Nutr ESPEN. 2021 Dec;46:200-205. doi: 10.1016/j.clnesp.2021.10.008. Epub 2021 Oct 20. PMID: 34857196.

389: Moroney C, O'Leary F, Flood VM. The Med-NKQ: A Reliable Mediterranean Diet Nutrition Knowledge Questionnaire for Cardiovascular Disease. Nutrients. 2021 Aug 25;13(9):2949. doi: 10.3390/nu13092949. PMID: 34578825; PMCID: PMC8471340.

390: Ferreiro B, Llopis-Salinero S, Lardies B, Granados-Colomina C, Milà-Villarroel R. Clinical and Nutritional Impact of a Semi-Elemental Hydrolyzed Whey Protein Diet in Patients with Active Crohn's Disease: A Prospective Observational Study. Nutrients. 2021 Oct 16;13(10):3623. doi: 10.3390/nu13103623. PMID: 34684624; PMCID: PMC8538212.

391: Bykowska-Derda A, Czlapka-Matyasik M, Kaluzna M, Ruchala M, Ziemnicka K. Diet quality scores in relation to fatness and nutritional knowledge in women with polycystic ovary syndrome: case-control study. Public Health Nutr. 2021 Aug;24(11):3389-3398. doi: 10.1017/S1368980020001755. Epub 2020 Jul 21. PMID: 32693854.

392: Yazdanpanah Z, Beigrezaei S, Mohseni-Takalloo S, Soltani S, Rajaie SH, Zohrabi T, Kaviani M, Forbes SC, Baker JS, Salehi-Abargouei A. Does exercise affect bone mineral density and content when added to a calorie-restricted diet? A systematic review and meta-analysis of controlled clinical trials. Osteoporos Int. 2022 Feb;33(2):339-354. doi: 10.1007/s00198-021-06187-9. Epub 2021 Oct 13. PMID: 34643754.

393: Charles D, Gethings LA, Potts JF, Burney PGJ, Garcia-Larsen V. Mass spectrometry-based metabolomics for the discovery of candidate markers of flavonoid and polyphenolic intake in adults. Sci Rep. 2021 Mar 11;11(1):5801. doi: 10.1038/s41598-021-85190-w. PMID: 33707702; PMCID: PMC7952705.

394: Wang X, Li X, Xing Y, Wang W, Li S, Zhang D, Zheng W, Shen X. Threshold Effects of Total Copper Intake on Cognitive Function in US Older Adults and the Moderating Effect of Fat and Saturated Fatty Acid Intake. J Acad Nutr Diet. 2021 Dec;121(12):2429-2442. doi: 10.1016/j.jand.2021.06.002. Epub 2021 Jul 1. PMID: 34219046.

395: Diamond DM, Alabdulgader AA, de Lorgeril M, Harcombe Z, Kendrick M, Malhotra A, O'Neill B, Ravnskov U, Sultan S, Volek JS. Dietary Recommendations for Familial Hypercholesterolaemia: an Evidence-Free Zone. BMJ Evid Based Med. 2021 Dec;26(6):295-301. doi: 10.1136/bmjebm-2020-111412. Epub 2020 Jul 5. PMID: 32631832; PMCID: PMC8639944.

396: Marchlewicz E, McCabe C, Djuric Z, Hoenerhoff M, Barks J, Tang L, Song PX, Peterson K, Padmanabhan V, Dolinoy DC. Gestational exposure to high fat diets and bisphenol A alters metabolic outcomes in dams and offspring, but produces hepatic steatosis only in dams. Chemosphere. 2022 Jan;286(Pt 2):131645. doi: 10.1016/j.chemosphere.2021.131645. Epub 2021 Jul 29. PMID: 34426127; PMCID: PMC8595757.

397: Rojo-López MI, Castelblanco E, Real J, Hernández M, Falguera M, Amigó N, Julve J, Alonso N, Franch-Nadal J, Granado-Casas M, Mauricio D. Advanced Quantitative Lipoprotein Characteristics Do Not Relate to Healthy Dietary Patterns in Adults from a Mediterranean Area. Nutrients. 2021 Dec 6;13(12):4369. doi: 10.3390/nu13124369. PMID: 34959921; PMCID: PMC8706087.

398: Santiago-Torres M, Contento I, Koch P, Tsai WY, Brickman AM, Gaffney AO, Thomson CA, Crane TE, Dominguez N, Sepulveda J, Marín-Chollom AM, Paul R, Shi Z, Ulanday KT, Hale C, Hershman D, Greenlee H. ¡Mi Vida Saludable! A randomized, controlled, 2 × 2 factorial trial of a diet and physical activity intervention among Latina breast cancer survivors: Study design and methods. Contemp Clin Trials. 2021 Nov;110:106524. doi: 10.1016/j.cct.2021.106524. Epub 2021 Aug 6. PMID: 34365016; PMCID: PMC8595705.

399: Mata-Fernández A, Hershey MS, Pastrana-Delgado JC, Sotos-Prieto M, Ruiz-Canela M, Kales SN, Martínez-González MA, Fernandez-Montero A. A Mediterranean lifestyle reduces the risk of cardiovascular disease in the "Seguimiento Universidad de Navarra" (SUN) cohort. Nutr Metab Cardiovasc Dis. 2021 Jun 7;31(6):1728-1737. doi: 10.1016/j.numecd.2021.02.022. Epub 2021 Feb 27. PMID: 33895077.

400: Askari M, Daneshzad E, Bellissimo N, Suitor K, Dorosty-Motlagh AR, Azadbakht L. Food quality score and anthropometric status among 6-year-old children: A cross-sectional study. Int J Clin Pract. 2021 Jun;75(6):e14102. doi: 10.1111/ijcp.14102. Epub 2021 Mar 11. PMID: 33630401.

401: Calvo-Malvar M, Benítez-Estévez AJ, Leis R, Sánchez-Castro J, Gude F. Changes in Dietary Patterns through a Nutritional Intervention with a Traditional Atlantic Diet: The Galiat Randomized Controlled Trial. Nutrients. 2021 Nov 25;13(12):4233. doi: 10.3390/nu13124233. PMID: 34959784; PMCID: PMC8704078.

402: Al-Awadi A, Grove J, Taylor M, Valdes A, Vijay A, Bawden S, Gowland P, Aithal G. Effects of an isoenergetic low Glycaemic Index (GI) diet on liver fat accumulation and gut microbiota composition in patients with non-alcoholic fatty liver disease (NAFLD): a study protocol of an efficacy mechanism evaluation. BMJ Open. 2021 Oct 7;11(10):e045802. doi: 10.1136/bmjopen-2020-045802. PMID: 34620653; PMCID: PMC8499287.

403: McCarthy M, Birney E. Personalized profiles for disease risk must capture all facets of health. Nature. 2021 Sep;597(7875):175-177. doi: 10.1038/d41586-021-02401-0. PMID: 34489576.

404: Ugartemendia L, Bravo R, Reuter M, Castaño MY, Plieger T, Zamoscik V, Kirsch P, Rodríguez AB. SLC6A4 polymorphisms modulate the efficacy of a tryptophan-enriched diet on age-related depression and social cognition. Clin Nutr. 2021 Apr;40(4):1487-1494. doi: 10.1016/j.clnu.2021.02.023. Epub 2021 Feb 22. PMID: 33743283.

405: Possa LO, Hinkelman JV, Santos CAD, Oliveira CA, Faria BS, Hermsdorff HHM, Rosa COB. Association of dietary total antioxidant capacity with anthropometric indicators, C-reactive protein, and clinical outcomes in hospitalized oncologic patients. Nutrition. 2021 Oct;90:111359. doi: 10.1016/j.nut.2021.111359. Epub 2021 May 27. PMID: 34243042.

406: Ruiz Herrero J, Cañedo Villarroya E, González Gutiérrez-Solana L, García Alcolea B, Gómez Fernández B, Puerta Macfarland LA, Pedrón-Giner C. Classic Ketogenic Diet and Modified Atkins Diet in SLC2A1 Positive and Negative Patients with Suspected GLUT1 Deficiency Syndrome: A Single Center Analysis of 18 Cases. Nutrients. 2021 Mar 4;13(3):840. doi: 10.3390/nu13030840. PMID: 33806661; PMCID: PMC8000344.

407: Ahmed S, Rahman T, Ripon MSH, Rashid HU, Kashem T, Md Ali MS, Khor BH, Khosla P, Karupaiah T, Daud ZAM. A Food Frequency Questionnaire for Hemodialysis Patients in Bangladesh (BDHD-FFQ): Development and Validation. Nutrients. 2021 Dec 17;13(12):4521. doi: 10.3390/nu13124521. PMID: 34960076; PMCID: PMC8707927.

408: da Rocha KF, de Araújo CR, de Morais IL, Padrão P, Moreira P, Ribeiro KDDS. Commercial foods for infants under the age of 36 months: an assessment of the availability and nutrient profile of ultra-processed foods. Public Health Nutr. 2021 Aug;24(11):3179-3186. doi: 10.1017/S1368980021001555. Epub 2021 Apr 12. PMID: 33843561.

409: Ihnatowicz P, Wątor P, Gębski J, Frąckiewicz J, Drywień ME. Are Nutritional Patterns among Polish Hashimoto Thyroiditis Patients Differentiated Internally and Related to Ailments and Other Diseases? Nutrients. 2021 Oct 20;13(11):3675. doi: 10.3390/nu13113675. PMID: 34835930; PMCID: PMC8624404.

410: Vahid F, Brito A, Le Coroller G, Vaillant M, Samouda H, Bohn T, On Behalf Of Oriscav Working Group. Dietary Intake of Adult Residents in Luxembourg Taking Part in Two Cross-Sectional Studies-ORISCAV-LUX (2007-2008) and ORISCAV-LUX 2 (2016-2017). Nutrients. 2021 Dec 7;13(12):4382. doi: 10.3390/nu13124382. PMID: 34959934; PMCID: PMC8706514.

411: McCullough D, Kirwan R, Butler T, Perez de Heredia F, Thijssen D, Lip GYH, Mills J, Davies IG. Feasibility of a high-PRotein Mediterranean-style diet and resistance Exercise in cardiac Rehabilitation patients with sarcopenic obesity (PRiMER): Study protocol for a randomised control trial. Clin Nutr ESPEN. 2021 Oct;45:492-498. doi: 10.1016/j.clnesp.2021.08.001. Epub 2021 Aug 15. PMID: 34620360.

412: Fung TT, Li Y, Bhupathiraju SN, Bromage S, Batis C, Holmes MD, Stampfer M, Hu FB, Deitchler M, Willett WC. Higher Global Diet Quality Score Is Inversely Associated with Risk of Type 2 Diabetes in US Women. J Nutr. 2021 Oct 23;151(12 Suppl 2):168S-175S. doi: 10.1093/jn/nxab195. PMID: 34689196; PMCID: PMC8542093.

413: Khushalani JS, Cudhea FP, Ekwueme DU, Ruan M, Shan Z, Harris DM, Mozaffarian D, Zhang FF. Estimated economic burden of cancer associated with suboptimal diet in the United States. Cancer Causes Control. 2022 Jan;33(1):73-80. doi: 10.1007/s10552-021-01503-4. Epub 2021 Oct 15. PMID: 34652592.

414: Gearan EC, Monzella K, Gola AA, Figueroa H. Adolescent Participants in the School Lunch Program Consume More Nutritious Lunches but Their 24-hour Diets Are

Similar to Nonparticipants. J Adolesc Health. 2021 Aug;69(2):308-314. doi: 10.1016/j.jadohealth.2020.12.003. Epub 2021 Jan 9. PMID: 33436144.

415: Zheng C, Gowda GAN, Raftery D, Neuhouser ML, Tinker LF, Prentice RL, Beresford SAA, Zhang Y, Bettcher L, Pepin R, Djukovic D, Gu H, Barding GA Jr, Song X, Lampe JW. Evaluation of potential metabolomic-based biomarkers of protein, carbohydrate and fat intakes using a controlled feeding study. Eur J Nutr. 2021 Dec;60(8):4207-4218. doi: 10.1007/s00394-021-02577-1. Epub 2021 May 15. PMID: 33991228; PMCID: PMC8572153.

416: Troeschel AN, Hartman TJ, Flanders WD, Akinyemiju T, Judd S, Bostick RM. A novel evolutionary-concordance lifestyle score is inversely associated with all-cause, all-cancer, and all-cardiovascular disease mortality risk. Eur J Nutr. 2021 Sep;60(6):3485-3497. doi: 10.1007/s00394-021-02529-9. Epub 2021 Mar 6. PMID: 33675389.

417: Dorling JL, Belsky DW, Racette SB, Das SK, Ravussin E, Redman LM, Höchsmann C, Huffman KM, Kraus WE, Kobor MS, MacIsaac JL, Lin DTS, Corcoran DL, Martin CK. Association between the FTO rs9939609 single nucleotide polymorphism and dietary adherence during a 2-year caloric restriction intervention: Exploratory analyses from CALERIE™ phase 2. Exp Gerontol. 2021 Nov;155:111555. doi: 10.1016/j.exger.2021.111555. Epub 2021 Sep 20. PMID: 34543722; PMCID: PMC8720865.

418: Alick CL, Maguire RL, Murphy SK, Fuemmeler BF, Hoyo C, House JS. Periconceptional Maternal Diet Characterized by High Glycemic Loading Is Associated with Offspring Behavior in NEST. Nutrients. 2021 Sep 13;13(9):3180. doi: 10.3390/nu13093180. PMID: 34579057; PMCID: PMC8469715.

419: Alamri FF, Khan A, Alshehri AO, Assiri A, Khan SI, Aldwihi LA, Alkathiri MA, Almohammed OA, Salamatullah AM, Alali AS, Badoghaish W, Alshamrani AA, AlRuthia Y, Alqahtani F. Association of Healthy Diet with Recovery Time from COVID-19: Results from a Nationwide Cross-Sectional Study. Int J Environ Res Public Health. 2021 Aug 4;18(16):8248. doi: 10.3390/ijerph18168248. PMID: 34443997; PMCID: PMC8394364.

420: Azevedo VZ, Dall'Alba V. Fructose intake is not associated to the risk of hepatic fibrosis in patients with Non-Alcoholic Fatty Liver Disease (NAFLD). Clin Nutr. 2021 Jun;40(6):4275-4283. doi: 10.1016/j.clnu.2021.01.022. Epub 2021 Jan 27. PMID: 33551215.

421: Gold N, Yau A, Rigby B, Dyke C, Remfry EA, Chadborn T. Effectiveness of Digital Interventions for Reducing Behavioral Risks of Cardiovascular Disease in Nonclinical Adult Populations: Systematic Review of Reviews. J Med Internet Res. 2021 May 14;23(5):e19688. doi: 10.2196/19688. PMID: 33988126; PMCID: PMC8164125.

422: Pourreza S, Khademi Z, Mirzababaei A, Yekaninejad MS, Sadeghniiat-Haghighi K, Naghshi S, Mirzaei K. Association of plant-based diet index with inflammatory markers and sleep quality in overweight and obese female adults: A cross-

sectional study. Int J Clin Pract. 2021 Sep;75(9):e14429. doi:
10.1111/ijcp.14429. Epub 2021 Jun 26. PMID: 34081826.

423: Dimidi E, Kabir B, Singh J, Ageridou A, Foster C, Ciclitira P, Dubois P, Whelan K. Predictors of adherence to a gluten-free diet in celiac disease: Do knowledge, attitudes, experiences, symptoms, and quality of life play a role? Nutrition. 2021 Oct;90:111249. doi: 10.1016/j.nut.2021.111249. Epub 2021 Mar 26. PMID: 33979762.

424: Jilcott Pitts SB, Wu Q, Truesdale KP, Rafferty AP, Haynes-Maslow L, Boys KA, McGuirt JT, Fleischhacker S, Johnson N, Kaur AP, Bell RA, Ammerman AS, Laska MN. A four-year observational study to examine the dietary impact of the North Carolina Healthy Food Small Retailer Program, 2017-2020. Int J Behav Nutr Phys Act. 2021 Mar 24;18(1):44. doi: 10.1186/s12966-021-01109-8. PMID: 33761952; PMCID: PMC7990380.

425: Alwosais EZM, Al-Ozairi E, Zafar TA, Alkandari S. Chia seed (<i>Salvia hispanica</i> L.) supplementation to the diet of adults with type 2 diabetes improved systolic blood pressure: A randomized controlled trial. Nutr Health. 2021 Jun;27(2):181-189. doi: 10.1177/0260106020981819. Epub 2021 Feb 2. PMID: 33530854.

426: Lee RE, Suh BC, Cameron C, O'Neal A, Jarrett S, O'Connor DP, Ohri-Vachaspati P, Todd M, Hughes RB. Psychometric properties of the Food Environment Assessment Survey Tool (FEAST) in people with mobility impairment. Public Health Nutr. 2021 Oct;24(15):4796-4802. doi: 10.1017/S1368980021002068. Epub 2021 May 12. PMID: 33975657.

427: Ballestero-Fernández C, Varela-Moreiras G, Úbeda N, Alonso-Aperte E. Nutritional Status in Spanish Adults with Celiac Disease Following a Long-Term Gluten-Free Diet Is Similar to Non-Celiac. Nutrients. 2021 May 12;13(5):1626. doi: 10.3390/nu13051626. PMID: 34066195; PMCID: PMC8151936.

428: Stefanolo JP, Tálamo M, Dodds S, de la Paz Temprano M, Costa AF, Moreno ML, Pinto-Sánchez MI, Smecuol E, Vázquez H, Gonzalez A, Niveloni SI, Mauriño E, Verdu EF, Bai JC. Real-World Gluten Exposure in Patients With Celiac Disease on Gluten-Free Diets, Determined From Gliadin Immunogenic Peptides in Urine and Fecal Samples. Clin Gastroenterol Hepatol. 2021 Mar;19(3):484-491.e1. doi: 10.1016/j.cgh.2020.03.038. Epub 2020 Mar 23. PMID: 32217152.

429: Mandracchia F, Llauradó E, Valls RM, Tarro L, Solà R. Evaluating Mediterranean Diet-Adherent, Healthy and Allergen-Free Meals Offered in Tarragona Province Restaurants (Catalonia, Spain): A Cross-Sectional Study. Nutrients. 2021 Jul 19;13(7):2464. doi: 10.3390/nu13072464. PMID: 34371970; PMCID: PMC8308532.

430: Matsumoto M, Hatamoto Y, Sakamoto A, Masumoto A, Murayama C, Ikemoto S. The Association between Milk Intake and Nutrient Intake Adequacy among Japanese Female Junior High School Students: A Cross-Sectional Study. Nutrients. 2021 Aug

18;13(8):2838. doi: 10.3390/nu13082838. PMID: 34444998; PMCID: PMC8398737.

431: Ptomey LT, Willis EA, Reitmeier K, Dreyer Gillette ML, Sherman JR, Sullivan DK. Comparison of energy intake assessed by image-assisted food records to doubly labelled water in adolescents with intellectual and developmental disabilities: a feasibility study. J Intellect Disabil Res. 2021 Apr;65(4):340-347. doi: 10.1111/jir.12816. Epub 2021 Jan 14. PMID: 33443319; PMCID: PMC8499687.

432: Cohen CC, Perng W, Sauder KA, Ringham BM, Bellatorre A, Scherzinger A, Stanislawski MA, Lange LA, Shankar K, Dabelea D. Associations of Nutrient Intake Changes During Childhood with Adolescent Hepatic Fat: The Exploring Perinatal Outcomes Among CHildren Study. J Pediatr. 2021 Oct;237:50-58.e3. doi: 10.1016/j.jpeds.2021.06.027. Epub 2021 Jun 24. PMID: 34171361; PMCID: PMC8478817.

433: Bromage S, Andersen CT, Tadesse AW, Passarelli S, Hemler EC, Fekadu H, Sudfeld CR, Worku A, Berhane H, Batis C, Bhupathiraju SN, Fung TT, Li Y, Stampfer MJ, Deitchler M, Willett WC, Fawzi WW. The Global Diet Quality Score is Associated with Higher Nutrient Adequacy, Midupper Arm Circumference, Venous Hemoglobin, and Serum Folate Among Urban and Rural Ethiopian Adults. J Nutr. 2021 Oct 23;151(12 Suppl 2):130S-142S. doi: 10.1093/jn/nxab264. PMID: 34689198; PMCID: PMC8564694.

434: Booshehri LG, Dugan J. Impact of the supplemental nutritional assistance program on diet-related disease morbidity among older adults. Health Serv Res. 2021 Oct;56(5):854-863. doi: 10.1111/1475-6773.13609. Epub 2021 Jan 24. PMID: 33491211; PMCID: PMC8522576.

435: Smith M, Rink E, Held S, Byker Shanks C, Miles MP. The effects of foods available through the Food Distribution Program on Indian Reservations (FDPIR) on inflammation response, appetite and energy intake. Public Health Nutr. 2021 Jul;24(10):3037-3048. doi: 10.1017/S1368980020002852. Epub 2020 Sep 1. PMID: 32867882.

436: Borsi E, Serban CL, Potre C, Potre O, Putnoky S, Samfireag M, Tudor R, Ionita I, Ionita H. High Carbohydrate Diet Is Associated with Severe Clinical Indicators, but Not with Nutrition Knowledge Score in Patients with Multiple Myeloma. Int J Environ Res Public Health. 2021 May 19;18(10):5444. doi: 10.3390/ijerph18105444. PMID: 34069672; PMCID: PMC8161065.

437: Robidoux MA, Winnepetonga D, Santosa S, Haman F. Assessing the contribution of traditional foods to food security for the Wapekeka First Nation of Canada. Appl Physiol Nutr Metab. 2021 Oct;46(10):1170-1178. doi: 10.1139/apnm-2020-0951. Epub 2021 Jul 26. PMID: 34310881.

438: Luecking CT, Vaughn AE, Burney R, Hennink-Kaminski H, Hales D, Ward DS. Fidelity and factors influencing implementation of Healthy Me, Healthy: process evaluation of a social marketing campaign for diet and physical activity

behaviors of children in childcare. Transl Behav Med. 2021 Apr 7;11(3):733-744. doi: 10.1093/tbm/ibab001. PMID: 33538306; PMCID: PMC8034246.

439: Jackson K, Kelty E, Meylan M, Tennant M. A Randomized Controlled Trial Assessing the Effects of Feeding High Water Soluble Carbohydrate (WSC) Oaten Hay Versus Low WSC Oaten Hay on Equine Peripheral Dental Caries. J Equine Vet Sci. 2021 Mar;98:103356. doi: 10.1016/j.jevs.2020.103356. Epub 2020 Dec 16. PMID: 33663727.

440: Stringer EJ BSc, Sidhu S BSc, Austin K BSc, Cosby C MSc. Nutrition Education Seminars for Prostate Cancer-Diet and Prostate Program: Evaluation and Recommendations (DAPPER Study). Can J Diet Pract Res. 2021 Mar 1;82(1):27-31. doi: 10.3148/cjdpr-2020-028. Epub 2020 Dec 15. PMID: 33320768.

441: Martinez-Perez C, San-Cristobal R, Guallar-Castillon P, Martínez-González MÁ, Salas-Salvadó J, Corella D, Castañer O, Martinez JA, Alonso-Gómez ÁM, Wärnberg J, Vioque J, Romaguera D, López-Miranda J, Estruch R, Tinahones FJ, Lapetra J, Serra-Majem L, Bueno-Cavanillas A, Tur JA, Sánchez VM, Pintó X, Gaforio JJ, Matía-Martín P, Vidal J, Vázquez C, Ros E, Bes-Rastrollo M, Babio N, Sorlí JV, Lassale C, Pérez-Sanz B, Vaquero-Luna J, Bazán MJA, Barceló-Iglesias MC, Konieczna J, Ríos AG, Bernal-López MR, Santos-Lozano JM, Toledo E, Becerra-Tomás N, Portoles O, Zomeño MD, Abete I, Moreno-Rodriguez A, Lecea-Juarez O, Nishi SK, Muñoz-Martínez J, Ordovás JM, Daimiel L. Use of Different Food Classification Systems to Assess the Association between Ultra-Processed Food Consumption and Cardiometabolic Health in an Elderly Population with Metabolic Syndrome (PREDIMED-Plus Cohort). Nutrients. 2021 Jul 20;13(7):2471. doi: 10.3390/nu13072471. PMID: 34371982; PMCID: PMC8308804.

442: Shen YC, Chang CE, Lin MN, Lin CL. Vegetarian Diet Is Associated with Lower Risk of Depression in Taiwan. Nutrients. 2021 Mar 24;13(4):1059. doi: 10.3390/nu13041059. PMID: 33805124; PMCID: PMC8064096.

443: Smith BT, Hack S, Jessri M, Arcand J, McLaren L, L'Abbé MR, Anderson LN, Hobin E, Hammond D, Manson H, Rosella LC, Manuel DG. The Equity and Effectiveness of Achieving Canada's Voluntary Sodium Reduction Guidance Targets: A Modelling Study Using the 2015 Canadian Community Health Survey-Nutrition. Nutrients. 2021 Feb 27;13(3):779. doi: 10.3390/nu13030779. PMID: 33673550; PMCID: PMC7997239.

444: Brenes JC, Gómez G, Quesada D, Kovalskys I, Rigotti A, Cortés LY, Yépez García MC, Liria-Domínguez R, Herrera-Cuenca M, Guajardo V, Fisberg RM, Leme ACB, Ferrari G, Fisberg M, On Behalf Of The Elans Study Group. Alcohol Contribution to Total Energy Intake and Its Association with Nutritional Status and Diet Quality in Eight Latina American Countries. Int J Environ Res Public Health. 2021 Dec 13;18(24):13130. doi: 10.3390/ijerph182413130. PMID: 34948740; PMCID: PMC8701082.

445: Dobson P, Burney R, Hales D, Vaughn A, Tovar A, Østbye T, Ward D. Self-Efficacy for Healthy Eating Moderates the Impact of Stress on Diet Quality Among

Family Child Care Home Providers. J Nutr Educ Behav. 2021 Apr;53(4):309-315. doi: 10.1016/j.jneb.2021.01.005. PMID: 33838763.

446: van der Fels-Klerx HJ, Smits NGE, Bremer MGEG, Schultink JM, Nijkamp MM, Castenmiller JJM, de Vries JHM. Detection of gluten in duplicate portions to determine gluten intake of coeliac disease patients on a gluten-free diet. Br J Nutr. 2021 May 14;125(9):1051-1057. doi: 10.1017/S0007114520002974. Epub 2020 Jul 28. PMID: 32723408.

447: Zhong QW, Wu YY, Xiong F, Liu M, Liu YP, Wang C, Chen YM. Higher flavonoid intake is associated with a lower progression risk of non-alcoholic fatty liver disease in adults: a prospective study. Br J Nutr. 2021 Feb 28;125(4):460-470. doi: 10.1017/S0007114520002846. Epub 2020 Jul 27. PMID: 32713378.

448: Chen X, Fan S, Lyu B, Zhang L, Yao S, Liu J, Shi Z, Wu Y. Occurrence and Dietary Intake of Organophosphate Esters via Animal-Origin Food Consumption in China: Results of a Chinese Total Diet Study. J Agric Food Chem. 2021 Nov 24;69(46):13964-13973. doi: 10.1021/acs.jafc.1c05697. Epub 2021 Nov 9. PMID: 34751562.

449: Moffat T, McKerracher L, Oresnik S, Atkinson SA, Barker M, McDonald SD, Murray-Davis B, Sloboda DM. Investigating the normalization and normative views of gestational weight gain: Balancing recommendations with the promotion and support of healthy pregnancy diets. Am J Hum Biol. 2021 Sep;33(5):e23604. doi: 10.1002/ajhb.23604. Epub 2021 May 6. PMID: 33956376.

450: Smith J, Ayre J, Jansen J, Cvejic E, McCaffery KJ, Doust J, Copp T. Impact of diagnostic labels and causal explanations for weight gain on diet intentions, cognitions and emotions: An experimental online study. Appetite. 2021 Dec 1;167:105612. doi: 10.1016/j.appet.2021.105612. Epub 2021 Jul 26. PMID: 34324910.

451: Kenđel Jovanović G, Dragaš Zubalj N, Klobučar Majanović S, Rahelić D, Rahelić V, Vučak Lončar J, Pavičić Žeželj S. The Outcome of COVID-19 Lockdown on Changes in Body Mass Index and Lifestyle among Croatian Schoolchildren: A Cross-Sectional Study. Nutrients. 2021 Oct 26;13(11):3788. doi: 10.3390/nu13113788. PMID: 34836045; PMCID: PMC8624838.

452: Chen MY, Ou SH, Yen MC, Lee MS, Chen NC, Yin CH, Chen CL. Vegetarian diet in dialysis patients: A significant gap between actual intake and current nutritional recommendations. Medicine (Baltimore). 2021 Feb 12;100(6):e24617. doi: 10.1097/MD.0000000000024617. PMID: 33578571; PMCID: PMC7886433.

453: Li J, Demirel A, Azuero A, Womack ED, Kroeger EN, McLain A, Yarar-Fisher C. Limited Association between the Total Healthy Eating Index-2015 Score and Cardiovascular Risk Factors in Individuals with Long-Standing Spinal Cord Injury: An Exploratory Study: An Exploratory Study. J Acad Nutr Diet. 2021 Nov;121(11):2260-2266. doi: 10.1016/j.jand.2021.04.010. Epub 2021 May 18. PMID: 34016562.

454: Taylor JL, Keating SE, Holland DJ, Finlayson G, King NA, Gomersall SR, Rowlands AV, Coombes JS, Leveritt MD. High intensity interval training does not result in short- or long-term dietary compensation in cardiac rehabilitation: Results from the FITR heart study. Appetite. 2021 Mar 1;158:105021. doi: 10.1016/j.appet.2020.105021. Epub 2020 Nov 6. PMID: 33161045.

455: Gallagher C, Moschonis G, Erbas B. Current methods inadequate in assessing the association between junk food intake and metabolic syndrome in children and adolescents: letter to editor. Eat Weight Disord. 2021 May;26(4):1277-1278. doi: 10.1007/s40519-020-01011-5. Epub 2020 Sep 20. PMID: 32951130.

456: Kim H, Lee K, Rebholz CM, Kim J. Association between unhealthy plant-based diets and the metabolic syndrome in adult men and women: a population-based study in South Korea. Br J Nutr. 2021 Mar 14;125(5):577-590. doi: 10.1017/S0007114520002895. Epub 2020 Jul 27. PMID: 32713361.

457: Lacerda JF, Lagos AC, Carolino E, Silva-Herdade AS, Silva M, Sousa Guerreiro C. Functional Food Components, Intestinal Permeability and Inflammatory Markers in Patients with Inflammatory Bowel Disease. Nutrients. 2021 Feb 16;13(2):642. doi: 10.3390/nu13020642. PMID: 33669400; PMCID: PMC7920414.

458: Birk N, Matsuzaki M, Fung TT, Li Y, Batis C, Stampfer MJ, Deitchler M, Willett WC, Fawzi WW, Bromage S, Kinra S, Bhupathiraju SN, Lake E. Exploration of Machine Learning and Statistical Techniques in Development of a Low-Cost Screening Method Featuring the Global Diet Quality Score for Detecting Prediabetes in Rural India. J Nutr. 2021 Oct 23;151(12 Suppl 2):110S-118S. doi: 10.1093/jn/nxab281. PMID: 34689190; PMCID: PMC8542097.

459: Vossenaar M, Solomons NW, Muslimatun S, Faber M, García OP, Monterrosa E, van Zutphen KG, Kraemer K. Nutrient Density as a Dimension of Dietary Quality: Findings of the Nutrient Density Approach in a Multi-Center Evaluation. Nutrients. 2021 Nov 10;13(11):4016. doi: 10.3390/nu13114016. PMID: 34836269; PMCID: PMC8622135.

460: Pacheco LS, Bradley RD, Denenberg JO, Anderson CAM, Allison MA. Effects of Different Allotments of Avocados on the Nutritional Status of Families: A Cluster Randomized Controlled Trial. Nutrients. 2021 Nov 11;13(11):4021. doi: 10.3390/nu13114021. PMID: 34836276; PMCID: PMC8623192.

461: Machado ÍE, Parajára MDC, Guedes LFF, Meireles AL, Menezes MC, Felisbino-Mendes MS, Verly-Junior E, Malta DC. Burden of non-communicable diseases attributable to dietary risks in Brazil, 1990-2019: an analysis of the Global Burden of Disease Study 2019. Rev Soc Bras Med Trop. 2022 Jan 28;55(suppl 1):e0282. doi: 10.1590/0037-8682-0282-2021. PMID: 35107532.

462: Williams KA, Fughhi I, Fugar S, Mazur M, Gates S, Sawyer S, Patel H, Chambers D, McDaniel R, Reiser JR, Mason T. Nutrition Intervention for Reduction

of Cardiovascular Risk in African Americans Using the 2019 American College of Cardiology/American Heart Association Primary Prevention Guidelines. Nutrients. 2021 Sep 28;13(10):3422. doi: 10.3390/nu13103422. PMID: 34684423; PMCID: PMC8537862.

463: Okuda M, Fujiwara A, Sasaki S. Adherence to the Japanese Food Guide: The Association between Three Scoring Systems and Cardiometabolic Risks in Japanese Adolescents. Nutrients. 2021 Dec 23;14(1):43. doi: 10.3390/nu14010043. PMID: 35010915; PMCID: PMC8746488.

464: Donfrancesco C, Lo Noce C, Russo O, Minutoli D, Di Lonardo A, Profumo E, Buttari B, Iacone R, Vespasiano F, Vannucchi S, Onder G, Galletti F, Galeone D, Bellisario P, Gulizia MM, Giampaoli S, Palmieri L, Strazzullo P. Trend of salt intake measured by 24-h urine collection in the Italian adult population between the 2008 and 2018 CUORE project surveys. Nutr Metab Cardiovasc Dis. 2021 Mar 10;31(3):802-813. doi: 10.1016/j.numecd.2020.10.017. Epub 2020 Oct 31. PMID: 33546949.

465: Rashid M MD, MEd, FRCP(C), Haskett J BSc, Parkinson McGraw L BSc, Noble A MD, MSc, FRCP(C), van Limbergen J MD, PhD, FRCP(C), Otley A MD, MSc, FRCP(C). Teaching Families of Children with Celiac Disease about Gluten-Free Diet Using Distributed Education: a Pilot Study. Can J Diet Pract Res. 2021 Mar 1;82(1):38-40. doi: 10.3148/cjdpr-2020-021. Epub 2020 Sep 9. PMID: 32902326.

466: Ajemu KF, Desta AA, Berhe AA, Woldegebriel AG, Bezabih NM, Negash D, Wuneh AD, Woldearegay TW. Magnitude, components and predictors of metabolic syndrome in Northern Ethiopia: Evidences from regional NCDs STEPS survey, 2016. PLoS One. 2021 Jun 21;16(6):e0253317. doi: 10.1371/journal.pone.0253317. PMID: 34153079; PMCID: PMC8216523.

467: Tettamanzi F, Bagnardi V, Louca P, Nogal A, Monti GS, Mambrini SP, Lucchetti E, Maestrini S, Mazza S, Rodriguez-Mateos A, Scacchi M, Valdes AM, Invitti C, Menni C. A High Protein Diet Is More Effective in Improving Insulin Resistance and Glycemic Variability Compared to a Mediterranean Diet-A Cross-Over Controlled Inpatient Dietary Study. Nutrients. 2021 Dec 7;13(12):4380. doi: 10.3390/nu13124380. PMID: 34959931; PMCID: PMC8707429.

468: Matsuzaki M, Birk N, Bromage S, Bowen L, Batis C, Fung TT, Li Y, Stampfer MJ, Deitchler M, Willett WC, Fawzi WW, Kinra S, Bhupathiraju SN. Validation of Global Diet Quality Score Among Nonpregnant Women of Reproductive Age in India: Findings from the Andhra Pradesh Children and Parents Study (APCAPS) and the Indian Migration Study (IMS). J Nutr. 2021 Oct 23;151(12 Suppl 2):101S-109S. doi: 10.1093/jn/nxab217. PMID: 34689191; PMCID: PMC8564710.

469: Nkambule SJ, Moodley I, Kuupiel D, Mashamba-Thompson TP. Association between food insecurity and key metabolic risk factors for diet-sensitive non-communicable diseases in sub-Saharan Africa: a systematic review and meta-analysis. Sci Rep. 2021 Mar 4;11(1):5178. doi: 10.1038/s41598-021-84344-0. PMID: 33664339; PMCID: PMC7933340.

470: Vaughn AE, Hennink-Kaminski H, Moore R, Burney R, Chittams JL, Parker P, Luecking CT, Hales D, Ward DS. Evaluating a child care-based social marketing approach for improving children's diet and physical activity: results from the Healthy Me, Healthy We cluster-randomized controlled trial. Transl Behav Med. 2021 Apr 7;11(3):775-784. doi: 10.1093/tbm/ibaa113. PMID: 33231679; PMCID: PMC8033596.

471: Bromage S, Zhang Y, Holmes MD, Sachs SE, Fanzo J, Remans R, Sachs JD, Batis C, Bhupathiraju SN, Fung TT, Li Y, Stampfer MJ, Deitchler M, Willett WC, Fawzi WW. The Global Diet Quality Score Is Inversely Associated with Nutrient Inadequacy, Low Midupper Arm Circumference, and Anemia in Rural Adults in Ten Sub-Saharan African Countries. J Nutr. 2021 Oct 23;151(12 Suppl 2):119S-129S. doi: 10.1093/jn/nxab161. PMID: 34689197; PMCID: PMC8542095.

472: Raffner Basson A, Gomez-Nguyen A, LaSalla A, Buttó L, Kulpins D, Warner A, Di Martino L, Ponzani G, Osme A, Rodriguez-Palacios A, Cominelli F. Replacing Animal Protein with Soy-Pea Protein in an "American Diet" Controls Murine Crohn Disease-Like Ileitis Regardless of Firmicutes: Bacteroidetes Ratio. J Nutr. 2021 Mar 11;151(3):579-590. doi: 10.1093/jn/nxaa386. PMID: 33484150; PMCID: PMC7948210.

473: Iyer P, Beck EJ, Walton KL. A systematic review of the effect of dietary interventions on cardiovascular disease risk in adults with spinal cord injury. J Spinal Cord Med. 2021 Mar;44(2):184-203. doi: 10.1080/10790268.2019.1592926. Epub 2019 Apr 4. PMID: 30945998; PMCID: PMC7952075.

474: Gómez-Donoso C, Martínez-González MÁ, Perez-Cornago A, Sayón-Orea C, Martínez JA, Bes-Rastrollo M. Association between the nutrient profile system underpinning the Nutri-Score front-of-pack nutrition label and mortality in the SUN project: A prospective cohort study. Clin Nutr. 2021 Mar;40(3):1085-1094. doi: 10.1016/j.clnu.2020.07.008. Epub 2020 Jul 17. PMID: 32768318.

475: Rozanski A, Gransar H, Hayes SW, Friedman JD, Thomson LEJ, Berman DS. Feasibility of Using an Ultrashort Lifestyle Questionnaire to Predict Future Mortality Risk among Patients with Suspected Heart Disease. Am J Cardiol. 2021 Aug 15;153:36-42. doi: 10.1016/j.amjcard.2021.05.014. Epub 2021 Jun 30. PMID: 34215356.

476: Andueza N, Navas-Carretero S, Cuervo M. Effectiveness of Nutritional Strategies on Improving the Quality of Diet of Children from 6 to 12 Years Old: A Systematic Review. Nutrients. 2022 Jan 15;14(2):372. doi: 10.3390/nu14020372. PMID: 35057552; PMCID: PMC8781853.

477: Rej A, Shaw CC, Buckle RL, Trott N, Agrawal A, Mosey K, Sanders K, Allen R, Martin S, Newton A, Robinson K, Elphick D, Chey WD, Aziz I, Sanders DS. The low FODMAP diet for IBS; A multicentre UK study assessing long term follow up. Dig Liver Dis. 2021 Nov;53(11):1404-1411. doi: 10.1016/j.dld.2021.05.004. Epub 2021 Jun 1. PMID: 34083153.

478: Nishida Y, Nakamura H, Sasaki S, Shirahata T, Sato H, Yogi S, Yamada Y, Nakae S, Tanaka S, Katsukawa F. Evaluation of energy intake by brief-type self-administered diet history questionnaire among male patients with stable/at risk for chronic obstructive pulmonary disease. BMJ Open Respir Res. 2021 Aug;8(1):e000807. doi: 10.1136/bmjresp-2020-000807. PMID: 34362765; PMCID: PMC8351478.

479: Skoczek-Rubińska A, Muzsik-Kazimierska A, Chmurzynska A, Jamka M, Walkowiak J, Bajerska J. Inflammatory Potential of Diet Is Associated with Biomarkers Levels of Inflammation and Cognitive Function among Postmenopausal Women. Nutrients. 2021 Jul 6;13(7):2323. doi: 10.3390/nu13072323. PMID: 34371834; PMCID: PMC8308633.

480: Kirkham AA, Beka V, Prado CM. The effect of caloric restriction on blood pressure and cardiovascular function: A systematic review and meta-analysis of randomized controlled trials. Clin Nutr. 2021 Mar;40(3):728-739. doi: 10.1016/j.clnu.2020.06.029. Epub 2020 Jul 1. PMID: 32675017.

481: Rubinfeld G, Driggin E, Woolf K, Slater J, Newman JD, Heffron S, Shah B. Factors associated with participation in a short-term dietary intervention study among patients with established coronary artery disease: insights from the EVADE CAD trial. Coron Artery Dis. 2021 May 1;32(3):258-260. doi: 10.1097/MCA.0000000000000925. PMID: 32639244; PMCID: PMC7779744.

482: Hori N, Shiraishi M, Harada R, Kurashima Y. Association of Lifestyle Changes Due to the COVID-19 Pandemic with Nutrient Intake and Physical Activity Levels during Pregnancy in Japan. Nutrients. 2021 Oct 26;13(11):3799. doi: 10.3390/nu13113799. PMID: 34836056; PMCID: PMC8625567.

483: Schönenberger KA, Schüpfer AC, Gloy VL, Hasler P, Stanga Z, Kaegi-Braun N, Reber E. Effect of Anti-Inflammatory Diets on Pain in Rheumatoid Arthritis: A Systematic Review and Meta-Analysis. Nutrients. 2021 Nov 24;13(12):4221. doi: 10.3390/nu13124221. PMID: 34959772; PMCID: PMC8706441.

484: Roca M, Donat E, Masip E, Crespo-Escobar P, Cañada-Martínez AJ, Polo B, Ribes-Koninckx C. Analysis of gluten immunogenic peptides in feces to assess adherence to the gluten-free diet in pediatric celiac patients. Eur J Nutr. 2021 Jun;60(4):2131-2140. doi: 10.1007/s00394-020-02404-z. Epub 2020 Oct 15. PMID: 33057793.

485: Russo GL, Siani A, Fogliano V, Geleijnse JM, Giacco R, Giampaoli S, Iacoviello L, Kromhout D, Lionetti L, Naska A, Pellegrini N, Riccardi G, Sofi F, Vitale M, Strazzullo P. The Mediterranean diet from past to future: Key concepts from the second "Ancel Keys" International Seminar. Nutr Metab Cardiovasc Dis. 2021 Mar 10;31(3):717-732. doi: 10.1016/j.numecd.2020.12.020. Epub 2021 Jan 2. PMID: 33558092.

486: Maitland SB, Brauer P, Mutch DM, Royall D, Klein D, Tremblay A, Rheaume C,

Dhaliwal R, Jeejeebhoy K. Evaluation of Latent Models Assessing Physical Fitness and the Healthy Eating Index in Community Studies: Time-, Sex-, and Diabetes-Status Invariance. Nutrients. 2021 Nov 26;13(12):4258. doi: 10.3390/nu13124258. PMID: 34959810; PMCID: PMC8708138.

487: Parastouei K, Sepandi M, Eskandari E. Predicting the 10-year risk of cardiovascular diseases and its relation to healthy diet indicator in Iranian military personnel. BMC Cardiovasc Disord. 2021 Sep 5;21(1):419. doi: 10.1186/s12872-021-02231-y. PMID: 34482840; PMCID: PMC8419937.

488: Vivanti A. Pilot investigation shows high sensitivity and specificity using an Abridged Subjective Global Assessment without physical examination in a tertiary hospital; pertinence for use amongst those without COVID-19 when physical distancing required during the pandemic. Clin Nutr ESPEN. 2021 Aug;44:463-465. doi: 10.1016/j.clnesp.2021.05.027. Epub 2021 Jun 9. PMID: 34330506; PMCID: PMC8187736.

489: Kołota A, Głąbska D. Analysis of Food Habits during Pandemic in a Polish Population-Based Sample of Primary School Adolescents: Diet and Activity of Youth during COVID-19 (DAY-19) Study. Nutrients. 2021 Oct 22;13(11):3711. doi: 10.3390/nu13113711. PMID: 34835967; PMCID: PMC8622037.

490: Oliván-Blázquez B, Aguilar-Latorre A, Motrico E, Gómez-Gómez I, Zabaleta-Del-Olmo E, Couso-Viana S, Clavería A, Maderuelo-Fernandez JA, Recio-Rodríguez JI, Moreno-Peral P, Casajuana-Closas M, López-Jiménez T, Bolíbar B, Llobera J, Sarasa-Bosque C, Sanchez-Perez Á, Bellón JÁ, Magallón-Botaya R. The Relationship between Adherence to the Mediterranean Diet, Intake of Specific Foods and Depression in an Adult Population (45-75 Years) in Primary Health Care. A Cross-Sectional Descriptive Study. Nutrients. 2021 Aug 7;13(8):2724. doi: 10.3390/nu13082724. PMID: 34444884; PMCID: PMC8399773.

491: Stupin A, Drenjančević I, Šušnjara P, Debeljak Ž, Kolobarić N, Jukić I, Mihaljević Z, Martinović G, Selthofer-Relatić K. Is There Association between Altered Adrenergic System Activity and Microvascular Endothelial Dysfunction Induced by a 7-Day High Salt Intake in Young Healthy Individuals. Nutrients. 2021 May 20;13(5):1731. doi: 10.3390/nu13051731. PMID: 34065261; PMCID: PMC8161165.

492: Mariath AB, Martins APB. Ultra-processed food industry regulation for tackling obesity and diet-related non-communicable diseases in the Brazilian legislature: many proposals, no enactments. Public Health Nutr. 2021 Jul;24(10):3110-3115. doi: 10.1017/S1368980020002530. Epub 2020 Aug 13. PMID: 32787985.

493: Bolesławska I, Kowalówka M, Dobrzyńska M, Karaźniewicz-Łada M, Przysławski J. Differences in the Concentration of Vitamin D Metabolites in Plasma Due to the Low-Carbohydrate-High-Fat Diet and the Eastern European Diet-A Pilot Study. Nutrients. 2021 Aug 13;13(8):2774. doi: 10.3390/nu13082774. PMID: 34444934; PMCID: PMC8400948.

494: Hernáez Á, Lassale C, Castro-Barquero S, Ros E, Tresserra-Rimbau A, Castañer O, Pintó X, Vázquez-Ruiz Z, Sorlí JV, Salas-Salvadó J, Lapetra J, Gómez-Gracia E, Alonso-Gómez ÁM, Fiol M, Serra-Majem L, Sacanella E, Razquin C, Corella D, Guasch-Ferré M, Cofán M, Estruch R. Mediterranean Diet Maintained Platelet Count within a Healthy Range and Decreased Thrombocytopenia-Related Mortality Risk: A Randomized Controlled Trial. Nutrients. 2021 Feb 8;13(2):559. doi: 10.3390/nu13020559. PMID: 33567733; PMCID: PMC7915168.

495: Crabtree CD, Kackley ML, Buga A, Fell B, LaFountain RA, Hyde PN, Sapper TN, Kraemer WJ, Scandling D, Simonetti OP, Volek JS. Comparison of Ketogenic Diets with and without Ketone Salts versus a Low-Fat Diet: Liver Fat Responses in Overweight Adults. Nutrients. 2021 Mar 17;13(3):966. doi: 10.3390/nu13030966. PMID: 33802651; PMCID: PMC8002465.

496: Cao S, Zhu Z, Zhou J, Li W, Dong Y, Qian Y, Wei P, Wu M. Associations of one-carbon metabolism-related gene polymorphisms with breast cancer risk are modulated by diet, being higher when adherence to the Mediterranean dietary pattern is low. Breast Cancer Res Treat. 2021 Jun;187(3):793-804. doi: 10.1007/s10549-021-06108-8. Epub 2021 Feb 18. PMID: 33599865.

497: Larussa T, Boccuto L, Luzza F, Abenavoli L. Potential role of fecal gluten immunogenic peptides to assess dietary compliance in celiac patients. Minerva Gastroenterol (Torino). 2021 Mar;67(1):69-71. doi: 10.23736/S2724-5985.20.02710-5. Epub 2020 May 13. PMID: 32403893.

498: Piskorz D, Puente Barragán A, López Santi R, Vázquez G, Solache Ortiz G, Ramírez Zambrano L, Méndez Castillo M, Roa C, Baños M, Guzmán Ramos M, Cabral L, Sánchez P, Spitz B, López Santi MP, Estrella J, Velarde González M, Rafael Horna E, Alexander B, Baranchuk A; CorCOVID Latam Study Investigators. Psychological Impact of the Pandemic on Ambulatory Cardiometabolic Patients Without Evidence of SARS-CoV-2 Infection. The CorCOVID Latam Psy Study. Curr Probl Cardiol. 2021 Apr;46(4):100737. doi: 10.1016/j.cpcardiol.2020.100737. Epub 2020 Nov 2. PMID: 33412349; PMCID: PMC7605724.

499: Griffin BA, Mensink RP, Lovegrove JA. Reply to: "Fasting lipids are not a good way to assess the effects of diet on cardiovascular risk". Atherosclerosis. 2021 Nov;336:53-54. doi: 10.1016/j.atherosclerosis.2021.09.023. Epub 2021 Sep 29. PMID: 34607707.

500: Narula N, Wong ECL, Dehghan M, Marshall JK, Moayyedi P, Yusuf S. Does a High-inflammatory Diet Increase the Risk of Inflammatory Bowel Disease? Results From the Prospective Urban Rural Epidemiology (PURE) Study: A Prospective Cohort Study. Gastroenterology. 2021 Oct;161(4):1333-1335.e1. doi: 10.1053/j.gastro.2021.06.007. Epub 2021 Jun 10. PMID: 34118227.

501: Domínguez-López I, Marhuenda-Muñoz M, Tresserra-Rimbau A, Hernáez Á, Moreno JJ, Martínez-González MÁ, Salas-Salvadó J, Corella D, Fitó M, Martínez JA, Alonso-Gómez ÁM, Wärnberg J, Vioque J, Romaguera D, López-Miranda J, Bernal-

Lopez MR, Lapetra J, Serra-Majem JL, Bueno-Cavanillas A, Tur JA, Martín-Sánchez V, Pintó X, Delgado-Rodríguez M, Matía-Martín P, Vidal J, Vázquez C, Daimiel L, Serra-Mir M, Vázquez-Ruiz Z, Nishi SK, Sorli JV, Castañer O, Abete I, Luna JV, Carabaño-Moral R, Asencio A, Prohens L, Garcia-Rios A, Casas R, Gomez-Perez AM, Santos-Lozano JM, Razquin C, Martínez MÁ, Saiz C, Robledo-Pastor V, Zulet MA, Salaverria I, Eguaras S, Babio N, Malcampo M, Ros E, Estruch R, López-Sabater MC, Lamuela-Raventós RM. Fruit and Vegetable Consumption is Inversely Associated with Plasma Saturated Fatty Acids at Baseline in Predimed Plus Trial. Mol Nutr Food Res. 2021 Sep;65(17):e2100363. doi: 10.1002/mnfr.202100363. Epub 2021 Jul 28. PMID: 34273124.

502: Clarke ED, Rollo ME, Collins CE, Wood L, Callister R, Schumacher T, Haslam RL. Changes in vegetable and fruit intakes and effects on anthropometric outcomes in males and females. Nutr Diet. 2021 Apr;78(2):192-201. doi: 10.1111/1747-0080.12638. Epub 2020 Sep 2. PMID: 32881281.

503: Morales A, Muñoz G, Corral C, Espinoza I, Fuentes AD, Cavalla F, Baeza M, Jara G, Giacaman RA, Suazo C, Bevensee I, Gamonal J. Developing a protocol for a preventive oral health exam for elderly people (EDePAM) using E-Delphi methodology. Braz Oral Res. 2022 Jan 14;36:e013. doi: 10.1590/1807-3107bor-2022.vol36.0013. PMID: 35081230.

504: Rossi M, Mascaretti F, Parpinel M, Serraino D, Crispo A, Celentano E, Giacosa A, La Vecchia C. Dietary intake of branched-chain amino acids and colorectal cancer risk. Br J Nutr. 2021 Jul 14;126(1):22-27. doi: 10.1017/S0007114520003724. Epub 2020 Sep 23. PMID: 32962776.

505: Wright KD, Klatt MD, Adams IR, Nguyen CM, Mion LC, Tan A, Monroe TB, Rose KM, Scharre DW. Mindfulness in Motion and Dietary Approaches to Stop Hypertension (DASH) in Hypertensive African Americans. J Am Geriatr Soc. 2021 Mar;69(3):773-778. doi: 10.1111/jgs.16947. Epub 2020 Nov 23. PMID: 33227157; PMCID: PMC8329944.

506: Zhang S, Liu X, Wang T, Chen L, Yang T, Huang P, Qin J. Association and interaction effect of UCP2 gene polymorphisms and dietary factors with congenital heart diseases in Chinese Han population. Sci Rep. 2021 Apr 22;11(1):8699. doi: 10.1038/s41598-021-88057-2. PMID: 33888769; PMCID: PMC8062668.

507: Malta MB, Gomes CB, Barros AJD, Baraldi LG, Takito MY, Benício MHD, Carvalhaes MABL. Effectiveness of an intervention focusing on diet and walking during pregnancy in the primary health care service. Cad Saude Publica. 2021 May 24;37(5):e00010320. doi: 10.1590/0102-311X00010320. PMID: 34037070.

508: Zhang S, Gu Y, Bian S, Górska MJ, Zhang Q, Liu L, Meng G, Yao Z, Wu H, Wang Y, Zhang T, Wang X, Sun S, Wang X, Zhou M, Jia Q, Song K, Qi L, Niu K. Dietary patterns and risk of non-alcoholic fatty liver disease in adults: A prospective cohort study. Clin Nutr. 2021 Oct;40(10):5373-5382. doi: 10.1016/j.clnu.2021.08.021. Epub 2021 Sep 4. PMID: 34560608.

509: Walker DM, DePuccio MJ, Hefner JL, Garner JA, Joseph JJ, Headings A, Clark A. Utilization Patterns of a Food Referral Program: Findings from the Mid-Ohio Farmacy. J Am Board Fam Med. 2021 Nov-Dec;34(6):1174-1182. doi: 10.3122/jabfm.2021.06.210036. PMID: 34772772.

510: Tagliamonte S, Laiola M, Ferracane R, Vitale M, Gallo MA, Meslier V, Pons N, Ercolini D, Vitaglione P. Mediterranean diet consumption affects the endocannabinoid system in overweight and obese subjects: possible links with gut microbiome, insulin resistance and inflammation. Eur J Nutr. 2021 Oct;60(7):3703-3716. doi: 10.1007/s00394-021-02538-8. Epub 2021 Mar 24. PMID: 33763720; PMCID: PMC8437855.

511: Huybrechts I, Miglio R, Mistura L, Grioni S, Pozzebon I, Odorifero C, Borea R, Gitto A, Terrafino M, Scipioni M, Turrini A, Krogh V, Ricci S, Martucci G, Longhi A. Relative Validity of an Italian EPIC Food Frequency Questionnaire for Dietary Factors in Children and Adolescents. A Rizzoli Orthopedic Institute Study. Nutrients. 2021 Apr 9;13(4):1245. doi: 10.3390/nu13041245. PMID: 33918879; PMCID: PMC8069881.

512: Polak E, Stępień AE, Gol O, Tabarkiewicz J. Potential Immunomodulatory Effects from Consumption of Nutrients in Whole Foods and Supplements on the Frequency and Course of Infection: Preliminary Results. Nutrients. 2021 Apr 1;13(4):1157. doi: 10.3390/nu13041157. PMID: 33915705; PMCID: PMC8065427.

513: Rubin LH, Gustafson DR, Warrior L, Sheira L, Fitzgerald KC, Dastgheyb R, Weber KM, Tien PC, French A, Spence AB, Sharma A, Williams DW, White CJ, Seaberg EC, Frongillo EA, Weiser SD. Dietary intake is associated with neuropsychological impairment in women with HIV. Am J Clin Nutr. 2021 Jul 1;114(1):378-389. doi: 10.1093/ajcn/nqab038. PMID: 33829235; PMCID: PMC8246600.

514: McCullough ML, Wang Y, Hartman TJ, Hodge RA, Flanders WD, Stevens VL, Sampson L, Mitchell DC, Patel AV. The Cancer Prevention Study-3 FFQ Is a Reliable and Valid Measure of Nutrient Intakes among Racial/Ethnic Subgroups, Compared with 24-Hour Recalls and Biomarkers. J Nutr. 2021 Mar 11;151(3):636-648. doi: 10.1093/jn/nxaa358. PMID: 33484132.

515: Rouvroye MD, Slottje P, van Gils T, Mulder CJ, Muris JW, Walstock D, Reinders M, Bouma G. Insight in the diagnosis and treatment of coeliac disease in general practice: A survey and case vignette study among 106 general practitioners. Eur J Gen Pract. 2021 Dec;27(1):313-319. doi: 10.1080/13814788.2021.1985455. PMID: 34743668; PMCID: PMC8583831.

516: Liu XB, Wang J, Li YJ, Tan HX, Liu YY, Yu W, Zhang Y, Yang LC, Piao JH, Liu XL, Yang XG. Suggested Sample Size of 24-hour Urine Collection in Assessing Iodine Status among Adult Males with Insufficient Iodine Intake. Biomed Environ Sci. 2021 Apr 20;34(4):324-329. doi: 10.3967/bes2021.042. PMID: 33894813.

517: Robles B, Jewell MP, Thomas Tobin CS, Smith LV, Kuo T. Varying levels of

depressive symptoms and lifestyle health behaviors in a low income, urban population. J Behav Med. 2021 Apr;44(2):212-221. doi: 10.1007/s10865-020-00179-6. Epub 2020 Sep 16. PMID: 32936373.

518: Gao Z, Wu F, Lv G, Zhuang X, Ma G. Development and Validity of a General Nutrition Knowledge Questionnaire (GNKQ) for Chinese Adults. Nutrients. 2021 Dec 3;13(12):4353. doi: 10.3390/nu13124353. PMID: 34959905; PMCID: PMC8707636.

519: El Kinany K, Mint Sidi Deoula M, Hatime Z, Boudouaya HA, Atassi M, El Asri A, Benslimane A, Nejjari C, Ibrahimi SA, Lagiou P, El Rhazi K. Modified Mediterranean diet score adapted to a southern Mediterranean population and its relation to overweight and obesity risk. Public Health Nutr. 2021 Sep;24(13):4064-4070. doi: 10.1017/S1368980020002062. Epub 2020 Jul 29. PMID: 32723406.

520: Robinson K, Rozga M, Braakhuis A, Ellis A, Monnard CR, Sinley R, Wanner A, Vargas AJ. Effect of Incorporating Genetic Testing Results into Nutrition Counseling and Care on Dietary Intake: An Evidence Analysis Center Systematic Review-Part I. J Acad Nutr Diet. 2021 Mar;121(3):553-581.e3. doi: 10.1016/j.jand.2020.04.001. Epub 2020 Jul 3. PMID: 32624394.

521: Udeh-Momoh CT, Watermeyer T, Sindi S, Giannakopoulou P, Robb CE, Ahmadi-Abhari S, Zheng B, Waheed A, McKeand J, Salman D, Beaney T, de Jager Loots CA, Price G, Atchison C, Car J, Majeed A, McGregor AH, Kivipelto M, Ward H, Middleton LT. Health, Lifestyle, and Psycho-Social Determinants of Poor Sleep Quality During the Early Phase of the COVID-19 Pandemic: A Focus on UK Older Adults Deemed Clinically Extremely Vulnerable. Front Public Health. 2021 Oct 28;9:753964. doi: 10.3389/fpubh.2021.753964. PMID: 34869170; PMCID: PMC8637825.

522: Salamanca-González P, Valls-Zamora RM, Pedret-Figuerola A, Sorlí-Aguilar M, Santigosa-Ayala A, Catalin RE, Pallejà-Millán M, Solà-Alberich R, Martin-Lujan F, The Cenit Research Group Investigators. Effectiveness of a Motivational Nutritional Intervention through Social Networks 2.0 to Increase Adherence to the Mediterranean Diet and Improve Lung Function in Active Smokers: The DIET Study, a Randomized, Controlled and Parallel Clinical Trial in Primary Care. Nutrients. 2021 Oct 14;13(10):3597. doi: 10.3390/nu13103597. PMID: 34684600; PMCID: PMC8538243.

523: Shimizu A, Fujishima I, Maeda K, Murotani K, Kayashita J, Ohno T, Nomoto A, Ueshima J, Ishida Y, Inoue T, Mori N. Texture-Modified Diets are Associated with Poor Appetite in Older Adults who are Admitted to a Post-Acute Rehabilitation Hospital. J Am Med Dir Assoc. 2021 Sep;22(9):1960-1965. doi: 10.1016/j.jamda.2021.05.018. Epub 2021 Jun 14. PMID: 34139151.

524: Wabo TMC, Nkondjock VRN, Onwuka JU, Sun C, Han T, Sira J. Association of fourteen years diet quality trajectories and type 2 diabetes mellitus with related biomarkers. Aging (Albany NY). 2021 Mar 26;13(7):10112-10127. doi: 10.18632/aging.202771. Epub 2021 Mar 26. PMID: 33819180; PMCID: PMC8064195.

525: Martínez-Rodríguez A, Martínez-Olcina M, Hernández-García M, Rubio-Arias JÁ, Sánchez-Sánchez J, Lara-Cobos D, Vicente-Martínez M, Carvalho MJ, Sánchez-Sáez JA. Mediterranean Diet Adherence, Body Composition and Performance in Beach Handball Players: A Cross Sectional Study. Int J Environ Res Public Health. 2021 Mar 10;18(6):2837. doi: 10.3390/ijerph18062837. PMID: 33802192; PMCID: PMC7999029.

526: Bowden K, Gray NA, Swanepoel E, Wright HH. A Mediterranean lifestyle is associated with favourable cardiometabolic markers in people with non-dialysis dependent chronic kidney disease. J Nutr Sci. 2021 Jun 4;10:e42. doi: 10.1017/jns.2021.33. PMID: 34164121; PMCID: PMC8190716.

527: Allen A, Perera S, Mettananda S, Rodrigo R, Perera L, Darshana T, Moggach F, Jackson Crawford A, Heirene L, Fisher C, Olivieri N, Rees D, Premawardhena A, Allen S. Oxidative status in the β-thalassemia syndromes in Sri Lanka; a cross-sectional survey. Free Radic Biol Med. 2021 Apr;166:337-347. doi: 10.1016/j.freeradbiomed.2021.02.028. Epub 2021 Mar 4. PMID: 33677065.

528: Craig WJ, Brothers CJ. Nutritional Content and Health Profile of Non-Dairy Plant-Based Yogurt Alternatives. Nutrients. 2021 Nov 14;13(11):4069. doi: 10.3390/nu13114069. PMID: 34836324; PMCID: PMC8619131.

529: Włodarczyk M, Śliżewska K. Efficiency of Resistant Starch and Dextrins as Prebiotics: A Review of the Existing Evidence and Clinical Trials. Nutrients. 2021 Oct 26;13(11):3808. doi: 10.3390/nu13113808. PMID: 34836063; PMCID: PMC8621223.

530: Mota JO, Guillou S, Pierre F, Membré JM. Public health risk-benefit assessment of red meat in France: Current consumption and alternative scenarios. Food Chem Toxicol. 2021 Mar;149:111994. doi: 10.1016/j.fct.2021.111994. Epub 2021 Jan 21. PMID: 33484790.

531: Martini D, Godos J, Marventano S, Tieri M, Ghelfi F, Titta L, Lafranconi A, Trigueiro H, Gambera A, Alonzo E, Sciacca S, Buscemi S, Ray S, Galvano F, Del Rio D, Grosso G. Nut and legume consumption and human health: an umbrella review of observational studies. Int J Food Sci Nutr. 2021 Nov;72(7):871-878. doi: 10.1080/09637486.2021.1880554. Epub 2021 Feb 4. PMID: 33541169.

532: Jafari A, Ghanbari M, Shahinfar H, Bellissimo N, Azadbakht L. The association between dietary acid load with cardiometabolic risk factors and inflammatory markers amongst elderly men: A cross-sectional study. Int J Clin Pract. 2021 Jun;75(6):e14109. doi: 10.1111/ijcp.14109. Epub 2021 Mar 5. PMID: 33624383.

533: Han S, Gao H, Song R, Zhang W, Li Y, Zhang J. Oat Fiber Modulates Hepatic Circadian Clock via Promoting Gut Microbiota-Derived Short Chain Fatty Acids. J Agric Food Chem. 2021 Dec 29;69(51):15624-15635. doi: 10.1021/acs.jafc.1c06130. Epub 2021 Dec 20. PMID: 34928598.

534: Vandevijvere S, De Ridder K, Drieskens S, Charafeddine R, Berete F, Demarest S. Food insecurity and its association with changes in nutritional habits among adults during the COVID-19 confinement measures in Belgium. Public Health Nutr. 2021 Apr;24(5):950-956. doi: 10.1017/S1368980020005005. Epub 2020 Dec 9. PMID: 33292888; PMCID: PMC7804079.

535: Nnadozie UU, Asouzu NC, Asouzu NC, Anekwu EM, Obayi NOK, Maduba CC, Anamazobi AE, Anikwe CC, Nnolim IB, Nnadozie AA. Feeding behavior among health-care workers in a tertiary health institution Southeast Nigeria. Ann Afr Med. 2021 Jul-Sep;20(3):169-177. doi: 10.4103/aam.aam_25_20. PMID: 34558445; PMCID: PMC8477277.

536: Lavaee F, Shahrokhi Sardo M, Zarei F, Shahrokhi Sardo M. Comparison of Serum and Dietary Selenium Levels in Participants with a Positive History of Recurrent Herpes Lesions and Healthy Individuals. Biomed Res Int. 2021 Dec 31;2021:6083716. doi: 10.1155/2021/6083716. PMID: 35005019; PMCID: PMC8741354.

537: Liu L, Huang X, Wang B, Song Y, Lin T, Zhou Z, Guo H, Chen P, Yang Y, Ling W, Qin X, Tang G, Liu C, Li J, Zhang Y, Spence JD, Huo Y, Zhang H, Xu X. Egg consumption associated with all-cause mortality in rural China: a 14-year follow-up study. Eur J Public Health. 2021 Jul 13;31(3):613-618. doi: 10.1093/eurpub/ckaa250. PMID: 33954663.

538: Guo F, Zhang Q, Jiang H, He Y, Li M, Ran J, Lin J, Tian L, Ma L. Dietary potato intake and risks of type 2 diabetes and gestational diabetes mellitus. Clin Nutr. 2021 Jun;40(6):3754-3764. doi: 10.1016/j.clnu.2021.04.039. Epub 2021 May 1. PMID: 34130021.

539: Doyev R, Axelrod R, Keinan-Boker L, Shimony T, Goldsmith R, Nitsan L, Dichtiar R, Sinai T. Energy Intake Is Highly Associated with Handgrip Strength in Community-Dwelling Elderly Adults. J Nutr. 2021 May 11;151(5):1249-1255. doi: 10.1093/jn/nxaa451. PMID: 33693862.

540: Iwasaki M, Ennibi OK, Bouziane A, Erraji S, Lakhdar L, Rhissassi M, Ansai T, Yoshida A, Miyazaki H. Association between periodontitis and the Mediterranean diet in young Moroccan individuals. J Periodontal Res. 2021 Apr;56(2):408-414. doi: 10.1111/jre.12833. Epub 2020 Dec 31. PMID: 33381869.

541: Francis D, Ghazanfar S, Havula E, Krycer JR, Strbenac D, Senior A, Minard AY, Geddes T, Nelson ME, Weiss F, Stöckli J, Yang JYH, James DE. Genome-wide analysis in Drosophila reveals diet-by-gene interactions and uncovers diet-responsive genes. G3 (Bethesda). 2021 Sep 27;11(10):jkab171. doi: 10.1093/g3journal/jkab171. PMID: 34568906; PMCID: PMC8496270.

542: Conte M, Rozza F, Fucile I, D'Avino G, Sorvillo G, De Luca N, Mancusi C. Low Awareness of Cardiovascular Risk Factor Among Patients Admitted in Cardiac Rehabilitation Unit. High Blood Press Cardiovasc Prev. 2021 May;28(3):321-324. doi: 10.1007/s40292-021-00444-y. Epub 2021 Mar 12. PMID: 33710600.

543: Ibsen DB, Overvad K, Laursen ASD, Halkjær J, Tjønneland A, Kilpeläinen TO, Parner ET, Jakobsen MU. Changes in intake of dairy product subgroups and risk of type 2 diabetes: modelling specified food substitutions in the Danish Diet, Cancer and Health cohort. Eur J Nutr. 2021 Sep;60(6):3449-3459. doi: 10.1007/s00394-021-02524-0. Epub 2021 Mar 4. PMID: 33661378.

544: Methuen M, Kauppinen S, Suominen AL, Eloranta AM, Väistö J, Lakka T, Vähänikkilä H, Anttonen V. Dental caries among Finnish teenagers participating in physical activity and diet intervention: association with anthropometrics and behavioural factors. BMC Oral Health. 2021 Jul 6;21(1):333. doi: 10.1186/s12903-021-01690-1. PMID: 34229689; PMCID: PMC8259164.

545: Oostra DL, Burse NR, Wolf LJ, Schleicher E, Mama SK, Bluethmann S, Schmitz K, Winkels RM. Understanding Nutritional Problems of Metastatic Breast Cancer Patients: Opportunities for Supportive Care Through eHealth. Cancer Nurs. 2021 Mar-Apr 01;44(2):154-162. doi: 10.1097/NCC.0000000000000788. PMID: 32022781.

546: Norwood DA, Dominguez LB, Paredes AA, Montalvan EE, Rodriguez Murillo A, Dougherty MK, Palsson OS, Dominguez RL, Morgan DR. Prevalence and Associated Dietary Factors of Rome IV Functional Gastrointestinal Disorders in Rural Western Honduras. Dig Dis Sci. 2021 Sep;66(9):3086-3095. doi: 10.1007/s10620-020-06639-y. Epub 2020 Oct 22. PMID: 33089482.

547: Schmidt KA, Cromer G, Burhans MS, Kuzma JN, Hagman DK, Fernando I, Murray M, Utzschneider KM, Holte S, Kraft J, Kratz M. The impact of diets rich in low-fat or full-fat dairy on glucose tolerance and its determinants: a randomized controlled trial. Am J Clin Nutr. 2021 Mar 11;113(3):534-547. doi: 10.1093/ajcn/nqaa301. PMID: 33184632; PMCID: PMC7948850.

548: Semlitsch T, Krenn C, Jeitler K, Berghold A, Horvath K, Siebenhofer A. Long-term effects of weight-reducing diets in people with hypertension. Cochrane Database Syst Rev. 2021 Feb 8;2(2):CD008274. doi: 10.1002/14651858.CD008274.pub4. PMID: 33555049; PMCID: PMC8093137.

549: Seneviratne SN, Sachchithananthan S, Gamage PSA, Peiris R, Wickramasinghe VP, Somasundaram N. Effectiveness and acceptability of a novel school-based healthy eating program among primary school children in urban Sri Lanka. BMC Public Health. 2021 Nov 13;21(1):2083. doi: 10.1186/s12889-021-12041-8. PMID: 34774025; PMCID: PMC8590231.

550: Park S, Chae M, Park H, Park K. Higher Branched-Chain Amino Acid Intake Is Associated with Handgrip Strength among Korean Older Adults. Nutrients. 2021 Apr 30;13(5):1522. doi: 10.3390/nu13051522. PMID: 33946360; PMCID: PMC8146867.

551: Uetsuki K, Kawashima H, Ohno E, Ishikawa T, Iida T, Yamamoto K, Furukawa K, Nakamura M, Honda T, Ishigami M, Hirooka Y, Fujishiro M. Measurement of fasting breath hydrogen concentration as a simple diagnostic method for pancreatic exocrine insufficiency. BMC Gastroenterol. 2021 May 10;21(1):211. doi: 10.1186/s12876-021-01776-8. PMID: 33971823; PMCID: PMC8111728.

552: Tertsunen HM, Hantunen S, Tuomainen TP, Virtanen JK. Adherence to a healthy Nordic diet and risk of type 2 diabetes among men: the Kuopio Ischaemic Heart Disease Risk Factor Study. Eur J Nutr. 2021 Oct;60(7):3927-3934. doi: 10.1007/s00394-021-02569-1. Epub 2021 Apr 27. PMID: 33904995; PMCID: PMC8437904.

553: Alamneh YM, Akalu TY, Shiferaw AA, Atnaf A. Magnitude of anemia and associated factors among children aged 6-59 months at Debre Markos referral hospital, Northwest Ethiopia: a hospital-based cross-sectional study. Ital J Pediatr. 2021 Aug 13;47(1):172. doi: 10.1186/s13052-021-01123-3. PMID: 34389033; PMCID: PMC8362241.

554: Duan S, Li M, Zhao J, Yang H, He J, Lei L, Cheng R, Hu T. A predictive nomogram: a cross-sectional study on a simple-to-use model for screening 12-year-old children for severe caries in middle schools. BMC Oral Health. 2021 Sep 20;21(1):457. doi: 10.1186/s12903-021-01819-2. PMID: 34544417; PMCID: PMC8451146.

555: Perez-Cornago A, Crowe FL, Appleby PN, Bradbury KE, Wood AM, Jakobsen MU, Johnson L, Sacerdote C, Steur M, Weiderpass E, Würtz AML, Kühn T, Katzke V, Trichopoulou A, Karakatsani A, La Vecchia C, Masala G, Tumino R, Panico S, Sluijs I, Skeie G, Imaz L, Petrova D, Quirós JR, Yohar SMC, Jakszyn P, Melander O, Sonestedt E, Andersson J, Wennberg M, Aune D, Riboli E, Schulze MB, di Angelantonio E, Wareham NJ, Danesh J, Forouhi NG, Butterworth AS, Key TJ. Plant foods, dietary fibre and risk of ischaemic heart disease in the European Prospective Investigation into Cancer and Nutrition (EPIC) cohort. Int J Epidemiol. 2021 Mar 3;50(1):212-222. doi: 10.1093/ije/dyaa155. PMID: 33245137; PMCID: PMC7938513.

556: Ferguson GM, Meeks Gardner JM, Nelson MR, Giray C, Sundaram H, Fiese BH, Koester B, Tran SP, Powell R. Food-Focused Media Literacy for Remotely Acculturating Adolescents and Mothers: A Randomized Controlled Trial of the "JUS Media? Programme". J Adolesc Health. 2021 Dec;69(6):1013-1023. doi: 10.1016/j.jadohealth.2021.06.006. Epub 2021 Jul 17. PMID: 34281754; PMCID: PMC8628116.

557: Im J, Park K. Association between Soy Food and Dietary Soy Isoflavone Intake and the Risk of Cardiovascular Disease in Women: A Prospective Cohort Study in Korea. Nutrients. 2021 Apr 22;13(5):1407. doi: 10.3390/nu13051407. PMID: 33922001; PMCID: PMC8143453.

558: Bernier-Jean A, Prince RL, Lewis JR, Craig JC, Hodgson JM, Lim WH, Teixeira-Pinto A, Wong G. Dietary plant and animal protein intake and decline in estimated glomerular filtration rate among elderly women: a 10-year longitudinal cohort study. Nephrol Dial Transplant. 2021 Aug 27;36(9):1640-1647. doi: 10.1093/ndt/gfaa081. PMID: 32457981.

559: Foers AD, Shoukat MS, Welsh OE, Donovan K, Petry R, Evans SC, FitzPatrick ME, Collins N, Klenerman P, Fowler A, Soilleux EJ. Classification of intestinal

T-cell receptor repertoires using machine learning methods can identify patients with coeliac disease regardless of dietary gluten status. J Pathol. 2021 Mar;253(3):279-291. doi: 10.1002/path.5592. Epub 2021 Jan 6. PMID: 33225446; PMCID: PMC7898595.

560: Mohseni H, Amini S, Abiri B, Kalantar M, Kaydani M, Barati B, Pirabbasi E, Bahrami F. Are history of dietary intake and food habits of patients with clinical symptoms of COVID 19 different from healthy controls? A case-control study. Clin Nutr ESPEN. 2021 Apr;42:280-285. doi: 10.1016/j.clnesp.2021.01.021. Epub 2021 Jan 29. PMID: 33745593; PMCID: PMC7846218.

561: Zhang S, Wu X, Bian S, Zhang Q, Liu L, Meng G, Yao Z, Wu H, Gu Y, Wang Y, Sun S, Wang X, Zhou M, Jia Q, Song K, Niu K. Association between consumption frequency of honey and non-alcoholic fatty liver disease: results from a cross-sectional analysis based on the Tianjin Chronic Low-grade Systemic Inflammation and Health (TCLSIH) Cohort Study. Br J Nutr. 2021 Mar 28;125(6):712-720. doi: 10.1017/S0007114520003190. Epub 2020 Aug 17. PMID: 32799936.

562: Mozzillo E, Zito E, Calcaterra V, Corciulo N, Di Pietro M, Di Sessa A, Franceschi R, Licenziati MR, Maltoni G, Morino G, Predieri B, Street ME, Trifirò G, Gallè F, Franzese A, Valerio G. Poor Health Related Quality of Life and Unhealthy Lifestyle Habits in Weight-Loss Treatment-Seeking Youth. Int J Environ Res Public Health. 2021 Sep 4;18(17):9355. doi: 10.3390/ijerph18179355. PMID: 34501945; PMCID: PMC8431705.

563: Nishi SK, Kendall CWC, Bazinet RP, Hanley AJ, Comelli EM, Jenkins DJA, Sievenpiper JL. Almond Bioaccessibility in a Randomized Crossover Trial: Is a Calorie a Calorie? Mayo Clin Proc. 2021 Sep;96(9):2386-2397. doi: 10.1016/j.mayocp.2021.01.026. Epub 2021 Apr 11. PMID: 33853731.

564: Hanly G, Campbell E, Bartlem K, Dray J, Fehily C, Bradley T, Murray S, Lecathelinais C, Wiggers J, Wolfenden L, Reid K, Reynolds T, Bowman J. Effectiveness of referral to a population-level telephone coaching service for improving health risk behaviours in people with a mental health condition: study protocol for a randomised controlled trial. Trials. 2022 Jan 17;23(1):49. doi: 10.1186/s13063-021-05971-6. PMID: 35039058; PMCID: PMC8762844.

565: Fortier M, Castellano CA, St-Pierre V, Myette-Côté É, Langlois F, Roy M, Morin MC, Bocti C, Fulop T, Godin JP, Delannoy C, Cuenoud B, Cunnane SC. A ketogenic drink improves cognition in mild cognitive impairment: Results of a 6-month RCT. Alzheimers Dement. 2021 Mar;17(3):543-552. doi: 10.1002/alz.12206. Epub 2020 Oct 26. PMID: 33103819; PMCID: PMC8048678.

566: Cortesi PA, Fornari C, Madotto F, Conti S, Naghavi M, Bikbov B, Briant PS, Caso V, Crotti G, Johnson C, Nguyen M, Palmieri L, Perico N, Profili F, Remuzzi G, Roth GA, Traini E, Voller F, Yadgir S, Mazzaglia G, Monasta L, Giampaoli S, Mantovani LG; GBD 2017 Italy Cardiovascular Diseases Collaborators. Trends in cardiovascular diseases burden and vascular risk factors in Italy: The Global Burden of Disease study 1990-2017. Eur J Prev Cardiol. 2021 May 8;28(4):385-396.

doi: 10.1177/2047487320949414. PMID: 33966080.

567: Moludi J, Kafil HS, Qaisar SA, Gholizadeh P, Alizadeh M, Vayghyan HJ. Effect of probiotic supplementation along with calorie restriction on metabolic endotoxemia, and inflammation markers in coronary artery disease patients: a double blind placebo controlled randomized clinical trial. Nutr J. 2021 Jun 1;20(1):47. doi: 10.1186/s12937-021-00703-7. PMID: 34074289; PMCID: PMC8170788.

568: Wang Y, Gallegos JL, Haskell-Ramsay C, Lodge JK. Effects of chronic consumption of specific fruit (berries, citrus and cherries) on CVD risk factors: a systematic review and meta-analysis of randomised controlled trials. Eur J Nutr. 2021 Mar;60(2):615-639. doi: 10.1007/s00394-020-02299-w. Epub 2020 Jun 13. Erratum in: Eur J Nutr. 2021 Jan 23;: PMID: 32535781; PMCID: PMC7900084.

569: Joufi AI, Claiborne DM, Shuman D. Oral Health Education and Promotion Activities by Early Head Start Programs in the United States: A systematic review. J Dent Hyg. 2021 Oct;95(5):14-21. PMID: 34654711.

570: Viroli G, Gonçalves C, Pinho O, Silva-Santos T, Padrão P, Moreira P. High Adherence to Mediterranean Diet Is Not Associated with an Improved Sodium and Potassium Intake. Nutrients. 2021 Nov 19;13(11):4151. doi: 10.3390/nu13114151. PMID: 34836406; PMCID: PMC8623388.

571: Chen S, Ma D, Xiao S, Li P, Lei H, Huang X. Effects of chronic apical periodontitis on the inflammatory response of the aorta in hyperlipemic rats. Clin Oral Investig. 2021 Jun;25(6):3845-3852. doi: 10.1007/s00784-020-03714-6. Epub 2021 Jan 6. PMID: 33404761.

572: Grabia M, Puścion-Jakubik A, Markiewicz-Żukowska R, Bielecka J, Mielech A, Nowakowski P, Socha K. Adherence to Mediterranean Diet and Selected Lifestyle Elements among Young Women with Type 1 Diabetes Mellitus from Northeast Poland: A Case-Control COVID-19 Survey. Nutrients. 2021 Apr 2;13(4):1173. doi: 10.3390/nu13041173. PMID: 33918177; PMCID: PMC8066783.

573: Consavage Stanley K, Harrigan PB, Serrano EL, Kraak VI. Applying a Multi-Dimensional Digital Food and Nutrition Literacy Model to Inform Research and Policies to Enable Adults in the U.S. Supplemental Nutrition Assistance Program to Make Healthy Purchases in the Online Food Retail Ecosystem. Int J Environ Res Public Health. 2021 Aug 6;18(16):8335. doi: 10.3390/ijerph18168335. PMID: 34444084; PMCID: PMC8394533.

574: Wild LE, Patterson WB, Jones RB, Plows JF, Berger PK, Rios C, Fogel JL, Goran MI, Alderete TL. Risk of Micronutrient Inadequacy among Hispanic, Lactating Mothers: Preliminary Evidence from the Southern California Mother's Milk Study. Nutrients. 2021 Sep 18;13(9):3252. doi: 10.3390/nu13093252. PMID: 34579129; PMCID: PMC8465791.

575: Bolte LA, Vich Vila A, Imhann F, Collij V, Gacesa R, Peters V, Wijmenga C, Kurilshikov A, Campmans-Kuijpers MJE, Fu J, Dijkstra G, Zhernakova A, Weersma

RK. Long-term dietary patterns are associated with pro-inflammatory and anti-inflammatory features of the gut microbiome. Gut. 2021 Jul;70(7):1287-1298. doi: 10.1136/gutjnl-2020-322670. Epub 2021 Apr 2. PMID: 33811041; PMCID: PMC8223641.

576: Wang Y. Tree nut consumption is associated with higher sex hormone-binding globulin levels in premenopausal US women. Nutr Res. 2021 Sep;93:61-68. doi: 10.1016/j.nutres.2021.07.003. Epub 2021 Jul 13. PMID: 34365198.

577: Newnham ED, Clayton-Chubb D, Nagarethinam M, Hosking P, Gibson PR. Randomised clinical trial: adjunctive induction therapy with oral effervescent budesonide in newly diagnosed coeliac disease. Aliment Pharmacol Ther. 2021 Aug;54(4):419-428. doi: 10.1111/apt.16446. Epub 2021 Jun 28. PMID: 34181750.

578: Craig WJ, Fresán U. International Analysis of the Nutritional Content and a Review of Health Benefits of Non-Dairy Plant-Based Beverages. Nutrients. 2021 Mar 4;13(3):842. doi: 10.3390/nu13030842. PMID: 33806688; PMCID: PMC7999853.

579: Hajri T, Zaiou M, Fungwe TV, Ouguerram K, Besong S. Epigenetic Regulation of Peroxisome Proliferator-Activated Receptor Gamma Mediates High-Fat Diet-Induced Non-Alcoholic Fatty Liver Disease. Cells. 2021 May 31;10(6):1355. doi: 10.3390/cells10061355. PMID: 34072832; PMCID: PMC8229510.

580: Koponen S, Nykänen I, Savela RM, Välimäki T, Suominen AL, Schwab U. Inadequate Intake of Energy and Nutrients Is Common in Older Family Caregivers. Nutrients. 2021 Aug 12;13(8):2763. doi: 10.3390/nu13082763. PMID: 34444923; PMCID: PMC8400852.

581: Abdullah MMH, Hughes J, Grafenauer S. Whole Grain Intakes Are Associated with Healthcare Cost Savings Following Reductions in Risk of Colorectal Cancer and Total Cancer Mortality in Australia: A Cost-of-Illness Model. Nutrients. 2021 Aug 27;13(9):2982. doi: 10.3390/nu13092982. PMID: 34578860; PMCID: PMC8468283.

582: Chen Y, Qin Y, Zhang Z, Huang S, Jiao C, Zhang Z, Bao W, Mao L. Association of the low-carbohydrate dietary pattern with postpartum weight retention in women. Food Funct. 2021 Nov 1;12(21):10764-10772. doi: 10.1039/d1fo00935d. PMID: 34609398.

583: Desai D, Kandasamy S, Limbachia J, Zulyniak MA, Ritvo P, Sherifali D, Wahi G, Anand SS, de Souza RJ. Studies to Improve Perinatal Health through Diet and Lifestyle among South Asian Women Living in Canada: A Brief History and Future Research Directions. Nutrients. 2021 Aug 24;13(9):2932. doi: 10.3390/nu13092932. PMID: 34578810; PMCID: PMC8465246.

584: Wu L, Lo ECM, McGrath C, Wong MCM, Ho SMY, Gao X. Motivational interviewing for caries prevention in adolescents: a randomized controlled trial. Clin Oral Investig. 2022 Jan;26(1):585-594. doi: 10.1007/s00784-021-04037-w. Epub 2021 Jul 13. PMID: 34254214.

585: Shin WY, Kim JH. Poor diet quality is associated with self-reported knee pain in community-dwelling women aged 50 years and older. PLoS One. 2021 Feb 16;16(2):e0245630. doi: 10.1371/journal.pone.0245630. PMID: 33591989; PMCID: PMC7886155.

586: Rajendiran E, Lamarche B, She Y, Ramprasath V, Eck P, Brassard D, Gigleux I, Levy E, Tremblay A, Couture P, House JD, Jones PJH, Desmarchelier C. A combination of single nucleotide polymorphisms is associated with the interindividual variability in the blood lipid response to dietary fatty acid consumption in a randomized clinical trial. Am J Clin Nutr. 2021 Aug 2;114(2):564-577. doi: 10.1093/ajcn/nqab064. PMID: 33871574.

587: Calvo-Malvar M, Benítez-Estévez AJ, Sánchez-Castro J, Leis R, Gude F. Effects of a Community-Based Behavioral Intervention with a Traditional Atlantic Diet on Cardiometabolic Risk Markers: A Cluster Randomized Controlled Trial ("The GALIAT Study"). Nutrients. 2021 Apr 7;13(4):1211. doi: 10.3390/nu13041211. PMID: 33916940; PMCID: PMC8067574.

588: Bao J, Zheng S, Huang J, Xie X, Zhang J, Yang S, Wu X, Zhang Y. Mental health is correlated with lipoprotein(a) levels in male patients with premature coronary heart disease. Ann Palliat Med. 2021 Jun;10(6):6482-6492. doi: 10.21037/apm-21-1024. PMID: 34237965.

589: Rees K, Al-Khudairy L, Takeda A, Stranges S. Vegan dietary pattern for the primary and secondary prevention of cardiovascular diseases. Cochrane Database Syst Rev. 2021 Feb 25;2(2):CD013501. doi: 10.1002/14651858.CD013501.pub2. PMID: 33629376; PMCID: PMC8092640.

590: Herrera JAR, Thomsen ST, Jakobsen LS, Fagt S, Banasik K, Izarzugaza JM, Brunak S, Pires SM. The burden of disease of three food-associated heavy metals in clusters in the Danish population - Towards targeted public health strategies. Food Chem Toxicol. 2021 Apr;150:112072. doi: 10.1016/j.fct.2021.112072. Epub 2021 Feb 18. PMID: 33610621.

591: Jafari Nasab S, Rafiee P, Bahrami A, Rezaeimanesh N, Rashidkhani B, Sohrab G, Naja F, Hejazi E, Sadeghi A. Diet-dependent acid load and the risk of colorectal cancer and adenoma: a case-control study. Public Health Nutr. 2021 Oct;24(14):4474-4481. doi: 10.1017/S1368980020003420. Epub 2020 Oct 22. PMID: 33087202.

592: Devaraj SM, Miller RG, Orchard TJ, Kriska AM, Gary-Webb T, Costacou T. Data driven patterns of nutrient intake and coronary artery disease risk in adults with type 1 diabetes. J Diabetes Complications. 2021 Oct;35(10):108016. doi: 10.1016/j.jdiacomp.2021.108016. Epub 2021 Aug 4. PMID: 34391636; PMCID: PMC8434996.

593: Flanagan R, Kuo B, Staller K. Utilizing Google Trends to Assess Worldwide Interest in Irritable Bowel Syndrome and Commonly Associated Treatments. Dig Dis Sci. 2021 Mar;66(3):814-822. doi: 10.1007/s10620-020-06290-7. Epub 2020 May 2.

PMID: 32361922.

594: Lampignano L, Donghia R, Sila A, Bortone I, Tatoli R, De Nucci S, Castellana F, Zupo R, Tirelli S, Giannoccaro V, Guerra V, Panza F, Lozupone M, Mastronardi M, De Pergola G, Giannelli G, Sardone R. Mediterranean Diet and Fatty Liver Risk in a Population of Overweight Older Italians: A Propensity Score-Matched Case-Cohort Study. Nutrients. 2022 Jan 7;14(2):258. doi: 10.3390/nu14020258. PMID: 35057439; PMCID: PMC8779579.

595: Santos JV, Gorasso V, Souza J, Wyper GMA, Grant I, Pinheiro V, Viana J, Ricciardi W, Haagsma JA, Devleesschauwer B, Plass D, Freitas A. Risk factors and their contribution to population health in the European Union (EU-28) countries in 2007 and 2017. Eur J Public Health. 2021 Oct 26;31(5):958-967. doi: 10.1093/eurpub/ckab145. PMID: 34468766.

596: Oyetunji IO, Duncan A, Booley S, Harbron J. Diet quality, food insecurity and risk of cardiovascular diseases among adults living with HIV/AIDS: a scoping review protocol. BMJ Open. 2021 Oct 12;11(10):e047314. doi: 10.1136/bmjopen-2020-047314. PMID: 34642188; PMCID: PMC8513254.

597: Wormer JR, Shankar A, Van Hensbroek MB, Hindori-Mohangoo AD, Covert H, Lichtveld MY, Zijlmans WCWR. Poor Adherence to the WHO Guidelines on Feeding Practices Increases the Risk for Respiratory Infections in Surinamese Preschool Children. Int J Environ Res Public Health. 2021 Oct 13;18(20):10739. doi: 10.3390/ijerph182010739. PMID: 34682480; PMCID: PMC8536009.

598: Kesztyüs D, Vorwieger E, Schönsteiner D, Gulich M, Kesztyüs T. Applicability of time-restricted eating for the prevention of lifestyle-dependent diseases in a working population: results of a pilot study in a pre-post design. Ger Med Sci. 2021 Mar 29;19:Doc04. doi: 10.3205/000291. PMID: 33911996; PMCID: PMC8051591.

599: Sithey G, Wen LM, Dzed L, Li M. Noncommunicable diseases risk factors in Bhutan: A secondary analysis of data from Bhutan's nationwide STEPS survey 2014. PLoS One. 2021 Sep 23;16(9):e0257385. doi: 10.1371/journal.pone.0257385. PMID: 34555064; PMCID: PMC8459987.

600: Kozioł-Kozakowska A, Salamon D, Grzenda-Adamek Z, Krawczyk A, Duplaga M, Gosiewski T, Kowalska-Duplaga K. Changes in Diet and Anthropometric Parameters in Children and Adolescents with Celiac Disease-One Year of Follow-Up. Nutrients. 2021 Nov 28;13(12):4306. doi: 10.3390/nu13124306. PMID: 34959858; PMCID: PMC8703461.

601: Barros NERP, Moreno LA, Arruda SPM, de Assis RC, Celedonio RF, Silva FRA, Pinto FJM, Maia CSC. Association between Eating Patterns and Excess Body Weight in Adolescents. Child Obes. 2021 Sep;17(6):400-407. doi: 10.1089/chi.2020.0265. Epub 2021 Apr 26. PMID: 33902325.

602: Clark JMR, Twamley EW, Krause JS. Mental health, pain, and sleep factors

associated with subjective cognitive difficulties in individuals with spinal cord injury. Rehabil Psychol. 2021 Aug;66(3):248-256. doi: 10.1037/rep0000384. PMID: 34472923.

603: Ghazaryan A, Carlson A, Rhone AY, Roy K. Association between the Nutritional Quality of Household At-Home Food Purchases and Chronic Diseases and Risk Factors in the United States, 2015. Nutrients. 2021 Sep 18;13(9):3260. doi: 10.3390/nu13093260. PMID: 34579136; PMCID: PMC8468462.

604: Timalsina P, Singh R. Assessment of Risk Factors of Noncommunicable Diseases among Semiurban Population of Kavre District, Nepal. J Environ Public Health. 2021 Jun 7;2021:5584561. doi: 10.1155/2021/5584561. PMID: 34211559; PMCID: PMC8205567.

605: Simunovic M, Supe-Domic D, Karin Z, Degoricija M, Paradzik M, Skrabic R, Jukic A, Bozic J, Skrabic V. The Relationship of Vitamin D Status, Adherence to the Mediterranean Diet, and Physical Activity in Obese Children and Adolescents. J Med Food. 2021 Apr;24(4):385-393. doi: 10.1089/jmf.2020.0032. Epub 2020 Aug 12. PMID: 32783677.

606: Barone Gibbs B, Kline CE, Huber KA, Paley JL, Perera S. Covid-19 shelter-at-home and work, lifestyle and well-being in desk workers. Occup Med (Lond). 2021 Apr 9;71(2):86-94. doi: 10.1093/occmed/kqab011. PMID: 33598681; PMCID: PMC7928687.

607: Gande N, Pechlaner R, Bernar B, Staudt A, Stock K, Hochmayr C, Geiger R, Kiechl-Kohlendorfer U, Knoflach M; Early Vascular Aging (EVA) Study Group. Cardiovascular health behaviors and associations of sex, age, and education in adolescents - Results from the EVA Tyrol study. Nutr Metab Cardiovasc Dis. 2021 Apr 9;31(4):1286-1292. doi: 10.1016/j.numecd.2020.11.002. Epub 2020 Nov 10. PMID: 33558093.

608: Emara MH, Soliman HH, Elnadry M, Mohamed Said E, Abd-Elsalam S, Elbatae HE, Zaher TI, Ezzeldin S Bazeed S, Abdel-Razik A, Youssef Mohamed S, Elfert A; "Egyptian Ramadan Fasting, Liver Diseases Interest Group". Ramadan fasting and liver diseases: A review with practice advices and recommendations. Liver Int. 2021 Mar;41(3):436-448. doi: 10.1111/liv.14775. Epub 2021 Jan 9. PMID: 33369880.

609: Noerman S, Kokla M, Koistinen VM, Lehtonen M, Tuomainen TP, Brunius C, Virtanen JK, Hanhineva K. Associations of the serum metabolite profile with a healthy Nordic diet and risk of coronary artery disease. Clin Nutr. 2021 May;40(5):3250-3262. doi: 10.1016/j.clnu.2020.10.051. Epub 2020 Oct 31. PMID: 33190988.

610: Gallardo-Alfaro L, Bibiloni MDM, Bouzas C, Mascaró CM, Martínez-González MÁ, Salas-Salvadó J, Corella D, Schröder H, Martínez JA, Alonso-Gómez ÁM, Wärnberg J, Vioque J, Romaguera D, Lopez-Miranda J, Estruch R, Tinahones FJ, Lapetra J, Serra-Majem L, Bueno-Cavanillas A, Micó RM, Pintó X, Gaforio JJ, Ortíz-Ramos M, Altés-Boronat A, Luca BL, Daimiel L, Ros E, Sayon-Orea C,

Becerra-Tomás N, Gimenez-Alba IM, Castañer O, Abete I, Tojal-Sierra L, Pérez-López J, Torres-Collado L, Colom A, Garcia-Rios A, Castro-Barquero S, Bernal R, Santos-Lozano JM, Fernandez-Lazaro CI, Hernández-Alonso P, Saiz C, Zomeño MD, Zulet MA, Belló-Mora MC, Basterra-Gortari FJ, Canudas S, Goday A, Tur JA; PREDIMED-Plus investigators. Physical activity and metabolic syndrome severity among older adults at cardiovascular risk: 1-Year trends. Nutr Metab Cardiovasc Dis. 2021 Sep 22;31(10):2870-2886. doi: 10.1016/j.numecd.2021.06.015. Epub 2021 Jun 30. PMID: 34366176.

611: Glenn AJ, Boucher BA, Kavcic CC, Khan TA, Paquette M, Kendall CWC, Hanley AJ, Jenkins DJA, Sievenpiper JL. Development of a Portfolio Diet Score and Its Concurrent and Predictive Validity Assessed by a Food Frequency Questionnaire. Nutrients. 2021 Aug 19;13(8):2850. doi: 10.3390/nu13082850. PMID: 34445009; PMCID: PMC8398786.

612: Miele MJ, Souza RT, Calderon IM, Feitosa FE, Leite DF, Rocha Filho EA, Vettorazzi J, Mayrink J, Fernandes KG, Vieira MC, Pacagnella RC, Cecatti JG. Maternal Nutrition Status Associated with Pregnancy-Related Adverse Outcomes. Nutrients. 2021 Jul 13;13(7):2398. doi: 10.3390/nu13072398. PMID: 34371906; PMCID: PMC8308922.

613: Pirjani R, Moini A, Heshmati J, Mardi-Mamaghani A, Esmaeili M, Shafaatdoost M, Maleki-Hajiagha A, Karimi E, Hossein-Boroujerdi M, Shokri F, Mosanezhad Z, Bajool N, Noori M, Hosseini L, Persad E, Sepidarkish M. Mothers and their children's health (MATCH): a study protocol for a population-based longitudinal cohort. BMC Pregnancy Childbirth. 2021 Apr 12;21(1):297. doi: 10.1186/s12884-021-03732-6. PMID: 33845792; PMCID: PMC8042918.

614: Nguyen LTK, Do BN, Vu DN, Pham KM, Vu MT, Nguyen HC, Tran TV, Le HP, Nguyen TTP, Nguyen QM, Tran CQ, Nguyen KT, Yang SH, Chao JC, Van Duong T. Physical Activity and Diet Quality Modify the Association between Comorbidity and Disability among Stroke Patients. Nutrients. 2021 May 13;13(5):1641. doi: 10.3390/nu13051641. PMID: 34068135; PMCID: PMC8152968.

615: Wang H, Fan J, Yu C, Guo Y, Pei P, Yang L, Chen Y, Du H, Meng F, Chen J, Chen Z, Lv J, Li L, On Behalf Of The China Kadoorie Biobank Collaborative Group. Consumption of Tea, Alcohol, and Fruits and Risk of Kidney Stones: A Prospective Cohort Study in 0.5 Million Chinese Adults. Nutrients. 2021 Mar 29;13(4):1119. doi: 10.3390/nu13041119. PMID: 33805392; PMCID: PMC8065818.

616: Fernández AI, Bermejo J, Yotti R, Martínez-Gonzalez MÁ, Mira A, Gophna U, Karlsson R, Al-Daccak R, Martín-Demiguel I, Gutiérrez-Ibanes E, Charron D, Fernández-Avilés F; MEDIMACS research team. The impact of Mediterranean diet on coronary plaque vulnerability, microvascular function, inflammation and microbiome after an acute coronary syndrome: study protocol for the MEDIMACS randomized, controlled, mechanistic clinical trial. Trials. 2021 Nov 12;22(1):795. doi: 10.1186/s13063-021-05746-z. PMID: 34772433; PMCID: PMC8588729.

617: Boniecka I, Jeznach-Steinhagen A, Michalska W, Rymarz A, Szostak-Węgierek D, Niemczyk S. Nutritional Status, Selected Nutrients Intake and Their Relationship with the Concentration of Ghrelin and Adiponectin in Patients with Diabetic Nephropathy. Nutrients. 2021 Dec 10;13(12):4416. doi: 10.3390/nu13124416. PMID: 34959967; PMCID: PMC8707934.

618: Miszta A, Huskens D, Donkervoort D, Roberts MJM, Wolberg AS, de Laat B. Assessing Plasmin Generation in Health and Disease. Int J Mol Sci. 2021 Mar 9;22(5):2758. doi: 10.3390/ijms22052758. PMID: 33803235; PMCID: PMC7963172.

619: Guevara M, Salamanca-Fernández E, Miqueleiz E, Gavrila D, Amiano P, Bonet C, Rodríguez-Barranco M, Huerta JM, Bujanda L, Sánchez MJ, Chirlaque MD, Agudo A, Ardanaz E, Castilla J. Inflammatory Potential of the Diet and Incidence of Crohn's Disease and Ulcerative Colitis in the EPIC-Spain Cohort. Nutrients. 2021 Jun 26;13(7):2201. doi: 10.3390/nu13072201. PMID: 34206846; PMCID: PMC8308349.

620: Choi Y, Steffen LM, Chu H, Duprez DA, Gallaher DD, Shikany JM, Schreiner PJ, Shroff GR, Jacobs DR. A Plant-Centered Diet and Markers of Early Chronic Kidney Disease during Young to Middle Adulthood: Findings from the Coronary Artery Risk Development in Young Adults (CARDIA) Cohort. J Nutr. 2021 Sep 4;151(9):2721-2730. doi: 10.1093/jn/nxab155. PMID: 34087933; PMCID: PMC8417917.

621: Kambale RM, Ngaboyeka GA, Kasengi JB, Niyitegeka S, Cinkenye BR, Baruti A, Mutuga KC, Van der Linden D. Minimum acceptable diet among children aged 6-23 months in South Kivu, Democratic Republic of Congo: a community-based cross-sectional study. BMC Pediatr. 2021 May 19;21(1):239. doi: 10.1186/s12887-021-02713-0. PMID: 34011304; PMCID: PMC8132412.

622: Ghaemi F, Firouzabadi FD, Moosaie F, Shadnoush M, Poopak A, Kermanchi J, Abhari SMF, Forouzanfar R, Mansournia MA, Khosravi A, Mohajer B, Ramandi MMA, Nakhjavani M, Esteghamati A. Effects of a Mediterranean diet on the development of diabetic complications: A longitudinal study from the nationwide diabetes report of the National Program for Prevention and Control of Diabetes (NPPCD 2016-2020). Maturitas. 2021 Nov;153:61-67. doi: 10.1016/j.maturitas.2021.08.003. Epub 2021 Aug 28. PMID: 34654529.

623: Weiman DI, Mahmud FH, Clarke ABM, Assor E, McDonald C, Saibil F, Lochnan HA, Punthakee Z, Marcon MA; CD-DIET Study Group. Impact of a Gluten-Free Diet on Quality of Life and Health Perception in Patients With Type 1 Diabetes and Asymptomatic Celiac Disease. J Clin Endocrinol Metab. 2021 Apr 23;106(5):e1984-e1992. doi: 10.1210/clinem/dgaa977. PMID: 33524131.

624: Mattila M, Hakola L, Niinistö S, Tapanainen H, Takkinen HM, Ahonen S, Ilonen J, Toppari J, Veijola R, Knip M, Virtanen SM. Maternal Vitamin C and Iron Intake during Pregnancy and the Risk of Islet Autoimmunity and Type 1 Diabetes in Children: A Birth Cohort Study. Nutrients. 2021 Mar 13;13(3):928. doi: 10.3390/nu13030928. PMID: 33805588; PMCID: PMC8001228.

625: Wang L, Du J, Cao W, Sun S. Trends of stroke attributable to high sodium

intake at the global, regional, and national levels from 1990 to 2019: a population-based study. Neurol Res. 2021 Jun;43(6):474-481. doi: 10.1080/01616412.2020.1867950. Epub 2020 Dec 30. PMID: 33377423.

626: Hershey MS, Sotos-Prieto M, Ruiz-Canela M, Christophi CA, Moffatt S, Martínez-González MÁ, Kales SN. The Mediterranean lifestyle (MEDLIFE) index and metabolic syndrome in a non-Mediterranean working population. Clin Nutr. 2021 May;40(5):2494-2503. doi: 10.1016/j.clnu.2021.03.026. Epub 2021 Mar 31. PMID: 33932793.

627: Grammatikopoulou MG, Gkiouras K, Polychronidou G, Kaparounaki C, Gkouskou KK, Magkos F, Donini LM, Eliopoulos AG, Goulis DG. Obsessed with Healthy Eating: A Systematic Review of Observational Studies Assessing Orthorexia Nervosa in Patients with Diabetes Mellitus. Nutrients. 2021 Oct 27;13(11):3823. doi: 10.3390/nu13113823. PMID: 34836080; PMCID: PMC8622186.

628: Verbeek J, Hoving J, Boschman J, Chong LY, Livingstone-Banks J, Bero L. Systematic Reviews Should Consider Effects From Both the Population and the Individual Perspective. Am J Public Health. 2021 May;111(5):820-825. doi: 10.2105/AJPH.2020.306147. PMID: 33826374; PMCID: PMC8034000.

629: Li H, Wang X, Ye M, Zhang S, Zhang Q, Meng G, Liu L, Wu H, Gu Y, Wang Y, Zhang T, Sun S, Wang X, Zhou M, Jia Q, Song K, Wang Y, Niu K. Does a high intake of green leafy vegetables protect from NAFLD? Evidence from a large population study. Nutr Metab Cardiovasc Dis. 2021 Jun 7;31(6):1691-1701. doi: 10.1016/j.numecd.2021.01.009. Epub 2021 Feb 2. PMID: 33838994.

630: San Onofre Bernat N, Quiles I Izquierdo J, Trescastro-López EM. Estilos de vida y factores sociodemográficos asociados a la alta adhesión a la dieta mediterránea en la población adulta de la Comunidat Valenciana (España) [Lifestyles and sociodemographic factors associated with high adherence to the Mediterranean diet in the adult population of the Comunitat Valenciana (Spain)]. Nutr Hosp. 2021 Apr 19;38(2):337-348. Spanish. doi: 10.20960/nh.03387. PMID: 33611915.

631: Hanley-Cook GT, Huybrechts I, Biessy C, Remans R, Kennedy G, Deschasaux-Tanguy M, Murray KA, Touvier M, Skeie G, Kesse-Guyot E, Argaw A, Casagrande C, Nicolas G, Vineis P, Millett CJ, Weiderpass E, Ferrari P, Dahm CC, Bueno-de-Mesquita HB, Sandanger TM, Ibsen DB, Freisling H, Ramne S, Jannasch F, van der Schouw YT, Schulze MB, Tsilidis KK, Tjønneland A, Ardanaz E, Bodén S, Cirera L, Gargano G, Halkjær J, Jakszyn P, Johansson I, Katzke V, Masala G, Panico S, Rodriguez-Barranco M, Sacerdote C, Srour B, Tumino R, Riboli E, Gunter MJ, Jones AD, Lachat C. Food biodiversity and total and cause-specific mortality in 9 European countries: An analysis of a prospective cohort study. PLoS Med. 2021 Oct 18;18(10):e1003834. doi: 10.1371/journal.pmed.1003834. PMID: 34662340; PMCID: PMC8559947.

632: Sun L, Zhao W, Li J, Tse LA, Xing X, Lin S, Zhao J, Ren Z, Zhang CX, Liu X. Dietary flavonoid intake and risk of esophageal squamous cell carcinoma: A

population-based case-control study. Nutrition. 2021 Sep;89:111235. doi: 10.1016/j.nut.2021.111235. Epub 2021 Mar 7. PMID: 33878555.

633: Delshad Aghdam S, Siassi F, Nasli Esfahani E, Qorbani M, Rajab A, Sajjadpour Z, Bashiri A, Aghayan M, Sotoudeh G. Dietary phytochemical index associated with cardiovascular risk factor in patients with type 1 diabetes mellitus. BMC Cardiovasc Disord. 2021 Jun 12;21(1):293. doi: 10.1186/s12872-021-02106-2. PMID: 34118879; PMCID: PMC8199677.

634: Kouvari M, Boutari C, Chrysohoou C, Fragkopoulou E, Antonopoulou S, Tousoulis D, Pitsavos C, Panagiotakos DB, Mantzoros CS; ATTICA study Investigators. Mediterranean diet is inversely associated with steatosis and fibrosis and decreases ten-year diabetes and cardiovascular risk in NAFLD subjects: Results from the ATTICA prospective cohort study. Clin Nutr. 2021 May;40(5):3314-3324. doi: 10.1016/j.clnu.2020.10.058. Epub 2020 Nov 7. PMID: 33234342.

635: Cattaneo L, Lopreiato V, Piccioli-Cappelli F, Trevisi E, Minuti A. Plasma albumin-to-globulin ratio before dry-off as a possible index of inflammatory status and performance in the subsequent lactation in dairy cows. J Dairy Sci. 2021 Jul;104(7):8228-8242. doi: 10.3168/jds.2020-19944. Epub 2021 Apr 15. PMID: 33865585.

636: Zhang T, Rayamajhi S, Meng G, Zhang Q, Liu L, Wu H, Gu Y, Wang Y, Zhang S, Wang X, Zhang J, Li H, Thapa A, Sun S, Wang X, Zhou M, Jia Q, Song K, Niu K. Edible mushroom consumption and incident hyperuricemia: results from the TCLSIH cohort study. Food Funct. 2021 Oct 4;12(19):9178-9187. doi: 10.1039/d1fo00650a. PMID: 34606546.

637: Hu J, Aris IM, Lin PD, Wan N, Liu Y, Wang Y, Wen D. Association of Maternal Dietary Patterns during Pregnancy and Offspring Weight Status across Infancy: Results from a Prospective Birth Cohort in China. Nutrients. 2021 Jun 15;13(6):2040. doi: 10.3390/nu13062040. PMID: 34203618; PMCID: PMC8232115.

638: Donat-Vargas C, Sandoval-Insausti H, Rey-García J, Moreno-Franco B, Åkesson A, Banegas JR, Rodríguez-Artalejo F, Guallar-Castillón P. High Consumption of Ultra-Processed Food is Associated with Incident Dyslipidemia: A Prospective Study of Older Adults. J Nutr. 2021 Aug 7;151(8):2390-2398. doi: 10.1093/jn/nxab118. PMID: 34038538.

639: Glenn AJ, Hernández-Alonso P, Kendall CWC, Martínez-González MÁ, Corella D, Fitó M, Martínez JA, Alonso-Gómez ÁM, Wärnberg J, Vioque J, Romaguera D, López-Miranda J, Estruch R, Tinahones FJ, Lapetra J, Serra-Majem JL, Bueno-Cavanillas A, Tur JA, Celada SR, Pintó X, Delgado-Rodríguez M, Matía-Martín P, Vidal J, Mas-Fontao S, Daimiel L, Ros E, Jenkins DJA, Toledo E, Sorlí JV, Castañer O, Abete I, Rodriguez AM, Barceló OF, Oncina-Canovas A, Konieczna J, Garcia-Rios A, Casas R, Gómez-Pérez AM, Santos-Lozano JM, Vazquez-Ruiz Z, Portolés O, Schröder H, Zulet MA, Eguaras S, Lete IS, Zomeño MD, Sievenpiper JL, Salas-Salvadó J. Longitudinal changes in adherence to the portfolio and DASH dietary patterns and

cardiometabolic risk factors in the PREDIMED-Plus study. Clin Nutr. 2021 May;40(5):2825-2836. doi: 10.1016/j.clnu.2021.03.016. Epub 2021 Mar 18. PMID: 33933749.

640: Nguyen LH, Cao Y, Hur J, Mehta RS, Sikavi DR, Wang Y, Ma W, Wu K, Song M, Giovannucci EL, Rimm EB, Willett WC, Garrett WS, Izard J, Huttenhower C, Chan AT. The Sulfur Microbial Diet Is Associated With Increased Risk of Early-Onset Colorectal Cancer Precursors. Gastroenterology. 2021 Nov;161(5):1423-1432.e4. doi: 10.1053/j.gastro.2021.07.008. Epub 2021 Jul 14. PMID: 34273347; PMCID: PMC8545755.

641: Rajabi H, Sabouri M, Hatami E. Associations between physical activity levels with nutritional status, physical fitness and biochemical indicators in older adults. Clin Nutr ESPEN. 2021 Oct;45:389-398. doi: 10.1016/j.clnesp.2021.07.014. Epub 2021 Jul 24. PMID: 34620345.

642: Clinton-McHarg T, Delaney T, Lamont H, Lecathelinais C, Yoong SL, Wolfenden L, Sutherland R, Wyse R. A Cross-Sectional Study of the Nutritional Quality of New South Wales High School Student Food and Drink Purchases Made via an Online Canteen Ordering System. Nutrients. 2021 Nov 30;13(12):4327. doi: 10.3390/nu13124327. PMID: 34959881; PMCID: PMC8706117.

643: Strong H, Harry O, Westcott E, Kidwell KM, Couch SC, Peairs A, Britto MT, Crosby LE. Weight status and health behaviors of adolescents and young adults with sickle cell disease: The emerging risk for obesity. Pediatr Hematol Oncol. 2021 Apr;38(3):265-271. doi: 10.1080/08880018.2020.1838010. Epub 2020 Nov 5. PMID: 33150822.

644: Picolo VL, Quadros VA, Canzian J, Grisolia CK, Goulart JT, Pantoja C, de Bem AF, Rosemberg DB. Short-term high-fat diet induces cognitive decline, aggression, and anxiety-like behavior in adult zebrafish. Prog Neuropsychopharmacol Biol Psychiatry. 2021 Aug 30;110:110288. doi: 10.1016/j.pnpbp.2021.110288. Epub 2021 Feb 21. PMID: 33626334.

645: Li N, Wu X, Zhuang W, Xia L, Chen Y, Wang Y, Wu C, Rao Z, Du L, Zhao R, Yi M, Wan Q, Zhou Y. Green leafy vegetable and lutein intake and multiple health outcomes. Food Chem. 2021 Oct 30;360:130145. doi: 10.1016/j.foodchem.2021.130145. Epub 2021 May 18. PMID: 34034049.

646: Chuang SY, Chang HY, Fang HL, Lee SC, Hsu YY, Yeh WT, Liu WL, Pan WH. The Healthy Taiwanese Eating Approach is inversely associated with all-cause and cause-specific mortality: A prospective study on the Nutrition and Health Survey in Taiwan, 1993-1996. PLoS One. 2021 May 6;16(5):e0251189. doi: 10.1371/journal.pone.0251189. PMID: 33956833; PMCID: PMC8101962.

647: Wang R, Wang W, Hu P, Zhang R, Dong X, Zhang D. Association of Dietary Vitamin D Intake, Serum 25(OH)D₃, 25(OH)D₂ with Cognitive Performance in the Elderly. Nutrients. 2021 Sep 2;13(9):3089. doi: 10.3390/nu13093089. PMID: 34578965; PMCID: PMC8467888.

648: Jia P, Liu L, Xie X, Yuan C, Chen H, Guo B, Zhou J, Yang S. Changes in dietary patterns among youths in China during COVID-19 epidemic: The COVID-19 impact on lifestyle change survey (COINLICS). Appetite. 2021 Mar 1;158:105015. doi: 10.1016/j.appet.2020.105015. Epub 2020 Oct 27. PMID: 33121998.

649: Habte A, Dessu S, Haile D. Determinants of practice of preconception care among women of reproductive age group in southern Ethiopia, 2020: content analysis. Reprod Health. 2021 May 21;18(1):100. doi: 10.1186/s12978-021-01154-3. PMID: 34020669; PMCID: PMC8139064.

650: Zhang T, Gan S, Ye M, Meng G, Zhang Q, Liu L, Wu H, Gu Y, Zhang S, Wang Y, Wang X, Sun S, Wang X, Zhou M, Jiao H, Jia Q, Song K, Wu Y, Niu K. Association between consumption of ultra-processed foods and hyperuricemia: TCLSIH prospective cohort study. Nutr Metab Cardiovasc Dis. 2021 Jun 30;31(7):1993-2003. doi: 10.1016/j.numecd.2021.04.001. Epub 2021 May 7. PMID: 34119375.

651: Peñalvo JL, Sagastume D, Mertens E, Uzhova I, Smith J, Wu JHY, Bishop E, Onopa J, Shi P, Micha R, Mozaffarian D. Effectiveness of workplace wellness programmes for dietary habits, overweight, and cardiometabolic health: a systematic review and meta-analysis. Lancet Public Health. 2021 Sep;6(9):e648-e660. doi: 10.1016/S2468-2667(21)00140-7. PMID: 34454642; PMCID: PMC8627548.

652: Kurmann S, Reber E, Vasiloglou MF, Schuetz P, Schoenenberger AW, Uhlmann K, Sterchi AB, Stanga Z. Energy and protein intake in medical and geriatric inpatients with MEDPass versus conventional administration of oral nutritional supplements: study protocol for the randomized controlled MEDPass Trial. Trials. 2021 Mar 16;22(1):210. doi: 10.1186/s13063-021-05145-4. PMID: 33726841; PMCID: PMC7962290.

653: Bruce JM, Cozart JS, Shook RP, Ruppen S, Siengsukon C, Simon S, Befort C, Lynch S, Mahmoud R, Drees B, Norouzinia AN, Bradish T, Posson P, Hibbing PR, Bruce AS. Modifying Diet and Exercise in MS (MoDEMS): Study design and protocol for a telehealth weight loss intervention for adults with obesity & Multiple Sclerosis. Contemp Clin Trials. 2021 Aug;107:106495. doi: 10.1016/j.cct.2021.106495. Epub 2021 Jul 1. PMID: 34216814.

654: Gao Q, Eshak ES, Muraki I, Shirai K, Yamagishi K, Tamakoshi A, Iso H. The apparent inverse association between dietary carotene intake and risk of cardiovascular mortality disappeared after adjustment for other cardioprotective dietary intakes: The Japan collaborative cohort study. Nutr Metab Cardiovasc Dis. 2021 Oct 28;31(11):3064-3075. doi: 10.1016/j.numecd.2021.07.026. Epub 2021 Jul 30. PMID: 34629253.

655: Khani Jeihooni A, Jormand H, Saadat N, Hatami M, Abdul Manaf R, Afzali Harsini P. The application of the theory of planned behavior to nutritional behaviors related to cardiovascular disease among the women. BMC Cardiovasc

Disord. 2021 Dec 7;21(1):589. doi: 10.1186/s12872-021-02399-3. PMID: 34876014; PMCID: PMC8650365.

656: Wang C, Sun Y, Jiang D, Wang C, Liu S. Risk-Attributable Burden of Ischemic Heart Disease in 137 Low- and Middle-Income Countries From 2000 to 2019. J Am Heart Assoc. 2021 Oct 5;10(19):e021024. doi: 10.1161/JAHA.121.021024. Epub 2021 Sep 29. PMID: 34585592.

657: Rodrigues C, Pinto A, Faria A, Teixeira D, van Wegberg AMJ, Ahring K, Feillet F, Calhau C, MacDonald A, Moreira-Rosário A, Rocha JC. Is the Phenylalanine-Restricted Diet a Risk Factor for Overweight or Obesity in Patients with Phenylketonuria (PKU)? A Systematic Review and Meta-Analysis. Nutrients. 2021 Sep 28;13(10):3443. doi: 10.3390/nu13103443. PMID: 34684443; PMCID: PMC8538431.

658: Amick KA, Mahapatra G, Bergstrom J, Gao Z, Craft S, Register TC, Shively CA, Molina AJA. Brain region-specific disruption of mitochondrial bioenergetics in cynomolgus macaques fed a Western versus a Mediterranean diet. Am J Physiol Endocrinol Metab. 2021 Nov 1;321(5):E652-E664. doi: 10.1152/ajpendo.00165.2021. Epub 2021 Sep 27. PMID: 34569271; PMCID: PMC8791787.

659: Rombouts C, Van Meulebroek L, De Spiegeleer M, Goethals S, Van Hecke T, De Smet S, De Vos WH, Vanhaecke L. Untargeted Metabolomics Reveals Elevated L-Carnitine Metabolism in Pig and Rat Colon Tissue Following Red Versus White Meat Intake. Mol Nutr Food Res. 2021 Apr;65(7):e2000463. doi: 10.1002/mnfr.202000463. Epub 2021 Mar 1. PMID: 33550692.

660: Fismen AS, Buoncristiano M, Williams J, Helleve A, Abdrakhmanova S, Bakacs M, Bergh IH, Boymatova K, Duleva V, Fijałkowska A, García-Solano M, Gualtieri A, Gutiérrez-González E, Hejgaard T, Huidumac-Petrescu C, Hyska J, Kelleher CC, Kierkegaard L, Kujundžić E, Kunešová M, Milanović SM, Nardone P, Nurk E, Ostojic SM, Ozcebe LH, Peterkova V, Petrauskiene A, Pudule I, Rakhmatulleoeva S, Rakovac I, Rito AI, Rutter H, Sacchini E, Stojisavljević D, Farrugia Sant'Angelo V, Shengelia L, Spinelli A, Spiroski I, Tanrygulyyeva M, Usupova Z, Weghuber D, Breda J. Socioeconomic differences in food habits among 6- to 9-year-old children from 23 countries-WHO European Childhood Obesity Surveillance Initiative (COSI 2015/2017). Obes Rev. 2021 Nov;22 Suppl 6:e13211. doi: 10.1111/obr.13211. Epub 2021 Jul 7. PMID: 34235830.

661: Rhee DK, Ji Y, Hong X, Pearson C, Wang X, Caulfield LE. Mediterranean-Style Diet and Birth Outcomes in an Urban, Multiethnic, and Low-Income US Population. Nutrients. 2021 Apr 3;13(4):1188. doi: 10.3390/nu13041188. PMID: 33916686; PMCID: PMC8066173.

662: Alzahrani AH, Skytte MJ, Samkani A, Thomsen MN, Astrup A, Ritz C, Frystyk J, Holst JJ, Madsbad S, Haugaard SB, Krarup T, Larsen TM, Magkos F. Effects of a Self-Prepared Carbohydrate-Reduced High-Protein Diet on Cardiovascular Disease Risk Markers in Patients with Type 2 Diabetes. Nutrients. 2021 May 17;13(5):1694. doi: 10.3390/nu13051694. PMID: 34067585; PMCID: PMC8157073.

663: Lai X, Li B, Fang Y, Wang J, Li Y, Liu J, Zhang Z, An S. Association of dietary isoflavone consumption with subclinical cardiovascular disease in middle-aged and elderly Chinese people. Nutr Metab Cardiovasc Dis. 2021 Jul 22;31(8):2302-2310. doi: 10.1016/j.numecd.2021.04.003. Epub 2021 Apr 18. PMID: 34154891.

664: Muñoz-Cabrejas A, Laclaustra M, Guallar-Castillón P, Casasnovas JA, Jarauta E, Sandoval-Insausti H, Donat-Vargas C, Moreno-Franco B. High-quality intake of carbohydrates is associated with lower prevalence of subclinical atherosclerosis in femoral arteries: The AWHS study. Clin Nutr. 2021 Jun;40(6):3883-3889. doi: 10.1016/j.clnu.2021.04.049. Epub 2021 May 14. PMID: 34134004.

665: Vitosyte M, Puriene A, Stankeviciene I, Rimkevicius A, Trumpaite-Vanagiene R, Aleksejuniene J, Stangvaltaite-Mouhat L. Oral Health among Adult Residents in Vilnius, Lithuania. Int J Environ Res Public Health. 2022 Jan 5;19(1):582. doi: 10.3390/ijerph19010582. PMID: 35010841; PMCID: PMC8745011.

666: Fang H, He X, Wu Y, Chen S, Zhang M, Pan F, Huang J, Liu A. Association Between Selenium Level in Blood and Glycolipid Metabolism in Residents of Enshi Prefecture, China. Biol Trace Elem Res. 2021 Jul;199(7):2456-2466. doi: 10.1007/s12011-020-02372-9. Epub 2020 Oct 6. PMID: 33025519.

667: Camacho-Barcia L, Munguía L, Lucas I, de la Torre R, Salas-Salvadó J, Pintó X, Corella D, Granero R, Jiménez-Murcia S, González-Monje I, Esteve-Luque V, Cuenca-Royo A, Gómez-Martínez C, Paz-Graniel I, Forcano L, Fernández-Aranda F. Metabolic, Affective and Neurocognitive Characterization of Metabolic Syndrome Patients with and without Food Addiction. Implications for Weight Progression. Nutrients. 2021 Aug 13;13(8):2779. doi: 10.3390/nu13082779. PMID: 34444940; PMCID: PMC8398101.

668: Azhar G, Wei JY, Schutzler SE, Coker K, Gibson RV, Kirby MF, Ferrando AA, Wolfe RR. Daily Consumption of a Specially Formulated Essential Amino Acid-Based Dietary Supplement Improves Physical Performance in Older Adults With Low Physical Functioning. J Gerontol A Biol Sci Med Sci. 2021 Jun 14;76(7):1184-1191. doi: 10.1093/gerona/glab019. PMID: 33475727; PMCID: PMC8202157.

669: Hand S, Dunstan F, Jones K, Doull I. The effect of diet in infancy on asthma in young adults: the Merthyr Allergy Prevention Study. Thorax. 2021 Nov;76(11):1072-1077. doi: 10.1136/thoraxjnl-2020-215040. Epub 2021 May 7. PMID: 33963089.

670: Ding EY, Mehawej J, Abu H, Lessard D, Saczynski JS, McManus DD, Kiefe CI, Goldberg RJ. Cardiovascular Health Metrics in Patients Hospitalized with an Acute Coronary Syndrome. Am J Med. 2021 Nov;134(11):1396-1402.e1. doi: 10.1016/j.amjmed.2021.06.016. Epub 2021 Jul 14. PMID: 34273284; PMCID: PMC8605989.

671: Capozzi F, Magkos F, Fava F, Milani GP, Agostoni C, Astrup A, Saguy IS. A Multidisciplinary Perspective of Ultra-Processed Foods and Associated Food Processing Technologies: A View of the Sustainable Road Ahead. Nutrients. 2021 Nov 5;13(11):3948. doi: 10.3390/nu13113948. PMID: 34836203; PMCID: PMC8619086.

672: Bzikowska-Jura A, Machaj N, Sobieraj P, Barbarska O, Olędzka G, Wesolowska A. Do Maternal Factors and Milk Expression Patterns Affect the Composition of Donor Human Milk? Nutrients. 2021 Jul 15;13(7):2425. doi: 10.3390/nu13072425. PMID: 34371935; PMCID: PMC8308884.

673: Zhang X, Gong Y, Della Corte K, Yu D, Xue H, Shan S, Tian G, Liang Y, Zhang J, He F, Yang D, Zhou R, Bao W, Buyken AE, Cheng G. Relevance of dietary glycemic index, glycemic load and fiber intake before and during pregnancy for the risk of gestational diabetes mellitus and maternal glucose homeostasis. Clin Nutr. 2021 May;40(5):2791-2799. doi: 10.1016/j.clnu.2021.03.041. Epub 2021 Apr 5. PMID: 33933745.

674: Madjd A, Taylor MA, Delavari A, Malekzadeh R, Macdonald IA, Farshchi HR. Effects of consuming later evening meal <i>v</i>. earlier evening meal on weight loss during a weight loss diet: a randomised clinical trial. Br J Nutr. 2021 Aug 28;126(4):632-640. doi: 10.1017/S0007114520004456. Epub 2020 Nov 11. PMID: 33172509.

675: Santaliestra-Pasías AM, Moreno LA, Gracia-Marco L, Buck C, Ahrens W, De Henauw S, Hebestreit A, Kourides Y, Lauria F, Lissner L, Molnar D, Veidebaum T, González-Gil EM; on behalf the IDEFICS consortium. Prospective physical fitness status and development of cardiometabolic risk in children according to body fat and lifestyle behaviours: The IDEFICS study. Pediatr Obes. 2021 Nov;16(11):e12819. doi: 10.1111/ijpo.12819. Epub 2021 May 18. PMID: 34002531.

676: Ali A, Sohaib M, Iqbal S, Hayat K, Khan AU, Rasool MF. Evaluation of COVID-19 Disease Awareness and Its Relation to Mental Health, Dietary Habits, and Physical Activity: A Cross-Sectional Study from Pakistan. Am J Trop Med Hyg. 2021 Mar 9;104(5):1687-1693. doi: 10.4269/ajtmh.20-1451. PMID: 33690156; PMCID: PMC8103453.

677: Oncina-Cánovas A, Vioque J, González-Palacios S, Martínez-González MÁ, Salas-Salvadó J, Corella D, Zomeño D, Martínez JA, Alonso-Gómez ÁM, Wärnberg J, Romaguera D, López-Miranda J, Estruch R, Bernal-Lopez RM, Lapetra J, Serra-Majem JL, Bueno-Cavanillas A, Tur JA, Martín-Sánchez V, Pintó X, Delgado-Rodríguez M, Matía-Martín P, Vidal J, Vázquez C, Daimiel L, Ros E, Toledo E, Babio N, Sorli JV, Schröder H, Zulet MA, Sorto-Sánchez C, Barón-López FJ, Compañ-Gabucio L, Morey M, García-Ríos A, Casas R, Gómez-Pérez AM, Santos-Lozano JM, Vázquez-Ruiz Z, Nishi SK, Asensio EM, Soldevila N, Abete I, Goicolea-Güemez L, Buil-Cosiales P, García-Gavilán JF, Canals E, Torres-Collado L, García-de-la-Hera M. Pro-vegetarian food patterns and cardiometabolic risk in the PREDIMED-Plus study: a cross-sectional baseline analysis. Eur J Nutr. 2022 Feb;61(1):357-372. doi: 10.1007/s00394-021-02647-4. Epub 2021 Aug 9. PMID: 34368892; PMCID: PMC8783853.

678: Medina-Vadora MM, Severi C, Lecot C, Ruiz-Lopez MD, Gil A. Study of Food Intake and Physical Activity Patterns in the Working Population of the Uruguayan State Electrical Company (UTE): Design, Protocol and Methodology. Nutrients. 2021 Oct 9;13(10):3545. doi: 10.3390/nu13103545. PMID: 34684546; PMCID: PMC8540219.

679: Riznik P, De Leo L, Dolinsek J, Gyimesi J, Klemenak M, Koletzko B, Koletzko S, Koltai T, Korponay-Szabó IR, Krencnik T, Milinovic M, Not T, Palcevski G, Sblattero D, Werkstetter KJ, Dolinsek J. The Knowledge About Celiac Disease Among Healthcare Professionals and Patients in Central Europe. J Pediatr Gastroenterol Nutr. 2021 Apr 1;72(4):552-557. doi: 10.1097/MPG.0000000000003019. PMID: 33346575.

680: Lopes Cortes M, Andrade Louzado J, Galvão Oliveira M, Moraes Bezerra V, Mistro S, Souto Medeiros D, Arruda Soares D, Oliveira Silva K, Nicolaevna Kochergin C, Honorato Dos Santos de Carvalho VC, Wildes Amorim W, Serrate Mengue S. Unhealthy Food and Psychological Stress: The Association between Ultra-Processed Food Consumption and Perceived Stress in Working-Class Young Adults. Int J Environ Res Public Health. 2021 Apr 7;18(8):3863. doi: 10.3390/ijerph18083863. PMID: 33917015; PMCID: PMC8103503.

681: Chiavaroli L, Lee D, Ahmed A, Cheung A, Khan TA, Blanco S, Mejia, Mirrahimi A, Jenkins DJA, Livesey G, Wolever TMS, Rahelić D, Kahleová H, Salas-Salvadó J, Kendall CWC, Sievenpiper JL. Effect of low glycaemic index or load dietary patterns on glycaemic control and cardiometabolic risk factors in diabetes: systematic review and meta-analysis of randomised controlled trials. BMJ. 2021 Aug 4;374:n1651. doi: 10.1136/bmj.n1651. Erratum in: BMJ. 2021 Aug 26;374:n2114. PMID: 34348965; PMCID: PMC8336013.

682: Turesson Wadell A, Bärebring L, Hulander E, Gjertsson I, Hagberg L, Lindqvist HM, Winkvist A. Effects on health-related quality of life in the randomized, controlled crossover trial ADIRA (Anti-inflammatory Diet In Rheumatoid Arthritis). PLoS One. 2021 Oct 14;16(10):e0258716. doi: 10.1371/journal.pone.0258716. PMID: 34648598; PMCID: PMC8516209.

683: Dewey KG, Güngör D, Donovan SM, Madan EM, Venkatramanan S, Davis TA, Kleinman RE, Taveras EM, Bailey RL, Novotny R, Terry N, Butera G, Obbagy J, de Jesus J, Stoody E. Breastfeeding and risk of overweight in childhood and beyond: a systematic review with emphasis on sibling-pair and intervention studies. Am J Clin Nutr. 2021 Nov 8;114(5):1774-1790. doi: 10.1093/ajcn/nqab206. PMID: 34224561.

684: Laurens C, Grundler F, Damiot A, Chery I, Le Maho AL, Zahariev A, Le Maho Y, Bergouignan A, Gauquelin-Koch G, Simon C, Blanc S, Wilhelmi de Toledo F. Is muscle and protein loss relevant in long-term fasting in healthy men? A prospective trial on physiological adaptations. J Cachexia Sarcopenia Muscle. 2021 Dec;12(6):1690-1703. doi: 10.1002/jcsm.12766. Epub 2021 Oct 20. PMID: 34668663; PMCID: PMC8718030.

685: Lee K. Weight underestimation and weight nonregulation behavior may be related to weak grip strength. Nutr Res. 2021 Mar;87:41-48. doi: 10.1016/j.nutres.2020.12.016. Epub 2020 Dec 24. PMID: 33596510.

686: AlZahrani AM, Zawawi MM, Almutairi NA, Alansari AY, Bargawi AA. The impact of Ramadan on visits related to diabetes emergencies at a tertiary care center. BMC Emerg Med. 2021 Dec 23;21(1):162. doi: 10.1186/s12873-021-00555-8. PMID: 34949164; PMCID: PMC8705188.

687: Emamat H, Ghalandari H, Totmaj AS, Tangestani H, Hekmatdoost A. Calcium to magnesium intake ratio and non-alcoholic fatty liver disease development: a case-control study. BMC Endocr Disord. 2021 Mar 18;21(1):51. doi: 10.1186/s12902-021-00721-w. PMID: 33736626; PMCID: PMC7972345.

688: Muniz IAF, Campos DES, Shinkai RSA, Trindade TGD, Cosme-Trindade DC. Case report of oral mucosa garlic burn during COVID-19 pandemic outbreak and role of teledentistry to manage oral health in an older adult woman. Spec Care Dentist. 2021 Sep;41(5):639-643. doi: 10.1111/scd.12605. Epub 2021 May 24. PMID: 34029421; PMCID: PMC8242564.

689: de Hoogh IM, Oosterman JE, Otten W, Krijger AM, Berbée-Zadelaar S, Pasman WJ, van Ommen B, Pijl H, Wopereis S. The Effect of a Lifestyle Intervention on Type 2 Diabetes Pathophysiology and Remission: The Stevenshof Pilot Study. Nutrients. 2021 Jun 25;13(7):2193. doi: 10.3390/nu13072193. PMID: 34202194; PMCID: PMC8308398.

690: McMahon EJ, Campbell KL, Bauer JD, Mudge DW, Kelly JT. Altered dietary salt intake for people with chronic kidney disease. Cochrane Database Syst Rev. 2021 Jun 24;6(6):CD010070. doi: 10.1002/14651858.CD010070.pub3. PMID: 34164803; PMCID: PMC8222708.

691: Gandhi S, Mohanty K, Sahu M, Naik SS, Pahuja E, Gunasekaran DM, Prasad MK. Profile of recipients of holistic health counselling in a psychiatric OPD in South India. Int J Soc Psychiatry. 2021 May;67(3):277-283. doi: 10.1177/0020764020946797. Epub 2020 Aug 2. PMID: 32744115.

692: Chou E, Lindeback R, D'Silva AM, Sampaio H, Neville K, Farrar MA. Growth and nutrition in pediatric neuromuscular disorders. Clin Nutr. 2021 Jun;40(6):4341-4348. doi: 10.1016/j.clnu.2021.01.013. Epub 2021 Jan 22. PMID: 33551221.

693: El-Kour TY, Kelley K, Bruening M, Robson S, Vogelzang J, Yang J, Jimenez EY. Dietetic Workforce Capacity Assessment for Public Health Nutrition and Community Nutrition. J Acad Nutr Diet. 2021 Jul;121(7):1379-1391.e21. doi: 10.1016/j.jand.2020.08.078. Epub 2020 Oct 21. PMID: 34344516; PMCID: PMC7577731.

694: Dong SS, Zhang K, Guo Y, Ding JM, Rong Y, Feng JC, Yao S, Hao RH, Jiang F, Chen JB, Wu H, Chen XF, Yang TL. Phenome-wide investigation of the causal associations between childhood BMI and adult trait outcomes: a two-sample

Mendelian randomization study. Genome Med. 2021 Mar 26;13(1):48. doi: 10.1186/s13073-021-00865-3. PMID: 33771188; PMCID: PMC8004431.

695: Zhuang P, Wu F, Mao L, Zhu F, Zhang Y, Chen X, Jiao J, Zhang Y. Egg and cholesterol consumption and mortality from cardiovascular and different causes in the United States: A population-based cohort study. PLoS Med. 2021 Feb 9;18(2):e1003508. doi: 10.1371/journal.pmed.1003508. PMID: 33561122; PMCID: PMC7872242.

696: Mitri J, Tomah S, Furtado J, Tasabehji MW, Hamdy O. Plasma Free Fatty Acids and Metabolic Effect in Type 2 Diabetes, an Ancillary Study from a Randomized Clinical Trial. Nutrients. 2021 Mar 31;13(4):1145. doi: 10.3390/nu13041145. PMID: 33807135; PMCID: PMC8065525.

697: Münzel T, Sørensen M, Lelieveld J, Hahad O, Al-Kindi S, Nieuwenhuijsen M, Giles-Corti B, Daiber A, Rajagopalan S. Heart healthy cities: genetics loads the gun but the environment pulls the trigger. Eur Heart J. 2021 Jul 1;42(25):2422-2438. doi: 10.1093/eurheartj/ehab235. PMID: 34005032; PMCID: PMC8248996.

698: Walker-Short E, Buckner T, Vigers T, Carry P, Vanderlinden LA, Dong F, Johnson RK, Yang IV, Kechris K, Rewers M, Norris JM. Epigenome-Wide Association Study of Infant Feeding and DNA Methylation in Infancy and Childhood in a Population at Increased Risk for Type 1 Diabetes. Nutrients. 2021 Nov 13;13(11):4057. doi: 10.3390/nu13114057. PMID: 34836312; PMCID: PMC8618577.

699: Dembiński Ł, Mazur A, Dąbrowski M, Jackowska T, Banaszkiewicz A. Knowledge of Medical Students and Medical Professionals Regarding Nutritional Deficiencies in Patients with Celiac Disease. Nutrients. 2021 May 22;13(6):1771. doi: 10.3390/nu13061771. PMID: 34067382; PMCID: PMC8224609.

700: Teo CH, Chin YS, Lim PY, Masrom SAH, Shariff ZM. Impacts of a School-Based Intervention That Incorporates Nutrition Education and a Supportive Healthy School Canteen Environment among Primary School Children in Malaysia. Nutrients. 2021 May 18;13(5):1712. doi: 10.3390/nu13051712. PMID: 34070053; PMCID: PMC8158127.

701: Kim Y, Chang Y, Kwon MJ, Hong YS, Kim MK, Sohn W, Cho YK, Shin H, Wild SH, Byrne CD, Ryu S. Fasting Ketonuria and the Risk of Incident Nonalcoholic Fatty Liver Disease With and Without Liver Fibrosis in Nondiabetic Adults. Am J Gastroenterol. 2021 Nov 1;116(11):2270-2278. doi: 10.14309/ajg.0000000000001344. PMID: 34114568.

702: Monteiro LZ, Varela AR, Lira BA, Rauber SB, Toledo JO, Spinola MDS, Carneiro MLA, Braga Junior F. Lifestyle and risk behaviors for chronic noncommunicable diseases among healthcare undergraduates in Midwest, Brazil. Cien Saude Colet. 2021 Jul;26(7):2911-2920. doi: 10.1590/1413-81232021267.20222019. Epub 2019 Nov 21. PMID: 34231703.

703: Mahmoudinezhad M, Farhangi MA, Kahroba H, Dehghan P. Personalized diet study of dietary advanced glycation end products (AGEs) and fatty acid desaturase 2 (FADS₂) genotypes in obesity. Sci Rep. 2021 Oct 5;11(1):19725. doi: 10.1038/s41598-021-99077-3. PMID: 34611217; PMCID: PMC8492634.

704: Menni C, Louca P, Berry SE, Vijay A, Astbury S, Leeming ER, Gibson R, Asnicar F, Piccinno G, Wolf J, Davies R, Mangino M, Segata N, Spector TD, Valdes AM. High intake of vegetables is linked to lower white blood cell profile and the effect is mediated by the gut microbiome. BMC Med. 2021 Feb 11;19(1):37. doi: 10.1186/s12916-021-01913-w. PMID: 33568158; PMCID: PMC7875684.

705: Ahrens AP, Culpepper T, Saldivar B, Anton S, Stoll S, Handberg EM, Xu K, Pepine C, Triplett EW, Aggarwal M. A Six-Day, Lifestyle-Based Immersion Program Mitigates Cardiovascular Risk Factors and Induces Shifts in Gut Microbiota, Specifically <i>Lachnospiraceae</i>, <i>Ruminococcaceae</i>, <i>Faecalibacterium prausnitzii</i>: A Pilot Study. Nutrients. 2021 Sep 29;13(10):3459. doi: 10.3390/nu13103459. PMID: 34684459; PMCID: PMC8539164.

706: Setavand Z, Ekramzadeh M, Honar N. Evaluation of malnutrition status and clinical indications in children with celiac disease: a cross-sectional study. BMC Pediatr. 2021 Mar 29;21(1):147. doi: 10.1186/s12887-021-02621-3. PMID: 33781226; PMCID: PMC8006373.

707: Picard K, Senior PA, Adame Perez S, Jindal K, Richard C, Mager DR. Low Mediterranean Diet scores are associated with reduced kidney function and health related quality of life but not other markers of cardiovascular risk in adults with diabetes and chronic kidney disease. Nutr Metab Cardiovasc Dis. 2021 May 6;31(5):1445-1453. doi: 10.1016/j.numecd.2021.02.002. Epub 2021 Feb 11. PMID: 33812736.

708: Deroover L, Vázquez-Castellanos JF, Vandermeulen G, Luypaerts A, Raes J, Courtin CM, Verbeke K. Wheat bran with reduced particle size increases serum SCFAs in obese subjects without improving health parameters compared with a maltodextrin placebo. Am J Clin Nutr. 2021 Oct 4;114(4):1328-1341. doi: 10.1093/ajcn/nqab196. PMID: 34224554.

709: Martínez-de-Quel Ó, Suárez-Iglesias D, López-Flores M, Pérez CA. Physical activity, dietary habits and sleep quality before and during COVID-19 lockdown: A longitudinal study. Appetite. 2021 Mar 1;158:105019. doi: 10.1016/j.appet.2020.105019. Epub 2020 Nov 5. PMID: 33161046; PMCID: PMC8580211.

710: Wu J, Zhu Y, Zhou L, Lu Y, Feng T, Dai M, Liu J, Xu W, Cheng W, Sun F, Liu H, Pan W, Yang X. Parasite-Derived Excretory-Secretory Products Alleviate Gut Microbiota Dysbiosis and Improve Cognitive Impairment Induced by a High-Fat Diet. Front Immunol. 2021 Oct 20;12:710513. doi: 10.3389/fimmu.2021.710513. PMID: 34745091; PMCID: PMC8564115.

711: Lim SY, Chan YM, Ramachandran V, Shariff ZM, Chin YS, Arumugam M. Dietary

Acid Load and Its Interaction with IGF1 (rs35767 and rs7136446) and IL6 (rs1800796) Polymorphisms on Metabolic Traits among Postmenopausal Women. Nutrients. 2021 Jun 23;13(7):2161. doi: 10.3390/nu13072161. PMID: 34201855; PMCID: PMC8308464.

712: Pourabbas M, Bagheri R, Hooshmand Moghadam B, Willoughby DS, Candow DG, Elliott BT, Forbes SC, Ashtary-Larky D, Eskandari M, Wong A, Dutheil F. Strategic Ingestion of High-Protein Dairy Milk during a Resistance Training Program Increases Lean Mass, Strength, and Power in Trained Young Males. Nutrients. 2021 Mar 15;13(3):948. doi: 10.3390/nu13030948. PMID: 33804259; PMCID: PMC7999866.

713: Andreu-Reinón ME, Chirlaque MD, Gavrila D, Amiano P, Mar J, Tainta M, Ardanaz E, Larumbe R, Colorado-Yohar SM, Navarro-Mateu F, Navarro C, Huerta JM. Mediterranean Diet and Risk of Dementia and Alzheimer's Disease in the EPIC-Spain Dementia Cohort Study. Nutrients. 2021 Feb 22;13(2):700. doi: 10.3390/nu13020700. PMID: 33671575; PMCID: PMC7927039.

714: Goonetilleke M, Kuk N, Correia J, Hodge A, Moore G, Gantier MP, Yeoh G, Sievert W, Lim R. Addressing the liver progenitor cell response and hepatic oxidative stress in experimental non-alcoholic fatty liver disease/non-alcoholic steatohepatitis using amniotic epithelial cells. Stem Cell Res Ther. 2021 Jul 28;12(1):429. doi: 10.1186/s13287-021-02476-6. PMID: 34321089; PMCID: PMC8317377.

715: Lin L, Wang A, He Y, Wang W, Gao Z, Tang X, Yan L, Wan Q, Luo Z, Qin G, Chen L, Mu Y, Dou J. Effects of the hemoglobin glycation index on hyperglycemia diagnosis: Results from the REACTION study. Diabetes Res Clin Pract. 2021 Oct;180:109039. doi: 10.1016/j.diabres.2021.109039. Epub 2021 Sep 3. PMID: 34481909.

716: Hossenbaccus L, Linton S, Ramchandani R, Gallant MJ, Ellis AK. Insights into allergic risk factors from birth cohort studies. Ann Allergy Asthma Immunol. 2021 Sep;127(3):312-317. doi: 10.1016/j.anai.2021.04.025. Epub 2021 May 7. PMID: 33971362.

717: Slurink IAL, den Braver NR, Rutters F, Kupper N, Smeets T, Elders PJM, Beulens JWJ, Soedamah-Muthu SS. Dairy product consumption and incident prediabetes in Dutch middle-aged adults: the Hoorn Studies prospective cohort. Eur J Nutr. 2022 Feb;61(1):183-196. doi: 10.1007/s00394-021-02626-9. Epub 2021 Jul 10. PMID: 34245355; PMCID: PMC8783852.

718: Zhang G, Li R, Li W, Yang S, Sun Q, Yin H, Wang C, Hou B, Wang H, Yu L, Chen R, Shi L, Zhang K, Liew CW, Qiang G, Sun Q, Liu C. Toll-like receptor 3 ablation prevented high-fat diet-induced obesity and metabolic disorder. J Nutr Biochem. 2021 Sep;95:108761. doi: 10.1016/j.jnutbio.2021.108761. Epub 2021 Jun 6. PMID: 33965533.

719: Leung GKW, Davis R, Huggins CE, Ware RS, Bonham MP. Does rearranging meal

times at night improve cardiovascular risk factors? An Australian pilot randomised trial in night shift workers. Nutr Metab Cardiovasc Dis. 2021 Jun 7;31(6):1890-1902. doi: 10.1016/j.numecd.2021.03.008. Epub 2021 Mar 19. PMID: 33994064.

720: Smith IC, Ostertag C, O'Reilly JJ, Rios JL, Klancic T, MacDonald GZ, Collins KH, Reimer RA, Herzog W. Contractility of permeabilized rat vastus intermedius muscle fibres following high-fat, high-sucrose diet consumption. Appl Physiol Nutr Metab. 2021 Nov;46(11):1389-1399. doi: 10.1139/apnm-2021-0238. Epub 2021 Jun 17. PMID: 34139131.

721: Sayar S, Aykut H, Kaya Ö, Kürbüz K, Ak Ç, Gökçen P, Mutlu Bilgiç N, Adalı G, Kahraman R, Doganay L, Özdil K. Bone Mineral Density Screening and the Frequency of Osteopenia/Osteoporosis in Turkish Adult Patients With Celiac Disease. Turk J Gastroenterol. 2021 Jul;32(7):600-607. doi: 10.5152/tjg.2021.20313. PMID: 34464324.

722: Bandhu Kalanidhi K, Ranjan P, Sarkar S, Kaur T, Dutt Upadhyay A, Singh A, Sahu A, Khan M, Vijay Prasad B, Baitha U, Kumar A. Development and validation of a questionnaire to assess socio-behavioural impact of COVID-19 on the general population. Diabetes Metab Syndr. 2021 Mar-Apr;15(2):601-603. doi: 10.1016/j.dsx.2021.02.019. Epub 2021 Feb 18. PMID: 33714135; PMCID: PMC7889470.

723: Caballero FF, Struijk EA, Lana A, Buño A, Rodríguez-Artalejo F, Lopez-Garcia E. Plasma acylcarnitines and risk of lower-extremity functional impairment in older adults: a nested case-control study. Sci Rep. 2021 Feb 8;11(1):3350. doi: 10.1038/s41598-021-82912-y. PMID: 33558555; PMCID: PMC7870673.

724: Wang SM, Katki HA, Graubard BI, Kahle LL, Chaturvedi A, Matthews CE, Freedman ND, Abnet CC. Population Attributable Risks of Subtypes of Esophageal and Gastric Cancers in the United States. Am J Gastroenterol. 2021 Sep 1;116(9):1844-1852. doi: 10.14309/ajg.0000000000001355. PMID: 34240714; PMCID: PMC8410651.

725: Patikorn C, Roubal K, Veettil SK, Chandran V, Pham T, Lee YY, Giovannucci EL, Varady KA, Chaiyakunapruk N. Intermittent Fasting and Obesity-Related Health Outcomes: An Umbrella Review of Meta-analyses of Randomized Clinical Trials. JAMA Netw Open. 2021 Dec 1;4(12):e2139558. doi: 10.1001/jamanetworkopen.2021.39558. PMID: 34919135; PMCID: PMC8683964.

726: Fois A, Torreggiani M, Trabace T, Chatrenet A, Longhitano E, Mazé B, Lippi F, Vigreux J, Beaumont C, Moio MR, Piccoli GB. Quality of Life in CKD Patients on Low-Protein Diets in a Multiple-Choice Diet System. Comparison between a French and an Italian Experience. Nutrients. 2021 Apr 18;13(4):1354. doi: 10.3390/nu13041354. PMID: 33919635; PMCID: PMC8073895.

727: van Keulen HM, van Breukelen G, de Vries H, Brug J, Mesters I. A randomized controlled trial comparing community lifestyle interventions to improve

adherence to diet and physical activity recommendations: the VitalUM study. Eur J Epidemiol. 2021 Mar;36(3):345-360. doi: 10.1007/s10654-020-00708-2. Epub 2020 Dec 30. PMID: 33377998; PMCID: PMC8032577.

728: van der Meer TP, Chung MK, van Faassen M, Makris KC, van Beek AP, Kema IP, Wolffenbuttel BHR, van Vliet-Ostaptchouk JV, Patel CJ. Temporal exposure and consistency of endocrine disrupting chemicals in a longitudinal study of individuals with impaired fasting glucose. Environ Res. 2021 Jun;197:110901. doi: 10.1016/j.envres.2021.110901. Epub 2021 Feb 20. PMID: 33617867.

729: Wu W, Bours MJL, Koole A, Kenkhuis MF, Eussen SJPM, Breukink SO, van Schooten FJ, Weijenberg MP, Hageman GJ. Cross-Sectional Associations between Dietary Daily Nicotinamide Intake and Patient-Reported Outcomes in Colorectal Cancer Survivors, 2 to 10 Years Post-Diagnosis. Nutrients. 2021 Oct 21;13(11):3707. doi: 10.3390/nu13113707. PMID: 34835963; PMCID: PMC8624000.

730: Taft TH, Carlson DA, Simons M, Zavala S, Hirano I, Gonsalves N, Pandolfino JE. Esophageal Hypervigilance and Symptom-Specific Anxiety in Patients with Eosinophilic Esophagitis. Gastroenterology. 2021 Oct;161(4):1133-1144. doi: 10.1053/j.gastro.2021.06.023. Epub 2021 Jun 19. PMID: 34153298; PMCID: PMC8463417.

731: Bil J, Możeńska O. The vicious cycle: a history of obesity and COVID-19. BMC Cardiovasc Disord. 2021 Jul 6;21(1):332. doi: 10.1186/s12872-021-02134-y. PMID: 34229605; PMCID: PMC8258476.

732: Minsky NC, Pachter D, Zacay G, Chishlevitz N, Ben-Hamo M, Weiner D, Segal-Lieberman G. Managing Obesity in Lockdown: Survey of Health Behaviors and Telemedicine. Nutrients. 2021 Apr 19;13(4):1359. doi: 10.3390/nu13041359. PMID: 33921602; PMCID: PMC8073707.

733: Buresh R, Kliszczewicz B, Hayes K, Julian J. Steps expressed relative to body fat mass predicts body composition and cardiometabolic risk in adults eating ad libitum. J Sports Med Phys Fitness. 2022 Jan;62(1):65-73. doi: 10.23736/S0022-4707.21.12038-9. Epub 2021 Feb 10. PMID: 33565754.

734: Muscogiuri G, Barrea L, Aprano S, Framondi L, Di Matteo R, Altieri B, Laudisio D, Pugliese G, Savastano S, Colao A. Chronotype and cardio metabolic health in obesity: does nutrition matter? Int J Food Sci Nutr. 2021 Nov;72(7):892-900. doi: 10.1080/09637486.2021.1885017. Epub 2021 Mar 24. PMID: 33759693.

735: Macicame I, Prista A, Parhofer KG, Cavele N, Manhiça C, Nhachungue S, Saathoff E, Rehfuess E. Social determinants and behaviors associated with overweight and obesity among youth and adults in a peri-urban area of Maputo City, Mozambique. J Glob Health. 2021 Mar 27;11:04021. doi: 10.7189/jogh.11.04021. PMID: 33868672; PMCID: PMC8038757.

736: Sinopoulou V, Gordon M, Dovey TM, Akobeng AK. Interventions for the

management of abdominal pain in ulcerative colitis. Cochrane Database Syst Rev. 2021 Jul 22;7(7):CD013589. doi: 10.1002/14651858.CD013589.pub2. PMID: 34291816; PMCID: PMC8407332.

737: Shah NA, Levy CJ. Emerging technologies for the management of type 2 diabetes mellitus. J Diabetes. 2021 Sep;13(9):713-724. doi: 10.1111/1753-0407.13188. Epub 2021 May 11. PMID: 33909352.

738: Zhong GC, Hu TY, Yang PF, Peng Y, Wu JJ, Sun WP, Cheng L, Wang CR. Chocolate consumption and all-cause and cause-specific mortality in a US population: a post hoc analysis of the PLCO cancer screening trial. Aging (Albany NY). 2021 Jul 29;13(14):18564-18585. doi: 10.18632/aging.203302. Epub 2021 Jul 29. PMID: 34329196; PMCID: PMC8351724.

739: Blekkenhorst LC, Sim M, Radavelli-Bagatini S, Bondonno NP, Bondonno CP, Devine A, Schousboe JT, Lim WH, Kiel DP, Woodman RJ, Hodgson JM, Prince RL, Lewis JR. Cruciferous vegetable intake is inversely associated with extensive abdominal aortic calcification in elderly women: a cross-sectional study. Br J Nutr. 2021 Feb 14;125(3):337-345. doi: 10.1017/S0007114520002706. Epub 2020 Jul 17. PMID: 32674743; PMCID: PMC7844610.

740: Ni Y, Szpiro A, Loftus C, Tylavsky F, Kratz M, Bush NR, LeWinn KZ, Sathyanarayana S, Enquobahrie DA, Davis R, Fitzpatrick AL, Sonney J, Zhao Q, Karr CJ. Associations Between Maternal Nutrition in Pregnancy and Child Blood Pressure at 4-6 Years: A Prospective Study in a Community-Based Pregnancy Cohort. J Nutr. 2021 Apr 8;151(4):949-961. doi: 10.1093/jn/nxaa395. PMID: 33561258; PMCID: PMC8030724.

741: DiVito B, Talavlikar R, Seifu S. Common Hematologic, Nutritional, Asthma/Allergic Conditions and Lead Screening/Management. Prim Care. 2021 Mar;48(1):67-81. doi: 10.1016/j.pop.2020.10.002. Epub 2020 Nov 27. PMID: 33516425.

742: Beaulieu K, Blundell JE, van Baak MA, Battista F, Busetto L, Carraça EV, Dicker D, Encantado J, Ermolao A, Farpour-Lambert N, Pramono A, Woodward E, Bellicha A, Oppert JM. Effect of exercise training interventions on energy intake and appetite control in adults with overweight or obesity: A systematic review and meta-analysis. Obes Rev. 2021 Jul;22 Suppl 4(Suppl 4):e13251. doi: 10.1111/obr.13251. Epub 2021 May 5. PMID: 33949089; PMCID: PMC8365695.

743: Bolesławska I, Błaszczyk-Bębenek E, Jagielski P, Jagielska A, Przysławski J. Nutritional behaviors of women and men in Poland during confinement related to the SARS-CoV-2 epidemic. Sci Rep. 2021 Oct 7;11(1):19984. doi: 10.1038/s41598-021-99561-w. PMID: 34620981; PMCID: PMC8497511.

744: Lo K, Glenn AJ, Yeung S, Kendall CWC, Sievenpiper JL, Jenkins DJA, Woo J. Prospective Association of the Portfolio Diet with All-Cause and Cause-Specific Mortality Risk in the Mr. OS and Ms. OS Study. Nutrients. 2021 Dec 3;13(12):4360. doi: 10.3390/nu13124360. PMID: 34959911; PMCID: PMC8705939.

745: Wang Z, Groen H, Cantineau AEP, van Elten TM, Karsten MDA, van Oers AM, Mol BWJ, Roseboom TJ, Hoek A. Effectiveness of a 6-Month Lifestyle Intervention on Diet, Physical Activity, Quality of Life, and Markers of Cardiometabolic Health in Women with PCOS and Obesity and Non-PCOS Obese Controls: One Size Fits All? Nutrients. 2021 Sep 28;13(10):3425. doi: 10.3390/nu13103425. PMID: 34684438; PMCID: PMC8538637.

746: Lyu C, Tsinovoi CL, Xun P, Song Y, Pu Y, Rosanoff A, Iribarren C, Schreiner PJ, Shikany JM, Jacobs DR, Kahe K. Magnesium intake was inversely associated with hostility among American young adults. Nutr Res. 2021 May;89:35-44. doi: 10.1016/j.nutres.2021.01.001. Epub 2021 Apr 21. PMID: 33894659; PMCID: PMC8098670.

747: Liddle DM , Lin X , Ward EM , Cox LC , Wright AJ , Robinson LE . Apple consumption reduces markers of postprandial inflammation following a high fat meal in overweight and obese adults: A randomized, crossover trial. Food Funct. 2021 Jul 21;12(14):6348-6362. doi: 10.1039/d1fo00392e. Epub 2021 Jun 8. PMID: 34105575.

748: Bellinge JW, Dalgaard F, Murray K, Connolly E, Blekkenhorst LC, Bondonno CP, Lewis JR, Sim M, Croft KD, Gislason G, Torp-Pedersen C, Tjønneland A, Overvad K, Hodgson JM, Schultz C, Bondonno NP. Vitamin K Intake and Atherosclerotic Cardiovascular Disease in the Danish Diet Cancer and Health Study. J Am Heart Assoc. 2021 Aug 17;10(16):e020551. doi: 10.1161/JAHA.120.020551. Epub 2021 Aug 7. PMID: 34369182; PMCID: PMC8475061.

749: Jin X, He W, Zhang Y, Gong E, Niu Z, Ji J, Li Y, Zeng Y, Yan LL. Association of APOE ε4 genotype and lifestyle with cognitive function among Chinese adults aged 80 years and older: A cross-sectional study. PLoS Med. 2021 Jun 1;18(6):e1003597. doi: 10.1371/journal.pmed.1003597. PMID: 34061824; PMCID: PMC8168868.

750: Botelho J, Vicente F, Dias L, Júdice A, Pereira P, Proença L, Machado V, Chambrone L, Mendes JJ. Periodontal Health, Nutrition and Anthropometry in Professional Footballers: A Preliminary Study. Nutrients. 2021 May 25;13(6):1792. doi: 10.3390/nu13061792. PMID: 34070244; PMCID: PMC8225082.

751: Snow SJ, Henriquez AR, Fisher A, Vallanat B, House JS, Schladweiler MC, Wood CE, Kodavanti UP. Peripheral metabolic effects of ozone exposure in healthy and diabetic rats on normal or high-cholesterol diet. Toxicol Appl Pharmacol. 2021 Mar 15;415:115427. doi: 10.1016/j.taap.2021.115427. Epub 2021 Jan 30. PMID: 33524448; PMCID: PMC8086744.

752: Li C, Xing C, Zhang J, Zhao H, Shi W, He B. Eight-hour time-restricted feeding improves endocrine and metabolic profiles in women with anovulatory polycystic ovary syndrome. J Transl Med. 2021 Apr 13;19(1):148. doi: 10.1186/s12967-021-02817-2. PMID: 33849562; PMCID: PMC8045367.

753: Crook J, Horgas A, Yoon SJ, Grundmann O, Johnson-Mallard V. Insufficient Vitamin C Levels among Adults in the United States: Results from the NHANES Surveys, 2003-2006. Nutrients. 2021 Oct 30;13(11):3910. doi: 10.3390/nu13113910. PMID: 34836166; PMCID: PMC8625707.

754: Genoni G, Menegon V, Monzani A, Archero F, Tagliaferri F, Mancioppi V, Peri C, Bellone S, Prodam F. Healthy Lifestyle Intervention and Weight Loss Improve Cardiovascular Dysfunction in Children with Obesity. Nutrients. 2021 Apr 15;13(4):1301. doi: 10.3390/nu13041301. PMID: 33920831; PMCID: PMC8071179.

755: Pengpid S, Peltzer K. Ideal Cardiovascular Health Behaviours in Nationally Representative School-Based Samples of Adolescents in the Caribbean. Vasc Health Risk Manag. 2021 May 4;17:187-194. doi: 10.2147/VHRM.S302168. PMID: 33976549; PMCID: PMC8106475.

756: Aittola K, Karhunen L, Männikkö R, Järvelä-Reijonen E, Mikkonen S, Absetz P, Kolehmainen M, Schwab U, Harjumaa M, Lindström J, Lakka T, Tilles-Tirkkonen T, Pihlajamäki J. Enhanced Eating Competence Is Associated with Improved Diet Quality and Cardiometabolic Profile in Finnish Adults with Increased Risk of Type 2 Diabetes. Nutrients. 2021 Nov 11;13(11):4030. doi: 10.3390/nu13114030. PMID: 34836283; PMCID: PMC8624442.

757: Zhu F, Qin Y, Bi Y, Su J, Cui L, Luo P, Du W, Miao W, Wang J, Zhou J. Fresh vegetable and fruit consumption and carotid atherosclerosis in high-cardiovascular-risk population: a cross-sectional study in Jiangsu, China. Cad Saude Publica. 2021 Jun 9;37(5):e00033020. doi: 10.1590/01021-311X00033020. PMID: 34133634.

758: Wei W, Jiang W, Huang J, Xu J, Wang X, Jiang X, Wang Y, Li G, Sun C, Li Y, Han T. Association of Meal and Snack Patterns With Mortality of All-Cause, Cardiovascular Disease, and Cancer: The US National Health and Nutrition Examination Survey, 2003 to 2014. J Am Heart Assoc. 2021 Jul 6;10(13):e020254. doi: 10.1161/JAHA.120.020254. Epub 2021 Jun 23. PMID: 34157852; PMCID: PMC8403276.

759: Pereira TS, Fonseca FAH, Fonseca MIH, Martins CM, Fonseca HAR, Fonzar WT, Goulart AC, Bensenor IM, Lotufo PA, Izar MC. Phytosterol consumption and markers of subclinical atherosclerosis: Cross-sectional results from ELSA-Brasil. Nutr Metab Cardiovasc Dis. 2021 Jun 7;31(6):1756-1766. doi: 10.1016/j.numecd.2021.02.031. Epub 2021 Mar 20. PMID: 33965299.

760: Mahboub HH, Beheiry RR, Shahin SE, Behairy A, Khedr MHE, Ibrahim SM, Elshopakey GE, Daoush WM, Altohamy DE, Ismail TA, El-Houseiny W. Adsorptivity of mercury on magnetite nano-particles and their influences on growth, economical, hemato-biochemical, histological parameters and bioaccumulation in Nile tilapia (Oreochromis niloticus). Aquat Toxicol. 2021 Jun;235:105828. doi: 10.1016/j.aquatox.2021.105828. Epub 2021 Apr 20. PMID: 33901865.

761: Tao S, Wu X, Li S, Ma L, Yu Y, Sun G, Zhang Y, Li T, Tao F. Circadian

rhythm abnormalities during the COVID-19 outbreak related to mental health in China: a nationwide university-based survey. Sleep Med. 2021 Aug;84:165-172. doi: 10.1016/j.sleep.2021.05.028. Epub 2021 Jun 6. PMID: 34153799.

762: Ahuja V, Aronen P, Pramodkumar TA, Looker H, Chetrit A, Bloigu AH, Juutilainen A, Bianchi C, La Sala L, Anjana RM, Pradeepa R, Venkatesan U, Jebarani S, Baskar V, Fiorentino TV, Timpel P, DeFronzo RA, Ceriello A, Del Prato S, Abdul-Ghani M, Keinänen-Kiukaanniemi S, Dankner R, Bennett PH, Knowler WC, Schwarz P, Sesti G, Oka R, Mohan V, Groop L, Tuomilehto J, Ripatti S, Bergman M, Tuomi T. Accuracy of 1-Hour Plasma Glucose During the Oral Glucose Tolerance Test in Diagnosis of Type 2 Diabetes in Adults: A Meta-analysis. Diabetes Care. 2021 Apr;44(4):1062-1069. doi: 10.2337/dc20-1688. Erratum in: Diabetes Care. 2021 Apr 30;: PMID: 33741697; PMCID: PMC8578930.

763: Lari A, Sohouli MH, Fatahi S, Cerqueira HS, Santos HO, Pourrajab B, Rezaei M, Saneie S, Rahideh ST. The effects of the Dietary Approaches to Stop Hypertension (DASH) diet on metabolic risk factors in patients with chronic disease: A systematic review and meta-analysis of randomized controlled trials. Nutr Metab Cardiovasc Dis. 2021 Sep 22;31(10):2766-2778. doi: 10.1016/j.numecd.2021.05.030. Epub 2021 Jun 10. PMID: 34353704.

764: Carballo-Casla A, Ortolá R, García-Esquinas E, Oliveira A, Sotos-Prieto M, Lopes C, Lopez-Garcia E, Rodríguez-Artalejo F. The Southern European Atlantic Diet and all-cause mortality in older adults. BMC Med. 2021 Feb 9;19(1):36. doi: 10.1186/s12916-021-01911-y. PMID: 33557823; PMCID: PMC7871632.

765: Muzaffar R, Khan MA, Mushtaq MH, Nasir M, Khan A, Haq IU, Muhammad J. Hyperhomocysteinemia as an Independent Risk Factor for Coronary Heart Disease. Comparison with Conventional Risk Factors. Braz J Biol. 2021 Sep 6;83:e249104. doi: 10.1590/1519-6984.249104. PMID: 34495168.

766: Wang X, Lv J, Yu C, Li L, Hu Y, Qin LQ, Dong JY. Dietary Soy Consumption and Cardiovascular Mortality among Chinese People with Type 2 Diabetes. Nutrients. 2021 Jul 23;13(8):2513. doi: 10.3390/nu13082513. PMID: 34444673; PMCID: PMC8398979.

767: Ismael S, Silvestre MP, Vasques M, Araújo JR, Morais J, Duarte MI, Pestana D, Faria A, Pereira-Leal JB, Vaz J, Ribeiro P, Teixeira D, Marques C, Calhau C. A Pilot Study on the Metabolic Impact of Mediterranean Diet in Type 2 Diabetes: Is Gut Microbiota the Key? Nutrients. 2021 Apr 8;13(4):1228. doi: 10.3390/nu13041228. PMID: 33917736; PMCID: PMC8068165.

768: Duc HN, Oh H, Yoon IM, Kim MS. Association between levels of thiamine intake, diabetes, cardiovascular diseases and depression in Korea: a national cross-sectional study. J Nutr Sci. 2021 Apr 27;10:e31. doi: 10.1017/jns.2021.23. PMID: 34094512; PMCID: PMC8141681.

769: Lu J, Zhang L, Zhai Q, Zhao J, Zhang H, Lee YK, Lu W, Li M, Chen W. Chinese gut microbiota and its associations with staple food type, ethnicity, and

urbanization. NPJ Biofilms Microbiomes. 2021 Sep 6;7(1):71. doi: 10.1038/s41522-021-00245-0. PMID: 34489454; PMCID: PMC8421333.

770: Khocht A, Orlich M, Paster B, Bellinger D, Lenoir L, Irani C, Fraser G. Cross-sectional comparisons of subgingival microbiome and gingival fluid inflammatory cytokines in periodontally healthy vegetarians versus non-vegetarians. J Periodontal Res. 2021 Dec;56(6):1079-1090. doi: 10.1111/jre.12922. Epub 2021 Aug 27. PMID: 34449089.

771: Pinho L, Correia T, Sampaio F, Sequeira C, Teixeira L, Lopes M, Fonseca C. The use of mental health promotion strategies by nurses to reduce anxiety, stress, and depression during the COVID-19 outbreak: A prospective cohort study. Environ Res. 2021 Apr;195:110828. doi: 10.1016/j.envres.2021.110828. Epub 2021 Feb 4. PMID: 33548294; PMCID: PMC7857980.

772: Matheson EM, Nelson JL, Baggs GE, Luo M, Deutz NE. Specialized oral nutritional supplement (ONS) improves handgrip strength in hospitalized, malnourished older patients with cardiovascular and pulmonary disease: A randomized clinical trial. Clin Nutr. 2021 Mar;40(3):844-849. doi: 10.1016/j.clnu.2020.08.035. Epub 2020 Sep 5. PMID: 32943241.

773: Nogay NH, Walton J, Roberts KM, Nahikian-Nelms M, Witwer AN. The Effect of the Low FODMAP Diet on Gastrointestinal Symptoms, Behavioral Problems and Nutrient Intake in Children with Autism Spectrum Disorder: A Randomized Controlled Pilot Trial. J Autism Dev Disord. 2021 Aug;51(8):2800-2811. doi: 10.1007/s10803-020-04717-8. PMID: 33057858.

774: Sciarrillo CM, Koemel NA, Keirns BH, Banks NF, Rogers EM, Rosenkranz SK, Kurti SP, Jenkins NDM, Emerson SR. Who would benefit most from postprandial lipid screening? Clin Nutr. 2021 Jul;40(7):4762-4771. doi: 10.1016/j.clnu.2021.04.022. Epub 2021 Apr 20. PMID: 34242916.

775: Jin YF, Dai T, Yu C, Zheng S, Nie YH, Wang MZ, Bai YN. [Effects of ambient particulate matter (PM₁₀) on prevalence of diabetes and fasting plasma glucose]. Zhonghua Yu Fang Yi Xue Za Zhi. 2021 Oct 6;55(10):1196-1202. Chinese. doi: 10.3760/cma.j.cn112150-20210305-00222. PMID: 34706504.

776: Hollstein T, Vinales K, Chen KY, Cypess AM, Basolo A, Schlögl M, Krakoff J, Piaggi P. Reduced brown adipose tissue activity during cold exposure is a metabolic feature of the human thrifty phenotype. Metabolism. 2021 Apr;117:154709. doi: 10.1016/j.metabol.2021.154709. Epub 2021 Jan 18. PMID: 33476636; PMCID: PMC7956243.

777: Hummel J, Fritsche L, Vosseler A, Dannecker C, Hoene M, Kantartzis K, Häring HU, Stefan N, Machann J, Birkenfeld AL, Weigert C, Wagner R, Peter A, Fritsche A, Heni M. Free fatty acids, glicentin and glucose-dependent insulinotropic polypeptide as potential major determinants of fasting substrate oxidation. Sci Rep. 2021 Aug 17;11(1):16642. doi: 10.1038/s41598-021-95750-9. PMID: 34404813; PMCID: PMC8371005.

778: Kruger HS, Ricci C, Pieters M, Botha-le Roux S, Moss SJ, Kruger IM, van Zyl T, Schutte AE. Lifestyle factors associated with the transition from healthy to unhealthy adiposity among black South African adults over 10 years. Nutr Metab Cardiovasc Dis. 2021 Jun 30;31(7):2023-2032. doi: 10.1016/j.numecd.2021.03.017. Epub 2021 Mar 26. PMID: 33975737.

779: Fletcher E, Gordon PM. Obesity-induced alterations to the immunoproteasome: a potential link to intramuscular lipotoxicity. Appl Physiol Nutr Metab. 2021 May;46(5):485-493. doi: 10.1139/apnm-2020-0655. Epub 2020 Nov 13. PMID: 33186056.

780: Bendavid I, Lobo DN, Barazzoni R, Cederholm T, Coëffier M, de van der Schueren M, Fontaine E, Hiesmayr M, Laviano A, Pichard C, Singer P. The centenary of the Harris-Benedict equations: How to assess energy requirements best? Recommendations from the ESPEN expert group. Clin Nutr. 2021 Mar;40(3):690-701. doi: 10.1016/j.clnu.2020.11.012. Epub 2020 Nov 20. PMID: 33279311.

781: Seo JW, Park SB. The Association of Hemoglobin A1c and Fasting Glucose Levels with hs-CRP in Adults Not Diagnosed with Diabetes from the KNHANES, 2017. J Diabetes Res. 2021 Apr 1;2021:5585938. doi: 10.1155/2021/5585938. PMID: 33869637; PMCID: PMC8035032.

782: Zhu R, Fogelholm M, Poppitt SD, Silvestre MP, Møller G, Huttunen-Lenz M, Stratton G, Sundvall J, Råman L, Jalo E, Taylor MA, Macdonald IA, Handjiev S, Handjieva-Darlenska T, Martinez JA, Muirhead R, Brand-Miller J, Raben A. Adherence to a Plant-Based Diet and Consumption of Specific Plant Foods-Associations with 3-Year Weight-Loss Maintenance and Cardiometabolic Risk Factors: A Secondary Analysis of the PREVIEW Intervention Study. Nutrients. 2021 Nov 1;13(11):3916. doi: 10.3390/nu13113916. PMID: 34836170; PMCID: PMC8618731.

783: Shen X, Xiao S, Liu R, Tong G, Liu T, Wang D. Personalized hypertension management based on serial assessment and telemedicine (PHMA): a cluster randomize controlled trial protocol in Anhui, China. BMC Cardiovasc Disord. 2021 Mar 12;21(1):135. doi: 10.1186/s12872-021-01943-5. PMID: 33711941; PMCID: PMC7953659.

784: Kazemi M, Kim JY, Parry SA, Azziz R, Lujan ME. Disparities in cardio metabolic risk between Black and White women with polycystic ovary syndrome: a systematic review and meta-analysis. Am J Obstet Gynecol. 2021 May;224(5):428-444.e8. doi: 10.1016/j.ajog.2020.12.019. Epub 2020 Dec 13. PMID: 33316275.

785: Kremers SHM, Remmelzwaal S, Schalkwijk CG, Elders PJM, Stehouwer CDA, van Ballegooijen AJ, Beulens JWJ. The role of serum and dietary advanced glycation endproducts in relation to cardiac function and structure: The Hoorn Study. Nutr Metab Cardiovasc Dis. 2021 Oct 28;31(11):3167-3175. doi: 10.1016/j.numecd.2021.07.020. Epub 2021 Jul 27. PMID: 34518083.

786: Hamedi-Shahraki S, Mir F, Amirkhizi F. Food Insecurity and Cardiovascular Risk Factors among Iranian Women. Ecol Food Nutr. 2021 Mar-Apr;60(2):163-181. doi: 10.1080/03670244.2020.1812596. Epub 2020 Sep 1. PMID: 32869659.

787: Zhu J, Zhao Q, Qiu Y, Zhang Y, Cui S, Yu Y, Chen B, Zhu M, Wang N, Liu X, Jiang Y, Xu W, Zhao G. Soy Isoflavones Intake and Obesity in Chinese Adults: A Cross-Sectional Study in Shanghai, China. Nutrients. 2021 Aug 6;13(8):2715. doi: 10.3390/nu13082715. PMID: 34444874; PMCID: PMC8399780.

788: Schwingshackl L, Schünemann HJ, Meerpohl JJ. Improving the trustworthiness of findings from nutrition evidence syntheses: assessing risk of bias and rating the certainty of evidence. Eur J Nutr. 2021 Sep;60(6):2893-2903. doi: 10.1007/s00394-020-02464-1. Epub 2020 Dec 30. PMID: 33377996; PMCID: PMC8354882.

789: Chung HS, Lee JS, Kim JA, Roh E, Lee YB, Hong SH, Yu JH, Kim NH, Yoo HJ, Seo JA, Kim SG, Kim NH, Baik SH, Choi KM. Fasting plasma glucose variability in midlife and risk of Parkinson's disease: A nationwide population-based study. Diabetes Metab. 2021 May;47(3):101195. doi: 10.1016/j.diabet.2020.08.006. Epub 2020 Oct 8. PMID: 33039669.

790: Tang Z, Ming Y, Wu M, Jing J, Xu S, Li H, Zhu Y. Effects of Caloric Restriction and Rope-Skipping Exercise on Cardiometabolic Health: A Pilot Randomized Controlled Trial in Young Adults. Nutrients. 2021 Sep 16;13(9):3222. doi: 10.3390/nu13093222. PMID: 34579097; PMCID: PMC8467906.

791: Mohamed A, Bolen C, Morgan J, Rice PA, Speas M, Abdelmonem A, Russo C. Reducing Morning Hypoglycemia Among Children Undergoing Treatment for Acute Lymphoblastic Leukemia. JCO Oncol Pract. 2021 Jun;17(6):e901-e907. doi: 10.1200/OP.20.00652. Epub 2021 Mar 17. PMID: 33729828.

792: Tolosa J, Rodríguez-Carrasco Y, Graziani G, Gaspari A, Ferrer E, Mañes J, Ritieni A. Mycotoxin Occurrence and Risk Assessment in Gluten-Free Pasta through UHPLC-Q-Exactive Orbitrap MS. Toxins (Basel). 2021 Apr 25;13(5):305. doi: 10.3390/toxins13050305. PMID: 33923097; PMCID: PMC8146712.

793: Comeglio P, Sarchielli E, Filippi S, Cellai I, Guarnieri G, Morelli A, Rastrelli G, Maseroli E, Cipriani S, Mello T, Galli A, Bruno BJ, Kim K, Vangara K, Papangkorn K, Chidambaram N, Patel MV, Maggi M, Vignozzi L. Treatment potential of LPCN 1144 on liver health and metabolic regulation in a non-genomic, high fat diet induced NASH rabbit model. J Endocrinol Invest. 2021 Oct;44(10):2175-2193. doi: 10.1007/s40618-021-01522-7. Epub 2021 Feb 13. PMID: 33586025; PMCID: PMC8421272.

794: Ma Q, Li R, Wang L, Yin P, Wang Y, Yan C, Ren Y, Qian Z, Vaughn MG, McMillin SE, Hay SI, Naghavi M, Cai M, Wang C, Zhang Z, Zhou M, Lin H, Yang Y. Temporal trend and attributable risk factors of stroke burden in China, 1990-2019: an analysis for the Global Burden of Disease Study 2019. Lancet Public Health. 2021 Dec;6(12):e897-e906. doi: 10.1016/S2468-2667(21)00228-0.

PMID: 34838196.

795: Matteo G, Hoyeck MP, Blair HL, Zebarth J, Rick KRC, Williams A, Gagné R, Buick JK, Yauk CL, Bruin JE. Prolonged Low-Dose Dioxin Exposure Impairs Metabolic Adaptability to High-Fat Diet Feeding in Female but Not Male Mice. Endocrinology. 2021 Jun 1;162(6):bqab050. doi: 10.1210/endocr/bqab050. PMID: 33693622; PMCID: PMC8101695.

796: Melhem S, Steven S, Taylor R, Al-Mrabeh A. Effect of Weight Loss by Low-Calorie Diet on Cardiovascular Health in Type 2 Diabetes: An Interventional Cohort Study. Nutrients. 2021 Apr 26;13(5):1465. doi: 10.3390/nu13051465. PMID: 33925808; PMCID: PMC8146720.

797: Liu J, Cheng NN, Zhou ZY, Zhang Y, Yang J, Liu LS, Song Y, Huang X, Tang GF, Wang BY, Qin XH, Xu XP, Kong XQ. Effect of fasting blood glucose on risk of new-onset hypertension in rural Chinese population: a 15-year follow-up cohort. BMC Cardiovasc Disord. 2021 Nov 8;21(1):531. doi: 10.1186/s12872-021-02336-4. PMID: 34749652; PMCID: PMC8573915.

798: Swareldhab ESE, Al-Jawaldeh A, Qureshi AB, Ali AME, Abu-Manga M, Al-Areeqi M, Dureab F. Assessment of Micronutrient Situation among Reproductive-Age Women (15-49) and Under-Five Children in Sudan. Nutrients. 2021 Aug 13;13(8):2784. doi: 10.3390/nu13082784. PMID: 34444943; PMCID: PMC8399272.

799: Proli F, Faragalli A, Talbotec C, Bucci A, Zemrani B, Chardot C, Abi Nader E, Goulet O, Lambe C. Variation of plasma citrulline as a predictive factor for weaning off long-term parenteral nutrition in children with neonatal short bowel syndrome. Clin Nutr. 2021 Aug;40(8):4941-4947. doi: 10.1016/j.clnu.2021.07.017. Epub 2021 Jul 18. PMID: 34358840.

800: Kapoor N, Al Najim W, Menezes C, Price RK, O'Boyle C, Bodnar Z, Spector AC, Docherty NG, le Roux CW. A Comparison of Total Food Intake at a Personalised Buffet in People with Obesity, before and 24 Months after Roux-en-Y-Gastric Bypass Surgery. Nutrients. 2021 Oct 29;13(11):3873. doi: 10.3390/nu13113873. PMID: 34836130; PMCID: PMC8625776.

801: Truijen SPM, Hayhoe RPG, Hooper L, Schoenmakers I, Forbes A, Welch AA. Predicting Malnutrition Risk with Data from Routinely Measured Clinical Biochemical Diagnostic Tests in Free-Living Older Populations. Nutrients. 2021 May 31;13(6):1883. doi: 10.3390/nu13061883. PMID: 34072686; PMCID: PMC8226876.

802: Gong Q, Zhang P, Wang J, Gregg EW, Cheng YJ, Li G, Bennett PH; Da Qing Diabetes Prevention Outcome Study Group. Efficacy of lifestyle intervention in adults with impaired glucose tolerance with and without impaired fasting plasma glucose: A post hoc analysis of Da Qing Diabetes Prevention Outcome Study. Diabetes Obes Metab. 2021 Oct;23(10):2385-2394. doi: 10.1111/dom.14481. Epub 2021 Jul 27. PMID: 34212465; PMCID: PMC8429240.

803: Calderon-Ticona JR, Taype-Rondan A, Villamonte G, Labán-Seminario LM,

Helguero-Santín LM, Miranda JJ, Lazo-Porras M. Diabetes care quality according to facility setting: A cross-sectional analysis in six Peruvian regions. Prim Care Diabetes. 2021 Jun;15(3):488-494. doi: 10.1016/j.pcd.2020.11.014. Epub 2020 Dec 24. PMID: 33358034.

804: Wade AN, Crowther NJ, Abrahams-Gessel S, Berkman L, George JA, Gómez-Olivé FX, Manne-Goehler J, Salomon JA, Wagner RG, Gaziano TA, Tollman SM, Cappola AR. Concordance between fasting plasma glucose and HbA_{1c} in the diagnosis of diabetes in black South African adults: a cross-sectional study. BMJ Open. 2021 Jun 17;11(6):e046060. doi: 10.1136/bmjopen-2020-046060. PMID: 34140342; PMCID: PMC8212405.

805: Pastor-Ibáñez R, Blanco-Heredia J, Etcheverry F, Sánchez-Palomino S, Díez-Fuertes F, Casas R, Navarrete-Muñoz MÁ, Castro-Barquero S, Lucero C, Fernández I, Leal L, Benito JM, Noguera-Julian M, Paredes R, Rallón N, Estruch R, Torrents D, García F. Adherence to a Supplemented Mediterranean Diet Drives Changes in the Gut Microbiota of HIV-1-Infected Individuals. Nutrients. 2021 Mar 30;13(4):1141. doi: 10.3390/nu13041141. PMID: 33808476; PMCID: PMC8067262.

806: McLennan SL, Henry A, Roberts LM, Siritharan SS, Ojurovic M, Yao A, Davis GK, Mangos G, Pettit F, Brown MA, O'Sullivan AJ. Maternal Adiposity and Energy Balance After Normotensive and Preeclamptic Pregnancies. J Clin Endocrinol Metab. 2021 Jul 13;106(8):e2941-e2952. doi: 10.1210/clinem/dgab223. PMID: 33824990; PMCID: PMC8277202.

807: Wiebe N, Ye F, Crumley ET, Bello A, Stenvinkel P, Tonelli M. Temporal Associations Among Body Mass Index, Fasting Insulin, and Systemic Inflammation: A Systematic Review and Meta-analysis. JAMA Netw Open. 2021 Mar 1;4(3):e211263. doi: 10.1001/jamanetworkopen.2021.1263. PMID: 33710289; PMCID: PMC7955272.

808: El-Sharkawy AM, Daliya P, Lewis-Lloyd C, Adiamah A, Malcolm FL, Boyd-Carson H, Couch D, Herrod PJJ, Hossain T, Couch J, Sarmah PB, Sian TS, Lobo DN; FaST Audit Group; East Midlands Surgical Academic Network (EMSAN). Fasting and surgery timing (FaST) audit. Clin Nutr. 2021 Mar;40(3):1405-1412. doi: 10.1016/j.clnu.2020.08.033. Epub 2020 Sep 5. PMID: 32933783; PMCID: PMC7957363.

809: Bi J, Song L, Wang L, Wu M, Chen S, Wang Y, Wu S, Tian Y. Visit-to-visit fasting blood glucose variability and lifetime risk of cardiovascular disease: a prospective study. Cardiovasc Diabetol. 2021 Oct 16;20(1):207. doi: 10.1186/s12933-021-01397-1. PMID: 34656122; PMCID: PMC8520235.

810: Shauly-Aharonov M, Shafrir A, Paltiel O, Calderon-Margalit R, Safadi R, Bicher R, Barenholz-Goultschin O, Stokar J. Both high and low pre-infection glucose levels associated with increased risk for severe COVID-19: New insights from a population-based study. PLoS One. 2021 Jul 22;16(7):e0254847. doi: 10.1371/journal.pone.0254847. PMID: 34293038; PMCID: PMC8297851.

811: van den Hoek AM, Verschuren L, Caspers MPM, Worms N, Menke AL, Princen HMG. Beneficial effects of elafibranor on NASH in E3L.CETP mice and differences

between mice and men. Sci Rep. 2021 Mar 3;11(1):5050. doi: 10.1038/s41598-021-83974-8. PMID: 33658534; PMCID: PMC7930243.

812: Robbins SR, Melo LRS, Urban H, Deveza LA, Asher R, Johnson VL, Hunter DJ. Effectiveness of Stepped-Care Intervention in Overweight and Obese Patients With Medial Tibiofemoral Osteoarthritis: A Randomized Controlled Trial. Arthritis Care Res (Hoboken). 2021 Apr;73(4):520-530. doi: 10.1002/acr.24148. PMID: 31961489.

813: Westeneng HJ, van Veenhuijzen K, van der Spek RA, Peters S, Visser AE, van Rheenen W, Veldink JH, van den Berg LH. Associations between lifestyle and amyotrophic lateral sclerosis stratified by C9orf72 genotype: a longitudinal, population-based, case-control study. Lancet Neurol. 2021 May;20(5):373-384. doi: 10.1016/S1474-4422(21)00042-9. PMID: 33894192.

814: Kato H, Ikeda N, Sugiyama T, Nomura M, Yoshita K, Nishi N. [Use of simulation models in health economic evaluation studies of dietary salt-reduction policies for cardiovascular disease prevention]. Nihon Koshu Eisei Zasshi. 2021 Sep 7;68(9):631-643. Japanese. doi: 10.11236/jph.20-150. Epub 2021 Jul 15. PMID: 34261839.

815: Beckman M, Harris J. Understanding individual and socio-cultural factors associated with hispanic parents' provision of sugar-sweetened beverages to young children. Appetite. 2021 Jun 1;161:105139. doi: 10.1016/j.appet.2021.105139. Epub 2021 Jan 26. PMID: 33513416.

816: Jahrami HA, Faris ME, I Janahi A, I Janahi M, Abdelrahim DN, Madkour MI, Sater MS, Hassan AB, Bahammam AS. Does four-week consecutive, dawn-to-sunset intermittent fasting during Ramadan affect cardiometabolic risk factors in healthy adults? A systematic review, meta-analysis, and meta-regression. Nutr Metab Cardiovasc Dis. 2021 Jul 22;31(8):2273-2301. doi: 10.1016/j.numecd.2021.05.002. Epub 2021 May 25. PMID: 34167865.

817: McCue A, Munten S, Herzig KH, Gagnon DD. Metabolic flexibility is unimpaired during exercise in the cold following acute glucose ingestion in young healthy adults. J Therm Biol. 2021 May;98:102912. doi: 10.1016/j.jtherbio.2021.102912. Epub 2021 Mar 17. PMID: 34016339.

818: Mazidi M, Kengne AP, George ES, Siervo M. The association of red meat intake with inflammation and circulating intermediate biomarkers of type 2 diabetes is mediated by central adiposity. Br J Nutr. 2021 May 14;125(9):1043-1050. doi: 10.1017/S0007114519002149. Epub 2019 Aug 22. PMID: 31434580.

819: Shah S, Locca A, Dorsett Y, Cantoni C, Ghezzi L, Lin Q, Bokoliya S, Panier H, Suther C, Gormley M, Liu Y, Evans E, Mikesell R, Obert K, Salter A, Cross AH, Tarr PI, Lovett-Racke A, Piccio L, Zhou Y. Alterations of the gut mycobiome in patients with MS. EBioMedicine. 2021 Sep;71:103557. doi: 10.1016/j.ebiom.2021.103557. Epub 2021 Aug 26. PMID: 34455391; PMCID:

PMC8399064.

820: Abbate M, Mascaró CM, Montemayor S, Barbería-Latasa M, Casares M, Gómez C, Angullo-Martinez E, Tejada S, Abete I, Zulet MA, Sureda A, Martínez JA, Tur JA. Energy Expenditure Improved Risk Factors Associated with Renal Function Loss in NAFLD and MetS Patients. Nutrients. 2021 Feb 15;13(2):629. doi: 10.3390/nu13020629. PMID: 33672073; PMCID: PMC7919687.

821: Gyeltshen T, Dorji L, Dorj L, Choden K. Outbreak of oral ulcers (glossitis) among the students of three central schools under Tsirang district Bhutan, 2018. BMC Oral Health. 2021 Sep 14;21(1):444. doi: 10.1186/s12903-021-01808-5. PMID: 34521401; PMCID: PMC8442262.

822: Mirzay Razzaz J, Moameri H, Akbarzadeh Z, Ariya M, Hosseini SA, Ghaemi A, Osati S, Ehrampoush E, Homayounfar R. Investigating the relationship between insulin resistance and adipose tissue in a randomized Tehrani population. Horm Mol Biol Clin Investig. 2021 Mar 15;42(3):235-244. doi: 10.1515/hmbci-2020-0084. PMID: 33711221.

823: Reeves MM, Terranova CO, Winkler EAH, McCarthy N, Hickman IJ, Ware RS, Lawler SP, Eakin EG, Demark-Wahnefried W. Effect of a Remotely Delivered Weight Loss Intervention in Early-Stage Breast Cancer: Randomized Controlled Trial. Nutrients. 2021 Nov 15;13(11):4091. doi: 10.3390/nu13114091. PMID: 34836345; PMCID: PMC8622393.

824: Ntsapi CM, Loos B. Neurons die with heightened but functional macro- and chaperone mediated autophagy upon increased amyloid-ß induced toxicity with region-specific protection in prolonged intermittent fasting. Exp Cell Res. 2021 Nov 15;408(2):112840. doi: 10.1016/j.yexcr.2021.112840. Epub 2021 Oct 9. PMID: 34624324.

825: Wang W, Chai Z, Cooper ME, Zimmet PZ, Guo H, Ding J, Yang F, Chen X, Lin X, Zhang K, Zhong Q, Li Z, Zhang P, Wu Z, Guan X, Zhang L, He K. High Fasting Blood Glucose Level With Unknown Prior History of Diabetes Is Associated With High Risk of Severe Adverse COVID-19 Outcome. Front Endocrinol (Lausanne). 2021 Dec 8;12:791476. doi: 10.3389/fendo.2021.791476. PMID: 34956098; PMCID: PMC8692378.

826: Lee JH, Fang C, Li X, Wu CS, Noh JY, Ye X, Chapkin RS, Sun K, Sun Y. GHS-R suppression in adipose tissues protects against obesity and insulin resistance by regulating adipose angiogenesis and fibrosis. Int J Obes (Lond). 2021 Jul;45(7):1565-1575. doi: 10.1038/s41366-021-00820-7. Epub 2021 Apr 26. PMID: 33903722; PMCID: PMC8238886.

827: Nur HA, Atoloye AT, Wengreen H, Archuleta M, Savoie-Roskos MR, Wille C, Jewkes M. A Scoping Review and Assessing the Evidence for Nutrition Education Delivery Strategies for Refugees in High-Income Countries. Adv Nutr. 2021 Dec 1;12(6):2508-2524. doi: 10.1093/advances/nmab080. PMID: 34245153; PMCID: PMC8634542.

828: Buckland NJ, Swinnerton LF, Ng K, Price M, Wilkinson LL, Myers A, Dalton M. Susceptibility to increased high energy dense sweet and savoury food intake in response to the COVID-19 lockdown: The role of craving control and acceptance coping strategies. Appetite. 2021 Mar 1;158:105017. doi: 10.1016/j.appet.2020.105017. Epub 2020 Nov 5. PMID: 33161044; PMCID: PMC8580210.

829: Mokwena MAM, Engwa GA, Nkeh-Chungag BN, Sewani-Rusike CR. Athrixia phylicoides tea infusion (bushman tea) improves adipokine balance, glucose homeostasis and lipid parameters in a diet-induced metabolic syndrome rat model. BMC Complement Med Ther. 2021 Nov 29;21(1):292. doi: 10.1186/s12906-021-03459-z. PMID: 34844584; PMCID: PMC8628465.

830: Trost SG, Byrne R, Williams KE, Johnson BJ, Bird A, Simon K, Chai LK, Terranova CO, Christian HE, Golley RK. Study protocol for Healthy Conversations @ Playgroup: a multi-site cluster randomized controlled trial of an intervention to promote healthy lifestyle behaviours in young children attending community playgroups. BMC Public Health. 2021 Sep 26;21(1):1757. doi: 10.1186/s12889-021-11789-3. PMID: 34565369; PMCID: PMC8474833.

831: Schindler V, Giezendanner S, Van Oudenhove L, Murray FR, Buehler J, Bordier V, Hente J, Pohl D. Better response to low FODMAP diet in disorders of gut-brain interaction patients with pronounced hydrogen response to a nutrient challenge test. J Gastroenterol Hepatol. 2021 Dec;36(12):3322-3328. doi: 10.1111/jgh.15573. Epub 2021 Jun 22. PMID: 34107550.

832: Huoman J, Martínez-Enguita D, Olsson E, Ernerudh J, Nilsson L, Duchén K, Gustafsson M, Jenmalm MC. Combined prenatal Lactobacillus reuteri and ω-3 supplementation synergistically modulates DNA methylation in neonatal T helper cells. Clin Epigenetics. 2021 Jun 30;13(1):135. doi: 10.1186/s13148-021-01115-4. PMID: 34193262; PMCID: PMC8247185.

833: Alqahtani S, Xia L, Jannasch A, Ferreira C, Franco J, Shannahan JH. Disruption of pulmonary resolution mediators contribute to exacerbated silver nanoparticle-induced acute inflammation in a metabolic syndrome mouse model. Toxicol Appl Pharmacol. 2021 Nov 15;431:115730. doi: 10.1016/j.taap.2021.115730. Epub 2021 Sep 30. PMID: 34601004; PMCID: PMC8545917.

834: Roudi F, Khayyatzadeh SS, Ghazizadeh H, Ferns GA, Bahrami-Taghanaki H, Mohammad-Zadeh M, Ghayour-Mobarhan M. The relationship between dietary intakes and prevalence of irritable bowel syndrome in adolescent girls: A cross-sectional study. Indian J Gastroenterol. 2021 Apr;40(2):220-226. doi: 10.1007/s12664-020-01126-8. Epub 2021 Mar 5. PMID: 33666888.

835: Asbaghi O, Ashtary-Larky D, Bagheri R, Moosavian SP, Olyaei HP, Nazarian B, Rezaei Kelishadi M, Wong A, Candow DG, Dutheil F, Suzuki K, Alavi Naeini A. Folic Acid Supplementation Improves Glycemic Control for Diabetes Prevention and Management: A Systematic Review and Dose-Response Meta-Analysis of Randomized Controlled Trials. Nutrients. 2021 Jul 9;13(7):2355. doi: 10.3390/nu13072355. PMID: 34371867; PMCID: PMC8308657.

836: Zhang Y, Tong M, Wang B, Shi Z, Wang P, Li L, Ning Y, Lu T. Geographic, Gender, and Seasonal Variation of Diabetes: A Nationwide Study With 1.4 Million Participants. J Clin Endocrinol Metab. 2021 Nov 19;106(12):e4981-e4992. doi: 10.1210/clinem/dgab543. PMID: 34314489.

837: Giontella A, Lotta LA, Overton JD, Baras A; Regeneron Genetics Center, Minuz P, Melander O, Gill D, Fava C. Causal Effect of Adiposity Measures on Blood Pressure Traits in 2 Urban Swedish Cohorts: A Mendelian Randomization Study. J Am Heart Assoc. 2021 Jul 6;10(13):e020405. doi: 10.1161/JAHA.120.020405. Epub 2021 Jun 14. PMID: 34120448; PMCID: PMC8403279.

838: Sawaya RD, Wakil C, Shayya S, Al Hariri M, Dakessian A, Wazir A, Makki M, Jamali S, Tamim H. Pediatric emergency department utilisation during Ramadan: a retrospective cross-sectional study. Arch Dis Child. 2021 Mar;106(3):272-275. doi: 10.1136/archdischild-2020-319173. Epub 2020 Sep 25. PMID: 32978143.

839: Marsh A, Radford-Smith G, Banks M, Lord A, Chachay V. Dietary intake of patients with inflammatory bowel disease aligns poorly with traditional Mediterranean diet principles. Nutr Diet. 2021 Nov 21. doi: 10.1111/1747-0080.12715. Epub ahead of print. PMID: 34806269.

840: Buja A, Grotto G, Montecchio L, De Battisti E, Sperotto M, Bertoncello C, Cocchio S, Baldovin T, Baldo V. Association between health literacy and dietary intake of sugar, fat and salt: a systematic review. Public Health Nutr. 2021 Jun;24(8):2085-2097. doi: 10.1017/S1368980020002311. Epub 2020 Aug 3. PMID: 32744216; PMCID: PMC8145460.

841: Alonso S, Tan M, Wang C, Kent S, Cobiac L, MacGregor GA, He FJ, Mihaylova B. Impact of the 2003 to 2018 Population Salt Intake Reduction Program in England: A Modeling Study. Hypertension. 2021 Apr;77(4):1086-1094. doi: 10.1161/HYPERTENSIONAHA.120.16649. Epub 2021 Mar 1. PMID: 33641370; PMCID: PMC7968966.

842: Tura A, Grespan E, Göbl CS, Koivula RW, Franks PW, Pearson ER, Walker M, Forgie IM, Giordano GN, Pavo I, Ruetten H, Dermitzakis ET, McCarthy MI, Pedersen O, Schwenk JM, Adamski J, De Masi F, Tsirigos KD, Brunak S, Viñuela A, Mahajan A, McDonald TJ, Kokkola T, Vangipurapu J, Cederberg H, Laakso M, Rutters F, Elders PJM, Koopman ADM, Beulens JW, Ridderstråle M, Hansen TH, Allin KH, Hansen T, Vestergaard H, Mari A; IMI DIRECT Consortium. Profiles of Glucose Metabolism in Different Prediabetes Phenotypes, Classified by Fasting Glycemia, 2-Hour OGTT, Glycated Hemoglobin, and 1-Hour OGTT: An IMI DIRECT Study. Diabetes. 2021 Sep;70(9):2092-2106. doi: 10.2337/db21-0227. Epub 2021 Jul 7. PMID: 34233929.

843: Smoak P, Harman N, Flores V, Kisiolek J, Pullen NA, Lisano J, Hayward R, Stewart LK. Kefir Is a Viable Exercise Recovery Beverage for Cancer Survivors Enrolled in a Structured Exercise Program. Med Sci Sports Exerc. 2021 Oct 1;53(10):2045-2053. doi: 10.1249/MSS.0000000000002690. PMID: 34519716.

844: Wood JA, Halmos EP, Taylor KM, Gibson PR. The Role of Epidemiological Evidence from Prospective Population Studies in Shaping Dietary Approaches to Therapy in Crohn's Disease. Mol Nutr Food Res. 2021 Mar;65(5):e2000294. doi: 10.1002/mnfr.202000294. Epub 2020 Oct 12. PMID: 33006435.

845: Tseng LY, Xie W, Pan W, Lyu H, Yu Z, Shi W, He Y, Chen W, Li T, Hsieh E. Validation of a six-item dietary calcium screening tool among HIV patients in China. Public Health Nutr. 2021 Oct;24(15):4786-4795. doi: 10.1017/S1368980021001427. Epub 2021 Apr 6. PMID: 33820577; PMCID: PMC8600937.

846: Schwab U, Reynolds AN, Sallinen T, Rivellese AA, Risérus U. Dietary fat intakes and cardiovascular disease risk in adults with type 2 diabetes: a systematic review and meta-analysis. Eur J Nutr. 2021 Sep;60(6):3355-3363. doi: 10.1007/s00394-021-02507-1. Epub 2021 Feb 21. PMID: 33611616.

847: Díaz-Orozco LE, Méndez-Sánchez N. Nutraceuticals and microbiota. Minerva Gastroenterol (Torino). 2021 Dec;67(4):326-338. doi: 10.23736/S2724-5985.21.02914-4. Epub 2021 May 12. PMID: 33978392.

848: Davis E, Martinez G, Blostein F, Marshall T, Jones AD, Jansen E, McNeil DW, Neiswanger K, Marazita ML, Foxman B. Dietary Patterns and Risk of a New Carious Lesion Postpartum: A Cohort Study. J Dent Res. 2021 Oct 5:220345211039478. doi: 10.1177/00220345211039478. Epub ahead of print. PMID: 34609222.

849: Keel T, Olvet DM, Cavuoto Petrizzo M, John JT, Dougherty R, Sheridan EM. Impact of an Expansion of a Clinical Nutrition Curriculum on Pre-Clerkship Medical Students' Perception of Their Knowledge and Skills Related to Performing a Nutritional Assessment. Nutrients. 2021 Nov 15;13(11):4081. doi: 10.3390/nu13114081. PMID: 34836343; PMCID: PMC8625690.

850: Breznik JA, Foley KP, Maddiboina D, Schertzer JD, Sloboda DM, Bowdish DME. Effects of Obesity-Associated Chronic Inflammation on Peripheral Blood Immunophenotype Are Not Mediated by TNF in Female C57BL/6J Mice. Immunohorizons. 2021 Jun 4;5(6):370-383. doi: 10.4049/immunohorizons.2100038. PMID: 34088851.

851: Ghimire K, Mishra SR, Satheesh G, Neupane D, Sharma A, Panda R, Kallestrup P, Mclachlan CS. Salt intake and salt-reduction strategies in South Asia: From evidence to action. J Clin Hypertens (Greenwich). 2021 Oct;23(10):1815-1829. doi: 10.1111/jch.14365. Epub 2021 Sep 9. PMID: 34498797; PMCID: PMC8678780.

852: Appannah G, Emi NA, Magendiran M, Mohd Shariff Z, Anuar Zaini A, Shamsuddin NH, Suppiah S, Mohamad Saini S, Thambiah SC, Ching SM. PUTRA-Adol study: protocol for an observational follow-up study to assess the tracking of dietary patterns linked to cardiometabolic risk factors and its prospective relationship with non-alcoholic fatty liver disease, carotid intima-medial thickness and mental well-being during adolescence in Malaysia. BMJ Open. 2021 May 25;11(5):e044747. doi: 10.1136/bmjopen-2020-044747. PMID: 34035098; PMCID: PMC8154930.

853: Lin YH. [Nutritional Care in Acute and Chronic Illness]. Hu Li Za Zhi. 2021 Jun;68(3):4-6. Chinese. doi: 10.6224/JN.202106_68(3).01. PMID: 34013499.

854: Ramírez AS, Wilson MD, Soederberg Miller LM. Segmented assimilation as a mechanism to explain the dietary acculturation paradox. Appetite. 2022 Feb 1;169:105820. doi: 10.1016/j.appet.2021.105820. Epub 2021 Nov 26. PMID: 34843752.

855: Kirschner SK, Deutz NEP, Engelen MPKJ. Intestinal dysfunction in chronic disease. Curr Opin Clin Nutr Metab Care. 2021 Sep 1;24(5):464-472. doi: 10.1097/MCO.0000000000000780. PMID: 34138768.

856: Becerra-Tomás N, Paz-Graniel I, Tresserra-Rimbau A, Martínez-González MÁ, Barrubés L, Corella D, Muñoz-Martínez J, Romaguera D, Vioque J, Alonso-Gómez ÁM, Wärnberg J, Martínez JA, Serra-Majem L, Estruch R, Bernal-López MR, Lapetra J, Pintó X, Tur JA, Garcia-Rios A, Riquelme Gallego B, Delgado-Rodríguez M, Matía-Martín P, Daimiel L, Velilla-Zancada S, Vidal J, Vázquez C, Ros E, Buil-Cosiales P, Babio N, Fernández-Carrión R, Pérez-Vega KA, Morey M, Torres-Collado L, Tojal-Sierra L, Pérez-López J, Abete I, Cabrera JP, Casas R, Fernández-García JC, Santos-Lozano JM, Esteve-Luque V, Bouzas C, Fernandez-Lazaro CI, Sorlí JV, Freixer G, Martín M, Muñoz MG, Salaverria-Lete I, Toledo E, Castañer O, Salas-Salvadó J; PREDIMED-Plus Investigators. Fruit consumption and cardiometabolic risk in the PREDIMED-plus study: A cross-sectional analysis. Nutr Metab Cardiovasc Dis. 2021 Jun 7;31(6):1702-1713. doi: 10.1016/j.numecd.2021.02.007. Epub 2021 Feb 17. PMID: 33838995.

857: Mousavi-Shirazi-Fard Z, Mazloom Z, Izadi S, Fararouei M. The effects of modified anti-inflammatory diet on fatigue, quality of life, and inflammatory biomarkers in relapsing-remitting multiple sclerosis patients: a randomized clinical trial. Int J Neurosci. 2021 Jul;131(7):657-665. doi: 10.1080/00207454.2020.1750398. Epub 2020 Apr 16. PMID: 32249637.

858: Schvey NA, Shank LM, Tanofsky-Kraff M, Ramirez S, Altman DR, Swanson T, Rubin AG, Kelly NR, LeMay-Russell S, Byrne ME, Parker MN, Broadney MM, Brady SM, Yanovski SZ, Yanovski JA. Weight-based teasing in youth: Associations with metabolic and inflammatory markers. Pediatr Obes. 2021 Mar;16(3):e12729. doi: 10.1111/ijpo.12729. Epub 2020 Oct 15. PMID: 33059389; PMCID: PMC8209784.

859: Patel YR, Imran TF, Ellison RC, Hunt SC, Carr JJ, Heiss G, Arnett DK, Pankow JS, Gaziano JM, Djoussé L. Sugar-Sweetened Beverage Consumption and Calcified Atherosclerotic Plaques in the Coronary Arteries: The NHLBI Family Heart Study. Nutrients. 2021 May 22;13(6):1775. doi: 10.3390/nu13061775. PMID: 34067500; PMCID: PMC8224703.

860: Berntson L. A pilot study of possible anti-inflammatory effects of the specific carbohydrate diet in children with juvenile idiopathic arthritis. Pediatr Rheumatol Online J. 2021 Jun 10;19(1):88. doi: 10.1186/s12969-021-00577-3. PMID: 34112181; PMCID: PMC8194161.

861: Fu X, Jia Y, Liu J, Lei Q, Li L, Li N, Hu Y, Wang S, Liu H, Yan S. The Predictive Effect of Health Examination in the Incidence of Diabetes Mellitus in Chinese Adults: A Population-Based Cohort Study. J Diabetes Res. 2021 Aug 11;2021:3552080. doi: 10.1155/2021/3552080. PMID: 34423045; PMCID: PMC8377476.

862: Schuppelius B, Peters B, Ottawa A, Pivovarova-Ramich O. Time Restricted Eating: A Dietary Strategy to Prevent and Treat Metabolic Disturbances. Front Endocrinol (Lausanne). 2021 Aug 12;12:683140. doi: 10.3389/fendo.2021.683140. PMID: 34456861; PMCID: PMC8387818.

863: Paratthakonkun C, Vimuttipong V, Nana A, Chaijenkij K, Soonthornworasiri N, Arthan D. The Effects of Crocodile Blood Supplementation on Delayed-Onset Muscle Soreness. Nutrients. 2021 Jul 5;13(7):2312. doi: 10.3390/nu13072312. PMID: 34371824; PMCID: PMC8308554.

864: Seon MJ, Hwang SY, Son Y, Song J, Kim OY. Circulating GLP-1 Levels as a Potential Indicator of Metabolic Syndrome Risk in Adult Women. Nutrients. 2021 Mar 6;13(3):865. doi: 10.3390/nu13030865. PMID: 33800785; PMCID: PMC8001839.

865: Browne J, Morey MC, Beckham JC, Bosworth HB, Porter Starr KN, Bales CW, McDermott J, Sloane R, Gregg JJ, Hall KS. Diet quality and exercise in older veterans with PTSD: a pilot study. Transl Behav Med. 2021 Dec 14;11(12):2116-2122. doi: 10.1093/tbm/ibab116. PMID: 34487181.

866: Lim SX, Loy SL, Colega MT, Lai JS, Godfrey KM, Lee YS, Tan KH, Yap F, Shek LP, Chong YS, Eriksson JG, Chan JKY, Chan SY, Chong MF. Prepregnancy adherence to plant-based diet indices and exploratory dietary patterns in relation to fecundability. Am J Clin Nutr. 2021 Oct 9:nqab344. doi: 10.1093/ajcn/nqab344. Epub ahead of print. PMID: 34626169.

867: Wuni R, Kuhnle GGC, Wynn-Jones AA, Vimaleswaran KS. A Nutrigenetic Update on CETP Gene-Diet Interactions on Lipid-Related Outcomes. Curr Atheroscler Rep. 2022 Jan 31. doi: 10.1007/s11883-022-00987-y. Epub ahead of print. PMID: 35098451.

868: Hughes RL, Davis CD, Lobach A, Holscher HD. An Overview of Current Knowledge of the Gut Microbiota and Low-Calorie Sweeteners. Nutr Today. 2021 May-Jun;56(3):105-113. doi: 10.1097/nt.0000000000000481. PMID: 34211238; PMCID: PMC8240869.

869: Mishu MP, Uphoff E, Aslam F, Philip S, Wright J, Tirbhowan N, Ajjan RA, Al Azdi Z, Stubbs B, Churchill R, Siddiqi N. Interventions for preventing type 2 diabetes in adults with mental disorders in low- and middle-income countries. Cochrane Database Syst Rev. 2021 Feb 16;2(2):CD013281. doi: 10.1002/14651858.CD013281.pub2. PMID: 33591592; PMCID: PMC8092639.

870: Peppa M, Mavroeidi I. Experimental Animal Studies Support the Role of Dietary Advanced Glycation End Products in Health and Disease. Nutrients. 2021 Sep 29;13(10):3467. doi: 10.3390/nu13103467. PMID: 34684468; PMCID: PMC8539226.

871: Milićević T, Romanić SH, Popović A, Mustać B, Đinović-Stojanović J, Jovanović G, Relić D. Human health risks and benefits assessment based on OCPs, PCBs, toxic elements and fatty acids in the pelagic fish species from the Adriatic Sea. Chemosphere. 2022 Jan;287(Pt 1):132068. doi: 10.1016/j.chemosphere.2021.132068. Epub 2021 Aug 28. PMID: 34481169.

872: Li M, Li X, Zhao Y, Zhang L, Yang J, Zhou M, Wang Z. The burden of ischemic heart disease and type 2 diabetes mellitus attributable to diet high in sugar-sweetened beverages in China: An analysis for the Global Burden of Disease Study 2017. J Diabetes. 2021 Jun;13(6):482-493. doi: 10.1111/1753-0407.13132. Epub 2020 Nov 24. PMID: 33151626.

873: Zhang Y, Zhang W, Huang J, Zhong X, Liu Y, Chen K. [Lead contamination status and assessment of potential risk to human health of commercial foods in Guangzhou City in 2017-2019]. Wei Sheng Yan Jiu. 2021 Sep;50(5):832-836. Chinese. doi: 10.19813/j.cnki.weishengyanjiu.2021.05.021. PMID: 34749880.

874: Soldevila-Domenech N, Forcano L, Vintró-Alcaraz C, Cuenca-Royo A, Pintó X, Jiménez-Murcia S, García-Gavilán JF, Nishi SK, Babio N, Gomis-González M, Corella D, Sorlí JV, Fernandez-Carrión R, Martínez-González MÁ, Marti A, Salas-Salvadó J, Castañer O, Fernández-Aranda F, Torre R. Interplay between cognition and weight reduction in individuals following a Mediterranean Diet: Three-year follow-up of the PREDIMED-Plus trial. Clin Nutr. 2021 Sep;40(9):5221-5237. doi: 10.1016/j.clnu.2021.07.020. Epub 2021 Aug 5. PMID: 34474192.

875: O'Donovan S, Palermo C, Ryan L. Competency-based assessment in nutrition education: A systematic literature review. J Hum Nutr Diet. 2022 Feb;35(1):102-111. doi: 10.1111/jhn.12946. Epub 2021 Sep 19. PMID: 34541713.

876: Hassanein M, Hussein Z, Shaltout I, Wan Seman WJ, Tong CV, Mohd Noor N, Buyukbese MA, El Tony L, Shaker GM, Alamoudi RM, Hafidh K, Fariduddin M, Batais MA, Shaikh S, Malek PR, Alabbood M, Sahay R, Alshenqete AM, Yakoob Ahmedani M. The DAR 2020 Global survey: Ramadan fasting during COVID 19 pandemic and the impact of older age on fasting among adults with Type 2 diabetes. Diabetes Res Clin Pract. 2021 Mar;173:108674. doi: 10.1016/j.diabres.2021.108674. Epub 2021 Jan 23. PMID: 33493579; PMCID: PMC7826018.

877: Guo F, Liu Y, Ding Z, Zhang Y, Zhang C, Fan J. Observations of the Effects of Maternal Fasting Plasma Glucose Changes in Early Pregnancy on Fetal Growth Profiles and Birth Outcomes. Front Endocrinol (Lausanne). 2021 Aug 19;12:666194. doi: 10.3389/fendo.2021.666194. PMID: 34489862; PMCID: PMC8417376.

878: Brown TJ, Williams H, Mafrici B, Jackson HS, Johansson L, Willingham F, McIntosh A, MacLaughlin HL. Dietary interventions with dietitian involvement in adults with chronic kidney disease: A systematic review. J Hum Nutr Diet. 2021 Aug;34(4):747-757. doi: 10.1111/jhn.12870. Epub 2021 Mar 8. PMID: 33682964.

879: Evers I, Heerings M, de Roos NM, Jongen PJ, Visser LH. Adherence to dietary

guidelines is associated with better physical and mental quality of life: results from a cross-sectional survey among 728 Dutch MS patients. Nutr Neurosci. 2021 Feb 12:1-8. doi: 10.1080/1028415X.2021.1885240. Epub ahead of print. PMID: 33573531.

880: Siddiqui NZ, Nguyen AN, Santos S, Voortman T. Diet quality and cardiometabolic health in childhood: the Generation R Study. Eur J Nutr. 2021 Sep 15. doi: 10.1007/s00394-021-02673-2. Epub ahead of print. PMID: 34528119.

881: Schade DS, Gonzales K, Kaminsky N, Adolphe A, Shey L, Eaton RP. Resolving the Egg and Cholesterol Intake Controversy: New Clinical Insights Into Cholesterol Regulation by the Liver and Intestine. Endocr Pract. 2022 Jan;28(1):102-109. doi: 10.1016/j.eprac.2021.09.004. Epub 2021 Sep 20. PMID: 34547473.

882: Mansour SR, Moustafa MAA, Saad BM, Hamed R, Moustafa AA. Impact of diet on human gut microbiome and disease risk. New Microbes New Infect. 2021 Feb 2;41:100845. doi: 10.1016/j.nmni.2021.100845. PMID: 34035924; PMCID: PMC8138677.

883: Windus JL, Burrows TL, Duncanson K, Collins CE, Rollo ME. Scoping review of nutrition intervention and dietary assessment studies in Khmer populations living in Cambodia. J Hum Nutr Diet. 2021 Dec;34(6):953-968. doi: 10.1111/jhn.12932. Epub 2021 Aug 16. PMID: 34231266.

884: Li R, Zhan W, Huang X, Zhang Z, Zhou M, Bao W, Li Q, Ma Y. Association of dietary inflammatory index and metabolic syndrome in the elderly over 55 years in Northern China. Br J Nutr. 2021 Oct 18:1-8. doi: 10.1017/S0007114521004207. Epub ahead of print. PMID: 34658314.

885: Sebastian RS, Fanelli Kuczmarski M, Wilkinson Enns C, Goldman JD, Murayi T, Moshfegh AJ, Zonderman AB, Evans MK. Application of the Database of Flavonoid Values for USDA Food Codes 2007-2010 in Assessing Intake Differences Between the Healthy Aging in Neighborhoods of Diversity across the Life Span (HANDLS) Study and What We Eat in America (WWEIA), NHANES. J Food Compost Anal. 2021 Dec;104:104124. doi: 10.1016/j.jfca.2021.104124. Epub 2021 Aug 21. PMID: 34898846; PMCID: PMC8664081.

886: Guagnano MT, D'Angelo C, Caniglia D, Di Giovanni P, Celletti E, Sabatini E, Speranza L, Bucci M, Cipollone F, Paganelli R. Improvement of Inflammation and Pain after Three Months' Exclusion Diet in Rheumatoid Arthritis Patients. Nutrients. 2021 Oct 9;13(10):3535. doi: 10.3390/nu13103535. PMID: 34684536; PMCID: PMC8539601.

887: Paillard F, Flageul O, Mahé G, Laviolle B, Dourmap C, Auffret V. Validation and reproducibility of a short food frequency questionnaire for cardiovascular prevention. Arch Cardiovasc Dis. 2021 Aug-Sep;114(8-9):570-576. doi: 10.1016/j.acvd.2020.12.008. Epub 2021 Apr 28. PMID: 33933403.

888: Andraos S, Beck KL, Jones MB, Han TL, Conlon CA, de Seymour JV.

Characterizing patterns of dietary exposure using metabolomic profiles of human biospecimens: a systematic review. Nutr Rev. 2022 Jan 12:nuab103. doi: 10.1093/nutrit/nuab103. Epub ahead of print. PMID: 35024860.

889: Sasso FC, Pafundi PC, Simeon V, De Nicola L, Chiodini P, Galiero R, Rinaldi L, Nevola R, Salvatore T, Sardu C, Marfella R, Adinolfi LE, Minutolo R; NID-2 Study Group Investigators. Efficacy and durability of multifactorial intervention on mortality and MACEs: a randomized clinical trial in type-2 diabetic kidney disease. Cardiovasc Diabetol. 2021 Jul 16;20(1):145. doi: 10.1186/s12933-021-01343-1. PMID: 34271948; PMCID: PMC8285851.

890: Davis R, Day A, Barrett J, Vanlint A, Andrews JM, Costello SP, Bryant RV. Habitual dietary fibre and prebiotic intake is inadequate in patients with inflammatory bowel disease: findings from a multicentre cross-sectional study. J Hum Nutr Diet. 2021 Apr;34(2):420-428. doi: 10.1111/jhn.12812. Epub 2020 Sep 21. PMID: 32954608.

891: Nikniaz Z, Asghari Jafarabadi M, Abbasalizad Farhangi M, Shirmohammadi M, Nikniaz L. Determinants of health-related quality of life in patients with celiac disease: a structural equation modeling. Health Qual Life Outcomes. 2021 Aug 24;19(1):204. doi: 10.1186/s12955-021-01842-5. PMID: 34429115; PMCID: PMC8386028.

892: Xu X, Yan AF, Wang Y, Shi Z. Dietary Patterns and Changes in Weight Status Among Chinese Men and Women During the COVID-19 Pandemic. Front Public Health. 2021 Dec 13;9:709535. doi: 10.3389/fpubh.2021.709535. PMID: 34966710; PMCID: PMC8710477.

893: Setayesh L, Yarizadeh H, Majidi N, Mehranfar S, Amini A, Himmerich H, Casazza K, Mirzaei K. The negative relationship of dietary inflammatory index and sleeping quality in obese and overweight women. Int J Vitam Nutr Res. 2021 Aug 4. doi: 10.1024/0300-9831/a000723. Epub ahead of print. PMID: 34344172.

894: Zhou JJ, Koska J, Bahn G, Reaven P. Fasting Glucose Variation Predicts Microvascular Risk in ACCORD and VADT. J Clin Endocrinol Metab. 2021 Mar 25;106(4):1150-1162. doi: 10.1210/clinem/dgaa941. PMID: 33367811; PMCID: PMC7993576.

895: Stege PB, Hordijk J, Shetty SA, Visser M, Viveen MC, Rogers MRC, Gijsbers E, Dierikx CM, van der Plaats RQJ, van Duijkeren E, Franz E, Willems RJL, Fuentes S, Paganelli FL. Impact of long-term dietary habits on the human gut resistome in the Dutch population. Sci Rep. 2022 Feb 3;12(1):1892. doi: 10.1038/s41598-022-05817-4. PMID: 35115599; PMCID: PMC8814023.

896: Gauci S, Young LM, White DJ, Reddan JM, Lassemillante AC, Meyer D, Pipingas A, Scholey A. Diet May Moderate the Relationship Between Arterial Stiffness and Cognitive Performance in Older Adults. J Alzheimers Dis. 2022;85(2):815-828. doi: 10.3233/JAD-210567. PMID: 34864661.

897: Xu B, Fu J, Qiao Y, Cao J, Deehan EC, Li Z, Jin M, Wang X, Wang Y. Higher intake of microbiota-accessible carbohydrates and improved cardiometabolic risk factors: a meta-analysis and umbrella review of dietary management in patients with type 2 diabetes. Am J Clin Nutr. 2021 Jun 1;113(6):1515-1530. doi: 10.1093/ajcn/nqaa435. PMID: 33693499.

898: Artegoitia VM, Krishnan S, Bonnel EL, Stephensen CB, Keim NL, Newman JW. Healthy eating index patterns in adults by sex and age predict cardiometabolic risk factors in a cross-sectional study. BMC Nutr. 2021 Jun 22;7(1):30. doi: 10.1186/s40795-021-00432-4. PMID: 34154665; PMCID: PMC8218401.

899: Ward SJ, Hill AM, Buckley JD, Banks S, Dhillon VS, Holman SL, Morrison JL, Coates AM. Minimal changes in telomere length after a 12-week dietary intervention with almonds in mid-age to older, overweight and obese Australians: results of a randomised clinical trial. Br J Nutr. 2021 May 11:1-13. doi: 10.1017/S0007114521001549. Epub ahead of print. PMID: 33971995.

900: Brennan L, Hu FB, Sun Q. Metabolomics Meets Nutritional Epidemiology: Harnessing the Potential in Metabolomics Data. Metabolites. 2021 Oct 19;11(10):709. doi: 10.3390/metabo11100709. PMID: 34677424; PMCID: PMC8537466.

901: Conley MM, McFarlane CM, Johnson DW, Kelly JT, Campbell KL, MacLaughlin HL. Interventions for weight loss in people with chronic kidney disease who are overweight or obese. Cochrane Database Syst Rev. 2021 Mar 30;3(3):CD013119. doi: 10.1002/14651858.CD013119.pub2. PMID: 33782940; PMCID: PMC8094234.

902: Schomburg L. Selenium Deficiency Due to Diet, Pregnancy, Severe Illness, or COVID-19-A Preventable Trigger for Autoimmune Disease. Int J Mol Sci. 2021 Aug 8;22(16):8532. doi: 10.3390/ijms22168532. PMID: 34445238; PMCID: PMC8395178.

903: Pereira RO, Correia LA, Farah D, Komoni G, Farah V, Fiorino P. Wistar rat as an animal model to study high-fat induced kidney damage: a systematic review. Arch Physiol Biochem. 2021 Dec 16:1-10. doi: 10.1080/13813455.2021.2017462. Epub ahead of print. PMID: 34915796.

904: Xu J, Chen X, Ma K, Nie K, Luo W, Wu X, Pan S, Wang X. Correlation Between Sleep, Life, Mood, and Diet and Severity of Inflammatory Bowel Disease in China: A Retrospective Study. Med Sci Monit. 2021 Aug 9;27:e930511. doi: 10.12659/MSM.930511. PMID: 34370718; PMCID: PMC8362338.

905: Anand S, Bose C, Kaur H, Mande SS. 'GutFeel': an in silico method for predicting gut health status based on the metabolic functional capabilities of the resident microbiome. FEBS Lett. 2021 Jul;595(13):1825-1843. doi: 10.1002/1873-3468.14107. Epub 2021 Jun 3. PMID: 33997973.

906: Becker L, Negash S, Kartschmit N, Kluttig A, Mikolajczyk R. Association between Parenthood and Health Behaviour in Later Life-Results from the Population-Based CARLA Study. Int J Environ Res Public Health. 2021 Dec 22;19(1):82. doi: 10.3390/ijerph19010082. PMID: 35010340; PMCID: PMC8751226.

907: Parker MK, Davy BM, Hedrick VE. Preliminary Assessment of the Healthy Beverage Index for US Children and Adolescents: A Tool to Quantify the Overall Beverage Intake Quality of 2- to 19-Year Olds. J Acad Nutr Diet. 2022 Feb;122(2):371-383.e6. doi: 10.1016/j.jand.2021.07.007. Epub 2021 Aug 8. PMID: 34371228.

908: Naughton F, Ward E, Khondoker M, Belderson P, Marie Minihane A, Dainty J, Hanson S, Holland R, Brown T, Notley C. Health behaviour change during the UK COVID-19 lockdown: Findings from the first wave of the C-19 health behaviour and well-being daily tracker study. Br J Health Psychol. 2021 May;26(2):624-643. doi: 10.1111/bjhp.12500. Epub 2021 Jan 6. PMID: 33410229.

909: Montanari C, Parolisi S, Borghi E, Putignani L, Bassanini G, Zuvadelli J, Bonfanti C, Tummolo A, Dionisi Vici C, Biasucci G, Burlina A, Carbone MT, Verduci E. Dysbiosis, Host Metabolism, and Non-communicable Diseases: Trialogue in the Inborn Errors of Metabolism. Front Physiol. 2021 Sep 6;12:716520. doi: 10.3389/fphys.2021.716520. PMID: 34588993; PMCID: PMC8475650.

910: Turrini A, Catasta G, Censi L, Comendador Azcarraga FJ, D'Addezio L, Ferrari M, Le Donne C, Martone D, Mistura L, Pettinelli A, Piccinelli R, Saba A, Sette S, Barbina D, Guerrera D, Carbone P, Mazzaccara A. A Dietary Assessment Training Course Path: The Italian IV SCAI Study on Children Food Consumption. Front Public Health. 2021 Mar 12;9:590315. doi: 10.3389/fpubh.2021.590315. Erratum in: Front Public Health. 2021 Jun 09;9:708291. PMID: 33777877; PMCID: PMC7994926.

911: Derks TGJ, Peeks F, de Boer F, Fokkert-Wilts M, van der Doef HPJ, van den Heuvel MC, Szymańska E, Rokicki D, Ryan PT, Weinstein DA. The potential of dietary treatment in patients with glycogen storage disease type IV. J Inherit Metab Dis. 2021 May;44(3):693-704. doi: 10.1002/jimd.12339. Epub 2020 Dec 21. PMID: 33332610; PMCID: PMC8246821.

912: Dewsbury LS, Lim CK, Steiner GZ. The Efficacy of Ketogenic Therapies in the Clinical Management of People with Neurodegenerative Disease: A Systematic Review. Adv Nutr. 2021 Jul 30;12(4):1571-1593. doi: 10.1093/advances/nmaa180. PMID: 33621313; PMCID: PMC8321843.

913: Alsulami S, Cruvinel NT, da Silva NR, Antoneli AC, Lovegrove JA, Horst MA, Vimaleswaran KS. Effect of dietary fat intake and genetic risk on glucose and insulin-related traits in Brazilian young adults. J Diabetes Metab Disord. 2021 Aug 13;20(2):1337-1347. doi: 10.1007/s40200-021-00863-7. PMID: 34900785; PMCID: PMC8630327.

914: Dodington DW, Young HE, Beaudette JR, Fritz PC, Ward WE. Improved Healing after Non-Surgical Periodontal Therapy Is Associated with Higher Protein Intake in Patients Who Are Non-Smokers. Nutrients. 2021 Oct 22;13(11):3722. doi: 10.3390/nu13113722. PMID: 34835978; PMCID: PMC8619233.

915: Miazgowski T, Kaczmarkiewicz A, Miazgowski B, Kopeć J. Cardiometabolic health, visceral fat and circulating irisin levels: results from a real-world weight loss study. J Endocrinol Invest. 2021 Jun;44(6):1243-1252. doi: 10.1007/s40618-020-01415-1. Epub 2020 Sep 6. PMID: 32892317; PMCID: PMC8124056.

916: Toh DWK, Sutanto CN, Loh WW, Lee WY, Yao Y, Ong CN, Kim JE. Skin carotenoids status as a potential surrogate marker for cardiovascular disease risk determination in middle-aged and older adults. Nutr Metab Cardiovasc Dis. 2021 Feb 8;31(2):592-601. doi: 10.1016/j.numecd.2020.10.016. Epub 2020 Oct 26. PMID: 33358716.

917: Puddu PE, Menotti A. Simple versus complex carbohydrates and health: A frequently neglected problem. Nutr Metab Cardiovasc Dis. 2021 Jun 30;31(7):1949-1952. doi: 10.1016/j.numecd.2021.03.001. Epub 2021 Mar 19. PMID: 33992508.

918: Kangalgil M, Sahinler A, Kırkbir IB, Ozcelik AO. Associations of maternal characteristics and dietary factors with anemia and iron-deficiency in pregnancy. J Gynecol Obstet Hum Reprod. 2021 Oct;50(8):102137. doi: 10.1016/j.jogoh.2021.102137. Epub 2021 Apr 7. PMID: 33838301.

919: Chen X, Huang B, Zhao Q, Wang Z, Liu W, Zhang J, Zhou Y, Sun Q, Huang H, Huang X, Jiang T, Liu J. Shellfish contamination with lipophilic toxins and dietary exposure assessments from consumption of shellfish products in Shenzhen, China. Ecotoxicol Environ Saf. 2021 Sep 15;221:112446. doi: 10.1016/j.ecoenv.2021.112446. Epub 2021 Jun 24. PMID: 34175823.

920: Carrillo A, Huffman JC, Kim S, Massey CN, Legler SR, Celano CM. An Adaptive Text Message Intervention to Promote Well-Being and Health Behavior Adherence for Patients With Cardiovascular Disease: Intervention Design and Preliminary Results. J Acad Consult Liaison Psychiatry. 2021 Nov-Dec;62(6):617-624. doi: 10.1016/j.jaclp.2021.06.001. Epub 2021 Jun 8. PMID: 34116259; PMCID: PMC8608707.

921: Zhang Y, Zhuang P, Wu F, He W, Mao L, Jia W, Zhang Y, Chen X, Jiao J. Cooking oil/fat consumption and deaths from cardiometabolic diseases and other causes: prospective analysis of 521,120 individuals. BMC Med. 2021 Apr 15;19(1):92. doi: 10.1186/s12916-021-01961-2. PMID: 33853582; PMCID: PMC8048052.

922: Gauci S, Young LM, Arnoldy L, Lassemillante AC, Scholey A, Pipingas A. Dietary patterns in middle age: effects on concurrent neurocognition and risk of age-related cognitive decline. Nutr Rev. 2021 Aug 15:nuab047. doi: 10.1093/nutrit/nuab047. Epub ahead of print. PMID: 34392373.

923: Amare H, Olsen MF, Friis H, Andersen ÅB, Abdissa A, Yilma D, Girma T, Faurholt-Jepsen D. Predictors of glucose metabolism and blood pressure among Ethiopian individuals with HIV/AIDS after one-year of antiretroviral therapy. Trop Med Int Health. 2021 Apr;26(4):428-434. doi: 10.1111/tmi.13544. Epub 2021 Feb 2. PMID: 33405245.

924: Payne Riches S, Piernas C, Aveyard P, Sheppard JP, Rayner M, Albury C, Jebb SA. A Mobile Health Salt Reduction Intervention for People With Hypertension: Results of a Feasibility Randomized Controlled Trial. JMIR Mhealth Uhealth. 2021 Oct 21;9(10):e26233. doi: 10.2196/26233. PMID: 34673535; PMCID: PMC8569539.

925: Miller S, Bruine de Bruin W, Livings M, Wilson J, Weber K, Frazzini A, Babboni M, de la Haye K. Self-reported dietary changes among Los Angeles County adults during the COVID-19 pandemic. Appetite. 2021 Nov 1;166:105586. doi: 10.1016/j.appet.2021.105586. Epub 2021 Jul 2. PMID: 34217761.

926: Czenczek-Lewandowska E, Wyszyńska J, Leszczak J, Baran J, Weres A, Mazur A, Lewandowski B. Health behaviours of young adults during the outbreak of the Covid-19 pandemic - a longitudinal study. BMC Public Health. 2021 Jun 2;21(1):1038. doi: 10.1186/s12889-021-11140-w. PMID: 34078340; PMCID: PMC8170456.

927: Sherf-Dagan S, Sinai T, Goldenshluger A, Globus I, Kessler Y, Schweiger C, Ben-Porat T. Nutritional Assessment and Preparation for Adult Bariatric Surgery Candidates: Clinical Practice. Adv Nutr. 2021 Jun 1;12(3):1020-1031. doi: 10.1093/advances/nmaa121. PMID: 33040143; PMCID: PMC8262552.

928: Sowerby LJ, Patel KB, Schmerk C, Rotenberg BW, Rocha T, Sommer DD. Effect of low salicylate diet on clinical and inflammatory markers in patients with aspirin exacerbated respiratory disease - a randomized crossover trial. J Otolaryngol Head Neck Surg. 2021 Apr 23;50(1):27. doi: 10.1186/s40463-021-00502-4. PMID: 33892819; PMCID: PMC8063291.

929: Vetrani C, Calabrese I, Di Rienzo S, Pagliuca M, Rivieccio A, De Angelis R, Riccardi G, Rivellese AA, Annuzzi G, Bozzetto L. Dietary Changes During COVID-19 Lockdown in Adults With Type 1 Diabetes on a Hybrid Artificial Pancreas. Front Public Health. 2021 Oct 27;9:752161. doi: 10.3389/fpubh.2021.752161. PMID: 34778186; PMCID: PMC8578275.

930: Wang Y, Li C, Li D, Yang H, Li X, Jin D, Xie W, Guo B. Estimated assessment of dietary exposure to artificial sweeteners from processed food in Nanjing, China. Food Addit Contam Part A Chem Anal Control Expo Risk Assess. 2021 Jul;38(7):1105-1117. doi: 10.1080/19440049.2021.1905883. Epub 2021 May 14. PMID: 33989115.

931: He J, Kong D, Yang Z, Guo R, Amponsah AE, Feng B, Zhang X, Zhang W, Liu A, Ma J, O'Brien T, Cui H. Clinical efficacy on glycemic control and safety of mesenchymal stem cells in patients with diabetes mellitus: Systematic review and meta-analysis of RCT data. PLoS One. 2021 Mar 11;16(3):e0247662. doi: 10.1371/journal.pone.0247662. PMID: 33705413; PMCID: PMC7951834.

932: Brouwer-Brolsma EM, Perenboom C, Sluik D, van de Wiel A, Geelen A, Feskens EJ, de Vries JH. Development and external validation of the 'Flower-FFQ': a FFQ designed for the Lifelines Cohort Study. Public Health Nutr. 2021 May 14:1-12. doi: 10.1017/S1368980021002111. Epub ahead of print. PMID: 33988111.

933: Llanaj E, Dejanovic GM, Valido E, Bano A, Gamba M, Kastrati L, Minder B, Stojic S, Voortman T, Marques-Vidal P, Stoyanov J, Metzger B, Glisic M, Kern H, Muka T. Effect of oat supplementation interventions on cardiovascular disease risk markers: a systematic review and meta-analysis of randomized controlled trials. Eur J Nutr. 2022 Jan 3. doi: 10.1007/s00394-021-02763-1. Epub ahead of print. PMID: 34977959.

934: Zhou Q, Wu WL, Lin CQ, Liang H, Long CY, Lv F, Pan JL, Liu ZT, Wang BY, Yang XF, Deng XL, Jiang AM. Occurrence and dietary exposure assessment of pentachlorophenol in livestock, poultry, and aquatic foods marketed in Guangdong Province, China: Based on food monitoring data from 2015 to 2018. J Food Sci. 2021 Mar;86(3):1132-1143. doi: 10.1111/1750-3841.15653. Epub 2021 Feb 18. PMID: 33598948.

935: Tsirimiagkou C, Karatzi K, Argyris A, Chalkidou F, Tzelefa V, Sfikakis PP, Yannakoulia M, Protogerou AD. Levels of dietary sodium intake: diverging associations with arterial stiffness and atheromatosis. Hellenic J Cardiol. 2021 Nov-Dec;62(6):439-446. doi: 10.1016/j.hjc.2021.02.005. Epub 2021 Feb 18. PMID: 33610752.

936: Casas R, Ribó-Coll M, Ros E, Fitó M, Lamuela-Raventos RM, Salas-Salvadó J, Zazpe I, Martínez-González MA, Sorlí JV, Estruch R, Sacanella E. Change to a healthy diet in people over 70 years old: the PREDIMED experience. Eur J Nutr. 2021 Nov 28. doi: 10.1007/s00394-021-02741-7. Epub ahead of print. PMID: 34839386.

937: Hardy I, Lloyd A, Morisset AS, Camirand Lemyre F, Baillargeon JP, Fraser WD. Healthy for My Baby Research Protocol- a Randomized Controlled Trial Assessing a Preconception Intervention to Improve the Lifestyle of Overweight Women and Their Partners. Front Public Health. 2021 Aug 3;9:670304. doi: 10.3389/fpubh.2021.670304. PMID: 34414154; PMCID: PMC8369366.

938: Qin G, Gao Y, Wen P, Liang G, Zhao P, Dong B, Tang S, Shekh K. Evaluation of the genotoxicity and teratogenicity of xylan using different model approaches. Drug Chem Toxicol. 2022 Jan;45(1):340-346. doi: 10.1080/01480545.2020.1745226. Epub 2020 Mar 31. PMID: 32228093.

939: Byrne J, Murphy C, Keogh JB, Clifton PM. The Effect of Magnesium Supplementation on Endothelial Function: A Randomised Cross-Over Pilot Study. Int J Environ Res Public Health. 2021 Aug 2;18(15):8169. doi: 10.3390/ijerph18158169. PMID: 34360460; PMCID: PMC8346147.

940: Machado V, Botelho J, Viana J, Pereira P, Lopes LB, Proença L, Delgado AS, Mendes JJ. Association between Dietary Inflammatory Index and Periodontitis: A Cross-Sectional and Mediation Analysis. Nutrients. 2021 Apr 5;13(4):1194. doi: 10.3390/nu13041194. PMID: 33916342; PMCID: PMC8066166.

941: Bonten TN, Verkleij SM, van der Kleij RM, Busch K, van den Hout WB,

Chavannes NH, Numans ME. Selective prevention of cardiovascular disease using integrated lifestyle intervention in primary care: protocol of the Healthy Heart stepped-wedge trial. BMJ Open. 2021 Jul 9;11(7):e043829. doi: 10.1136/bmjopen-2020-043829. PMID: 34244248; PMCID: PMC8273466.

942: Song P, Man Q, Li Y, Jia S, Yu D, Zhang J, Ding G. Association between Dietary Patterns and Low HDL-C among Community-Dwelling Elders in North China. Nutrients. 2021 Sep 22;13(10):3308. doi: 10.3390/nu13103308. PMID: 34684309; PMCID: PMC8537052.

943: Fang L, Li J, Yao X, Yao S, Guo Y, Zhao Y, Jin M. [Contamination status and the evaluation of dietary exposure of marine biotoxins in seafood in Ningbo City in 2017-2019]. Wei Sheng Yan Jiu. 2021 Mar;50(2):296-300. Chinese. doi: 10.19813/j.cnki.weishengyanjiu.2021.02.022. PMID: 33985640.

944: Jiang Y, Ogunade IM, Vyas D, Adesogan AT. Aflatoxin in Dairy Cows: Toxicity, Occurrence in Feedstuffs and Milk and Dietary Mitigation Strategies. Toxins (Basel). 2021 Apr 17;13(4):283. doi: 10.3390/toxins13040283. PMID: 33920591; PMCID: PMC8074160.

945: Kwasnicka D, Kale D, Schneider V, Keller J, Yeboah-Asiamah Asare B, Powell D, Naughton F, Ten Hoor GA, Verboon P, Perski O. Systematic review of ecological momentary assessment (EMA) studies of five public health-related behaviours: review protocol. BMJ Open. 2021 Jul 16;11(7):e046435. doi: 10.1136/bmjopen-2020-046435. PMID: 34272218; PMCID: PMC8287614.

946: Abdulsalam NM, Khateeb NA, Aljerbi SS, Alqumayzi WM, Balubaid SS, Almarghlani AA, Ayad AA, Williams LL. Assessment of Dietary Habits and Physical Activity Changes during the Full COVID-19 Curfew Period and Its Effect on Weight among Adults in Jeddah, Saudi Arabia. Int J Environ Res Public Health. 2021 Aug 13;18(16):8580. doi: 10.3390/ijerph18168580. PMID: 34444328; PMCID: PMC8391203.

947: Hill Gallant KM, Vorland CJ. Intestinal phosphorus absorption: recent findings in translational and clinical research. Curr Opin Nephrol Hypertens. 2021 Jul 1;30(4):404-410. doi: 10.1097/MNH.0000000000000719. PMID: 34027902; PMCID: PMC8153371.

948: Bosetti R, Tabatabai L, Naufal G, Menser T, Kash B. Comprehensive cost-effectiveness of diabetes management for the underserved in the United States: A systematic review. PLoS One. 2021 Nov 18;16(11):e0260139. doi: 10.1371/journal.pone.0260139. PMID: 34793562; PMCID: PMC8601459.

949: Cheshire WP. Salt: The paradoxical philosopher's stone of autonomic medicine. Auton Neurosci. 2021 Dec;236:102895. doi: 10.1016/j.autneu.2021.102895. Epub 2021 Oct 12. PMID: 34655930.

950: Augimeri G, Galluccio A, Caparello G, Avolio E, La Russa D, De Rose D, Morelli C, Barone I, Catalano S, Andò S, Giordano C, Sisci D, Bonofiglio D. Potential Antioxidant and Anti-Inflammatory Properties of Serum from Healthy

Adolescents with Optimal Mediterranean Diet Adherence: Findings from DIMENU Cross-Sectional Study. Antioxidants (Basel). 2021 Jul 23;10(8):1172. doi: 10.3390/antiox10081172. PMID: 34439419; PMCID: PMC8389034.

951: Vahid F, Rahmani D, Davoodi SH. The correlation between serum inflammatory, antioxidant, glucose handling biomarkers, and Dietary Antioxidant Index (DAI) and the role of DAI in obesity/overweight causation: population-based case-control study. Int J Obes (Lond). 2021 Dec;45(12):2591-2599. doi: 10.1038/s41366-021-00944-w. Epub 2021 Aug 20. PMID: 34417552.

952: Ikem A, Garth J, Wetzel J, Caldwell G. Concentration dataset of 8 selected trace elements in cultured rainbow trout (<i>Oncorhynchus mykiss</i>) and dietary exposure risks in the Missouri adult population. Data Brief. 2021 Oct 23;39:107502. doi: 10.1016/j.dib.2021.107502. PMID: 34754891; PMCID: PMC8560972.

953: Zhang H, Greenwood DC, Risch HA, Bunce D, Hardie LJ, Cade JE. Meat consumption and risk of incident dementia: cohort study of 493,888 UK Biobank participants. Am J Clin Nutr. 2021 Jul 1;114(1):175-184. doi: 10.1093/ajcn/nqab028. PMID: 33748832; PMCID: PMC8246598.

954: Amadi CN, Bocca B, Ruggieri F, Ezejiofor AN, Uzah G, Domingo JL, Rovira J, Frazzoli C, Orisakwe OE. Human dietary exposure to metals in the Niger delta region, Nigeria: Health risk assessment. Environ Res. 2022 May 1;207:112234. doi: 10.1016/j.envres.2021.112234. Epub 2021 Oct 20. PMID: 34678257.

955: Wang Y, Jiao Y, Kong Q, Zheng F, Shao L, Zhang T, Jiang D, Gao X. Occurrence of polycyclic aromatic hydrocarbons in fried and grilled fish from Shandong China and health risk assessment. Environ Sci Pollut Res Int. 2021 Feb 25. doi: 10.1007/s11356-021-13045-y. Epub ahead of print. PMID: 33630261.

956: Wu O. Microsimulation Model for Health Economic Evaluation of Public Health Policies: An Imperfect but Useful Tool. Circulation. 2021 Oct 26;144(17):1377-1379. doi: 10.1161/CIRCULATIONAHA.121.056502. Epub 2021 Oct 25. PMID: 34694892.

957: Podadera-Herreros A, Alcala-Diaz JF, Gutierrez-Mariscal FM, Jimenez-Torres J, Cruz-Ares S, Arenas-de Larriva AP, Cardelo MP, Torres-Peña JD, Luque RM, Ordovas JM, Delgado-Lista J, Lopez-Miranda J, Yubero-Serrano EM. Long-term consumption of a mediterranean diet or a low-fat diet on kidney function in coronary heart disease patients: The CORDIOPREV randomized controlled trial. Clin Nutr. 2022 Feb;41(2):552-559. doi: 10.1016/j.clnu.2021.12.041. Epub 2022 Jan 6. PMID: 35030530.

958: Deszczyńska K, Górska R, Haładyj A. Clinical condition of the oral cavity in overweight and obese patients. Dent Med Probl. 2021 Apr-Jun;58(2):147-154. doi: 10.17219/dmp/127873. PMID: 34062055.

959: Chotivichien S, Chongchaithet N, Aksornchu P, Boonmongkol N, Duangmusik P, Knowles J, Sinawat S. Assessment of the contribution of industrially processed

foods to salt and iodine intake in Thailand. PLoS One. 2021 Jul 6;16(7):e0253590. doi: 10.1371/journal.pone.0253590. PMID: 34228736; PMCID: PMC8259997.

960: Capra ME, Pederiva C, Viggiano C, De Santis R, Banderali G, Biasucci G. Nutritional Approach to Prevention and Treatment of Cardiovascular Disease in Childhood. Nutrients. 2021 Jul 10;13(7):2359. doi: 10.3390/nu13072359. PMID: 34371871; PMCID: PMC8308497.

961: Gao Q, Niu Y, Wang B, Liu J, Zhao Y, Zhang J, Wang Y, Shao B. Estimation of lactating mothers' daily intakes of bisphenol A using breast milk. Environ Pollut. 2021 Oct 1;286:117545. doi: 10.1016/j.envpol.2021.117545. Epub 2021 Jun 9. PMID: 34438484.

962: Moulaei K, Sheikhtaheri A, Ghafaripour Z, Bahaadinbeigy K. The Development and Usability Assessment of an mHealth Application to Encourage Self-Care in Pregnant Women against COVID-19. J Healthc Eng. 2021 Jul 20;2021:9968451. doi: 10.1155/2021/9968451. PMID: 34336175; PMCID: PMC8292075.

963: Menon K, Cameron JD, de Courten M, de Courten B. Use of carnosine in the prevention of cardiometabolic risk factors in overweight and obese individuals: study protocol for a randomised, double-blind placebo-controlled trial. BMJ Open. 2021 May 13;11(5):e043680. doi: 10.1136/bmjopen-2020-043680. PMID: 33986049; PMCID: PMC8126302.

964: Tasevska N, Sagi-Kiss V, Palma-Duran SA, Barrett B, Chaloux M, Commins J, O'Brien DM, Johnston CS, Midthune D, Kipnis V, Freedman LS. Investigating the performance of 24-h urinary sucrose and fructose as a biomarker of total sugars intake in US participants - a controlled feeding study. Am J Clin Nutr. 2021 Aug 2;114(2):721-730. doi: 10.1093/ajcn/nqab158. Erratum in: Am J Clin Nutr. 2021 Aug 2;114(2):827. PMID: 34036321; PMCID: PMC8326031.

965: Pan XF, Huang Y, Li X, Wang Y, Ye Y, Chen H, Marklund M, Wen Y, Liu Y, Zeng H, Qi X, Yang X, Yang CX, Liu G, Gibson RA, Xu S, Yu D, Chen D, Li Y, Mei Z, Pan A, Wu JHY. Circulating fatty acids and risk of gestational diabetes mellitus: prospective analyses in China. Eur J Endocrinol. 2021 May 24;185(1):87-97. doi: 10.1530/EJE-21-0118. PMID: 33914701.

966: Porper K, Shpatz Y, Plotkin L, Pechthold RG, Talianski A, Champ CE, Furman O, Shimoni-Sebag A, Symon Z, Amit U, Hemi R, Kanety H, Mardor Y, Cohen ZR, Jan E, Genssin H, Anikster Y, Zach L, Lawrence YR. A Phase I clinical trial of dose-escalated metabolic therapy combined with concomitant radiation therapy in high-grade glioma. J Neurooncol. 2021 Jul;153(3):487-496. doi: 10.1007/s11060-021-03786-8. Epub 2021 Jun 21. PMID: 34152528.

967: de Araújo AR, Sampaio GR, da Silva LR, Portal VL, Markoski MM, de Quadros AS, Rogero MM, da Silva Torres EAF, Marcadenti A. Effects of extra virgin olive oil and pecans on plasma fatty acids in patients with stable coronary artery disease. Nutrition. 2021 Nov-Dec;91-92:111411. doi: 10.1016/j.nut.2021.111411.

Epub 2021 Jul 15. PMID: 34425320.

968: Yu D, Yang Y, Long J, Xu W, Cai Q, Wu J, Cai H, Zheng W, Shu XO. Long-term Diet Quality and Gut Microbiome Functionality: A Prospective, Shotgun Metagenomic Study among Urban Chinese Adults. Curr Dev Nutr. 2021 Apr 2;5(4):nzab026. doi: 10.1093/cdn/nzab026. PMID: 33937616; PMCID: PMC8068758.

969: Wehedy E, Shatat IF, Al Khodor S. The Human Microbiome in Chronic Kidney Disease: A Double-Edged Sword. Front Med (Lausanne). 2022 Jan 17;8:790783. doi: 10.3389/fmed.2021.790783. PMID: 35111779; PMCID: PMC8801809.

970: Salas-Huetos A, Mínguez-Alarcón L, Mitsunami M, Arvizu M, Ford JB, Souter I, Yeste M, Chavarro JE; EARTH Study Team. Paternal adherence to healthy dietary patterns in relation to sperm parameters and outcomes of assisted reproductive technologies. Fertil Steril. 2022 Feb;117(2):298-312. doi: 10.1016/j.fertnstert.2021.10.021. Epub 2021 Dec 15. PMID: 34920872; PMCID: PMC8821200.

971: Cui K, Wu X, Zhang Y, Cao J, Wei D, Xu J, Dong F, Liu X, Zheng Y. Cumulative risk assessment of dietary exposure to triazole fungicides from 13 daily-consumed foods in China. Environ Pollut. 2021 Oct 1;286:117550. doi: 10.1016/j.envpol.2021.117550. Epub 2021 Jun 9. PMID: 34126511.

972: Javaid N, Iqbal AZ, Hameeda M. NUTRITIONAL MANAGEMENT OF LIVER CIRRHOSIS AND ITS COMPLICATIONS IN HOSPITALIZED PATIENTS. Arq Gastroenterol. 2021 Apr-Jun;58(2):246-252. doi: 10.1590/S0004-2803.202100000-43. PMID: 34287534.

973: Pina A, Castelletti S. COVID-19 and Cardiovascular Disease: a Global Perspective. Curr Cardiol Rep. 2021 Aug 19;23(10):135. doi: 10.1007/s11886-021-01566-4. PMID: 34410538; PMCID: PMC8374116.

974: Kulathunga MRDL, Wijayawardena MAA, Naidu R. Heavy metal(loid)s and health risk assessment of Dambulla vegetable market in Sri Lanka. Environ Monit Assess. 2021 Mar 27;193(4):230. doi: 10.1007/s10661-021-09020-2. PMID: 33772647.

975: Calabrese EJ, Calabrese V, Dhawan G, Kapoor R, Giordano J. Hormesis and neural stem cells. Free Radic Biol Med. 2022 Jan;178:314-329. doi: 10.1016/j.freeradbiomed.2021.12.003. Epub 2021 Dec 4. PMID: 34871764.

976: Lobene AJ, Stremke ER, McCabe GP, Moe SM, Moorthi RN, Hill Gallant KM. Spot Urine Samples to Estimate Na and K Intake in Patients With Chronic Kidney Disease and Healthy Adults: A Secondary Analysis From a Controlled Feeding Study. J Ren Nutr. 2021 Nov;31(6):602-610. doi: 10.1053/j.jrn.2020.09.007. Epub 2020 Dec 9. PMID: 33309410; PMCID: PMC8187446.

977: Keser I, Cvijetić S, Ilić A, Colić Barić I, Boschiero D, Ilich JZ. Assessment of Body Composition and Dietary Intake in Nursing-Home Residents: Could Lessons Learned from the COVID-19 Pandemic Be Used to Prevent Future Casualties in Older Individuals? Nutrients. 2021 Apr 29;13(5):1510. doi:

10.3390/nu13051510. PMID: 33947099; PMCID: PMC8146998.

978: Sasanfar B, Toorang F, Maleki F, Esmaillzadeh A, Zendehdel K. Association between dietary total antioxidant capacity and breast cancer: a case-control study in a Middle Eastern country. Public Health Nutr. 2021 Apr;24(5):965-972. doi: 10.1017/S1368980019004397. Epub 2020 Apr 1. PMID: 32234094.

979: Tsiogkas SG, Grammatikopoulou MG, Gkiouras K, Zafiriou E, Papadopoulos I, Liaskos C, Dardiotis E, Sakkas LI, Bogdanos DP. Effect of <i>Crocus sativus</i> (Saffron) Intake on Top of Standard Treatment, on Disease Outcomes and Comorbidities in Patients with Rheumatic Diseases: Synthesis without Meta-Analysis (SWiM) and Level of Adherence to the CONSORT Statement for Randomized Controlled Trials Delivering Herbal Medicine Interventions. Nutrients. 2021 Nov 27;13(12):4274. doi: 10.3390/nu13124274. PMID: 34959826; PMCID: PMC8706139.

980: Farag MA, Shakour ZTA, Elmassry MM, Donia MS. Metabolites profiling reveals gut microbiome-mediated biotransformation of green tea polyphenols in the presence of N-nitrosamine as pro-oxidant. Food Chem. 2022 Mar 1;371:131147. doi: 10.1016/j.foodchem.2021.131147. Epub 2021 Sep 16. PMID: 34808759.

981: van Wissen K, Blanchard D. The 'work' of self-care for people with cardiovascular disease and prediabetes: An interpretive description. Int J Nurs Stud. 2021 Apr;116:103548. doi: 10.1016/j.ijnurstu.2020.103548. Epub 2020 Feb 12. PMID: 32143811.

982: Yu J, Cao G, Yuan S, Luo C, Yu J, Cai M. Probiotic supplements and bone health in postmenopausal women: a meta-analysis of randomised controlled trials. BMJ Open. 2021 Mar 2;11(3):e041393. doi: 10.1136/bmjopen-2020-041393. PMID: 33653743; PMCID: PMC7929795.

983: Stefler D, Landstra E, Bobak M. Household availability of dietary fats and cardiovascular disease and mortality: prospective evidence from Russia. Eur J Public Health. 2021 Oct 26;31(5):1037-1041. doi: 10.1093/eurpub/ckab128. PMID: 34329405; PMCID: PMC8565488.

984: Williams GM, Tapsell LC, O'Brien CL, Tosh SM, Barrett EM, Beck EJ. Gut microbiome responses to dietary intake of grain-based fibers with the potential to modulate markers of metabolic disease: a systematic literature review. Nutr Rev. 2021 Oct 11;79(11):1274-1292. doi: 10.1093/nutrit/nuaa128. PMID: 33369654.

985: Billich N, Maugeri I, Calligaro L, Truby H, Davidson ZE. Weight management interventions that include dietary components for young people with chronic health care needs: A systematic review. Nutr Diet. 2021 Aug 8. doi: 10.1111/1747-0080.12698. Epub ahead of print. PMID: 34369055.

986: Hanners A, Melnyk B, Volek J, Kelley MM. Ketogenic diet, African American women, and cardiovascular health: A systematic review. Worldviews Evid Based Nurs. 2022 Jan 19. doi: 10.1111/wvn.12561. Epub ahead of print. PMID: 35044076.

987: Bernier-Jean A, Wong G, Saglimbene V, Ruospo M, Palmer SC, Natale P, Garcia-Larsen V, Johnson DW, Tonelli M, Hegbrant J, Craig JC, Teixeira-Pinto A, Strippoli GFM. Dietary Potassium Intake and All-Cause Mortality in Adults Treated with Hemodialysis. Clin J Am Soc Nephrol. 2021 Dec 1;16(12):1851–61. doi: 10.2215/CJN.08360621. Epub ahead of print. PMID: 34853064; PMCID: PMC8729497.

988: Gupta K, Testa H, Greenwood T, Kostek M, Haushalter K, Kris-Etherton PM, Petersen KS. The effect of herbs and spices on risk factors for cardiometabolic diseases: a review of human clinical trials. Nutr Rev. 2021 Jun 3:nuab034. doi: 10.1093/nutrit/nuab034. Epub ahead of print. PMID: 34080628.

989: Paterson EN, Neville CE, Wallace SM, Woodside JV, Kee F, Young IS, Cruise S, McGuinness B, Maxwell AP, McKay GJ. Dietary patterns associated with renal impairment in the Northern Ireland Cohort for the Longitudinal Study of Ageing (NICOLA). Eur J Nutr. 2021 Oct;60(7):4045-4054. doi: 10.1007/s00394-021-02579-z. Epub 2021 May 7. PMID: 33959803; PMCID: PMC8437851.

990: Bodar V, Ho YL, Cho K, Gagnon D, Gaziano JM, Djoussé L. Consumption of potatoes and incidence rate of coronary artery disease: The Million Veteran Program. Clin Nutr ESPEN. 2021 Apr;42:201-205. doi: 10.1016/j.clnesp.2021.01.039. Epub 2021 Feb 10. PMID: 33745578.

991: Tsirimiagkou C, Karatzi K, Argyris A, Basdeki ED, Kaloudi P, Yannakoulia M, Protogerou AD. Dietary sodium and cardiovascular morbidity/mortality: a brief commentary on the 'J-shape hypothesis'. J Hypertens. 2021 Dec 1;39(12):2335-2343. doi: 10.1097/HJH.0000000000002953. PMID: 34326279.

992: Piekarska M, Pszczółka M, Parol D, Szewczyk P, Śliż D, Mamcarz A. Sleeping Disorders in Healthy Individuals with Different Dietary Patterns and BMI, Questionnaire Assessment. Int J Environ Res Public Health. 2021 Nov 23;18(23):12285. doi: 10.3390/ijerph182312285. PMID: 34886011; PMCID: PMC8656560.

993: Yazbeck R, Howarth GS, Kosek M, Davidson GP, Butler RN. Breath ¹³CO₂-evidence for a noninvasive biomarker to measure added refined sugar uptake. J Appl Physiol (1985). 2021 Apr 1;130(4):1025-1032. doi: 10.1152/japplphysiol.00648.2020. Epub 2021 Jan 14. PMID: 33444124.

994: Steimle A, Grant ET, Desai MS. Quantitative assay to detect bacterial glycan-degrading enzyme activities in mouse and human fecal samples. STAR Protoc. 2021 Feb 17;2(1):100326. doi: 10.1016/j.xpro.2021.100326. PMID: 33665621; PMCID: PMC7902548.

995: Burton-Freeman B, Freeman M, Zhang X, Sandhu A, Edirisinghe I. Watermelon and L-Citrulline in Cardio-Metabolic Health: Review of the Evidence 2000-2020. Curr Atheroscler Rep. 2021 Dec 11;23(12):81. doi: 10.1007/s11883-021-00978-5. PMID: 34894302.

996: Atmis V, Bülbül B, Bahşi R, Gümüşsoy M, Yalçin A, Doğan Z, Demir Ö, Erdoğan M, Atli T. Iodine concentration and prevalence of thyroid disease in older people after salt iodization in Turkey. East Mediterr Health J. 2021 Feb 25;27(2):151-158. doi: 10.26719/2021.27.2.151. PMID: 33665799.

997: Ashaolu TJ, Ashaolu JO, Adeyeye SAO. Fermentation of prebiotics by human colonic microbiota in vitro and short-chain fatty acids production: a critical review. J Appl Microbiol. 2021 Mar;130(3):677-687. doi: 10.1111/jam.14843. Epub 2020 Sep 20. PMID: 32892434.

998: Rahman KMT, Khalequzzaman M, Khan FA, Rayna SE, Samin S, Hasan M, Islam SS. Factors associated with the nutritional status of the older population in a selected area of Dhaka, Bangladesh. BMC Geriatr. 2021 Mar 5;21(1):161. doi: 10.1186/s12877-021-02068-2. PMID: 33673811; PMCID: PMC7934473.

999: Hill L, Popov J, Figueiredo M, Caputi V, Hartung E, Moshkovich M, Pai N. Protocol for a systematic review on the role of the gut microbiome in paediatric neurological disorders. Acta Neuropsychiatr. 2021 Aug;33(4):211-216. doi: 10.1017/neu.2021.8. Epub 2021 Apr 5. PMID: 33818352.

1000: Wang Y, Lebwohl B, Mehta R, Cao Y, Green PHR, Grodstein F, Jovani M, Lochhead P, Okereke OI, Sampson L, Willett WC, Sun Q, Chan AT. Long-term Intake of Gluten and Cognitive Function Among US Women. JAMA Netw Open. 2021 May 3;4(5):e2113020. doi: 10.1001/jamanetworkopen.2021.13020. PMID: 34019084; PMCID: PMC8140370.

1001: Thomsen ST, Assunção R, Afonso C, Boué G, Cardoso C, Cubadda F, Garre A, Kruisselbrink JW, Mantovani A, Pitter JG, Poulsen M, Verhagen H, Ververis E, Voet HV, Watzl B, Pires SM. Human health risk-benefit assessment of fish and other seafood: a scoping review. Crit Rev Food Sci Nutr. 2021 May 6:1-22. doi: 10.1080/10408398.2021.1915240. Epub ahead of print. PMID: 33951954.

1002: Jekl V, Brinek A, Zikmund T, Jeklova E, Kaiser J. Use of Micro-CT Imaging to Assess Ventral Mandibular Cortical Thickness and Volume in an Experimental Rodent Model With Chronic High-Phosphorus Intake. Front Vet Sci. 2021 Dec 9;8:759093. doi: 10.3389/fvets.2021.759093. PMID: 34957278; PMCID: PMC8695870.

1003: Ibsen DB, Jakobsen MU, Halkjær J, Tjønneland A, Kilpeläinen TO, Parner ET, Overvad K. Replacing Red Meat with Other Nonmeat Food Sources of Protein is Associated with a Reduced Risk of Type 2 Diabetes in a Danish Cohort of Middle-Aged Adults. J Nutr. 2021 May 11;151(5):1241-1248. doi: 10.1093/jn/nxaa448. PMID: 33693801.

1004: Kabalan M, El-Hajj M, Khachman D, Awada S, Rachidi S, Al-Hajje A, Ajrouche R. Public awareness of environmental risk factors of cancer and attitude towards its prevention among the Lebanese general population. J Prev Med Hyg. 2021 Jul 30;62(2):E466-E478. doi: 10.15167/2421-4248/jpmh2021.62.2.1974. PMID: 34604588; PMCID: PMC8451343.

1005: Chow EJ, Doody DR, Di C, Armenian SH, Baker KS, Bricker JB, Gopal AK, Hagen AM, Ketterl TG, Lee SJ, Reding KW, Schenk JM, Syrjala KL, Taylor SA, Wang G, Neuhouser ML, Mendoza JA. Feasibility of a behavioral intervention using mobile health applications to reduce cardiovascular risk factors in cancer survivors: a pilot randomized controlled trial. J Cancer Surviv. 2021 Aug;15(4):554-563. doi: 10.1007/s11764-020-00949-w. Epub 2020 Oct 10. PMID: 33037989; PMCID: PMC8035343.

1006: Bakhsh MA, Khawandanah J, Naaman RK, Alashmali S. The impact of COVID-19 quarantine on dietary habits and physical activity in Saudi Arabia: a cross-sectional study. BMC Public Health. 2021 Jul 30;21(1):1487. doi: 10.1186/s12889-021-11540-y. PMID: 34330241; PMCID: PMC8323088.

1007: Grootveld M. Evidence-Based Challenges to the Continued Recommendation and Use of Peroxidatively-Susceptible Polyunsaturated Fatty Acid-Rich Culinary Oils for High-Temperature Frying Practises: Experimental Revelations Focused on Toxic Aldehydic Lipid Oxidation Products. Front Nutr. 2022 Jan 5;8:711640. doi: 10.3389/fnut.2021.711640. PMID: 35071288; PMCID: PMC8769064.

1008: Di Lorenzo L, Vimercati L, Pipoli A, Manghisi NM, Lampignano L, Caputi A, De Maria L, Zupo R, De Pergola G. Interplay Between Adherence to the Mediterranean Diet and Lipid Profile: A Comparative Survey Between Day-Time Healthcare and Non-healthcare Female Workers. Front Public Health. 2021 Nov 4;9:649760. doi: 10.3389/fpubh.2021.649760. PMID: 34805058; PMCID: PMC8599123.

1009: Schiborn C, Kühn T, Mühlenbruch K, Kuxhaus O, Weikert C, Fritsche A, Kaaks R, Schulze MB. A newly developed and externally validated non-clinical score accurately predicts 10-year cardiovascular disease risk in the general adult population. Sci Rep. 2021 Oct 4;11(1):19609. doi: 10.1038/s41598-021-99103-4. PMID: 34608230; PMCID: PMC8490374.

1010: LaBarre JL, Singer K, Burant CF. Advantages of Studying the Metabolome in Response to Mixed-Macronutrient Challenges and Suggestions for Future Research Designs. J Nutr. 2021 Oct 1;151(10):2868-2881. doi: 10.1093/jn/nxab223. PMID: 34255076; PMCID: PMC8681069.

1011: Ruth KS, Day FR, Hussain J, Martínez-Marchal A, Aiken CE, Azad A, Thompson DJ, Knoblochova L, Abe H, Tarry-Adkins JL, Gonzalez JM, Fontanillas P, Claringbould A, Bakker OB, Sulem P, Walters RG, Terao C, Turon S, Horikoshi M, Lin K, Onland-Moret NC, Sankar A, Hertz EPT, Timshel PN, Shukla V, Borup R, Olsen KW, Aguilera P, Ferrer-Roda M, Huang Y, Stankovic S, Timmers PRHJ, Ahearn TU, Alizadeh BZ, Naderi E, Andrulis IL, Arnold AM, Aronson KJ, Augustinsson A, Bandinelli S, Barbieri CM, Beaumont RN, Becher H, Beckmann MW, Benonisdottir S, Bergmann S, Bochud M, Boerwinkle E, Bojesen SE, Bolla MK, Boomsma DI, Bowker N, Brody JA, Broer L, Buring JE, Campbell A, Campbell H, Castelao JE, Catamo E, Chanock SJ, Chenevix-Trench G, Ciullo M, Corre T, Couch FJ, Cox A, Crisponi L, Cross SS, Cucca F, Czene K, Smith GD, de Geus EJCN, de Mutsert R, De Vivo I, Demerath EW, Dennis J, Dunning AM, Dwek M, Eriksson M, Esko T, Fasching PA, Faul JD, Ferrucci L, Franceschini N, Frayling TM, Gago-Dominguez M, Mezzavilla M,

García-Closas M, Gieger C, Giles GG, Grallert H, Gudbjartsson DF, Gudnason V, Guénel P, Haiman CA, Håkansson N, Hall P, Hayward C, He C, He W, Heiss G, Høffding MK, Hopper JL, Hottenga JJ, Hu F, Hunter D, Ikram MA, Jackson RD, Joaquim MDR, John EM, Joshi PK, Karasik D, Kardia SLR, Kartsonaki C, Karlsson R, Kitahara CM, Kolcic I, Kooperberg C, Kraft P, Kurian AW, Kutalik Z, La Bianca M, LaChance G, Langenberg C, Launer LJ, Laven JSE, Lawlor DA, Le Marchand L, Li J, Lindblom A, Lindstrom S, Lindstrom T, Linet M, Liu Y, Liu S, Luan J, Mägi R, Magnusson PKE, Mangino M, Mannermaa A, Marco B, Marten J, Martin NG, Mbarek H, McKnight B, Medland SE, Meisinger C, Meitinger T, Menni C, Metspalu A, Milani L, Milne RL, Montgomery GW, Mook-Kanamori DO, Mulas A, Mulligan AM, Murray A, Nalls MA, Newman A, Noordam R, Nutile T, Nyholt DR, Olshan AF, Olsson H, Painter JN, Patel AV, Pedersen NL, Perjakova N, Peters A, Peters U, Pharoah PDP, Polasek O, Porcu E, Psaty BM, Rahman I, Rennert G, Rennert HS, Ridker PM, Ring SM, Robino A, Rose LM, Rosendaal FR, Rossouw J, Rudan I, Rueedi R, Ruggiero D, Sala CF, Saloustros E, Sandler DP, Sanna S, Sawyer EJ, Sarnowski C, Schlessinger D, Schmidt MK, Schoemaker MJ, Schraut KE, Scott C, Shekari S, Shrikhande A, Smith AV, Smith BH, Smith JA, Sorice R, Southey MC, Spector TD, Spinelli JJ, Stampfer M, Stöckl D, van Meurs JBJ, Strauch K, Styrkarsdottir U, Swerdlow AJ, Tanaka T, Teras LR, Teumer A, Þorsteinsdottir U, Timpson NJ, Toniolo D, Traglia M, Troester MA, Truong T, Tyrrell J, Uitterlinden AG, Ulivi S, Vachon CM, Vitart V, Völker U, Vollenweider P, Völzke H, Wang Q, Wareham NJ, Weinberg CR, Weir DR, Wilcox AN, van Dijk KW, Willemsen G, Wilson JF, Wolffenbuttel BHR, Wolk A, Wood AR, Zhao W, Zygmunt M; Biobank-based Integrative Omics Study (BIOS) Consortium; eQTLGen Consortium; Biobank Japan Project; China Kadoorie Biobank Collaborative Group; kConFab Investigators; LifeLines Cohort Study; InterAct consortium; 23andMe Research Team, Chen Z, Li L, Franke L, Burgess S, Deelen P, Pers TH, Grøndahl ML, Andersen CY, Pujol A, Lopez-Contreras AJ, Daniel JA, Stefansson K, Chang-Claude J, van der Schouw YT, Lunetta KL, Chasman DI, Easton DF, Visser JA, Ozanne SE, Namekawa SH, Solc P, Murabito JM, Ong KK, Hoffmann ER, Murray A, Roig I, Perry JRB. Genetic insights into biological mechanisms governing human ovarian ageing. Nature. 2021 Aug;596(7872):393-397. doi: 10.1038/s41586-021-03779-7. Epub 2021 Aug 4. PMID: 34349265; PMCID: PMC7611832.

1012: Lu L, Chen C, Li Y, Guo W, Zhang S, Brockman J, Shikany JM, Kahe K. Magnesium intake is inversely associated with risk of non-alcoholic fatty liver disease among American adults. Eur J Nutr. 2021 Nov 6. doi: 10.1007/s00394-021-02732-8. Epub ahead of print. PMID: 34741649.

1013: Kim H, Lichtenstein AH, White K, Wong KE, Miller ER 3rd, Coresh J, Appel LJ, Rebholz CM. Plasma Metabolites Associated with a Protein-Rich Dietary Pattern: Results from the OmniHeart Trial. Mol Nutr Food Res. 2022 Jan 26:e2100890. doi: 10.1002/mnfr.202100890. Epub ahead of print. PMID: 35081272.

1014: Hasan S, Faragallah A, Shanableh SD, Alebrahem SQ. Assessing the need for native language in pharmacy education and practice: a survey in the UAE. Int J Pharm Pract. 2021 Mar 17;29(2):170-177. doi: 10.1093/ijpp/riaa006. PMID: 33729529.

1015: Sugita C, Yamashita A, Tsutsumi S, Kai H, Sonoda T, Yoshida H, Yamamoto R,

Asada Y, Kurokawa M. Brazilian propolis (AF-08) inhibits collagen-induced platelet aggregation without affecting blood coagulation. J Nat Med. 2021 Sep;75(4):975-984. doi: 10.1007/s11418-021-01518-w. Epub 2021 May 4. PMID: 33945121.

1016: Egnell M, Seconda L, Neal B, Mhurchu CN, Rayner M, Jones A, Touvier M, Kesse-Guyot E, Hercberg S, Julia C. Prospective associations of the original Food Standards Agency nutrient profiling system and three variants with weight gain, overweight and obesity risk: results from the French NutriNet-Santé cohort. Br J Nutr. 2021 Apr 28;125(8):902-914. doi: 10.1017/S0007114520003384. Epub 2020 Sep 3. PMID: 32878658.

1017: Liu C, Wu S, Pan X. Clustering of cardio-metabolic risk factors and pre-diabetes among U.S. adolescents. Sci Rep. 2021 Mar 3;11(1):5015. doi: 10.1038/s41598-021-84128-6. PMID: 33658537; PMCID: PMC7930049.

1018: Banerjee S, Radak T, Khubchandani J, Dunn P. Food Insecurity and Mortality in American Adults: Results From the NHANES-Linked Mortality Study. Health Promot Pract. 2021 Mar;22(2):204-214. doi: 10.1177/1524839920945927. Epub 2020 Aug 4. PMID: 32748673.

1019: Mutsekwa RN, Edwards JT, Angus RL. Exclusive enteral nutrition in the management of Crohn's disease: a qualitative exploration of experiences, challenges and enablers in adult patients. J Hum Nutr Diet. 2021 Apr;34(2):440-449. doi: 10.1111/jhn.12829. Epub 2020 Oct 22. PMID: 33089552.

1020: Kazemian E, Akbari ME, Moradi N, Gharibzadeh S, Amouzegar A, Rozek LS, Mondul AM, Khademolmele M, Zarins KR, Ghodoosi N, Shateri Z, Fallah S, Davoodi SH. Assessment the effect of vitamin D supplementation on plasma vitamin D levels, inflammation, and oxidative stress biomarkers based on vitamin D receptor genetic variation in breast cancer survivors: a protocol for clinical trial. J Health Popul Nutr. 2021 Nov 2;40(1):46. doi: 10.1186/s41043-021-00272-9. PMID: 34727991; PMCID: PMC8561968.

1021: Aghapour S, Bina B, Tarrahi MJ, Amiri F, Ebrahimi A. Comparative health risk assessment of nitrate in drinking groundwater resources of urban and rural regions (Isfahan, Iran), using GIS. Environ Monit Assess. 2021 Nov 12;193(12):794. doi: 10.1007/s10661-021-09575-0. PMID: 34767107.

1022: Duijster J, Mughini-Gras L, Neefjes J, Franz E. Occupational exposure and risk of colon cancer: a nationwide registry study with emphasis on occupational exposure to zoonotic gastrointestinal pathogens. BMJ Open. 2021 Aug 10;11(8):e050611. doi: 10.1136/bmjopen-2021-050611. PMID: 34376453; PMCID: PMC8356182.

1023: Tye CB, Gardner PA, Dion GR, Simpson CB, Dominguez LM. Impact of Fiberoptic Endoscopic Evaluation of Swallowing Outcomes and Dysphagia Management in Neurodegenerative Diseases. Laryngoscope. 2021 Apr;131(4):726-730. doi: 10.1002/lary.28791. Epub 2020 Jun 16. PMID: 32542698.

1024: Fiolet T, Mahamat-Saleh Y, Frenoy P, Kvaskoff M, Romana Mancini F. Background exposure to polychlorinated biphenyls and all-cause, cancer-specific, and cardiovascular-specific mortality: A systematic review and meta-analysis. Environ Int. 2021 Sep;154:106663. doi: 10.1016/j.envint.2021.106663. Epub 2021 May 31. PMID: 34082240.

1025: Raj P, Louis XL, Yu L, Siow YL, Suh M, Aukema HM, Netticadan T. Saskatoon berry supplementation prevents cardiac remodeling without improving renal disease in an animal model of reno-cardiac syndrome. J Food Biochem. 2021 Oct;45(10):e13893. doi: 10.1111/jfbc.13893. Epub 2021 Aug 29. PMID: 34459008.

1026: Maasen K, Scheijen JLJM, Opperhuizen A, Stehouwer CDA, Van Greevenbroek MM, Schalkwijk CG. Quantification of dicarbonyl compounds in commonly consumed foods and drinks; presentation of a food composition database for dicarbonyls. Food Chem. 2021 Mar 1;339:128063. doi: 10.1016/j.foodchem.2020.128063. Epub 2020 Sep 15. Erratum in: Food Chem. 2021 May 15;344:128578. PMID: 33152865.

1027: Childs CE, Munblit D, Ulfman L, Gómez-Gallego C, Lehtoranta L, Recker T, Salminen S, Tiemessen M, Collado MC. Potential Biomarkers, Risk Factors and their Associations with IgE-mediated Food Allergy in Early Life: A Narrative Review. Adv Nutr. 2021 Oct 1:nmab122. doi: 10.1093/advances/nmab122. Epub ahead of print. PMID: 34596662.

1028: Moz-Christofoletti MA, Wollgast J. Sugars, Salt, Saturated Fat and Fibre Purchased through Packaged Food and Soft Drinks in Europe 2015-2018: Are We Making Progress? Nutrients. 2021 Jul 14;13(7):2416. doi: 10.3390/nu13072416. PMID: 34371927; PMCID: PMC8308506.

1029: Baranauskas MN, Altherr CA, Gruber AH, Coggan AR, Raglin JS, Gupta SK, Carter SJ. Beetroot supplementation in women enjoying exercise together (BEE SWEET): Rationale, design and methods. Contemp Clin Trials Commun. 2020 Dec 25;21:100693. doi: 10.1016/j.conctc.2020.100693. PMID: 33392416; PMCID: PMC7773568.

1030: Di Bonito P, Valerio G, Licenziati MR, Campana G, Del Giudice EM, Di Sessa A, Morandi A, Maffeis C, Chiesa C, Pacifico L, Baroni MG, Manco M. Uric acid, impaired fasting glucose and impaired glucose tolerance in youth with overweight and obesity. Nutr Metab Cardiovasc Dis. 2021 Feb 8;31(2):675-680. doi: 10.1016/j.numecd.2020.10.007. Epub 2020 Oct 13. PMID: 33272808.

1031: Odukoya JO, Odukoya JO, Ndinteh DT. Elemental measurements and health risk assessment of sub-Saharan African medicinal plants used for cardiovascular diseases' and related risk factors' treatment. J Trace Elem Med Biol. 2021 May;65:126725. doi: 10.1016/j.jtemb.2021.126725. Epub 2021 Jan 24. PMID: 33561634.

1032: Gordillo Jaramillo FX, Kim DH, Lee SH, Kwon SK, Jha R, Lee KW. Role of oregano and Citrus species-based essential oil preparation for the control of

coccidiosis in broiler chickens. J Anim Sci Biotechnol. 2021 Apr 6;12(1):47. doi: 10.1186/s40104-021-00569-z. PMID: 33820552; PMCID: PMC8022417.

1033: Moradi F, Heshmati J, Daneshzad E, Ahmadi A, Jafari T, Persad E, Fazelian S. Association between dietary satisfaction and depression, anxiety and stress in obese and overweight patients during the coronavirus pandemic. Clin Nutr ESPEN. 2021 Oct;45:399-403. doi: 10.1016/j.clnesp.2021.07.013. Epub 2021 Jul 23. PMID: 34620346; PMCID: PMC8299144.

1034: Golabi P, Paik JM, AlQahtani S, Younossi Y, Tuncer G, Younossi ZM. Burden of non-alcoholic fatty liver disease in Asia, the Middle East and North Africa: Data from Global Burden of Disease 2009-2019. J Hepatol. 2021 Oct;75(4):795-809. doi: 10.1016/j.jhep.2021.05.022. Epub 2021 May 31. PMID: 34081959.

1035: Castellani F, Manzoli L, Martellucci CA, Flacco ME, Astolfi ML, Fabiani L, Mastrantonio R, Avino P, Protano C, Vitali M. Levels of Polychlorinated Dibenzo-p-Dioxins/Furans and Polychlorinated Biphenyls in Free-Range Hen Eggs in Central Italy and Estimated Human Dietary Exposure. J Food Prot. 2021 Aug 1;84(8):1455-1462. doi: 10.4315/JFP-21-126. PMID: 33852724.

1036: Mielech A, Puścion-Jakubik A, Socha K. Assessment of the Risk of Contamination of Food for Infants and Toddlers. Nutrients. 2021 Jul 9;13(7):2358. doi: 10.3390/nu13072358. PMID: 34371868; PMCID: PMC8308760.

1037: Williams AJ, Paramsothy R, Wu N, Ghaly S, Leach S, Paramsothy S, Corte C, O'Brien C, Burke C, Wark G, Samocha-Bonet D, Lambert K, Ahlenstiel G, Wasinger V, Dutt S, Pavli P, Grimm M, Lemberg D, Connor S, Leong R, Hold G. Australia IBD Microbiome (AIM) Study: protocol for a multicentre longitudinal prospective cohort study. BMJ Open. 2021 Feb 16;11(2):e042493. doi: 10.1136/bmjopen-2020-042493. PMID: 33593778; PMCID: PMC7888320.

1038: Zhang Y, Guo X, Gao J, Wei C, Zhao S, Liu Z, Sun H, Wang J, Liu L, Li Y, Han T, Sun C. The associations of circulating common and uncommon polyunsaturated fatty acids and modification effects on dietary quality with all-cause and disease-specific mortality in NHANES 2003-2004 and 2011-2012. Ann Med. 2021 Dec;53(1):1744-1757. doi: 10.1080/07853890.2021.1937693. PMID: 34672217; PMCID: PMC8547849.

1039: Perez L, Scarcello E, Ibouraadaten S, Yakoub Y, Leinardi R, Ambroise J, Bearzatto B, Gala JL, Paquot A, Muccioli GG, Bouzin C, van den Brule S, Lison D. Dietary nanoparticles alter the composition and function of the gut microbiota in mice at dose levels relevant for human exposure. Food Chem Toxicol. 2021 Aug;154:112352. doi: 10.1016/j.fct.2021.112352. Epub 2021 Jun 18. PMID: 34153347.

1040: Khan FA, Khalequzzaman M, Hasan M, Choudhury SR, Chiang C, Aoyama A, Islam SS. Dietary salt intake and its correlates among adults in a slum area in Dhaka, Bangladesh: a cross-sectional study. Nagoya J Med Sci. 2021 Aug;83(3):589-599. doi: 10.18999/nagjms.83.3.589. PMID: 34552291; PMCID: PMC8438000.

1041: Pang YJ, Yu CQ, Guo Y, Lyu J, Li LM. [Associations of lifestyles with major chronic diseases in Chinese adults: evidence from the China Kadoorie Biobank]. Zhonghua Liu Xing Bing Xue Za Zhi. 2021 Mar 10;42(3):369-375. Chinese. doi: 10.3760/cma.j.cn112338-20210111-00024. PMID: 33618446.

1042: Fernández Miaja M, Suárez González M, Díaz Martín JJ, Jiménez Treviño S, Bousoño García CA. Análisis de la calidad de vida relacionada con la salud en pacientes celiacos [Analysis of health-related quality life in celiac patients]. Nutr Hosp. 2021 Jul 29;38(4):715-721. Spanish. doi: 10.20960/nh.03538. PMID: 34030447.

1043: Bai A, Chen A, Chen W, Luo X, Liu S, Zhang M, Liu Y, Zhang D. Study on degradation behaviour, residue distribution, and dietary risk assessment of propiconazole in celery and onion under field application. J Sci Food Agric. 2021 Mar 30;101(5):1998-2005. doi: 10.1002/jsfa.10817. Epub 2020 Oct 5. PMID: 32949153.

1044: Ahmed S, Innes JK, Calder PC. Influence of different intravenous lipid emulsions on fatty acid status and laboratory and clinical outcomes in adult patients receiving home parenteral nutrition: A systematic review. Clin Nutr. 2021 Mar;40(3):1115-1122. doi: 10.1016/j.clnu.2020.07.014. Epub 2020 Jul 23. PMID: 32758383.

1045: Kaufman-Shriqui V, Navarro DA, Raz O, Boaz M. Dietary changes and anxiety during the coronavirus pandemic: a multinational survey. Eur J Clin Nutr. 2022 Jan;76(1):84-92. doi: 10.1038/s41430-021-00897-3. Epub 2021 Mar 19. PMID: 33742156; PMCID: PMC7976683.

1046: Lampignano L, Quaranta N, Bortone I, Tirelli S, Zupo R, Castellana F, Donghia R, Guerra V, Griseta C, Pesole PL, Chieppa M, Logroscino G, Lozupone M, Cisternino AM, De Pergola G, Panza F, Giannelli G, Boeing H, Sardone R. Dietary Habits and Nutrient Intakes Are Associated to Age-Related Central Auditory Processing Disorder in a Cohort From Southern Italy. Front Aging Neurosci. 2021 May 6;13:629017. doi: 10.3389/fnagi.2021.629017. PMID: 34025388; PMCID: PMC8134698.

1047: Saxe-Custack A, Todem D, Anthony JC, Kerver JM, LaChance J, Hanna-Attisha M. Effect of a pediatric fruit and vegetable prescription program on child dietary patterns, food security, and weight status: a study protocol. BMC Public Health. 2022 Jan 21;22(1):150. doi: 10.1186/s12889-022-12544-y. PMID: 35062926; PMCID: PMC8778506.

1048: Ba DM, Gao X, Muscat J, Al-Shaar L, Chinchilli V, Zhang X, Ssentongo P, Beelman RB, Richie JP Jr. Association of mushroom consumption with all-cause and cause-specific mortality among American adults: prospective cohort study findings from NHANES III. Nutr J. 2021 Apr 22;20(1):38. doi: 10.1186/s12937-021-00691-8. PMID: 33888143; PMCID: PMC8061446.

1049: Fan JC, He HL, Ren R, Wang ST. Monochloropropanediol in edible vegetable oils from Hangzhou market in China: occurrence and exposure risk assessment. Food Addit Contam Part A Chem Anal Control Expo Risk Assess. 2021 Nov;38(11):1867-1874. doi: 10.1080/19440049.2021.1954702. Epub 2021 Jul 19. PMID: 34279183.

1050: Cairoli E, Aresta C, Giovanelli L, Eller-Vainicher C, Migliaccio S, Giannini S, Giusti A, Marcocci C, Gonnelli S, Isaia GC, Rossini M, Chiodini I, Di Stefano M; Italian Society for Osteoporosis, Mineral Metabolism, Skeletal Diseases (SIOMMMS). Dietary calcium intake in a cohort of individuals evaluated for low bone mineral density: a multicenter Italian study. Aging Clin Exp Res. 2021 Dec;33(12):3223-3235. doi: 10.1007/s40520-021-01856-5. Epub 2021 Apr 28. PMID: 33909280; PMCID: PMC8668846.

1051: Saha Turna N, Wu F. Estimation of Tolerable Daily Intake (TDI) for Immunological Effects of Aflatoxin. Risk Anal. 2021 Jun 19. doi: 10.1111/risa.13770. Epub ahead of print. PMID: 34147038.

1052: Williams MB, Wang W, Taniguchi T, Salvatore AL, Groover WK, Wetherill M, Love C, Cannady T, Grammar M, Standridge J, Fox J, Jernigan VBB. Impact of a Healthy Retail Intervention on Fruits and Vegetables and Total Sales in Tribally Owned Convenience Stores: Findings From the THRIVE Study. Health Promot Pract. 2021 Nov;22(6):796-805. doi: 10.1177/1524839920953122. Epub 2020 Sep 10. PMID: 32912007; PMCID: PMC7943643.

1053: Hughes J, Grafenauer S. Oat and Barley in the Food Supply and Use of Beta Glucan Health Claims. Nutrients. 2021 Jul 26;13(8):2556. doi: 10.3390/nu13082556. PMID: 34444720; PMCID: PMC8401220.

1054: Matthews JJ, Dolan E, Swinton PA, Santos L, Artioli GG, Turner MD, Elliott-Sale KJ, Sale C. Effect of Carnosine or β-Alanine Supplementation on Markers of Glycemic Control and Insulin Resistance in Humans and Animals: A Systematic Review and Meta-analysis. Adv Nutr. 2021 Dec 1;12(6):2216-2231. doi: 10.1093/advances/nmab087. PMID: 34333586; PMCID: PMC8634390.

1055: Deane KHO, Jimoh OF, Biswas P, O'Brien A, Hanson S, Abdelhamid AS, Fox C, Hooper L. Omega-3 and polyunsaturated fat for prevention of depression and anxiety symptoms: systematic review and meta-analysis of randomised trials. Br J Psychiatry. 2021 Mar;218(3):135-142. doi: 10.1192/bjp.2019.234. PMID: 31647041.

1056: Bailey RL, Stang JS, Davis TA, Naimi TS, Schneeman BO, Dewey KG, Donovan SM, Novotny R, Kleinman RE, Taveras EM, Bazzano L, Snetselaar LG, de Jesus J, Casavale KO, Stoody EE, Goldman JD, Moshfegh AJ, Rhodes DG, Herrick KA, Koegel K, Perrine CG, Pannucci T. Dietary and Complementary Feeding Practices of US Infants, 6 to 12 Months: A Narrative Review of the Federal Nutrition Monitoring Data. J Acad Nutr Diet. 2021 Oct 21:S2212-2672(21)01384-8. doi: 10.1016/j.jand.2021.10.017. Epub ahead of print. PMID: 34688966.

1057: Cui K, Wu X, Wei D, Zhang Y, Cao J, Xu J, Dong F, Liu X, Zheng Y. Health

risks to dietary neonicotinoids are low for Chinese residents based on an analysis of 13 daily-consumed foods. Environ Int. 2021 Apr;149:106385. doi: 10.1016/j.envint.2021.106385. Epub 2021 Jan 24. PMID: 33503555.

1058: Zhou L, Yang Y, Feng Y, Zhao X, Fan Y, Rong J, Zhao L, Yu Y. Association between dietary sodium intake and non-alcoholic fatty liver disease in the US population. Public Health Nutr. 2021 Apr;24(5):993-1000. doi: 10.1017/S136898001900483X. Epub 2020 Apr 21. PMID: 32312347.

1059: Hasani M, Mansour A, Asayesh H, Djalalinia S, Mahdavi Gorabi A, Ochi F, Qorbani M. Effect of glutamine supplementation on cardiometabolic risk factors and inflammatory markers: a systematic review and meta-analysis. BMC Cardiovasc Disord. 2021 Apr 17;21(1):190. doi: 10.1186/s12872-021-01986-8. PMID: 33865313; PMCID: PMC8053267.

1060: Rempelos L, Wang J, Barański M, Watson A, Volakakis N, Hoppe HW, Kühn-Velten WN, Hadall C, Hasanaliyeva G, Chatzidimitriou E, Magistrali A, Davis H, Vigar V, Średnicka-Tober D, Rushton S, Iversen PO, Seal CJ, Leifert C. Diet and food type affect urinary pesticide residue excretion profiles in healthy individuals: results of a randomized controlled dietary intervention trial. Am J Clin Nutr. 2021 Oct 27:nqab308. doi: 10.1093/ajcn/nqab308. Epub ahead of print. PMID: 34718382.

1061: Kris-Etherton PM, Petersen KS, Després JP, Braun L, de Ferranti SD, Furie KL, Lear SA, Lobelo F, Morris PB, Sacks FM. Special Considerations for Healthy Lifestyle Promotion Across the Life Span in Clinical Settings: A Science Advisory From the American Heart Association. Circulation. 2021 Dec 14;144(24):e515-e532. doi: 10.1161/CIR.0000000000001014. Epub 2021 Oct 25. PMID: 34689570.

1062: Ostojic SM. Safety of Dietary Guanidinoacetic Acid: A Villain of a Good Guy? Nutrients. 2021 Dec 24;14(1):75. doi: 10.3390/nu14010075. PMID: 35010949; PMCID: PMC8746922.

1063: Zhu A, Chen H, Shen J, Wang X, Li Z, Zhao A, Shi X, Yan L, Zeng Y, Yuan C, Ji JS. Interaction between plant-based dietary pattern and air pollution on cognitive function: a prospective cohort analysis of Chinese older adults. Lancet Reg Health West Pac. 2022 Jan 5;20:100372. doi: 10.1016/j.lanwpc.2021.100372. PMID: 35028630; PMCID: PMC8741490.

1064: Yoder AD, Proaño GV, Handu D. Retail Nutrition Programs and Outcomes: An Evidence Analysis Center Scoping Review. J Acad Nutr Diet. 2021 Sep;121(9):1866-1880.e4. doi: 10.1016/j.jand.2020.08.080. Epub 2020 Nov 20. PMID: 33229206.

1065: Lamb JJ, Stone M, D'Adamo CR, Volkov A, Metti D, Aronica L, Minich D, Leary M, Class M, Carullo M, Ryan JJ, Larson IA, Lundquist E, Contractor N, Eck B, Ordovas JM, Bland JS. Personalized Lifestyle Intervention and Functional Evaluation Health Outcomes SurvEy: Presentation of the LIFEHOUSE Study Using

N-of-One Tent-Umbrella-Bucket Design. J Pers Med. 2022 Jan 15;12(1):115. doi: 10.3390/jpm12010115. PMID: 35055430; PMCID: PMC8779079.

1066: Dijksterhuis GB, van Bergen G, de Wijk RA, Zandstra EH, Kaneko D, Vingerhoeds M. Exploring impact on eating behaviour, exercise and well-being during COVID-19 restrictions in the Netherlands. Appetite. 2022 Jan 1;168:105720. doi: 10.1016/j.appet.2021.105720. Epub 2021 Sep 29. PMID: 34597741; PMCID: PMC8479541.

1067: Schaffner M, Mühlberger N, Conrads-Frank A, Qerimi Rushaj V, Sroczynski G, Koukkou E, Heinsbaek Thuesen B, Völzke H, Oberaigner W, Siebert U, Rochau U. Benefits and Harms of a Prevention Program for Iodine Deficiency Disorders: Predictions of the Decision-Analytic EUthyroid Model. Thyroid. 2021 Mar;31(3):494-508. doi: 10.1089/thy.2020.0062. Epub 2020 Oct 21. PMID: 32847437.

1068: Ziaie S, Namazi N, Afzal G, Barati S, Mohebbi R, Mir M, Esmaily H, Mehralian G. Assessing multiple sclerosis-related quality of life among Iranian patients using the MSQOL-54 tool: a cross-sectional study. BMC Neurol. 2021 Aug 31;21(1):333. doi: 10.1186/s12883-021-02357-8. PMID: 34465284; PMCID: PMC8406745.

1069: Smit SE, Manirafasha C, Marais E, Johnson R, Huisamen B. Cardioprotective Function of Green Rooibos (Aspalathus linearis) Extract Supplementation in Ex Vivo Ischemic Prediabetic Rat Hearts. Planta Med. 2022 Jan;88(1):62-78. doi: 10.1055/a-1239-9236. Epub 2020 Dec 7. PMID: 33285593.

1070: Jiang D, Cheng Z, Chen X, Dong F, Xu J, Liu X, Wu X, Pan X, An X, Zheng Y. Occurrences of eight common-used pesticide adjuvants in ten vegetable species and implications for dietary intake in North China. Food Chem. 2021 Jun 15;347:128984. doi: 10.1016/j.foodchem.2020.128984. Epub 2021 Jan 6. PMID: 33503574.

1071: Ogundijo DA, Tas AA, Onarinde BA. An assessment of nutrition information on front of pack labels and healthiness of foods in the United Kingdom retail market. BMC Public Health. 2021 Feb 8;21(1):220. doi: 10.1186/s12889-021-10255-4. PMID: 33550987; PMCID: PMC7868120.

1072: Park S, Lee S, Kim Y, Lee Y, Kang MW, Kim K, Kim YC, Han SS, Lee H, Lee JP, Joo KW, Lim CS, Kim YS, Kim DK. Causal effects of relative fat, protein, and carbohydrate intake on chronic kidney disease: a Mendelian randomization study. Am J Clin Nutr. 2021 Apr 6;113(4):1023-1031. doi: 10.1093/ajcn/nqaa379. PMID: 33564816.

1073: Melzer TM, Manosso LM, Yau SY, Gil-Mohapel J, Brocardo PS. In Pursuit of Healthy Aging: Effects of Nutrition on Brain Function. Int J Mol Sci. 2021 May 10;22(9):5026. doi: 10.3390/ijms22095026. PMID: 34068525; PMCID: PMC8126018.

1074: Kim S, Park M, Song R. Effects of self-management programs on behavioral modification among individuals with chronic disease: A systematic review and

meta-analysis of randomized trials. PLoS One. 2021 Jul 23;16(7):e0254995. doi: 10.1371/journal.pone.0254995. PMID: 34297741; PMCID: PMC8301623.

1075: Duarte A, Belo O. Cardiac well-being indexes: a decision support tool to monitor cardiovascular health. J Integr Bioinform. 2021 Mar 29;18(2):127-138. doi: 10.1515/jib-2020-0040. PMID: 33770831; PMCID: PMC8238473.

1076: Mohd Yusof BN, Hasbullah FY, Mohd Shahar AS, Omar N, Abu Zaid Z, Mukhtar F, Liu RXY, Marczewska A, Hamdy O. Changes in dietary intake improve glycemic control following a structured nutrition therapy during Ramadan in individuals with type 2 diabetes. Clin Nutr ESPEN. 2021 Dec;46:314-324. doi: 10.1016/j.clnesp.2021.09.738. Epub 2021 Sep 30. PMID: 34857213.

1077: Mailloux NA, Henegan CP, Lsoto D, Patterson KP, West PC, Foley JA, Patz JA. Climate Solutions Double as Health Interventions. Int J Environ Res Public Health. 2021 Dec 18;18(24):13339. doi: 10.3390/ijerph182413339. PMID: 34948948; PMCID: PMC8705042.

1078: Borghi L, Salvatici E, Banderali G, Riva E, Giovannini M, Vegni E. Psychological wellbeing in parents of children with phenylketonuria and association with treatment adherence. Minerva Pediatr (Torino). 2021 Aug;73(4):330-339. doi: 10.23736/S2724-5276.18.05126-5. Epub 2018 Apr 12. PMID: 29651832.

1079: Padhani ZA, Moazzam Z, Ashraf A, Bilal H, Salam RA, Das JK, Bhutta ZA. Vitamin C supplementation for prevention and treatment of pneumonia. Cochrane Database Syst Rev. 2021 Nov 18;11(11):CD013134. doi: 10.1002/14651858.CD013134.pub3. PMID: 34791642; PMCID: PMC8599445.

1080: Cheng B, Chu X, Yang X, Wen Y, Jia Y, Liang C, Yao Y, Ye J, Cheng S, Liu L, Wu C, Zhang F. Dietary Habit Is Associated with Depression and Intelligence: An Observational and Genome-Wide Environmental Interaction Analysis in the UK Biobank Cohort. Nutrients. 2021 Mar 31;13(4):1150. doi: 10.3390/nu13041150. PMID: 33807197; PMCID: PMC8067152.

1081: Huang S, Qi Z, Ma S, Li G, Long C, Yu Y. A critical review on human internal exposure of phthalate metabolites and the associated health risks. Environ Pollut. 2021 Jun 15;279:116941. doi: 10.1016/j.envpol.2021.116941. Epub 2021 Mar 13. PMID: 33756240.

1082: Kaushik A, Peralta-Alvarez F, Gupta P, Bazo-Alvarez JC, Ofori S, Bobrow K, Monyeki D, Guinto RR, Baumgartner J, Mohan S. Assessing the Policy Landscape for Salt Reduction in South-East Asian and Latin American Countries - An Initiative Towards Developing an Easily Accessible, Integrated, Searchable Online Repository. Glob Heart. 2021 Jul 15;16(1):49. doi: 10.5334/gh.929. PMID: 34381671; PMCID: PMC8284507.

1083: Khazeei Tabari MA, Iranpanah A, Bahramsoltani R, Rahimi R. Flavonoids as Promising Antiviral Agents against SARS-CoV-2 Infection: A Mechanistic Review.

Molecules. 2021 Jun 25;26(13):3900. doi: 10.3390/molecules26133900. PMID: 34202374; PMCID: PMC8271800.

1084: Nartey EB, Spector J, Adu-Afarwuah S, Jones CL, Jackson A, Ohemeng A, Shah R, Koryo-Dabrah A, Kuma AB, Hyacinth HI, Steiner-Asiedu M. Nutritional perspectives on sickle cell disease in Africa: a systematic review. BMC Nutr. 2021 Mar 18;7(1):9. doi: 10.1186/s40795-021-00410-w. PMID: 33731225; PMCID: PMC7972183.

1085: Xu FF, Song J, Li YQ, Lai YF, Lin J, Pan JL, Chi HQ, Wang Y, Li ZY, Zhang GQ, Cai ZF, Liang XX, Ma AD, Tan CT, Wu WL, Yang XF. Bioaccessibility and bioavailability adjusted dietary exposure of cadmium for local residents from a high-level environmental cadmium region. J Hazard Mater. 2021 Oct 15;420:126550. doi: 10.1016/j.jhazmat.2021.126550. Epub 2021 Jul 1. PMID: 34252664.

1086: Mehmood A, Zhao L, Wang Y, Pan F, Hao S, Zhang H, Iftikhar A, Usman M. Dietary anthocyanins as potential natural modulators for the prevention and treatment of non-alcoholic fatty liver disease: A comprehensive review. Food Res Int. 2021 Apr;142:110180. doi: 10.1016/j.foodres.2021.110180. Epub 2021 Feb 2. PMID: 33773656.

1087: Jansen EC, Dolinoy D, Peterson KE, O'Brien LM, Chervin RD, Cantoral A, Tellez-Rojo MM, Solano-Gonzalez M, Goodrich J. Adolescent sleep timing and dietary patterns in relation to DNA methylation of core circadian genes: a pilot study of Mexican youth. Epigenetics. 2021 Aug;16(8):894-907. doi: 10.1080/15592294.2020.1827719. Epub 2020 Oct 4. PMID: 33016191; PMCID: PMC8331002.

1088: Kodama S, Horikawa C, Fujihara K, Hatta M, Takeda Y, Nedachi R, Kato K, Watanabe K, Sone H. Meta-analytic research of the dose-response relationship between salt intake and risk of heart failure. Hypertens Res. 2021 Jul;44(7):885-887. doi: 10.1038/s41440-021-00632-2. Epub 2021 Mar 2. Erratum in: Hypertens Res. 2021 Mar 25;: PMID: 33654246.

1089: Damen NA, Gillingham M, Hansen JG, Thornburg KL, Purnell JQ, Marshall NE. Maternal dietary fat intake during pregnancy and newborn body composition. J Perinatol. 2021 May;41(5):1007-1013. doi: 10.1038/s41372-021-00922-0. Epub 2021 Jan 28. PMID: 33510420; PMCID: PMC8119319.

1090: Wingard MC, Dalal S, Shook PL, Myers R, Connelly BA, Thewke DP, Singh M, Singh K. Deficiency of ataxia-telangiectasia mutated kinase modulates functional and biochemical parameters of the heart in response to Western-type diet. Am J Physiol Heart Circ Physiol. 2021 Jun 1;320(6):H2324-H2338. doi: 10.1152/ajpheart.00990.2020. Epub 2021 Apr 30. PMID: 33929897; PMCID: PMC8289354.

1091: Du C, Wang W, Hsiao PY, Ludy MJ, Tucker RM. Insufficient Sleep and Poor Sleep Quality Completely Mediate the Relationship between Financial Stress and Dietary Risk among Higher Education Students. Behav Sci (Basel). 2021 May

5;11(5):69. doi: 10.3390/bs11050069. PMID: 34063082; PMCID: PMC8147970.

1092: Bergwall S, Johansson A, Sonestedt E, Acosta S. High versus low-added sugar consumption for the primary prevention of cardiovascular disease. Cochrane Database Syst Rev. 2022 Jan 5;1(1):CD013320. doi: 10.1002/14651858.CD013320.pub2. PMID: 34986271; PMCID: PMC8730703.

1093: Mozaffari H, Askari M, Bellissimo N, Azadbakht L. Associations between dietary intake of B vitamins and cardiovascular risk factors in elderly men: A cross-sectional study. Int J Clin Pract. 2021 Oct;75(10):e14691. doi: 10.1111/ijcp.14691. Epub 2021 Aug 15. PMID: 34331825.

1094: Glynn H, Möller SP, Wilding H, Apputhurai P, Moore G, Knowles SR. Prevalence and Impact of Post-traumatic Stress Disorder in Gastrointestinal Conditions: A Systematic Review. Dig Dis Sci. 2021 Dec;66(12):4109-4119. doi: 10.1007/s10620-020-06798-y. Epub 2021 Jan 12. PMID: 33433790.

1095: Trieu K, Coyle DH, Afshin A, Neal B, Marklund M, Wu JHY. The estimated health impact of sodium reduction through food reformulation in Australia: A modeling study. PLoS Med. 2021 Oct 26;18(10):e1003806. doi: 10.1371/journal.pmed.1003806. PMID: 34699528; PMCID: PMC8547659.

1096: Osokpo O, Riegel B. Cultural factors influencing self-care by persons with cardiovascular disease: An integrative review. Int J Nurs Stud. 2021 Apr;116:103383. doi: 10.1016/j.ijnurstu.2019.06.014. Epub 2019 Jul 9. PMID: 31353026.

1097: Henderson AL, Colaiácovo MP. Exposure to phthalates: germline dysfunction and aneuploidy. Prenat Diagn. 2021 Apr;41(5):610-619. doi: 10.1002/pd.5921. Epub 2021 Mar 10. PMID: 33583068.

1098: Wu LY, Cheah IK, Chong JR, Chai YL, Tan JY, Hilal S, Vrooman H, Chen CP, Halliwell B, Lai MKP. Low plasma ergothioneine levels are associated with neurodegeneration and cerebrovascular disease in dementia. Free Radic Biol Med. 2021 Dec;177:201-211. doi: 10.1016/j.freeradbiomed.2021.10.019. Epub 2021 Oct 19. PMID: 34673145.

1099: Yaseen MO, Jamshaid H, Saif A, Hussain T. Immunomodulatory role and potential utility of various nutrients and dietary components in SARS-CoV-2 infection. Int J Vitam Nutr Res. 2022 Jan;92(1):35-48. doi: 10.1024/0300-9831/a000715. Epub 2021 Jun 8. PMID: 34100300.

1100: Muscat DM, Morris GM, Bell K, Cvejic E, Smith J, Jansen J, Thomas R, Bonner C, Doust J, McCaffery K. Benefits and Harms of Hypertension and High-Normal Labels: A Randomized Experiment. Circ Cardiovasc Qual Outcomes. 2021 Apr;14(4):e007160. doi: 10.1161/CIRCOUTCOMES.120.007160. Epub 2021 Apr 5. PMID: 33813855.

1101: Wang H, Mao WF, Jiang DG, Liu SJ, Zhang L. Cumulative Risk Assessment of

Exposure to Heavy Metals through Aquatic Products in China. Biomed Environ Sci. 2021 Aug 20;34(8):606-615. doi: 10.3967/bes2021.084. PMID: 34474720.

1102: Goldstein SP, Zhang F, Klasnja P, Hoover A, Wing RR, Thomas JG. Optimizing a Just-in-Time Adaptive Intervention to Improve Dietary Adherence in Behavioral Obesity Treatment: Protocol for a Microrandomized Trial. JMIR Res Protoc. 2021 Dec 6;10(12):e33568. doi: 10.2196/33568. PMID: 34874892; PMCID: PMC8691411.

1103: Soltero EG, Jáuregui A, Hernandez E, Barquera S, Jáuregui E, López-Taylor JR, Ortiz-Hernández L, Lévesque L, Lee RE. Associations between Screen-Based Activities, Physical Activity, and Dietary Habits in Mexican Schoolchildren. Int J Environ Res Public Health. 2021 Jun 24;18(13):6788. doi: 10.3390/ijerph18136788. PMID: 34202680; PMCID: PMC8297222.

1104: Maksoud R, Balinas C, Holden S, Cabanas H, Staines D, Marshall-Gradisnik S. A systematic review of nutraceutical interventions for mitochondrial dysfunctions in myalgic encephalomyelitis/chronic fatigue syndrome. J Transl Med. 2021 Feb 17;19(1):81. doi: 10.1186/s12967-021-02742-4. PMID: 33596913; PMCID: PMC7890871.

1105: Madlala HP, Steyn NP, Kalk E, Davies MA, Nyemba D, Malaba TR, Mehta U, Petro G, Boulle A, Myer L. Association between food intake and obesity in pregnant women living with and without HIV in Cape Town, South Africa: a prospective cohort study. BMC Public Health. 2021 Aug 4;21(1):1504. doi: 10.1186/s12889-021-11566-2. PMID: 34348683; PMCID: PMC8335890.

1106: Saxena S, Kumar S, Hajare SN, Gupta S, Gautam S, Ghosh SK. 'BhAVI-23'-A spice-herb based dietary infusion possessing in-vitro anti-viral potential. J Ayurveda Integr Med. 2021 Apr-Jun;12(2):312-319. doi: 10.1016/j.jaim.2020.11.005. Epub 2021 Jan 5. PMID: 33413968; PMCID: PMC7783457.

1107: Gao Y, Zhang J, Chen H, Wang Z, Hou J, Wang L. Dimethylamine enhances platelet hyperactivity in chronic kidney disease model. J Bioenerg Biomembr. 2021 Oct;53(5):585-595. doi: 10.1007/s10863-021-09913-4. Epub 2021 Jul 30. PMID: 34327565.

1108: Leandro-Merhi VA, Aquino JLB, Bertelli HD, Ramos GG, Mendes ET, Mendonça JA. Factors associated with patient weight loss and prescribed diet during hospitalization. Nutr Hosp. 2021 Jul 29;38(4):749-757. English. doi: 10.20960/nh.03249. PMID: 33966443.

1109: Mersha J, Tariku A, Gonete KA. Undernutrition and Associated Factors Among School Adolescent Girls Attending Schools in Mirab-Armachiho District, Northwest Ethiopia. Ecol Food Nutr. 2021 Jul-Aug;60(4):473-490. doi: 10.1080/03670244.2021.1872022. Epub 2021 Jan 11. PMID: 33426928.

1110: McIntyre RS, Subramaniapillai M, Shekotikhina M, Carmona NE, Lee Y, Mansur RB, Brietzke E, Fus D, Coles AS, Iacobucci M, Park C, Potts R, Amer M, Gillard J, James C, Anglin R, Surette MG. Characterizing the gut microbiota in adults

with bipolar disorder: a pilot study. Nutr Neurosci. 2021 Mar;24(3):173-180. doi: 10.1080/1028415X.2019.1612555. Epub 2019 May 28. PMID: 31132957.

1111: Hassen HY, Aerts N, Demarest S, Manzar MD, Abrams S, Bastiaens H. Validation of the Dutch-Flemish translated ABCD questionnaire to measure cardiovascular diseases knowledge and risk perception among adults. Sci Rep. 2021 Apr 26;11(1):8952. doi: 10.1038/s41598-021-88456-5. PMID: 33903718; PMCID: PMC8076268.

1112: Schuetz P, Seres D, Lobo DN, Gomes F, Kaegi-Braun N, Stanga Z. Management of disease-related malnutrition for patients being treated in hospital. Lancet. 2021 Nov 20;398(10314):1927-1938. doi: 10.1016/S0140-6736(21)01451-3. Epub 2021 Oct 14. PMID: 34656286.

1113: Viet SM, Dellarco M, Chen E, McDade T, Faustman E, Brachvogel S, Smith M, Wright R. Recommendations for Assessment of Environmental Exposures in Longitudinal Life Course Studies Such as the National Children's Study. Front Pediatr. 2021 Apr 29;9:629487. doi: 10.3389/fped.2021.629487. PMID: 33996684; PMCID: PMC8116497.

1114: Johansson HKL, Christiansen S, Draskau MK, Svingen T, Boberg J. Classical toxicity endpoints in female rats are insensitive to the human endocrine disruptors diethylstilbestrol and ketoconazole. Reprod Toxicol. 2021 Apr;101:9-17. doi: 10.1016/j.reprotox.2021.01.003. Epub 2021 Feb 8. PMID: 33571642.

1115: Yiallourou SR, Carrington MJ. Improved sleep efficiency is associated with reduced cardio-metabolic risk: Findings from the MODERN trial. J Sleep Res. 2021 Dec;30(6):e13389. doi: 10.1111/jsr.13389. Epub 2021 Jun 2. PMID: 34080247.

1116: Mirmiran P, Yuzbashian E, Rahbarinejad P, Asghari G, Azizi F. Dietary intakes of total polyphenol and its subclasses in association with the incidence of chronic kidney diseases: a prospective population-based cohort study. BMC Nephrol. 2021 Mar 10;22(1):84. doi: 10.1186/s12882-021-02286-1. PMID: 33691637; PMCID: PMC7944599.

1117: Oliveira A, Fernandes SA, Carteri RB, Tovo CV. EVALUATION OF REST ENERGY EXPENDITURE IN PATIENTS WITH NON ALCOHOLIC FATTY LIVER DISEASE. Arq Gastroenterol. 2021 Apr-Jun;58(2):157-163. doi: 10.1590/S0004-2803.202100000-27. PMID: 34190778.

1118: Tao Y, Jia C, Jing J, Zhang J, Yu P, He M, Wu J, Chen L, Zhao E. Occurrence and dietary risk assessment of 37 pesticides in wheat fields in the suburbs of Beijing, China. Food Chem. 2021 Jul 15;350:129245. doi: 10.1016/j.foodchem.2021.129245. Epub 2021 Feb 8. PMID: 33601091.

1119: de Lima TR, González-Chica DA, D'Orsi E, Sui X, Silva DAS. Individual and Combined Association Between Healthy Lifestyle Habits With Muscle Strength According to Cardiovascular Health Status in Adults and Older Adults. J Phys Act

Health. 2021 Jun 30;18(8):973-980. doi: 10.1123/jpah.2021-0105. PMID: 34193625.

1120: Kavanagh R, Cooper D, Bolton J, Keaver L. The impact of a 6-week community-based physical activity and health education intervention-a pilot study among Irish farmers. Ir J Med Sci. 2022 Feb;191(1):433-445. doi: 10.1007/s11845-021-02579-2. Epub 2021 Mar 8. PMID: 33686569.

1121: Henry Osokpo O, James R, Riegel B. Maintaining cultural identity: A systematic mixed studies review of cultural influences on the self-care of African immigrants living with non-communicable disease. J Adv Nurs. 2021 Sep;77(9):3600-3617. doi: 10.1111/jan.14804. Epub 2021 Feb 22. PMID: 33619819.

1122: Alvarado M, Penney TL, Unwin N, Murphy MM, Adams J. Evidence of a health risk 'signalling effect' following the introduction of a sugar-sweetened beverage tax. Food Policy. 2021 Jul;102:102104. doi: 10.1016/j.foodpol.2021.102104. PMID: 34404960; PMCID: PMC8346947.

1123: Abdulrahman AT, Alnagar DK. Data Analysis and Computational Methods for Assessing Knowledge of Obesity Risk Factors among Saudi Citizens. Comput Math Methods Med. 2021 Oct 26;2021:1371336. doi: 10.1155/2021/1371336. PMID: 34737785; PMCID: PMC8563112.

1124: Aaseth J, Alexander J, Alehagen U. Coenzyme Q₁₀ supplementation - In ageing and disease. Mech Ageing Dev. 2021 Jul;197:111521. doi: 10.1016/j.mad.2021.111521. Epub 2021 Jun 12. PMID: 34129891.

1125: Ng C, Major G, Smyth AR. Timing of pancreatic enzyme replacement therapy (PERT) in cystic fibrosis. Cochrane Database Syst Rev. 2021 Aug 2;8(8):CD013488. doi: 10.1002/14651858.CD013488.pub2. PMID: 34339047; PMCID: PMC8406465.

1126: Manukian G, Kivolowitz C, DeAngelis T, Shastri AA, Savage JE, Camphausen K, Rodeck U, Zarif JC, Simone NL. Caloric Restriction Impairs Regulatory T cells Within the Tumor Microenvironment After Radiation and Primes Effector T cells. Int J Radiat Oncol Biol Phys. 2021 Aug 1;110(5):1341-1349. doi: 10.1016/j.ijrobp.2021.02.029. Epub 2021 Feb 26. PMID: 33647370; PMCID: PMC8286289.

1127: Amonoo HL, Celano CM, Sadlonova M, Huffman JC. Is Optimism a Protective Factor for Cardiovascular Disease? Curr Cardiol Rep. 2021 Oct 1;23(11):158. doi: 10.1007/s11886-021-01590-4. PMID: 34599386.

1128: Krapf J, Schuhbeck A, Wendel T, Fritz J, Scholl-Bürgi S, Bösmüller C, Oberhuber R, Margreiter C, Maglione M, Stättner S, Messner F, Berchtold V, Braunwarth E, Primavesi F, Cardini B, Resch T, Karall D, Öfner D, Margreiter R, Schneeberger S. Assessment of the Clinical Impact of a Liver-Specific, BCAA-Enriched Diet in Major Liver Surgery. Transplant Proc. 2021 Mar;53(2):624-629. doi: 10.1016/j.transproceed.2020.09.013. Epub 2020 Nov 1. PMID: 33139038.

1129: Liu FH, Liu C, Gong TT, Gao S, Sun H, Jiang YT, Zhang JY, Zhang M, Gao C,

Li XY, Zhao YH, Wu QJ. Dietary Inflammatory Index and Health Outcomes: An Umbrella Review of Systematic Review and Meta-Analyses of Observational Studies. Front Nutr. 2021 May 19;8:647122. doi: 10.3389/fnut.2021.647122. PMID: 34095187; PMCID: PMC8169973.

1130: Ruys CA, van de Lagemaat M, Rotteveel J, Finken MJJ, Lafeber HN. Improving long-term health outcomes of preterm infants: how to implement the findings of nutritional intervention studies into daily clinical practice. Eur J Pediatr. 2021 Jun;180(6):1665-1673. doi: 10.1007/s00431-021-03950-2. Epub 2021 Jan 30. PMID: 33517483; PMCID: PMC8105221.

1131: Iacone R, Iaccarino Idelson P, Formisano P, Russo O, Lo Noce C, Donfrancesco C, Macchia PE, Palmieri L, Galeone D, di Lenarda A, Giampaoli S, Strazzullo P. Iodine Intake Estimated by 24 h Urine Collection in the Italian Adult Population: 2008-2012 Survey. Nutrients. 2021 May 1;13(5):1529. doi: 10.3390/nu13051529. PMID: 34062834; PMCID: PMC8147380.

1132: Zhu J, Sun L, Yang J, Fan J, Tse LA, Li Y. Genetic Predisposition to Type 2 Diabetes and Insulin Levels Is Positively Associated With Serum Urate Levels. J Clin Endocrinol Metab. 2021 Jun 16;106(7):e2547-e2556. doi: 10.1210/clinem/dgab200. PMID: 33770169.

1133: Nishi K, Kanouchi H, Tanaka A, Nakamura M, Hamada T, Mishima Y, Goto Y, Kume K, Beppu M, Hijioka H, Tabata H, Mori K, Uchino Y, Yamashiro K, Matsumura Y, Higashi Y, Makizako H, Kubozono T, Takenaka T, Ohishi M, Sugiura T. Relationship between Oral Hypofunction, and Protein Intake: A Cross-Sectional Study in Local Community-Dwelling Adults. Nutrients. 2021 Dec 7;13(12):4377. doi: 10.3390/nu13124377. PMID: 34959928; PMCID: PMC8705970.

1134: Pengpid S, Peltzer K. Multiple behavioural risk factors of non-communicable diseases among adolescents in four Caribbean countries: prevalence and correlates. Int J Adolesc Med Health. 2021 Jun 18;33(6):305-312. doi: 10.1515/ijamh-2021-0021. PMID: 34142510.

1135: Wang JX, Cheng YF, Pan XH, Luo P. Tissue-specific accumulation, transformation, and depuration of fipronil in adult crucian carp (Carassius auratus). Ecotoxicol Environ Saf. 2022 Jan 24;232:113234. doi: 10.1016/j.ecoenv.2022.113234. Epub ahead of print. PMID: 35085889.

1136: Sellem L, Jackson KG, Paper L, Givens ID, Lovegrove JA. Can individual fatty acids be used as functional biomarkers of dairy fat consumption in relation to cardiometabolic health? A narrative review. Br J Nutr. 2022 Jan 28:1-38. doi: 10.1017/S0007114522000289. Epub ahead of print. PMID: 35086579.

1137: Kazmierski KFM, Gillespie ML, Kuo S, Zurita T, Felix D, Rao U. Stress-Induced Eating Among Racial/Ethnic Groups in the United States: a Systematic Review. J Racial Ethn Health Disparities. 2021 Aug;8(4):912-926. doi: 10.1007/s40615-020-00849-w. Epub 2020 Aug 24. PMID: 32839895; PMCID: PMC7902740.

1138: Ashen MD, Carson KA, Ratchford EV. Coronary Calcium Scanning and Cardiovascular Risk Assessment Among Firefighters. Am J Prev Med. 2022 Jan;62(1):18-25. doi: 10.1016/j.amepre.2021.06.005. Epub 2021 Aug 26. PMID: 34456104.

1139: Mpagama SG, Msaji KS, Kaswaga O, Zurba LJ, Mbelele PM, Allwood BW, Ngungwa BS, Kisonga RM, Lesosky M, Rylance J, Mortimer K. The burden and determinants of post-TB lung disease. Int J Tuberc Lung Dis. 2021 Oct 1;25(10):846-853. doi: 10.5588/ijtld.21.0278. PMID: 34615582; PMCID: PMC8504494.

1140: Langer RD, Larsen SC, Ward LC, Heitmann BL. Phase angle measured by bioelectrical impedance analysis and the risk of cardiovascular disease among adult Danes. Nutrition. 2021 Sep;89:111280. doi: 10.1016/j.nut.2021.111280. Epub 2021 Apr 18. PMID: 34090217.

1141: Chai YH, Yang H, Huang GP, Wu T, Dong Y. Nursing Outcomes and Risk Factors of Patients with Chronic Obstructive Pulmonary Disease After Discharge. Int J Chron Obstruct Pulmon Dis. 2021 Oct 21;16:2911-2916. doi: 10.2147/COPD.S321375. PMID: 34707355; PMCID: PMC8544792.

1142: Kemp BJ, Thompson DR, Watson CJ, McGuigan K, Woodside JV, Ski CF. Effectiveness of family-based eHealth interventions in cardiovascular disease risk reduction: A systematic review. Prev Med. 2021 Aug;149:106608. doi: 10.1016/j.ypmed.2021.106608. Epub 2021 May 11. PMID: 33984372.

1143: Marijanović I, Kraljević M, Bevanda Glibo D, Buhovac T, Černi Obrdalj E. The Role of Family Physicians in the Prevention and Early Detection of Cancer in Herzegovina-Neretva and West-Herzegovina Canton. Psychiatr Danub. 2021 Sep;33(Suppl 10):89-96. PMID: 34672277.

1144: Laguzzi F, Maitusong B, Strawbridge RJ, Baldassarre D, Veglia F, Humphries SE, Rauramaa R, Kurl S, Smit AJ, Giral P, Silveira A, Tremoli E, Hamsten A, de Faire U, Gigante B, Leander K; IMPROVE Study group. Intake of food rich in saturated fat in relation to subclinical atherosclerosis and potential modulating effects from single genetic variants. Sci Rep. 2021 Apr 12;11(1):7866. doi: 10.1038/s41598-021-86324-w. PMID: 33846368; PMCID: PMC8042105.

1145: Wen J, Ma H, Yu Y, Zhang X, Guo D, Yin X, Yu X, Yin N, Wang J, Zhao Y. Sugar Content of Market Beverages and Children's Sugar Intake from Beverages in Beijing, China. Nutrients. 2021 Nov 28;13(12):4297. doi: 10.3390/nu13124297. PMID: 34959849; PMCID: PMC8708007.

1146: Schütze A, Morales-Agudelo P, Vidal M, Calafat AM, Ospina M. Quantification of glyphosate and other organophosphorus compounds in human urine via ion chromatography isotope dilution tandem mass spectrometry. Chemosphere. 2021 Jul;274:129427. doi: 10.1016/j.chemosphere.2020.129427. Epub 2021 Jan 12. PMID: 33529959; PMCID: PMC8717241.

1147: Garimella PS, Tighiouart H, Sarnak MJ, Levey AS, Ix JH. Tubular Secretion of Creatinine and Risk of Kidney Failure: The Modification of Diet in Renal Disease (MDRD) Study. Am J Kidney Dis. 2021 Jun;77(6):992-994. doi: 10.1053/j.ajkd.2020.09.017. Epub 2020 Nov 20. PMID: 33221368; PMCID: PMC8134514.

1148: Nikooyeh B, Abdollahi Z, Shariatzadeh N, Kalayi A, Zahedirad M, Neyestani T. Effect of latitude on seasonal variations of vitamin D and some cardiometabolic risk factors: national food and nutrition surveillance. East Mediterr Health J. 2021 Mar 23;27(3):269-278. doi: 10.26719/emhj.20.119. PMID: 33788216.

1149: Gatti M, Ippoliti I, Poluzzi E, Antonazzo IC, Moro PA, Moretti U, Menniti-Ippolito F, Mazzanti G, De Ponti F, Raschi E. Assessment of adverse reactions to α-lipoic acid containing dietary supplements through spontaneous reporting systems. Clin Nutr. 2021 Mar;40(3):1176-1185. doi: 10.1016/j.clnu.2020.07.028. Epub 2020 Jul 29. PMID: 32778460.

1150: Oh S, Lee SY, Kim DY, Woo S, Kim Y, Lee HJ, Jang HB, Park SI, Park KH, Lim H. Association of Dietary Patterns with Weight Status and Metabolic Risk Factors among Children and Adolescents. Nutrients. 2021 Mar 31;13(4):1153. doi: 10.3390/nu13041153. PMID: 33807269; PMCID: PMC8065894.

1151: Buah-Kwofie A, Humphries MS. Organochlorine pesticide accumulation in fish and catchment sediments of Lake St Lucia: Risks for Africa's largest estuary. Chemosphere. 2021 Jul;274:129712. doi: 10.1016/j.chemosphere.2021.129712. Epub 2021 Jan 25. PMID: 33529950.

1152: Svendsen K, Torheim LE, Fjelberg V, Sorprud A, Narverud I, Retterstøl K, Bogsrud MP, Holven KB, Myhrstad MCW, Telle-Hansen VH. Gender differences in nutrition literacy levels among university students and employees: a descriptive study. J Nutr Sci. 2021 Jul 30;10:e56. doi: 10.1017/jns.2021.47. PMID: 34367630; PMCID: PMC8327390.

1153: Luo F, Zeng KM, Cao JX, Zhou T, Lin SX, Ma WJ, Yang YP, Zhang ZH, Lu FT, Huang Y, Zhao HY, Zhang L. Predictive value of a reduction in the level of high-density lipoprotein-cholesterol in patients with non-small-cell lung cancer undergoing radical resection and adjuvant chemotherapy: a retrospective observational study. Lipids Health Dis. 2021 Sep 20;20(1):109. doi: 10.1186/s12944-021-01538-1. PMID: 34544437; PMCID: PMC8454045.

1154: Pucci G, Bisogni V, Battista F, D'Abbondanza M, Anastasio F, Crapa ME, Sanesi L, Desantis F, Troiani L, Papi F, Vaudo G. Association between Ideal Cardiovascular Health and aortic stiffness in Italian adolescents. The MACISTE study. Nutr Metab Cardiovasc Dis. 2021 Aug 26;31(9):2724-2732. doi: 10.1016/j.numecd.2021.05.035. Epub 2021 Jun 12. PMID: 34334290.

1155: Bhargava A, Bhargava M, Velayutham B, Thiruvengadam K, Watson B, Kulkarni B, Singh M, Dayal R, Pathak RR, Mitra A, Rade K, Sachdeva KS. The RATIONS (Reducing Activation of Tuberculosis by Improvement of Nutritional Status)

study: a cluster randomised trial of nutritional support (food rations) to reduce TB incidence in household contacts of patients with microbiologically confirmed pulmonary tuberculosis in communities with a high prevalence of undernutrition, Jharkhand, India. BMJ Open. 2021 May 20;11(5):e047210. doi: 10.1136/bmjopen-2020-047210. PMID: 34016663; PMCID: PMC8141431.

1156: Zhang C, Wang L, Sun W, Chen L, Zhang C, Li H, Yu J, Fan J, Ruan H, Zheng T, Wu D, Li S, Lu H, Wang M, Mol BW, Huang H, Wu Y. Effect of an individualised nutritional intervention on gestational diabetes mellitus prevention in a high-risk population screened by a prediction model: study protocol for a multicentre randomised controlled trial. BMC Pregnancy Childbirth. 2021 Aug 24;21(1):586. doi: 10.1186/s12884-021-04039-2. PMID: 34429102; PMCID: PMC8385988.

1157: Ahmad MH, Man CS, Othman F, He FJ, Salleh R, Noor NSM, Kozil WNKW, MacGregor G, Aris T. High sodium food consumption pattern among Malaysian population. J Health Popul Nutr. 2021 May 31;40(Suppl 1):4. doi: 10.1186/s41043-021-00230-5. PMID: 34059160; PMCID: PMC8165971.

1158: Man CS, Hock LK, Ying CY, Cheong KC, Kuay LK, Huey TC, Baharudin A, Aziz NSA. Is fast-food consumption a problem among adolescents in Malaysia? An analysis of the National School-Based Nutrition Survey, 2012. J Health Popul Nutr. 2021 Jul 16;40(1):31. doi: 10.1186/s41043-021-00254-x. PMID: 34271986; PMCID: PMC8285850.

1159: Johnston EA, Ibiebele TI, van der Pols JC, Webb PM; OPAL Study Group. Dietitian encounters after treatment for ovarian cancer. J Hum Nutr Diet. 2021 Dec;34(6):1053-1063. doi: 10.1111/jhn.12898. Epub 2021 Apr 9. PMID: 33749900.

1160: Lee C, Yang Q, Wolever RQ, Vorderstrasse A. Health Behavior Trajectories in High Cardiovascular Risk Populations: Secondary Analysis of a Clinical Trial. J Cardiovasc Nurs. 2021 Nov-Dec 01;36(6):E80-E90. doi: 10.1097/JCN.0000000000000850. PMID: 34495915.

1161: Zimmer M, Moshfegh AJ, Vernarelli JA, Barroso CS. Participation in the Special Supplemental Nutrition Program for Women, Infants, and Children and Dietary Intake in Children: Associations With Race and Ethnicity. Am J Prev Med. 2021 Dec 27:S0749-3797(21)00591-2. doi: 10.1016/j.amepre.2021.10.017. Epub ahead of print. PMID: 34969606.

1162: Manoogian ENC, Zadourian A, Lo HC, Gutierrez NR, Shoghi A, Rosander A, Pazargadi A, Wang X, Fleischer JG, Golshan S, Taub PR, Panda S. Protocol for a randomised controlled trial on the feasibility and effects of 10-hour time-restricted eating on cardiometabolic disease risk among career firefighters doing 24-hour shift work: the Healthy Heroes Study. BMJ Open. 2021 Jun 16;11(6):e045537. doi: 10.1136/bmjopen-2020-045537. PMID: 34135038; PMCID: PMC8211050.

1163: Baic S. Managing malnutrition in the community during the COVID-19 pandemic. Nurs Stand. 2021 Mar 3;36(3):61-66. doi: 10.7748/ns.2021.e11667. Epub

2021 Feb 22. PMID: 33615758.

1164: Dhana K, Aggarwal NT, Rajan KB, Barnes LL, Evans DA, Morris MC. Impact of the Apolipoprotein E ε4 Allele on the Relationship Between Healthy Lifestyle and Cognitive Decline: A Population-Based Study. Am J Epidemiol. 2021 Jul 1;190(7):1225-1233. doi: 10.1093/aje/kwab033. PMID: 33585904; PMCID: PMC8484773.

1165: Leung YY, Jin A, Tan KB, Ang LW, Yuan JM, Koh WP. Food sources of dietary fibre and risk of total knee replacement related to severe osteoarthritis, the Singapore Chinese Health Study. RMD Open. 2021 Jul;7(2):e001602. doi: 10.1136/rmdopen-2021-001602. PMID: 34330847; PMCID: PMC8327838.

1166: Janyajirawong R, Vilaichone RK, Sethasine S. Efficacy of Zinc Supplement in Minimal hepatic Encephalopathy: A prospective, Randomized Controlled Study (Zinc-MHE Trial). Asian Pac J Cancer Prev. 2021 Sep 1;22(9):2879-2887. doi: 10.31557/APJCP.2021.22.9.2879. PMID: 34582657.

1167: Merizian R, Mallinson RK, Kitsantas P, Gallo S. A Cross-Sectional Study of Psychological Flexibility as a Mediator for the Relationship Between Acculturative Stress and Cardiovascular Health Behaviors Among Second-Generation Arab Americans. J Transcult Nurs. 2021 Nov;32(6):697-706. doi: 10.1177/1043659620984515. Epub 2020 Dec 29. PMID: 33375879.

1168: Kim H, Anderson CA, Hu EA, Zheng Z, Appel LJ, He J, Feldman HI, Anderson AH, Ricardo AC, Bhat Z, Kelly TN, Chen J, Vasan RS, Kimmel PL, Grams ME, Coresh J, Clish CB, Rhee EP, Rebholz CM. Plasma Metabolomic Signatures of Healthy Dietary Patterns in the Chronic Renal Insufficiency Cohort (CRIC) Study. J Nutr. 2021 Oct 1;151(10):2894-2907. doi: 10.1093/jn/nxab203. PMID: 34195833; PMCID: PMC8485904.

1169: Firman S, Ramachandran R, Whelan K, Witard OC, O'Keeffe M. Protein status of people with phenylketonuria: a scoping review protocol. BMJ Open. 2021 Sep 14;11(9):e049883. doi: 10.1136/bmjopen-2021-049883. PMID: 34521668; PMCID: PMC8442069.

1170: Rothenberg SE, Korrick SA, Liu J, Nong Y, Nong H, Hong C, Trinh EP, Jiang X, Biasini FJ, Ouyang F. Maternal methylmercury exposure through rice ingestion and child neurodevelopment in the first three years: a prospective cohort study in rural China. Environ Health. 2021 Apr 28;20(1):50. doi: 10.1186/s12940-021-00732-z. PMID: 33910568; PMCID: PMC8082930.

1171: Chang SM, Lu IC, Chen YC, Hsuan CF, Lin YJ, Chuang HY. Behavioral Factors Associated with Medication Nonadherence in Patients with Hypertension. Int J Environ Res Public Health. 2021 Sep 12;18(18):9614. doi: 10.3390/ijerph18189614. PMID: 34574540; PMCID: PMC8469687.

1172: Sadeghi M, Simani M, Mohammadifard N, Talaei M, Roohafza H, Hassannejad R, Sarrafzadegan N. Longitudinal association of dietary fat intake with cardiovascular events in a prospective cohort study in Eastern Mediterranean

region. Int J Food Sci Nutr. 2021 Dec;72(8):1095-1104. doi: 10.1080/09637486.2021.1895725. Epub 2021 Mar 9. PMID: 33719857.

1173: Chapman JJ, Malacova E, Patterson S, Reavley N, Wyder M, Brown WJ, Hielscher E, Childs S, Scott JG. Psychosocial and lifestyle predictors of distress and well-being in people with mental illness during the COVID-19 pandemic. Australas Psychiatry. 2021 Dec;29(6):617-624. doi: 10.1177/10398562211025040. Epub 2021 Jun 30. PMID: 34192474.

1174: Liu YS, Wu QJ, Lv JL, Jiang YT, Sun H, Xia Y, Chang Q, Zhao YH. Dietary Carbohydrate and Diverse Health Outcomes: Umbrella Review of 30 Systematic Reviews and Meta-Analyses of 281 Observational Studies. Front Nutr. 2021 Apr 29;8:670411. doi: 10.3389/fnut.2021.670411. PMID: 33996880; PMCID: PMC8116488.

1175: Benaich S, Mehdad S, Andaloussi Z, Boutayeb S, Alamy M, Aguenaou H, Taghzouti K. Weight status, dietary habits, physical activity, screen time and sleep duration among university students. Nutr Health. 2021 Mar;27(1):69-78. doi: 10.1177/0260106020960863. Epub 2020 Oct 12. PMID: 33045923.

1176: Ma Y, He FJ, Sun Q, Yuan C, Kieneker LM, Curhan GC, MacGregor GA, Bakker SJL, Campbell NRC, Wang M, Rimm EB, Manson JE, Willett WC, Hofman A, Gansevoort RT, Cook NR, Hu FB. 24-Hour Urinary Sodium and Potassium Excretion and Cardiovascular Risk. N Engl J Med. 2022 Jan 20;386(3):252-263. doi: 10.1056/NEJMoa2109794. Epub 2021 Nov 13. PMID: 34767706.

1177: Guillaume JD, Jagai JS, Makelarski JA, Abramsohn EM, Lindau ST, Verma R, Ciaccio CE. COVID-19-Related Food Insecurity Among Households with Dietary Restrictions: A National Survey. J Allergy Clin Immunol Pract. 2021 Sep;9(9):3323-3330.e3. doi: 10.1016/j.jaip.2021.06.015. Epub 2021 Jun 24. PMID: 34174493; PMCID: PMC8421756.

1178: Rowan CJ, Eskander MA, Seabright E, Rodriguez DE, Linares EC, Gutierrez RQ, Adrian JC, Cummings D, Beheim B, Tolstrup K, Achrekar A, Kraft T, Michalik DE, Miyamoto MI, Allam AH, Wann LS, Narula J, Trumble BC, Stieglitz J, Thompson RC, Thomas GS, Kaplan HS, Gurven MD. Very Low Prevalence and Incidence of Atrial Fibrillation among Bolivian Forager-Farmers. Ann Glob Health. 2021 Feb 16;87(1):18. doi: 10.5334/aogh.3252. PMID: 33633929; PMCID: PMC7894370.

1179: Goraya N, Munoz-Maldonado Y, Simoni J, Wesson DE. Treatment of Chronic Kidney Disease-Related Metabolic Acidosis With Fruits and Vegetables Compared to NaHCO₃ Yields More and Better Overall Health Outcomes and at Comparable Five-Year Cost. J Ren Nutr. 2021 May;31(3):239-247. doi: 10.1053/j.jrn.2020.08.001. Epub 2020 Sep 18. PMID: 32952009.

1180: Yoon G, Davidson LA, Goldsby JS, Mullens DA, Ivanov I, Donovan SM, Chapkin RS. Exfoliated epithelial cell transcriptome reflects both small and large intestinal cell signatures in piglets. Am J Physiol Gastrointest Liver Physiol. 2021 Jul 1;321(1):G41-G51. doi: 10.1152/ajpgi.00017.2021. Epub 2021 May 5. PMID: 33949197; PMCID: PMC8321797.

1181: Sangild PT, Vonderohe C, Melendez Hebib V, Burrin DG. Potential Benefits of Bovine Colostrum in Pediatric Nutrition and Health. Nutrients. 2021 Jul 26;13(8):2551. doi: 10.3390/nu13082551. PMID: 34444709; PMCID: PMC8402036.

1182: Bortone I, Sardone R, Lampignano L, Castellana F, Zupo R, Lozupone M, Moretti B, Giannelli G, Panza F. How gait influences frailty models and health-related outcomes in clinical-based and population-based studies: a systematic review. J Cachexia Sarcopenia Muscle. 2021 Apr;12(2):274-297. doi: 10.1002/jcsm.12667. Epub 2021 Feb 16. PMID: 33590975; PMCID: PMC8061366.

1183: Huang YJ, Lee SL, Wu LM. Health-Promoting Lifestyle and Its Predictors in Adolescent Survivors of Childhood Cancer. J Pediatr Oncol Nurs. 2021 Jul-Aug;38(4):233-241. doi: 10.1177/1043454221992322. Epub 2021 Feb 17. PMID: 33595358.

1184: Kwok SWH, Pang PCP, Chung MH, Wu CST. Faculty Service-Learning Students as Home-Visitors: Outcomes of a Lifestyle Modification Program for Vulnerable Families With Residents in Rural Indonesian Communities. Front Public Health. 2021 May 12;9:597851. doi: 10.3389/fpubh.2021.597851. PMID: 34055707; PMCID: PMC8149593.

1185: Guerrant RL, Bolick DT, Swann JR. Modeling Enteropathy or Diarrhea with the Top Bacterial and Protozoal Pathogens: Differential Determinants of Outcomes. ACS Infect Dis. 2021 May 14;7(5):1020-1031. doi: 10.1021/acsinfecdis.0c00831. Epub 2021 Apr 26. PMID: 33901398; PMCID: PMC8154416.

1186: Agustina R, Meilianawati, Fenny, Atmarita, Suparmi, Susiloretni KA, Lestari W, Pritasari K, Shankar AH. Psychosocial, Eating Behavior, and Lifestyle Factors Influencing Overweight and Obesity in Adolescents. Food Nutr Bull. 2021 Jun;42(1_suppl):S72-S91. doi: 10.1177/0379572121992750. PMID: 34282658.

1187: Kowalska-Duplaga K, Gawlik-Scislo A, Krzesiek E, Jarocka-Cyrta E, Łazowska-Przeorek I, Duplaga M, Banaszkiewicz A. Determinants of disease-specific knowledge among children with inflammatory bowel disease and their parents: A multicentre study. World J Gastroenterol. 2021 Jul 21;27(27):4468-4480. doi: 10.3748/wjg.v27.i27.4468. PMID: 34366617; PMCID: PMC8316911.

1188: Sobieraj P, Bzikowska-Jura A, Raciborski F, Kucharska A, Szostak-Węgierek D, Kahan T. Is sodium and potassium intake assessed by diet-related mobile applications more harm than benefit? Kardiol Pol. 2022 Feb 8. doi: 10.33963/KP.a2022.0039. Epub ahead of print. PMID: 35133002.

1189: Qasem Surrati AM, Mohammedsaeed W, Shikieri ABE. Cardiovascular Risk Awareness and Calculated 10-Year Risk Among Female Employees at Taibah University 2019. Front Public Health. 2021 Oct 4;9:658243. doi: 10.3389/fpubh.2021.658243. PMID: 34671586; PMCID: PMC8520983.

1190: Pang S, Song P, Sun X, Qi W, Yang C, Song G, Wang Y, Zhang J. Dietary fructose and risk of metabolic syndrome in Chinese residents aged 45 and above: results from the China National Nutrition and Health Survey. Nutr J. 2021 Oct 3;20(1):83. doi: 10.1186/s12937-021-00739-9. PMID: 34602079; PMCID: PMC8489071.

1191: Gorgulho B, Alves MA, Teixeira JA, Santos RO, de Matos SA, Bittencourt MS, Benseñor I, Lotufo P, Marchioni DM. Dietary patterns associated with subclinical atherosclerosis: a cross-sectional analysis of the Brazilian Longitudinal Study of Adult Health (ELSA-Brasil) study. Public Health Nutr. 2021 Oct;24(15):5006-5014. doi: 10.1017/S1368980020005340. Epub 2021 Jan 8. PMID: 33413712.

1192: Zhang S, Gan S, Zhang Q, Liu L, Meng G, Yao Z, Wu H, Gu Y, Wang Y, Zhang T, Wang X, Sun S, Wang X, Zhou M, Jia Q, Song K, Qi L, Niu K. Ultra-processed food consumption and the risk of non-alcoholic fatty liver disease in the Tianjin Chronic Low-grade Systemic Inflammation and Health Cohort Study. Int J Epidemiol. 2021 Sep 16:dyab174. doi: 10.1093/ije/dyab174. Epub ahead of print. PMID: 34528679.

1193: Boe LA, Tinker LF, Shaw PA. An approximate quasi-likelihood approach for error-prone failure time outcomes and exposures. Stat Med. 2021 Oct 15;40(23):5006-5024. doi: 10.1002/sim.9108. Epub 2021 Jun 22. PMID: 34519082.

1194: Chung M, Ruan M, Cara KC, Yao Q, Penkert LP, Chen J. Vitamin D and Calcium in Children 0-36 Months: A Scoping Review of Health Outcomes. J Am Coll Nutr. 2021 May-Jun;40(4):367-396. doi: 10.1080/07315724.2020.1774822. Epub 2020 Jul 14. PMID: 32662755.

1195: Laine JE, Huybrechts I, Gunter MJ, Ferrari P, Weiderpass E, Tsilidis K, Aune D, Schulze MB, Bergmann M, Temme EHM, Boer JMA, Agnoli C, Ericson U, Stubbendorff A, Ibsen DB, Dahm CC, Deschasaux M, Touvier M, Kesse-Guyot E, Sánchez Pérez MJ, Rodríguez Barranco M, Tong TYN, Papier K, Knuppel A, Boutron-Ruault MC, Mancini F, Severi G, Srour B, Kühn T, Masala G, Agudo A, Skeie G, Rylander C, Sandanger TM, Riboli E, Vineis P. Co-benefits from sustainable dietary shifts for population and environmental health: an assessment from a large European cohort study. Lancet Planet Health. 2021 Nov;5(11):e786-e796. doi: 10.1016/S2542-5196(21)00250-3. Epub 2021 Oct 22. PMID: 34688354; PMCID: PMC8581185.

1196: Hallum S, Gerds TA, Sehested TSG, Jakobsen MA, Tjønneland A, Kamper-Jørgensen M. Impact of Male-Origin Microchimerism on Cardiovascular Disease in Women: A Prospective Cohort Study. Am J Epidemiol. 2021 May 4;190(5):853-863. doi: 10.1093/aje/kwaa250. PMID: 33184639.

1197: Noble EE, Olson CA, Davis E, Tsan L, Chen YW, Schade R, Liu C, Suarez A, Jones RB, de La Serre C, Yang X, Hsiao EY, Kanoski SE. Gut microbial taxa elevated by dietary sugar disrupt memory function. Transl Psychiatry. 2021 Mar 31;11(1):194. doi: 10.1038/s41398-021-01309-7. PMID: 33790226; PMCID:

PMC8012713.

1198: Naran V, Namous N, Eddy VJ, Le Guen CL, Sarwer DB, Soans RS. The effects of the COVID-19 pandemic on patients with obesity undergoing bariatric care. Surg Obes Relat Dis. 2021 Oct;17(10):1714-1720. doi: 10.1016/j.soard.2021.05.026. Epub 2021 May 27. PMID: 34187742; PMCID: PMC8154188.

1199: Enriquez-Martinez OG, Martins MCT, Pereira TSS, Pacheco SOS, Pacheco FJ, Lopez KV, Huancahuire-Vega S, Silva DA, Mora-Urda AI, Rodriguez-Vásquez M, Montero López MP, Molina MCB. Diet and Lifestyle Changes During the COVID-19 Pandemic in Ibero-American Countries: Argentina, Brazil, Mexico, Peru, and Spain. Front Nutr. 2021 Jun 2;8:671004. doi: 10.3389/fnut.2021.671004. PMID: 34150828; PMCID: PMC8206276.

1200: Zhang LX, Li CX, Kakar MU, Khan MS, Wu PF, Amir RM, Dai DF, Naveed M, Li QY, Saeed M, Shen JQ, Rajput SA, Li JH. Resveratrol (RV): A pharmacological review and call for further research. Biomed Pharmacother. 2021 Nov;143:112164. doi: 10.1016/j.biopha.2021.112164. Epub 2021 Oct 2. PMID: 34649335.

1201: Norman K, Haß U, Pirlich M. Malnutrition in Older Adults-Recent Advances and Remaining Challenges. Nutrients. 2021 Aug 12;13(8):2764. doi: 10.3390/nu13082764. PMID: 34444924; PMCID: PMC8399049.

1202: Venthodika A, Chhikara N, Mann S, Garg MK, Sofi SA, Panghal A. Bioactive compounds of Aegle marmelos L., medicinal values and its food applications: A critical review. Phytother Res. 2021 Apr;35(4):1887-1907. doi: 10.1002/ptr.6934. Epub 2020 Nov 6. PMID: 33159390.

1203: Li M, Xiao M, Xiao Q, Chen Y, Guo Y, Sun J, Li R, Li C, Zhu Z, Qiu H, Liu X, Lu S. Perchlorate and chlorate in breast milk, infant formulas, baby supplementary food and the implications for infant exposure. Environ Int. 2022 Jan;158:106939. doi: 10.1016/j.envint.2021.106939. Epub 2021 Oct 18. PMID: 34673317.

1204: Zhang Y, Guo X, Zhang N, Yan X, Li M, Zhou M, He H, Li Y, Guo W, Zhang M, Zhang J, Ma G. Effect of Mobile-Based Lifestyle Intervention on Body Weight, Glucose and Lipid Metabolism among the Overweight and Obese Elderly Population in China: A Randomized Controlled Trial Protocol. Int J Environ Res Public Health. 2021 May 1;18(9):4854. doi: 10.3390/ijerph18094854. PMID: 34062901; PMCID: PMC8125256.

1205: Montoro-Huguet MA, Santolaria-Piedrafita S, Cañamares-Orbis P, García-Erce JA. Iron Deficiency in Celiac Disease: Prevalence, Health Impact, and Clinical Management. Nutrients. 2021 Sep 28;13(10):3437. doi: 10.3390/nu13103437. PMID: 34684433; PMCID: PMC8537360.

1206: Majeed M, Majeed S, Nagabhushanam K. An Open-Label Pilot Study on Macumax Supplementation for Dry-Type Age-Related Macular Degeneration. J Med Food. 2021

May;24(5):551-557. doi: 10.1089/jmf.2020.0097. Epub 2020 Aug 27. PMID: 33180005; PMCID: PMC8140349.

1207: Du X, Xin H. Association between cholesterol intake and all-cause mortality: NHANES-linked mortality study. Cent Eur J Public Health. 2021 Jun;29(2):117-121. doi: 10.21101/cejph.a6276. PMID: 34245551.

1208: Pearson GJ, Thanassoulis G, Anderson TJ, Barry AR, Couture P, Dayan N, Francis GA, Genest J, Grégoire J, Grover SA, Gupta M, Hegele RA, Lau D, Leiter LA, Leung AA, Lonn E, Mancini GBJ, Manjoo P, McPherson R, Ngui D, Piché ME, Poirier P, Sievenpiper J, Stone J, Ward R, Wray W. 2021 Canadian Cardiovascular Society Guidelines for the Management of Dyslipidemia for the Prevention of Cardiovascular Disease in Adults. Can J Cardiol. 2021 Aug;37(8):1129-1150. doi: 10.1016/j.cjca.2021.03.016. Epub 2021 Mar 26. PMID: 33781847.

1209: Tippairote T, Peana M, Chirumbolo S, Bjørklund G. Individual risk management strategy for SARS-CoV-2 infection: A step toward personalized healthcare. Int Immunopharmacol. 2021 Jul;96:107629. doi: 10.1016/j.intimp.2021.107629. Epub 2021 Apr 1. PMID: 33862554; PMCID: PMC8015431.

1210: Zaman A, Sloggett KJ, Caldwell AE, Catenacci V, Cornier MA, Grau L, Vetter C, Rynders C, Thomas E. The Effects of the COVID-19 Pandemic on Weight Loss in Participants in a Behavioral Weight Loss Intervention. Obesity (Silver Spring). 2022 Feb 3. doi: 10.1002/oby.23399. Epub ahead of print. PMID: 35118814.

1211: Razmpoosh E, Safi S, Nadjarzadeh A, Fallahzadeh H, Abdollahi N, Mazaheri M, Nazari M, Salehi-Abargouei A. The effect of Nigella sativa supplementation on cardiovascular risk factors in obese and overweight women: a crossover, double-blind, placebo-controlled randomized clinical trial. Eur J Nutr. 2021 Jun;60(4):1863-1874. doi: 10.1007/s00394-020-02374-2. Epub 2020 Sep 2. PMID: 32876804.

1212: Cui X, Zhang L, Yang D, Li J, Liu Q, Sui H, Liu Z, Zhou P. Occurrence of 3- and 2-monochloropropanediol esters in infant formulas in China and exposure assessment. Food Addit Contam Part A Chem Anal Control Expo Risk Assess. 2021 Sep;38(9):1470-1480. doi: 10.1080/19440049.2021.1925164. Epub 2021 Jul 6. PMID: 34229561.

1213: Chen Z, Dong F, Ren X, Wu X, Yuan L, Li L, Li W, Zheng Y. Enantioselective fate of dinotefuran from tomato cultivation to home canning for refining dietary exposure. J Hazard Mater. 2021 Mar 5;405:124254. doi: 10.1016/j.jhazmat.2020.124254. Epub 2020 Oct 12. PMID: 33535352.

1214: Kim J, Jeong K, Lee S, Seo BN, Baek Y. Low nutritional status links to the prevalence of pre-metabolic syndrome and its cluster in metabolically high-risk Korean adults: A cross-sectional study. Medicine (Baltimore). 2021 May 21;100(20):e25905. doi: 10.1097/MD.0000000000025905. PMID: 34011058; PMCID: PMC8137084.

1215: Eisenhauer CM, Brito F, Kupzyk K, Yoder A, Almeida F, Beller RJ, Miller J, Hageman PA. Mobile health assisted self-monitoring is acceptable for supporting weight loss in rural men: a pragmatic randomized controlled feasibility trial. BMC Public Health. 2021 Aug 18;21(1):1568. doi: 10.1186/s12889-021-11618-7. PMID: 34407782; PMCID: PMC8375071.

1216: Sakamoto Y, Oono F, Iida K, Wang PL, Tachi Y. Relationship between vitamin D receptor gene polymorphisms (BsmI, TaqI, ApaI, and FokI) and calcium intake on bone mass in young Japanese women. BMC Womens Health. 2021 Feb 19;21(1):76. doi: 10.1186/s12905-021-01222-7. PMID: 33607983; PMCID: PMC7893901.

1217: Cooper J, Baumgartner K, Smith A, St Louis J. Liver Disease: Nonalcoholic Fatty Liver Disease. FP Essent. 2021 Dec;511:29-35. PMID: 34855339.

1218: Fang K, He Y, Fang Y, Lian Y. Relationship of Sodium Intake with Overweight/Obesity among Chinese Children and Adolescents: Data from the CNNHS 2010-2012. Int J Environ Res Public Health. 2021 Apr 25;18(9):4551. doi: 10.3390/ijerph18094551. PMID: 33923000; PMCID: PMC8123343.

1219: Kelly JR, Gounden P, McLoughlin A, Legris Z, O'Carroll T, McCafferty R, Marques L, Haran M, Farrelly R, Loughrey K, Flynn G, Corvin A, Dolan C. Minding metabolism: targeted interventions to improve cardio-metabolic monitoring across early and chronic psychosis. Ir J Med Sci. 2022 Feb;191(1):337-346. doi: 10.1007/s11845-021-02576-5. Epub 2021 Mar 8. PMID: 33683562; PMCID: PMC7938026.

1220: Huang L, Xiao D, Zhang X, Sandhu AK, Chandra P, Kay C, Edirisinghe I, Burton-Freeman B. Strawberry Consumption, Cardiometabolic Risk Factors, and Vascular Function: A Randomized Controlled Trial in Adults with Moderate Hypercholesterolemia. J Nutr. 2021 Jun 1;151(6):1517-1526. doi: 10.1093/jn/nxab034. PMID: 33758944.

1221: Qing Y, Yang J, Zhu Y, Li Y, Zheng W, Wu M, He G. Dose-response evaluation of urinary cadmium and kidney injury biomarkers in Chinese residents and dietary limit standards. Environ Health. 2021 Jun 30;20(1):75. doi: 10.1186/s12940-021-00760-9. PMID: 34193170; PMCID: PMC8247151.

1222: Van Horne YO, Chief K, Charley PH, Begay MG, Lothrop N, Bell ML, Canales RA, Teufel-Shone NI, Beamer PI. Impacts to Diné activities with the San Juan River after the Gold King Mine Spill. J Expo Sci Environ Epidemiol. 2021 Sep;31(5):852-866. doi: 10.1038/s41370-021-00290-z. Epub 2021 Feb 1. PMID: 33526814; PMCID: PMC8325715.

1223: Yang BI, Park JA, Lee JY, Jin BH. Effects of Lingual and Palatal Site Toothbrushing on Periodontal Disease in the Elderly: A Cross-Sectional Study. Int J Environ Res Public Health. 2021 May 11;18(10):5067. doi: 10.3390/ijerph18105067. PMID: 34064811; PMCID: PMC8151310.

1224: Iacone R, Iaccarino Idelson P, Russo O, Donfrancesco C, Krogh V, Sieri S,

Macchia PE, Formisano P, Lo Noce C, Palmieri L, Galeone D, Rendina D, Galletti F, Di Lenarda A, Giampaoli S, Strazzullo P, On Behalf Of The Minisal-Gircsi Study Group. Iodine Intake from Food and Iodized Salt as Related to Dietary Salt Consumption in the Italian Adult General Population. Nutrients. 2021 Sep 30;13(10):3486. doi: 10.3390/nu13103486. PMID: 34684487; PMCID: PMC8537510.

1225: Aminde LN, Phung HN, Phung D, Cobiac LJ, Veerman JL. Dietary Salt Reduction, Prevalence of Hypertension and Avoidable Burden of Stroke in Vietnam: Modelling the Health and Economic Impacts. Front Public Health. 2021 Jun 4;9:682975. doi: 10.3389/fpubh.2021.682975. PMID: 34150712; PMCID: PMC8213032.

1226: Wang B, Cheng H, Lin C, Zhang X, Duan X, Wang Q, Xu D. Arsenic exposure analysis for children living in central China: From ingestion exposure to biomarkers. Chemosphere. 2022 Jan;287(Pt 2):132194. doi: 10.1016/j.chemosphere.2021.132194. Epub 2021 Sep 7. PMID: 34509767.

1227: Bidira K, Tamiru D, Belachew T. Anthropometric failures and its associated factors among preschool-aged children in a rural community in southwest Ethiopia. PLoS One. 2021 Nov 29;16(11):e0260368. doi: 10.1371/journal.pone.0260368. PMID: 34843555; PMCID: PMC8629177.

1228: Manzoor S, Hisam A, Aziz S, Mashhadi SF, Haq ZU. Effectiveness of Mobile Health Augmented Cardiac Rehabilitation on Behavioural Outcomes among Post-acute Coronary Syndrome Patients: A Randomised Controlled Trial. J Coll Physicians Surg Pak. 2021 Oct;31(10):1148-1153. doi: 10.29271/jcpsp.2021.10.1148. PMID: 34601832.

1229: Trieu K, Bhat S, Dai Z, Leander K, Gigante B, Qian F, Korat AVA, Sun Q, Pan XF, Laguzzi F, Cederholm T, de Faire U, Hellénius ML, Wu JHY, Risérus U, Marklund M. Biomarkers of dairy fat intake, incident cardiovascular disease, and all-cause mortality: A cohort study, systematic review, and meta-analysis. PLoS Med. 2021 Sep 21;18(9):e1003763. doi: 10.1371/journal.pmed.1003763. PMID: 34547017; PMCID: PMC8454979.

1230: Zhang H, Qiu Y, Zhang J, Ma Z, Amoah AN, Cao Y, Wang X, Fu P, Lyu Q. The effect of oral nutritional supplements on the nutritional status of community elderly people with malnutrition or risk of malnutrition. Asia Pac J Clin Nutr. 2021 Sep;30(3):415-423. doi: 10.6133/apjcn.202109_30(3).0008. PMID: 34587701.

1231: Fletcher P, Hamilton RF Jr, Rhoderick JF, Postma B, Buford M, Pestka JJ, Holian A. Therapeutic treatment of dietary docosahexaenoic acid for particle-induced pulmonary inflammation in Balb/c mice. Inflamm Res. 2021 Mar;70(3):359-373. doi: 10.1007/s00011-021-01443-4. Epub 2021 Feb 10. PMID: 33566171; PMCID: PMC8127607.

1232: Chan K, Gallant J, Leemaqz S, Baldwin DA, Borath M, Kroeun H, Measelle JR, Ngik R, Prak S, Wieringa FT, Yelland LN, Green TJ, Whitfield KC. Assessment of salt intake to consider salt as a fortification vehicle for thiamine in Cambodia. Ann N Y Acad Sci. 2021 Aug;1498(1):85-95. doi: 10.1111/nyas.14562.

Epub 2021 Jan 7. PMID: 33415757; PMCID: PMC8451827.

1233: Del Chierico F, Trapani V, Petito V, Reddel S, Pietropaolo G, Graziani C, Masi L, Gasbarrini A, Putignani L, Scaldaferri F, Wolf FI. Dietary Magnesium Alleviates Experimental Murine Colitis through Modulation of Gut Microbiota. Nutrients. 2021 Nov 23;13(12):4188. doi: 10.3390/nu13124188. PMID: 34959740; PMCID: PMC8707433.

1234: Wagnild JM, Pollard TM. How is television time linked to cardiometabolic health in adults? A critical systematic review of the evidence for an effect of watching television on eating, movement, affect and sleep. BMJ Open. 2021 May 5;11(5):e040739. doi: 10.1136/bmjopen-2020-040739. PMID: 33952532; PMCID: PMC8103379.

1235: Sellem L, Flourakis M, Jackson KG, Joris PJ, Lumley J, Lohner S, Mensink RP, Soedamah-Muthu SS, Lovegrove JA. Impact of Individual Dietary Saturated Fatty Acid Replacement on Circulating Lipids and Other Biomarkers of Cardiometabolic Health: A Systematic Review and Meta-analysis of RCTs in Humans. Adv Nutr. 2021 Nov 25:nmab143. doi: 10.1093/advances/nmab143. Epub ahead of print. PMID: 34849532.

1236: Miklavcic JJ, Li Q, Skolnick J, Thomson ABR, Mazurak VC, Clandinin MT. Ganglioside Alters Phospholipase Trafficking, Inhibits NF-κB Assembly, and Protects Tight Junction Integrity. Front Nutr. 2021 Jul 5;8:705172. doi: 10.3389/fnut.2021.705172. PMID: 34291075; PMCID: PMC8286996.

1237: Wang DD, Li Y, Bhupathiraju SN, Rosner BA, Sun Q, Giovannucci EL, Rimm EB, Manson JE, Willett WC, Stampfer MJ, Hu FB. Fruit and Vegetable Intake and Mortality: Results From 2 Prospective Cohort Studies of US Men and Women and a Meta-Analysis of 26 Cohort Studies. Circulation. 2021 Apr 27;143(17):1642-1654. doi: 10.1161/CIRCULATIONAHA.120.048996. Epub 2021 Mar 1. PMID: 33641343; PMCID: PMC8084888.

1238: Zhang P, Sun J, Guo Y, Han M, Yang F, Sun Y. Association between retinol intake and hyperuricaemia in adults. Public Health Nutr. 2021 Jun;24(8):2205-2214. doi: 10.1017/S1368980020000324. Epub 2020 May 21. PMID: 32434600.

1239: Heydenreich J, Schweter A, Lührmann P. Impact of physical activity, anthropometric, body composition, and dietary factors on bone stiffness in German university students. J Sports Med Phys Fitness. 2021 Apr;61(4):571-581. doi: 10.23736/S0022-4707.20.11281-7. Epub 2020 Jul 30. PMID: 32744046.

1240: Terman SW, Aubert CE, Hill CE, Skvarce J, Burke JF, Mintzer S. Cardiovascular disease risk, awareness, and treatment in people with epilepsy. Epilepsy Behav. 2021 Apr;117:107878. doi: 10.1016/j.yebeh.2021.107878. Epub 2021 Mar 6. PMID: 33690068.

1241: Caspersen IH, Iglesias-Vázquez L, Abel MH, Brantsæter AL, Arija V, Erlund

I, Meltzer HM. Iron status in mid-pregnancy and associations with interpregnancy interval, hormonal contraceptives, dietary factors and supplement use. Br J Nutr. 2021 Oct 28;126(8):1270-1280. doi: 10.1017/S0007114521000295. Epub 2021 Jan 26. PMID: 33494856.

1242: Wauters L, Slaets H, De Paepe K, Ceulemans M, Wetzels S, Geboers K, Toth J, Thys W, Dybajlo R, Walgraeve D, Biessen E, Verbeke K, Tack J, Van de Wiele T, Hellings N, Vanuytsel T. Efficacy and safety of spore-forming probiotics in the treatment of functional dyspepsia: a pilot randomised, double-blind, placebo-controlled trial. Lancet Gastroenterol Hepatol. 2021 Oct;6(10):784-792. doi: 10.1016/S2468-1253(21)00226-0. Epub 2021 Aug 3. Erratum in: Lancet Gastroenterol Hepatol. 2021 Aug 27;: PMID: 34358486.

1243: LaBarre JL, Miller AL, Bauer KW, Burant CF, Lumeng JC. Early life stress exposure associated with reduced polyunsaturated-containing lipids in low-income children. Pediatr Res. 2021 Apr;89(5):1310-1315. doi: 10.1038/s41390-020-0989-0. Epub 2020 Jun 3. PMID: 32492693; PMCID: PMC7710594.

1244: Peng X, Gao Q, Zhou J, Ma J, Zhao D, Hao L. Association between dietary antioxidant vitamins intake and homocysteine levels in middle-aged and older adults with hypertension: a cross-sectional study. BMJ Open. 2021 Oct 12;11(10):e045732. doi: 10.1136/bmjopen-2020-045732. PMID: 34642187; PMCID: PMC8513268.

1245: Andreu A, Flores L, Molero J, Mestre C, Obach A, Torres F, Moizé V, Vidal J, Navinés R, Peri JM, Cañizares S. Patients Undergoing Bariatric Surgery: a Special Risk Group for Lifestyle, Emotional and Behavioral Adaptations During the COVID-19 Lockdown. Lessons from the First Wave. Obes Surg. 2022 Feb;32(2):441-449. doi: 10.1007/s11695-021-05792-1. Epub 2021 Nov 17. PMID: 34791617; PMCID: PMC8598099.

1246: Zarkani AA, Schikora A. Mechanisms adopted by Salmonella to colonize plant hosts. Food Microbiol. 2021 Oct;99:103833. doi: 10.1016/j.fm.2021.103833. Epub 2021 May 20. PMID: 34119117.

1247: Pasricha SR, Hasan MI, Braat S, Larson LM, Tipu SMM, Hossain SJ, Shiraji S, Baldi A, Bhuiyan MSA, Tofail F, Fisher J, Grantham-McGregor S, Simpson JA, Hamadani JD, Biggs BA. Benefits and Risks of Iron Interventions in Infants in Rural Bangladesh. N Engl J Med. 2021 Sep 9;385(11):982-995. doi: 10.1056/NEJMoa2034187. PMID: 34496174.

1248: Wang L, Ze F, Li J, Mi L, Han B, Niu H, Zhao N. Trends of global burden of atrial fibrillation/flutter from Global Burden of Disease Study 2017. Heart. 2021 Jun;107(11):881-887. doi: 10.1136/heartjnl-2020-317656. Epub 2020 Nov 4. PMID: 33148545.

1249: Hu W, Jiang S, Liao Y, Li J, Dong F, Guo J, Wang X, Fei L, Cui Y, Ren X, Xu N, Zhao L, Chen L, Zheng Y, Li L, Patzak A, Persson PB, Zheng Z, Lai EY. High phosphate impairs arterial endothelial function through AMPK-related pathways in

mouse resistance arteries. Acta Physiol (Oxf). 2021 Apr;231(4):e13595. doi: 10.1111/apha.13595. Epub 2020 Dec 20. PMID: 33835704.

1250: Horikawa C, Aida R, Tanaka S, Kamada C, Tanaka S, Yoshimura Y, Kodera R, Fujihara K, Kawasaki R, Moriya T, Yamashita H, Ito H, Sone H, Araki A. Sodium Intake and Incidence of Diabetes Complications in Elderly Patients with Type 2 Diabetes-Analysis of Data from the Japanese Elderly Diabetes Intervention Study (J-EDIT). Nutrients. 2021 Feb 21;13(2):689. doi: 10.3390/nu13020689. PMID: 33670045; PMCID: PMC7926689.

1251: Cassidy N, Sheahan D, Fox L, Brown L, Galvin L, Cassidy E, Sheridan M, O'Dowd G, O'Reilly KMA. Perspectives of Interstitial Lung Disease Patients and Carers During COVID-19. Ir Med J. 2021 Aug 19;114(7):410. PMID: 34520645.

1252: Jodczyk AM, Gruba G, Sikora Z, Kasiak PS, Gębarowska J, Adamczyk N, Mamcarz A, Śliż D. PaLS Study: How Has the COVID-19 Pandemic Influenced Physical Activity and Nutrition? Observations a Year after the Outbreak of the Pandemic. Int J Environ Res Public Health. 2021 Sep 13;18(18):9632. doi: 10.3390/ijerph18189632. PMID: 34574557; PMCID: PMC8470829.

1253: Rafie C, Hosig K, Wenzel SG, Borowski S, Jiles KA, Schlenker E. Implementation and outcomes of the Balanced Living with Diabetes program conducted by Cooperative Extension in rural communities in Virginia. Rural Remote Health. 2021 Aug;21(3):6620. doi: 10.22605/RRH6620. Epub 2021 Aug 25. PMID: 34428915.

1254: Okoye EA, Bocca B, Ruggieri F, Ezejiofor AN, Nwaogazie IL, Frazzoli C, Orisakwe OE. Arsenic and toxic metals in meat and fish consumed in Niger delta, Nigeria: Employing the margin of exposure approach in human health risk assessment. Food Chem Toxicol. 2022 Jan;159:112767. doi: 10.1016/j.fct.2021.112767. Epub 2021 Dec 11. PMID: 34906653.

1255: Sharma A, Rao SSC, Kearns K, Orleck KD, Waldman SA. Review article: diagnosis, management and patient perspectives of the spectrum of constipation disorders. Aliment Pharmacol Ther. 2021 Jun;53(12):1250-1267. doi: 10.1111/apt.16369. Epub 2021 Apr 28. PMID: 33909919; PMCID: PMC8252518.

1256: Zaharia OP, Kupriyanova Y, Karusheva Y, Markgraf DF, Kantartzis K, Birkenfeld AL, Trenell M, Sahasranaman A, Cheyette C, Kössler T, Bódis K, Burkart V, Hwang JH, Roden M, Szendroedi J, Pesta DH. Improving insulin sensitivity, liver steatosis and fibrosis in type 2 diabetes by a food-based digital education-assisted lifestyle intervention program: a feasibility study. Eur J Nutr. 2021 Oct;60(7):3811-3818. doi: 10.1007/s00394-021-02521-3. Epub 2021 Apr 11. PMID: 33839905; PMCID: PMC8437928.

1257: Monteiro M, Fontes T, Ferreira-Pêgo C. Nutrition Literacy of Portuguese Adults-A Pilot Study. Int J Environ Res Public Health. 2021 Mar 19;18(6):3177. doi: 10.3390/ijerph18063177. PMID: 33808637; PMCID: PMC8003506.

1258: Munjral S, Ahluwalia P, Jamthikar AD, Puvvula A, Saba L, Faa G, Singh IM, Chadha PS, Turk M, Johri AM, Khanna NN, Viskovic K, Mavrogeni S, Laird JR, Pareek G, Miner M, Sobel DW, Balestrieri A, Sfikakis PP, Tsoulfas G, Protogerou A, Misra P, Agarwal V, Kitas GD, Kolluri R, Teji J, Al-Maini M, Dhanjil SK, Sockalingam M, Saxena A, Sharma A, Rathore V, Fatemi M, Alizad A, Viswanathan V, Krishnan PK, Omerzu T, Naidu S, Nicolaides A, Suri JS. Nutrition, atherosclerosis, arterial imaging, cardiovascular risk stratification, and manifestations in COVID-19 framework: a narrative review. Front Biosci (Landmark Ed). 2021 Nov 30;26(11):1312-1339. doi: 10.52586/5026. PMID: 34856770.

1259: Sørensen M, Hvidtfeldt UA, Poulsen AH, Thygesen LC, Frohn LM, Ketzel M, Christensen JH, Brandt J, Khan J, Raaschou-Nielsen O. The effect of adjustment to register-based and questionnaire-based covariates on the association between air pollution and cardiometabolic disease. Environ Res. 2022 Jan;203:111886. doi: 10.1016/j.envres.2021.111886. Epub 2021 Aug 16. PMID: 34411546.

1260: Barnes C, Yoong SL, Nathan N, Wolfenden L, Wedesweiler T, Kerr J, Ward DS, Grady A. Feasibility of a Web-Based Implementation Intervention to Improve Child Dietary Intake in Early Childhood Education and Care: Pilot Randomized Controlled Trial. J Med Internet Res. 2021 Dec 15;23(12):e25902. doi: 10.2196/25902. PMID: 34914617; PMCID: PMC8717135.

1261: Shah JM, Ramsbotham J, Seib C, Muir R, Bonner A. A scoping review of the role of health literacy in chronic kidney disease self-management. J Ren Care. 2021 Dec;47(4):221-233. doi: 10.1111/jorc.12364. Epub 2021 Feb 2. PMID: 33533199.

1262: Razavi AC, Bazzano LA, He J, Krousel-Wood M, Chen J, Fernandez C, Whelton SP, Kelly TN. Early Contributors to Healthy Arterial Aging Versus Premature Atherosclerosis in Young Adults: The Bogalusa Heart Study. J Am Heart Assoc. 2021 Jun 15;10(12):e020774. doi: 10.1161/JAHA.121.020774. Epub 2021 Jun 5. PMID: 34096330; PMCID: PMC8477892.

1263: Hadrová S, Sedláková K, Křížová L, Malyugina S. Alternative and Unconventional Feeds in Dairy Diets and Their Effect on Fatty Acid Profile and Health Properties of Milk Fat. Animals (Basel). 2021 Jun 18;11(6):1817. doi: 10.3390/ani11061817. PMID: 34207160; PMCID: PMC8234496.

1264: Jiang YW, Sun ZH, Tong WW, Yang K, Guo KQ, Liu G, Pan A. Dietary Intake and Circulating Concentrations of Carotenoids and Risk of Type 2 Diabetes: A Dose-Response Meta-Analysis of Prospective Observational Studies. Adv Nutr. 2021 Oct 1;12(5):1723-1733. doi: 10.1093/advances/nmab048. PMID: 33979433; PMCID: PMC8483954.

1265: Yang J, Xie Q, Wang Y, Wang J, Zhang Y, Zhang C, Wang D. Exposure of the residents around the Three Gorges Reservoir, China to chromium, lead and arsenic and their health risk via food consumption. Ecotoxicol Environ Saf. 2021 Nov 19;228:112997. doi: 10.1016/j.ecoenv.2021.112997. Epub ahead of print. PMID: 34808509.

1266: Xu F, Du G, Xu D, Chen L, Zha X, Guo Z. Residual behavior and dietary intake risk assessment of flonicamid, dinotefuran and its metabolites on peach trees. J Sci Food Agric. 2021 Nov;101(14):5842-5850. doi: 10.1002/jsfa.11236. Epub 2021 Apr 14. PMID: 33788960.

1267: Hiruy AF, Opoku S, Xiong Q, Jin Q, Zhao J, Lin X, He S, Zuo X, Ying C. Nutritional predictors associated with malnutrition in continuous ambulatory peritoneal dialysis patients. Clin Nutr ESPEN. 2021 Oct;45:454-461. doi: 10.1016/j.clnesp.2021.06.033. Epub 2021 Jul 10. PMID: 34620355.

1268: Geretto M, Ferrari M, De Angelis R, Crociata F, Sebastiani N, Pulliero A, Au W, Izzotti A. Occupational Exposures and Environmental Health Hazards of Military Personnel. Int J Environ Res Public Health. 2021 May 18;18(10):5395. doi: 10.3390/ijerph18105395. PMID: 34070145; PMCID: PMC8158372.

1269: Sahebkar A, Katsiki N, Ward N, Reiner Ž. Flaxseed Supplementation Reduces Plasma Lipoprotein(a) Levels: A Meta-Analysis. Altern Ther Health Med. 2021 May;27(3):50-53. PMID: 31634874.

1270: Draskau MK, Rosenmai AK, Scholze M, Pedersen M, Boberg J, Christiansen S, Svingen T. Human-relevant concentrations of the antifungal drug clotrimazole disrupt maternal and fetal steroid hormone profiles in rats. Toxicol Appl Pharmacol. 2021 Jul 1;422:115554. doi: 10.1016/j.taap.2021.115554. Epub 2021 Apr 25. PMID: 33910022.

1271: Xu Y, Xiong J, Gao W, Wang X, Shan S, Zhao L, Cheng G. Dietary Fat and Polyunsaturated Fatty Acid Intakes during Childhood Are Prospectively Associated with Puberty Timing Independent of Dietary Protein. Nutrients. 2022 Jan 10;14(2):275. doi: 10.3390/nu14020275. PMID: 35057456; PMCID: PMC8778261.

1272: Clarke H, Clark S, Birkin M, Iles-Smith H, Glaser A, Morris MA. Understanding Barriers to Novel Data Linkages: Topic Modeling of the Results of the LifeInfo Survey. J Med Internet Res. 2021 May 17;23(5):e24236. doi: 10.2196/24236. PMID: 33998998; PMCID: PMC8167605.

1273: Macey R, Walsh T, Riley P, Glenny AM, Worthington HV, Clarkson JE, Ricketts D. Electrical conductance for the detection of dental caries. Cochrane Database Syst Rev. 2021 Mar 16;3(3):CD014547. doi: 10.1002/14651858.CD014547. PMID: 33724442; PMCID: PMC8406820.

1274: He J, Huang JF, Li C, Chen J, Lu X, Chen JC, He H, Li JX, Cao J, Chen CS, Bazzano LA, Hu D, Kelly TN, Gu DF. Sodium Sensitivity, Sodium Resistance, and Incidence of Hypertension: A Longitudinal Follow-Up Study of Dietary Sodium Intervention. Hypertension. 2021 Jul;78(1):155-164. doi: 10.1161/HYPERTENSIONAHA.120.16758. Epub 2021 Apr 26. PMID: 33896191; PMCID: PMC8192427.

1275: Chintam K, Chang AR. Strategies to Treat Obesity in Patients With CKD. Am

J Kidney Dis. 2021 Mar;77(3):427-439. doi: 10.1053/j.ajkd.2020.08.016. Epub 2020 Oct 16. PMID: 33075388; PMCID: PMC7904606.

1276: Hasan SMM, Rahman M, Nakamura K, Tashiro Y, Miyashita A, Seino K. Relationship between diabetes self-care practices and control of periodontal disease among type2 diabetes patients in Bangladesh. PLoS One. 2021 Apr 6;16(4):e0249011. doi: 10.1371/journal.pone.0249011. Erratum in: PLoS One. 2021 Apr 20;16(4):e0250683. PMID: 33822810; PMCID: PMC8023471.

1277: Kalveram L, Gohlisch J, Brauchmann J, Overberg J, Kühnen P, Wiegand S. Gustatory Function Can Improve after Multimodal Lifestyle Intervention: A Longitudinal Observational Study in Pediatric Patients with Obesity. Child Obes. 2021 Mar;17(2):136-143. doi: 10.1089/chi.2020.0318. Epub 2021 Feb 1. PMID: 33524304.

1278: Zhang X, Fang YJ, Feng XL, Abulimiti A, Huang CY, Luo H, Zhang CX. Interactions Between Vitamin D and Calcium Intake, Vitamin D Receptor Genetic Polymorphisms, and Colorectal Cancer Risk. Dig Dis Sci. 2021 Jun;66(6):1895-1905. doi: 10.1007/s10620-020-06455-4. Epub 2020 Jul 5. PMID: 32627088.

1279: Cheikh Ismail L, Osaili TM, Mohamad MN, Al Marzouqi A, Jarrar AH, Zampelas A, Habib-Mourad C, Omar Abu Jamous D, Ali HI, Al Sabbah H, Hasan H, AlMarzooqi LMR, Stojanovska L, Hashim M, Shaker Obaid RR, ElFeky S, Saleh ST, Shawar ZAM, Al Dhaheri AS. Assessment of eating habits and lifestyle during the coronavirus 2019 pandemic in the Middle East and North Africa region: a cross-sectional study. Br J Nutr. 2021 Sep 14;126(5):757-766. doi: 10.1017/S0007114520004547. Epub 2020 Nov 17. PMID: 33198840; PMCID: PMC7804075.

1280: Sestini S, Paneghetti L, Lampe C, Betti G, Bond S, Bellettato CM, Maurizio S. Social and medical needs of rare metabolic patients: results from a MetabERN survey. Orphanet J Rare Dis. 2021 Aug 3;16(1):336. doi: 10.1186/s13023-021-01948-5. PMID: 34344397; PMCID: PMC8329639.

1281: Sathkumara HD, Eaton JL, Field MA, Govan BL, Ketheesan N, Kupz A. A murine model of tuberculosis/type 2 diabetes comorbidity for investigating the microbiome, metabolome and associated immune parameters. Animal Model Exp Med. 2021 Mar 23;4(2):181-188. doi: 10.1002/ame2.12159. PMID: 34179725; PMCID: PMC8212822.

1282: Holmes CJ, Racette SB. The Utility of Body Composition Assessment in Nutrition and Clinical Practice: An Overview of Current Methodology. Nutrients. 2021 Jul 22;13(8):2493. doi: 10.3390/nu13082493. PMID: 34444653; PMCID: PMC8399582.

1283: Mariani J, Giménez VMM, Bergam I, Tajer C, Antonietti L, Inserra F, Ferder L, Manucha W. Association Between Vitamin D Deficiency and COVID-19 Incidence, Complications, and Mortality in 46 Countries: An Ecological Study. Health Secur. 2021 May-Jun;19(3):302-308. doi: 10.1089/hs.2020.0137. Epub 2020 Dec 14. PMID:

33325788.

1284: Manoogian ENC, Wei-Shatzel J, Panda S. Assessing temporal eating pattern in free living humans through the myCircadianClock app. Int J Obes (Lond). 2022 Jan 8. doi: 10.1038/s41366-021-01038-3. Epub ahead of print. PMID: 34997205.

1285: Verduci E, Carbone MT, Fiori L, Gualdi C, Banderali G, Carducci C, Leuzzi V, Biasucci G, Zuccotti GV. Creatine Levels in Patients with Phenylketonuria and Mild Hyperphenylalaninemia: A Pilot Study. Life (Basel). 2021 May 6;11(5):425. doi: 10.3390/life11050425. PMID: 34066566; PMCID: PMC8148514.

1286: Chan CY, Chiu CY. Disordered eating behaviors and psychological health during the COVID-19 pandemic. Psychol Health Med. 2022 Jan;27(1):249-256. doi: 10.1080/13548506.2021.1883687. Epub 2021 Feb 9. PMID: 33559483.

1287: Diouf JBN, Sougou NM. Vitamin A Supplementation in Children in Guédiawaye Health District, Senegal. Indian Pediatr. 2021 Nov 15;58(11):1094-1095. PMID: 34837369.

1288: Carasso S, Fishman B, Lask LS, Shochat T, Geva-Zatorsky N, Tauber E. Metagenomic analysis reveals the signature of gut microbiota associated with human chronotypes. FASEB J. 2021 Nov;35(11):e22011. doi: 10.1096/fj.202100857RR. PMID: 34695305.

1289: Sinopoulou V, Gordon M, Akobeng AK, Gasparetto M, Sammaan M, Vasiliou J, Dovey TM. Interventions for the management of abdominal pain in Crohn's disease and inflammatory bowel disease. Cochrane Database Syst Rev. 2021 Nov 29;11(11):CD013531. doi: 10.1002/14651858.CD013531.pub2. PMID: 34844288; PMCID: PMC8629648.

1290: Sugiura T, Takase H, Ohte N, Dohi Y. Dietary salt intake increases with age in Japanese adults. Nutr Res. 2021 May;89:1-9. doi: 10.1016/j.nutres.2021.02.002. Epub 2021 Mar 21. PMID: 33866192.

1291: Mitchell CM, Davy BM, Ponder MA, McMillan RP, Hughes MD, Hulver MW, Neilson AP, Davy KP. Prebiotic Inulin Supplementation and Peripheral Insulin Sensitivity in adults at Elevated Risk for Type 2 Diabetes: A Pilot Randomized Controlled Trial. Nutrients. 2021 Sep 17;13(9):3235. doi: 10.3390/nu13093235. PMID: 34579112; PMCID: PMC8471706.

1292: Moleyar-Narayana P, Ranganathan S. Cancer Screening. 2021 Oct 9. In: StatPearls [Internet]. Treasure Island (FL): StatPearls Publishing; 2022 Jan–. PMID: 33085285.

1293: Gallè F, Sabella EA, Roma P, Ferracuti S, Da Molin G, Diella G, Montagna MT, Orsi GB, Liguori G, Napoli C. Knowledge and Lifestyle Behaviors Related to COVID-19 Pandemic in People over 65 Years Old from Southern Italy. Int J Environ Res Public Health. 2021 Oct 16;18(20):10872. doi: 10.3390/ijerph182010872. PMID: 34682618; PMCID: PMC8535846.

1294: Perrar I, Buyken AE, Penczynski KJ, Remer T, Kuhnle GG, Herder C, Roden M, Della Corte K, Nöthlings U, Alexy U. Relevance of fructose intake in adolescence for fatty liver indices in young adulthood. Eur J Nutr. 2021 Sep;60(6):3029-3041. doi: 10.1007/s00394-020-02463-2. Epub 2021 Jan 19. PMID: 33464363; PMCID: PMC8354997.

1295: Busquets-Cortés C, Bennasar-Veny M, López-González AA, Fresneda S, Aguiló A, Yanez A. Fatty liver index and progression to type 2 diabetes: a 5-year longitudinal study in Spanish workers with pre-diabetes. BMJ Open. 2021 Aug 25;11(8):e045498. doi: 10.1136/bmjopen-2020-045498. PMID: 34433590; PMCID: PMC8388308.

1296: Yang ZY, Zhang Q, Zhai Y, Xu T, Wang YY, Chen BW, Tang XJ, Yuan XL, Fang HY, Zhu Y, Pang XH, Wang S, Xu J, Li RL, Si X, Zhao WH. National Nutrition and Health Systematic Survey for Children 0-17 Years of Age in China. Biomed Environ Sci. 2021 Nov 20;34(11):891-899. doi: 10.3967/bes2021.122. PMID: 34955149.

1297: Ougier E, Fiore K, Rousselle C, Assunção R, Martins C, Buekers J. Burden of osteoporosis and costs associated with human biomonitored cadmium exposure in three European countries: France, Spain and Belgium. Int J Hyg Environ Health. 2021 May;234:113747. doi: 10.1016/j.ijheh.2021.113747. Epub 2021 Apr 13. PMID: 33862487.

1298: Minamino H, Katsushima M, Hashimoto M, Fujita Y, Torii M, Ikeda K, Isomura N, Oguri Y, Yamamoto W, Watanabe R, Murakami K, Murata K, Nishitani K, Tanaka M, Ito H, Uda M, Nin K, Arai H, Matsuda S, Morinobu A, Inagaki N. Influence of dietary habits on depression among patients with rheumatoid arthritis: A cross-sectional study using KURAMA cohort database. PLoS One. 2021 Aug 5;16(8):e0255526. doi: 10.1371/journal.pone.0255526. PMID: 34351967; PMCID: PMC8341538.

1299: Gil Á, Urrialde R, Varela-Moreiras G. Posicionamiento sobre la definición de azúcares añadidos y su declaración en el etiquetado de los productos alimenticios en España [Position statement on the definition of added sugars and their declaration on the labelling of foodstuffs in Spain]. Nutr Hosp. 2021 Jun 10;38(3):645-660. Spanish. doi: 10.20960/nh.03493. PMID: 33720744.

1300: Alongi M, Verardo G, Gorassini A, Sillani S, Degrassi C, Anese M. Reformulation and food combination as strategies to modulate glycaemia: the case of apple pomace containing biscuits administered with apple juice to healthy rats. Int J Food Sci Nutr. 2021 Mar;72(2):174-183. doi: 10.1080/09637486.2020.1786025. Epub 2020 Jun 28. PMID: 32597255.

1301: Lee ME, Ali H, Staggers KA, Harpavat M, Natarajan Y. What's in Your Drink? Poster Educates Families About Sugar Content and Fatty Liver Disease. J Nutr Educ Behav. 2021 Oct;53(10):851-857. doi: 10.1016/j.jneb.2021.04.463. Epub 2021 Aug 6. PMID: 34366233.

1302: Sari DW, Noguchi-Watanabe M, Sasaki S, Sahar J, Yamamoto-Mitani N. Estimation of sodium and potassium intakes assessed by two 24-hour urine collections in a city of Indonesia. Br J Nutr. 2021 Nov 28;126(10):1537-1548. doi: 10.1017/S0007114521000271. Epub 2021 Jan 26. PMID: 33494843; PMCID: PMC8524422.

1303: Pham VT, Calatayud M, Rotsaert C, Seifert N, Richard N, Van den Abbeele P, Marzorati M, Steinert RE. Antioxidant Vitamins and Prebiotic FOS and XOS Differentially Shift Microbiota Composition and Function and Improve Intestinal Epithelial Barrier In Vitro. Nutrients. 2021 Mar 29;13(4):1125. doi: 10.3390/nu13041125. PMID: 33805552; PMCID: PMC8066074.

1304: Abbate JM, Macrì F, Arfuso F, Iaria C, Capparucci F, Anfuso C, Ieni A, Cicero L, Briguglio G, Lanteri G. Anti-Atherogenic Effect of 10% Supplementation of Anchovy (<i>Engraulis encrasicolus</i>) Waste Protein Hydrolysates in ApoE-Deficient Mice. Nutrients. 2021 Jun 22;13(7):2137. doi: 10.3390/nu13072137. PMID: 34206655; PMCID: PMC8308468.

1305: Taherifard E, Moradian MJ, Taherifard E, Hemmati A, Rastegarfar B, Molavi Vardanjani H. The prevalence of risk factors associated with non-communicable diseases in Afghan refugees in southern Iran: a cross-sectional study. BMC Public Health. 2021 Mar 5;21(1):442. doi: 10.1186/s12889-021-10482-9. PMID: 33663464; PMCID: PMC7934241.

1306: Harrington K, Zenk SN, Van Horn L, Giurini L, Mahakala N, Kershaw KN. The Use of Food Images and Crowdsourcing to Capture Real-time Eating Behaviors: Acceptability and Usability Study. JMIR Form Res. 2021 Dec 2;5(12):e27512. doi: 10.2196/27512. PMID: 34860666; PMCID: PMC8686467.

1307: So H, Park D, Choi MK, Kim YS, Shin MJ, Park YK. Development and Validation of a Food Literacy Assessment Tool for Community-Dwelling Elderly People. Int J Environ Res Public Health. 2021 May 7;18(9):4979. doi: 10.3390/ijerph18094979. PMID: 34067139; PMCID: PMC8124382.

1308: Gao M, Chen W, Dong S, Chen Y, Zhang Q, Sun H, Zhang Y, Wu W, Pan Z, Gao S, Lin L, Shen J, Tan L, Wang G, Zhang W. Assessing the impact of drinking water iodine concentrations on the iodine intake of Chinese pregnant women living in areas with restricted iodized salt supply. Eur J Nutr. 2021 Mar;60(2):1023-1030. doi: 10.1007/s00394-020-02308-y. Epub 2020 Jun 23. PMID: 32577887.

1309: Bondonno CP, Dalgaard F, Blekkenhorst LC, Murray K, Lewis JR, Croft KD, Kyrø C, Torp-Pedersen C, Gislason G, Tjønneland A, Overvad K, Bondonno NP, Hodgson JM. Vegetable nitrate intake, blood pressure and incident cardiovascular disease: Danish Diet, Cancer, and Health Study. Eur J Epidemiol. 2021 Aug;36(8):813-825. doi: 10.1007/s10654-021-00747-3. Epub 2021 Apr 21. PMID: 33884541; PMCID: PMC8416839.

1310: Cormick G, Ciapponi A, Cafferata ML, Cormick MS, Belizán JM. Calcium supplementation for prevention of primary hypertension. Cochrane Database Syst

Rev. 2022 Jan 11;1(1):CD010037. doi: 10.1002/14651858.CD010037.pub4. PMID: 35014026; PMCID: PMC8748265.

1311: Nagieb CS, Harhash TA, Fayed HL, Ali S. Evaluation of diode laser versus topical corticosteroid in management of Behcet's disease-associated oral ulcers: a randomized clinical trial. Clin Oral Investig. 2022 Jan;26(1):697-704. doi: 10.1007/s00784-021-04047-8. Epub 2021 Jul 2. PMID: 34212234.

1312: Bondyra-Wiśniewska B, Myszkowska-Ryciak J, Harton A. Impact of Lifestyle Intervention Programs for Children and Adolescents with Overweight or Obesity on Body Weight and Selected Cardiometabolic Factors-A Systematic Review. Int J Environ Res Public Health. 2021 Feb 20;18(4):2061. doi: 10.3390/ijerph18042061. PMID: 33672502; PMCID: PMC7923753.

1313: Khadilkar V, Shah N. Evaluation of Children and Adolescents with Obesity. Indian J Pediatr. 2021 Dec;88(12):1214-1221. doi: 10.1007/s12098-021-03893-4. Epub 2021 Aug 18. PMID: 34406593.

1314: Santos JA, Tekle D, Rosewarne E, Flexner N, Cobb L, Al-Jawaldeh A, Kim WJ, Breda J, Whiting S, Campbell N, Neal B, Webster J, Trieu K. A Systematic Review of Salt Reduction Initiatives Around the World: A Midterm Evaluation of Progress Towards the 2025 Global Non-Communicable Diseases Salt Reduction Target. Adv Nutr. 2021 Oct 1;12(5):1768-1780. doi: 10.1093/advances/nmab008. PMID: 33693460; PMCID: PMC8483946.

1315: Liu X, Zhang H, Tian Y, Fang M, Xu L, Wang Q, Li J, Shen H, Wu Y, Gong Z. Bioavailability Evaluation of Perchlorate in Different Foods <i>In Vivo</i>: Comparison with <i>In Vitro</i> Assays and Implications for Human Health Risk Assessment. J Agric Food Chem. 2021 May 5;69(17):5189-5197. doi: 10.1021/acs.jafc.1c00539. Epub 2021 Apr 21. PMID: 33881845.

1316: Enikuomehin AC, Adejumo OA, Akinbodewa AA, Muhammad FY, Lawal OM, Junaid OA. Type 2 diabetes mellitus risk assessment among doctors in Ondo state. Malawi Med J. 2021 Jun;33(2):114-120. doi: 10.4314/mmj.v33i2.6. PMID: 34777706; PMCID: PMC8560352.

1317: Sivasubramaniyam T, Yang J, Cheng HS, Zyla A, Li A, Besla R, Dotan I, Revelo XS, Shi SY, Le H, Schroer SA, Dodington DW, Park YJ, Kim MJ, Febbraro D, Ruel I, Genest J, Kim RH, Mak TW, Winer DA, Robbins CS, Woo M. Dj1 deficiency protects against atherosclerosis with anti-inflammatory response in macrophages. Sci Rep. 2021 Feb 25;11(1):4723. doi: 10.1038/s41598-021-84063-6. PMID: 33633277; PMCID: PMC7907332.

1318: Alfhili MA, Aljuraiban GS. Lauric Acid, a Dietary Saturated Medium-Chain Fatty Acid, Elicits Calcium-Dependent Eryptosis. Cells. 2021 Dec 1;10(12):3388. doi: 10.3390/cells10123388. PMID: 34943896; PMCID: PMC8699421.

1319: Esfandiari Z, Mirlohi M, Tanha JM, Hadian M, Mossavi SI, Ansariyan A, Ghassami N, Adib S, Bahraini T, Safaeian L, Pakmehr F, Mashhadian F, Abolhasani

M, Marasi MR, Isteki F, Abedi R, Ghorbani P, Shoaei P, Kelishadi R. Effect of Face-to-Face Education on Knowledge, Attitudes, and Practices Toward "Traffic Light" Food Labeling in Isfahan Society, Iran. Int Q Community Health Educ. 2021 Apr;41(3):275-284. doi: 10.1177/0272684X20916612. Epub 2020 Apr 6. PMID: 32252587.

1320: Thacher TD, Sempos CT, Durazo-Arvizu RA, Fischer PR, Munns CF, Pettifor JM. The Validity of Serum Alkaline Phosphatase to Identify Nutritional Rickets in Nigerian Children on a Calcium-Deprived Diet. J Clin Endocrinol Metab. 2021 Aug 18;106(9):e3559-e3564. doi: 10.1210/clinem/dgab328. PMID: 33982091.

1321: Tian W, Yan W, Liu Y, Zhou F, Wang H, Sun W. The Status and Knowledge of Iodine among Pregnant Women in Shanghai. Biol Trace Elem Res. 2021 Dec;199(12):4489-4497. doi: 10.1007/s12011-021-02587-4. Epub 2021 Jan 18. PMID: 33462796.

1322: Turner G, Green R, Alae-Carew C, Dangour AD. The association of dimensions of fruit and vegetable access in the retail food environment with consumption; a systematic review. Glob Food Sec. 2021 Jun;29:100528. doi: 10.1016/j.gfs.2021.100528. PMID: 34164256; PMCID: PMC8202327.

1323: Setyaningsih WAW, Sari DCR, Romi MM, Arfian N. Liver fibrosis associated with adipose tissue and liver inflammation in an obesity model. Med J Malaysia. 2021 May;76(3):304-310. PMID: 34031327.

1324: Rosales Chávez JB, Jehn M, Lee RE, Ohri-Vachaspati P, Ortiz-Hernandez L, Romo-Aviles M, Bruening M. Development and Interrater Reliability of a Street Food Stand Assessment Tool. J Nutr Educ Behav. 2021 Dec;53(12):1072-1080. doi: 10.1016/j.jneb.2021.06.015. Epub 2021 Aug 3. PMID: 34362667.

1325: Willerslev-Olsen M, Lorentzen J, Røhder K, Ritterband-Rosenbaum A, Justiniano M, Guzzetta A, Lando AV, Jensen AB, Greisen G, Ejlersen S, Pedersen LZ, Andersen B, Lipthay Behrend P, Nielsen JB. COpenhagen Neuroplastic TRaining Against Contractures in Toddlers (CONTRACT): protocol of an open-label randomised clinical trial with blinded assessment for prevention of contractures in infants with high risk of cerebral palsy. BMJ Open. 2021 Jul 6;11(7):e044674. doi: 10.1136/bmjopen-2020-044674. PMID: 34230015; PMCID: PMC8261878.

1326: Doaei S, Bourbour F, Rastgoo S, Akbari ME, Gholamalizadeh M, Hajipour A, Moslem A, Ghorat F, Badeli M, Bagheri SE, Alizadeh A, Mokhtari Z, Pishdad S, JavadiKooshesh S, Azizi Tabesh G, Montazeri F, Joola P, Rezaei S, Dorosti M, Mosavi Jarrahi SA. Interactions of anthropometric indices, rs9939609 FTO gene polymorphism and breast cancer: A case-control study. J Cell Mol Med. 2021 Apr;25(7):3252-3257. doi: 10.1111/jcmm.16394. Epub 2021 Feb 25. PMID: 33634577; PMCID: PMC8034447.

1327: Beck AL, Huang JC, Lendzion L, Fernandez A, Martinez S. Impact of the Coronavirus Disease 2019 Pandemic on Parents' Perception of Health Behaviors in Children With Overweight and Obesity. Acad Pediatr. 2021 Nov-

Dec;21(8):1434-1440. doi: 10.1016/j.acap.2021.05.015. Epub 2021 May 21. PMID: 34023488; PMCID: PMC8561012.

1328: Kwon OY, Jang Y, Lee JY, Kim SU, Ahn SH. Development and initial validation of the nonalcoholic fatty liver disease self-management questionnaire. Res Nurs Health. 2021 Oct;44(5):844-853. doi: 10.1002/nur.22164. Epub 2021 Jun 13. PMID: 34120361.

1329: Pertiwi K, Küpers LK, de Goede J, Zock PL, Kromhout D, Geleijnse JM. Dietary and Circulating Long-Chain Omega-3 Polyunsaturated Fatty Acids and Mortality Risk After Myocardial Infarction: A Long-Term Follow-Up of the Alpha Omega Cohort. J Am Heart Assoc. 2021 Dec 7;10(23):e022617. doi: 10.1161/JAHA.121.022617. Epub 2021 Nov 30. PMID: 34845924.

1330: Cachero K, Granger M, Mollard RC, Askin N, Okoli GN, Abou-Setta AM, MacKay D. Efficacy and safety of clinically managed weight loss programs: a systematic review and meta-analysis protocol. Syst Rev. 2021 Jul 2;10(1):197. doi: 10.1186/s13643-021-01750-1. PMID: 34215334; PMCID: PMC8254293.

1331: Alothman SA, Alghannam AF, Almasud AA, Altalhi AS, Al-Hazzaa HM. Lifestyle behaviors trend and their relationship with fear level of COVID-19: Cross-sectional study in Saudi Arabia. PLoS One. 2021 Oct 13;16(10):e0257904. doi: 10.1371/journal.pone.0257904. PMID: 34644323; PMCID: PMC8513859.

1332: Khan RJ, Needham BL, Advani S, Brown K, Dagnall C, Xu R, Gibbons GH, Davis SK. Association of Childhood Socioeconomic Status with Leukocyte Telomere Length Among African Americans and the Mediating Role of Behavioral and Psychosocial Factors: Results from the GENE-FORECAST Study. J Racial Ethn Health Disparities. 2021 May 4. doi: 10.1007/s40615-021-01040-5. Epub ahead of print. PMID: 33948907.

1333: Bošković M, Živković M, Korićanac G, Stanišić J, Zec M, Krga I, Stanković A. Walnut Supplementation Restores the SIRT1-FoxO3a-MnSOD/Catalase Axis in the Heart, Promotes an Anti-Inflammatory Fatty Acid Profile in Plasma, and Lowers Blood Pressure on Fructose-Rich Diet. Oxid Med Cell Longev. 2021 Apr 21;2021:5543025. doi: 10.1155/2021/5543025. PMID: 33976753; PMCID: PMC8086433.

1334: Mollerup A, Larsen SC, Bennetzen AS, Henriksen M, Simonsen MK, Weis N, Kofod LM, Heitmann BL. PEP-CoV protocol: a PEP flute-self-care randomised controlled trial to prevent respiratory deterioration and hospitalisation in early COVID-19. BMJ Open. 2021 Jun 30;11(6):e050582. doi: 10.1136/bmjopen-2021-050582. PMID: 34193503; PMCID: PMC8249175.

1335: Fernandez-Carbonell C, Charvet LE, Krupp LB. Enhancing Mood, Cognition, and Quality of Life in Pediatric Multiple Sclerosis. Paediatr Drugs. 2021 Jul;23(4):317-329. doi: 10.1007/s40272-021-00451-5. Epub 2021 May 17. PMID: 33997945; PMCID: PMC8275506.

1336: Larson LR, Mullenbach LE, Browning MHEM, Rigolon A, Thomsen J, Metcalf EC,

Reigner NP, Sharaievska I, McAnirlin O, D'Antonio A, Cloutier S, Helbich M, Labib SM. Greenspace and park use associated with less emotional distress among college students in the United States during the COVID-19 pandemic. Environ Res. 2022 Mar;204(Pt D):112367. doi: 10.1016/j.envres.2021.112367. Epub 2021 Nov 10. PMID: 34774510; PMCID: PMC8648327.

1337: Mirzaee F, Ahmadi A. Overview of the Effect of Complementary Medicine on Treating or Mitigating the Risk of Endometriosis. Rev Bras Ginecol Obstet. 2021 Dec;43(12):919-925. English. doi: 10.1055/s-0041-1735156. Epub 2021 Dec 21. PMID: 34933385.

1338: Dekeryte R, Franklin Z, Hull C, Croce L, Kamli-Salino S, Helk O, Hoffmann PA, Yang Z, Riedel G, Delibegovic M, Platt B. The BACE1 inhibitor LY2886721 improves diabetic phenotypes of BACE1 knock-in mice. Biochim Biophys Acta Mol Basis Dis. 2021 Jul 1;1867(7):166149. doi: 10.1016/j.bbadis.2021.166149. Epub 2021 Apr 20. PMID: 33892080.

1339: Deng YY, Zhong QW, Zhong HL, Xiong F, Ke YB, Chen YM. Higher Healthy Lifestyle Score is associated with lower presence of non-alcoholic fatty liver disease in middle-aged and older Chinese adults: a community-based cross-sectional study. Public Health Nutr. 2021 Oct;24(15):5081-5089. doi: 10.1017/S1368980021000902. Epub 2021 Feb 26. PMID: 33634772.

1340: Harmon KK, Stout JR, Fukuda DH, Pabian PS, Rawson ES, Stock MS. The Application of Creatine Supplementation in Medical Rehabilitation. Nutrients. 2021 May 27;13(6):1825. doi: 10.3390/nu13061825. PMID: 34071875; PMCID: PMC8230227.

1341: Troshina EA, Makolina NP, Senyushkina ES, Nikankina LV, Malysheva NM, Fetisova AV. [Iodine Deficiency Disorders: Current State of the Problem in the Bryansk Region]. Probl Endokrinol (Mosk). 2021 Aug 3;67(4):84-93. Russian. doi: 10.14341/probl12793. PMID: 34533016.

1342: Pifferi F, Laurent B, Plourde M. Lipid Transport and Metabolism at the Blood-Brain Interface: Implications in Health and Disease. Front Physiol. 2021 Mar 30;12:645646. doi: 10.3389/fphys.2021.645646. PMID: 33868013; PMCID: PMC8044814.

1343: Zhang S, Miller DD, Li W. Non-Musculoskeletal Benefits of Vitamin D beyond the Musculoskeletal System. Int J Mol Sci. 2021 Feb 21;22(4):2128. doi: 10.3390/ijms22042128. PMID: 33669918; PMCID: PMC7924658.

1344: Song S, Stern Y, Gu Y. Modifiable lifestyle factors and cognitive reserve: A systematic review of current evidence. Ageing Res Rev. 2022 Feb;74:101551. doi: 10.1016/j.arr.2021.101551. Epub 2021 Dec 21. PMID: 34952208; PMCID: PMC8794051.

1345: Sellem L, Srour B, Jackson KG, Hercberg S, Galan P, Kesse-Guyot E, Julia C, Fezeu L, Deschasaux-Tanguy M, Lovegrove J, Touvier M. Consumption of dairy

products and CVD risk: results from the French prospective cohort NutriNet-Santé. Br J Nutr. 2021 Apr 29:1-11. doi: 10.1017/S0007114521001422. Epub ahead of print. PMID: 33910667.

1346: Lafuente M, Rodríguez González-Herrero ME, Romeo Villadóniga S, Domingo JC. Antioxidant Activity and Neuroprotective Role of Docosahexaenoic Acid (DHA) Supplementation in Eye Diseases That Can Lead to Blindness: A Narrative Review. Antioxidants (Basel). 2021 Mar 5;10(3):386. doi: 10.3390/antiox10030386. PMID: 33807538; PMCID: PMC8000043.

1347: Aydın F, Akşit E, Yıldırım ÖT, Hüseyinoğlu Aydın A, Samsa M. Assessment of secondary prevention awareness among patients with coronary artery disease: A survey including patients from 3 centers. Turk Kardiyol Dern Ars. 2021 Oct;49(7):556-567. doi: 10.5543/tkda.2021.32302. PMID: 34623299.

1348: Smith-Ryan AE, Weaver MA, Viera AJ, Weinberger M, Blue MNM, Hirsch KR. Promoting Exercise and Healthy Diet Among Primary Care Patients: Feasibility, Preliminary Outcomes, and Lessons Learned From a Pilot Trial With High Intensity Interval Exercise. Front Sports Act Living. 2021 Jul 16;3:690243. doi: 10.3389/fspor.2021.690243. PMID: 34337406; PMCID: PMC8322607.

1349: Roschel H, Gualano B, Ostojic SM, Rawson ES. Creatine Supplementation and Brain Health. Nutrients. 2021 Feb 10;13(2):586. doi: 10.3390/nu13020586. PMID: 33578876; PMCID: PMC7916590.

1350: Kołodziejczyk-Nowotarska A, Bokiniec R, Seliga-Siwecka J. Monitored Supplementation of Vitamin D in Preterm Infants: A Randomized Controlled Trial. Nutrients. 2021 Sep 28;13(10):3442. doi: 10.3390/nu13103442. PMID: 34684442; PMCID: PMC8537871.

1351: Saneian H, Khalilian L, Heidari-Beni M, Khademian M, Famouri F, Nasri P, Hassanzadeh A, Kelishadi R. Effect of l-carnitine supplementation on children and adolescents with nonalcoholic fatty liver disease (NAFLD): a randomized, triple-blind, placebo-controlled clinical trial. J Pediatr Endocrinol Metab. 2021 May 4;34(7):897-904. doi: 10.1515/jpem-2020-0642. PMID: 33939897.

1352: Liu C, Roth DL, Gottesman RF, Sheehan OC, Blinka MD, Howard VJ, Judd SE, Cushman M. Change in Life's Simple 7 Measure of Cardiovascular Health After Incident Stroke: The REGARDS Study. Stroke. 2021 Mar;52(3):878-886. doi: 10.1161/STROKEAHA.120.030836. Epub 2021 Jan 20. PMID: 33467879; PMCID: PMC7902464.

1353: Romaszko J, Dragańska E, Cymes I, Drozdowski M, Gromadziński L, Glińska-Lewczuk K. Are the levels of uric acid associated with biometeorological conditions? Sci Total Environ. 2021 Dec 7:152020. doi: 10.1016/j.scitotenv.2021.152020. Epub ahead of print. PMID: 35007576.

1354: Bennett JP, Liu YE, Quon BK, Kelly NN, Wong MC, Kennedy SF, Chow DC, Garber AK, Weiss EJ, Heymsfield SB, Shepherd JA. Assessment of clinical measures

of total and regional body composition from a commercial 3-dimensional optical body scanner. Clin Nutr. 2022 Jan;41(1):211-218. doi: 10.1016/j.clnu.2021.11.031. Epub 2021 Dec 7. PMID: 34915272; PMCID: PMC8727542.

1355: Castro Henríquez E. Estudio metabólico en nefrolitiasis: una herramienta subutilizada y cómo implementarla en la práctica clínica [Metabolic Stone assessment: Underused tool and how to implement it on daily practice.]. Arch Esp Urol. 2021 Nov;74(9):823-834. Spanish. PMID: 34726618.

1356: Chao J, Cheng HY, Chang ML, Huang SS, Liao JW, Cheng YC, Peng WH, Pao LH. Gallic Acid Ameliorated Impaired Lipid Homeostasis in a Mouse Model of High-Fat Diet-and Streptozotocin-Induced NAFLD and Diabetes through Improvement of <i>β</i>-oxidation and Ketogenesis. Front Pharmacol. 2021 Feb 12;11:606759. doi: 10.3389/fphar.2020.606759. PMID: 33643038; PMCID: PMC7907449.

1357: Zhu J, Zhao X, Yang M, Zheng B, Sun C, Zou X, Liu Z, Harada KH. Levels of urinary metabolites of benzene compounds, trichloroethylene, and polycyclic aromatic hydrocarbons and their correlations with socioeconomic, demographic, dietary factors among pregnant women in six cities of China. Environ Sci Pollut Res Int. 2022 Jan;29(4):6278-6293. doi: 10.1007/s11356-021-16030-7. Epub 2021 Aug 27. PMID: 34453244.

1358: Mayer SB, Graybill S, Raffa SD, Tracy C, Gaar E, Wisbach G, Goldstein MG, Sall J. Synopsis of the 2020 U.S. VA/DoD Clinical Practice Guideline for the Management of Adult Overweight and Obesity. Mil Med. 2021 Aug 28;186(9-10):884-896. doi: 10.1093/milmed/usab114. PMID: 33904926.

1359: Kohatsu K, Shimizu S, Shibagaki Y, Sakurada T. Association between Daily Urinary Sodium Excretion, Ratio of Extracellular Water-to-Total Body Water Ratio, and Kidney Outcome in Patients with Chronic Kidney Disease. Nutrients. 2021 Feb 17;13(2):650. doi: 10.3390/nu13020650. PMID: 33671239; PMCID: PMC7922304.

1360: She R, Yan Z, Hao Y, Zhang Z, Du Y, Liang Y, Vetrano DL, Dekker J, Bai B, Lau JTF, Qiu C. Health-related quality of life after first-ever acute ischemic stroke: associations with cardiovascular health metrics. Qual Life Res. 2021 Oct;30(10):2907-2917. doi: 10.1007/s11136-021-02853-x. Epub 2021 May 1. PMID: 33932220.

1361: Gao Y, Li J, Cheng FW, Cui L, Shu R, Wu S, Gao X. Poor Sleep Quality Is Associated with Altered Taste Perception in Chinese Adults. J Acad Nutr Diet. 2021 Mar;121(3):435-445. doi: 10.1016/j.jand.2020.06.019. Epub 2020 Aug 19. PMID: 32828739.

1362: Chiu YH, Fadadu RP, Gaskins AJ, Rifas-Shiman SL, Laue HE, Moley KH, Hivert MF, Baccarelli A, Oken E, Chavarro JE, Cardenas A. Dietary fat intake during early pregnancy is associated with cord blood DNA methylation at IGF2 and H19 genes in newborns. Environ Mol Mutagen. 2021 Aug;62(7):388-398. doi: 10.1002/em.22452. Epub 2021 Jul 31. PMID: 34288135; PMCID: PMC8364885.

1363: Tan ST, Tan CX, Tan SS. Changes in Dietary Intake Patterns and Weight Status during the COVID-19 Lockdown: A Cross-Sectional Study Focusing on Young Adults in Malaysia. Nutrients. 2022 Jan 10;14(2):280. doi: 10.3390/nu14020280. PMID: 35057460; PMCID: PMC8778075.

1364: Si J, Li J, Yu C, Guo Y, Bian Z, Millwood I, Yang L, Walters R, Chen Y, Du H, Yin L, Chen J, Chen J, Chen Z, Li L, Liang L, Lv J. Improved lipidomic profile mediates the effects of adherence to healthy lifestyles on coronary heart disease. Elife. 2021 Feb 9;10:e60999. doi: 10.7554/eLife.60999. PMID: 33558007; PMCID: PMC7872516.

1365: Topping M, Kim J, Fletcher J. Geographic variation in Alzheimer's disease mortality. PLoS One. 2021 Jul 1;16(7):e0254174. doi: 10.1371/journal.pone.0254174. PMID: 34197566; PMCID: PMC8248693.

1366: Bashyam A, Frangieh CJ, Raigani S, Sogo J, Bronson RT, Uygun K, Yeh H, Ausiello DA, Cima MJ. A portable single-sided magnetic-resonance sensor for the grading of liver steatosis and fibrosis. Nat Biomed Eng. 2021 Mar;5(3):240-251. doi: 10.1038/s41551-020-00638-0. Epub 2020 Nov 30. PMID: 33257853.

1367: Wu Z, Broad J, Sluyter J, Waayer D, Camargo CA Jr, Scragg R. Effect of monthly vitamin D on diverticular disease hospitalization: Post-hoc analysis of a randomized controlled trial. Clin Nutr. 2021 Mar;40(3):839-843. doi: 10.1016/j.clnu.2020.08.030. Epub 2020 Aug 31. PMID: 32919816.

1368: Battino M, Giampieri F, Cianciosi D, Ansary J, Chen X, Zhang D, Gil E, Forbes-Hernández T. The roles of strawberry and honey phytochemicals on human health: A possible clue on the molecular mechanisms involved in the prevention of oxidative stress and inflammation. Phytomedicine. 2021 Jun;86:153170. doi: 10.1016/j.phymed.2020.153170. Epub 2020 Jan 11. PMID: 31980299.

1369: McHugh C, Hind K, Wyse J, Davey D, Wilson F. Increases in DXA-Derived Visceral Fat Across One Season in Professional Rugby Union Players: Importance of Visceral Fat Monitoring in Athlete Body Composition Assessment. J Clin Densitom. 2021 Apr-Jun;24(2):206-213. doi: 10.1016/j.jocd.2020.09.001. Epub 2020 Sep 18. PMID: 33023826.

1370: Yakut K, Öcal DF, Öztürk FH, Öztürk M, Oğuz Y, Sınacı S, Çağlar T. Is GDF-15 level associated with gestational diabetes mellitus and adverse perinatal outcomes? Taiwan J Obstet Gynecol. 2021 Mar;60(2):221-224. doi: 10.1016/j.tjog.2020.12.004. PMID: 33678319.

1371: Michelogiannakis D, Gajendra S, Pathagunti SR, Sayers MS, Newton JT, Zhou Z, Feng C, Rossouw PE. Patients' and parents' expectations of orthodontic treatment in university settings. Am J Orthod Dentofacial Orthop. 2021 Apr;159(4):443-452. doi: 10.1016/j.ajodo.2020.02.009. Epub 2021 Feb 7. PMID: 33568276.

1372: Adamska-Tomaszewska D, Kocełak P, Owczarek AJ, Olszanecka-Glinianowicz M, Chudek J. Factors affecting vitamin D status in outpatients with abdominal aortic aneurysm and peripheral artery disease- a single centre study. Nutr Metab Cardiovasc Dis. 2021 Oct 28;31(11):3161-3166. doi: 10.1016/j.numecd.2021.07.013. Epub 2021 Jul 24. PMID: 34518086.

1373: Tadipatri R, Lyon K, Azadi A, Fonkem E. A view of the epidemiologic landscape: how population-based studies can lend novel insights regarding the pathophysiology of glioblastoma. Chin Clin Oncol. 2021 Aug;10(4):35. doi: 10.21037/cco.2020.02.07. Epub 2020 Apr 8. PMID: 32279523.

1374: Bosi Bağcı TA, Kanadıkırık A, Somyürek E, Gerçek G, Tanrıkulu HB, Öntaş E, Uzun S. Impact of COVID-19 on eating habits, sleeping behaviour and physical activity status of final-year medical students in Ankara, Turkey. Public Health Nutr. 2021 Dec;24(18):6369-6376. doi: 10.1017/S1368980021003906. Epub 2021 Sep 9. PMID: 34496994; PMCID: PMC8505814.

1375: Smith AD, Fan A, Qin B, Desai N, Zhao A, Shea-Donohue T. IL-25 Treatment Improves Metabolic Syndrome in High-Fat Diet and Genetic Models of Obesity. Diabetes Metab Syndr Obes. 2021 Dec 21;14:4875-4887. doi: 10.2147/DMSO.S335761. PMID: 34992396; PMCID: PMC8710075.

1376: Caruso I, Di Molfetta S, Guarini F, Giordano F, Cignarelli A, Natalicchio A, Perrini S, Leonardini A, Giorgino F, Laviola L. Reduction of hypoglycaemia, lifestyle modifications and psychological distress during lockdown following SARS-CoV-2 outbreak in type 1 diabetes. Diabetes Metab Res Rev. 2021 Sep;37(6):e3404. doi: 10.1002/dmrr.3404. Epub 2020 Oct 5. PMID: 32918324.

1377: Lee JH, Han JH, Jung EJ, Nallapaneni HK, Kim NS, Kim H, Lee J, Baek SY. Development and validation of liquid chromatography-tandem mass spectrometry method for screening six selective androgen receptor modulators in dietary supplements. Food Addit Contam Part A Chem Anal Control Expo Risk Assess. 2021 Jul;38(7):1075-1086. doi: 10.1080/19440049.2021.1906954. Epub 2021 May 1. PMID: 33934684.

1378: Silva LK, Espenship MF, Newman CA, Zhang L, Zhu W, Blount BC, De Jesús VR. Assessment of Serum Concentrations of 12 Aldehydes in the U.S. Population from the 2013-2014 National Health and Nutrition Examination Survey. Environ Sci Technol. 2021 Apr 20;55(8):5076-5083. doi: 10.1021/acs.est.0c07294. Epub 2021 Apr 1. PMID: 33793230.

1379: Moghtaderi F, Amiri M, Zimorovat A, Raeisi-Dehkordi H, Rahmanian M, Hosseinzadeh M, Fallahzadeh H, Salehi-Abargouei A. The effect of canola, sesame and sesame-canola oils on body fat and composition in adults: a triple-blind, three-way randomised cross-over clinical trial. Int J Food Sci Nutr. 2021 Mar;72(2):226-235. doi: 10.1080/09637486.2020.1786024. Epub 2020 Jul 20. PMID: 32684099.

1380: Chao SM, Yen M, Lin HS, Sung JM, Hung SY, Natashia D. Effects of helping

relationships on health-promoting lifestyles among patients with chronic kidney disease: A randomized controlled trial. Int J Nurs Stud. 2022 Feb;126:104137. doi: 10.1016/j.ijnurstu.2021.104137. Epub 2021 Nov 22. PMID: 34890837.

1381: Rodríguez-Carrillo A, Rosenmai AK, Mustieles V, Couderq S, Fini JB, Vela-Soria F, Molina-Molina JM, Ferrando-Marco P, Wielsøe M, Long M, Bonefeld-Jorgensen EC, Olea N, Vinggaard AM, Fernández MF. Assessment of chemical mixtures using biomarkers of combined biological activity: A screening study in human placentas. Reprod Toxicol. 2021 Mar;100:143-154. doi: 10.1016/j.reprotox.2021.01.002. Epub 2021 Jan 11. PMID: 33444715.

1382: Jahan S, Mahmud MH, Khan Z, Alam A, Khalil AA, Rauf A, Tareq AM, Nainu F, Tareq SM, Emran TB, Khan M, Khan IN, Wilairatana P, Mubarak MS. Health promoting benefits of pongamol: An overview. Biomed Pharmacother. 2021 Oct;142:112109. doi: 10.1016/j.biopha.2021.112109. Epub 2021 Aug 27. PMID: 34470730.

1383: Jiang YW, Zhang YB, Pan A. [Consumption of sugar-sweetened beverages and artificially sweetened beverages and risk of cardiovascular disease: a meta-analysis]. Zhonghua Yu Fang Yi Xue Za Zhi. 2021 Sep 6;55(9):1159-1167. Chinese. doi: 10.3760/cma.j.cn112150-20210729-00726. PMID: 34619938.

1384: Wedekind LE, Mitchell CM, Andersen CC, Knowler WC, Hanson RL. Epidemiology of Type 2 Diabetes in Indigenous Communities in the United States. Curr Diab Rep. 2021 Nov 22;21(11):47. doi: 10.1007/s11892-021-01406-3. PMID: 34807308; PMCID: PMC8665733.

1385: Cárdenas-Fuentes G, Homs C, Ramírez-Contreras C, Juton C, Casas-Esteve R, Grau M, Aguilar-Palacio I, Fitó M, Gomez SF, Schröder H. Prospective Association of Maternal Educational Level with Child's Physical Activity, Screen Time, and Diet Quality. Nutrients. 2021 Dec 30;14(1):160. doi: 10.3390/nu14010160. PMID: 35011035; PMCID: PMC8747409.

1386: White MJ, Holliday KM, Hoover S, Robinson-Ezekwe N, Corbie-Smith G, Williams A, Bess K, Frerichs L. The significant places of African American adults and their perceived influence on cardiovascular disease risk behaviors. BMC Public Health. 2021 Nov 5;21(1):2018. doi: 10.1186/s12889-021-12022-x. PMID: 34740336; PMCID: PMC8570769.

1387: Ashtary-Larky D, Bagheri R, Tinsley GM, Asbaghi O, Salehpour S, Kashkooli S, Kooti W, Wong A. Betaine supplementation fails to improve body composition: a systematic review and meta-analysis. Br J Nutr. 2021 Oct 7:1-14. doi: 10.1017/S0007114521004062. Epub ahead of print. PMID: 34743773.

1388: Cruijsen E, de Ruiter AJ, Küpers LK, Busstra MC, Geleijnse JM. Alcohol intake and long-term mortality risk after myocardial infarction in the Alpha Omega Cohort. Am J Clin Nutr. 2021 Nov 3:nqab366. doi: 10.1093/ajcn/nqab366. Epub ahead of print. PMID: 34734223.

1389: Hose AJ, Pagani G, Karvonen AM, Kirjavainen PV, Roduit C, Genuneit J,

Schmaußer-Hechfellner E, Depner M, Frei R, Lauener R, Riedler J, Schaub B, Fuchs O, von Mutius E, Divaret-Chauveau A, Pekkanen J, Ege MJ. Excessive Unbalanced Meat Consumption in the First Year of Life Increases Asthma Risk in the PASTURE and LUKAS2 Birth Cohorts. Front Immunol. 2021 Apr 27;12:651709. doi: 10.3389/fimmu.2021.651709. PMID: 33986744; PMCID: PMC8111016.

1390: Baghban F, Hosseinzadeh M, Mozaffari-Khosravi H, Dehghan A, Fallahzadeh H. The effect of L-Carnitine supplementation on clinical symptoms, C-reactive protein and malondialdehyde in obese women with knee osteoarthritis: a double blind randomized controlled trial. BMC Musculoskelet Disord. 2021 Feb 17;22(1):195. doi: 10.1186/s12891-021-04059-1. PMID: 33596883; PMCID: PMC7891026.

1391: Shahril MR, Zakarai NS, Appannah G, Nurnazahiah A, Mohamed HJJ, Ahmad A, Lua PL, Fenech M. 'Energy-Dense, High-SFA and Low-Fiber' Dietary Pattern Lowered Adiponectin but Not Leptin Concentration of Breast Cancer Survivors. Nutrients. 2021 Sep 24;13(10):3339. doi: 10.3390/nu13103339. PMID: 34684340; PMCID: PMC8540181.

1392: Zanobini P, Lorini C, Lastrucci V, Minardi V, Possenti V, Masocco M, Garofalo G, Mereu G, Bonaccorsi G. Health Literacy, Socio-Economic Determinants, and Healthy Behaviours: Results from a Large Representative Sample of Tuscany Region, Italy. Int J Environ Res Public Health. 2021 Nov 26;18(23):12432. doi: 10.3390/ijerph182312432. PMID: 34886157; PMCID: PMC8656600.

1393: Ajime TT, Serré J, Wüst RCI, Burniston JG, Maes K, Janssens W, Troosters T, Gayan-Ramirez G, Degens H. The combination of smoking with vitamin D deficiency impairs skeletal muscle fiber hypertrophy in response to overload in mice. J Appl Physiol (1985). 2021 Jul 1;131(1):339-351. doi: 10.1152/japplphysiol.00733.2020. Epub 2021 Jun 3. PMID: 34080919.

1394: Stratakis N, Siskos AP, Papadopoulou E, Nguyen AN, Zhao Y, Margetaki K, Lau CE, Coen M, Maitre L, Fernández-Barrés S, Agier L, Andrusaityte S, Basagaña X, Brantsaeter AL, Casas M, Fossati S, Grazuleviciene R, Heude B, McEachan RR, Meltzer HM, Millett C, Rauber F, Robinson O, Roumeliotaki T, Borras E, Sabidó E, Urquiza J, Vafeiadi M, Vineis P, Voortman T, Wright J, Conti DV, Vrijheid M, Keun HC, Chatzi L. Urinary metabolic biomarkers of diet quality in European children are associated with metabolic health. Elife. 2022 Jan 25;11:e71332. doi: 10.7554/eLife.71332. PMID: 35076016; PMCID: PMC8789316.

1395: Ebrahimzadeh A, Abbasi F, Ebrahimzadeh A, Jibril AT, Milajerdi A. Effects of curcumin supplementation on inflammatory biomarkers in patients with Rheumatoid Arthritis and Ulcerative colitis: A systematic review and meta-analysis. Complement Ther Med. 2021 Sep;61:102773. doi: 10.1016/j.ctim.2021.102773. Epub 2021 Aug 31. PMID: 34478838.

1396: Heise M, Fink A, Baumert J, Heidemann C, Du Y, Frese T, Carmienke S. Patterns and associated factors of diabetes self-management: Results of a latent class analysis in a German population-based study. PLoS One. 2021 Mar

19;16(3):e0248992. doi: 10.1371/journal.pone.0248992. PMID: 33740024; PMCID: PMC7978380.

1397: Zoellner JM, Porter KJ, You W, Reid AL, Frederick C, Hilgart M, Brock DP, Tate DF, Ritterband LM. Study protocol for iSIPsmarter: A randomized-controlled trial to evaluate the efficacy, reach, and engagement of a technology-based behavioral intervention to reduce sugary beverages among rural Appalachian adults. Contemp Clin Trials. 2021 Nov;110:106566. doi: 10.1016/j.cct.2021.106566. Epub 2021 Sep 4. PMID: 34492306; PMCID: PMC8595813.

1398: Olasinde YT, Idowu A, Olasinde A, Aremu AO, Ogunlaja OA. Infant and Young Child Feeding Practices among Mothers of Children 6-23 Months Old Attending the Immunisation Clinic of a Private Tertiary Health Institution in Nigeria. West Afr J Med. 2021 May 29;38:445-453. PMID: 34051716.

1399: Moghadam BH, Bagheri R, Roozbeh B, Ashtary-Larky D, Gaeini AA, Dutheil F, Wong A. Impact of saffron (Crocus Sativus Linn) supplementation and resistance training on markers implicated in depression and happiness levels in untrained young males. Physiol Behav. 2021 May 1;233:113352. doi: 10.1016/j.physbeh.2021.113352. Epub 2021 Feb 6. PMID: 33556410.

1400: Fengler VH, Macheiner T, Goessler W, Ratzer M, Haybaeck J, Sargsyan K. Hepatic Response of Magnesium-Restricted Wild Type Mice. Metabolites. 2021 Nov 6;11(11):762. doi: 10.3390/metabo11110762. PMID: 34822420; PMCID: PMC8625093.

1401: Koopaie M, Salamati M, Montazeri R, Davoudi M, Kolahdooz S. Salivary cystatin S levels in children with early childhood caries in comparison with caries-free children; statistical analysis and machine learning. BMC Oral Health. 2021 Dec 18;21(1):650. doi: 10.1186/s12903-021-02016-x. PMID: 34922509; PMCID: PMC8683819.

1402: Piccioni A, de Cunzo T, Valletta F, Covino M, Rinninella E, Raoul P, Zanza C, Mele MC, Franceschi F. Gut Microbiota and Environment in Coronary Artery Disease. Int J Environ Res Public Health. 2021 Apr 16;18(8):4242. doi: 10.3390/ijerph18084242. PMID: 33923612; PMCID: PMC8073779.

1403: Behrendt I, Fasshauer M, Eichner G. Gluten intake and metabolic health: conflicting findings from the UK Biobank. Eur J Nutr. 2021 Apr;60(3):1547-1559. doi: 10.1007/s00394-020-02351-9. Epub 2020 Aug 6. PMID: 32761538; PMCID: PMC7987594.

1404: Management of osteoporosis in postmenopausal women: the 2021 position statement of The North American Menopause Society. Menopause. 2021 Sep 1;28(9):973-997. doi: 10.1097/GME.0000000000001831. PMID: 34448749.

1405: Shi L, Wan Y, Liu J, He Z, Xu S, Xia W. Insecticide fipronil and its transformation products in human blood and urine: Assessment of human exposure in general population of China. Sci Total Environ. 2021 Sep 10;786:147342. doi: 10.1016/j.scitotenv.2021.147342. Epub 2021 Apr 24. PMID: 33964773.

1406: Gunadi, Ivana G, Mursalin DA, Pitaka RT, Zain MW, Puspitarani DA, Afandy D, Simanjaya S, Dwihantoro A, Makhmudi A. Functional outcomes of patients with short-segment Hirschsprung disease after transanal endorectal pull-through. BMC Gastroenterol. 2021 Feb 23;21(1):85. doi: 10.1186/s12876-021-01668-x. PMID: 33622253; PMCID: PMC7903717.

1407: Zhao L, Ogden CL, Yang Q, Jackson SL, Loria CM, Galuska DA, Wiltz JL, Merritt R, Cogswell ME. Association of Usual Sodium Intake with Obesity Among US Children and Adolescents, NHANES 2009-2016. Obesity (Silver Spring). 2021 Mar;29(3):587-594. doi: 10.1002/oby.23102. Epub 2021 Feb 2. PMID: 33528899.

1408: Chapman LE, Berkowitz SA, Ammerman A, De Marco M, Ng SW, Zimmer C, Caspi CE. Association between hourly wages and dietary intake after the first phase of implementation of the Minneapolis minimum wage ordinance. Public Health Nutr. 2021 Aug;24(11):3552-3565. doi: 10.1017/S1368980021000707. Epub 2021 Feb 26. PMID: 33634771; PMCID: PMC8316265.

1409: Al-Musharaf S, Alabdulaaly A, Bin Mujalli H, Alshehri H, Alajaji H, Bogis R, Alnafisah R, Alfehaid S, Alhodaib H, Murphy AM, Hussain SD, Sabico S, McTernan PG, Al-Daghri N. Sleep Quality Is Associated with Vitamin B12 Status in Female Arab Students. Int J Environ Res Public Health. 2021 Apr 25;18(9):4548. doi: 10.3390/ijerph18094548. PMID: 33922970; PMCID: PMC8123316.

1410: Zielinski CE. Regulation of T Cell Responses by Ionic Salt Signals. Cells. 2021 Sep 9;10(9):2365. doi: 10.3390/cells10092365. PMID: 34572015; PMCID: PMC8471541.

1411: Skórska KB, Grajeta H, Zabłocka-Słowińska KA. Frequency of legume consumption related to sociodemographic factors, health status and health-related variables among surveyed adults from Poland. Public Health Nutr. 2021 May;24(7):1895-1905. doi: 10.1017/S1368980020002116. Epub 2020 Aug 13. PMID: 32787977.

1412: Silva TR, Oppermann K, Reis FM, Spritzer PM. Nutrition in Menopausal Women: A Narrative Review. Nutrients. 2021 Jun 23;13(7):2149. doi: 10.3390/nu13072149. PMID: 34201460; PMCID: PMC8308420.

1413: Vanoni FO, Milani GP, Agostoni C, Treglia G, Faré PB, Camozzi P, Lava SAG, Bianchetti MG, Janett S. Magnesium Metabolism in Chronic Alcohol-Use Disorder: Meta-Analysis and Systematic Review. Nutrients. 2021 Jun 7;13(6):1959. doi: 10.3390/nu13061959. PMID: 34200366; PMCID: PMC8229336.

1414: Zanganeh M, Adab P, Li B, Pallan M, Liu WJ, Hemming K, Lin R, Liu W, Martin J, Cheng KK, Frew E. Cost-Effectiveness of a School-and Family-Based Childhood Obesity Prevention Programme in China: The "CHIRPY DRAGON" Cluster-Randomised Controlled Trial. Int J Public Health. 2021 Aug 25;66:1604025. doi: 10.3389/ijph.2021.1604025. PMID: 34531712; PMCID: PMC8439195.

1415: Fatemeh G, Sajjad M, Niloufar R, Neda S, Leila S, Khadijeh M. Effect of melatonin supplementation on sleep quality: a systematic review and meta-analysis of randomized controlled trials. J Neurol. 2022 Jan;269(1):205-216. doi: 10.1007/s00415-020-10381-w. Epub 2021 Jan 8. PMID: 33417003.

1416: Zhang JQJ, Saravanabavan S, Cheng KM, Raghubanshi A, Chandra AN, Munt A, Rayner B, Zhang Y, Chau K, Wong ATY, Rangan GK. Long-term dietary nitrate supplementation does not reduce renal cyst growth in experimental autosomal dominant polycystic kidney disease. PLoS One. 2021 Apr 22;16(4):e0248400. doi: 10.1371/journal.pone.0248400. PMID: 33886581; PMCID: PMC8061912.

1417: Li Y, Toseef MU, Jensen GA, Ortiz K, González HM, Tarraf W. Gains in insurance coverage following the affordable care act and change in preventive services use among non-elderly US immigrants. Prev Med. 2021 Jul;148:106546. doi: 10.1016/j.ypmed.2021.106546. Epub 2021 Apr 7. PMID: 33838157.

1418: Snook J, Bhala N, Beales ILP, Cannings D, Kightley C, Logan RP, Pritchard DM, Sidhu R, Surgenor S, Thomas W, Verma AM, Goddard AF. British Society of Gastroenterology guidelines for the management of iron deficiency anaemia in adults. Gut. 2021 Nov;70(11):2030-2051. doi: 10.1136/gutjnl-2021-325210. Epub 2021 Sep 8. PMID: 34497146; PMCID: PMC8515119.

1419: Lin Y, Chen D, Wu J, Chen Z. Iodine status five years after the adjustment of universal salt iodization: a cross-sectional study in Fujian Province, China. Nutr J. 2021 Feb 23;20(1):17. doi: 10.1186/s12937-021-00676-7. PMID: 33622335; PMCID: PMC7903767.

1420: Attwells S, Setiawan E, Rusjan PM, Xu C, Kish SJ, Vasdev N, Houle S, Santhirakumar A, Meyer JH. A double-blind placebo-controlled trial of minocycline on translocator protein distribution volume in treatment-resistant major depressive disorder. Transl Psychiatry. 2021 May 29;11(1):334. doi: 10.1038/s41398-021-01450-3. PMID: 34052828; PMCID: PMC8164633.

1421: Akins JD, Curtis BM, Patik JC, Olvera G, Nasirian A, Campbell JC, Shiva S, Brothers RM. Blunted hyperemic response to mental stress in young, non-Hispanic black men is not impacted by acute dietary nitrate supplementation. J Appl Physiol (1985). 2021 May 1;130(5):1510-1521. doi: 10.1152/japplphysiol.00453.2020. Epub 2021 Mar 25. PMID: 33764167.

1422: Ageel HK, Harrad S, Abdallah MA. Occurrence, human exposure, and risk of microplastics in the indoor environment. Environ Sci Process Impacts. 2022 Jan 26;24(1):17-31. doi: 10.1039/d1em00301a. PMID: 34842877.

1423: Naghshi S, Aune D, Beyene J, Mobarak S, Asadi M, Sadeghi O. Dietary intake and biomarkers of alpha linolenic acid and risk of all cause, cardiovascular, and cancer mortality: systematic review and dose-response meta-analysis of cohort studies. BMJ. 2021 Oct 13;375:n2213. doi: 10.1136/bmj.n2213. PMID: 34645650; PMCID: PMC8513503.

1424: Ajabnoor SM, Thorpe G, Abdelhamid A, Hooper L. Long-term effects of increasing omega-3, omega-6 and total polyunsaturated fats on inflammatory bowel disease and markers of inflammation: a systematic review and meta-analysis of randomized controlled trials. Eur J Nutr. 2021 Aug;60(5):2293-2316. doi: 10.1007/s00394-020-02413-y. Epub 2020 Oct 21. PMID: 33084958.

1425: Christophersen DV, Møller P, Thomsen MB, Lykkesfeldt J, Loft S, Wallin H, Vogel U, Jacobsen NR. Accelerated atherosclerosis caused by serum amyloid A response in lungs of ApoE^{-/-} mice. FASEB J. 2021 Mar;35(3):e21307. doi: 10.1096/fj.202002017R. PMID: 33638910.

1426: Gebreyesus HA, Abreha GF, Besherae SD, Abera MA, Weldegerima AH, Kidane EG, Bezabih AM, Lemma TB, Nigatu TG. Eating behavior among persons with type 2 diabetes mellitus in North Ethiopia: a cross-sectional study. BMC Endocr Disord. 2021 May 17;21(1):99. doi: 10.1186/s12902-021-00750-5. PMID: 34001064; PMCID: PMC8127210.

1427: Hantikainen E, Trolle Lagerros Y, Ye W, Serafini M, Adami HO, Bellocco R, Bonn S. Dietary Antioxidants and the Risk of Parkinson Disease: The Swedish National March Cohort. Neurology. 2021 Feb 9;96(6):e895-e903. doi: 10.1212/WNL.0000000000011373. Epub 2021 Jan 6. PMID: 33408141.

1428: Abbasnezhad A, Falahi E, Ghavamzadeh S, Beiranvand R, Talaiezadeh A, Hasanvand A, Angali KA, Choghakhori R. Association between deficient levels of circulating vitamin D, dietary intake of vitamin D, calcium and retinol, and risk of colorectal cancer in an Iranian population: A case control study. Asia Pac J Clin Oncol. 2021 Apr 14. doi: 10.1111/ajco.13524. Epub ahead of print. PMID: 33852772.

1429: Zalvan C, Yuen E, Geliebter J, Tiwari R. A Trigger Reduction Approach to Treatment of Paradoxical Vocal Fold Motion Disorder in the Pediatric Population. J Voice. 2021 Mar;35(2):323.e9-323.e15. doi: 10.1016/j.jvoice.2019.08.013. Epub 2019 Sep 16. PMID: 31537408.

1430: Palmer M, Hill J, Hosking B, Naumann F, Stoney R, Ross L, Woodward T, Josephson C. Quality of nutritional care provided to patients who develop hospital acquired malnutrition: A study across five Australian public hospitals. J Hum Nutr Diet. 2021 Aug;34(4):695-704. doi: 10.1111/jhn.12876. Epub 2021 Apr 15. PMID: 33855787.

1431: Ng SW, Colchero MA, White M. How should we evaluate sweetened beverage tax policies? A review of worldwide experience. BMC Public Health. 2021 Oct 26;21(1):1941. doi: 10.1186/s12889-021-11984-2. PMID: 34702248; PMCID: PMC8546197.

1432: Martínez-Urbistondo D, Suarez Del Villar R, Ramos-Lopez O, Fernández MA, Segovia RC, Domínguez A, de la Garza RG, Gómez ML, Ramos LP, San-Cristobal R, Daimiel L, Fernández PV, Martinez JA. Interactions of Comorbidity and Five Simple Environmental Unhealthy Habits Concerning Physical and Mental Quality of

Life in the Clinical Setting. Int J Environ Res Public Health. 2021 Sep 12;18(18):9590. doi: 10.3390/ijerph18189590. PMID: 34574515; PMCID: PMC8467323.

1433: Jiang H, Qian Y, Shen Z, Liu Y, He Y, Gao R, Shen M, Chen S, Fu Q, Yang T. Circulating microRNA-135a-3p in serum extracellular vesicles as a potential biological marker of non-alcoholic fatty liver disease. Mol Med Rep. 2021 Jul;24(1):498. doi: 10.3892/mmr.2021.12137. Epub 2021 May 6. PMID: 33955511; PMCID: PMC8127071.

1434: Litton MM, Beavers AW. The Relationship between Food Security Status and Fruit and Vegetable Intake during the COVID-19 Pandemic. Nutrients. 2021 Feb 24;13(3):712. doi: 10.3390/nu13030712. PMID: 33668207; PMCID: PMC7995961.

1435: Pinart M, Nimptsch K, Forslund SK, Schlicht K, Gueimonde M, Brigidi P, Turroni S, Ahrens W, Hebestreit A, Wolters M, Dötsch A, Nöthlings U, Oluwagbemigun K, Cuadrat RRC, Schulze MB, Standl M, Schloter M, De Angelis M, Iozzo P, Guzzardi MA, Vlaemynck G, Penders J, Jonkers DMAE, Stemmer M, Chiesa G, Cavalieri D, De Filippo C, Ercolini D, De Filippis F, Ribet D, Achamrah N, Tavolacci MP, Déchelotte P, Bouwman J, Laudes M, Pischon T. Identification and Characterization of Human Observational Studies in Nutritional Epidemiology on Gut Microbiomics for Joint Data Analysis. Nutrients. 2021 Sep 21;13(9):3292. doi: 10.3390/nu13093292. PMID: 34579168; PMCID: PMC8466729.

1436: Händel MN, Rohde JF, Jacobsen R, Heitmann BL. Processed Meat Consumption and the Risk of Cancer: A Critical Evaluation of the Constraints of Current Evidence from Epidemiological Studies. Nutrients. 2021 Oct 14;13(10):3601. doi: 10.3390/nu13103601. PMID: 34684602; PMCID: PMC8537381.

1437: Liu L, Qiao S, Zhuang L, Xu S, Chen L, Lai Q, Wang W. Choline Intake Correlates with Cognitive Performance among Elder Adults in the United States. Behav Neurol. 2021 Oct 29;2021:2962245. doi: 10.1155/2021/2962245. PMID: 34745383; PMCID: PMC8570899.

1438: Li S, Shao W, Wang C, Wang L, Xia R, Yao S, Du M, Ji X, Chu H, Zhang Z, Wang M, Wang SL. Identification of common genetic variants associated with serum concentrations of p, p'-DDE in non-occupational populations in eastern China. Environ Int. 2021 Jul;152:106507. doi: 10.1016/j.envint.2021.106507. Epub 2021 Mar 20. PMID: 33756427.

1439: Markey O, Vasilopoulou D, Kliem KE, Fagan CC, Grandison AS, Sutton R, Humphries DJ, Todd S, Jackson KG, Givens DI, Lovegrove JA. Postprandial Fatty Acid Profile, but Not Cardiometabolic Risk Markers, Is Modulated by Dairy Fat Manipulation in Adults with Moderate Cardiovascular Disease Risk: The Randomized Controlled REplacement of SaturatEd fat in dairy on Total cholesterol (RESET) Study. J Nutr. 2021 Jul 1;151(7):1755-1768. doi: 10.1093/jn/nxab050. PMID: 33758921; PMCID: PMC8327197.

1440: da Silva JT, Garzillo JMF, Rauber F, Kluczkovski A, Rivera XS, da Cruz GL, Frankowska A, Martins CA, da Costa Louzada ML, Monteiro CA, Reynolds C, Bridle

S, Levy RB. Greenhouse gas emissions, water footprint, and ecological footprint of food purchases according to their degree of processing in Brazilian metropolitan areas: a time-series study from 1987 to 2018. Lancet Planet Health. 2021 Nov;5(11):e775-e785. doi: 10.1016/S2542-5196(21)00254-0. Erratum in: Lancet Planet Health. 2021 Dec;5(12):e861. PMID: 34774121.

1441: Kroke A, Schmidt A, Amini AM, Kalotai N, Lehmann A, Haardt J, Bauer JM, Bischoff-Ferrari HA, Boeing H, Egert S, Ellinger S, Kühn T, Louis S, Lorkowski S, Nimptsch K, Remer T, Schulze MB, Siener R, Stangl GI, Volkert D, Zittermann A, Buyken AE, Watzl B, Schwingshackl L; German Nutrition Society. Dietary protein intake and health-related outcomes: a methodological protocol for the evidence evaluation and the outline of an evidence to decision framework underlying the evidence-based guideline of the German Nutrition Society. Eur J Nutr. 2022 Jan 14. doi: 10.1007/s00394-021-02789-5. Epub ahead of print. PMID: 35031889.

1442: Kim J, Aschard H, Kang JH, Lentjes MAH, Do R, Wiggs JL, Khawaja AP, Pasquale LR; Modifiable Risk Factors for Glaucoma Collaboration. Intraocular Pressure, Glaucoma, and Dietary Caffeine Consumption: A Gene-Diet Interaction Study from the UK Biobank. Ophthalmology. 2021 Jun;128(6):866-876. doi: 10.1016/j.ophtha.2020.12.009. Epub 2020 Dec 14. PMID: 33333105; PMCID: PMC8154631.

1443: Wang Y, Feng Y, Zhang H, Niu Q, Liang K, Bian C, Li H. Clinical Value and Role of miR-129-5p in Non-Alcoholic Fatty Liver Disease. Horm Metab Res. 2021 Oct;53(10):692-698. doi: 10.1055/a-1587-9211. Epub 2021 Sep 15. PMID: 34528223.

1444: Iguacel I, Perez-Cornago A, Schmidt JA, Van Puyvelde H, Travis R, Casagrande C, Nicolas G, Riboli E, Weiderpass E, Ardanaz E, Barricarte A, Bodén S, Bruno E, Ching-López A, Aune D, Jensen TE, Ericson U, Johansson I, Ma Huerta J, Katzke V, Kühn T, Sacerdote C, Schulze MB, Skeie G, Ramne S, Ward H, Gunter MJ, Huybrechts I. Evaluation of protein and amino acid intake estimates from the EPIC dietary questionnaires and 24-h dietary recalls using different food composition databases. Nutr Metab Cardiovasc Dis. 2022 Jan;32(1):80-89. doi: 10.1016/j.numecd.2021.09.012. Epub 2021 Sep 20. PMID: 34696945.

1445: Goodday SM, Travis S, Walsh A, Friend SH. Stress-related consequences of the coronavirus disease 2019 pandemic on symptoms of Crohn's disease. Eur J Gastroenterol Hepatol. 2021 Dec 1;33(12):1511-1516. doi: 10.1097/MEG.0000000000002081. PMID: 33512845; PMCID: PMC8555884.

1446: Wuopio J, Orho-Melander M, Ärnlöv J, Nowak C. Estimated salt intake and risk of atrial fibrillation in a prospective community-based cohort. J Intern Med. 2021 May;289(5):700-708. doi: 10.1111/joim.13194. Epub 2020 Nov 19. PMID: 33210391; PMCID: PMC8246952.

1447: Mirsky JB, Zack RM, Berkowitz SA, Fiechtner L. Massachusetts General Hospital Revere Food Pantry: Addressing hunger and health at an academic medical center community clinic. Healthc (Amst). 2021 Dec;9(4):100589. doi:

10.1016/j.hjdsi.2021.100589. Epub 2021 Oct 7. PMID: 34628211.

1448: Mirizzi A, Aballay LR, Misciagna G, Caruso MG, Bonfiglio C, Sorino P, Bianco A, Campanella A, Franco I, Curci R, Procino F, Cisternino AM, Notarnicola M, D'Aprile PF, Osella AR. Modified WCRF/AICR Score and All-Cause, Digestive System, Cardiovascular, Cancer and Other-Cause-Related Mortality: A Competing Risk Analysis of Two Cohort Studies Conducted in Southern Italy. Nutrients. 2021 Nov 10;13(11):4002. doi: 10.3390/nu13114002. PMID: 34836259; PMCID: PMC8620807.

1449: Lo JA, Kim JS, Jo MJ, Cho EJ, Ahn SY, Ko GJ, Kwon YJ, Kim JE. Impact of water consumption on renal function in the general population: a cross-sectional analysis of KNHANES data (2008-2017). Clin Exp Nephrol. 2021 Apr;25(4):376-384. doi: 10.1007/s10157-020-01997-3. Epub 2021 Jan 4. PMID: 33398596; PMCID: PMC7966133.

1450: Sharma AK, Baig VN, Ahuja J, Sharma S, Panwar RB, Katoch VM, Gupta R. Efficacy of IVRS-based mHealth intervention in reducing cardiovascular risk in metabolic syndrome: A cluster randomized trial. Diabetes Metab Syndr. 2021 Sep-Oct;15(5):102182. doi: 10.1016/j.dsx.2021.06.019. Epub 2021 Jun 21. PMID: 34330073.

1451: Hong S, Nagayach A, Lu Y, Peng H, Duong QA, Pham NB, Vuong CA, Bazan NG. A high fat, sugar, and salt Western diet induces motor-muscular and sensory dysfunctions and neurodegeneration in mice during aging: Ameliorative action of metformin. CNS Neurosci Ther. 2021 Dec;27(12):1458-1471. doi: 10.1111/cns.13726. Epub 2021 Sep 12. PMID: 34510763; PMCID: PMC8611779.

1452: Tsirimiagkou C, Argyris A, Karatzi K, Konstantina N, Sfikakis PP, Protogerou AD. Dietary sugars and subclinical vascular damage in moderate-to-high cardiovascular risk adults. Nutr Metab Cardiovasc Dis. 2022 Jan;32(1):98-108. doi: 10.1016/j.numecd.2021.09.027. Epub 2021 Oct 8. PMID: 34823975.

1453: Cardoso SM, Honicky M, Moreno YMF, de Lima LRA, Pacheco MA, Back IC. Subclinical atherosclerosis in children and adolescents with congenital heart disease. Cardiol Young. 2021 Apr;31(4):631-638. doi: 10.1017/S1047951120004448. Epub 2020 Dec 11. PMID: 33303049.

1454: White S, Zarotti N, Beever D, Bradburn M, Norman P, Coates E, Stavroulakis T, White D, McGeachan A, Williams I, Hackney G, Halliday V, McDermott C; HighCALS group. The nutritional management of people living with amyotrophic lateral sclerosis: A national survey of dietitians. J Hum Nutr Diet. 2021 Dec;34(6):1064-1071. doi: 10.1111/jhn.12900. Epub 2021 Apr 24. PMID: 33786908.

1455: Pecoraro P, Gallè F, Muscariello E, Di Mauro V, Daniele O, Forte S, Ricchiuti R, Liguori G, Valerio G. A telehealth intervention for ensuring continuity of care of pediatric obesity during the CoVid-19 lockdown in Italy. Nutr Metab Cardiovasc Dis. 2021 Nov 29;31(12):3502-3507. doi: 10.1016/j.numecd.2021.09.026. Epub 2021 Oct 7. PMID: 34728130; PMCID:

PMC8496959.

1456: Virgens IPA, Santana NM, Lima SCVC, Fayh APT. Can COVID-19 be a risk for cachexia for patients during intensive care? Narrative review and nutritional recommendations. Br J Nutr. 2021 Aug 28;126(4):552-560. doi: 10.1017/S0007114520004420. Epub 2020 Nov 5. PMID: 33261670; PMCID: PMC7711335.

1457: Pu Y, Zhu G, Xu Y, Zheng S, Tang B, Huang H, Wu IXY, Huang D, Liu Y, Zhang X. Association Between Vitamin D Exposure and Head and Neck Cancer: A Systematic Review With Meta-Analysis. Front Immunol. 2021 Feb 23;12:627226. doi: 10.3389/fimmu.2021.627226. PMID: 33732250; PMCID: PMC7959800.

1458: Pierce SR, Fang Z, Yin Y, West L, Asher M, Hao T, Zhang X, Tucker K, Staley A, Fan Y, Sun W, Moore DT, Xu C, Tsai YH, Parker J, Prabhu VV, Allen JE, Lee D, Zhou C, Bae-Jump V. Targeting dopamine receptor D2 as a novel therapeutic strategy in endometrial cancer. J Exp Clin Cancer Res. 2021 Feb 8;40(1):61. doi: 10.1186/s13046-021-01842-9. PMID: 33557912; PMCID: PMC7869513.

1459: Tsirimiagkou C, Basdeki ED, Kyriazopoulou Korovesi AA, Chairistanidou C, Ouamer DS, Argyris A, Sfikakis PP, Karatzi K, Protogerou AD. Habitual consumption of instant coffee is favorably associated with arterial stiffness but not with atheromatosis. Clin Nutr ESPEN. 2021 Oct;45:363-368. doi: 10.1016/j.clnesp.2021.07.018. Epub 2021 Aug 10. PMID: 34620341.

1460: Ali HI, Attlee A, Alhebshi S, Elmi F, Al Dhaheri AS, Stojanovska L, El Mesmoudi N, Platat C. Feasibility Study of a Newly Developed Technology-Mediated Lifestyle Intervention for Overweight and Obese Young Adults. Nutrients. 2021 Jul 26;13(8):2547. doi: 10.3390/nu13082547. PMID: 34444707; PMCID: PMC8399959.

1461: Sisti JS, Mezzacca TA, Anekwe A, Farley SM. Examining Trends in Beverage Sales in New York City During Comprehensive Efforts to Reduce Sugary Drink Consumption, 2010-2015. J Community Health. 2021 Jun;46(3):609-617. doi: 10.1007/s10900-020-00911-y. PMID: 32920704.

1462: Yoo JI, Han JK, Youn HS, Jung JH. Comparison of Health Awareness in South Korean Middle School Students According to Type of Online Physical Education Classes during the COVID-19 Pandemic. Int J Environ Res Public Health. 2021 Jul 27;18(15):7937. doi: 10.3390/ijerph18157937. PMID: 34360230; PMCID: PMC8345634.

1463: Cremers SL, Khan ARG, Ahn J, Cremers L, Weber J, Kossler AL, Pigotti C, Martinez A. New Indicator of Children's Excessive Electronic Screen Use and Factors in Meibomian Gland Atrophy. Am J Ophthalmol. 2021 Sep;229:63-70. doi: 10.1016/j.ajo.2021.03.035. Epub 2021 Apr 17. PMID: 33857506.

1464: Akhavan Zanjani M, Rahmani S, Mehranfar S, Zarrin M, Bazyar H, Moradi Poodeh B, Zare Javid A, Hosseini SA, Sadeghian M. Soy Foods and the Risk of Fracture: A Systematic Review of Prospective Cohort Studies. Complement Med Res. 2021 Sep 21:1-10. English. doi: 10.1159/000519036. Epub ahead of print. PMID: 34547749.

1465: Nowak J, Hudzik B, Jagielski P, Kulik-Kupka K, Danikiewicz A, Zubelewicz-Szkodzińska B. Lack of Seasonal Variations in Vitamin D Concentrations among Hospitalized Elderly Patients. Int J Environ Res Public Health. 2021 Feb 9;18(4):1676. doi: 10.3390/ijerph18041676. PMID: 33572447; PMCID: PMC7916205.

1466: Murphy MJ, Brandie F, Ebare M, Harrison M, Dow E, Bartlett WA, Craig D. Personalising laboratory medicine in the 'real world': Assessing clinical utility, by clinical indication, of serum total B₁₂ and Active-B₁₂® (holotranscobalamin) in the diagnosis of vitamin B₁₂ deficiency. Ann Clin Biochem. 2021 Sep;58(5):445-451. doi: 10.1177/00045632211003605. Epub 2021 Apr 23. PMID: 33715445; PMCID: PMC8458683.

1467: Arriagada-Petersen C, Fernandez P, Gomez M, Ravello N, Palomo I, Fuentes E, Ávila F. Effect of advanced glycation end products on platelet activation and aggregation: a comparative study of the role of glyoxal and methylglyoxal. Platelets. 2021 May 19;32(4):507-515. doi: 10.1080/09537104.2020.1767770. Epub 2020 May 23. PMID: 32449466.

1468: Szőcs H, Horváth Z, Vizin G. A szégyen mediációs szerepe a stigma és az életminőség kapcsolatában coeliakiában szenvedő betegek körében. The Hungarian adaptation of the Stigma Scale for Chronic Illness-8 [Shame mediates the relationship between stigma and quality of life among patients with coeliac disease. A 8 tételes Stigmatizáció Krónikus Betegségekben Kérdőív magyar adaptálása]. Orv Hetil. 2021 Dec 5;162(49):1968-1976. Hungarian. doi: 10.1556/650.2021.32258. PMID: 34864639.

1469: Marzolla V, Feraco A, Limana F, Kolkhof P, Armani A, Caprio M. Class-specific responses of brown adipose tissue to steroidal and nonsteroidal mineralocorticoid receptor antagonists. J Endocrinol Invest. 2022 Jan;45(1):215-220. doi: 10.1007/s40618-021-01635-z. Epub 2021 Jul 16. PMID: 34272678; PMCID: PMC8285041.

1470: Nury E, Morze J, Grummich K, Rücker G, Hoffmann G, Angele CM, Steinacker JM, Conrad J, Schmid D, Meerpohl JJ, Schwingshackl L. Effects of nutrition intervention strategies in the primary prevention of overweight and obesity in school settings: a protocol for a systematic review and network meta-analysis. Syst Rev. 2021 Apr 22;10(1):122. doi: 10.1186/s13643-021-01661-1. PMID: 33888162; PMCID: PMC8063346.

1471: Wang B, Lin C, Cheng H, Duan X, Wang Q, Xu D. Health Risk Assessment of Metals via Multi-Source Oral Exposure for Children Living in Areas with Intense Electronic Manufacturing Activities. Int J Environ Res Public Health. 2021 Oct 29;18(21):11409. doi: 10.3390/ijerph182111409. PMID: 34769926; PMCID: PMC8583640.

1472: Waid JL, Sinharoy SS, Ali M, Alam MM, Wendt AS, Gabrysch S. What Were the Drivers of Improving Child Nutritional Status in Bangladesh? An Analysis of National Household Data from 1992 to 2005 Guided by the UNICEF Framework. J

Nutr. 2021 Apr 8;151(4):987-998. doi: 10.1093/jn/nxaa425. PMID: 33693774.

1473: Sala-Vila A, Arenaza-Urquijo EM, Sánchez-Benavides G, Suárez-Calvet M, Milà-Alomà M, Grau-Rivera O, González-de-Echávarri JM, Crous-Bou M, Minguillón C, Fauria K, Operto G, Falcón C, Salvadó G, Cacciaglia R, Ingala S, Barkhof F, Schröder H, Scarmeas N, Gispert JD, Molinuevo JL; ALFA study. DHA intake relates to better cerebrovascular and neurodegeneration neuroimaging phenotypes in middle-aged adults at increased genetic risk of Alzheimer disease. Am J Clin Nutr. 2021 Jun 1;113(6):1627-1635. doi: 10.1093/ajcn/nqab016. PMID: 33733657; PMCID: PMC8168359.

1474: Smith C, Goss HR, Issartel J, Belton S. Health Literacy in Schools? A Systematic Review of Health-Related Interventions Aimed at Disadvantaged Adolescents. Children (Basel). 2021 Feb 25;8(3):176. doi: 10.3390/children8030176. PMID: 33668861; PMCID: PMC7996245.

1475: Bjelakovic M, Nikolova D, Bjelakovic G, Gluud C. Vitamin D supplementation for chronic liver diseases in adults. Cochrane Database Syst Rev. 2021 Aug 25;8(8):CD011564. doi: 10.1002/14651858.CD011564.pub3. PMID: 34431511; PMCID: PMC8407054.

1476: Rodrigues SG, Abraldes JG, Tsochatzis E, Bosch J, Berzigotti A. Royal Free Hospital-estimated glomerular filtration rate for prognostic stratification of first acute kidney injury in cirrhosis. Liver Int. 2021 Apr;41(4):819-827. doi: 10.1111/liv.14765. Epub 2021 Jan 15. PMID: 33314543.

1477: Traugott M, Hoepler W, Kitzberger R, Pavlata S, Seitz T, Baumgartner S, Placher-Sorko G, Pirker-Krassnig D, Ehehalt U, Grasnek A, Beham-Kacerovsky M, Friese E, Wenisch C, Neuhold S. Successful treatment of intubation-induced severe neurogenic post-extubation dysphagia using pharyngeal electrical stimulation in a COVID-19 survivor: a case report. J Med Case Rep. 2021 Mar 22;15(1):148. doi: 10.1186/s13256-021-02763-z. PMID: 33752743; PMCID: PMC7983095.

1478: Rabbani E, Golgiri F, Janani L, Moradi N, Fallah S, Abiri B, Vafa M. Randomized Study of the Effects of Zinc, Vitamin A, and Magnesium Co-supplementation on Thyroid Function, Oxidative Stress, and hs-CRP in Patients with Hypothyroidism. Biol Trace Elem Res. 2021 Nov;199(11):4074-4083. doi: 10.1007/s12011-020-02548-3. Epub 2021 Jan 7. PMID: 33409923.

1479: Guo Y, Tong Y, Zhu H, Xiao Y, Guo H, Shang L, Zheng W, Ma S, Liu X, Bai Y. Quercetin suppresses pancreatic ductal adenocarcinoma progression via inhibition of SHH and TGF-β/Smad signaling pathways. Cell Biol Toxicol. 2021 Jun;37(3):479-496. doi: 10.1007/s10565-020-09562-0. Epub 2020 Oct 17. PMID: 33070227.

1480: Starbuck JM, Llambrich S, Gonzàlez R, Albaigès J, Sarlé A, Wouters J, González A, Sevillano X, Sharpe J, De La Torre R, Dierssen M, Vande Velde G, Martínez-Abadías N. Green tea extracts containing epigallocatechin-3-gallate

modulate facial development in Down syndrome. Sci Rep. 2021 Feb 25;11(1):4715. doi: 10.1038/s41598-021-83757-1. PMID: 33633179; PMCID: PMC7907288.

1481: Duarte GBS, Callou KRA, Almondes KGS, Rogero MM, Pollak DF, Cozzolino SMF. Evaluation of biomarkers related to zinc nutritional status, antioxidant activity and oxidative stress in rheumatoid arthritis patients. Nutr Health. 2021 May 18:2601060211015594. doi: 10.1177/02601060211015594. Epub ahead of print. PMID: 34006137.

1482: Islam FMA, Lambert EA, Islam SMS, Islam MA, Biswas D, McDonald R, Maddison R, Thompson B, Lambert GW. Lowering blood pressure by changing lifestyle through a motivational education program: a cluster randomized controlled trial study protocol. Trials. 2021 Jul 8;22(1):438. doi: 10.1186/s13063-021-05379-2. PMID: 34238363; PMCID: PMC8264477.

1483: Rücklová K, Hrubá E, Pavlíková M, Hanák P, Farolfi M, Chrastina P, Vlášková H, Kousal B, Smolka V, Foltenová H, Adam T, Friedecký D, Ješina P, Zeman J, Kožich V, Honzík T. Impact of Newborn Screening and Early Dietary Management on Clinical Outcome of Patients with Long Chain 3-Hydroxyacyl-CoA Dehydrogenase Deficiency and Medium Chain Acyl-CoA Dehydrogenase Deficiency-A Retrospective Nationwide Study. Nutrients. 2021 Aug 24;13(9):2925. doi: 10.3390/nu13092925. PMID: 34578803; PMCID: PMC8469775.

1484: Mateo-Fernández M, Valenzuela-Gómez F, Font R, Del Río-Celestino M, Merinas-Amo T, Alonso-Moraga Á. In Vivo and In Vitro Assays Evaluating the Biological Activity of Taurine, Glucose and Energetic Beverages. Molecules. 2021 Apr 11;26(8):2198. doi: 10.3390/molecules26082198. PMID: 33920365; PMCID: PMC8069289.

1485: Matovu JKB, Kabwama SN, Ssekamatte T, Ssenkusu J, Wanyenze RK. COVID-19 Awareness, Adoption of COVID-19 Preventive Measures, and Effects of COVID-19 Lockdown Among Adolescent Boys and Young Men in Kampala, Uganda. J Community Health. 2021 Aug;46(4):842-853. doi: 10.1007/s10900-021-00961-w. Epub 2021 Jan 22. PMID: 33481156; PMCID: PMC7820821.

1486: García Pérez de Sevilla G, Barceló Guido O, De la Cruz MP, Blanco Fernández A, Alejo LB, Montero Martínez M, Pérez-Ruiz M. Adherence to a Lifestyle Exercise and Nutrition Intervention in University Employees during the COVID-19 Pandemic: A Randomized Controlled Trial. Int J Environ Res Public Health. 2021 Jul 14;18(14):7510. doi: 10.3390/ijerph18147510. PMID: 34299960; PMCID: PMC8305330.

1487: Walkowiak D, Mikołuć B, Mozrzymas R, Kałużny Ł, Didycz B, Jaglowska J, Kurylak D, Walkowiak J. The Impact of the First 2020 COVID-19 Lockdown on the Metabolic Control of Patients with Phenylketonuria. Nutrients. 2021 Jun 12;13(6):2024. doi: 10.3390/nu13062024. PMID: 34204602; PMCID: PMC8231121.

1488: Lam CN, Watt AE, Isenring EA, de van der Schueren MAE, van der Meij BS. The effect of oral omega-3 polyunsaturated fatty acid supplementation on muscle

maintenance and quality of life in patients with cancer: A systematic review and meta-analysis. Clin Nutr. 2021 Jun;40(6):3815-3826. doi: 10.1016/j.clnu.2021.04.031. Epub 2021 Apr 27. PMID: 34130028.

1489: Wright KM, McFerrin J, Alcázar Magaña A, Roberts J, Caruso M, Kretzschmar D, Stevens JF, Maier CS, Quinn JF, Soumyanath A. Developing a Rational, Optimized Product of <i>Centella asiatica</i> for Examination in Clinical Trials: Real World Challenges. Front Nutr. 2022 Jan 14;8:799137. doi: 10.3389/fnut.2021.799137. PMID: 35096945; PMCID: PMC8797052.

1490: Mølmen KS, Hammarström D, Pedersen K, Lian Lie AC, Steile RB, Nygaard H, Khan Y, Hamarsland H, Koll L, Hanestadhaugen M, Eriksen AL, Grindaker E, Whist JE, Buck D, Ahmad R, Strand TA, Rønnestad BR, Ellefsen S. Vitamin D₃ supplementation does not enhance the effects of resistance training in older adults. J Cachexia Sarcopenia Muscle. 2021 Jun;12(3):599-628. doi: 10.1002/jcsm.12688. Epub 2021 Mar 31. PMID: 33788419; PMCID: PMC8200443.

1491: Sweeny L, Mayland E, Swendseid BP, Curry JM, Kejner AE, Thomas CM, Kain JJ, Cannady SB, Tasche K, Rosenthal EL, DiLeo M, Luginbuhl AJ, Theeuwen H, Sarwary JR, Petrisor D, Wax MK. Microvascular Reconstruction of Osteonecrosis: Assessment of Long-term Quality of Life. Otolaryngol Head Neck Surg. 2021 Nov;165(5):636-646. doi: 10.1177/0194599821990682. Epub 2021 Feb 23. PMID: 33618563.

1492: López-Moreno A, Acuña I, Torres-Sánchez A, Ruiz-Moreno Á, Cerk K, Rivas A, Suárez A, Monteoliva-Sánchez M, Aguilera M. Next Generation Probiotics for Neutralizing Obesogenic Effects: Taxa Culturing Searching Strategies. Nutrients. 2021 May 12;13(5):1617. doi: 10.3390/nu13051617. PMID: 34065873; PMCID: PMC8151043.

1493: Koch M, Furtado JD, DeKosky ST, Fitzpatrick AL, Lopez OL, Kuller LH, Mukamal KJ, Jensen MK. Case-cohort study of plasma phospholipid fatty acid profiles, cognitive function, and risk of dementia: a secondary analysis in the Ginkgo Evaluation of Memory Study. Am J Clin Nutr. 2021 Jul 1;114(1):154-162. doi: 10.1093/ajcn/nqab087. PMID: 33880495; PMCID: PMC8277434.

1494: Schoen MS, Singh RH. Plasma metabolomic profile changes in females with phenylketonuria (PKU) following a camp intervention. Am J Clin Nutr. 2021 Dec 2:nqab400. doi: 10.1093/ajcn/nqab400. Epub ahead of print. PMID: 34864852.

1495: Rashid M, Verhoeven AJM, Mulder MT, Timman R, Ozcan B, van Beek-Nieuwland Y, Chow LM, van de Laar RJJM, Dik WA, Sijbrands EJG, Berk KA. The effect of monomeric and oligomeric FLAVAnols in patients with type 2 diabetes and microalbuminuria (FLAVA-trial): A double-blind randomized controlled trial. Clin Nutr. 2021 Nov;40(11):5587-5594. doi: 10.1016/j.clnu.2021.09.038. Epub 2021 Sep 24. PMID: 34656955.

1496: Sobczyńska-Malefora A, Delvin E, McCaddon A, Ahmadi KR, Harrington DJ. Vitamin B₁₂ status in health and disease: a critical review.

Diagnosis of deficiency and insufficiency - clinical and laboratory pitfalls.
Crit Rev Clin Lab Sci. 2021 Sep;58(6):399-429. doi:
10.1080/10408363.2021.1885339. Epub 2021 Apr 21. PMID: 33881359.

1497: Shuai M, Zuo LS, Miao Z, Gou W, Xu F, Jiang Z, Ling CW, Fu Y, Xiong F, Chen YM, Zheng JS. Multi-omics analyses reveal relationships among dairy consumption, gut microbiota and cardiometabolic health. EBioMedicine. 2021 Apr;66:103284. doi: 10.1016/j.ebiom.2021.103284. Epub 2021 Mar 19. PMID: 33752125; PMCID: PMC7985282.

1498: Phelan S, Jelalian E, Coustan D, Caughey AB, Castorino K, Hagobian T, Muñoz-Christian K, Schaffner A, Shields L, Heaney C, McHugh A, Wing RR. Protocol for a randomized controlled trial of pre-pregnancy lifestyle intervention to reduce recurrence of gestational diabetes: Gestational Diabetes Prevention/Prevención de la Diabetes Gestacional. Trials. 2021 Apr 7;22(1):256. doi: 10.1186/s13063-021-05204-w. PMID: 33827659; PMCID: PMC8024941.

1499: Schmidt FP, Herzog J, Schnorbus B, Ostad MA, Lasetzki L, Hahad O, Schäfers G, Gori T, Sørensen M, Daiber A, Münzel T. The impact of aircraft noise on vascular and cardiac function in relation to noise event number: a randomized trial. Cardiovasc Res. 2021 Apr 23;117(5):1382-1390. doi: 10.1093/cvr/cvaa204. PMID: 32914847; PMCID: PMC8064430.

1500: Hiltunen K, Saarela RKT, Kautiainen H, Roitto HM, Pitkälä KH, Mäntylä P. Relationship between Fried's frailty phenotype and oral frailty in long-term care residents. Age Ageing. 2021 Nov 10;50(6):2133-2139. doi: 10.1093/ageing/afab177. PMID: 34473831; PMCID: PMC8581380.

1501: Maestre A, Sospedra I, Martínez-Sanz JM, Gutierrez-Hervas A, Fernández-Saez J, Hurtado-Sánchez JA, Norte A. Assessment of Spanish Food Consumption Patterns during COVID-19 Home Confinement. Nutrients. 2021 Nov 17;13(11):4122. doi: 10.3390/nu13114122. PMID: 34836377; PMCID: PMC8617653.

1502: Boedt T, Matthys C, Lie Fong S, De Neubourg D, Vereeck S, Seghers J, Van der Gucht K, Weyn B, Geerts D, Spiessens C, Dancet EAF. Systematic development of a mobile preconception lifestyle programme for couples undergoing IVF: the PreLiFe-programme. Hum Reprod. 2021 Aug 18;36(9):2493-2505. doi: 10.1093/humrep/deab166. PMID: 34379119.

1503: Flythe JE, Karlsson N, Sundgren A, Cordero P, Grandinetti A, Cremisi H, Rydén A. Development of a preliminary conceptual model of the patient experience of chronic kidney disease: a targeted literature review and analysis. BMC Nephrol. 2021 Jun 23;22(1):233. doi: 10.1186/s12882-021-02440-9. PMID: 34162354; PMCID: PMC8220773.

1504: Crisan LL, Lee HM, Fan W, Wong ND. Association of cardiovascular health with mortality among COPD patients: National Health and Nutrition Examination Survey III. Respir Med Res. 2021 Nov;80:100860. doi: 10.1016/j.resmer.2021.100860. Epub 2021 Sep 9. PMID: 34600350.

1505: Kallings LV, Blom V, Ekblom B, Holmlund T, Eriksson JS, Andersson G, Wallin P, Ekblom-Bak E. Workplace sitting is associated with self-reported general health and back/neck pain: a cross-sectional analysis in 44,978 employees. BMC Public Health. 2021 May 6;21(1):875. doi: 10.1186/s12889-021-10893-8. PMID: 33957889; PMCID: PMC8101162.

1506: Hofman A, Limpens MAM, de Crom TOE, Ikram MA, Luik AI, Voortman T. Trajectories and Determinants of Physical Activity during COVID-19 Pandemic: A Population-Based Study of Middle-Aged and Elderly Individuals in The Netherlands. Nutrients. 2021 Oct 27;13(11):3832. doi: 10.3390/nu13113832. PMID: 34836085; PMCID: PMC8618734.

1507: Kassie Tesema A, Liyew AM, Alem AZ, Yeshaw Y, Tesema GA, Teshale AB. Spatial distribution and determinants of undernutrition among reproductive age women of Ethiopia: A multilevel analysis. PLoS One. 2021 Sep 20;16(9):e0257664. doi: 10.1371/journal.pone.0257664. PMID: 34543339; PMCID: PMC8452048.

1508: Mann JFE, Chang TI, Cushman WC, Furth SL, Ix JH, Hou FF, Knoll GA, Muntner P, Pecoits-Filho R, Sarnak MJ, Tomson CRV, Craig JC, Tunnicliffe DJ, Howell M, Tonelli M, Cheung M, Earley A, Cheung AK. Commentary on the KDIGO 2021 Clinical Practice Guideline for the Management of Blood Pressure in CKD. Curr Cardiol Rep. 2021 Aug 16;23(9):132. doi: 10.1007/s11886-021-01559-3. PMID: 34398316; PMCID: PMC8366157.

1509: Zandieh N, Hemami MR, Darvishi A, Hasheminejad SM, Abdollahi Z, Zarei M, Heshmat R. Economic evaluation of a national vitamin D supplementation program among Iranian adolescents for the prevention of adulthood type 2 diabetes mellitus. BMC Complement Med Ther. 2022 Jan 3;22(1):1. doi: 10.1186/s12906-021-03474-0. PMID: 34980092; PMCID: PMC8722369.

1510: Martins VL, Belo I, Luz A, Moleiro P. Adolescer Saudável: screening and follow-up of risk at school. Einstein (Sao Paulo). 2021 Sep 22;19:eAO5849. doi: 10.31744/einstein_journal/2021AO5849. PMID: 34586156; PMCID: PMC8428807.

1511: Galarregui C, Cantero I, Marin-Alejandre BA, Monreal JI, Elorz M, Benito-Boillos A, Herrero JI, de la O V, Ruiz-Canela M, Hermsdorff HHM, Bressan J, Tur JA, Martínez JA, Zulet MA, Abete I. Dietary intake of specific amino acids and liver status in subjects with nonalcoholic fatty liver disease: fatty liver in obesity (FLiO) study. Eur J Nutr. 2021 Jun;60(4):1769-1780. doi: 10.1007/s00394-020-02370-6. Epub 2020 Aug 28. PMID: 32857176.

1512: Karimi E, Bitarafan S, Mousavi SM, Zargarzadeh N, Mokhtari P, Hawkins J, Meysamie A, Koohdani F. The effect of vitamin D supplementation on fibroblast growth factor-23 in patients with chronic kidney disease: A systematic review and meta-analysis. Phytother Res. 2021 Oct;35(10):5339-5351. doi: 10.1002/ptr.7139. Epub 2021 Apr 30. PMID: 33928687.

1513: Perez-Diaz-Del-Campo N, Marin-Alejandre BA, Cantero I, Monreal JI, Elorz

M, Herrero JI, Benito-Boillos A, Riezu-Boj JI, Milagro FI, Tur JA, Martinez JA, Abete I, Zulet MA. Differential response to a 6-month energy-restricted treatment depending on SH2B1 rs7359397 variant in NAFLD subjects: Fatty Liver in Obesity (FLiO) Study. Eur J Nutr. 2021 Sep;60(6):3043-3057. doi: 10.1007/s00394-020-02476-x. Epub 2021 Jan 20. PMID: 33474638.

1514: Bastida G, Herrera-de Guise C, Algaba A, Ber Nieto Y, Soares JM, Robles V, Bermejo F, Sáez-González E, Gomollón F, Nos P. Sucrosomial Iron Supplementation for the Treatment of Iron Deficiency Anemia in Inflammatory Bowel Disease Patients Refractory to Oral Iron Treatment. Nutrients. 2021 May 22;13(6):1770. doi: 10.3390/nu13061770. PMID: 34067320; PMCID: PMC8224651.

1515: Cho SMJ, Lee H, Shim JS, Jeon JY, Kim HC. Association between physical activity and inflammatory markers in community-dwelling, middle-aged adults. Appl Physiol Nutr Metab. 2021 Jul;46(7):828-836. doi: 10.1139/apnm-2020-1069. Epub 2021 Feb 10. PMID: 33566730.

1516: Lechien JR, Bobin F, Muls V, Saussez S, Hans S. Laryngopharyngeal Reflux Disease is More Severe in Obese Patients: A Prospective Multicenter Study. Laryngoscope. 2021 Nov;131(11):E2742-E2748. doi: 10.1002/lary.29676. Epub 2021 Jun 4. PMID: 34086292.

1517: Simpson HL, Roberts CL, Thompson LM, Leiper CR, Gittens N, Trotter E, Duckworth CA, Papoutsopoulou S, Miyajima F, Roberts P, O'Kennedy N, Rhodes JM, Campbell BJ. Soluble Non-Starch Polysaccharides From Plantain (<i>Musa x paradisiaca</i> L.) Diminish Epithelial Impact of <i>Clostridioides difficile</i>. Front Pharmacol. 2021 Dec 10;12:766293. doi: 10.3389/fphar.2021.766293. PMID: 34955836; PMCID: PMC8707065.

1518: Adriaans DJ, Dierick-van Daele AT, van Bakel MJHM, Nieuwenhuijzen GA, Teijink JA, Heesakkers FF, van Laarhoven HW. Digital Self-Management Support Tools in the Care Plan of Patients With Cancer: Review of Randomized Controlled Trials. J Med Internet Res. 2021 Jun 29;23(6):e20861. doi: 10.2196/20861. PMID: 34184997; PMCID: PMC8278296.

1519: Cheng TJ, More SL, Maddaloni MA, Fung ES. Evaluation of potential gastrointestinal carcinogenicity associated with the ingestion of asbestos. Rev Environ Health. 2020 Sep 23;36(1):15-26. doi: 10.1515/reveh-2020-0061. PMID: 32966235.

1520: Wangaskar SA, Sahu SK, Majella MG, Rajaa S. Prevalence of malnutrition and its associated sociodemographic and clinical factors among adolescents in selected schools of Urban Puducherry, India. Niger Postgrad Med J. 2021 Oct-Dec;28(4):285-290. doi: 10.4103/npmj.npmj_684_21. PMID: 34850757.

1521: Kistler BM, Moore LW, Benner D, Biruete A, Boaz M, Brunori G, Chen J, Drechsler C, Guebre-Egziabher F, Hensley MK, Iseki K, Kovesdy CP, Kuhlmann MK, Saxena A, Wee PT, Brown-Tortorici A, Garibotto G, Price SR, Yee-Moon Wang A, Kalantar-Zadeh K. The International Society of Renal Nutrition and Metabolism

Commentary on the National Kidney Foundation and Academy of Nutrition and Dietetics KDOQI Clinical Practice Guideline for Nutrition in Chronic Kidney Disease. J Ren Nutr. 2021 Mar;31(2):116-120.e1. doi: 10.1053/j.jrn.2020.05.002. Epub 2020 Jul 29. PMID: 32737016; PMCID: PMC8045140.

1522: Guillien A, Lepeule J, Seyve E, Le Moual N, Pin I, Degano B, Garcia-Aymerich J, Pépin JL, Pison C, Dumas O, Varraso R, Siroux V. Profile of exposures and lung function in adults with asthma: An exposome approach in the EGEA study. Environ Res. 2021 May;196:110422. doi: 10.1016/j.envres.2020.110422. Epub 2020 Nov 5. PMID: 33160974.

1523: Judge C, O'Donnell MJ, Hankey GJ, Rangarajan S, Chin SL, Rao-Melacini P, Ferguson J, Smyth A, Xavier D, Lisheng L, Zhang H, Lopez-Jaramillo P, Damasceno A, Langhorne P, Rosengren A, Dans AL, Elsayed A, Avezum A, Mondo C, Ryglewicz D, Czlonkowska A, Pogosova N, Weimar C, Diaz R, Yusoff K, Yusufali A, Oguz A, Wang X, Lanas F, Ogah OS, Ogunniyi A, Iversen HK, Malaga G, Rumboldt Z, Oveisgharan S, Al Hussain F, Yusuf S. Urinary Sodium and Potassium, and Risk of Ischemic and Hemorrhagic Stroke (INTERSTROKE): A Case-Control Study. Am J Hypertens. 2021 Apr 20;34(4):414-425. doi: 10.1093/ajh/hpaa176. PMID: 33197265; PMCID: PMC8057138.

1524: Sisto A, Vicinanza F, Tuccinardi D, Watanabe M, Gallo IF, D'Alessio R, Manfrini S, Quintiliani L. The psychological impact of COVID-19 pandemic on patients included in a bariatric surgery program. Eat Weight Disord. 2021 Aug;26(6):1737-1747. doi: 10.1007/s40519-020-00988-3. Epub 2020 Aug 28. PMID: 32857287; PMCID: PMC7453189.

1525: Walsh JJ, Neudorf H, Little JP. 14-Day Ketone Supplementation Lowers Glucose and Improves Vascular Function in Obesity: A Randomized Crossover Trial. J Clin Endocrinol Metab. 2021 Mar 25;106(4):e1738-e1754. doi: 10.1210/clinem/dgaa925. PMID: 33367782; PMCID: PMC7993591.

1526: Van Parys A, Brække MS, Karlsson T, Vinknes KJ, Tell GS, Haugsgjerd TR, Ueland PM, Øyen J, Dierkes J, Nygård O, Lysne V. Assessment of Dietary Choline Intake, Contributing Food Items and Associations with One-carbon and Lipid Metabolites in Middle-aged and Elderly Adults: The Hordaland Health Study. J Nutr. 2021 Oct 13:nxab367. doi: 10.1093/jn/nxab367. Epub ahead of print. PMID: 34643705.

1527: Wang S, Zhao W, Sun L, Xiao SM, Lin S, Zhao J, Xiao H, Xing X, Lao XQ, Chen YM, Liu X. Independent and opposing associations of dietary phytosterols intake and PLCE1 rs2274223 polymorphisms on esophageal squamous cell carcinoma risk. Eur J Nutr. 2021 Dec;60(8):4357-4366. doi: 10.1007/s00394-021-02561-9. Epub 2021 May 27. PMID: 34046701.

1528: Zhang T, Zhang Y, Li W, Wang L, Jiao Y, Wang Y, Jiang D, Gao X. Occurrence and dietary exposure of heavy metals in marketed vegetables and fruits of Shandong Province, China. Food Sci Nutr. 2021 Jul 23;9(9):5166-5173. doi: 10.1002/fsn3.2485. PMID: 34532025; PMCID: PMC8441287.

1529: Nohara-Shitama Y, Adachi H, Enomoto M, Fukami A, Morikawa N, Sakaue A, Toyomasu K, Yamamoto M, Fukumoto Y. Differential impacts of 24 hour urinary sodium excretion on cardiovascular diseases or cancer mortality in a general population. J Cardiol. 2021 Oct;78(4):334-340. doi: 10.1016/j.jjcc.2021.04.013. Epub 2021 May 24. PMID: 34039467.

1530: Matysik S, Krautbauer S, Liebisch G, Schött HF, Kjølbaek L, Astrup A, Blachier F, Beaumont M, Nieuwdorp M, Hartstra A, Rampelli S, Pagotto U, Iozzo P. Short-chain fatty acids and bile acids in human faeces are associated with the intestinal cholesterol conversion status. Br J Pharmacol. 2021 Aug;178(16):3342-3353. doi: 10.1111/bph.15440. Epub 2021 May 5. PMID: 33751575.

1531: Yao N, Yan S, Guo Y, Wang H, Li X, Wang L, Hu W, Li B, Cui W. The association between carotenoids and subjects with overweight or obesity: a systematic review and meta-analysis. Food Funct. 2021 Jun 8;12(11):4768-4782. doi: 10.1039/d1fo00004g. PMID: 33977977.

1532: Dalrymple KV, Tydeman FAS, Taylor PD, Flynn AC, O'Keeffe M, Briley AL, Santosh P, Hayes L, Robson SC, Nelson SM, Sattar N, Whitworth MK, Mills HL, Singh C, Seed CStat PT, White SL, Lawlor DA, Godfrey KM, Poston L; UPBEAT consortium. Adiposity and cardiovascular outcomes in three-year-old children of participants in UPBEAT, an RCT of a complex intervention in pregnant women with obesity. Pediatr Obes. 2021 Mar;16(3):e12725. doi: 10.1111/ijpo.12725. Epub 2020 Sep 11. PMID: 32914569; PMCID: PMC7116719.

1533: Fraser DA, Wang X, Lund J, Nikolić N, Iruarrizaga-Lejarreta M, Skjaeret T, Alonso C, Kastelein JJP, Rustan AC, Kim YO, Schuppan D. A structurally engineered fatty acid, icosabutate, suppresses liver inflammation and fibrosis in NASH. J Hepatol. 2021 Dec 13:S0168-8278(21)02244-3. doi: 10.1016/j.jhep.2021.12.004. Epub ahead of print. PMID: 34915054.

1534: Martínez-Vázquez SE, Ceballos-Rasgado M, Posada-Velázquez R, Hunot-Alexander C, Nava-González EJ, Ramírez-Silva I, Aguilar-López DK, Quiroz-Olguín G, López-Jara B, Delgado-de-la-Cruz C, Huescas-Juárez S, Silva M, Kaufer-Horwitz M. Perceived Diet Quality, Eating Behaviour, and Lifestyle Changes in a Mexican Population with Internet Access during Confinement for the COVID-19 Pandemic: ESCAN-COVID19Mx Survey. Nutrients. 2021 Nov 26;13(12):4256. doi: 10.3390/nu13124256. PMID: 34959806; PMCID: PMC8706190.

1535: Kwon S, Lee M, Crowley G, Schwartz T, Zeig-Owens R, Prezant DJ, Liu M, Nolan A. Dynamic Metabolic Risk Profiling of World Trade Center Lung Disease: A Longitudinal Cohort Study. Am J Respir Crit Care Med. 2021 Nov 1;204(9):1035-1047. doi: 10.1164/rccm.202006-2617OC. PMID: 34473012; PMCID: PMC8663002.

1536: Christidis R, Lock M, Walker T, Egan M, Browne J. Concerns and priorities of Aboriginal and Torres Strait Islander peoples regarding food and nutrition: a systematic review of qualitative evidence. Int J Equity Health. 2021 Oct 7;20(1):220. doi: 10.1186/s12939-021-01551-x. PMID: 34620180; PMCID: PMC8499519.

1537: Takenaka T, Hasan A, Marumo T, Kobori H, Inoue T, Miyazaki T, Suzuki H, Nishiyama A, Ishii N, Hayashi M. Klotho supplementation attenuates blood pressure and albuminuria in murine model of IgA nephropathy. J Hypertens. 2021 Aug 1;39(8):1567-1576. doi: 10.1097/HJH.0000000000002845. PMID: 33758157.

1538: Subramaniam S, Dhillon JS, Wan Ahmad WF. Behavioral Theory-Based Framework for Prediabetes Self-Care System-Design Perspectives and Validation Results. Int J Environ Res Public Health. 2021 Aug 31;18(17):9160. doi: 10.3390/ijerph18179160. PMID: 34501750; PMCID: PMC8430489.

1539: Hartono FA, Martin-Arrowsmith PW, Peeters WM, Churchward-Venne TA. The Effects of Dietary Protein Supplementation on Acute Changes in Muscle Protein Synthesis and Longer-Term Changes in Muscle Mass, Strength, and Aerobic Capacity in Response to Concurrent Resistance and Endurance Exercise in Healthy Adults: A Systematic Review. Sports Med. 2022 Feb 3. doi: 10.1007/s40279-021-01620-9. Epub ahead of print. PMID: 35113389.

1540: Endalkachew K, Ferede YM, Derso T, Kebede A. Prevalence and associated factors of undernutrition among adult TB patients attending Amhara National Regional State hospitals, Northwest Ethiopia. J Clin Tuberc Other Mycobact Dis. 2021 Dec 21;26:100291. doi: 10.1016/j.jctube.2021.100291. PMID: 35028435; PMCID: PMC8715103.

1541: Lin Y, Ying YY, Li SX, Wang SJ, Gong QH, Li H. Association between alcohol consumption and metabolic syndrome among Chinese adults. Public Health Nutr. 2021 Oct;24(14):4582-4590. doi: 10.1017/S1368980020004449. Epub 2020 Nov 10. PMID: 33168121.

1542: Sun Y, Liu B, Rong S, Zhang J, Du Y, Xu G, Snetselaar LG, Wallace RB, Lehmler HJ, Bao W. Association of Seafood Consumption and Mercury Exposure With Cardiovascular and All-Cause Mortality Among US Adults. JAMA Netw Open. 2021 Nov 1;4(11):e2136367. doi: 10.1001/jamanetworkopen.2021.36367. PMID: 34842923; PMCID: PMC8630568.

1543: He S, Le NA, Ramìrez-Zea M, Martorell R, Narayan KMV, Stein AD. Postprandial glycemic response differed by early life nutritional exposure in a longitudinal cohort: a single- and multi-biomarker approach. Eur J Nutr. 2021 Jun;60(4):1973-1984. doi: 10.1007/s00394-020-02389-9. Epub 2020 Sep 24. PMID: 32970235; PMCID: PMC7987862.

1544: Papadimitriou N, Bouras E, van den Brandt PA, Muller DC, Papadopoulou A, Heath AK, Critselis E, Gunter MJ, Vineis P, Ferrari P, Weiderpass E, Boeing H, Bastide N, Merritt MA, Lopez DS, Bergmann MM, Perez-Cornago A, Schulze M, Skeie G, Srour B, Eriksen AK, Boden S, Johansson I, Nøst TH, Lukic M, Ricceri F, Ericson U, Huerta JM, Dahm CC, Agnoli C, Amiano PE, Tjønneland A, Gurrea AB, Bueno-de-Mesquita B, Ardanaz E, Berntsson J, Sánchez MJ, Tumino R, Panico S, Katzke V, Jakszyn P, Masala G, Derksen JWG, Quirós JR, Severi G, Cross AJ, Riboli E, Tzoulaki I, Tsilidis KK. A Prospective Diet-Wide Association Study for

Risk of Colorectal Cancer in EPIC. Clin Gastroenterol Hepatol. 2021 Apr 24:S1542-3565(21)00462-6. doi: 10.1016/j.cgh.2021.04.028. Epub ahead of print. PMID: 33901663.

1545: Oliai Araghi S, Kiefte-de Jong JC, van Dijk SC, Swart KMA, Ploegmakers KJ, Zillikens MC, van Schoor NM, de Groot LCPGM, Lips P, Stricker BH, Uitterlinden AG, van der Velde N. Long-term effects of folic acid and vitamin-B12 supplementation on fracture risk and cardiovascular disease: Extended follow-up of the B-PROOF trial. Clin Nutr. 2021 Mar;40(3):1199-1206. doi: 10.1016/j.clnu.2020.07.033. Epub 2020 Aug 5. PMID: 32800386.

1546: de Moura E Dias M, Dos Reis Louzano SA, da Conceição LL, da Conceição Fernandes R, de Oliveira Mendes TA, Pereira SS, de Oliveira LL, Gouveia Peluzio MDC. Antibiotic Followed by a Potential Probiotic Increases Brown Adipose Tissue, Reduces Biometric Measurements, and Changes Intestinal Microbiota Phyla in Obesity. Probiotics Antimicrob Proteins. 2021 Dec;13(6):1621-1631. doi: 10.1007/s12602-021-09760-0. Epub 2021 Apr 5. PMID: 33818711.

1547: Svensson T, Sawada N, Mimura M, Nozaki S, Shikimoto R, Tsugane S. Midlife intake of the isoflavone genistein and soy, and the risk of late-life cognitive impairment: the JPHC Saku Mental Health Study. J Epidemiol. 2021 Dec 18. doi: 10.2188/jea.JE20210199. Epub ahead of print. PMID: 34924453.

1548: Cicero AFG, Kennedy C, Knežević T, Bove M, Georges CMG, Šatrauskienė A, Toth PP, Fogacci F. Efficacy and Safety of Armolipid Plus®: An Updated PRISMA Compliant Systematic Review and Meta-Analysis of Randomized Controlled Clinical Trials. Nutrients. 2021 Feb 16;13(2):638. doi: 10.3390/nu13020638. PMID: 33669333; PMCID: PMC7920267.

1549: Williams AR, Wilson-Genderson M, Thomson MD. A cross-sectional analysis of associations between lifestyle advice and behavior changes in patients with hypertension or diabetes: NHANES 2015-2018. Prev Med. 2021 Apr;145:106426. doi: 10.1016/j.ypmed.2021.106426. Epub 2021 Jan 12. PMID: 33450214.

1550: Petrov ME, Jiao N, Panchanathan SS, Reifsnider E, Coonrod DV, Liu L, Krajmalnik-Brown R, Gu H, Davidson LA, Chapkin RS, Whisner CM. Protocol of the Snuggle Bug/Acurrucadito Study: a longitudinal study investigating the influences of sleep-wake patterns and gut microbiome development in infancy on rapid weight gain, an early risk factor for obesity. BMC Pediatr. 2021 Aug 31;21(1):374. doi: 10.1186/s12887-021-02832-8. PMID: 34465311; PMCID: PMC8405858.

1551: Whitley A, Yahia N. Efficacy of Clinic-Based Telehealth vs. Face-to-Face Interventions for Obesity Treatment in Children and Adolescents in the United States and Canada: A Systematic Review. Child Obes. 2021 Jul;17(5):299-310. doi: 10.1089/chi.2020.0347. Epub 2021 Apr 29. PMID: 33926238.

1552: Urbano T, Filippini T, Lasagni D, De Luca T, Grill P, Sucato S, Polledri E, Djeukeu Noumbi G, Malavolti M, Santachiara A, Pertinhez TA, Baricchi R,

Fustinoni S, Michalke B, Vinceti M. Association of Urinary and Dietary Selenium and of Serum Selenium Species with Serum Alanine Aminotransferase in a Healthy Italian Population. Antioxidants (Basel). 2021 Sep 24;10(10):1516. doi: 10.3390/antiox10101516. PMID: 34679651; PMCID: PMC8532767.

1553: Papamichael MM, Katsardis C, Tsoukalas D, Lambert K, Erbas B, Itsiopoulos C. Potential role of folate status on pulmonary function in pediatric asthma. Nutrition. 2021 Oct;90:111267. doi: 10.1016/j.nut.2021.111267. Epub 2021 Apr 7. PMID: 33979761.

1554: Lechien JR, Huet K, Finck C, Blecic S, Delvaux V, Piccaluga M, Saussez S, Harmegnies B. Are the Acoustic Measurements Reliable in the Assessment of Voice Quality? A Methodological Prospective Study. J Voice. 2021 Mar;35(2):203-215. doi: 10.1016/j.jvoice.2019.08.022. Epub 2019 Sep 23. PMID: 31558334.

1555: Knight MG, Anekwe C, Washington K, Akam EY, Wang E, Stanford FC. Weight regulation in menopause. Menopause. 2021 May 24;28(8):960-965. doi: 10.1097/GME.0000000000001792. PMID: 34033603; PMCID: PMC8373626.

1556: de Juras AR, Hsu WC, Hu SC. The Double Burden of Malnutrition at the Individual Level Among Adults: A Nationwide Survey in the Philippines. Front Nutr. 2021 Nov 15;8:760437. doi: 10.3389/fnut.2021.760437. PMID: 34869531; PMCID: PMC8634133.

1557: Walley SN, Krumm EA, Yasrebi A, Kwiecinski J, Wright V, Baker C, Roepke TA. Maternal organophosphate flame-retardant exposure alters offspring energy and glucose homeostasis in a sexually dimorphic manner in mice. J Appl Toxicol. 2021 Apr;41(4):572-586. doi: 10.1002/jat.4066. Epub 2020 Sep 24. PMID: 32969501.

1558: Fortuna R, Hart DA, Sharkey KA, Schachar RA, Johnston K, Reimer RA. Effect of a prebiotic supplement on knee joint function, gut microbiota, and inflammation in adults with co-morbid obesity and knee osteoarthritis: study protocol for a randomized controlled trial. Trials. 2021 Apr 7;22(1):255. doi: 10.1186/s13063-021-05212-w. PMID: 33827639; PMCID: PMC8025512.

1559: Hugo C, Weihprecht H, Banas B, Schröppel B, Jank S, Arns W, Schenker P, Rath T, Hergesell O, Feldkamp T, Hermann B, Schiffer M. Renal Function and Patient-Reported Outcomes in Stable Kidney Transplant Patients Following Conversion From Twice-Daily Immediate-Release Tacrolimus to Once-Daily Prolonged-Release Tacrolimus: A 12-Month Observational Study in Routine Clinical Practice in Germany (ADAGIO). Transplant Proc. 2021 Jun;53(5):1484-1493. doi: 10.1016/j.transproceed.2021.01.034. Epub 2021 Feb 17. PMID: 33610306.

1560: Siqueira de Andrade MI, Oliveira JS, Leal VS, Cabral PC, Lira PIC. Independent predictors of insulin resistance in Brazilian adolescents: Results of the study of cardiovascular risk in adolescents-Brazil. PLoS One. 2021 Feb 9;16(2):e0246445. doi: 10.1371/journal.pone.0246445. PMID: 33561171; PMCID: PMC7872259.

1561: Bassatne A, Basbous M, Chakhtoura M, El Zein O, Rahme M, El-Hajj Fuleihan G. The link between COVID-19 and VItamin D (VIVID): A systematic review and meta-analysis. Metabolism. 2021 Jun;119:154753. doi: 10.1016/j.metabol.2021.154753. Epub 2021 Mar 24. PMID: 33774074; PMCID: PMC7989070.

1562: Fryer S, Stone K, Paterson C, Brown M, Faulkner J, Lambrick D, Credeur D, Zieff G, Martínez Aguirre-Betolaza A, Stoner L. Central and peripheral arterial stiffness responses to uninterrupted prolonged sitting combined with a high-fat meal: a randomized controlled crossover trial. Hypertens Res. 2021 Oct;44(10):1332-1340. doi: 10.1038/s41440-021-00708-z. Epub 2021 Aug 2. PMID: 34334790; PMCID: PMC8490151.

1563: Slowik V, Wasserkrug H, Fischer RT, Connelly M, Deacy AD, Hampl S, Daniel JF. Readiness to Change and Prospective Effects of Weight Management Programs in Pediatric Nonalcoholic Fatty Liver Disease. Clin Transl Sci. 2021 Mar;14(2):582-588. doi: 10.1111/cts.12913. Epub 2020 Nov 22. PMID: 33142354; PMCID: PMC7993262.

1564: Colombo F, Di Lorenzo C, Petroni K, Silano M, Pilu R, Falletta E, Biella S, Restani P. Pigmented Corn Varieties as Functional Ingredients for Gluten-Free Products. Foods. 2021 Jul 30;10(8):1770. doi: 10.3390/foods10081770. PMID: 34441547; PMCID: PMC8392392.

1565: Bockus LB, Biggs ML, Lai HTM, de Olivera Otto MC, Fretts AM, McKnight B, Sotoodehnia N, King IB, Song X, Siscovick DS, Mozaffarian D, Lemaitre RN. Assessment of Plasma Phospholipid Very-Long-Chain Saturated Fatty Acid Levels and Healthy Aging. JAMA Netw Open. 2021 Aug 2;4(8):e2120616. doi: 10.1001/jamanetworkopen.2021.20616. PMID: 34383061; PMCID: PMC8571866.

1566: Nishi SK, Viguiliouk E, Blanco Mejia S, Kendall CWC, Bazinet RP, Hanley AJ, Comelli EM, Salas Salvadó J, Jenkins DJA, Sievenpiper JL. Are fatty nuts a weighty concern? A systematic review and meta-analysis and dose-response meta-regression of prospective cohorts and randomized controlled trials. Obes Rev. 2021 Nov;22(11):e13330. doi: 10.1111/obr.13330. Epub 2021 Sep 8. PMID: 34494363.

1567: Litwin L, Sundholm JKM, Meinilä J, Kulmala J, Tammelin TH, Rönö K, Koivusalo SB, Eriksson JG, Sarkola T. Ideal Cardiovascular Health and Vascular Phenotype Associations in Mothers with Obesity and Their Six-Year-Old Children. Diabetes Metab Syndr Obes. 2021 Jul 13;14:3187-3197. doi: 10.2147/DMSO.S315402. PMID: 34285526; PMCID: PMC8286111.

1568: Du L, La X, Zhu L, Jiang H, Xu B, Chen A, Li M. Utilization of preconception care and its impacts on health behavior changes among expectant couples in Shanghai, China. BMC Pregnancy Childbirth. 2021 Jul 7;21(1):491. doi: 10.1186/s12884-021-03940-0. PMID: 34233653; PMCID: PMC8262048.

1569: Nair-Shalliker V, Smith DP, Gebski V, Patel MI, Frydenberg M, Yaxley JW, Gardiner R, Espinoza D, Kimlin MG, Fenech M, Gillatt D, Woo H, Armstrong BK,

Rasiah K, Awad N, Symons J, Gurney H. High-dose vitamin D supplementation to prevent prostate cancer progression in localised cases with low-to-intermediate risk of progression on active surveillance (ProsD): protocol of a phase II randomised controlled trial. BMJ Open. 2021 Mar 2;11(3):e044055. doi: 10.1136/bmjopen-2020-044055. PMID: 33653757; PMCID: PMC7929872.

1570: Albert U, Losurdo P, Leschiutta A, Macchi S, Samardzic N, Casaganda B, de Manzini N, Palmisano S. Effect of SARS-CoV-2 (COVID-19) Pandemic and Lockdown on Body Weight, Maladaptive Eating Habits, Anxiety, and Depression in a Bariatric Surgery Waiting List Cohort. Obes Surg. 2021 May;31(5):1905-1911. doi: 10.1007/s11695-021-05257-5. Epub 2021 Feb 21. PMID: 33611765; PMCID: PMC7896875.

1571: Godos J, Micek A, Brzostek T, Toledo E, Iacoviello L, Astrup A, Franco OH, Galvano F, Martinez-Gonzalez MA, Grosso G. Egg consumption and cardiovascular risk: a dose-response meta-analysis of prospective cohort studies. Eur J Nutr. 2021 Jun;60(4):1833-1862. doi: 10.1007/s00394-020-02345-7. Epub 2020 Aug 31. PMID: 32865658; PMCID: PMC8137614.

1572: Appelhans BM, Thomas AS, Roisman GI, Booth-LaForce C, Bleil ME. Preexisting Executive Function Deficits and Change in Health Behaviors During the COVID-19 Pandemic. Int J Behav Med. 2021 Dec;28(6):813-819. doi: 10.1007/s12529-021-09974-0. Epub 2021 Mar 2. PMID: 33649889; PMCID: PMC7920747.

1573: Bragg AE, Crowe-White KM, Ellis AC, Studer M, Phillips F, Samsel S, Parton J, Locher JL, Ard JD. Changes in Cardiometabolic Risk Among Older Adults with Obesity: An Ancillary Analysis of a Randomized Controlled Trial Investigating Exercise Plus Weight Maintenance and Exercise Plus Intentional Weight Loss by Caloric Restriction. J Acad Nutr Diet. 2022 Feb;122(2):354-362. doi: 10.1016/j.jand.2021.07.009. Epub 2021 Sep 1. PMID: 34486528; PMCID: PMC8792147.

1574: Alfawaz H, Khan N, Alqahtani S, Ansari MGA, Khattak MNK, Aljumah MA, Al-Daghri NM. Difference on the prevalence, patterns and awareness of soft drink consumption among male and female Arab students. J Public Health (Oxf). 2021 Dec 10;43(4):e657-e666. doi: 10.1093/pubmed/fdaa177. PMID: 33097929.

1575: Wang J, You D, Wang H, Yang Y, Zhang D, Lv J, Luo S, Liao R, Ma L. Association between homocysteine and obesity: A meta-analysis. J Evid Based Med. 2021 Sep;14(3):208-217. doi: 10.1111/jebm.12412. Epub 2020 Nov 3. PMID: 33145936.

1576: Simakova AV, Chitnis N, Babkina IB, Fedorova OS, Fedotova MM, Babkin AM, Khodkevich NE. Abundance of <i>Opisthorchis felineus</i> Metacercariae in cyprinid fish in the middle Ob River basin (Tomsk region, Russia). Food Waterborne Parasitol. 2021 Feb 5;22:e00113. doi: 10.1016/j.fawpar.2021.e00113. PMID: 33681491; PMCID: PMC7930129.

1577: Sadeghi F, Javid AZ, Nazarinasab M, Haghighi-Zadeh MH. Effects of PMS50 supplementation on psychological symptoms of students with premenstrual syndrome. Int J Gynaecol Obstet. 2022 Feb;156(2):247-255. doi:

10.1002/ijgo.13703. Epub 2021 May 29. PMID: 33837571.

1578: Guazzelli Williamson V, Lee AM, Miller D, Huo T, Maner JK, Cardel M. Psychological Resilience, Experimentally Manipulated Social Status, and Dietary Intake among Adolescents. Nutrients. 2021 Mar 1;13(3):806. doi: 10.3390/nu13030806. PMID: 33804409; PMCID: PMC7998543.

1579: Mendham AE, Lundin-Olsson L, Goedecke JH, Micklesfield LK, Christensen DL, Gallagher IJ, Myburgh KH, Odunitan-Wayas FA, Lambert EV, Kalula S, Hunter AM, Brooks NE. Sarcopenic Obesity in Africa: A Call for Diagnostic Methods and Appropriate Interventions. Front Nutr. 2021 Apr 16;8:661170. doi: 10.3389/fnut.2021.661170. PMID: 33937309; PMCID: PMC8085278.

1580: Wang Y, He D, Fu C, Dong X, Jiang F, Su M, Xu Q, Huang P, Wang N, Chen Y, Jiang Q. Thyroid Function Changes and Pubertal Progress in Females: A Longitudinal Study in Iodine-Sufficient Areas of East China. Front Endocrinol (Lausanne). 2021 May 11;12:653680. doi: 10.3389/fendo.2021.653680. PMID: 34046012; PMCID: PMC8146907.

1581: India State-Level Disease Burden Initiative Neurological Disorders Collaborators. The burden of neurological disorders across the states of India: the Global Burden of Disease Study 1990-2019. Lancet Glob Health. 2021 Aug;9(8):e1129-e1144. doi: 10.1016/S2214-109X(21)00164-9. Epub 2021 Jul 14. PMID: 34273302; PMCID: PMC8295043.

1582: Balomenos V, Ntanasi E, Anastasiou CA, Charisis S, Velonakis G, Karavasilis E, Tsapanou A, Yannakoulia M, Kosmidis MH, Dardiotis E, Hadjigeorgiou G, Sakka P, Scarmeas N. Association Between Sleep Disturbances and Frailty: Evidence From a Population-Based Study. J Am Med Dir Assoc. 2021 Mar;22(3):551-558.e1. doi: 10.1016/j.jamda.2020.08.012. Epub 2020 Sep 25. PMID: 32988763.

1583: Imbert A, Vialaneix N, Marquis J, Vion J, Charpagne A, Metairon S, Laurens C, Moro C, Boulet N, Walter O, Lefebvre G, Hager J, Langin D, Saris WHM, Astrup A, Viguerie N, Valsesia A. Network Analyses Reveal Negative Link Between Changes in Adipose Tissue GDF15 and BMI During Dietary-induced Weight Loss. J Clin Endocrinol Metab. 2022 Jan 1;107(1):e130-e142. doi: 10.1210/clinem/dgab621. PMID: 34415992.

1584: Clasen JL, Heath AK, Van Puyvelde H, Huybrechts I, Park JY, Ferrari P, Johansson M, Scelo G, Ulvik A, Midttun Ø, Ueland PM, Dahm CC, Halkjær J, Olsen A, Johnson T, Katzke V, Schulze MB, Masala G, Segrado F, de Magistris MS, Sacerdote C, Ocké MC, Luján-Barroso L, Ching-López A, Huerta JM, Ardanaz E, Amiano P, Ericson U, Manjer J, Gylling B, Johansson I, Schmidt J, Weiderpass E, Riboli E, Cross AJ, Muller DC. A comparison of complementary measures of vitamin B6 status, function, and metabolism in the European Prospective Investigation into Cancer and Nutrition (EPIC) study. Am J Clin Nutr. 2021 Jul 1;114(1):338-347. doi: 10.1093/ajcn/nqab045. PMID: 33829249; PMCID: PMC8246608.

1585: Alustiza E, Perales A, Mateo-Abad M, Ozcoidi I, Aizpuru G, Albaina O, Vergara I; en representación del Grupo PRE-STARt Euskadi. Tackling risk factors for type 2 diabetes in adolescents: PRE-STARt study in Euskadi. An Pediatr (Engl Ed). 2021 Sep;95(3):186-196. doi: 10.1016/j.anpede.2020.11.005. Epub 2021 Aug 9. PMID: 34384737.

1586: Souheil H, Clara R, Hala S, Mirna W, Pascale S. The Preschool Asthma Risk Factors Scale: A predictive tool for asthma and respiratory symptoms among preschool children in Lebanon. Allergol Immunopathol (Madr). 2021 Jul 1;49(4):38-46. doi: 10.15586/aei.v49i4.97. PMID: 34224217.

1587: Roudi F, Khademi G, Ranjbar G, Pouryazdanpanah M, Pahlavani N, Boskabady A, Sezavar M, Nematy M. The effects of implementation of a stepwise algorithmic protocol for nutrition care process in gastro-intestinal surgical children in Pediatric Intensive Care Unit (PICU). Clin Nutr ESPEN. 2021 Jun;43:250-258. doi: 10.1016/j.clnesp.2021.04.004. Epub 2021 Apr 22. PMID: 34024524.

1588: Ihsen Z, Khadija M, Marwa C, Imtinen BM, Fethia BM, Sofien K, Sondos K. Étude des facteurs de mauvaise qualité de vie de l'insuffisant cardiaque chronique à fraction d'éjection altérée [Study of the factors contributing to poor quality of life in chronic heart failure with reduced ejection fraction]. Ann Cardiol Angeiol (Paris). 2021 Oct;70(4):231-236. French. doi: 10.1016/j.ancard.2021.07.004. Epub 2021 Sep 10. PMID: 34517976.

1589: Vasudevan B, Karunakaran U, Antony A, Ramachandran R. Vitamin D status and associated factors among peri menopausal women in two selected districts of Kerala. Indian J Public Health. 2021 Apr-Jun;65(2):166-171. doi: 10.4103/ijph.IJPH_760_20. PMID: 34135186.

1590: Bernal-Jiménez MÁ, Calle-Pérez G, Gutiérrez-Barrios A, Gheorghe L, Solano-Mulero AM, Rodríguez-Martín A, Tur JA, Vázquez-García R, Santi-Cano MJ. Lifestyle and Treatment Adherence Intervention after a Coronary Event Based on an Interactive Web Application (EVITE): Randomized Controlled Clinical Trial Protocol. Nutrients. 2021 May 27;13(6):1818. doi: 10.3390/nu13061818. PMID: 34071782; PMCID: PMC8226528.

1591: Ramos da Silva B, Rufato S, Mialich MS, Cruz LP, Gozzo T, Jordao AA. Metabolic syndrome and unfavorable outcomes on body composition and in visceral adiposities indexes among early breast cancer women post-chemotherapy. Clin Nutr ESPEN. 2021 Aug;44:306-315. doi: 10.1016/j.clnesp.2021.06.001. Epub 2021 Jun 9. PMID: 34330483.

1592: Xie B, Shi X, Li Y, Xia B, Zhou J, Du M, Xing X, Bai L, Liu E, Alvarez F, Jin L, Deng S, Mitchell GA, Pan D, Li M, Wu J. Deficiency of ASGR1 in pigs recapitulates reduced risk factor for cardiovascular disease in humans. PLoS Genet. 2021 Nov 11;17(11):e1009891. doi: 10.1371/journal.pgen.1009891. PMID: 34762653; PMCID: PMC8584755.

1593: Camp-Spivey LJ, Newman SD, Stevens RN, Nichols M. "We've Had to Build the

Plane as We Flew It.": Impacts of the COVID-19 Pandemic on School-Based Weight Management Interventions. Child Obes. 2021 Dec;17(8):497-506. doi: 10.1089/chi.2021.0037. Epub 2021 Jul 1. PMID: 34197215.

1594: Chudasama YV, Zaccardi F, Gillies CL, Razieh C, Yates T, Kloecker DE, Rowlands AV, Davies MJ, Islam N, Seidu S, Forouhi NG, Khunti K. Patterns of multimorbidity and risk of severe SARS-CoV-2 infection: an observational study in the U.K. BMC Infect Dis. 2021 Sep 4;21(1):908. doi: 10.1186/s12879-021-06600-y. PMID: 34481456; PMCID: PMC8418288.

1595: Dover S, Stephens S, Schneiderman JE, Pullenayegum E, Wells GD, Levy DM, Marcuz JA, Whitney K, Schulze A, Tein I, Feldman BM. The Effect of Creatine Supplementation on Muscle Function in Childhood Myositis: A Randomized, Double-blind, Placebo-controlled Feasibility Study. J Rheumatol. 2021 Mar;48(3):434-441. doi: 10.3899/jrheum.191375. Epub 2020 Aug 1. PMID: 32739897.

1596: Karpyn A, Headley MG, Knowles Z, Hepburn E, Kennedy N, Wolgast HK, Riser D, Osei Sarfo AR. Validity of the Food Insecurity Experience Scale and prevalence of food insecurity in The Bahamas. Rural Remote Health. 2021 Nov;21(4):6724. doi: 10.22605/RRH6724. Epub 2021 Nov 10. PMID: 34753291.

1597: Dong JY, Kimura T, Ikehara S, Cui M, Kawanishi Y, Kimura T, Ueda K, Iso H; Japan Environment and Children’s Study Group. Soy consumption and incidence of gestational diabetes mellitus: the Japan Environment and Children's Study. Eur J Nutr. 2021 Mar;60(2):897-904. doi: 10.1007/s00394-020-02294-1. Epub 2020 Jun 6. PMID: 32506178.

1598: Jewell A, Williams H, Hoad CL, Gellert PR, Ashford MB, Butler J, Stolnik S, Scurr D, Stocks MJ, Marciani L, Gowland PA, Gershkovich P. Assessing Lymphatic Uptake of Lipids Using Magnetic Resonance Imaging: A Feasibility Study in Healthy Human Volunteers with Potential Application for Tracking Lymph Node Delivery of Drugs and Formulation Excipients. Pharmaceutics. 2021 Aug 27;13(9):1343. doi: 10.3390/pharmaceutics13091343. PMID: 34575420; PMCID: PMC8470042.

1599: Hackett DA, Mitchell L, Wilson GC, Valenzuela T, Hollings M, Fiatarone Singh M. A Case Study of an 87-Year-Old Male Bodybuilder with Complex Health Conditions. Medicina (Kaunas). 2021 Jun 28;57(7):664. doi: 10.3390/medicina57070664. PMID: 34203123; PMCID: PMC8306742.

1600: Jia F, Liu F, Li X, Shi X, Liu Y, Cao F. Cognitive reserve, modifiable-risk-factor profile and incidence of dementia: results from a longitudinal study of CFAS Wales. Aging Ment Health. 2021 Dec;25(12):2286-2292. doi: 10.1080/13607863.2020.1828270. Epub 2020 Oct 6. PMID: 33021096.

1601: Bakhtiary M, Morvaridzadeh M, Agah S, Rahimlou M, Christopher E, Zadro JR, Heshmati J. Effect of Probiotic, Prebiotic, and Synbiotic Supplementation on Cardiometabolic and Oxidative Stress Parameters in Patients With Chronic Kidney Disease: A Systematic Review and Meta-analysis. Clin Ther. 2021

Mar;43(3):e71-e96. doi: 10.1016/j.clinthera.2020.12.021. Epub 2021 Jan 30. PMID: 33526314.

1602: Foster H, Polz P, Mair F, Gill J, O'Donnell CA. Understanding the influence of socioeconomic status on the association between combinations of lifestyle factors and adverse health outcomes: a systematic review protocol. BMJ Open. 2021 May 27;11(5):e042212. doi: 10.1136/bmjopen-2020-042212. PMID: 34045211; PMCID: PMC8162079.

1603: Crivelli JJ, Maalouf NM, Paiste HJ, Wood KD, Hughes AE, Oates GR, Assimos DG. Disparities in Kidney Stone Disease: A Scoping Review. J Urol. 2021 Sep;206(3):517-525. doi: 10.1097/JU.0000000000001846. Epub 2021 Apr 27. PMID: 33904797; PMCID: PMC8355087.

1604: Mohan D, Mente A, Dehghan M, Rangarajan S, O'Donnell M, Hu W, Dagenais G, Wielgosz A, Lear S, Wei L, Diaz R, Avezum A, Lopez-Jaramillo P, Lanas F, Swaminathan S, Kaur M, Vijayakumar K, Mohan V, Gupta R, Szuba A, Iqbal R, Yusuf R, Mohammadifard N, Khatib R, Yusoff K, Gulec S, Rosengren A, Yusufali A, Wentzel-Viljoen E, Chifamba J, Dans A, Alhabib KF, Yeates K, Teo K, Gerstein HC, Yusuf S; PURE, ONTARGET, TRANSCEND, and ORIGIN investigators. Associations of Fish Consumption With Risk of Cardiovascular Disease and Mortality Among Individuals With or Without Vascular Disease From 58 Countries. JAMA Intern Med. 2021 May 1;181(5):631-649. doi: 10.1001/jamainternmed.2021.0036. Erratum in: JAMA Intern Med. 2021 May 1;181(5):727. PMID: 33683310; PMCID: PMC7941252.

1605: Salvat H, Mohammadi MN, Molavi P, Mostafavi SA, Rostami R, Salehinejad MA. Nutrient intake, dietary patterns, and anthropometric variables of children with ADHD in comparison to healthy controls: a case-control study. BMC Pediatr. 2022 Jan 29;22(1):70. doi: 10.1186/s12887-022-03123-6. PMID: 35093041; PMCID: PMC8800296.

1606: Feehan J, Degabrielle E, Tripodi N, Al Saedi A, Vogrin S, Duque G. The effect of vitamin D supplementation on circulating osteoprogenitor cells: A pilot randomized controlled trial. Exp Gerontol. 2021 Jul 15;150:111399. doi: 10.1016/j.exger.2021.111399. Epub 2021 May 7. PMID: 33971278.

1607: Gribble AK, Sayón-Orea C, Bes-Rastrollo M, Kales SN, Shirahama R, Martínez-González MÁ, Fernandez-Montero A. Risk of Developing Metabolic Syndrome Is Affected by Length of Daily Siesta: Results from a Prospective Cohort Study. Nutrients. 2021 Nov 22;13(11):4182. doi: 10.3390/nu13114182. PMID: 34836438; PMCID: PMC8619148.

1608: Wu L, Liu C, Chang DY, Zhan R, Zhao M, Man Lam S, Shui G, Zhao MH, Zheng L, Chen M. The Attenuation of Diabetic Nephropathy by Annexin A1 via Regulation of Lipid Metabolism Through the AMPK/PPARα/CPT1b Pathway. Diabetes. 2021 Oct;70(10):2192-2203. doi: 10.2337/db21-0050. Epub 2021 Jun 8. PMID: 34103347.

1609: Kryczyk-Kozioł J, Zagrodzki P, Prochownik E, Błażewska-Gruszczyk A, Słowiaczek M, Sun Q, Schomburg L, Ochab E, Bartyzel M. Positive effects of

selenium supplementation in women with newly diagnosed Hashimoto's thyroiditis in an area with low selenium status. Int J Clin Pract. 2021 Sep;75(9):e14484. doi: 10.1111/ijcp.14484. Epub 2021 Jun 30. PMID: 34107151.

1610: Foulkes S, Kukuljan S, Nowson CA, Sanders KM, Daly RM. Effects of a multi-modal resistance exercise program and calcium-vitamin D₃ fortified milk on blood pressure and blood lipids in middle-aged and older men: secondary analysis of an 18-month factorial design randomised controlled trial. Eur J Nutr. 2021 Apr;60(3):1289-1299. doi: 10.1007/s00394-020-02325-x. Epub 2020 Jul 14. PMID: 32666313.

1611: Chalasani NP, Maddur H, Russo MW, Wong RJ, Reddy KR; Practice Parameters Committee of the American College of Gastroenterology. ACG Clinical Guideline: Diagnosis and Management of Idiosyncratic Drug-Induced Liver Injury. Am J Gastroenterol. 2021 May 1;116(5):878-898. doi: 10.14309/ajg.0000000000001259. PMID: 33929376.

1612: Mahalhal A, Frau A, Burkitt MD, Ijaz UZ, Lamb CA, Mansfield JC, Lewis S, Pritchard DM, Probert CS. Oral Ferric Maltol Does Not Adversely Affect the Intestinal Microbiome of Patients or Mice, But Ferrous Sulphate Does. Nutrients. 2021 Jun 30;13(7):2269. doi: 10.3390/nu13072269. PMID: 34209042; PMCID: PMC8308237.

1613: Hosseini R, Montazerifar F, Shahraki E, Karajibani M, Mokhtari AM, Dashipour AR, Ferns GA, Jalali M. The Effects of Zinc Sulfate Supplementation on Serum Copeptin, C-Reactive Protein and Metabolic Markers in Zinc-Deficient Diabetic Patients on Hemodialysis: A Randomized, Double-Blind, Placebo-Controlled Trial. Biol Trace Elem Res. 2022 Jan;200(1):76-83. doi: 10.1007/s12011-021-02649-7. Epub 2021 Mar 3. PMID: 33655432.

1614: Khodashenas M, Mardi P, Taherzadeh-Ghahfarokhi N, Tavakoli-Far B, Jamee M, Ghodrati N. Quality of Life and Related Paraclinical Factors in Iranian Patients with Transfusion-Dependent Thalassemia. J Environ Public Health. 2021 Aug 18;2021:2849163. doi: 10.1155/2021/2849163. PMID: 34457009; PMCID: PMC8387194.

1615: Lee HH, Sudhakara P, Desai S, Miranda K, Martinez LR. Understanding the Basis of METH Mouth Using a Rodent Model of Methamphetamine Injection, Sugar Consumption, and Streptococcus mutans Infection. mBio. 2021 Mar 9;12(2):e03534-20. doi: 10.1128/mBio.03534-20. PMID: 33688011; PMCID: PMC8092307.

1616: Zerdo Z, Bastiaens H, Anthierens S, Massebo F, Masne M, Biresaw G, Shewangizaw M, Tunje A, Chisha Y, Yohannes T, Van Geertruyden JP. Prevalence and associated risk factors of asymptomatic malaria and anaemia among school-aged children in Dara Mallo and Uba Debretsehay districts: results from baseline cluster randomized trial. Malar J. 2021 Oct 13;20(1):400. doi: 10.1186/s12936-021-03937-2. PMID: 34645464; PMCID: PMC8513194.

1617: Lu MC, Lo HC, Chang HH, Hsu CW, Koo M. Factors associated with the use of

complementary therapies in Taiwanese patients with systemic lupus erythematosus: a cross-sectional study. BMC Complement Med Ther. 2021 Oct 1;21(1):247. doi: 10.1186/s12906-021-03416-w. PMID: 34598699; PMCID: PMC8485480.

1618: Southam K, de Sousa C, Daniel A, Taylor BV, Foa L, Premilovac D. Development and characterisation of a rat model that exhibits both metabolic dysfunction and neurodegeneration seen in type 2 diabetes. J Physiol. 2022 Feb 6. doi: 10.1113/JP282454. Epub ahead of print. PMID: 35128667.

1619: Assmus F, Galbete C, Knueppel S, Schulze MB, Beune E, Meeks K, Nicolaou M, Amoah S, Agyemang C, Klipstein-Grobusch K, Bahendeka S, Spranger J, Mockenhaupt FP, Smeeth L, Stronks K, Danquah I. Carbohydrate-dense snacks are a key feature of the nutrition transition among Ghanaian adults - findings from the RODAM study. Food Nutr Res. 2021 May 6;65. doi: 10.29219/fnr.v65.5435. PMID: 34512231; PMCID: PMC8388941.

1620: Mahrouseh N, Andrade CAS, Kovács N, Njuguna DW, Varga O. Diabetes Mellitus and Associated Factors in Slovakia: Results from the European Health Interview Survey 2009, 2014, and 2019. Nutrients. 2021 Jun 23;13(7):2156. doi: 10.3390/nu13072156. PMID: 34201793; PMCID: PMC8308286.

1621: Medeiros PBS, Salomão RG, Teixeira SR, Rassi DM, Rodrigues L, Aragon DC, Fassini PG, Ued FV, Tostes RC, Monteiro JP, Ferriani VPL, de Carvalho LM. Disease activity index is associated with subclinical atherosclerosis in childhood-onset systemic lupus erythematosus. Pediatr Rheumatol Online J. 2021 Mar 20;19(1):35. doi: 10.1186/s12969-021-00513-5. PMID: 33743717; PMCID: PMC7981852.

1622: Islam SJ, Hwan Kim J, Joseph E, Topel M, Baltrus P, Liu C, Ko YA, Almuwaqqat Z, Mujahid MS, Sims M, Mubasher M, Ejaz K, Searles C, Dunbar SB, Pemu P, Taylor H, Bremner JD, Vaccarino V, Quyyumi AA, Lewis TT. Association Between Early Trauma and Ideal Cardiovascular Health Among Black Americans: Results From the Morehouse-Emory Cardiovascular (MECA) Center for Health Equity. Circ Cardiovasc Qual Outcomes. 2021 Sep;14(9):e007904. doi: 10.1161/CIRCOUTCOMES.121.007904. Epub 2021 Aug 12. PMID: 34380328; PMCID: PMC8455434.

1623: Sia MW, Foo JN, Saffari SE, Wong AS, Khor CC, Yuan JM, Tan EK, Koh WP, Tan LC. Polygenic Risk Scores in a Prospective Parkinson's Disease Cohort. Mov Disord. 2021 Dec;36(12):2936-2940. doi: 10.1002/mds.28761. Epub 2021 Aug 17. PMID: 34402545; PMCID: PMC8688232.

1624: Pacyga DC, Gardiner JC, Flaws JA, Li Z, Calafat AM, Korrick SA, Schantz SL, Strakovsky RS. Maternal phthalate and phthalate alternative metabolites and urinary biomarkers of estrogens and testosterones across pregnancy. Environ Int. 2021 Oct;155:106676. doi: 10.1016/j.envint.2021.106676. Epub 2021 Jun 8. PMID: 34116379; PMCID: PMC8292204.

1625: Mba CM, Koulman A, Forouhi NG, Imamura F, Assah F, Mbanya JC, Wareham NJ.

Associations of Serum Folate and Holotranscobalamin with Cardiometabolic Risk Factors in Rural and Urban Cameroon. Nutrients. 2021 Dec 30;14(1):178. doi: 10.3390/nu14010178. PMID: 35011051; PMCID: PMC8747568.

1626: Andersen ZJ, Hoffmann B, Morawska L, Adams M, Furman E, Yorgancioglu A, Greenbaum D, Neira M, Brunekreef B, Forastiere F, Rice MB, Wakenhut F, Coleen E, Boogaard H, Gehring U, Melén E, Ward B, De Matteis S. Air pollution and COVID-19: clearing the air and charting a post-pandemic course: a joint workshop report of ERS, ISEE, HEI and WHO. Eur Respir J. 2021 Aug 19;58(2):2101063. doi: 10.1183/13993003.01063-2021. PMID: 34385271; PMCID: PMC8361303.

1627: Salerno EA, Saint-Maurice PF, Willis EA, Moore SC, DiPietro L, Matthews CE. Ambulatory Function and Mortality among Cancer Survivors in the NIH-AARP Diet and Health Study. Cancer Epidemiol Biomarkers Prev. 2021 Apr;30(4):690-698. doi: 10.1158/1055-9965.EPI-20-1473. Epub 2021 Mar 4. PMID: 33664017; PMCID: PMC8300589.

1628: Mao QQ, Li BY, Meng JM, Gan RY, Xu XY, Gu YY, Wang XH, Li HB. Effects of several tea extracts on nonalcoholic fatty liver disease in mice fed with a high-fat diet. Food Sci Nutr. 2021 Apr 9;9(6):2954-2967. doi: 10.1002/fsn3.2255. PMID: 34136163; PMCID: PMC8194756.

1629: Yu EYT, Yeung CHN, Wan EYF, Tang EHM, Wong CKH, Cheung BMY, Lam CLK. Association between health behaviours and cardiometabolic dysregulation: a population-based survey among healthy adults in Hong Kong. BMJ Open. 2021 Jul 9;11(7):e043503. doi: 10.1136/bmjopen-2020-043503. PMID: 34244247; PMCID: PMC8273464.

1630: Kang JH, Vyas CM, Okereke OI, Ogata S, Albert M, Lee IM, D'Agostino D, Buring JE, Cook NR, Grodstein F, Manson JE. Effect of vitamin D on cognitive decline: results from two ancillary studies of the VITAL randomized trial. Sci Rep. 2021 Dec 1;11(1):23253. doi: 10.1038/s41598-021-02485-8. PMID: 34853363; PMCID: PMC8636504.

1631: Krause K, Pyrczak-Felczykowska A, Karczewska M, Narajczyk M, Herman-Antosiewicz A, Szalewska-Pałasz A, Nowicki D. Dietary Isothiocyanates, Sulforaphane and 2-Phenethyl Isothiocyanate, Effectively Impair <i>Vibrio cholerae</i> Virulence. Int J Mol Sci. 2021 Sep 22;22(19):10187. doi: 10.3390/ijms221910187. PMID: 34638525; PMCID: PMC8508596.

1632: Poli A, Marangoni F, Corsini A, Manzato E, Marrocco W, Martini D, Medea G, Visioli F. Phytosterols, Cholesterol Control, and Cardiovascular Disease. Nutrients. 2021 Aug 16;13(8):2810. doi: 10.3390/nu13082810. PMID: 34444970; PMCID: PMC8399210.

1633: Jones J, Lembo A, Heidelbaugh J, Kuritzky L, Lacy B. Management of irritable bowel syndrome with diarrhea: focus on eluxadoline. Curr Med Res Opin. 2021 Apr;37(4):567-578. doi: 10.1080/03007995.2021.1888705. Epub 2021 Mar 3. PMID: 33566707.

1634: Lee Y, Nemet I, Wang Z, Lai HTM, de Oliveira Otto MC, Lemaitre RN, Fretts AM, Sotoodehnia N, Budoff M, DiDonato JA, McKnight B, Tang WHW, Psaty BM, Siscovick DS, Hazen SL, Mozaffarian D. Longitudinal Plasma Measures of Trimethylamine N-Oxide and Risk of Atherosclerotic Cardiovascular Disease Events in Community-Based Older Adults. J Am Heart Assoc. 2021 Sep 7;10(17):e020646. doi: 10.1161/JAHA.120.020646. Epub 2021 Aug 16. PMID: 34398665; PMCID: PMC8649305.

1635: Sheftel J, van Stuijvenberg ME, Dhansay MA, Suri DJ, Grahn M, Keuler NS, Binkley NC, Tanumihardjo SA. Chronic and acute hypervitaminosis A are associated with suboptimal anthropometric measurements in a cohort of South African preschool children. Am J Clin Nutr. 2022 Jan 14:nqab422. doi: 10.1093/ajcn/nqab422. Epub ahead of print. PMID: 35030234.

1636: Christiansen MR, Ureña MG, Borisevich D, Grarup N, Martínez JA, Oppert JM, Sørensen TI, Hansen T, Blaak EE, Kilpeläinen TO. Abdominal and gluteofemoral fat depots show opposing associations with postprandial lipemia. Am J Clin Nutr. 2021 Oct 4;114(4):1467-1475. doi: 10.1093/ajcn/nqab219. PMID: 34254976.

1637: D'Alessandro MCO, Gomes AD, Morais JF, Mizubuti YGG, Silva TAD, Fernandes SM, Mendes LL, Correia MITD, Generoso SV. SHORT-TERM EFFECT OF WHEY PROTEIN SUPPLEMENTATION ON THE QUALITY OF LIFE OF PATIENTS WAITING FOR LIVER TRANSPLANTATION: A DOUBLE BLINDED RANDOMIZED CLINICAL TRIAL. Arq Bras Cir Dig. 2021 Oct 15;34(2):e1596. doi: 10.1590/0102-672020210002e1596. PMID: 34669886; PMCID: PMC8521869.

1638: Edbrooke L, Khaw P, Freimund A, Carpenter D, McNally O, Joubert L, Loeliger J, Traill A, Gough K, Mileshkin L, Denehy L. ENhAncing Lifestyle Behaviors in EndometriaL CancEr (ENABLE): A Pilot Randomized Controlled Trial. Integr Cancer Ther. 2022 Jan-Dec;21:15347354211069885. doi: 10.1177/15347354211069885. PMID: 35045735; PMCID: PMC8785429.

1639: Wu K, Zhou Y, Ke S, Huang J, Gao X, Li B, Lin X, Liu X, Liu X, Ma L, Wang L, Wu L, Wu L, Xie C, Xu J, Wang Y, Liu L. Lifestyle is associated with thyroid function in subclinical hypothyroidism: a cross-sectional study. BMC Endocr Disord. 2021 May 28;21(1):112. doi: 10.1186/s12902-021-00772-z. PMID: 34049544; PMCID: PMC8161919.

1640: Chang K, Khandpur N, Neri D, Touvier M, Huybrechts I, Millett C, Vamos EP. Association Between Childhood Consumption of Ultraprocessed Food and Adiposity Trajectories in the Avon Longitudinal Study of Parents and Children Birth Cohort. JAMA Pediatr. 2021 Sep 1;175(9):e211573. doi: 10.1001/jamapediatrics.2021.1573. Epub 2021 Sep 7. PMID: 34125152; PMCID: PMC8424476.

1641: Sandebring-Matton A, Goikolea J, Björkhem I, Paternain L, Kemppainen N, Laatikainen T, Ngandu T, Rinne J, Soininen H, Cedazo-Minguez A, Solomon A, Kivipelto M. 27-Hydroxycholesterol, cognition, and brain imaging markers in the

FINGER randomized controlled trial. Alzheimers Res Ther. 2021 Mar 6;13(1):56. doi: 10.1186/s13195-021-00790-y. PMID: 33676572; PMCID: PMC7937194.

1642: Abu Rass R, Kustin T, Zamostiano R, Smorodinsky N, Ben Meir D, Feder D, Mishra N, Lipkin WI, Eldar A, Ehrlich M, Stern A, Bacharach E. Inferring protein function in an emerging virus: detection of the nucleoprotein in Tilapia Lake Virus. J Virol. 2022 Feb 2:JVI0175721. doi: 10.1128/JVI.01757-21. Epub ahead of print. PMID: 35107373.

1643: D'Addario L, Kuo T, Robles B. Do knowledge about sodium, health status by self-report, and having hypertension predict sodium consumption behaviors among Southern California hospital employees? Transl Behav Med. 2021 Jun 17;11(6):1254-1263. doi: 10.1093/tbm/ibaa148. PMID: 33755177.

1644: Hartung TJ, Moustsen IR, Larsen SB, Wreford Andersen EA, Suppli NP, Johansen C, Tjønneland A, Friberg AS, Kjær SK, Brasso K, Kessing LV, Mehnert A, Dalton SO. Antidepressant prescriptions and associated factors in men with prostate cancer and their female partners. J Cancer Surviv. 2021 Aug;15(4):536-545. doi: 10.1007/s11764-020-00947-y. Epub 2020 Oct 13. PMID: 33051756; PMCID: PMC8272693.

1645: Lari A, Fatahi S, Sohouli MH, Shidfar F. The Impact of Chromium Supplementation on Blood Pressure: A Systematic Review and Dose-Response Meta-Analysis of Randomized-Controlled Trials. High Blood Press Cardiovasc Prev. 2021 Jul;28(4):333-342. doi: 10.1007/s40292-021-00456-8. Epub 2021 Jun 3. PMID: 34081296.

1646: Rondanelli M, Peroni G, Riva A, Petrangolini G, Allegrini P, Fazia T, Bernardinelli L, Naso M, Faliva MA, Tartara A, Gasparri C, Infantino V, Perna S. Bergamot phytosome improved visceral fat and plasma lipid profiles in overweight and obese class I subject with mild hypercholesterolemia: A randomized placebo controlled trial. Phytother Res. 2021 Apr;35(4):2045-2056. doi: 10.1002/ptr.6950. Epub 2020 Nov 13. PMID: 33188552; PMCID: PMC8246838.

1647: Pereira LCR, Shannon OM, Mazidi M, Babateen AM, Ashor AW, Stephan BCM, Siervo M. Relationship between urinary nitrate concentrations and cognitive function in older adults: findings from the NHANES survey. Int J Food Sci Nutr. 2021 Sep;72(6):805-815. doi: 10.1080/09637486.2020.1868411. Epub 2021 Jan 4. PMID: 33397165.

1648: Dangerfield F, Lamb KE, Oostenbach LH, Ball K, Thornton LE. Urban-regional patterns of food purchasing behaviour: a cross-sectional analysis of the 2015-2016 Australian Household Expenditure Survey. Eur J Clin Nutr. 2021 Apr;75(4):697-707. doi: 10.1038/s41430-020-00746-9. Epub 2020 Sep 12. Erratum in: Eur J Clin Nutr. 2020 Nov 2;: PMID: 32920603.

1649: Mitra S, Rauf A, Tareq AM, Jahan S, Emran TB, Shahriar TG, Dhama K, Alhumaydhi FA, Aljohani ASM, Rebezov M, Uddin MS, Jeandet P, Shah ZA, Shariati MA, Rengasamy KR. Potential health benefits of carotenoid lutein: An updated

review. Food Chem Toxicol. 2021 Aug;154:112328. doi: 10.1016/j.fct.2021.112328. Epub 2021 Jun 8. PMID: 34111488.

1650: Chan SW, Chu TTW, Choi SW, Benzie IFF, Tomlinson B. Impact of short-term bilberry supplementation on glycemic control, cardiovascular disease risk factors, and antioxidant status in Chinese patients with type 2 diabetes. Phytother Res. 2021 Jun;35(6):3236-3245. doi: 10.1002/ptr.7038. Epub 2021 Feb 18. PMID: 33599340.

1651: Hernández-Martínez A, Gavilán-Carrera B, Vargas-Hitos JA, Morillas-de-Laguno P, Sola-Rodríguez S, Rosales-Castillo A, Artero EG, Sabio JM, Soriano-Maldonado A. Ideal cardiovascular health in women with systemic lupus erythematosus: Association with arterial stiffness, inflammation, and fitness. Int J Cardiol. 2021 May 1;330:207-213. doi: 10.1016/j.ijcard.2021.02.040. Epub 2021 Feb 20. PMID: 33621624.

1652: Chou SH, Murata EM, Yu C, Danik J, Kotler G, Cook NR, Bubes V, Mora S, Chandler PD, Tobias DK, Copeland T, Buring JE, Manson JE, LeBoff MS. Effects of Vitamin D3 Supplementation on Body Composition in the VITamin D and OmegA-3 TriaL (VITAL). J Clin Endocrinol Metab. 2021 Apr 23;106(5):1377-1388. doi: 10.1210/clinem/dgaa981. PMID: 33513226; PMCID: PMC8063236.

1653: Varsamis NA, Christou GA, Kiortsis DN. A critical review of the effects of vitamin K on glucose and lipid homeostasis: its potential role in the prevention and management of type 2 diabetes. Hormones (Athens). 2021 Sep;20(3):415-422. doi: 10.1007/s42000-020-00268-w. Epub 2021 Jan 16. PMID: 33454929.

1654: Pitchik HO, Tofail F, Rahman M, Akter F, Sultana J, Shoab AK, Huda TMN, Jahir T, Amin MR, Hossain MK, Das JB, Chung EO, Byrd KA, Yeasmin F, Kwong LH, Forsyth JE, Mridha MK, Winch PJ, Luby SP, Fernald LC. A holistic approach to promoting early child development: a cluster randomised trial of a group-based, multicomponent intervention in rural Bangladesh. BMJ Glob Health. 2021 Mar;6(3):e004307. doi: 10.1136/bmjgh-2020-004307. PMID: 33727278; PMCID: PMC7970287.

1655: Camarero C, De Andrés A, García-Hoz C, Roldán B, Muriel A, León F, Roy G. Assessment of Duodenal Intraepithelial Lymphocyte Composition (Lymphogram) for Accurate and Prompt Diagnosis of Celiac Disease in Pediatric Patients. Clin Transl Gastroenterol. 2021 Nov 10;12(11):e00426. doi: 10.14309/ctg.0000000000000426. PMID: 34757327; PMCID: PMC8585297.

1656: Luo L, Lu S, Huang C, Wang F, Ren Y, Cao H, Lin Q, Tan Z, Wen X. A survey of chloramphenicol residues in aquatic products of Shenzhen, South China. Food Addit Contam Part A Chem Anal Control Expo Risk Assess. 2021 Jun;38(6):914-921. doi: 10.1080/19440049.2021.1898680. Epub 2021 Apr 1. PMID: 33792514.

1657: Ten Cate D, Mellema M, Ettema RGA, Schuurmans MJ, Schoonhoven L. Older Adults' and Their Informal Caregivers' Experiences and Needs regarding Nutritional Care Provided in the Periods before, during and after

Hospitalization: A Qualitative Study. J Nutr Gerontol Geriatr. 2021 Apr-Jun;40(2-3):80-107. doi: 10.1080/21551197.2021.1906822. Epub 2021 Apr 9. PMID: 33835889.

1658: Li Q, Guo S, Yang C, Liu X, Chen X, He J, Tong C, Ding Y, Peng C, Geng Y, Mu X, Liu T, Li F, Wang Y, Gao R. High-fat diet-induced obesity primes fatty acid β-oxidation impairment and consequent ovarian dysfunction during early pregnancy. Ann Transl Med. 2021 May;9(10):887. doi: 10.21037/atm-21-2027. PMID: 34164521; PMCID: PMC8184480.

1659: Colijn JM, Meester-Smoor M, Verzijden T, de Breuk A, Silva R, Merle BMJ, Cougnard-Grégoire A, Hoyng CB, Fauser S, Coolen A, Creuzot-Garcher C, Hense HW, Ueffing M, Delcourt C, den Hollander AI, Klaver CCW; EYE-RISK Consortium. Genetic Risk, Lifestyle, and Age-Related Macular Degeneration in Europe: The EYE-RISK Consortium. Ophthalmology. 2021 Jul;128(7):1039-1049. doi: 10.1016/j.ophtha.2020.11.024. Epub 2020 Nov 28. PMID: 33253757.

1660: Eggers S, Safdar N, Kates A, Sethi AK, Peppard PE, Kanarek MS, Malecki KMC. Urinary lead level and colonization by antibiotic resistant bacteria: Evidence from a population-based study. Environ Epidemiol. 2021 Nov 3;5(6):e175. doi: 10.1097/EE9.0000000000000175. PMID: 34909555; PMCID: PMC8663876.

1661: Bouhrara M, Khattar N, Elango P, Resnick SM, Ferrucci L, Spencer RG. Evidence of association between obesity and lower cerebral myelin content in cognitively unimpaired adults. Int J Obes (Lond). 2021 Apr;45(4):850-859. doi: 10.1038/s41366-021-00749-x. Epub 2021 Jan 22. PMID: 33483582; PMCID: PMC8009848.

1662: Sadria M, Layton AT. Interactions among mTORC, AMPK and SIRT: a computational model for cell energy balance and metabolism. Cell Commun Signal. 2021 May 20;19(1):57. doi: 10.1186/s12964-021-00706-1. PMID: 34016143; PMCID: PMC8135154.

1663: do Rosario VA, Fitzgerald Z, Broyd S, Paterson A, Roodenrys S, Thomas S, Bliokas V, Potter J, Walton K, Weston-Green K, Yousefi M, Williams D, Wright IMR, Charlton K. Food anthocyanins decrease concentrations of TNF-α in older adults with mild cognitive impairment: A randomized, controlled, double blind clinical trial. Nutr Metab Cardiovasc Dis. 2021 Mar 10;31(3):950-960. doi: 10.1016/j.numecd.2020.11.024. Epub 2020 Dec 5. PMID: 33546942.

1664: Langton CR, Whitcomb BW, Purdue-Smithe AC, Sievert LL, Hankinson SE, Manson JE, Rosner BA, Bertone-Johnson ER. Association of oral contraceptives and tubal ligation with risk of early natural menopause. Hum Reprod. 2021 Jun 18;36(7):1989-1998. doi: 10.1093/humrep/deab054. PMID: 33822044; PMCID: PMC8487650.

1665: Shahinfar H, Jayedi A, Khan TA, Shab-Bidar S. Coffee consumption and cardiovascular diseases and mortality in patients with type 2 diabetes: A systematic review and dose-response meta-analysis of cohort studies. Nutr Metab Cardiovasc Dis. 2021 Aug 26;31(9):2526-2538. doi: 10.1016/j.numecd.2021.05.014.

Epub 2021 May 24. PMID: 34112583.

1666: Sharma A, Eluri S, Philpott H, Lemberg DA, Dellon ES. EoE Down Under Is Still EoE: Variability in Provider Practice Patterns in Australia and New Zealand Among Pediatric Gastroenterologists. Dig Dis Sci. 2021 Jul;66(7):2301-2310. doi: 10.1007/s10620-020-06534-6. Epub 2020 Aug 20. PMID: 32816214.

1667: Taxová Braunerová R, Kunešová M, Heinen MM, Rutter H, Hassapidou M, Duleva V, Pudule I, Petrauskienė A, Sjöberg A, Lissner L, Spiroski I, Gutiérrez-González E, Kelleher CC, Bergh IH, Metelcová T, Vignerová J, Brabec M, Buoncristiano M, Williams J, Simmonds P, Zamrazilová H, Hainer V, Yngve A, Rakovac I, Breda J. Waist circumference and waist-to-height ratio in 7-year-old children-WHO Childhood Obesity Surveillance Initiative. Obes Rev. 2021 Nov;22 Suppl 6:e13208. doi: 10.1111/obr.13208. Epub 2021 Aug 17. PMID: 34402567.

1668: Ruppert PM, Deng L, Hooiveld GJ, Hangelbroek RW, Zeigerer A, Kersten S. RNA sequencing reveals niche gene expression effects of beta-hydroxybutyrate in primary myotubes. Life Sci Alliance. 2021 Aug 18;4(10):e202101037. doi: 10.26508/lsa.202101037. PMID: 34407998; PMCID: PMC8380668.

1669: Händel MN, Jacobsen R, Thorsteinsdottir F, Keller AC, Stougaard M, Jensen CB, Moos C, Duus KS, Jensen A, Schiøler Kesmodel U, Abrahamsen B, Heitmann BL. Assessing Health Consequences of Vitamin D Fortification Utilizing a Societal Experiment Design: Methodological Lessons Learned from the D-Tect Project. Int J Environ Res Public Health. 2021 Jul 31;18(15):8136. doi: 10.3390/ijerph18158136. PMID: 34360427; PMCID: PMC8345774.

1670: Buoli M, Caldiroli L, Guenzani D, Carnevali GS, Cesari M, Turolo S, Barkin JL, Messa P, Agostoni C, Vettoretti S; DREAM Project Group. Associations Between Cholesterol and Fatty Acid Profile on the Severity of Depression in Older Persons With Nondialysis Chronic Kidney Disease. J Ren Nutr. 2021 Sep;31(5):537-540. doi: 10.1053/j.jrn.2020.08.017. Epub 2020 Dec 29. PMID: 33386204.

1671: Foerster C, Ríos-Gajardo G, Gómez P, Muñoz K, Cortés S, Maldonado C, Ferreccio C. Assessment of Mycotoxin Exposure in a Rural County of Chile by Urinary Biomarker Determination. Toxins (Basel). 2021 Jun 25;13(7):439. doi: 10.3390/toxins13070439. PMID: 34202116; PMCID: PMC8309762.

1672: Neriman A, Hakan Y, Ozge U. The psychotropic effect of vitamin D supplementation on schizophrenia symptoms. BMC Psychiatry. 2021 Jun 15;21(1):309. doi: 10.1186/s12888-021-03308-w. PMID: 34130647; PMCID: PMC8204117.

1673: Chun M, Clarke R, Cairns BJ, Clifton D, Bennett D, Chen Y, Guo Y, Pei P, Lv J, Yu C, Yang L, Li L, Chen Z, Zhu T; China Kadoorie Biobank Collaborative Group. Stroke risk prediction using machine learning: a prospective cohort study of 0.5 million Chinese adults. J Am Med Inform Assoc. 2021 Jul

30;28(8):1719-1727. doi: 10.1093/jamia/ocab068. PMID: 33969418; PMCID: PMC8324240.

1674: Fiorindi C, Cuffaro F, Piemonte G, Cricchio M, Addasi R, Dragoni G, Scaringi S, Nannoni A, Ficari F, Giudici F. Effect of long-lasting nutritional prehabilitation on postoperative outcome in elective surgery for IBD. Clin Nutr. 2021 Mar;40(3):928-935. doi: 10.1016/j.clnu.2020.06.020. Epub 2020 Jul 1. PMID: 32684485.

1675: Zhu M, Wang T, Huang Y, Zhao X, Ding Y, Zhu M, Ji M, Wang C, Dai J, Yin R, Xu L, Ma H, Wei Q, Jin G, Hu Z, Shen H. Genetic Risk for Overall Cancer and the Benefit of Adherence to a Healthy Lifestyle. Cancer Res. 2021 Sep 1;81(17):4618-4627. doi: 10.1158/0008-5472.CAN-21-0836. Epub 2021 Jul 28. PMID: 34321244.

1676: Koelman L, Reichmann R, Börnhorst C, Schulze MB, Weikert C, Biemann R, Isermann B, Fritsche A, Aleksandrova K. Determinants of elevated chemerin as a novel biomarker of immunometabolism: data from a large population-based cohort. Endocr Connect. 2021 Sep 20;10(9):1200-1211. doi: 10.1530/EC-21-0273. PMID: 34431786; PMCID: PMC8494416.

1677: Jolliffe DA, Camargo CA Jr, Sluyter JD, Aglipay M, Aloia JF, Ganmaa D, Bergman P, Bischoff-Ferrari HA, Borzutzky A, Damsgaard CT, Dubnov-Raz G, Esposito S, Gilham C, Ginde AA, Golan-Tripto I, Goodall EC, Grant CC, Griffiths CJ, Hibbs AM, Janssens W, Khadilkar AV, Laaksi I, Lee MT, Loeb M, Maguire JL, Majak P, Mauger DT, Manaseki-Holland S, Murdoch DR, Nakashima A, Neale RE, Pham H, Rake C, Rees JR, Rosendahl J, Scragg R, Shah D, Shimizu Y, Simpson-Yap S, Trilok-Kumar G, Urashima M, Martineau AR. Vitamin D supplementation to prevent acute respiratory infections: a systematic review and meta-analysis of aggregate data from randomised controlled trials. Lancet Diabetes Endocrinol. 2021 May;9(5):276-292. doi: 10.1016/S2213-8587(21)00051-6. Epub 2021 Mar 30. PMID: 33798465.

1678: Cheung AK, Chang TI, Cushman WC, Furth SL, Hou FF, Ix JH, Knoll GA, Muntner P, Pecoits-Filho R, Sarnak MJ, Tobe SW, Tomson CRV, Lytvyn L, Craig JC, Tunnicliffe DJ, Howell M, Tonelli M, Cheung M, Earley A, Mann JFE. Executive summary of the KDIGO 2021 Clinical Practice Guideline for the Management of Blood Pressure in Chronic Kidney Disease. Kidney Int. 2021 Mar;99(3):559-569. doi: 10.1016/j.kint.2020.10.026. PMID: 33637203.

1679: Wu Y, Ma X, Fraser WD, Li M, Wang W, Huang H, Landry M, Hao Y, Liu H, Semenic S, Zhang Y, Wang H, Zhang J, Yu J, La X, Zhang C, Marc I, Jiang H. Caregivers' perceptions, challenges and service needs related to tackling childhood overweight and obesity: a qualitative study in three districts of Shanghai, China. BMC Public Health. 2021 Apr 21;21(1):768. doi: 10.1186/s12889-021-10744-6. PMID: 33882878; PMCID: PMC8061052.

1680: Webber BJ, Lang MA, Stuever DM, Escobar JD, Bylsma VFH, Wolff GG. Health-Related Behaviors and Odds of COVID-19 Hospitalization in a Military Population.

Prev Chronic Dis. 2021 Nov 11;18:E96. doi: 10.5888/pcd18.210222. PMID: 34762027; PMCID: PMC8588871.

1681: Gallè F, Veshi A, Sabella EA, Çitozi M, Da Molin G, Ferracuti S, Liguori G, Orsi GB, Napoli C, Napoli C. Awareness and Behaviors Regarding COVID-19 among Albanian Undergraduates. Behav Sci (Basel). 2021 Mar 31;11(4):45. doi: 10.3390/bs11040045. PMID: 33807250; PMCID: PMC8067270.

1682: Li M, Shao F, Qian Q, Yu W, Zhang Z, Chen B, Su D, Guo Y, Phan AV, Song LS, Stephens SB, Sebag J, Imai Y, Yang L, Cao H. A putative long noncoding RNA-encoded micropeptide maintains cellular homeostasis in pancreatic β cells. Mol Ther Nucleic Acids. 2021 Jul 16;26:307-320. doi: 10.1016/j.omtn.2021.06.027. PMID: 34513312; PMCID: PMC8416971.

1683: Hoofnagle JH, Bonkovsky HL, Phillips EJ, Li YJ, Ahmad J, Barnhart H, Durazo F, Fontana RJ, Gu J, Khan I, Kleiner DE, Koh C, Rockey DC, Seeff LB, Serrano J, Stolz A, Tillmann HL, Vuppalanchi R, Navarro VJ; Drug-Induced Liver Injury Network. HLA-B*35:01 and Green Tea-Induced Liver Injury. Hepatology. 2021 Jun;73(6):2484-2493. doi: 10.1002/hep.31538. Epub 2021 May 17. PMID: 32892374; PMCID: PMC8052949.

1684: Baysson H, Pennachio F, Wisniak A, Zabella ME, Pullen N, Collombet P, Lorthe E, Joost S, Balavoine JF, Bachmann D, Azman A, Pittet D, Chappuis F, Kherad O, Kaiser L, Guessous I, Stringhini S; Specchio-COVID19 study group. Specchio-COVID19 cohort study: a longitudinal follow-up of SARS-CoV-2 serosurvey participants in the canton of Geneva, Switzerland. BMJ Open. 2022 Jan 31;12(1):e055515. doi: 10.1136/bmjopen-2021-055515. PMID: 35105645; PMCID: PMC8804307.

1685: Hutton GB, Brugulat-Panés A, Bhagtani D, Mba Maadjhou C, Birch JM, Shih H, Okop K, Muti M, Wadende P, Tatah L, Mogo E, Guariguata L, Unwin N. A Systematic Scoping Review of the Impacts of Community Food Production Initiatives in Kenya, Cameroon, and South Africa. J Glob Health Rep. 2021 Mar 24;5:e2021010. doi: 10.29392/001c.19468. PMID: 33829114; PMCID: PMC7610539.

1686: Dobrzyńska M, Drzymała-Czyż S, Jakubowski K, Kurek S, Walkowiak J, Przysławski J. Copper and Zinc Content in Infant Milk Formulae Available on the Polish Market and Contribution to Dietary Intake. Nutrients. 2021 Jul 25;13(8):2542. doi: 10.3390/nu13082542. PMID: 34444702; PMCID: PMC8400833.

1687: Jørgensen HS, Eide IA, Jenssen T, Åsberg A, Bollerslev J, Godang K, Hartmann A, Schmidt EB, Svensson M. Marine n-3 Polyunsaturated Fatty Acids and Bone Mineral Density in Kidney Transplant Recipients: A Randomized, Placebo-Controlled Trial. Nutrients. 2021 Jul 10;13(7):2361. doi: 10.3390/nu13072361. PMID: 34371870; PMCID: PMC8308635.

1688: Herbert BM, Johnson AE, Paasche-Orlow MK, Brooks MM, Magnani JW. Disparities in Reporting a History of Cardiovascular Disease Among Adults With Limited English Proficiency and Angina. JAMA Netw Open. 2021 Dec

1;4(12):e2138780. doi: 10.1001/jamanetworkopen.2021.38780. PMID: 34905003; PMCID: PMC8672228.

1689: Djonor SK, Ako-Nnubeng IT, Owusu EA, Akuffo KO, Nortey P, Agyei-Manu E, Danso-Appiah A. Determinants of blood glucose control among people with Type 2 diabetes in a regional hospital in Ghana. PLoS One. 2021 Dec 22;16(12):e0261455. doi: 10.1371/journal.pone.0261455. PMID: 34936668; PMCID: PMC8694475.

1690: Djalalinia S, Hasani M, Asayesh H, Ejtahed HS, Malmir H, Kasaeian A, Zarei M, Baygi F, Rastad H, Mahdavi Gorabi A, Qorbani M. The effects of dietary selenium supplementation on inflammatory markers among patients with metabolic diseases: a systematic review and meta-analysis of randomized controlled trials. J Diabetes Metab Disord. 2021 Jun 9;20(1):1051-1062. doi: 10.1007/s40200-021-00821-3. PMID: 34222098; PMCID: PMC8212246.

1691: Huang MH, Wang YP, Wu PS, Chan YE, Cheng CM, Yang CH, Tsai SJ, Lu CL, Tsai CF. Association between gastrointestinal symptoms and depression among older adults in Taiwan: A cross-sectional study. J Chin Med Assoc. 2021 Mar 1;84(3):331-335. doi: 10.1097/JCMA.0000000000000460. Erratum in: J Chin Med Assoc. 2021 May 1;84(5):555. PMID: 33186213.

1692: Adamson D, Blazeby J, Porter C, Hurt C, Griffiths G, Nelson A, Sewell B, Jones M, Svobodova M, Fitzsimmons D, Nixon L, Fitzgibbon J, Thomas S, Millin A, Crosby T, Staffurth J, Byrne A. Palliative radiotherapy combined with stent insertion to reduce recurrent dysphagia in oesophageal cancer patients: the ROCS RCT. Health Technol Assess. 2021 May;25(31):1-144. doi: 10.3310/hta25310. PMID: 34042566; PMCID: PMC8182443.

1693: Guracho TT, Atomssa EM, Megersa OA, Tolossa T. Determinants of dental caries among adolescent patients attending Hospitals in West Wollega Zone, Western Ethiopia: A case-control study. PLoS One. 2021 Dec 2;16(12):e0260427. doi: 10.1371/journal.pone.0260427. PMID: 34855813; PMCID: PMC8639066.

1694: Zhou YF, Song XY, Pan XF, Feng L, Luo N, Yuan JM, Pan A, Koh WP. Association Between Combined Lifestyle Factors and Healthy Ageing in Chinese Adults: The Singapore Chinese Health Study. J Gerontol A Biol Sci Med Sci. 2021 Sep 13;76(10):1796-1805. doi: 10.1093/gerona/glab033. PMID: 33522576; PMCID: PMC8436980.

1695: Kim JA, Jang JH, Lee SY. An Updated Comprehensive Review on Vitamin A and Carotenoids in Breast Cancer: Mechanisms, Genetics, Assessment, Current Evidence, and Future Clinical Implications. Nutrients. 2021 Sep 10;13(9):3162. doi: 10.3390/nu13093162. PMID: 34579037; PMCID: PMC8465379.

1696: Popoviciu MS, Marin VN, Vesa CM, Stefan SD, Stoica RA, Serafinceanu C, Merlo EM, Rizvi AA, Rizzo M, Busnatu S, Stoian AP. Correlations between Diabetes Mellitus Self-Care Activities and Glycaemic Control in the Adult Population: A Cross-Sectional Study. Healthcare (Basel). 2022 Jan 17;10(1):174. doi: 10.3390/healthcare10010174. PMID: 35052337; PMCID: PMC8775516.

1697: Evans RG, Subasinghe AK, Busingye D, Srikanth VK, Kartik K, Kalyanram K, Suresh O, Arabshahi S, Curkpatrick I, O'Dea K, Walker KZ, Kaye M, Yang J, Thomas N, Arulappan G, Thrift AG. Renal and dietary factors associated with hypertension in a setting of disadvantage in rural India. J Hum Hypertens. 2021 Dec;35(12):1118-1128. doi: 10.1038/s41371-020-00473-5. Epub 2021 Jan 18. PMID: 33462389.

1698: Namkhah Z, Naeini F, Mahdi Rezayat S, Mehdi Yaseri, Mansouri S, Javad Hosseinzadeh-Attar M. Does naringenin supplementation improve lipid profile, severity of hepatic steatosis and probability of liver fibrosis in overweight/obese patients with NAFLD? A randomised, double-blind, placebo-controlled, clinical trial. Int J Clin Pract. 2021 Nov;75(11):e14852. doi: 10.1111/ijcp.14852. Epub 2021 Sep 18. PMID: 34516703.

1699: Ražnatović Đurović M, Janković J, Đurović M, Spirić J, Janković S. Adolescents' beliefs and perceptions of acne vulgaris: A cross-sectional study in Montenegrin schoolchildren. PLoS One. 2021 Jun 16;16(6):e0253421. doi: 10.1371/journal.pone.0253421. PMID: 34133464; PMCID: PMC8208562.

1700: Rahabi M, Salon M, Bruno-Bonnet C, Prat M, Jacquemin G, Benmoussa K, Alaeddine M, Parny M, Bernad J, Bertrand B, Auffret Y, Robert-Jolimaître P, Alric L, Authier H, Coste A. Bioactive fish collagen peptides weaken intestinal inflammation by orienting colonic macrophages phenotype through mannose receptor activation. Eur J Nutr. 2022 Jan 8. doi: 10.1007/s00394-021-02787-7. Epub ahead of print. PMID: 34999930.

1701: Mohammed A, He S. A Double-Blind, Randomized, Placebo-Controlled Trial to Evaluate the Efficacy of a Hydrolyzed Chicken Collagen Type II Supplement in Alleviating Joint Discomfort. Nutrients. 2021 Jul 18;13(7):2454. doi: 10.3390/nu13072454. PMID: 34371963; PMCID: PMC8308696.

1702: Seidel K, Wan X, Zhang M, Zhou Y, Zang M, Han J. Alcohol Binge Drinking Selectively Stimulates Protein S-Glutathionylation in Aorta and Liver of <i>ApoE</i>^{-/-} Mice. Front Cardiovasc Med. 2021 Mar 16;8:649813. doi: 10.3389/fcvm.2021.649813. PMID: 33796575; PMCID: PMC8007763.

1703: Chang HH, Chiang SY, Chen PC, Tsai CH, Yang RC, Tsai CL, Wu TH, Hsieh YW, Lin YC, Kuo YT, Chen KC, Chu HT. A system for reporting and evaluating adverse drug reactions of herbal medicine in Taiwan from 1998 to 2016. Sci Rep. 2021 Nov 2;11(1):21476. doi: 10.1038/s41598-021-00704-w. PMID: 34728662; PMCID: PMC8564513.

1704: Moradi M, Sohrabi G, Golbidi M, Yarmohammadi S, Hemati N, Campbell MS, Moradi S, Kermani MAH, Farzaei MH. Effects of artichoke on blood pressure: A systematic review and meta-analysis. Complement Ther Med. 2021 Mar;57:102668. doi: 10.1016/j.ctim.2021.102668. Epub 2021 Jan 16. PMID: 33465383.

1705: Perdomo CM, Gómez-Ambrosi J, Becerril S, Valentí V, Moncada R, Fernández-

Sáez EM, Méndez-Giménez L, Ezquerro S, Catalán V, Silva C, Escalada J, Frühbeck G, Rodríguez A. Role of ANGPTL8 in NAFLD Improvement after Bariatric Surgery in Experimental and Human Obesity. Int J Mol Sci. 2021 Nov 30;22(23):12945. doi: 10.3390/ijms222312945. PMID: 34884755; PMCID: PMC8657645.

1706: Zhen J, Stefanolo JP, Temprano MP, Seiler CL, Caminero A, de-Madaria E, Huguet MM, Santiago V, Niveloni SI, Smecuol EG, Dominguez LU, Trucco E, Lopez V, Olano C, Mansueto P, Carroccio A, Green PH, Duerksen D, Day AS, Tye-Din JA, Bai JC, Ciacci C, Verdú EF, Lebwohl B, Pinto-Sanchez MI. Risk perception and knowledge of COVID-19 in patients with celiac disease. World J Gastroenterol. 2021 Mar 28;27(12):1213-1225. doi: 10.3748/wjg.v27.i12.1213. PMID: 33828395; PMCID: PMC8006100.

1707: Kumah E, Afriyie EK, Abuosi AA, Ankomah SE, Fusheini A, Otchere G. Influence of the Model of Care on the Outcomes of Diabetes Self-Management Education Program: A Scoping Review. J Diabetes Res. 2021 Feb 19;2021:2969243. doi: 10.1155/2021/2969243. PMID: 33688505; PMCID: PMC7914106.

1708: Jafari SA, Rezaeian A, Namjou Z, Ghayour-Mobarhan M, Ghaneifar Z. Evaluation of nutritional adjustment program on quality of life in children with chronic liver disease. J Educ Health Promot. 2021 May 20;10:141. doi: 10.4103/jehp.jehp_855_20. PMID: 34222516; PMCID: PMC8224514.

1709: Riegel B, Westland H, Iovino P, Barelds I, Bruins Slot J, Stawnychy MA, Osokpo O, Tarbi E, Trappenburg JCA, Vellone E, Strömberg A, Jaarsma T. Characteristics of self-care interventions for patients with a chronic condition: A scoping review. Int J Nurs Stud. 2021 Apr;116:103713. doi: 10.1016/j.ijnurstu.2020.103713. Epub 2020 Jul 15. PMID: 32768137.

1710: Cromer SJ, Yu EW. Challenges and Opportunities for Osteoporosis Care During the COVID-19 Pandemic. J Clin Endocrinol Metab. 2021 Nov 19;106(12):e4795-e4808. doi: 10.1210/clinem/dgab570. PMID: 34343287; PMCID: PMC8385842.

1711: Stern JM, Burk RD, Asplin J, Krieger NS, Suadicani SO, Wang Y, Usyk M, Lee JA, Chen L, Becker J, Chan M, Bushinsky DA. Kidney stone formation and the gut microbiome are altered by antibiotics in genetic hypercalciuric stone-forming rats. Urolithiasis. 2021 Jun;49(3):185-193. doi: 10.1007/s00240-020-01223-5. Epub 2020 Nov 7. PMID: 33161469.

1712: Kachroo N, Lange D, Penniston KL, Stern J, Tasian G, Bajic P, Wolfe AJ, Suryavanshi M, Ticinesi A, Meschi T, Monga M, Miller AW. Meta-analysis of Clinical Microbiome Studies in Urolithiasis Reveal Age, Stone Composition, and Study Location as the Predominant Factors in Urolithiasis-Associated Microbiome Composition. mBio. 2021 Aug 31;12(4):e0200721. doi: 10.1128/mBio.02007-21. Epub 2021 Aug 10. PMID: 34372696; PMCID: PMC8406293.

1713: Okuyan B, Ozcan V, Balta E, Durak-Albayrak O, Turker M, Sancar M, Yavuz BB, Uner S, Ozcebe H. The impact of community pharmacists on older adults in

Turkey. J Am Pharm Assoc (2003). 2021 Nov-Dec;61(6):e83-e92. doi: 10.1016/j.japh.2021.06.009. Epub 2021 Jun 11. PMID: 34238671.

1714: Shen S, Luo M, Meng X, Deng Y, Cheng S. All-Cause Mortality Risk Associated With Solid Fuel Use Among Chinese Elderly People: A National Retrospective Longitudinal Study. Front Public Health. 2021 Oct 14;9:741637. doi: 10.3389/fpubh.2021.741637. PMID: 34722448; PMCID: PMC8551618.

1715: Zhang Y, Lu Y, Wang S, Yang L, Xia H, Sun G. Excessive Vitamin A Supplementation Increased the Incidence of Acute Respiratory Tract Infections: A Systematic Review and Meta-Analysis. Nutrients. 2021 Nov 26;13(12):4251. doi: 10.3390/nu13124251. PMID: 34959803; PMCID: PMC8706818.

1716: Lim S, Deaver JW, Rosa-Caldwell ME, Lee DE, Morena Da Silva F, Cabrera AR, Schrems ER, Saling LW, Washington TA, Fluckey JD, Greene NP. Muscle miR-16 deletion results in impaired insulin sensitivity and contractile function in a sex-dependent manner. Am J Physiol Endocrinol Metab. 2022 Jan 24. doi: 10.1152/ajpendo.00333.2021. Epub ahead of print. PMID: 35068192.

1717: Chang TY, Wu CH, Chang CY, Lee FJ, Wang BW, Doong JY, Lin YS, Kuo CS, Huang RS. Optimal Dietary Intake Composition of Choline and Betaine Is Associated with Minimized Visceral Obesity-Related Hepatic Steatosis in a Case-Control Study. Nutrients. 2022 Jan 8;14(2):261. doi: 10.3390/nu14020261. PMID: 35057441; PMCID: PMC8779168.

1718: Neumeier WH, Guerra N, Hsieh K, Thirumalai M, Ervin D, Rimmer JH. POWERSforID: Personalized online weight and exercise response system for individuals with intellectual disability: A randomized controlled trial. Disabil Health J. 2021 Oct;14(4):101111. doi: 10.1016/j.dhjo.2021.101111. Epub 2021 Apr 28. PMID: 33965364; PMCID: PMC8448903.

1719: Şahin-Bodur G, Keser A, Akçil-Ok M, Ünsal EN, Akın O. Children's Power of Food Scale: Turkish validity and reliability study. Public Health Nutr. 2021 Dec;24(17):5608-5615. doi: 10.1017/S1368980021003773. Epub 2021 Sep 2. PMID: 34472427; PMCID: PMC8609363.

1720: Bell KJ, Saad S, Tillett BJ, McGuire HM, Bordbar S, Yap YA, Nguyen LT, Wilkins MR, Corley S, Brodie S, Duong S, Wright CJ, Twigg S, de St Groth BF, Harrison LC, Mackay CR, Gurzov EN, Hamilton-Williams EE, Mariño E. Metabolite-based dietary supplementation in human type 1 diabetes is associated with microbiota and immune modulation. Microbiome. 2022 Jan 19;10(1):9. doi: 10.1186/s40168-021-01193-9. PMID: 35045871; PMCID: PMC8772108.

1721: Akagawa S, Akagawa Y, Nakai Y, Yamagishi M, Yamanouchi S, Kimata T, Chino K, Tamiya T, Hashiyada M, Akane A, Tsuji S, Kaneko K. Fiber-Rich Barley Increases Butyric Acid-Producing Bacteria in the Human Gut Microbiota. Metabolites. 2021 Aug 22;11(8):559. doi: 10.3390/metabo11080559. PMID: 34436500; PMCID: PMC8399161.

1722: Vlassopoulou M, Yannakoulia M, Pletsa V, Zervakis GI, Kyriacou A. Effects of fungal beta-glucans on health - a systematic review of randomized controlled trials. Food Funct. 2021 Apr 26;12(8):3366-3380. doi: 10.1039/d1fo00122a. PMID: 33876798.

1723: Nguyen PH, Kachwaha S, Pant A, Tran LM, Ghosh S, Sharma PK, Shastri VD, Escobar-Alegria J, Avula R, Menon P. Impact of COVID-19 on household food insecurity and interlinkages with child feeding practices and coping strategies in Uttar Pradesh, India: a longitudinal community-based study. BMJ Open. 2021 Apr 21;11(4):e048738. doi: 10.1136/bmjopen-2021-048738. PMID: 33883156; PMCID: PMC8061560.

1724: Mohajeri M, Horriatkhah E, Mohajery R. The effect of glutamine supplementation on serum levels of some inflammatory factors, oxidative stress, and appetite in COVID-19 patients: a case-control study. Inflammopharmacology. 2021 Dec;29(6):1769-1776. doi: 10.1007/s10787-021-00881-0. Epub 2021 Oct 28. Erratum in: Inflammopharmacology. 2021 Dec 20;: PMID: 34709541; PMCID: PMC8552429.

1725: Hsieh HF, Mistry R, Lee DB, Scott BA, Eisman AB, Heinze JE, Zimmerman MA. The Longitudinal Association Between Exposure to Violence and Patterns of Health Risk Behaviors Among African American Youth. Am J Health Promot. 2021 Jul;35(6):794-802. doi: 10.1177/0890117121995776. Epub 2021 Mar 4. PMID: 33657868.

1726: Palmer CR, Bellinge JW, Dalgaard F, Sim M, Murray K, Connolly E, Blekkenhorst LC, Bondonno CP, Croft KD, Gislason G, Tjønneland A, Overvad K, Schultz C, Lewis JR, Hodgson JM, Bondonno NP. Association between vitamin K₁ intake and mortality in the Danish Diet, Cancer, and Health cohort. Eur J Epidemiol. 2021 Oct;36(10):1005-1014. doi: 10.1007/s10654-021-00806-9. Epub 2021 Sep 30. PMID: 34591201; PMCID: PMC8542554.

1727: Xia Y, Luo Q, Chen J, Huang C, Jahangir A, Pan T, Wei X, Liu W, Chen Z. Retinal astrocytes and microglia activation in diabetic retinopathy rhesus monkey models. Curr Eye Res. 2021 Sep 21. doi: 10.1080/02713683.2021.1984535. Epub ahead of print. PMID: 34547966.

1728: Lazar RM, Howard VJ, Kernan WN, Aparicio HJ, Levine DA, Viera AJ, Jordan LC, Nyenhuis DL, Possin KL, Sorond FA, White CL; American Heart Association Stroke Council. A Primary Care Agenda for Brain Health: A Scientific Statement From the American Heart Association. Stroke. 2021 Jun;52(6):e295-e308. doi: 10.1161/STR.0000000000000367. Epub 2021 Mar 15. PMID: 33719523.

1729: Julian V, Ciba I, Olsson R, Dahlbom M, Furthner D, Gomahr J, Maruszczak K, Morwald K, Pixner T, Schneider A, Pereira B, Duclos M, Weghuber D, Thivel D, Bergsten P, Forslund A. Association between Metabolic Syndrome Diagnosis and the Physical Activity-Sedentary Profile of Adolescents with Obesity: A Complementary Analysis of the Beta-JUDO Study. Nutrients. 2021 Dec 24;14(1):60. doi: 10.3390/nu14010060. PMID: 35010936; PMCID: PMC8746544.

1730: Sina E, Buck C, Veidebaum T, Siani A, Reisch L, Pohlabeln H, Pala V, Moreno LA, Molnar D, Lissner L, Kourides Y, De Henauw S, Eiben G, Ahrens W, Hebestreit A; IDEFICS, I.Family consortia. Media use trajectories and risk of metabolic syndrome in European children and adolescents: the IDEFICS/I.Family cohort. Int J Behav Nutr Phys Act. 2021 Oct 18;18(1):134. doi: 10.1186/s12966-021-01186-9. PMID: 34663352; PMCID: PMC8521295.

1731: Matizirofa L, Chikobvu D. Analysing and quantifying the effect of predictors of stroke direct costs in South Africa using quantile regression. BMC Public Health. 2021 Aug 17;21(1):1560. doi: 10.1186/s12889-021-11592-0. PMID: 34404386; PMCID: PMC8369801.

1732: Simpson CA, Zhang JH, Vanderschueren D, Fu L, Pennestri TC, Bouillon R, Cole DEC, Carpenter TO. 25-OHD response to vitamin D supplementation in children: effect of dose but not GC haplotype. Eur J Endocrinol. 2021 Jul 7;185(2):333-342. doi: 10.1530/EJE-21-0349. PMID: 34128826; PMCID: PMC8284876.

1733: Luo Y, Zou P, Wang K, Li X, Wang J. Prevalence and Risk Factors of Urinary Incontinence Among Elderly Adults in Rural China: A Cross-Sectional Survey. J Wound Ostomy Continence Nurs. 2022 Jan-Feb 01;49(1):78-86. doi: 10.1097/WON.0000000000000829. PMID: 35040817.

1734: Shoeibi S, Mahdipour E, Mohammadi S, Moohebati M, Ghayour-Mobarhan M. Treatment of atherosclerosis through transplantation of endothelial progenitor cells overexpressing dimethylarginine dimethylaminohydrolase (DDAH) in rabbits. Int J Cardiol. 2021 May 15;331:189-198. doi: 10.1016/j.ijcard.2021.01.036. Epub 2021 Jan 31. PMID: 33535073.

1735: Stevens Y, Winkens B, Jonkers D, Masclee A. The effect of olive leaf extract on cardiovascular health markers: a randomized placebo-controlled clinical trial. Eur J Nutr. 2021 Jun;60(4):2111-2120. doi: 10.1007/s00394-020-02397-9. Epub 2020 Oct 9. PMID: 33034707; PMCID: PMC8137474.

1736: Thapa S, Venkatachalam A, Khan N, Naqvi M, Balderas M, Runge JK, Haag A, Hoch KM, Glaze DG, Luna RA, Motil KJ. Assessment of the gut bacterial microbiome and metabolome of girls and women with Rett Syndrome. PLoS One. 2021 May 6;16(5):e0251231. doi: 10.1371/journal.pone.0251231. PMID: 33956889; PMCID: PMC8101921.

1737: Faghfouri AH, Zarezadeh M, Aghapour B, Izadi A, Rostamkhani H, Majnouni A, Abu-Zaid A, Kord Varkaneh H, Ghoreishi Z, Ostadrahimi A. Clinical efficacy of zinc supplementation in improving antioxidant defense system: A comprehensive systematic review and time-response meta-analysis of controlled clinical trials. Eur J Pharmacol. 2021 Sep 15;907:174243. doi: 10.1016/j.ejphar.2021.174243. Epub 2021 Jun 6. PMID: 34102185.

1738: Jayawardena R, Swarnamali H, Ranasinghe P, Misra A. Health effects of coconut oil: Summary of evidence from systematic reviews and meta-analysis of

interventional studies. Diabetes Metab Syndr. 2021 Mar-Apr;15(2):549-555. doi: 10.1016/j.dsx.2021.02.032. Epub 2021 Mar 3. PMID: 33689936.

1739: Burton CS, Gonzalez G, Vaculik K, Khalil C, Zektser Y, Arnold C, Almario CV, Spiegel BMR, Anger JT. Female Lower Urinary Tract Symptom Prevention and Treatment Strategies on Social Media: Mixed Correlation With Evidence. Urology. 2021 Apr;150:139-145. doi: 10.1016/j.urology.2020.06.056. Epub 2020 Jul 13. PMID: 32673678.

1740: Morshedzadeh N, Rahimlou M, Shahrokh S, Karimi S, Mirmiran P, Zali MR. The effects of flaxseed supplementation on metabolic syndrome parameters, insulin resistance and inflammation in ulcerative colitis patients: An open-labeled randomized controlled trial. Phytother Res. 2021 Jul;35(7):3781-3791. doi: 10.1002/ptr.7081. Epub 2021 Apr 15. PMID: 33856729.

1741: Liu M, Nordstrom M, Forand S, Lewis-Michl E, Wattigney WA, Kannan K, Wang W, Irvin-Barnwell E, Hwang SA. Assessing exposures to per- and polyfluoroalkyl substances in two populations of Great Lakes Basin fish consumers in Western New York State. Int J Hyg Environ Health. 2022 Mar;240:113902. doi: 10.1016/j.ijheh.2021.113902. Epub 2021 Dec 13. PMID: 34915281.

1742: Binder EF, Christensen JC, Stevens-Lapsley J, Bartley J, Berry SD, Dobs AS, Fortinsky RH, Hildreth KL, Kiel DP, Kuchel GA, Marcus RL, McDonough CM, Orwig D, Sinacore DR, Schwartz RS, Volpi E, Magaziner J, Schechtman KB. A multi-center trial of exercise and testosterone therapy in women after hip fracture: Design, methods and impact of the COVID-19 pandemic. Contemp Clin Trials. 2021 May;104:106356. doi: 10.1016/j.cct.2021.106356. Epub 2021 Mar 11. PMID: 33716173.

1743: Neeland IJ, Marso SP, Ayers CR, Lewis B, Oslica R, Francis W, Rodder S, Pandey A, Joshi PH. Effects of liraglutide on visceral and ectopic fat in adults with overweight and obesity at high cardiovascular risk: a randomised, double-blind, placebo-controlled, clinical trial. Lancet Diabetes Endocrinol. 2021 Sep;9(9):595-605. doi: 10.1016/S2213-8587(21)00179-0. Epub 2021 Aug 3. PMID: 34358471.

1744: Lu Q, Guo P, Liu A, Ares I, Martínez-Larrañaga MR, Wang X, Anadón A, Martínez MA. The role of long noncoding RNA in lipid, cholesterol, and glucose metabolism and treatment of obesity syndrome. Med Res Rev. 2021 May;41(3):1751-1774. doi: 10.1002/med.21775. Epub 2020 Dec 24. PMID: 33368430.

1745: Li C, Luo F, Liu C, Xiong N, Xu Z, Zhang W, Yang M, Wang Y, Liu D, Yu C, Zeng J, Zhang L, Li D, Liu Y, Feng M, Liu R, Mei J, Deng S, Zeng Z, He Y, Liu H, Shi Z, Duan M, Kang D, Liao J, Li W, Liu L. Effect of a genetically engineered interferon-alpha versus traditional interferon-alpha in the treatment of moderate-to-severe COVID-19: a randomised clinical trial. Ann Med. 2021 Dec;53(1):391-401. doi: 10.1080/07853890.2021.1890329. PMID: 33620016; PMCID: PMC7906612.

1746: Mansour-Assi SJ, Golaszewski NM, Costello VL, Wing D, Persinger H, Coleman A, Lytle L, Larsen BA, Jain S, Weibel N, Rock CL, Patrick K, Hekler E, Godino JG. Social Mobile Approaches to Reducing Weight (SMART) 2.0: protocol of a randomized controlled trial among young adults in university settings. Trials. 2022 Jan 3;23(1):7. doi: 10.1186/s13063-021-05938-7. PMID: 34980208; PMCID: PMC8721474.

1747: Deftereos I, Yeung JMC, Arslan J, Carter VM, Isenring E, Kiss N, On Behalf Of The Nourish Point Prevalence Study Group. Assessment of Nutritional Status and Nutrition Impact Symptoms in Patients Undergoing Resection for Upper Gastrointestinal Cancer: Results from the Multi-Centre NOURISH Point Prevalence Study. Nutrients. 2021 Sep 24;13(10):3349. doi: 10.3390/nu13103349. PMID: 34684353; PMCID: PMC8539371.

1748: Rousseau-Ralliard D, Richard C, Hoarau P, Lallemand MS, Morillon L, Aubrière MC, Valentino SA, Dahirel M, Guinot M, Fournier N, Morin G, Mourier E, Camous S, Slama R, Cassee FR, Couturier-Tarrade A, Chavatte-Palmer P. Prenatal air pollution exposure to diesel exhaust induces cardiometabolic disorders in adulthood in a sex-specific manner. Environ Res. 2021 Sep;200:111690. doi: 10.1016/j.envres.2021.111690. Epub 2021 Jul 14. PMID: 34273365.

1749: Akbarpour E, Sadjadi A, Derakhshan MH, Roshandel G, Alimohammadian M. Gastric Cancer in Iran: An Overview of Risk Factors and Preventive Measures. Arch Iran Med. 2021 Jul 1;24(7):556-567. doi: 10.34172/aim.2021.79. PMID: 34488321.

1750: Ivey KL, Nguyen XT, Tobias DK, Song R, Rogers GB, Ho YL, Li R, Wilson PW, Cho K, Gaziano JM, Willett WC, Djoussé L. Dietary yogurt is distinct from other dairy foods in its association with circulating lipid profile: Findings from the Million Veteran Program. Clin Nutr ESPEN. 2021 Jun;43:456-463. doi: 10.1016/j.clnesp.2021.02.022. Epub 2021 Mar 20. PMID: 34024555.

1751: Felix CMM, Ghisi GLM, Seixas MB, Batalha APDB, Ezequiel DGA, Trevizan PF, Pereira DAG, Silva LPD. Translation, cross-cultural adaptation, and psychometric properties of the Brazilian Portuguese version of the DiAbeTes Education Questionnaire (DATE-Q). Braz J Phys Ther. 2021 Sep-Oct;25(5):583-592. doi: 10.1016/j.bjpt.2021.03.003. Epub 2021 Mar 26. PMID: 33824060; PMCID: PMC8536859.

1752: Mohammad A, Falahi E, Mohd Yusof BN, Hanipah ZN, Sabran MR, Mohamad Yusof L, Gheitasvand M. The effects of the ginger supplements on inflammatory parameters in type 2 diabetes patients: A systematic review and meta-analysis of randomised controlled trials. Clin Nutr ESPEN. 2021 Dec;46:66-72. doi: 10.1016/j.clnesp.2021.10.013. Epub 2021 Oct 22. PMID: 34857250.

1753: Shah M, Leong T, Freeman AJ. Order Set Use and Education Association With Pediatric Acute Pancreatitis Outcomes. Hosp Pediatr. 2021 Aug;11(8):885-892. doi: 10.1542/hpeds.2020-000323. PMID: 34315724.

1754: Yarizadeh H, Setayesh L, Majidi N, Rasaei N, Mehranfar S, Ebrahimi R,

Casazzza K, Mirzaei K. Nutrient patterns and their relation to obesity and metabolic syndrome in Iranian overweight and obese adult women. Eat Weight Disord. 2021 Jul 17. doi: 10.1007/s40519-021-01268-4. Epub ahead of print. PMID: 34273097.

1755: Rondanelli M, Riva A, Petrangolini G, Allegrini P, Giacosa A, Fazia T, Bernardinelli L, Gasparri C, Peroni G, Perna S. Berberine Phospholipid Is an Effective Insulin Sensitizer and Improves Metabolic and Hormonal Disorders in Women with Polycystic Ovary Syndrome: A One-Group Pretest-Post-Test Explanatory Study. Nutrients. 2021 Oct 19;13(10):3665. doi: 10.3390/nu13103665. PMID: 34684666; PMCID: PMC8538182.

1756: Pattem J, Davrandi M, Aguayo S, Slak B, Maev R, Allan E, Spratt D, Bozec L. Dependency of hydration and growth conditions on the mechanical properties of oral biofilms. Sci Rep. 2021 Aug 10;11(1):16234. doi: 10.1038/s41598-021-95701-4. PMID: 34376751; PMCID: PMC8355335.

1757: Abreu A, Keyes SK, Faries MD. Physician Assistant Students' Perceptions and Competencies Concerning Lifestyle Medicine. J Physician Assist Educ. 2021 Jun 1;32(2):97-101. doi: 10.1097/JPA.0000000000000355. PMID: 33966003.

1758: Wang MX, Gwee SXW, Pang J. Micronutrients Deficiency, Supplementation and Novel Coronavirus Infections-A Systematic Review and Meta-Analysis. Nutrients. 2021 May 10;13(5):1589. doi: 10.3390/nu13051589. PMID: 34068656; PMCID: PMC8151981.

1759: Severino M, Caruso S, Ferrazzano GF, Pisaneschi A, Fiasca F, Caruso S, De Giorgio S. Prevalence of Early Childhood Caries (ECC) in a paediatric italian population: An epidemiological study. Eur J Paediatr Dent. 2021 Sep;22(3):189-198. doi: 10.23804/ejpd.2021.22.03.3. PMID: 34544246.

1760: van Son J, Serlie MJ, Ståhlman M, Bäckhed F, Nieuwdorp M, Aron-Wisnewsky J. Plasma Imidazole Propionate Is Positively Correlated with Blood Pressure in Overweight and Obese Humans. Nutrients. 2021 Aug 6;13(8):2706. doi: 10.3390/nu13082706. PMID: 34444866; PMCID: PMC8399073.

1761: Gantumur G, Batsaikhan B, Huang CI, Yeh ML, Huang CF, Lin YH, Lin TC, Liang PC, Liu TW, Lee JJ, Lin YC, Lin IL, Huang JF, Chuang WL, Yu ML, Tu HP, Dai CY. The association between hepatitis C virus infection and renal function. J Chin Med Assoc. 2021 Aug 1;84(8):757-765. doi: 10.1097/JCMA.0000000000000561. PMID: 34074934.

1762: Xu X, Bao H, Tian Z, Zhu H, Zhu L, Niu L, Yan T, Dong H, Fang X, Zhang X. Prevalence, awareness, treatment, and control of hypertension in Northern China: a cross-sectional study. BMC Cardiovasc Disord. 2021 Nov 4;21(1):525. doi: 10.1186/s12872-021-02333-7. PMID: 34736420; PMCID: PMC8567672.

1763: Kirschner KM, Foryst-Ludwig A, Gohlke S, Li C, Flores RE, Kintscher U, Schupp M, Schulz TJ, Scholz H. Wt1 haploinsufficiency induces browning of

epididymal fat and alleviates metabolic dysfunction in mice on high-fat diet. Diabetologia. 2022 Mar;65(3):528-540. doi: 10.1007/s00125-021-05621-1. Epub 2021 Nov 30. PMID: 34846543; PMCID: PMC8803700.

1764: A MDH, Cifuentes L, Al-Ward R, Shah M, Murray JA, Mundi M. Post-bariatric Surgery Outcomes and Complications in Patients with Celiac Disease: a Matched Case-Control Study. Obes Surg. 2021 Oct;31(10):4405-4418. doi: 10.1007/s11695-021-05601-9. Epub 2021 Aug 5. PMID: 34350534.

1765: Rao KU, Henderson DI, Krishnan N, Puthia M, Glegola-Madejska I, Brive L, Bjarnemark F, Millqvist Fureby A, Hjort K, Andersson DI, Tenland E, Sturegård E, Robertson BD, Godaly G. A broad spectrum anti-bacterial peptide with an adjunct potential for tuberculosis chemotherapy. Sci Rep. 2021 Feb 18;11(1):4201. doi: 10.1038/s41598-021-83755-3. PMID: 33603037; PMCID: PMC7892554.

1766: Oh S, Tsujimoto T, Kim B, Uchida F, Suzuki H, Iizumi S, Isobe T, Sakae T, Tanaka K, Shoda J. Weight-loss-independent benefits of exercise on liver steatosis and stiffness in Japanese men with NAFLD. JHEP Rep. 2021 Feb 10;3(3):100253. doi: 10.1016/j.jhepr.2021.100253. PMID: 33898958; PMCID: PMC8059085.

1767: Toupchian O, Abdollahi S, Salehi-Abargouei A, Heshmati J, Clark CCT, Sheikhha MH, Fallahzadeh H, Mozaffari-Khosravi H. The effects of resveratrol supplementation on PPARα, p16, p53, p21 gene expressions, and sCD163/sTWEAK ratio in patients with type 2 diabetes mellitus: A double-blind controlled randomized trial. Phytother Res. 2021 Jun;35(6):3205-3213. doi: 10.1002/ptr.7031. Epub 2021 Feb 13. PMID: 33580595.

1768: Beale AL, O'Donnell JA, Nakai ME, Nanayakkara S, Vizi D, Carter K, Dean E, Ribeiro RV, Yiallourou S, Carrington MJ, Marques FZ, Kaye DM. The Gut Microbiome of Heart Failure With Preserved Ejection Fraction. J Am Heart Assoc. 2021 Jul 6;10(13):e020654. doi: 10.1161/JAHA.120.020654. Epub 2021 Jul 2. PMID: 34212778; PMCID: PMC8403331.

1769: Nadinskaia M, Maevskaya M, Ivashkin V, Kodzoeva K, Pirogova I, Chesnokov E, Nersesov A, Kaibullayeva J, Konysbekova A, Raissova A, Khamrabaeva F, Zueva E. Ursodeoxycholic acid as a means of preventing atherosclerosis, steatosis and liver fibrosis in patients with nonalcoholic fatty liver disease. World J Gastroenterol. 2021 Mar 14;27(10):959-975. doi: 10.3748/wjg.v27.i10.959. PMID: 33776366; PMCID: PMC7968130.

1770: Ayyildiz F, Yildiran H, Afandiyeva N, Gülbahar Ö, Köktürk O. The effects of vitamin D supplemantation on prognosis in patients with mild obstructive sleep apnea syndrome. Turk J Med Sci. 2021 Oct 21;51(5):2524-2533. doi: 10.3906/sag-2101-83. PMID: 34174792.

1771: Vahedian-Azimi A, Abbasifard M, Rahimi-Bashar F, Guest PC, Majeed M, Mohammadi A, Banach M, Jamialahmadi T, Sahebkar A. Effectiveness of Curcumin on Outcomes of Hospitalized COVID-19 Patients: A Systematic Review of Clinical

Trials. Nutrients. 2022 Jan 7;14(2):256. doi: 10.3390/nu14020256. PMID: 35057437; PMCID: PMC8779570.

1772: Parcha V, Patel N, Gutierrez OM, Li P, Gamble KL, Musunuru K, Margulies KB, Cappola TP, Wang TJ, Arora G, Arora P. Chronobiology of Natriuretic Peptides and Blood Pressure in Lean and Obese Individuals. J Am Coll Cardiol. 2021 May 11;77(18):2291-2303. doi: 10.1016/j.jacc.2021.03.291. PMID: 33958126; PMCID: PMC8138944.

1773: Kudinov VA, Torkhovskaya TI, Zakharova TS, Morozevich GE, Artyushev RI, Zubareva MY, Markin SS. High-density lipoprotein remodeling by phospholipid nanoparticles improves cholesterol efflux capacity and protects from atherosclerosis. Biomed Pharmacother. 2021 Sep;141:111900. doi: 10.1016/j.biopha.2021.111900. Epub 2021 Jul 13. PMID: 34328100.

1774: Marzullo P, Bettini S, Menafra D, Aprano S, Muscogiuri G, Barrea L, Savastano S, Colao A; Obesity Programs of nutrition, Education, Research and Assessment (OPERA) group. Spot-light on microbiota in obesity and cancer. Int J Obes (Lond). 2021 Nov;45(11):2291-2299. doi: 10.1038/s41366-021-00866-7. Epub 2021 Aug 6. PMID: 34363002.

1775: Hafez EN, Hamed WFAE. The Efficacy of Citrus maxima Peels Aqueous Extract Against Cryptosporidiosis in Immunecompromised Mice. Acta Parasitol. 2021 Jun;66(2):638-653. doi: 10.1007/s11686-020-00315-x. Epub 2021 Jan 20. PMID: 33471284.

1776: Babu AF, Csader S, Lok J, Gómez-Gallego C, Hanhineva K, El-Nezami H, Schwab U. Positive Effects of Exercise Intervention without Weight Loss and Dietary Changes in NAFLD-Related Clinical Parameters: A Systematic Review and Meta-Analysis. Nutrients. 2021 Sep 8;13(9):3135. doi: 10.3390/nu13093135. PMID: 34579012; PMCID: PMC8466505.

1777: Siljander H, Jason E, Ruohtula T, Selvenius J, Koivusaari K, Salonen M, Ahonen S, Honkanen J, Ilonen J, Vaarala O, Virtanen SM, Lähdeaho ML, Knip M. Effect of Early Feeding on Intestinal Permeability and Inflammation Markers in Infants with Genetic Susceptibility to Type 1 Diabetes: A Randomized Clinical Trial. J Pediatr. 2021 Nov;238:305-311.e3. doi: 10.1016/j.jpeds.2021.07.042. Epub 2021 Jul 20. PMID: 34293372.

1778: Hou Y, Ma R, Gao S, Kaudimba KK, Yan H, Liu T, Wang R. The Effect of Low and Moderate Exercise on Hyperuricemia: Protocol for a Randomized Controlled Study. Front Endocrinol (Lausanne). 2021 Sep 2;12:716802. doi: 10.3389/fendo.2021.716802. PMID: 34539569; PMCID: PMC8443794.

1779: Tajmim A, Cuevas-Ocampo AK, Siddique AB, Qusa MH, King JA, Abdelwahed KS, Sonju JJ, El Sayed KA. (-)-Oleocanthal Nutraceuticals for Alzheimer's Disease Amyloid Pathology: Novel Oral Formulations, Therapeutic, and Molecular Insights in 5xFAD Transgenic Mice Model. Nutrients. 2021 May 18;13(5):1702. doi: 10.3390/nu13051702. PMID: 34069842; PMCID: PMC8157389.

1780: Zhou M, Zhang N, Zhang Y, Yan X, Li M, Guo W, Guo X, He H, Guo K, Ma G. Effect of Mobile-Based Lifestyle Intervention on Weight Loss among the Overweight and Obese Elderly Population in China: A Randomized Controlled Trial. Int J Environ Res Public Health. 2021 Aug 21;18(16):8825. doi: 10.3390/ijerph18168825. PMID: 34444573; PMCID: PMC8393964.

1781: Ramos RJ, Mottin CC, Alves LB, Mulazzani CM, Padoin AV. Vitamin B12 supplementation orally and intramuscularly in people with obesity undergoing gastric bypass. Obes Res Clin Pract. 2021 Mar-Apr;15(2):177-179. doi: 10.1016/j.orcp.2021.02.002. Epub 2021 Feb 20. PMID: 33622624.

1782: Yang YL, Leu HB, Yin WH, Tseng WK, Wu YW, Lin TH, Yeh HI, Chang KC, Wang JH, Wu CC, Chen JW. Adherence to healthy lifestyle improved clinical outcomes in coronary artery disease patients after coronary intervention. J Chin Med Assoc. 2021 Jun 1;84(6):596-605. doi: 10.1097/JCMA.0000000000000536. PMID: 33871387.

1783: Kozlov Y, Poloyan S, Kapuller V. Laparoscopic Treatment of Type I of Pyloric Atresia: Case Report. J Laparoendosc Adv Surg Tech A. 2021 Dec;31(12):1507-1510. doi: 10.1089/lap.2021.0254. Epub 2021 Nov 25. PMID: 34846943.

1784: Allanach JR, Farrell JW 3rd, Mésidor M, Karimi-Abdolrezaee S. Current status of neuroprotective and neuroregenerative strategies in multiple sclerosis: A systematic review. Mult Scler. 2022 Jan;28(1):29-48. doi: 10.1177/13524585211008760. Epub 2021 Apr 19. PMID: 33870797; PMCID: PMC8688986.

1785: Singh S, Sk MF, Sonawane A, Kar P, Sadhukhan S. Plant-derived natural polyphenols as potential antiviral drugs against SARS-CoV-2 <i>via</i> RNA-dependent RNA polymerase (RdRp) inhibition: an <i>in-silico</i> analysis. J Biomol Struct Dyn. 2021 Oct;39(16):6249-6264. doi: 10.1080/07391102.2020.1796810. Epub 2020 Jul 28. PMID: 32720577; PMCID: PMC7441777.

1786: Lee-Bravatti MA, O'Neill HJ, Wurth RC, Sotos-Prieto M, Gao X, Falcon LM, Tucker KL, Mattei J. Lifestyle Behavioral Factors and Integrative Successful Aging Among Puerto Ricans Living in the Mainland United States. J Gerontol A Biol Sci Med Sci. 2021 May 22;76(6):1108-1116. doi: 10.1093/gerona/glaa259. PMID: 33045072; PMCID: PMC8248899.

1787: Wei J, Liu S, Cheng Y, Yang W, Zhu Z, Zeng L. Association of Infant Physical Development and Rapid Growth With Pubertal Onset Among Girls in Rural China. JAMA Netw Open. 2021 May 3;4(5):e216831. doi: 10.1001/jamanetworkopen.2021.6831. PMID: 33938939; PMCID: PMC8094009.

1788: Patten EV, Spruance L, Vaterlaus JM, Jones M, Beckstead E. Disaster Management and School Nutrition: A Qualitative Study of Emergency Feeding During the COVID-19 Pandemic. J Acad Nutr Diet. 2021 Aug;121(8):1441-1453. doi: 10.1016/j.jand.2021.04.012. Epub 2021 Apr 17. PMID: 33994141.

1789: Alghadir AH, Iqbal ZA, A Gabr S. The Relationships of Watching Television, Computer Use, Physical Activity, and Food Preferences to Body Mass Index: Gender and Nativity Differences among Adolescents in Saudi Arabia. Int J Environ Res Public Health. 2021 Sep 21;18(18):9915. doi: 10.3390/ijerph18189915. PMID: 34574844; PMCID: PMC8469276.

1790: Yang B, Yue Y, Chen Y, Ding M, Li B, Wang L, Wang Q, Stanton C, Ross RP, Zhao J, Zhang H, Chen W. <i>Lactobacillus plantarum</i> CCFM1143 Alleviates Chronic Diarrhea <i>via</i> Inflammation Regulation and Gut Microbiota Modulation: A Double-Blind, Randomized, Placebo-Controlled Study. Front Immunol. 2021 Oct 15;12:746585. doi: 10.3389/fimmu.2021.746585. PMID: 34721416; PMCID: PMC8555466.

1791: Baxter JB, Wasan Y, Hussain A, Soofi SB, Ahmed I, Bhutta ZA. Characterizing Micronutrient Status and Risk Factors among Late Adolescent and Young Women in Rural Pakistan: A Cross-Sectional Assessment of the MaPPS Trial. Nutrients. 2021 Apr 9;13(4):1237. doi: 10.3390/nu13041237. PMID: 33918630; PMCID: PMC8069550.

1792: Ramirez-Perez FI, Cabral-Amador FJ, Whaley-Connell AT, Aroor AR, Morales-Quinones M, Woodford ML, Ghiarone T, Ferreira-Santos L, Jurrissen TJ, Manrique-Acevedo CM, Jia G, DeMarco VG, Padilla J, Martinez-Lemus LA, Lastra G. Cystamine reduces vascular stiffness in Western diet-fed female mice. Am J Physiol Heart Circ Physiol. 2022 Feb 1;322(2):H167-H180. doi: 10.1152/ajpheart.00431.2021. Epub 2021 Dec 10. PMID: 34890280; PMCID: PMC8742720.

1793: Alshahrani SM, Alghannam AF, Taha N, Alqahtani SS, Al-Mutairi A, Al-Saud N, Alghnam S. The Impact of COVID-19 Pandemic on Weight and Body Mass Index in Saudi Arabia: A Longitudinal Study. Front Public Health. 2022 Jan 17;9:775022. doi: 10.3389/fpubh.2021.775022. PMID: 35111714; PMCID: PMC8801912.

1794: Vasconcelos CEGDC, Cabral MMLGF, Ramos ECP, Mendes RDC. Benefits of adding food education sessions to an exercise programme on cardiovascular risk factors in patients with type 2 diabetes. J Nutr Sci. 2021 Aug 11;10:e59. doi: 10.1017/jns.2021.50. PMID: 34422261; PMCID: PMC8358841.

1795: Mathur P, Kulothungan V, Leburu S, Krishnan A, Chaturvedi HK, Salve HR, Amarchand R, Nongkynrih B, Ganeshkumar P, Urs K S V, Laxmaiah A, Boruah M, Kumar S, Patro BK, Raghav PR, Rajkumar P, Sarma PS, Sharma R, Tambe M, Arlappa N, Mahanta TG, Bhuyan PJ, Joshi RP, Pakhare A, Galhotra A, Kumar D, Behera BK, Topno RK, Gupta MK, Rustagi N, Trivedi AV, Thankappan KR, Gupta S, Garg S, Shelke SC; ICMR-NNMS investigator group. Baseline risk factor prevalence among adolescents aged 15-17 years old: findings from National Non-communicable Disease Monitoring Survey (NNMS) of India. BMJ Open. 2021 Jun 29;11(6):e044066. doi: 10.1136/bmjopen-2020-044066. PMID: 34187814; PMCID: PMC8245441.

1796: Amerikanou C, Kanoni S, Kaliora AC, Barone A, Bjelan M, D'Auria G, Gioxari A, Gosalbes MJ, Mouchti S, Stathopoulou MG, Soriano B, Stojanoski S, Banerjee R,

Halabalaki M, Mikropoulou EV, Kannt A, Lamont J, Llorens C, Marascio F, Marascio M, Roig FJ, Smyrnioudis I, Varlamis I, Visvikis-Siest S, Vukic M, Milic N, Medic-Stojanoska M, Cesarini L, Campolo J, Gastaldelli A, Deloukas P, Trivella MG, Francino MP, Dedoussis GV; MAST4HEALTH consortium. Effect of Mastiha supplementation on NAFLD: The MAST4HEALTH Randomised, Controlled Trial. Mol Nutr Food Res. 2021 May;65(10):e2001178. doi: 10.1002/mnfr.202001178. Epub 2021 Apr 16. PMID: 33629536.

1797: van Bilsen JHM, van den Brink W, van den Hoek AM, Dulos R, Caspers MPM, Kleemann R, Wopereis S, Verschuren L. Mechanism-Based Biomarker Prediction for Low-Grade Inflammation in Liver and Adipose Tissue. Front Physiol. 2021 Nov 10;12:703370. doi: 10.3389/fphys.2021.703370. PMID: 34858196; PMCID: PMC8631400.

1798: Lewandowska M. Gestational Diabetes Mellitus (GDM) Risk for Declared Family History of Diabetes, in Combination with BMI Categories. Int J Environ Res Public Health. 2021 Jun 28;18(13):6936. doi: 10.3390/ijerph18136936. PMID: 34203509; PMCID: PMC8293805.

1799: Mahurin HM, Tarabadkar E, Hippe DS, Lachance K, Kim EJ, Loggers ET, Shinohara MM. Integrative medicine use in patients with cutaneous T-Cell lymphoma: A cross-sectional survey study. Complement Ther Med. 2021 Sep;61:102762. doi: 10.1016/j.ctim.2021.102762. Epub 2021 Jul 21. PMID: 34302983.

1800: Gül-Klein S, Haxhiraj D, Seelig J, Kästner A, Hackler J, Sun Q, Heller RA, Lachmann N, Pratschke J, Schmelzle M, Schomburg L. Serum Selenium Status as a Diagnostic Marker for the Prognosis of Liver Transplantation. Nutrients. 2021 Feb 14;13(2):619. doi: 10.3390/nu13020619. PMID: 33672988; PMCID: PMC7918136.

1801: Baldwin C, de van der Schueren MA, Kruizenga HM, Weekes CE. Dietary advice with or without oral nutritional supplements for disease-related malnutrition in adults. Cochrane Database Syst Rev. 2021 Dec 21;12(12):CD002008. doi: 10.1002/14651858.CD002008.pub5. PMID: 34931696; PMCID: PMC8691169.

1802: Ponticelli C, Favi E, Ferraresso M. New-Onset Diabetes after Kidney Transplantation. Medicina (Kaunas). 2021 Mar 8;57(3):250. doi: 10.3390/medicina57030250. PMID: 33800138; PMCID: PMC7998982.

1803: Cirillo M, Cavallo P, Zulli E, Villa R, Veneziano R, Costanzo S, Magnacca S, Di Castelnuovo A, Iacoviello L, On Behalf Of Moli-Sani Study Investigators. Sodium Intake and Proteinuria/Albuminuria in the Population-Observational, Cross-Sectional Study. Nutrients. 2021 Apr 11;13(4):1255. doi: 10.3390/nu13041255. PMID: 33920400; PMCID: PMC8068813.

1804: Damani JJ, De Souza MJ, VanEvery HL, Strock NCA, Rogers CJ. The Role of Prunes in Modulating Inflammatory Pathways to Improve Bone Health in Postmenopausal Women. Adv Nutr. 2022 Jan 3:nmab162. doi: 10.1093/advances/nmab162. Epub ahead of print. PMID: 34978320.

1805: Hart CN, Hawley NL, Coffman DL, Raynor HA, Carskadon MA, Jelalian E, Owens JA, Spaeth A, Wing RR. Randomized controlled trial to enhance children's sleep, eating, and weight. Pediatr Res. 2021 Dec 20. doi: 10.1038/s41390-021-01870-3. Epub ahead of print. PMID: 34930967.

1806: Mukherjee S, Chakraborty M, Ulmasov B, McCommis K, Zhang J, Carpenter D, Msengi EN, Haubner J, Guo C, Pike DP, Ghoshal S, Ford DA, Neuschwander-Tetri BA, Chakraborty A. Pleiotropic actions of IP6K1 mediate hepatic metabolic dysfunction to promote nonalcoholic fatty liver disease and steatohepatitis. Mol Metab. 2021 Dec;54:101364. doi: 10.1016/j.molmet.2021.101364. Epub 2021 Oct 28. PMID: 34757046; PMCID: PMC8609165.

1807: Kanoni S, Kumar S, Amerikanou C, Kurth MJ, Stathopoulou MG, Bourgeois S, Masson C, Kannt A, Cesarini L, Kontoe MS, Milanović M, Roig FJ, Beribaka M, Campolo J, Jiménez-Hernández N, Milošević N, Llorens C, Smyrnioudis I, Francino MP, Milić N, Kaliora AC, Trivella MG, Ruddock MW, Medić-Stojanoska M, Gastaldelli A, Lamont J, Deloukas P, Dedoussis GV, Visvikis-Siest S. Nutrigenetic Interactions Might Modulate the Antioxidant and Anti-Inflammatory Status in Mastiha-Supplemented Patients With NAFLD. Front Immunol. 2021 May 7;12:683028. doi: 10.3389/fimmu.2021.683028. PMID: 34025683; PMCID: PMC8138178.

1808: Xing Y, Zhao B, Yin L, Guo M, Shi H, Zhu Z, Zhang L, He J, Ling Y, Gao M, Lu H, Mao E, Zhang L. Vitamin C supplementation is necessary for patients with coronavirus disease: An ultra-high-performance liquid chromatography-tandem mass spectrometry finding. J Pharm Biomed Anal. 2021 Mar 20;196:113927. doi: 10.1016/j.jpba.2021.113927. Epub 2021 Jan 27. PMID: 33549875; PMCID: PMC7839397.

1809: Feng KM, Chien WC, Chen JT, Chen YH, Chung CH, Sun CA, Chen CL. The impact of glucosamine on age-related macular degeneration in patients: A nationwide, population-based cohort study. PLoS One. 2021 May 19;16(5):e0251925. doi: 10.1371/journal.pone.0251925. PMID: 34010361; PMCID: PMC8133402.

1810: Lin S, Zhang Y, Jiang L, Li J, Chai J, Pei L, Shang X. Interactive Effects of Maternal Vitamin D Status and Socio-Economic Status on the Risk of Spontaneous Abortion: Evidence from Henan Province, China. Nutrients. 2022 Jan 11;14(2):291. doi: 10.3390/nu14020291. PMID: 35057472; PMCID: PMC8780117.

1811: Rossato MS, Brilli E, Ferri N, Giordano G, Tarantino G. Observational study on the benefit of a nutritional supplement, supporting immune function and energy metabolism, on chronic fatigue associated with the SARS-CoV-2 post-infection progress. Clin Nutr ESPEN. 2021 Dec;46:510-518. doi: 10.1016/j.clnesp.2021.08.031. Epub 2021 Sep 6. PMID: 34857243; PMCID: PMC8420132.

1812: Yuan J, Jiang X, Zhu T, Zhang Y, Wang Y, Yang X, Shang L. Caregivers' feeding behaviour, children's eating behaviour and weight status among children of preschool age in China. J Hum Nutr Diet. 2021 Oct;34(5):807-818. doi: 10.1111/jhn.12869. Epub 2021 Feb 27. PMID: 33639028; PMCID: PMC8518084.

1813: Gendler Y, Seguier-Lipszyc E, Silbermintz A, Hain M, Stern Y, Kravarusic D, Politi K, Amir G, Katz J, Zeitlin Y, Grozovski S, Nitzan Y, Eshel Y, Shimoni A, Fischer Y, Serfaty D, Shnayderman T, Assi K, Barbash L, Stafler P. Aerodigestive Clinics as Emerging Pediatric Care Model: The First 100 Patients in Israel. Isr Med Assoc J. 2021 Sep;23(9):569-575. PMID: 34472232.

1814: Hadrup N, Frederiksen M, Wedebye EB, Nikolov NG, Carøe TK, Sørli JB, Frydendall KB, Liguori B, Sejbaek CS, Wolkoff P, Flachs EM, Schlünssen V, Meyer HW, Clausen PA, Hougaard KS. Asthma-inducing potential of 28 substances in spray cleaning products-Assessed by quantitative structure activity relationship (QSAR) testing and literature review. J Appl Toxicol. 2022 Jan;42(1):130-153. doi: 10.1002/jat.4215. Epub 2021 Jul 11. PMID: 34247391.

1815: Cooper TE, Dalton A, Kieu A, Howell M, Jayanti S, Khalid R, Lim WH, Scholes-Robertson N, Craig JC, Teixeira-Pinto A, Bourke MJ, Tong A, Wong G. The CKD bowel health study: understanding the bowel health and gastrointestinal symptom management in patients with chronic kidney disease: a mixed-methods observational longitudinal study (protocol). BMC Nephrol. 2021 Nov 21;22(1):388. doi: 10.1186/s12882-021-02600-x. PMID: 34802445; PMCID: PMC8606224.

1816: Dat NQ, Thuy LTT, Hieu VN, Hai H, Hoang DV, Thi Thanh Hai N, Thuy TTV, Komiya T, Rombouts K, Dong MP, Hanh NV, Hoang TH, Sato-Matsubara M, Daikoku A, Kadono C, Oikawa D, Yoshizato K, Tokunaga F, Pinzani M, Kawada N. Hexa Histidine-Tagged Recombinant Human Cytoglobin Deactivates Hepatic Stellate Cells and Inhibits Liver Fibrosis by Scavenging Reactive Oxygen Species. Hepatology. 2021 Jun;73(6):2527-2545. doi: 10.1002/hep.31752. Epub 2021 May 22. PMID: 33576020; PMCID: PMC8251927.

1817: Katz L, Tata A, Woolman M, Zarrine-Afsar A. Lipid Profiling in Cancer Diagnosis with Hand-Held Ambient Mass Spectrometry Probes: Addressing the Late-Stage Performance Concerns. Metabolites. 2021 Sep 28;11(10):660. doi: 10.3390/metabo11100660. PMID: 34677375; PMCID: PMC8537725.

1818: Schultz NM, Bhardwaj S, Barclay C, Gaspar L, Schwartz J. Global Burden of Dry Age-Related Macular Degeneration: A Targeted Literature Review. Clin Ther. 2021 Oct;43(10):1792-1818. doi: 10.1016/j.clinthera.2021.08.011. Epub 2021 Sep 20. PMID: 34548176.

1819: Maghsoumi-Norouzabad L, Zare Javid A, Mansoori A, Dadfar M, Serajian A. The effects of Vitamin D3 supplementation on Spermatogram and endocrine factors in asthenozoospermia infertile men: a randomized, triple blind, placebo-controlled clinical trial. Reprod Biol Endocrinol. 2021 Jul 5;19(1):102. doi: 10.1186/s12958-021-00789-y. PMID: 34225767; PMCID: PMC8256550.

1820: Figge A, Sydor S, Wenning C, Manka P, Assmuth S, Vilchez-Vargas R, Link A, Jähnert A, Brodesser S, Lucas C, Nevzorova YA, Faber KN, Moshage H, Porsch-Özcürümez M, Gerken G, Cubero FJ, Canbay A, Bechmann LP. Gender and gut microbiota composition determine hepatic bile acid, metabolic and inflammatory response to a single fast-food meal in healthy adults. Clin Nutr. 2021

May;40(5):2609-2619. doi: 10.1016/j.clnu.2021.04.008. Epub 2021 Apr 20. PMID: 33933727.

1821: Azul AM, Almendra R, Quatorze M, Loureiro A, Reis F, Tavares R, Mota-Pinto A, Cunha A, Rama L, Malva JO, Santana P, Ramalho-Santos J; HeaLIQs4Cities consortium. Unhealthy lifestyles, environment, well-being and health capability in rural neighbourhoods: a community-based cross-sectional study. BMC Public Health. 2021 Sep 6;21(1):1628. doi: 10.1186/s12889-021-11661-4. PMID: 34488709; PMCID: PMC8422758.

1822: Amini L, Chekini R, Nateghi MR, Haghani H, Jamialahmadi T, Sathyapalan T, Sahebkar A. The Effect of Combined Vitamin C and Vitamin E Supplementation on Oxidative Stress Markers in Women with Endometriosis: A Randomized, Triple-Blind Placebo-Controlled Clinical Trial. Pain Res Manag. 2021 May 26;2021:5529741. doi: 10.1155/2021/5529741. PMID: 34122682; PMCID: PMC8172324.

1823: Abdulle AE, Arends S, van Goor H, Brouwer E, van Roon AM, Westra J, Herrick AL, de Leeuw K, Mulder DJ. Low body weight and involuntary weight loss are associated with Raynaud's phenomenon in both men and women. Scand J Rheumatol. 2021 Mar;50(2):153-160. doi: 10.1080/03009742.2020.1780310. Epub 2020 Oct 16. PMID: 33063580.

1824: Juarez D, Fruman DA. Targeting the Mevalonate Pathway in Cancer. Trends Cancer. 2021 Jun;7(6):525-540. doi: 10.1016/j.trecan.2020.11.008. Epub 2021 Jan 6. PMID: 33358111; PMCID: PMC8137523.

1825: Durão C, Vaz C, de Oliveira VN, Calhau C. Confinement During the COVID-19 Pandemic After Metabolic and Bariatric Surgery-Associations Between Emotional Distress, Energy-Dense Foods, and Body Mass Index. Obes Surg. 2021 Oct;31(10):4452-4460. doi: 10.1007/s11695-021-05608-2. Epub 2021 Aug 6. PMID: 34357533; PMCID: PMC8343354.

1826: Lim SY, Wang R, Tan DJH, Ng CH, Lim WH, Quek J, Syn N, Nah BKY, Wong ET, Huang DQ, Vathsala A, Siddiqui MS, Fung J, Muthiah MD, Tan EX. A meta-analysis of the cumulative incidence, risk factors, and clinical outcomes associated with chronic kidney disease after liver transplantation. Transpl Int. 2021 Dec;34(12):2524-2533. doi: 10.1111/tri.14149. Epub 2021 Nov 15. PMID: 34714569.

1827: Gustin K, Barman M, Skröder H, Jacobsson B, Sandin A, Sandberg AS, Wold AE, Vahter M, Kippler M. Thyroid hormones in relation to toxic metal exposure in pregnancy, and potential interactions with iodine and selenium. Environ Int. 2021 Dec;157:106869. doi: 10.1016/j.envint.2021.106869. Epub 2021 Sep 13. PMID: 34530290.

1828: Lin M, Heizati M, Wang L, Nurula M, Yang Z, Wang Z, Abudoyreyimu R, Wu Z, Li N. A systematic review and meta-analysis of effects of spironolactone on blood pressure, glucose, lipids, renal function, fibrosis and inflammation in patients with hypertension and diabetes. Blood Press. 2021 Jun;30(3):145-153. doi: 10.1080/08037051.2021.1880881. Epub 2021 Mar 8. PMID: 33682538.

1829: Van Olden CC, Van de Laar AW, Meijnikman AS, Aydin O, Van Olst N, Hoozemans JB, De Brauw LM, Bruin SC, Acherman YIZ, Verheij J, Pyykkö JE, Hagedoorn M, Sanderman R, Bosma NC, Tremaroli V, Lundqvist A, Olofsson LE, Herrema H, Lappa D, Hjorth S, Nielsen J, Schwartz T, Groen AK, Nieuwdorp M, Bäckhed F, Gerdes VEA. A systems biology approach to understand gut microbiota and host metabolism in morbid obesity: design of the BARIA Longitudinal Cohort Study. J Intern Med. 2021 Mar;289(3):340-354. doi: 10.1111/joim.13157. Epub 2020 Sep 29. PMID: 32640105; PMCID: PMC7984244.

1830: Jiang K, Slee A, Davenport A. Body composition and weakness of hand grip strength and pinch strength in patients with chronic kidney disease from different ethnic backgrounds. J Hum Nutr Diet. 2021 Apr;34(2):450-455. doi: 10.1111/jhn.12825. Epub 2020 Oct 21. PMID: 33085839.

1831: Makarem N, Sears DD, St-Onge MP, Zuraikat FM, Gallo LC, Talavera GA, Castaneda SF, Lai Y, Aggarwal B. Variability in Daily Eating Patterns and Eating Jetlag Are Associated With Worsened Cardiometabolic Risk Profiles in the American Heart Association Go Red for Women Strategically Focused Research Network. J Am Heart Assoc. 2021 Sep 21;10(18):e022024. doi: 10.1161/JAHA.121.022024. Epub 2021 Sep 6. PMID: 34482703; PMCID: PMC8649529.

1832: Nieto A, Zhang L, Bhandari D, Zhu W, Blount BC, De Jesús VR. Exposure to 1,3-Butadiene in the U.S. Population: National Health and Nutrition Examination Survey 2011-2016. Biomarkers. 2021 Jun;26(4):371-383. doi: 10.1080/1354750X.2021.1904000. Epub 2021 Apr 8. PMID: 33729088.

1833: Asbaghi O, Moradi S, Nezamoleslami S, Moosavian SP, Hojjati Kermani MA, Lazaridi AV, Miraghajani M. The Effects of Magnesium Supplementation on Lipid Profile Among Type 2 Diabetes Patients: a Systematic Review and Meta-analysis of Randomized Controlled Trials. Biol Trace Elem Res. 2021 Mar;199(3):861-873. doi: 10.1007/s12011-020-02209-5. Epub 2020 May 28. PMID: 32468224.

1834: Leng J, McNally S, Walton G, Swann J, Proudman C, Argo C, Emery S, La Ragione R, Eustace R. Hay vs haylage: Forage type influences the equine urinary metabonome and faecal microbiota. Equine Vet J. 2021 Apr 26. doi: 10.1111/evj.13456. Epub ahead of print. PMID: 33900659.

1835: Komolafe O, Buzzetti E, Linden A, Best LM, Madden AM, Roberts D, Chase TJ, Fritche D, Freeman SC, Cooper NJ, Sutton AJ, Milne EJ, Wright K, Pavlov CS, Davidson BR, Tsochatzis E, Gurusamy KS. Nutritional supplementation for nonalcohol-related fatty liver disease: a network meta-analysis. Cochrane Database Syst Rev. 2021 Jul 19;7(7):CD013157. doi: 10.1002/14651858.CD013157.pub2. PMID: 34280304; PMCID: PMC8406904.

1836: Hernández-Alonso P, Canudas S, Boughanem H, Toledo E, Sorlí JV, Estruch R, Castañer O, Lapetra J, Alonso-Gómez AM, Gutiérrez-Bedmar M, Fiol M, Serra-Majem L, Pintó X, Ros E, Fernandez-Lazaro CI, Ramirez-Sabio JB, Fitó M, Portu-Zapirain J, Macias-González M, Babio N, Salas-Salvadó J. Dietary vitamin D intake and

colorectal cancer risk: a longitudinal approach within the PREDIMED study. Eur J Nutr. 2021 Dec;60(8):4367-4378. doi: 10.1007/s00394-021-02585-1. Epub 2021 May 28. PMID: 34050394.

1837: Bagheri R, Negaresh R, Motevalli MS, Wong A, Ashtary-Larky D, Kargarfard M, Rashidlamir A. Spirulina supplementation during gradual weight loss in competitive wrestlers. Br J Nutr. 2022 Jan 28;127(2):248-256. doi: 10.1017/S000711452100091X. Epub 2021 Mar 15. PMID: 33715648.

1838: Gabryszewski SJ, Dudley J, Grundmeier RW, Hill DA. Early-life environmental exposures associate with individual and cumulative allergic morbidity. Pediatr Allergy Immunol. 2021 Jul;32(5):1089-1093. doi: 10.1111/pai.13486. Epub 2021 Mar 8. PMID: 33616233; PMCID: PMC8249342.

1839: Miao J, Bachmann KN, Huang S, Su YR, Dusek J, Newton-Cheh C, Arora P, Wang TJ. Effects of Vitamin D Supplementation on Cardiovascular and Glycemic Biomarkers. J Am Heart Assoc. 2021 May 18;10(10):e017727. doi: 10.1161/JAHA.120.017727. Epub 2021 May 7. PMID: 33960201; PMCID: PMC8200713.

1840: Beigmohammadi MT, Bitarafan S, Hoseindokht A, Abdollahi A, Amoozadeh L, Soltani D. The effect of supplementation with vitamins A, B, C, D, and E on disease severity and inflammatory responses in patients with COVID-19: a randomized clinical trial. Trials. 2021 Nov 14;22(1):802. doi: 10.1186/s13063-021-05795-4. PMID: 34776002; PMCID: PMC8590866.

1841: Salvado R, Santos-Minguez S, Agudo-Conde C, Lugones-Sanchez C, Cabo-Laso A, Mª Hernandez-Sanchez J, Benito R, Rodriguez-Sanchez E, Gomez-Marcos MA, Hernandez-Rivas JM, Guimarães Cunha P, Garcia-Ortiz L, Investigators M. Gut microbiota composition and arterial stiffness measured by pulse wave velocity: case-control study protocol (MIVAS study). BMJ Open. 2021 Feb 11;11(2):e038933. doi: 10.1136/bmjopen-2020-038933. PMID: 33574140; PMCID: PMC7880115.

1842: Khoramjouy M, Naderi N, Kobarfard F, Heidarli E, Faizi M. An Intensified Acrolein Exposure Can Affect Memory and Cognition in Rat. Neurotox Res. 2021 Apr;39(2):277-291. doi: 10.1007/s12640-020-00278-x. Epub 2020 Sep 2. PMID: 32876917.

1843: Hall M, Walicka M, Panczyk M, Traczyk I. Metabolic Parameters in Patients with Suspected Reactive Hypoglycemia. J Pers Med. 2021 Apr 7;11(4):276. doi: 10.3390/jpm11040276. PMID: 33916952; PMCID: PMC8067537.

1844: Cabey K, Long DM, Law A, Gray NE, McClure C, Caruso M, Lak P, Wright KM, Stevens JF, Maier CS, Soumyanath A, Kretzschmar D. <i>Withania somnifera</i> and <i>Centella asiatica</i> Extracts Ameliorate Behavioral Deficits in an In Vivo <i>Drosophila melanogaster</i> Model of Oxidative Stress. Antioxidants (Basel). 2022 Jan 6;11(1):121. doi: 10.3390/antiox11010121. PMID: 35052625; PMCID: PMC8773428.

1845: Wang S, Ren Y, Fu X, Chen H, Ma H. [Application of an intervention plan

based on unplanned readmission risk model in the rehabilitation of patients with acute myocardial infarction complicated with cardiogenic shock after percutaneous coronary intervention]. Zhonghua Wei Zhong Bing Ji Jiu Yi Xue. 2021 Apr;33(4):487-490. Chinese. doi: 10.3760/cma.j.cn121430-20210304-00333. PMID: 34053496.

1846: Sangouni AA, Alizadeh M, Jamalzehi A, Parastouei K. Effects of garlic powder supplementation on metabolic syndrome components, insulin resistance, fatty liver index, and appetite in subjects with metabolic syndrome: A randomized clinical trial. Phytother Res. 2021 Aug;35(8):4433-4441. doi: 10.1002/ptr.7146. Epub 2021 May 11. PMID: 33974725.

1847: Baruteau J, Cunningham SC, Yilmaz BS, Perocheau DP, Eaglestone S, Burke D, Thrasher AJ, Waddington SN, Lisowski L, Alexander IE, Gissen P. Safety and efficacy of an engineered hepatotropic AAV gene therapy for ornithine transcarbamylase deficiency in cynomolgus monkeys. Mol Ther Methods Clin Dev. 2021 Sep 14;23:135-146. doi: 10.1016/j.omtm.2021.09.005. PMID: 34703837; PMCID: PMC8517016.

1848: Tatta J, Nijs J, Elma Ö, Malfliet A, Magnusson D. The Critical Role of Nutrition Care to Improve Pain Management: A Global Call to Action for Physical Therapist Practice. Phys Ther. 2022 Jan 3:pzab296. doi: 10.1093/ptj/pzab296. Epub ahead of print. PMID: 35023558.

1849: Pacheco LS, Lacey JV Jr, Martinez ME, Lemus H, Sears DD, Araneta MRG, Anderson CAM. Association Between Sugar-Sweetened Beverage Intake and Mortality Risk in Women: The California Teachers Study. J Acad Nutr Diet. 2022 Feb;122(2):320-333.e6. doi: 10.1016/j.jand.2021.08.099. Epub 2021 Aug 11. PMID: 34389488.

1850: Bartstra JW, Draaisma F, Zwakenberg SR, Lessmann N, Wolterink JM, van der Schouw YT, de Jong PA, Beulens JWJ. Six months vitamin K treatment does not affect systemic arterial calcification or bone mineral density in diabetes mellitus 2. Eur J Nutr. 2021 Apr;60(3):1691-1699. doi: 10.1007/s00394-020-02412-z. Epub 2020 Oct 17. PMID: 33068157; PMCID: PMC7987615.

1851: Conti MG, Terreri S, Piano Mortari E, Albano C, Natale F, Boscarino G, Zacco G, Palomba P, Cascioli S, Corrente F, Capponi C, Mirabella M, Salinas AF, Marciano A, De Luca F, Pangallo I, Quaranta C, Alteri C, Russo C, Galoppi P, Brunelli R, Perno CF, Terrin G, Carsetti R. Immune Response of Neonates Born to Mothers Infected With SARS-CoV-2. JAMA Netw Open. 2021 Nov 1;4(11):e2132563. doi: 10.1001/jamanetworkopen.2021.32563. PMID: 34730817; PMCID: PMC8567114.

1852: Li Y, Li L, Guo Z, Zhang S. Comparative effectiveness of furosemide vs torasemide in symptomatic therapy in heart failure patients: A randomized controlled study protocol. Medicine (Baltimore). 2021 Feb 19;100(7):e24661. doi: 10.1097/MD.0000000000024661. PMID: 33607802; PMCID: PMC7899842.

1853: Christensen KE, Malysheva OV, Carlin S, Matias F, MacFarlane AJ, Jacobs

RL, Caudill MA, Rozen R. Mild Choline Deficiency and MTHFD1 Synthetase Deficiency Interact to Increase Incidence of Developmental Delays and Defects in Mice. Nutrients. 2021 Dec 28;14(1):127. doi: 10.3390/nu14010127. PMID: 35011003; PMCID: PMC8747146.

1854: Naazie IN, Mwinyogle A, Nejim B, Al-Nouri O, Cajas-Monson L, Malas MB. The association of estimated glomerular filtration rate with outcomes following infrainguinal bypass for peripheral arterial disease. J Vasc Surg. 2021 Sep;74(3):788-797.e1. doi: 10.1016/j.jvs.2021.01.068. Epub 2021 Feb 26. PMID: 33647436.

1855: Cabalín C, Iturriaga C, Pérez-Mateluna G, Echeverría D, Camargo CA Jr, Borzutzky A. Vitamin D status and supplementation in Antarctica: a systematic review and meta- analysis. Int J Circumpolar Health. 2021 Dec;80(1):1926133. doi: 10.1080/22423982.2021.1926133. PMID: 33983101; PMCID: PMC8128169.

1856: Nonterah EA, Crowther NJ, Oduro A, Agongo G, Micklesfield LK, Boua PR, Choma SSR, Mohamed SF, Sorgho H, Tollman SM, Norris SA, Raal FJ, Grobbee DE, Ramsay M, Bots ML, Klipstein-Grobusch K; as part of the H3Africa AWI-Gen study. Poor cardiovascular health is associated with subclinical atherosclerosis in apparently healthy sub-Saharan African populations: an H3Africa AWI-Gen study. BMC Med. 2021 Feb 10;19(1):30. doi: 10.1186/s12916-021-01909-6. PMID: 33563289; PMCID: PMC7874493.

1857: Rozga M, Handu D, Kelley K, Jimenez EY, Martin H, Schofield M, Steiber A. Telehealth During the COVID-19 Pandemic: A Cross-Sectional Survey of Registered Dietitian Nutritionists. J Acad Nutr Diet. 2021 Dec;121(12):2524-2535. doi: 10.1016/j.jand.2021.01.009. Epub 2021 Jan 18. PMID: 33612436; PMCID: PMC7834621.

1858: Saberi Hosnijeh F, Casabonne D, Nieters A, Solans M, Naudin S, Ferrari P, Mckay JD, Benavente Y, Weiderpass E, Freisling H, Severi G, Boutron Ruault MC, Besson C, Agnoli C, Masala G, Sacerdote C, Tumino R, Huerta JM, Amiano P, Rodriguez-Barranco M, Bonet C, Barricarte A, Christakoudi S, Knuppel A, Bueno-de-Mesquita B, Schulze MB, Kaaks R, Canzian F, Späth F, Jerkeman M, Rylander C, Tjønneland A, Olsen A, Borch KB, Vermeulen R. Association between anthropometry and lifestyle factors and risk of B-cell lymphoma: An exposome-wide analysis. Int J Cancer. 2021 May 1;148(9):2115-2128. doi: 10.1002/ijc.33369. Epub 2020 Nov 12. PMID: 33128820; PMCID: PMC8048490.

1859: Walsh JJ, Caldwell HG, Neudorf H, Ainslie PN, Little JP. Short-term ketone monoester supplementation improves cerebral blood flow and cognition in obesity: A randomized cross-over trial. J Physiol. 2021 Nov;599(21):4763-4778. doi: 10.1113/JP281988. Epub 2021 Oct 4. PMID: 34605026.

1860: da Silveira AC, Leite ÁJM, Cabral PC, de Oliveira AC, de Oliveira KA, de Lira PIC. Toxic stress, health and nutrition among Brazilian children in shelters. BMC Pediatr. 2021 Mar 6;21(1):112. doi: 10.1186/s12887-021-02577-4. PMID: 33676454; PMCID: PMC7936454.

1861: Kumaran K, Krishnaveni GV, Suryanarayana KG, Prasad MP, Belavendra A, Atkinson S, Balasubramaniam R, Bandsma RHJ, Bhutta ZA, Chandak GR, Comelli EM, Davidge ST, Dennis CL, Hammond GL, Jha P, Joseph KS, Joshi SR, Krishna M, Lee K, Lye S, McGowan P, Nepomnaschy P, Padvetnaya V, Pyne S, Sachdev HS, Sahariah SA, Singhal N, Trasler J, Yajnik CS, Baird J, Barker M, Martin MC, Husain N, Sellen D, Fall CHD, Shah PS, Matthews SG. Protocol for a cluster randomised trial evaluating a multifaceted intervention starting preconceptionally-Early Interventions to Support Trajectories for Healthy Life in India (EINSTEIN): a Healthy Life Trajectories Initiative (HeLTI) Study. BMJ Open. 2021 Feb 16;11(2):e045862. doi: 10.1136/bmjopen-2020-045862. PMID: 33593789; PMCID: PMC7888364.

1862: Duarte-Hospital C, Tête A, Brial F, Benoit L, Koual M, Tomkiewicz C, Kim MJ, Blanc EB, Coumoul X, Bortoli S. Mitochondrial Dysfunction as a Hallmark of Environmental Injury. Cells. 2021 Dec 30;11(1):110. doi: 10.3390/cells11010110. PMID: 35011671; PMCID: PMC8750015.

1863: Kilonzo VW, Sasuclark AR, Torres DJ, Coyle C, Pilat JM, Williams CS, Pitts MW. Juvenile Selenium Deficiency Impairs Cognition, Sensorimotor Gating, and Energy Homeostasis in Mice. Front Nutr. 2021 May 7;8:667587. doi: 10.3389/fnut.2021.667587. PMID: 34026810; PMCID: PMC8138326.

1864: Huang T, Fu X, Wang N, Yang M, Zhang M, Wang B, Chen T, Majaz S, Wang H, Wong CW, Liu J, Guan M. Andrographolide prevents bone loss via targeting estrogen-related receptor-α-regulated metabolic adaption of osteoclastogenesis. Br J Pharmacol. 2021 Nov;178(21):4352-4367. doi: 10.1111/bph.15614. Epub 2021 Aug 24. PMID: 34233019.

1865: Sawh MC, Wallace M, Shapiro E, Goyal NP, Newton KP, Yu EL, Bross C, Durelle J, Knott C, Gangoiti JA, Barshop BA, Gengatharan JM, Meurs N, Schlein A, Middleton MS, Sirlin CB, Metallo CM, Schwimmer JB. Dairy Fat Intake, Plasma Pentadecanoic Acid, and Plasma Iso-heptadecanoic Acid Are Inversely Associated With Liver Fat in Children. J Pediatr Gastroenterol Nutr. 2021 Apr 1;72(4):e90-e96. doi: 10.1097/MPG.0000000000003040. PMID: 33399331.

1866: Smith SG, Sestak I, Morris MA, Harvie M, Howell A, Forbes J, Cuzick J. The impact of body mass index on breast cancer incidence among women at increased risk: an observational study from the International Breast Intervention Studies. Breast Cancer Res Treat. 2021 Jul;188(1):215-223. doi: 10.1007/s10549-021-06141-7. Epub 2021 Mar 3. PMID: 33656637; PMCID: PMC8233270.

1867: Tieu J, Cheah JT, Black RJ, Christensen R, Ghosh N, Richards P, Robson J, Shea B, Simon LS, Singhi JA, Tugwell P, Boers M, Garibay MAA, Campochiaro C, Decary S, de Witt M, Fernandez AP, Keen HI, King L, Hinojosa-Azaola A, Hofstetter C, Gaydukova I, George MD, Gupta L, Lyne S, Makol A, Mukhtyar C, Oo WM, Petri M, Pisaniello HL, Sattui SE, Russell O, Teixeira V, Toupin-April K, Uhunmwangho C, Whitstock M, Yip K, Mackie SL, Goodman SM, Hill CL. Improving benefit-harm assessment of glucocorticoid therapy incorporating the patient perspective: The OMERACT glucocorticoid core domain set. Semin Arthritis Rheum.

2021 Oct;51(5):1139-1145. doi: 10.1016/j.semarthrit.2021.06.010. Epub 2021 Jun 25. PMID: 34253398.

1868: Han Y, Hu Y, Yu C, Guo Y, Pei P, Yang L, Chen Y, Du H, Sun D, Pang Y, Chen N, Clarke R, Chen J, Chen Z, Li L, Lv J; China Kadoorie Biobank Collaborative Group. Lifestyle, cardiometabolic disease, and multimorbidity in a prospective Chinese study. Eur Heart J. 2021 Sep 7;42(34):3374-3384. doi: 10.1093/eurheartj/ehab413. PMID: 34333624; PMCID: PMC8423468.

1869: Corrao S, Mallaci Bocchio R, Lo Monaco M, Natoli G, Cavezzi A, Troiani E, Argano C. Does Evidence Exist to Blunt Inflammatory Response by Nutraceutical Supplementation during COVID-19 Pandemic? An Overview of Systematic Reviews of Vitamin D, Vitamin C, Melatonin, and Zinc. Nutrients. 2021 Apr 12;13(4):1261. doi: 10.3390/nu13041261. PMID: 33921297; PMCID: PMC8069903.

1870: Howard LSGE, He J, Watson GMJ, Huang L, Wharton J, Luo Q, Kiely DG, Condliffe R, Pepke-Zaba J, Morrell NW, Sheares KK, Ulrich A, Quan R, Zhao Z, Jing X, An C, Liu Z, Xiong C, Robbins PA, Dawes T, de Marvao A, Rhodes CJ, Richter MJ, Gall H, Ghofrani HA, Zhao L, Huson L, Wilkins MR. Supplementation with Iron in Pulmonary Arterial Hypertension. Two Randomized Crossover Trials. Ann Am Thorac Soc. 2021 Jun;18(6):981-988. doi: 10.1513/AnnalsATS.202009-1131OC. PMID: 33735594; PMCID: PMC8456720.

1871: Rist PM, Buring JE, Cook NR, Manson JE, Rexrode KM. Effect of vitamin D and/or omega-3 fatty acid supplementation on stroke outcomes: A randomized trial. Eur J Neurol. 2021 Mar;28(3):809-815. doi: 10.1111/ene.14623. Epub 2020 Nov 24. PMID: 33131164; PMCID: PMC7952033.

1872: Chen L, Tan Y, Yu C, Guo Y, Pei P, Yang L, Chen Y, Du H, Wang X, Chen J, Chen Z, Lv J, Li L; China Kadoorie Biobank Collaborative Group. Educational disparities in ischaemic heart disease among 0.5 million Chinese adults: a cohort study. J Epidemiol Community Health. 2021 Nov;75(11):1033-1043. doi: 10.1136/jech-2020-216314. Epub 2021 Mar 29. PMID: 33782052; PMCID: PMC8515104.

1873: Dagne S, Menber Y, Petrucka P, Wassihun Y. Prevalence and associated factors of abdominal obesity among the adult population in Woldia town, Northeast Ethiopia, 2020: Community-based cross-sectional study. PLoS One. 2021 Mar 8;16(3):e0247960. doi: 10.1371/journal.pone.0247960. PMID: 33684157; PMCID: PMC7939297.

1874: Zhu S, Li X, Song L, Huang Y, Xiao Y, Chu Q, Kang Y, Duan S, Wu D, Ren Z. Stachyose inhibits vancomycin-resistant Enterococcus colonization and affects gut microbiota in mice. Microb Pathog. 2021 Oct;159:105094. doi: 10.1016/j.micpath.2021.105094. Epub 2021 Jul 17. PMID: 34280500.

1875: Tanaka K, Okubo H, Sasaki S, Arakawa M, Miyake Y. Maternal caffeine intake during pregnancy and risk of food allergy in young Japanese children. J Paediatr Child Health. 2021 Jun;57(6):903-907. doi: 10.1111/jpc.15351. Epub 2021 Jan 19. PMID: 33464698.

1876: Basaqr R, Skleres M, Jayswal R, Thomas DT. The effect of dietary nitrate and vitamin C on endothelial function, oxidative stress and blood lipids in untreated hypercholesterolemic subjects: A randomized double-blind crossover study. Clin Nutr. 2021 Apr;40(4):1851-1860. doi: 10.1016/j.clnu.2020.10.012. Epub 2020 Oct 14. PMID: 33115598.

1877: Sikavi DR, Nguyen LH, Haruki K, Ugai T, Ma W, Wang DD, Thompson KN, Yan Y, Branck T, Wilkinson JE, Akimoto N, Zhong R, Lau MC, Mima K, Kosumi K, Morikawa T, Rimm EB, Garrett WS, Izard J, Cao Y, Song M, Huttenhower C, Ogino S, Chan AT. The Sulfur Microbial Diet and Risk of Colorectal Cancer by Molecular Subtypes and Intratumoral Microbial Species in Adult Men. Clin Transl Gastroenterol. 2021 Aug 1;12(8):e00338. doi: 10.14309/ctg.0000000000000338. PMID: 34333506; PMCID: PMC8323793.

1878: Wang X, Lin S, Gan S, Gu Y, Yang Y, Zhang Q, Liu L, Meng G, Yao Z, Zheng D, Wu H, Zhang S, Wang Y, Zhang T, Sun S, Jia Q, Song K, Wu XH, Wu Y, Niu K. Higher plain water intake is related to lower newly diagnosed nonalcoholic fatty liver disease risk: a population-based study. Eur J Clin Nutr. 2021 Dec;75(12):1801-1808. doi: 10.1038/s41430-021-00891-9. Epub 2021 Apr 9. PMID: 33837275.

1879: Segovia-Zafra A, Di Zeo-Sánchez DE, López-Gómez C, Pérez-Valdés Z, García-Fuentes E, Andrade RJ, Lucena MI, Villanueva-Paz M. Preclinical models of idiosyncratic drug-induced liver injury (iDILI): Moving towards prediction. Acta Pharm Sin B. 2021 Dec;11(12):3685-3726. doi: 10.1016/j.apsb.2021.11.013. Epub 2021 Nov 18. PMID: 35024301; PMCID: PMC8727925.

1880: Kangasmaa H, Tanner T, Laitala ML, Mulic A, Kopperud SE, Vähänikkilä H, Anttonen V, Alaraudanjoki V. Knowledge on and treatment practices of erosive tooth wear among Finnish dentists. Acta Odontol Scand. 2021 Oct;79(7):499-505. doi: 10.1080/00016357.2021.1896031. Epub 2021 Mar 9. PMID: 33689559.

1881: Wu PW, Tsai S, Lee CY, Lin WT, Chin YT, Huang HL, Seal DW, Chen T, Lee CH. Contribution of insulin resistance to the relationship between sugar-sweetened beverage intake and a constellation of cardiometabolic abnormalities in adolescents. Int J Obes (Lond). 2021 Apr;45(4):828-839. doi: 10.1038/s41366-021-00745-1. Epub 2021 Jan 20. PMID: 33473177.

1882: Luo F, Xing C, Asrani SK, Li S, Liang G, Hobbs HH, Cohen JC. Missense variant in insulin receptor (Y1355H) segregates in family with fatty liver disease. Mol Metab. 2021 Nov;53:101299. doi: 10.1016/j.molmet.2021.101299. Epub 2021 Jul 13. PMID: 34271222; PMCID: PMC8371211.

1883: Forte M, Marchitti S, Cotugno M, Di Nonno F, Stanzione R, Bianchi F, Schirone L, Schiavon S, Vecchio D, Sarto G, Scioli M, Raffa S, Tocci G, Relucenti M, Torrisi MR, Valenti V, Versaci F, Vecchione C, Volpe M, Frati G, Rubattu S, Sciarretta S. Trehalose, a natural disaccharide, reduces stroke occurrence in the stroke-prone spontaneously hypertensive rat. Pharmacol Res.

2021 Nov;173:105875. doi: 10.1016/j.phrs.2021.105875. Epub 2021 Sep 7. PMID: 34500062.

1884: Stephen R, Ngandu T, Liu Y, Peltonen M, Antikainen R, Kemppainen N, Laatikainen T, Lötjönen J, Rinne J, Strandberg T, Tuomilehto J, Vanninen R, Soininen H, Kivipelto M, Solomon A; FINGER Study Group. Change in CAIDE Dementia Risk Score and Neuroimaging Biomarkers During a 2-Year Multidomain Lifestyle Randomized Controlled Trial: Results of a Post-Hoc Subgroup Analysis. J Gerontol A Biol Sci Med Sci. 2021 Jul 13;76(8):1407-1414. doi: 10.1093/gerona/glab130. PMID: 33970268; PMCID: PMC8277089.

1885: Tsiogkas SG, Mavropoulos A, Skyvalidas DN, Patrikiou E, Ntavari N, Daponte AI, Grammatikopoulou MG, Dardiotis E, Roussaki-Schulze AV, Sakkas LI, Zafiriou E, Bogdanos DP. Delphinidin diminishes in vitro interferon-γ and interleukin-17 producing cells in patients with psoriatic disease. Immunol Res. 2021 Nov 25. doi: 10.1007/s12026-021-09251-y. Epub ahead of print. PMID: 34825313.

1886: Wu D, Plyku D, Kulkarni K, Garcia C, Atkins F, Tefera E, Burman KD, Wartofsky L, Van Nostrand D. Optimal Time for 124I PET/CT Imaging in Metastatic Differentiated Thyroid Cancer. Clin Nucl Med. 2021 Apr 1;46(4):283-288. doi: 10.1097/RLU.0000000000003505. PMID: 33492859.

1887: Heileson JL, Anzalone AJ, Carbuhn AF, Askow AT, Stone JD, Turner SM, Hillyer LM, Ma DWL, Luedke JA, Jagim AR, Oliver JM. The effect of omega-3 fatty acids on a biomarker of head trauma in NCAA football athletes: a multi-site, non-randomized study. J Int Soc Sports Nutr. 2021 Sep 27;18(1):65. doi: 10.1186/s12970-021-00461-1. PMID: 34579748; PMCID: PMC8477477.

1888: Nachit M, Kwanten WJ, Thissen JP, Op De Beeck B, Van Gaal L, Vonghia L, Verrijken A, Driessen A, Horsmans Y, Francque S, Leclercq IA. Muscle fat content is strongly associated with NASH: A longitudinal study in patients with morbid obesity. J Hepatol. 2021 Aug;75(2):292-301. doi: 10.1016/j.jhep.2021.02.037. Epub 2021 Apr 15. PMID: 33865909.

1889: Niknam Z, Jafari A, Golchin A, Danesh Pouya F, Nemati M, Rezaei-Tavirani M, Rasmi Y. Potential therapeutic options for COVID-19: an update on current evidence. Eur J Med Res. 2022 Jan 13;27(1):6. doi: 10.1186/s40001-021-00626-3. PMID: 35027080; PMCID: PMC8755901.

1890: Beulens JWJ, Pinho MGM, Abreu TC, den Braver NR, Lam TM, Huss A, Vlaanderen J, Sonnenschein T, Siddiqui NZ, Yuan Z, Kerckhoffs J, Zhernakova A, Brandao Gois MF, Vermeulen RCH. Environmental risk factors of type 2 diabetes-an exposome approach. Diabetologia. 2022 Feb;65(2):263-274. doi: 10.1007/s00125-021-05618-w. Epub 2021 Nov 18. PMID: 34792619.

1891: Bataille S, Pedinielli N, Carreno E, Prezelin-Reydit M, Chauveau P, Jean G, Robert T, Bobot M, Seret G, Jouve E, Lavainne F, Serveaux M, Vrigneaud L, Gentile S. VITADIAL "Does correction of 25 OH-VITAmin D with cholecalciferol supplementation increase muscle strength in hemoDIALysis patients?": study

protocol for a randomized controlled trial. Trials. 2021 May 25;22(1):364. doi: 10.1186/s13063-021-05302-9. PMID: 34034786; PMCID: PMC8146204.

1892: Vermandere K, Bostick RM, Tran HQ, Gewirtz AT, Barry EL, Rutherford RE, Seabrook ME, Fedirko V. Effects of Supplemental Calcium and Vitamin D on Circulating Biomarkers of Gut Barrier Function in Patients with Colon Adenoma: A Randomized Clinical Trial. Cancer Prev Res (Phila). 2021 Mar;14(3):393-402. doi: 10.1158/1940-6207.CAPR-20-0461. Epub 2020 Nov 23. PMID: 33229339; PMCID: PMC8137511.

1893: Salminen AV, Silvani A, Allen RP, Clemens S, Garcia-Borreguero D, Ghorayeb I, Ferré S, Li Y, Ondo W, Picchietti DL, Rye D, Siegel JM, Winkelman JW, Manconi M; International Restless Legs Syndrome Study Group (IRLSSG). Consensus Guidelines on Rodent Models of Restless Legs Syndrome. Mov Disord. 2021 Mar;36(3):558-569. doi: 10.1002/mds.28401. Epub 2020 Dec 31. PMID: 33382140; PMCID: PMC8313425.

1894: Mihaescu A, Masood E, Zafran M, Khokhar HT, Augustine AM, Filippo A, Van Biesen W, Farrigton K, Carrero JJ, Covic A, Nistor I. Nutritional status improvement in elderly CKD patients: a systematic review. Int Urol Nephrol. 2021 Aug;53(8):1603-1621. doi: 10.1007/s11255-020-02775-6. Epub 2021 Jan 18. PMID: 33459956.

1895: Al-Musharaf S, Aljuraiban G, Bogis R, Alnafisah R, Aldhwayan M, Tahrani A. Lifestyle changes associated with COVID-19 quarantine among young Saudi women: A prospective study. PLoS One. 2021 Apr 29;16(4):e0250625. doi: 10.1371/journal.pone.0250625. PMID: 33914800; PMCID: PMC8084143.

1896: Victoria-Montesinos D, Sánchez-Macarro M, Gabaldón-Hernández JA, Abellán-Ruiz MS, Querol-Calderón M, Luque-Rubia AJ, Bernal-Morell E, Ávila-Gandía V, López-Román FJ. Effect of Dietary Supplementation with a Natural Extract of <i>Sclerocarya birrea</i> on Glycemic Metabolism in Subjects with Prediabetes: A Randomized Double-Blind Placebo-Controlled Study. Nutrients. 2021 Jun 6;13(6):1948. doi: 10.3390/nu13061948. PMID: 34204042; PMCID: PMC8229573.

1897: Bibiloni MDM, Gallardo-Alfaro L, Gómez SF, Wärnberg J, Osés-Recalde M, González-Gross M, Gusi N, Aznar S, Marín-Cascales E, González-Valeiro M, Serra-Majem L, Terrados N, Segu M, Lassale C, Homs C, Benavente-Marín JC, Labayen I, Zapico AG, Sánchez-Gómez J, Jiménez-Zazo F, Alcaraz PE, Sevilla-Sánchez M, Herrera-Ramos E, Pulgar S, Sistac C, Schröder H, Bouzas C, Tur JA. Combined Body Mass Index and Waist-to-Height Ratio and Its Association with Lifestyle and Health Factors among Spanish Children: The PASOS Study. Nutrients. 2022 Jan 6;14(2):234. doi: 10.3390/nu14020234. PMID: 35057414; PMCID: PMC8781132.

1898: Elis A, Klempfner R, Gurevitz C, Gilady E, Goldenberg I. Apixaban in Patients with Atrial Fibrillation and Severe Renal Dysfunction: Findings from a National Registry. Isr Med Assoc J. 2021 Jun;23(6):353-358. PMID: 34155848.

1899: Carson AP, Long DL, Cherrington AL, Dutton GR, Howard VJ, Brown TM, Howard

G, Safford MM, Cushman M. Sex Differences in Factors Contributing to the Racial Disparity in Diabetes Risk. Am J Prev Med. 2021 Apr;60(4):e169-e177. doi: 10.1016/j.amepre.2020.09.016. Epub 2020 Dec 2. PMID: 33279367; PMCID: PMC7987785.

1900: Asbaghi O, Ashtary-Larky D, Bagheri R, Moosavian SP, Nazarian B, Afrisham R, Kelishadi MR, Wong A, Dutheil F, Suzuki K, Alavi Naeini A. Effects of Folic Acid Supplementation on Inflammatory Markers: A Grade-Assessed Systematic Review and Dose-Response Meta-Analysis of Randomized Controlled Trials. Nutrients. 2021 Jul 6;13(7):2327. doi: 10.3390/nu13072327. PMID: 34371837; PMCID: PMC8308638.

1901: Poles J, Karhu E, McGill M, McDaniel HR, Lewis JE. The effects of twenty-four nutrients and phytonutrients on immune system function and inflammation: A narrative review. J Clin Transl Res. 2021 May 27;7(3):333-376. PMID: 34239993; PMCID: PMC8259612.

1902: Rabelo ACS, Borghesi J, Noratto GD. The role of dietary polyphenols in osteosarcoma: A possible clue about the molecular mechanisms involved in a process that is just in its infancy. J Food Biochem. 2022 Jan;46(1):e14026. doi: 10.1111/jfbc.14026. Epub 2021 Dec 7. PMID: 34873724.

1903: Mathur P, Kulothungan V, Leburu S, Krishnan A, Chaturvedi HK, Salve HR, Amarchand R, Nongkynrih B, Kumar PG, K S VU, Ramakrishnan L, Laxmaiah A, Boruah M, Kumar S, Patro BK, Raghav PR, Rajkumar P, Sarma PS, Sharma R, Tambe M, Thankappan KR, Arlappa N, Mahanta TG, Joshi RP, Rustagi N, Gupta S, Behera BK, Shelke SC, Galhotra A, Bhuyan PJ, Pakhare AP, Kumar D, Topno RK, Gupta MK, Trivedi AV, Garg S. National noncommunicable disease monitoring survey (NNMS) in India: Estimating risk factor prevalence in adult population. PLoS One. 2021 Mar 2;16(3):e0246712. doi: 10.1371/journal.pone.0246712. PMID: 33651825; PMCID: PMC7924800.

1904: Li T, Yuan Q, Gong H, Du M, Mao X. Gut microbiota mediates the alleviative effect of polar lipids-enriched milk fat globule membrane on obesity-induced glucose metabolism disorders in peripheral tissues in rat dams. Int J Obes (Lond). 2022 Jan 29. doi: 10.1038/s41366-021-01029-4. Epub ahead of print. PMID: 35091670.

1905: Refaat MM, Gharios C, Moorthy MV, Abdulhai F, Blumenthal RS, Jaffa MA, Mora S. Exercise-Induced Ventricular Ectopy and Cardiovascular Mortality in Asymptomatic Individuals. J Am Coll Cardiol. 2021 Dec 7;78(23):2267-2277. doi: 10.1016/j.jacc.2021.09.1366. PMID: 34857087; PMCID: PMC8720278.

1906: Sondo P, Tahita MC, Rouamba T, Derra K, Kaboré B, Compaoré CS, Ouédraogo F, Rouamba E, Ilboudo H, Bambara EA, Nana M, Sawadogo EY, Sorgho H, Somé AM, Valéa I, Dahal P, Traoré/Coulibaly M, Tinto H. Assessment of a combined strategy of seasonal malaria chemoprevention and supplementation with vitamin A, zinc and Plumpy'Doz™ to prevent malaria and malnutrition in children under 5 years old in Burkina Faso: a randomized open-label trial (SMC-NUT). Trials. 2021 May 24;22(1):360. doi: 10.1186/s13063-021-05320-7. PMID: 34030705; PMCID:

PMC8142067.

1907: Huang G, Pencina K, Li Z, Apovian CM, Travison TG, Storer TW, Gagliano-Jucá T, Basaria S, Bhasin S. Effect of Protein Intake on Visceral Abdominal Fat and Metabolic Biomarkers in Older Men With Functional Limitations: Results From a Randomized Clinical Trial. J Gerontol A Biol Sci Med Sci. 2021 May 22;76(6):1084-1089. doi: 10.1093/gerona/glab007. PMID: 33417663; PMCID: PMC8140050.

1908: Osuna-Prieto FJ, Martinez-Tellez B, Ortiz-Alvarez L, Di X, Jurado-Fasoli L, Xu H, Ceperuelo-Mallafré V, Núñez-Roa C, Kohler I, Segura-Carretero A, García-Lario JV, Gil A, Aguilera CM, Llamas-Elvira JM, Rensen PCN, Vendrell J, Ruiz JR, Fernández-Veledo S. Elevated plasma succinate levels are linked to higher cardiovascular disease risk factors in young adults. Cardiovasc Diabetol. 2021 Jul 27;20(1):151. doi: 10.1186/s12933-021-01333-3. PMID: 34315463; PMCID: PMC8314524.

1909: Pickens CM, Flores-Ayala R, Ford ND, Whitehead RD, Tanumihardjo SA, Ngalombi S, Halati S, Mapango C, Sheftel J, Jefferds MED. Relation between Timing of High-Dose Vitamin A Supplementation and Modified-Relative-Dose-Response Values in Children 12-23 Months in Uganda. J Nutr. 2021 Apr 8;151(4):1025-1028. doi: 10.1093/jn/nxaa424. PMID: 33561264.

1910: Perna S, Ilyas Z, Giacosa A, Gasparri C, Peroni G, Faliva MA, Rigon C, Naso M, Riva A, Petrangolini G, A Redha A, Rondanelli M. Is Probiotic Supplementation Useful for the Management of Body Weight and Other Anthropometric Measures in Adults Affected by Overweight and Obesity with Metabolic Related Diseases? A Systematic Review and Meta-Analysis. Nutrients. 2021 Feb 19;13(2):666. doi: 10.3390/nu13020666. PMID: 33669580; PMCID: PMC7922558.

1911: Zhang Y, Yang H, Li S, Li WD, Wang Y. Consumption of coffee and tea and risk of developing stroke, dementia, and poststroke dementia: A cohort study in the UK Biobank. PLoS Med. 2021 Nov 16;18(11):e1003830. doi: 10.1371/journal.pmed.1003830. PMID: 34784347; PMCID: PMC8594796.

1912: Nevins JEH, Donovan SM, Snetselaar L, Dewey KG, Novotny R, Stang J, Taveras EM, Kleinman RE, Bailey RL, Raghavan R, Scinto-Madonich SR, Venkatramanan S, Butera G, Terry N, Altman J, Adler M, Obbagy JE, Stoody EE, de Jesus J. Omega-3 Fatty Acid Dietary Supplements Consumed During Pregnancy and Lactation and Child Neurodevelopment: A Systematic Review. J Nutr. 2021 Nov 2;151(11):3483-3494. doi: 10.1093/jn/nxab238. PMID: 34383914; PMCID: PMC8764572.

1913: van Zutphen M, Boshuizen HC, Kenkhuis MF, Wesselink E, Geijsen AJMR, de Wilt JHW, van Halteren HK, Spillenaar Bilgen EJ, Keulen ETP, Janssen-Heijnen MLG, Breukink SO, Bours MJL, Kok DE, Winkels RM, Weijenberg MP, Kampman E, van Duijnhoven FJB. Lifestyle after colorectal cancer diagnosis in relation to recurrence and all-cause mortality. Am J Clin Nutr. 2021 Jun 1;113(6):1447-1457. doi: 10.1093/ajcn/nqaa394. PMID: 33677488; PMCID: PMC8168353.

1914: Lechien JR, Hans S, De Marrez LG, Dequanter D, Rodriguez A, Muls V, Ben Abdelouahed F, Evrard L, Maniaci A, Saussez S, Bobin F. Prevalence and Features of Laryngopharyngeal Reflux in Patients with Primary Burning Mouth Syndrome. Laryngoscope. 2021 Oct;131(10):E2627-E2633. doi: 10.1002/lary.29604. Epub 2021 May 19. PMID: 34009647.

1915: Ramirez-Sanchez I, Navarrete-Yañez V, Garate-Carrillo A, Lara-Hernandez M, Espinosa-Raya J, Moreno-Ulloa A, Gomez-Diaz B, Cedeño-Garcidueñas AL, Ceballos G, Villarreal F. Restorative potential of (-)-epicatechin in a rat model of Gulf War illness muscle atrophy and fatigue. Sci Rep. 2021 Nov 8;11(1):21861. doi: 10.1038/s41598-021-01093-w. PMID: 34750405; PMCID: PMC8575952.

1916: Yang D, Lyu W, Hu Z, Gao J, Zheng Z, Wang W, Firrman J, Ren D. Probiotic Effects of <i>Lactobacillus fermentum</i> ZJUIDS06 and <i>Lactobacillus plantarum</i> ZY08 on Hypercholesteremic Golden Hamsters. Front Nutr. 2021 Jun 28;8:705763. doi: 10.3389/fnut.2021.705763. PMID: 34262929; PMCID: PMC8273167.

1917: Karampatsou SI, Genitsaridi SM, Michos A, Kourkouni E, Kourlaba G, Kassari P, Manios Y, Charmandari E. The Effect of a Life-Style Intervention Program of Diet and Exercise on Irisin and FGF-21 Concentrations in Children and Adolescents with Overweight and Obesity. Nutrients. 2021 Apr 13;13(4):1274. doi: 10.3390/nu13041274. PMID: 33924457; PMCID: PMC8070027.

1918: Wang Y, Zhang W, Xia F, Wan H, Chen C, Chen Y, Wang N, Lu Y. Moderation effect of economic status in the association between early life famine exposure and MAFLD in adulthood. Liver Int. 2022 Feb;42(2):299-308. doi: 10.1111/liv.15088. Epub 2021 Nov 17. PMID: 34687278.

1919: Vonaesch P, Djorie SG, Kandou KJE, Rakotondrainipiana M, Schaeffer L, Andriatsalama PV, Randriamparany R, Gondje BP, Nigatoloum S, Vondo SS, Etienne A, Robinson A, Hunald FA, Raharimalala L, Giles-Vernick T, Tondeur L, Randrianirina F, Bastaraud A, Gody JC, Sansonetti PJ, Randremanana RV; AFRIBIOTA Investigators. Factors Associated with Stunted Growth in Children Under Five Years in Antananarivo, Madagascar and Bangui, Central African Republic. Matern Child Health J. 2021 Oct;25(10):1626-1637. doi: 10.1007/s10995-021-03201-8. Epub 2021 Aug 12. PMID: 34383227; PMCID: PMC8448698.

1920: Kim IS, Hwang CW, Yang WS, Kim CH. Current Perspectives on the Physiological Activities of Fermented Soybean-Derived Cheonggukjang. Int J Mol Sci. 2021 May 27;22(11):5746. doi: 10.3390/ijms22115746. PMID: 34072216; PMCID: PMC8198423.

1921: Patti AM, Giglio RV, Papanas N, Serban D, Stoian AP, Pafili K, Al Rasadi K, Rajagopalan K, Rizvi AA, Ciaccio M, Rizzo M. Experimental and Emerging Free Fatty Acid Receptor Agonists for the Treatment of Type 2 Diabetes. Medicina (Kaunas). 2022 Jan 11;58(1):109. doi: 10.3390/medicina58010109. PMID: 35056417; PMCID: PMC8779029.

1922: Williams AM, Tanumihardjo SA, Rhodes EC, Mapango C, Kazembe B, Phiri F, Kang'ombe DD, Sheftel J, Orchardson V, Tripp K, Suchdev PS. Vitamin A deficiency has declined in Malawi, but with evidence of elevated vitamin A in children. Am J Clin Nutr. 2021 Apr 6;113(4):854-864. doi: 10.1093/ajcn/nqab004. PMID: 33751046; PMCID: PMC8023849.

1923: Munk T, Svendsen JA, Knudsen AW, Østergaard TB, Thomsen T, Olesen SS, Rasmussen HH, Beck AM. A multimodal nutritional intervention after discharge improves quality of life and physical function in older patients - a randomized controlled trial. Clin Nutr. 2021 Nov;40(11):5500-5510. doi: 10.1016/j.clnu.2021.09.029. Epub 2021 Sep 24. PMID: 34656032.

1924: Nguyen ML, Sachdev V, Burklow TR, Li W, Startzell M, Auh S, Brown RJ. Leptin Attenuates Cardiac Hypertrophy in Patients With Generalized Lipodystrophy. J Clin Endocrinol Metab. 2021 Oct 21;106(11):e4327-e4339. doi: 10.1210/clinem/dgab499. PMID: 34223895; PMCID: PMC8530723.

1925: Ali Z, Waseem S, Shahzadi I, Bukhari S, Anis RA, Ahmed I, Anees M. Association of cell free mitochondrial DNA and caspase-1 expression with disease severity and ARTs efficacy in HIV infection. Mol Biol Rep. 2021 Apr;48(4):3327-3336. doi: 10.1007/s11033-021-06313-0. Epub 2021 Apr 22. PMID: 33886057.

1926: Jiang S, Shui Y, Cui Y, Tang C, Wang X, Qiu X, Hu W, Fei L, Li Y, Zhang S, Zhao L, Xu N, Dong F, Ren X, Liu R, Persson PB, Patzak A, Lai EY, Wei Q, Zheng Z. Gut microbiota dependent trimethylamine N-oxide aggravates angiotensin II-induced hypertension. Redox Biol. 2021 Oct;46:102115. doi: 10.1016/j.redox.2021.102115. Epub 2021 Aug 25. PMID: 34474396; PMCID: PMC8408632.

1927: Bae S, Kamynina E, Guetterman HM, Farinola AF, Caudill MA, Berry RJ, Cassano PA, Stover PJ. Provision of folic acid for reducing arsenic toxicity in arsenic-exposed children and adults. Cochrane Database Syst Rev. 2021 Oct 18;10(10):CD012649. doi: 10.1002/14651858.CD012649.pub2. PMID: 34661903; PMCID: PMC8522704.

1928: Isanaka S, Garba S, Plikaytis B, Malone McNeal M, Guindo O, Langendorf C, Adehossi E, Ciglenecki I, Grais RF. Immunogenicity of an oral rotavirus vaccine administered with prenatal nutritional support in Niger: A cluster randomized clinical trial. PLoS Med. 2021 Aug 10;18(8):e1003720. doi: 10.1371/journal.pmed.1003720. Erratum in: PLoS Med. 2021 Oct 15;18(10):e1003776. PMID: 34375336; PMCID: PMC8354620.

1929: Ferrières J, Banks V, Pillas D, Giorgianni F, Gantzer L, Lekens B, Ricci L, Dova-Boivin M, Chauny JV, Villa G, Désaméricq G. Screening and treatment of familial hypercholesterolemia in a French sample of ambulatory care patients: A retrospective longitudinal cohort study. PLoS One. 2021 Aug 2;16(8):e0255345. doi: 10.1371/journal.pone.0255345. PMID: 34339471; PMCID: PMC8328334.

1930: Wang PY, Chen XK, Liu Q, Xu L, Zhang RX, Liu XB, Li Y. Application of four nutritional risk indexes in perioperative management for esophageal cancer patients. J Cancer Res Clin Oncol. 2021 Oct;147(10):3099-3111. doi: 10.1007/s00432-021-03585-8. Epub 2021 Mar 9. PMID: 33687565; PMCID: PMC7941130.

1931: Linseisen J, Grundmann N, Zoller D, Kühn T, Jansen EHJM, Chajès V, Fedirko V, Weiderpass E, Dahm CC, Overvad K, Tjønneland A, Boutron-Ruault MC, Rothwell JA, Severi G, Kaaks R, Schulze MB, Aleksandrova K, Sieri S, Panico S, Tumino R, Masala G, De Marco L, Bueno-de-Mesquita B, Vermeulen R, Gram IT, Skeie G, Chirlaque MD, Ardanaz E, Agudo A, Sánchez MJ, Amiano P, Wennberg M, Bodén S, Perez-Cornago A, Aglago EK, Gunter MJ, Jenab M, Heath AK, Nieters A. Red Blood Cell Fatty Acids and Risk of Colorectal Cancer in The European Prospective Investigation into Cancer and Nutrition (EPIC). Cancer Epidemiol Biomarkers Prev. 2021 May;30(5):874-885. doi: 10.1158/1055-9965.EPI-20-1426. Epub 2021 Feb 22. PMID: 33619024.

1932: Pang X, Yang Z, Wang J, Duan Y, Zhao L, Yu D, Lai J. Relationship between Serum 25OH-Vitamin D2 Level and Vitamin D Status of Children Aged 3-5 Years in China. Nutrients. 2021 Nov 19;13(11):4135. doi: 10.3390/nu13114135. PMID: 34836390; PMCID: PMC8619457.

1933: Alferink LJM, Radjabzadeh D, Erler NS, Vojinovic D, Medina-Gomez C, Uitterlinden AG, de Knegt RJ, Amin N, Ikram MA, Janssen HLA, Kiefte-de Jong JC, Metselaar HJ, van Duijn CM, Kraaij R, Darwish Murad S. Microbiomics, Metabolomics, Predicted Metagenomics, and Hepatic Steatosis in a Population-Based Study of 1,355 Adults. Hepatology. 2021 Mar;73(3):968-982. doi: 10.1002/hep.31417. PMID: 32530501.

1934: Joh HK, Lee DH, Hur J, Nimptsch K, Chang Y, Joung H, Zhang X, Rezende LFM, Lee JE, Ng K, Yuan C, Tabung FK, Meyerhardt JA, Chan AT, Pischon T, Song M, Fuchs CS, Willett WC, Cao Y, Ogino S, Giovannucci E, Wu K. Simple Sugar and Sugar-Sweetened Beverage Intake During Adolescence and Risk of Colorectal Cancer Precursors. Gastroenterology. 2021 Jul;161(1):128-142.e20. doi: 10.1053/j.gastro.2021.03.028. Epub 2021 Mar 19. PMID: 33753105; PMCID: PMC8238879.

1935: Anderson AS, Martin RM, Renehan AG, Cade J, Copson ER, Cross AJ, Grimmett C, Keaver L, King A, Riboli E, Shaw C, Saxton JM; UK NIHR Cancer and Nutrition Collaboration (Population Health Stream). Cancer survivorship, excess body fatness and weight-loss intervention-where are we in 2020? Br J Cancer. 2021 Mar;124(6):1057-1065. doi: 10.1038/s41416-020-01155-2. Epub 2020 Nov 25. PMID: 33235316; PMCID: PMC7961062.

1936: Machicado JD, Wani S, Quingalahua E, Han S, Simon V, Hegyi P, Papachristou GI, Yadav D. Practice patterns and adherence to nutrition guidelines in acute pancreatitis: An international physician survey. Pancreatology. 2021 Apr;21(3):642-648. doi: 10.1016/j.pan.2021.01.001. Epub 2021 Jan 14. PMID: 33632665.

1937: Bennett CT, Robertson A. Depuration Kinetics and Growth Dilution of Caribbean Ciguatoxin in the Omnivore <i>Lagodon rhomboides:</i> Implications for Trophic Transfer and Ciguatera Risk. Toxins (Basel). 2021 Nov 1;13(11):774. doi: 10.3390/toxins13110774. PMID: 34822558; PMCID: PMC8623479.

1938: Martens N, Schepers M, Zhan N, Leijten F, Voortman G, Tiane A, Rombaut B, Poisquet J, Sande NV, Kerksiek A, Kuipers F, Jonker JW, Liu H, Lütjohann D, Vanmierlo T, Mulder MT. 24(S)-Saringosterol Prevents Cognitive Decline in a Mouse Model for Alzheimer's Disease. Mar Drugs. 2021 Mar 27;19(4):190. doi: 10.3390/md19040190. PMID: 33801706; PMCID: PMC8065937.

1939: Ma H, Zhou T, Heianza Y, Qi L. Habitual use of vitamin D supplements and risk of coronavirus disease 2019 (COVID-19) infection: a prospective study in UK Biobank. Am J Clin Nutr. 2021 May 8;113(5):1275-1281. doi: 10.1093/ajcn/nqaa381. PMID: 33515005; PMCID: PMC7929381.

1940: Koole JL, Bours MJL, Geijsen AJMR, Gigic B, Ulvik A, Kok DE, Brezina S, Ose J, Baierl A, Böhm J, Brenner H, Breukink SO, Chang-Claude J, van Duijnhoven FJB, van Duijvendijk P, Gumpenberger T, Habermann N, van Halteren HK, Hoffmeister M, Holowatyj AN, Janssen-Heijnen MLG, Keulen ETP, Kiblawi R, Kruyt FM, Li CI, Lin T, Midttun Ø, Peoples AR, van Roekel EH, Schneider MA, Schrotz-King P, Ulrich AB, Vickers K, Wesselink E, de Wilt JHW, Gsur A, Ueland PM, Ulrich CM, Kampman E, Weijenberg MP. Circulating B-vitamin biomarkers and B-vitamin supplement use in relation to quality of life in patients with colorectal cancer: results from the FOCUS consortium. Am J Clin Nutr. 2021 Jun 1;113(6):1468-1481. doi: 10.1093/ajcn/nqaa422. PMID: 33668069; PMCID: PMC8168355.

1941: Pena MJ, Costa R, Rodrigues I, Martins S, Guimarães JT, Faria A, Calhau C, Rocha JC, Borges N. Unveiling the Metabolic Effects of Glycomacropeptide. Int J Mol Sci. 2021 Sep 8;22(18):9731. doi: 10.3390/ijms22189731. PMID: 34575895; PMCID: PMC8470927.

1942: Genton L, Teta D, Pruijm M, Stoermann C, Marangon N, Mareschal J, Bassi I, Wurzner-Ghajarzadeh A, Lazarevic V, Cynober L, Cani PD, Herrmann FR, Schrenzel J. Glycine increases fat-free mass in malnourished haemodialysis patients: a randomized double-blind crossover trial. J Cachexia Sarcopenia Muscle. 2021 Dec;12(6):1540-1552. doi: 10.1002/jcsm.12780. Epub 2021 Sep 14. PMID: 34519439; PMCID: PMC8718019.

1943: Muñoz-Esparza NC, Costa-Catala J, Comas-Basté O, Toro-Funes N, Latorre-Moratalla ML, Veciana-Nogués MT, Vidal-Carou MC. Occurrence of Polyamines in Foods and the Influence of Cooking Processes. Foods. 2021 Jul 29;10(8):1752. doi: 10.3390/foods10081752. PMID: 34441529; PMCID: PMC8392025.

1944: Lee MKS, Kraakman MJ, Dragoljevic D, Hanssen NMJ, Flynn MC, Al-Sharea A, Sreejit G, Bertuzzo-Veiga C, Cooney OD, Baig F, Morriss E, Cooper ME, Josefsson EC, Kile BT, Nagareddy PR, Murphy AJ. Apoptotic Ablation of Platelets Reduces Atherosclerosis in Mice With Diabetes. Arterioscler Thromb Vasc Biol. 2021

Mar;41(3):1167-1178. doi: 10.1161/ATVBAHA.120.315369. Epub 2021 Jan 14. PMID: 33441028; PMCID: PMC7904582.

1945: Boonyakrai C, Kanjanabuch T, Puapatanakul P, Halue G, Johnson DW, Lorvinitnun P, Tangjittrong K, Kittiskulnam P, Pongpirul K, Bieber B, Tungsanga K; Thailand PDOPPS Steering Committee. Association between self-reported appetite and clinical outcomes of peritoneal dialysis patients: Findings from a low middle-income country. Nephrology (Carlton). 2021 May;26(5):454-462. doi: 10.1111/nep.13859. Epub 2021 Mar 10. PMID: 33550668.

1946: Arroyo-Díaz JA, Julve J, Vlacho B, Corcoy R, Ponte P, Román E, Navas-Méndez E, Llauradó G, Franch-Nadal J, Domingo P, Mauricio D. Previous Vitamin D Supplementation and Morbidity and Mortality Outcomes in People Hospitalised for COVID19: A Cross-Sectional Study. Front Public Health. 2021 Sep 24;9:758347. doi: 10.3389/fpubh.2021.758347. PMID: 34631653; PMCID: PMC8498099.

1947: Lechien JR, Hans S, Bobin F, Calvo-Henriquez C, Saussez S, Karkos PD. Atypical Clinical Presentation of Laryngopharyngeal Reflux: A 5-Year Case Series. J Clin Med. 2021 May 31;10(11):2439. doi: 10.3390/jcm10112439. PMID: 34072701; PMCID: PMC8198232.

1948: Ajana S, Cougnard-Grégoire A, Colijn JM, Merle BMJ, Verzijden T, de Jong PTVM, Hofman A, Vingerling JR, Hejblum BP, Korobelnik JF, Meester-Smoor MA, Ueffing M, Jacqmin-Gadda H, Klaver CCW, Delcourt C; EYE-RISK Consortium. Predicting Progression to Advanced Age-Related Macular Degeneration from Clinical, Genetic, and Lifestyle Factors Using Machine Learning. Ophthalmology. 2021 Apr;128(4):587-597. doi: 10.1016/j.ophtha.2020.08.031. Epub 2020 Sep 2. PMID: 32890546.

1949: Boakye D, Jansen L, Schöttker B, Jansen EHJM, Halama N, Maalmi H, Gào X, Chang-Claude J, Hoffmeister M, Brenner H. The association of vitamin D with survival in colorectal cancer patients depends on antioxidant capacity. Am J Clin Nutr. 2021 Jun 1;113(6):1458-1467. doi: 10.1093/ajcn/nqaa405. PMID: 33740035.

1950: Colaco NA, Wang TS, Ma Y, Scherzer R, Ilkayeva OR, Desvigne-Nickens P, Braunwald E, Hernandez AF, Butler J, Shah SH, Shah SJ, Hsue PY. Transmethylamine-N-Oxide Is Associated With Diffuse Cardiac Fibrosis in People Living With HIV. J Am Heart Assoc. 2021 Aug 17;10(16):e020499. doi: 10.1161/JAHA.120.020499. Epub 2021 Aug 7. PMID: 34365799; PMCID: PMC8475032.

1951: Govaere O, Petersen SK, Martinez-Lopez N, Wouters J, Van Haele M, Mancina RM, Jamialahmadi O, Bilkei-Gorzo O, Lassen PB, Darlay R, Peltier J, Palmer JM, Younes R, Tiniakos D, Aithal GP, Allison M, Vacca M, Göransson M, Berlinguer-Palmini R, Clark JE, Drinnan MJ, Yki-Järvinen H, Dufour JF, Ekstedt M, Francque S, Petta S, Bugianesi E, Schattenberg JM, Day CP, Cordell HJ, Topal B, Clément K, Romeo S, Ratziu V, Roskams T, Daly AK, Anstee QM, Trost M, Härtlova A. Macrophage Scavenger Receptor 1 mediates lipid-induced inflammation in non-alcoholic fatty liver disease. J Hepatol. 2021 Dec 20:S0168-8278(21)02254-6.

doi: 10.1016/j.jhep.2021.12.012. Epub ahead of print. PMID: 34942286.

1952: Katani R, Schilling MA, Lyimo B, Eblate E, Martin A, Tonui T, Cattadori IM, Francesconi SC, Estes AB, Rentsch D, Srinivasan S, Lyimo S, Munuo L, Tiambo CK, Stomeo F, Gwakisa P, Mosha F, Hudson PJ, Buza JJ, Kapur V. Identification of Bacillus anthracis, Brucella spp., and Coxiella burnetii DNA signatures from bushmeat. Sci Rep. 2021 Jul 21;11(1):14876. doi: 10.1038/s41598-021-94112-9. PMID: 34290271; PMCID: PMC8295346.

1953: Battaglia Y, Bellasi A, Bortoluzzi A, Tondolo F, Esposito P, Provenzano M, Russo D, Andreucci M, Cianciolo G, Storari A. Bone Mineral Density Changes in Long-Term Kidney Transplant Recipients: A Real-Life Cohort Study of Native Vitamin D Supplementation. Nutrients. 2022 Jan 13;14(2):323. doi: 10.3390/nu14020323. PMID: 35057505; PMCID: PMC8780110.

1954: Pérez-Castrillón JL, Dueñas-Laita A, Brandi ML, Jódar E, Del Pino-Montes J, Quesada-Gómez JM, Cereto Castro F, Gómez-Alonso C, Gallego López L, Olmos Martínez JM, Alhambra Expósito MR, Galarraga B, González-Macías J, Bouillon R, Hernández-Herrero G, Fernández-Hernando N, Arranz-Gutiérrez P, Chinchilla SP. Calcifediol is superior to cholecalciferol in improving vitamin D status in postmenopausal women: a randomized trial. J Bone Miner Res. 2021 Oct;36(10):1967-1978. doi: 10.1002/jbmr.4387. Epub 2021 Jun 24. PMID: 34101900; PMCID: PMC8597097.

1955: Moodi V, Abedi S, Esmaeilpour M, Asbaghi O, Izadi F, Shirinbakhshmasoleh M, Behrouzian M, Shahriari A, Ghaedi E, Miraghajani M. The effect of grapes/grape products on glycemic response: A systematic review and meta-analysis of randomized controlled trials. Phytother Res. 2021 Sep;35(9):5053-5067. doi: 10.1002/ptr.7135. Epub 2021 Apr 24. PMID: 33893683.

1956: Desgrouas M, Merdji H, Bretagnol A, Barin-Le Guellec C, Halimi JM, Ehrmann S, Salmon Gandonnière C. Kinetic Glomerular Filtration Rate Equations in Patients With Shock: Comparison With the Iohexol-Based Gold-Standard Method. Crit Care Med. 2021 Aug 1;49(8):e761-e770. doi: 10.1097/CCM.0000000000004946. PMID: 33710029.

1957: Jakimiuk K, Sari S, Milewski R, Supuran CT, Şöhretoğlu D, Tomczyk M. Flavonoids as tyrosinase inhibitors in <i>in silico</i> and <i>in vitro</i> models: basic framework of SAR using a statistical modelling approach. J Enzyme Inhib Med Chem. 2022 Dec;37(1):421-430. doi: 10.1080/14756366.2021.2014832. Erratum in: J Enzyme Inhib Med Chem. 2022 Dec;37(1):514. PMID: 34923888; PMCID: PMC8735877.

1958: Guasch-Ferré M, Li Y, Willett WC, Sun Q, Sampson L, Salas-Salvadó J, Martínez-González MA, Stampfer MJ, Hu FB. Consumption of Olive Oil and Risk of Total and Cause-Specific Mortality Among U.S. Adults. J Am Coll Cardiol. 2022 Jan 18;79(2):101-112. doi: 10.1016/j.jacc.2021.10.041. PMID: 35027106.

1959: Toupin-April K, Décary S, de Wit M, Meara A, Barton JL, Fraenkel L, Li LC,

Brooks P, Shea B, Stacey D, Légaré F, Lydiatt A, Hofstetter C, Proulx L, Christensen R, Voshaar M, Suarez-Almazor ME, Boonen A, Meade T, March L, Jull JE, Campbell W, Alten R, Morgan EM, Kelly A, Kaufman J, Hill S, Maxwell LJ, Guillemin F, Beaton D, El-Miedany Y, Mittoo S, Westrich Robertson T, Bartlett SJ, Singh JA, Mannion M, Nasef SI, de Souza S, Boel A, Adebajo A, Arnaud L, Gill TK, Moholt E, Burt J, Jayatilleke A, Hmamouchi I, Carrott D, Blanco FJ, Mather K, Maharaj A, Sharma S, Caso F, Fong C, Fernandez AP, Mackie S, Nikiphorou E, Jones A, Greer-Smith R, Sloan VS, Akpabio A, Strand V, Umaefulam V, Monti S, Melburn C, Abaza N, Schultz K, Stones S, Kiwalkar S, Srinivasalu H, Constien D, King LK, Tugwell P. Endorsement of the OMERACT core domain set for shared decision making interventions in rheumatology trials: Results from a multi-stepped consensus-building approach. Semin Arthritis Rheum. 2021 Jun;51(3):593-600. doi: 10.1016/j.semarthrit.2021.03.017. Epub 2021 Apr 6. PMID: 33892937.

1960: Salmani M, Alipoor E, Navid H, Farahbakhsh P, Yaseri M, Imani H. Effect of l-arginine on cardiac reverse remodeling and quality of life in patients with heart failure. Clin Nutr. 2021 May;40(5):3037-3044. doi: 10.1016/j.clnu.2021.01.044. Epub 2021 Feb 5. PMID: 33610421.

1961: Martinez-Perez C, Daimiel L, Climent-Mainar C, Martínez-González MÁ, Salas-Salvadó J, Corella D, Schröder H, Martinez JA, Alonso-Gómez ÁM, Wärnberg J, Vioque J, Romaguera D, López-Miranda J, Estruch R, Tinahones FJ, Lapetra J, Serra-Majem L, Bueno-Cavanillas A, Tur JA, Sánchez VM, Pintó X, Delgado-Rodríguez M, Matía-Martín P, Vidal J, Vázquez C, Ros E, Basterra J, Babio N, Guillem-Saiz P, Zomeño MD, Abete I, Vaquero-Luna J, Barón-López FJ, Gonzalez-Palacios S, Konieczna J, Garcia-Rios A, Bernal-López MR, Santos-Lozano JM, Bes-Rastrollo M, Khoury N, Saiz C, Pérez-Vega KA, Zulet MA, Tojal-Sierra L, Ruiz ZV, Martinez MA, Malcampo M, Ordovás JM, San-Cristobal R. Integrative development of a short screening questionnaire of highly processed food consumption (sQ-HPF). Int J Behav Nutr Phys Act. 2022 Jan 24;19(1):6. doi: 10.1186/s12966-021-01240-6. PMID: 35073909; PMCID: PMC8785596.

1962: Haufe S, Hupa-Breier KL, Bayerle P, Boeck HT, Rolff S, Sundermeier T, Kerling A, Eigendorf J, Kück M, Hanke AA, Ensslen R, Nachbar L, Lauenstein D, Böthig D, Hilfiker-Kleiner D, Stiesch M, Terkamp C, Wedemeyer H, Haverich A, Tegtbur U. Telemonitoring-Supported Exercise Training in Employees With Metabolic Syndrome Improves Liver Inflammation and Fibrosis. Clin Transl Gastroenterol. 2021 Jun 18;12(6):e00371. doi: 10.14309/ctg.0000000000000371. PMID: 34140456; PMCID: PMC8216678.

1963: Cuzzubbo S, Mangsbo S, Nagarajan D, Habra K, Pockley AG, McArdle SEB. Cancer Vaccines: Adjuvant Potency, Importance of Age, Lifestyle, and Treatments. Front Immunol. 2021 Feb 17;11:615240. doi: 10.3389/fimmu.2020.615240. PMID: 33679703; PMCID: PMC7927599.

1964: Yuan WL, Kramer MS, Michael N, Sadananthan SA, Tint MT, Chen LW, Pang WW, Velan SS, Godfrey KM, Chong YS, Chong MFF, Choo JTL, Ling LH, Eriksson JG, Lee YS. Trajectories of Systolic Blood Pressure in Children: Risk Factors and

Cardiometabolic Correlates. J Pediatr. 2021 Sep;236:86-94.e6. doi: 10.1016/j.jpeds.2021.05.027. Epub 2021 May 18. PMID: 34019883; PMCID: PMC7611585.

1965: Mishra A, Bentur SA, Thakral S, Garg R, Duggal B. The use of integrative therapy based on Yoga and Ayurveda in the treatment of a high-risk case of COVID-19/SARS-CoV-2 with multiple comorbidities: a case report. J Med Case Rep. 2021 Feb 24;15(1):95. doi: 10.1186/s13256-020-02624-1. PMID: 33627186; PMCID: PMC7903378.

1966: Butler-Laporte G, Nakanishi T, Mooser V, Morrison DR, Abdullah T, Adeleye O, Mamlouk N, Kimchi N, Afrasiabi Z, Rezk N, Giliberti A, Renieri A, Chen Y, Zhou S, Forgetta V, Richards JB. Vitamin D and COVID-19 susceptibility and severity in the COVID-19 Host Genetics Initiative: A Mendelian randomization study. PLoS Med. 2021 Jun 1;18(6):e1003605. doi: 10.1371/journal.pmed.1003605. PMID: 34061844; PMCID: PMC8168855.

1967: Tsilidis KK, Papadimitriou N, Dimou N, Gill D, Lewis SJ, Martin RM, Murphy N, Markozannes G, Zuber V, Cross AJ, Burrows K, Lopez DS, Key TJ, Travis RC, Perez-Cornago A, Hunter DJ, van Duijnhoven FJB, Albanes D, Arndt V, Berndt SI, Bézieau S, Bishop DT, Boehm J, Brenner H, Burnett-Hartman A, Campbell PT, Casey G, Castellví-Bel S, Chan AT, Chang-Claude J, de la Chapelle A, Figueiredo JC, Gallinger SJ, Giles GG, Goodman PJ, Gsur A, Hampe J, Hampel H, Hoffmeister M, Jenkins MA, Keku TO, Kweon SS, Larsson SC, Le Marchand L, Li CI, Li L, Lindblom A, Martín V, Milne RL, Moreno V, Nan H, Nassir R, Newcomb PA, Offit K, Pharoah PDP, Platz EA, Potter JD, Qi L, Rennert G, Sakoda LC, Schafmayer C, Slattery ML, Snetselaar L, Schenk J, Thibodeau SN, Ulrich CM, Van Guelpen B, Harlid S, Visvanathan K, Vodickova L, Wang H, White E, Wolk A, Woods MO, Wu AH, Zheng W, Bueno-de-Mesquita B, Boutron-Ruault MC, Hughes DJ, Jakszyn P, Kühn T, Palli D, Riboli E, Giovannucci EL, Banbury BL, Gruber SB, Peters U, Gunter MJ. Genetically predicted circulating concentrations of micronutrients and risk of colorectal cancer among individuals of European descent: a Mendelian randomization study. Am J Clin Nutr. 2021 Jun 1;113(6):1490-1502. doi: 10.1093/ajcn/nqab003. Erratum in: Am J Clin Nutr. 2021 Jun 1;113(6):1715. PMID: 33740060; PMCID: PMC8168352.

1968: Tran DL, Gibson H, Maiorana AJ, Verrall CE, Baker DW, Clode M, Lubans DR, Zannino D, Bullock A, Ferrie S, Briody J, Simm P, Wijesekera V, D'Almeida M, Gosbell SE, Davis GM, Weintraub R, Keech AC, Puranik R, Ugander M, Justo R, Zentner D, Majumdar A, Grigg L, Coombes JS, d'Udekem Y, Morris NR, Ayer J, Celermajer DS, Cordina R. Exercise Intolerance, Benefits, and Prescription for People Living With a Fontan Circulation: The Fontan Fitness Intervention Trial (F-FIT)-Rationale and Design. Front Pediatr. 2022 Jan 6;9:799125. doi: 10.3389/fped.2021.799125. PMID: 35071139; PMCID: PMC8771702.

1969: Vallejo AN, Mroczkowski HJ, Michel JJ, Woolford M, Blair HC, Griffin P, McCracken E, Mihalik SJ, Reyes-Mugica M, Vockley J. Pervasive inflammatory activation in patients with deficiency in very-long-chain acyl-coA dehydrogenase (VLCADD). Clin Transl Immunology. 2021 Jun 27;10(6):e1304. doi:

10.1002/cti2.1304. PMID: 34194748; PMCID: PMC8236555.

1970: Napoli A, Sciacca L, Pintaudi B, Tumminia A, Dalfrà MG, Festa C, Formoso G, Fresa R, Graziano G, Lencioni C, Nicolucci A, Rossi MC, Succurro E, Sculli MA, Scavini M, Vitacolonna E, Bonomo M, Torlone E; STRONG Study Collaborators and the AMD-SID Diabetes and Pregnancy Study Group. Screening of postpartum diabetes in women with gestational diabetes: high-risk subgroups and areas for improvements-the STRONG observational study. Acta Diabetol. 2021 Sep;58(9):1187-1197. doi: 10.1007/s00592-021-01707-9. Epub 2021 Apr 12. Erratum in: Acta Diabetol. 2021 Jun 11;: PMID: 33842997; PMCID: PMC8316164.

1971: Lin X, Yao H, Guo J, Huang Y, Wang W, Yin B, Li X, Wang T, Li C, Xu X, Zhou G, Voglmeir J, Liu L. Protein Glycosylation and Gut Microbiota Utilization Can Limit the In Vitro and In Vivo Metabolic Cellular Incorporation of Neu5Gc. Mol Nutr Food Res. 2021 Dec 18:e2100615. doi: 10.1002/mnfr.202100615. Epub ahead of print. PMID: 34921741.

1972: Novak Z, Zaky A, Spangler EL, McFarland GE, Tolwani A, Beck AW. Incidence and predictors of early and delayed renal function decline after aortic aneurysm repair in the Vascular Quality Initiative database. J Vasc Surg. 2021 Nov;74(5):1537-1547. doi: 10.1016/j.jvs.2021.04.049. Epub 2021 May 18. PMID: 34019992.

1973: Mallick H, Rahnavard A, McIver LJ, Ma S, Zhang Y, Nguyen LH, Tickle TL, Weingart G, Ren B, Schwager EH, Chatterjee S, Thompson KN, Wilkinson JE, Subramanian A, Lu Y, Waldron L, Paulson JN, Franzosa EA, Bravo HC, Huttenhower C. Multivariable association discovery in population-scale meta-omics studies. PLoS Comput Biol. 2021 Nov 16;17(11):e1009442. doi: 10.1371/journal.pcbi.1009442. PMID: 34784344; PMCID: PMC8714082.

1974: Puder L, Roth S, Krabusch P, Wiegand S, Opitz R, Bald M, Flück C, Schulz E, Voss E, Markó L, Linz P, Berger F, Müller DN, Kuehne T, Litt MJ, Cone RD, Kühnen P, Kelm M. Cardiac Phenotype and Tissue Sodium Content in Adolescents With Defects in the Melanocortin System. J Clin Endocrinol Metab. 2021 Aug 18;106(9):2606-2616. doi: 10.1210/clinem/dgab368. PMID: 34036349; PMCID: PMC8372645.

1975: Mujahid MS, Gao X, Tabb LP, Morris C, Lewis TT. Historical redlining and cardiovascular health: The Multi-Ethnic Study of Atherosclerosis. Proc Natl Acad Sci U S A. 2021 Dec 21;118(51):e2110986118. doi: 10.1073/pnas.2110986118. PMID: 34903653; PMCID: PMC8713797.

1976: Calandra-Buonaura G, Alfonsi E, Vignatelli L, Benarroch EE, Giannini G, Iranzo A, Low PA, Martinelli P, Provini F, Quinn N, Tolosa E, Wenning GK, Abbruzzese G, Bower P, Antonini A, Bhatia KP, Bonavita J, Pellecchia MT, Pizzorni N, Tison F, Ghorayeb I, Meissner WG, Ozawa T, Pacchetti C, Pozzi NG, Vicini C, Schindler A, Cortelli P, Kaufmann H. Dysphagia in multiple system atrophy consensus statement on diagnosis, prognosis and treatment. Parkinsonism Relat Disord. 2021 May;86:124-132. doi: 10.1016/j.parkreldis.2021.03.027. Epub

2021 Mar 30. PMID: 33839029.

1977: Allaband C, Lingaraju A, Martino C, Russell B, Tripathi A, Poulsen O, Dantas Machado AC, Zhou D, Xue J, Elijah E, Malhotra A, Dorrestein PC, Knight R, Haddad GG, Zarrinpar A. Intermittent Hypoxia and Hypercapnia Alter Diurnal Rhythms of Luminal Gut Microbiome and Metabolome. mSystems. 2021 Jun 29;6(3):e0011621. doi: 10.1128/mSystems.00116-21. Epub ahead of print. PMID: 34184915; PMCID: PMC8269208.

1978: League GP, Degner EC, Pitcher SA, Hafezi Y, Tennant E, Cruz PC, Krishnan RS, Garcia Castillo SS, Alfonso-Parra C, Avila FW, Wolfner MF, Harrington LC. The impact of mating and sugar feeding on blood-feeding physiology and behavior in the arbovirus vector mosquito Aedes aegypti. PLoS Negl Trop Dis. 2021 Sep 30;15(9):e0009815. doi: 10.1371/journal.pntd.0009815. PMID: 34591860; PMCID: PMC8509887.

1979: Murray B, Kerfoot E, Chen L, Deng J, Graham MS, Sudre CH, Molteni E, Canas LS, Antonelli M, Klaser K, Visconti A, Hammers A, Chan AT, Franks PW, Davies R, Wolf J, Spector TD, Steves CJ, Modat M, Ourselin S. Accessible data curation and analytics for international-scale citizen science datasets. Sci Data. 2021 Nov 22;8(1):297. doi: 10.1038/s41597-021-01071-x. PMID: 34811392; PMCID: PMC8608807.

1980: Iglesias-Vázquez L, Arija V, Aranda N, Aglago EK, Cross AJ, Schulze MB, Quintana Pacheco D, Kühn T, Weiderpass E, Tumino R, Redondo-Sánchez D, de Magistris MS, Palli D, Ardanaz E, Laouali N, Sonestedt E, Drake I, Rizzolo L, Santiuste C, Sacerdote C, Quirós R, Amiano P, Agudo A, Jakszyn P. Factors associated with serum ferritin levels and iron excess: results from the EPIC-EurGast study. Eur J Nutr. 2022 Feb;61(1):101-114. doi: 10.1007/s00394-021-02625-w. Epub 2021 Jul 2. PMID: 34213605.

1981: Kvalvik LG, Klungsøyr K, Igland J, Caspersen IH, Brantsæter AL, Solberg BS, Hartman C, Schweren LJS, Larsson H, Li L, Forthun I, Johansson S, Arias Vasquez A, Haavik J. Association of sweetened carbonated beverage consumption during pregnancy and ADHD symptoms in the offspring: a study from the Norwegian Mother, Father and Child Cohort Study (MoBa). Eur J Nutr. 2022 Jan 23. doi: 10.1007/s00394-022-02798-y. Epub ahead of print. PMID: 35066701.

1982: Li DK, Smith LE, Rookyard AW, Lingam SJ, Koay YC, McEwen HP, Twigg SM, Don AS, O'Sullivan JF, Cordwell SJ, White MY. Multi-omics of a pre-clinical model of diabetic cardiomyopathy reveals increased fatty acid supply impacts mitochondrial metabolic selectivity. J Mol Cell Cardiol. 2021 Nov 24;164:92-109. doi: 10.1016/j.yjmcc.2021.11.009. Epub ahead of print. PMID: 34826416.

1983: Rodríguez-Morató J, Boronat A, Serreli G, Enríquez L, Gomez-Gomez A, Pozo OJ, Fitó M, de la Torre R. Effects of Wine and Tyrosol on the Lipid Metabolic Profile of Subjects at Risk of Cardiovascular Disease: Potential Cardioprotective Role of Ceramides. Antioxidants (Basel). 2021 Oct 25;10(11):1679. doi: 10.3390/antiox10111679. PMID: 34829550; PMCID: PMC8614856.

1984: Paz-Graniel I, Babio N, Becerra-Tomás N, Toledo E, Camacho-Barcia L, Corella D, Castañer-Niño O, Romaguera D, Vioque J, Alonso-Gómez ÁM, Wärnberg J, Martínez JA, Serra-Majem L, Estruch R, Tinahones FJ, Fernandez-Aranda F, Lapetra J, Pintó X, Tur JA, García-Rios A, Bueno-Cavanillas A, Gaforio JJ, Matía-Martín P, Daimiel L, Sánchez VM, Vidal J, Prieto-Sanchez L, Ros E, Razquin C, Mestres C, Sorli JV, Cuenca-Royo AM, Rios A, Torres-Collado L, Vaquero-Luna J, Pérez-Farinós N, Zulet MA, Sanchez-Villegas A, Casas R, Bernal-Lopez MR, Santos-Lozano JM, Corbella X, Mateos D, Buil-Cosiales P, Jiménez-Murcia S, Fernandez-Carrion R, Forcano-Gamazo L, López M, Sempere-Pascual MÁ, Moreno-Rodriguez A, Gea A, de la Torre-Fornell R, Salas-Salvadó J; PREDIMED-Plus Investigators. Association between coffee consumption and total dietary caffeine intake with cognitive functioning: cross-sectional assessment in an elderly Mediterranean population. Eur J Nutr. 2021 Aug;60(5):2381-2396. doi: 10.1007/s00394-020-02415-w. Epub 2020 Oct 30. PMID: 33125576.

1985: Bąk-Sosnowska M, Gruszczyńska M, Skrypnik D, Grzegorczyn S, Karolkiewicz J, Ratajczak M, Mądry E, Walkowiak J, Bogdański P. Type of Physical Training and Selected Aspects of Psychological Functioning of Women with Obesity: A Randomised Trial. Nutrients. 2021 Jul 26;13(8):2555. doi: 10.3390/nu13082555. PMID: 34444714; PMCID: PMC8400574.

1986: Askari G, Alikiaii B, Soleimani D, Sahebkar A, Mirjalili M, Feizi A, Iraj B, Bagherniya M. Effect of curcumin-pipeine supplementation on clinical status, mortality rate, oxidative stress, and inflammatory markers in critically ill ICU patients with COVID-19: a structured summary of a study protocol for a randomized controlled trial. Trials. 2021 Jul 6;22(1):434. doi: 10.1186/s13063-021-05372-9. PMID: 34229742; PMCID: PMC8258487.

1987: Felder M, Maushart CI, Gashi G, Senn JR, Becker AS, Müller J, Balaz M, Wolfrum C, Burger IA, Betz MJ. Fluvastatin Reduces Glucose Tolerance in Healthy Young Individuals Independently of Cold Induced BAT Activity. Front Endocrinol (Lausanne). 2021 Nov 10;12:765807. doi: 10.3389/fendo.2021.765807. PMID: 34858338; PMCID: PMC8631514.

1988: Onopiuk BM, Dąbrowska ZN, Rogalska J, Brzóska MM, Dąbrowski A, Bijowski K, Onopiuk P, Mroczko B, Orywal K, Dąbrowska E. The Beneficial Impact of the Black Chokeberry Extract against the Oxidative Stress in the Sublingual Salivary Gland of Rats Intoxicated with Cadmium. Oxid Med Cell Longev. 2021 Dec 31;2021:6622245. doi: 10.1155/2021/6622245. PMID: 35003519; PMCID: PMC8741350.

1989: Cromwell EA, Osborne JCP, Unnasch TR, Basáñez MG, Gass KM, Barbre KA, Hill E, Johnson KB, Donkers KM, Shirude S, Schmidt CA, Adekanmbi V, Adetokunboh OO, Afarideh M, Ahmadpour E, Ahmed MB, Akalu TY, Al-Aly Z, Alanezi FM, Alanzi TM, Alipour V, Andrei CL, Ansari F, Ansha MG, Anvari D, Appiah SCY, Arabloo J, Arnold BF, Ausloos M, Ayanore MA, Baig AA, Banach M, Barac A, Bärnighausen TW, Bayati M, Bhattacharyya K, Bhutta ZA, Bibi S, Bijani A, Bohlouli S, Bohluli M, Brady OJ, Bragazzi NL, Butt ZA, Carvalho F, Chatterjee S, Chattu VK, Chattu SK, Cormier NM, Dahlawi SMA, Damiani G, Daoud F, Darwesh AM, Daryani A, Deribe K, Dharmaratne SD, Diaz D, Do HT, El Sayed Zaki M, El Tantawi M, Elemineh DA, Faraj

A, Fasihi Harandi M, Fatahi Y, Feigin VL, Fernandes E, Foigt NA, Foroutan M, Franklin RC, Gubari MIM, Guido D, Guo Y, Haj-Mirzaian A, Hamagharib Abdullah K, Hamidi S, Herteliu C, Hidru HD, Higazi TB, Hossain N, Hosseinzadeh M, Househ M, Ilesanmi OS, Ilic MD, Ilic IM, Iqbal U, Irvani SSN, Jha RP, Joukar F, Jozwiak JJ, Kabir Z, Kalankesh LR, Kalhor R, Karami Matin B, Karimi SE, Kasaeian A, Kavetskyy T, Kayode GA, Kazemi Karyani A, Kelbore AG, Keramati M, Khalilov R, Khan EA, Khan MNN, Khatab K, Khater MM, Kianipour N, Kibret KT, Kim YJ, Kosen S, Krohn KJ, Kusuma D, La Vecchia C, Lansingh VC, Lee PH, LeGrand KE, Li S, Longbottom J, Magdy Abd El Razek H, Magdy Abd El Razek M, Maleki A, Mamun AA, Manafi A, Manafi N, Mansournia MA, Martins-Melo FR, Mazidi M, McAlinden C, Meharie BG, Mendoza W, Mengesha EW, Mengistu DT, Mereta ST, Mestrovic T, Miller TR, Miri M, Moghadaszadeh M, Mohammadian-Hafshejani A, Mohammadpourhodki R, Mohammed S, Mohammed S, Moradi M, Moradzadeh R, Moraga P, Mosser JF, Naderi M, Nagarajan AJ, Naik G, Negoi I, Nguyen CT, Nguyen HLT, Nguyen TH, Nikbakhsh R, Oancea B, Olagunju TO, Olagunju AT, Omar Bali A, Onwujekwe OE, Pana A, Pourjafar H, Rahim F, Rahman MHU, Rathi P, Rawaf S, Rawaf DL, Rawassizadeh R, Resnikoff S, Reta MA, Rezapour A, Rubagotti E, Rubino S, Sadeghi E, Saghafipour A, Sajadi SM, Samy AM, Sarmiento-Suárez R, Sawhney M, Schipp MF, Shaheen AA, Shaikh MA, Shamsizadeh M, Sharafi K, Sheikh A, Shetty BSK, Shin JI, Shivakumar KM, Simonetti B, Singh JA, Skiadaresi E, Soheili A, Soltani S, Spurlock EE, Sufiyan MB, Tabuchi T, Tapak L, Thompson RL, Thomson AJ, Traini E, Tran BX, Ullah I, Ullah S, Uneke CJ, Unnikrishnan B, Uthman OA, Vinkeles Melchers NVS, Violante FS, Wolde HF, Wonde TE, Yamada T, Yaya S, Yazdi-Feyzabadi V, Yip P, Yonemoto N, Yousof HSA, Yu C, Yu Y, Yusefzadeh H, Zaki L, Zaman SB, Zamanian M, Zhang ZJ, Zhang Y, Ziapour A, Hay SI, Pigott DM. Predicting the environmental suitability for onchocerciasis in Africa as an aid to elimination planning. PLoS Negl Trop Dis. 2021 Jul 28;15(7):e0008824. doi: 10.1371/journal.pntd.0008824. PMID: 34319976; PMCID: PMC8318275.

1990: Kim H, Lipsyc-Sharf M, Zong X, Wang X, Hur J, Song M, Wang M, Smith-Warner SA, Fuchs C, Ogino S, Wu K, Chan AT, Cao Y, Ng K, Giovannucci EL. Total Vitamin D Intake and Risks of Early-Onset Colorectal Cancer and Precursors. Gastroenterology. 2021 Oct;161(4):1208-1217.e9. doi: 10.1053/j.gastro.2021.07.002. Epub 2021 Jul 7. PMID: 34245763; PMCID: PMC8463427.

1991: Quek SXZ, Loo EXL, Demutska A, Chua CE, Kew GS, Wong S, Lau HX, Low EXS, Loh TL, Lung OS, Hung ECW, Rahman MM, Ghoshal UC, Wong SH, Cheung CKY, Syam AF, Tan N, Xiao Y, Liu JS, Lu F, Chen CL, Lee YY, Maralit RM, Kim YS, Oshima T, Miwa H, Pang J, Siah KTH. Impact of the coronavirus disease 2019 pandemic on irritable bowel syndrome. J Gastroenterol Hepatol. 2021 Aug;36(8):2187-2197. doi: 10.1111/jgh.15466. Epub 2021 Mar 4. PMID: 33615534; PMCID: PMC8014795.

1992: Kragsnaes MS, Kjeldsen J, Horn HC, Munk HL, Pedersen JK, Just SA, Ahlquist P, Pedersen FM, de Wit M, Möller S, Andersen V, Kristiansen K, Kinggaard Holm D, Holt HM, Christensen R, Ellingsen T. Safety and efficacy of faecal microbiota transplantation for active peripheral psoriatic arthritis: an exploratory randomised placebo-controlled trial. Ann Rheum Dis. 2021 Sep;80(9):1158-1167. doi: 10.1136/annrheumdis-2020-219511. Epub 2021 Apr 29. PMID: 33926922.

1993: Leyrolle Q, Cserjesi R, Demeure R, Neyrinck AM, Amadieu C, Rodriguez J, Kärkkäinen O, Hanhineva K, Paquot N, Cnop M, Cani PD, Thissen JP, Bindels LB, Klein O, Luminet O, Delzenne NM. Microbiota and Metabolite Profiling as Markers of Mood Disorders: A Cross-Sectional Study in Obese Patients. Nutrients. 2021 Dec 29;14(1):147. doi: 10.3390/nu14010147. PMID: 35011021; PMCID: PMC8746987.

1994: Global Burden of Disease 2019 Cancer Collaboration, Kocarnik JM, Compton K, Dean FE, Fu W, Gaw BL, Harvey JD, Henrikson HJ, Lu D, Pennini A, Xu R, Ababneh E, Abbasi-Kangevari M, Abbastabar H, Abd-Elsalam SM, Abdoli A, Abedi A, Abidi H, Abolhassani H, Adedeji IA, Adnani QES, Advani SM, Afzal MS, Aghaali M, Ahinkorah BO, Ahmad S, Ahmad T, Ahmadi A, Ahmadi S, Ahmed Rashid T, Ahmed Salih Y, Akalu GT, Aklilu A, Akram T, Akunna CJ, Al Hamad H, Alahdab F, Al-Aly Z, Ali S, Alimohamadi Y, Alipour V, Aljunid SM, Alkhayyat M, Almasi-Hashiani A, Almasri NA, Al-Maweri SAA, Almustanyir S, Alonso N, Alvis-Guzman N, Amu H, Anbesu EW, Ancuceanu R, Ansari F, Ansari-Moghaddam A, Antwi MH, Anvari D, Anyasodor AE, Aqeel M, Arabloo J, Arab-Zozani M, Aremu O, Ariffin H, Aripov T, Arshad M, Artaman A, Arulappan J, Asemi Z, Asghari Jafarabadi M, Ashraf T, Atorkey P, Aujayeb A, Ausloos M, Awedew AF, Ayala Quintanilla BP, Ayenew T, Azab MA, Azadnajafabad S, Azari Jafari A, Azarian G, Azzam AY, Badiye AD, Bahadory S, Baig AA, Baker JL, Balakrishnan S, Banach M, Bärnighausen TW, Barone-Adesi F, Barra F, Barrow A, Behzadifar M, Belgaumi UI, Bezabhe WMM, Bezabih YM, Bhagat DS, Bhagavathula AS, Bhardwaj N, Bhardwaj P, Bhaskar S, Bhattacharyya K, Bhojaraja VS, Bibi S, Bijani A, Biondi A, Bisignano C, Bjørge T, Bleyer A, Blyuss O, Bolarinwa OA, Bolla SR, Braithwaite D, Brar A, Brenner H, Bustamante-Teixeira MT, Butt NS, Butt ZA, Caetano Dos Santos FL, Cao Y, Carreras G, Catalá-López F, Cembranel F, Cerin E, Cernigliaro A, Chakinala RC, Chattu SK, Chattu VK, Chaturvedi P, Chimed-Ochir O, Cho DY, Christopher DJ, Chu DT, Chung MT, Conde J, Cortés S, Cortesi PA, Costa VM, Cunha AR, Dadras O, Dagnew AB, Dahlawi SMA, Dai X, Dandona L, Dandona R, Darwesh AM, das Neves J, De la Hoz FP, Demis AB, Denova-Gutiérrez E, Dhamnetiya D, Dhimal ML, Dhimal M, Dianatinasab M, Diaz D, Djalalinia S, Do HP, Doaei S, Dorostkar F, Dos Santos Figueiredo FW, Driscoll TR, Ebrahimi H, Eftekharzadeh S, El Tantawi M, El-Abid H, Elbarazi I, Elhabashy HR, Elhadi M, El-Jaafary SI, Eshrati B, Eskandarieh S, Esmaeilzadeh F, Etemadi A, Ezzikouri S, Faisaluddin M, Faraon EJA, Fares J, Farzadfar F, Feroze AH, Ferrero S, Ferro Desideri L, Filip I, Fischer F, Fisher JL, Foroutan M, Fukumoto T, Gaal PA, Gad MM, Gadanya MA, Gallus S, Gaspar Fonseca M, Getachew Obsa A, Ghafourifard M, Ghashghaee A, Ghith N, Gholamalizadeh M, Gilani SA, Ginindza TG, Gizaw ATT, Glasbey JC, Golechha M, Goleij P, Gomez RS, Gopalani SV, Gorini G, Goudarzi H, Grosso G, Gubari MIM, Guerra MR, Guha A, Gunasekera DS, Gupta B, Gupta VB, Gupta VK, Gutiérrez RA, Hafezi-Nejad N, Haider MR, Haj-Mirzaian A, Halwani R, Hamadeh RR, Hameed S, Hamidi S, Hanif A, Haque S, Harlianto NI, Haro JM, Hasaballah AI, Hassanipour S, Hay RJ, Hay SI, Hayat K, Heidari G, Heidari M, Herrera-Serna BY, Herteliu C, Hezam K, Holla R, Hossain MM, Hossain MBH, Hosseini MS, Hosseini M, Hosseinzadeh M, Hostiuc M, Hostiuc S, Househ M, Hsairi M, Huang J, Hugo FN, Hussain R, Hussein NR, Hwang BF, Iavicoli I, Ibitoye SE, Ida F, Ikuta KS, Ilesanmi OS, Ilic IM, Ilic MD, Irham LM, Islam JY, Islam RM, Islam SMS, Ismail NE, Isola G, Iwagami M, Jacob L, Jain V, Jakovljevic MB, Javaheri T, Jayaram S, Jazayeri SB, Jha RP, Jonas JB, Joo T, Joseph N, Joukar F,

Jürisson M, Kabir A, Kahrizi D, Kalankesh LR, Kalhor R, Kaliyadan F, Kalkonde Y, Kamath A, Kameran Al-Salihi N, Kandel H, Kapoor N, Karch A, Kasa AS, Katikireddi SV, Kauppila JH, Kavetskyy T, Kebede SA, Keshavarz P, Keykhaei M, Khader YS, Khalilov R, Khan G, Khan M, Khan MN, Khan MAB, Khang YH, Khater AM, Khayamzadeh M, Kim GR, Kim YJ, Kisa A, Kisa S, Kissimova-Skarbek K, Kopec JA, Koteeswaran R, Koul PA, Koulmane Laxminarayana SL, Koyanagi A, Kucuk Bicer B, Kugbey N, Kumar GA, Kumar N, Kumar N, Kurmi OP, Kutluk T, La Vecchia C, Lami FH, Landires I, Lauriola P, Lee SW, Lee SWH, Lee WC, Lee YH, Leigh J, Leong E, Li J, Li MC, Liu X, Loureiro JA, Lunevicius R, Magdy Abd El Razek M, Majeed A, Makki A, Male S, Malik AA, Mansournia MA, Martini S, Masoumi SZ, Mathur P, McKee M, Mehrotra R, Mendoza W, Menezes RG, Mengesha EW, Mesregah MK, Mestrovic T, Miao Jonasson J, Miazgowski B, Miazgowski T, Michalek IM, Miller TR, Mirzaei H, Mirzaei HR, Misra S, Mithra P, Moghadaszadeh M, Mohammad KA, Mohammad Y, Mohammadi M, Mohammadi SM, Mohammadian-Hafshejani A, Mohammed S, Moka N, Mokdad AH, Molokhia M, Monasta L, Moni MA, Moosavi MA, Moradi Y, Moraga P, Morgado-da-Costa J, Morrison SD, Mosapour A, Mubarik S, Mwanri L, Nagarajan AJ, Nagaraju SP, Nagata C, Naimzada MD, Nangia V, Naqvi AA, Narasimha Swamy S, Ndejjo R, Nduaguba SO, Negoi I, Negru SM, Neupane Kandel S, Nguyen CT, Nguyen HLT, Niazi RK, Nnaji CA, Noor NM, Nuñez-Samudio V, Nzoputam CI, Oancea B, Ochir C, Odukoya OO, Ogbo FA, Olagunju AT, Olakunde BO, Omar E, Omar Bali A, Omonisi AEE, Ong S, Onwujekwe OE, Orru H, Ortega-Altamirano DV, Otstavnov N, Otstavnov SS, Owolabi MO, P A M, Padubidri JR, Pakshir K, Pana A, Panagiotakos D, Panda-Jonas S, Pardhan S, Park EC, Park EK, Pashazadeh Kan F, Patel HK, Patel JR, Pati S, Pattanshetty SM, Paudel U, Pereira DM, Pereira RB, Perianayagam A, Pillay JD, Pirouzpanah S, Pishgar F, Podder I, Postma MJ, Pourjafar H, Prashant A, Preotescu L, Rabiee M, Rabiee N, Radfar A, Radhakrishnan RA, Radhakrishnan V, Rafiee A, Rahim F, Rahimzadeh S, Rahman M, Rahman MA, Rahmani AM, Rajai N, Rajesh A, Rakovac I, Ram P, Ramezanzadeh K, Ranabhat K, Ranasinghe P, Rao CR, Rao SJ, Rawassizadeh R, Razeghinia MS, Renzaho AMN, Rezaei N, Rezaei N, Rezapour A, Roberts TJ, Rodriguez JAB, Rohloff P, Romoli M, Ronfani L, Roshandel G, Rwegerera GM, S M, Sabour S, Saddik B, Saeed U, Sahebkar A, Sahoo H, Salehi S, Salem MR, Salimzadeh H, Samaei M, Samy AM, Sanabria J, Sankararaman S, Santric-Milicevic MM, Sardiwalla Y, Sarveazad A, Sathian B, Sawhney M, Saylan M, Schneider IJC, Sekerija M, Seylani A, Shafaat O, Shaghaghi Z, Shaikh MA, Shamsoddin E, Shannawaz M, Sharma R, Sheikh A, Sheikhbahaei S, Shetty A, Shetty JK, Shetty PH, Shibuya K, Shirkoohi R, Shivakumar KM, Shivarov V, Siabani S, Siddappa Malleshappa SK, Silva DAS, Singh JA, Sintayehu Y, Skryabin VY, Skryabina AA, Soeberg MJ, Sofi-Mahmudi A, Sotoudeh H, Steiropoulos P, Straif K, Subedi R, Sufiyan MB, Sultan I, Sultana S, Sur D, Szerencsés V, Szócska M, Tabarés-Seisdedos R, Tabuchi T, Tadbiri H, Taherkhani A, Takahashi K, Talaat IM, Tan KK, Tat VY, Tedla BAA, Tefera YG, Tehrani-Banihashemi A, Temsah MH, Tesfay FH, Tessema GA, Thapar R, Thavamani A, Thoguluva Chandrasekar V, Thomas N, Tohidinik HR, Touvier M, Tovani-Palone MR, Traini E, Tran BX, Tran KB, Tran MTN, Tripathy JP, Tusa BS, Ullah I, Ullah S, Umapathi KK, Unnikrishnan B, Upadhyay E, Vacante M, Vaezi M, Valadan Tahbaz S, Velazquez DZ, Veroux M, Violante FS, Vlassov V, Vo B, Volovici V, Vu GT, Waheed Y, Wamai RG, Ward P, Wen YF, Westerman R, Winkler AS, Yadav L, Yahyazadeh Jabbari SH, Yang L, Yaya S, Yazie TSY, Yeshaw Y, Yonemoto N, Younis MZ, Yousefi Z, Yu C, Yuce D, Yunusa I, Zadnik V, Zare F, Zastrozhin MS, Zastrozhina A, Zhang J, Zhong C, Zhou L, Zhu C, Ziapour A,

Zimmermann IR, Fitzmaurice C, Murray CJL, Force LM. Cancer Incidence, Mortality, Years of Life Lost, Years Lived With Disability, and Disability-Adjusted Life Years for 29 Cancer Groups From 2010 to 2019: A Systematic Analysis for the Global Burden of Disease Study 2019. JAMA Oncol. 2021 Dec 30:e216987. doi: 10.1001/jamaoncol.2021.6987. Epub ahead of print. PMID: 34967848; PMCID: PMC8719276.

1995: Benjamin EJ, Al-Khatib SM, Desvigne-Nickens P, Alonso A, Djoussé L, Forman DE, Gillis AM, Hendriks JML, Hills MT, Kirchhof P, Link MS, Marcus GM, Mehra R, Murray KT, Parkash R, Piña IL, Redline S, Rienstra M, Sanders P, Somers VK, Van Wagoner DR, Wang PJ, Cooper LS, Go AS. Research Priorities in the Secondary Prevention of Atrial Fibrillation: A National Heart, Lung, and Blood Institute Virtual Workshop Report. J Am Heart Assoc. 2021 Aug 17;10(16):e021566. doi: 10.1161/JAHA.121.021566. Epub 2021 Aug 5. PMID: 34351783; PMCID: PMC8475065.

1996: Stepien M, Lopez-Nogueroles M, Lahoz A, Kühn T, Perlemuter G, Voican C, Ciocan D, Boutron-Ruault MC, Jansen E, Viallon V, Leitzmann M, Tjønneland A, Severi G, Mancini FR, Dong C, Kaaks R, Fortner RT, Bergmann MM, Boeing H, Trichopoulou A, Karakatsani A, Peppa E, Palli D, Krogh V, Tumino R, Sacerdote C, Panico S, Bueno-de-Mesquita HB, Skeie G, Merino S, Ros RZ, Sánchez MJ, Amiano P, Huerta JM, Barricarte A, Sjöberg K, Ohlsson B, Nyström H, Werner M, Perez-Cornago A, Schmidt JA, Freisling H, Scalbert A, Weiderpass E, Christakoudi S, Gunter MJ, Jenab M. Prediagnostic alterations in circulating bile acid profiles in the development of hepatocellular carcinoma. Int J Cancer. 2021 Nov 29. doi: 10.1002/ijc.33885. Epub ahead of print. PMID: 34843121.

1997: Martelli A, Piragine E, Gorica E, Citi V, Testai L, Pagnotta E, Lazzeri L, Pecchioni N, Ciccone V, Montanaro R, Di Cesare Mannelli L, Ghelardini C, Brancaleone V, Morbidelli L, Calderone V. The H₂S-Donor Erucin Exhibits Protective Effects against Vascular Inflammation in Human Endothelial and Smooth Muscle Cells. Antioxidants (Basel). 2021 Jun 15;10(6):961. doi: 10.3390/antiox10060961. PMID: 34203803; PMCID: PMC8232611.

1998: Graham MS, Sudre CH, May A, Antonelli M, Murray B, Varsavsky T, Kläser K, Canas LS, Molteni E, Modat M, Drew DA, Nguyen LH, Polidori L, Selvachandran S, Hu C, Capdevila J; COVID-19 Genomics UK (COG-UK) Consortium, Hammers A, Chan AT, Wolf J, Spector TD, Steves CJ, Ourselin S. Changes in symptomatology, reinfection, and transmissibility associated with the SARS-CoV-2 variant B.1.1.7: an ecological study. Lancet Public Health. 2021 May;6(5):e335-e345. doi: 10.1016/S2468-2667(21)00055-4. Epub 2021 Apr 12. PMID: 33857453; PMCID: PMC8041365.
