# Supplementary material for: Perspectives of Dietary Assessment in Human Health and Disease
Source: Nutrients. 2022 Feb 16;14(4):830. doi: 10.3390/nu14040830 (PMC8877528; doi:10.3390/nu14040830)
Supplement: Supplementary file 1 [file nutrients-14-00830-s001.zip › Table S2.pdf]

Table S2 - PubMed search keywords "dietary assessment human health disease", filters “1 year” and “reviews”

starting date 08/02/2022

Type of article: Reviews

n = 222

1: Roschel H, Gualano B, Ostojic SM, Rawson ES. Creatine Supplementation and Brain Health. Nutrients. 2021 Feb 10;13(2):586. doi: 10.3390/nu13020586. PMID: 33578876; PMCID: PMC7916590.

2: Sutter DO, Bender N. Nutrient status and growth in vegan children. Nutr Res. 2021 Jul;91:13-25. doi: 10.1016/j.nutres.2021.04.005. Epub 2021 May 18. PMID: 34130207.

3: Miszta A, Huskens D, Donkervoort D, Roberts MJM, Wolberg AS, de Laat B. Assessing Plasmin Generation in Health and Disease. Int J Mol Sci. 2021 Mar 9;22(5):2758. doi: 10.3390/ijms22052758. PMID: 33803235; PMCID: PMC7963172.

4: Schuetz P, Seres D, Lobo DN, Gomes F, Kaegi-Braun N, Stanga Z. Management of disease-related malnutrition for patients being treated in hospital. Lancet. 2021 Nov 20;398(10314):1927-1938. doi: 10.1016/S0140-6736(21)01451-3. Epub 2021 Oct 14. PMID: 34656286.

5: Chintam K, Chang AR. Strategies to Treat Obesity in Patients With CKD. Am J Kidney Dis. 2021 Mar;77(3):427-439. doi: 10.1053/j.ajkd.2020.08.016. Epub 2020 Oct 16. PMID: 33075388; PMCID: PMC7904606.

6: Riegel B, Westland H, Iovino P, Barelds I, Bruins Slot J, Stawnychy MA, Osokpo O, Tarbi E, Trappenburg JCA, Vellone E, Strömberg A, Jaarsma T. Characteristics of self-care interventions for patients with a chronic condition: A scoping review. Int J Nurs Stud. 2021 Apr;116:103713. doi: 10.1016/j.ijnurstu.2020.103713. Epub 2020 Jul 15. PMID: 32768137.

7: Schulz R, Slavin J. Perspective: Defining Carbohydrate Quality for Human Health and Environmental Sustainability. Adv Nutr. 2021 Jul 30;12(4):1108-1121.

doi: 10.1093/advances/nmab050. PMID: 33951143.

8: Kistler BM, Moore LW, Benner D, Biruete A, Boaz M, Brunori G, Chen J, Drechsler C, Guebre-Egziabher F, Hensley MK, Iseki K, Kovesdy CP, Kuhlmann MK, Saxena A, Wee PT, Brown-Tortorici A, Garibotto G, Price SR, Yee-Moon Wang A, Kalantar-Zadeh K. The International Society of Renal Nutrition and Metabolism Commentary on the National Kidney Foundation and Academy of Nutrition and Dietetics KDOQI Clinical Practice Guideline for Nutrition in Chronic Kidney Disease. J Ren Nutr. 2021 Mar;31(2):116-120.e1. doi: 10.1053/j.jrn.2020.05.002. Epub 2020 Jul 29. PMID: 32737016; PMCID: PMC8045140.

9: Petersen KS, Kris-Etherton PM. Diet Quality Assessment and the Relationship between Diet Quality and Cardiovascular Disease Risk. Nutrients. 2021 Nov 28;13(12):4305. doi: 10.3390/nu13124305. PMID: 34959857; PMCID: PMC8706326.

10: Marx W, Veronese N, Kelly JT, Smith L, Hockey M, Collins S, Trakman GL, Hoare E, Teasdale SB, Wade A, Lane M, Aslam H, Davis JA, O'Neil A, Shivappa N, Hebert JR, Blekkenhorst LC, Berk M, Segasby T, Jacka F. The Dietary Inflammatory Index and Human Health: An Umbrella Review of Meta-Analyses of Observational Studies. Adv Nutr. 2021 Oct 1;12(5):1681-1690. doi: 10.1093/advances/nmab037. PMID: 33873204; PMCID: PMC8483957.

11: Melzer TM, Manosso LM, Yau SY, Gil-Mohapel J, Brocardo PS. In Pursuit of Healthy Aging: Effects of Nutrition on Brain Function. Int J Mol Sci. 2021 May 10;22(9):5026. doi: 10.3390/ijms22095026. PMID: 34068525; PMCID: PMC8126018.

12: Patikorn C, Roubal K, Veettil SK, Chandran V, Pham T, Lee YY, Giovannucci EL, Varady KA, Chaiyakunapruk N. Intermittent Fasting and Obesity-Related Health Outcomes: An Umbrella Review of Meta-analyses of Randomized Clinical Trials. JAMA Netw Open. 2021 Dec 1;4(12):e2139558. doi: 10.1001/jamanetworkopen.2021.39558. PMID: 34919135; PMCID: PMC8683964.

13: Baygi F, Mohammadi-Nasrabadi F, Zyriax BC, Jensen OC, Bygvraa DA, Oldenburg M, Nielsen JB. Global overview of dietary outcomes and dietary intake assessment

methods in maritime settings: a systematic review. BMC Public Health. 2021 Aug 21;21(1):1579. doi: 10.1186/s12889-021-11593-z. PMID: 34419000; PMCID: PMC8379789.

14: Poles J, Karhu E, McGill M, McDaniel HR, Lewis JE. The effects of twenty-four nutrients and phytonutrients on immune system function and inflammation: A narrative review. J Clin Transl Res. 2021 May 27;7(3):333-376. PMID: 34239993; PMCID: PMC8259612.

15: Montoro-Huguet MA, Santolaria-Piedrafita S, Cañamares-Orbis P, García-Erce JA. Iron Deficiency in Celiac Disease: Prevalence, Health Impact, and Clinical Management. Nutrients. 2021 Sep 28;13(10):3437. doi: 10.3390/nu13103437. PMID: 34684433; PMCID: PMC8537360.

16: Mann JFE, Chang TI, Cushman WC, Furth SL, Ix JH, Hou FF, Knoll GA, Muntner P, Pecoits-Filho R, Sarnak MJ, Tomson CRV, Craig JC, Tunnicliffe DJ, Howell M, Tonelli M, Cheung M, Earley A, Cheung AK. Commentary on the KDIGO 2021 Clinical Practice Guideline for the Management of Blood Pressure in CKD. Curr Cardiol Rep. 2021 Aug 16;23(9):132. doi: 10.1007/s11886-021-01559-3. PMID: 34398316; PMCID: PMC8366157.

17: Sharma A, Rao SSC, Kearns K, Orleck KD, Waldman SA. Review article: diagnosis, management and patient perspectives of the spectrum of constipation disorders. Aliment Pharmacol Ther. 2021 Jun;53(12):1250-1267. doi: 10.1111/apt.16369. Epub 2021 Apr 28. PMID: 33909919; PMCID: PMC8252518.

18: Bendavid I, Lobo DN, Barazzoni R, Cederholm T, Coëffier M, de van der Schueren M, Fontaine E, Hiesmayr M, Laviano A, Pichard C, Singer P. The centenary of the Harris-Benedict equations: How to assess energy requirements best? Recommendations from the ESPEN expert group. Clin Nutr. 2021 Mar;40(3):690-701. doi: 10.1016/j.clnu.2020.11.012. Epub 2020 Nov 20. PMID: 33279311.

19: Charlot A, Hutt F, Sabatier E, Zoll J. Beneficial Effects of Early Time-

Restricted Feeding on Metabolic Diseases: Importance of Aligning Food Habits with the Circadian Clock. Nutrients. 2021 Apr 22;13(5):1405. doi: 10.3390/nu13051405. PMID: 33921979; PMCID: PMC8143522.

20: Fenton S, Burrows TL, Skinner JA, Duncan MJ. The influence of sleep health on dietary intake: a systematic review and meta-analysis of intervention studies. J Hum Nutr Diet. 2021 Apr;34(2):273-285. doi: 10.1111/jhn.12813. Epub 2020 Oct 1. PMID: 33001515.

21: Pifferi F, Laurent B, Plourde M. Lipid Transport and Metabolism at the Blood-Brain Interface: Implications in Health and Disease. Front Physiol. 2021 Mar 30;12:645646. doi: 10.3389/fphys.2021.645646. PMID: 33868013; PMCID: PMC8044814.

22: Ajabnoor SM, Thorpe G, Abdelhamid A, Hooper L. Long-term effects of increasing omega-3, omega-6 and total polyunsaturated fats on inflammatory bowel disease and markers of inflammation: a systematic review and meta-analysis of randomized controlled trials. Eur J Nutr. 2021 Aug;60(5):2293-2316. doi: 10.1007/s00394-020-02413-y. Epub 2020 Oct 21. PMID: 33084958.

23: Huang S, Qi Z, Ma S, Li G, Long C, Yu Y. A critical review on human internal exposure of phthalate metabolites and the associated health risks. Environ Pollut. 2021 Jun 15;279:116941. doi: 10.1016/j.envpol.2021.116941. Epub 2021 Mar 13. PMID: 33756240.

24: Filip R, Anchidin-Norocel L, Gheorghita R, Savage WK, Dimian M. Changes in Dietary Patterns and Clinical Health Outcomes in Different Countries during the SARS-CoV-2 Pandemic. Nutrients. 2021 Oct 15;13(10):3612. doi: 10.3390/nu13103612. PMID: 34684615; PMCID: PMC8539259.

25: Fatemeh G, Sajjad M, Niloufar R, Neda S, Leila S, Khadijeh M. Effect of melatonin supplementation on sleep quality: a systematic review and meta-analysis of randomized controlled trials. J Neurol. 2022 Jan;269(1):205-216. doi: 10.1007/s00415-020-10381-w. Epub 2021 Jan 8. PMID: 33417003.

26: Peppa M, Mavroeidi I. Experimental Animal Studies Support the Role of Dietary Advanced Glycation End Products in Health and Disease. Nutrients. 2021 Sep 29;13(10):3467. doi: 10.3390/nu13103467. PMID: 34684468; PMCID: PMC8539226.

27: Porykali B, Davies A, Brooks C, Melville H, Allman-Farinelli M, Coombes J. Effects of Nutritional Interventions on Cardiovascular Disease Health Outcomes in Aboriginal and Torres Strait Islander Australians: A Scoping Review. Nutrients. 2021 Nov 15;13(11):4084. doi: 10.3390/nu13114084. PMID: 34836337; PMCID: PMC8620344.

28: Ramírez-Mejía MM, Díaz-Orozco LE, Barranco-Fragoso B, Méndez-Sánchez N. A Review of the Increasing Prevalence of Metabolic-Associated Fatty Liver Disease (MAFLD) in Children and Adolescents Worldwide and in Mexico and the Implications for Public Health. Med Sci Monit. 2021 Aug 30;27:e934134. doi: 10.12659/MSM.934134. PMID: 34456329; PMCID: PMC8415038.

29: Sobczyńska-Malefora A, Delvin E, McCaddon A, Ahmadi KR, Harrington DJ. Vitamin B₁₂ status in health and disease: a critical review. Diagnosis of deficiency and insufficiency - clinical and laboratory pitfalls. Crit Rev Clin Lab Sci. 2021 Sep;58(6):399-429. doi: 10.1080/10408363.2021.1885339. Epub 2021 Apr 21. PMID: 33881359.

30: Mailloux NA, Henegan CP, Lsoto D, Patterson KP, West PC, Foley JA, Patz JA. Climate Solutions Double as Health Interventions. Int J Environ Res Public Health. 2021 Dec 18;18(24):13339. doi: 10.3390/ijerph182413339. PMID: 34948948; PMCID: PMC8705042.

31: Knight MG, Anekwe C, Washington K, Akam EY, Wang E, Stanford FC. Weight regulation in menopause. Menopause. 2021 May 24;28(8):960-965. doi: 10.1097/GME.0000000000001792. PMID: 34033603; PMCID: PMC8373626.

32: Lecorguillé M, Teo S, Phillips CM. Maternal Dietary Quality and Dietary Inflammation Associations with Offspring Growth, Placental Development, and DNA

Methylation. Nutrients. 2021 Sep 8;13(9):3130. doi: 10.3390/nu13093130. PMID: 34579008; PMCID: PMC8468062.

33: Ennis MA, Elango R. A discussion on the 'dispensable' amino acids. Curr Opin Clin Nutr Metab Care. 2021 Sep 1;24(5):395-401. doi: 10.1097/MCO.0000000000000784. PMID: 34387624.

34: Prentice RL, Howard BV, Van Horn L, Neuhouser ML, Anderson GL, Tinker LF, Lampe JW, Raftery D, Pettinger M, Aragaki AK, Thomson CA, Mossavar-Rahmani Y, Stefanick ML, Cauley JA, Rossouw JE, Manson JE, Chlebowski RT. Nutritional epidemiology and the Women's Health Initiative: a review. Am J Clin Nutr. 2021 May 8;113(5):1083-1092. doi: 10.1093/ajcn/nqab091. PMID: 33876183; PMCID: PMC8120331.

35: Ma Z, Hummel SL, Sun N, Chen Y. From salt to hypertension, what is missed? J Clin Hypertens (Greenwich). 2021 Dec;23(12):2033-2041. doi: 10.1111/jch.14402. Epub 2021 Nov 30. PMID: 34846798; PMCID: PMC8696232.

36: Mendoza-Vasconez AS, Landry MJ, Crimarco A, Bladier C, Gardner CD. Sustainable Diets for Cardiovascular Disease Prevention and Management. Curr Atheroscler Rep. 2021 May 10;23(7):31. doi: 10.1007/s11883-021-00929-0. PMID: 33970349.

37: Khadilkar V, Shah N. Evaluation of Children and Adolescents with Obesity. Indian J Pediatr. 2021 Dec;88(12):1214-1221. doi: 10.1007/s12098-021-03893-4. Epub 2021 Aug 18. PMID: 34406593.

38: Burton-Freeman B, Freeman M, Zhang X, Sandhu A, Edirisinghe I. Watermelon and L-Citrulline in Cardio-Metabolic Health: Review of the Evidence 2000-2020. Curr Atheroscler Rep. 2021 Dec 11;23(12):81. doi: 10.1007/s11883-021-00978-5. PMID: 34894302.

39: Cox S, Sandall A, Smith L, Rossi M, Whelan K. Food additive emulsifiers: a review of their role in foods, legislation and classifications, presence in food

supply, dietary exposure, and safety assessment. Nutr Rev. 2021 May 12;79(6):726-741. doi: 10.1093/nutrit/nuaa038. PMID: 32626902.

40: Lukomskyj N, Allman-Farinelli M, Shi Y, Rangan A. Dietary exposures in childhood and adulthood and cardiometabolic outcomes: a systematic scoping review. J Hum Nutr Diet. 2021 Jun;34(3):511-523. doi: 10.1111/jhn.12841. Epub 2021 Jan 6. PMID: 33406314.

41: Poli A, Marangoni F, Corsini A, Manzato E, Marrocco W, Martini D, Medea G, Visioli F. Phytosterols, Cholesterol Control, and Cardiovascular Disease. Nutrients. 2021 Aug 16;13(8):2810. doi: 10.3390/nu13082810. PMID: 34444970; PMCID: PMC8399210.

42: Chen G. Dietary N-epsilon-carboxymethyllysine as for a major glycotoxin in foods: A review. Compr Rev Food Sci Food Saf. 2021 Sep;20(5):4931-4949. doi: 10.1111/1541-4337.12817. Epub 2021 Aug 10. PMID: 34378329.

43: Silva TR, Oppermann K, Reis FM, Spritzer PM. Nutrition in Menopausal Women: A Narrative Review. Nutrients. 2021 Jun 23;13(7):2149. doi: 10.3390/nu13072149. PMID: 34201460; PMCID: PMC8308420.

44: LeVatte M, Keshteli AH, Zarei P, Wishart DS. Applications of Metabolomics to Precision Nutrition. Lifestyle Genom. 2022;15(1):1-9. doi: 10.1159/000518489. Epub 2021 Sep 8. PMID: 34518463.

45: Baderol Allam FN, Ab Hamid MR, Buhari SS, Md Noor H. Web-Based Dietary and Physical Activity Intervention Programs for Patients With Hypertension: Scoping Review. J Med Internet Res. 2021 Mar 15;23(3):e22465. doi: 10.2196/22465. PMID: 33720036; PMCID: PMC8074856.

46: Mielech A, Puścion-Jakubik A, Socha K. Assessment of the Risk of Contamination of Food for Infants and Toddlers. Nutrients. 2021 Jul 9;13(7):2358. doi: 10.3390/nu13072358. PMID: 34371868; PMCID: PMC8308760.

47: Jahan S, Mahmud MH, Khan Z, Alam A, Khalil AA, Rauf A, Tareq AM, Nainu F, Tareq SM, Emran TB, Khan M, Khan IN, Wilairatana P, Mubarak MS. Health promoting benefits of pongamol: An overview. Biomed Pharmacother. 2021 Oct;142:112109. doi: 10.1016/j.biopha.2021.112109. Epub 2021 Aug 27. PMID: 34470730.

48: Sherf-Dagan S, Sinai T, Goldenshluger A, Globus I, Kessler Y, Schweiger C, Ben-Porat T. Nutritional Assessment and Preparation for Adult Bariatric Surgery Candidates: Clinical Practice. Adv Nutr. 2021 Jun 1;12(3):1020-1031. doi: 10.1093/advances/nmaa121. PMID: 33040143; PMCID: PMC8262552.

49: Pina A, Castelletti S. COVID-19 and Cardiovascular Disease: a Global Perspective. Curr Cardiol Rep. 2021 Aug 19;23(10):135. doi: 10.1007/s11886-021-01566-4. PMID: 34410538; PMCID: PMC8374116.

50: Potter T, Vieira R, de Roos B. Perspective: Application of N-of-1 Methods in Personalized Nutrition Research. Adv Nutr. 2021 Jun 1;12(3):579-589. doi: 10.1093/advances/nmaa173. PMID: 33460438; PMCID: PMC8166550.

51: Sharma J, Ludin H, Chauhan M, Zodpey S. Public health nutrition in Afghanistan-policies, strategies and capacity-building: current scenario and initiatives. East Mediterr Health J. 2021 Jul 29;27(7):728-737. doi: 10.26719/emhj.21.043. PMID: 34369588.

52: Rafiq T, Azab SM, Teo KK, Thabane L, Anand SS, Morrison KM, de Souza RJ, Britz-McKibbin P. Nutritional Metabolomics and the Classification of Dietary Biomarker Candidates: A Critical Review. Adv Nutr. 2021 Dec 1;12(6):2333-2357. doi: 10.1093/advances/nmab054. PMID: 34015815; PMCID: PMC8634495.

53: Aljada B, Zohni A, El-Matary W. The Gluten-Free Diet for Celiac Disease and Beyond. Nutrients. 2021 Nov 9;13(11):3993. doi: 10.3390/nu13113993. PMID: 34836247; PMCID: PMC8625243.

54: Yaseen MO, Jamshaid H, Saif A, Hussain T. Immunomodulatory role and potential utility of various nutrients and dietary components in SARS-CoV-2

infection. Int J Vitam Nutr Res. 2022 Jan;92(1):35-48. doi: 10.1024/0300-9831/a000715. Epub 2021 Jun 8. PMID: 34100300.

55: Yoder AD, Proaño GV, Handu D. Retail Nutrition Programs and Outcomes: An Evidence Analysis Center Scoping Review. J Acad Nutr Diet. 2021 Sep;121(9):1866-1880.e4. doi: 10.1016/j.jand.2020.08.080. Epub 2020 Nov 20. PMID: 33229206.

56: Aaseth J, Alexander J, Alehagen U. Coenzyme Q₁₀ supplementation - In ageing and disease. Mech Ageing Dev. 2021 Jul;197:111521. doi: 10.1016/j.mad.2021.111521. Epub 2021 Jun 12. PMID: 34129891.

57: Amonoo HL, Celano CM, Sadlonova M, Huffman JC. Is Optimism a Protective Factor for Cardiovascular Disease? Curr Cardiol Rep. 2021 Oct 1;23(11):158. doi: 10.1007/s11886-021-01590-4. PMID: 34599386.

58: Geretto M, Ferrari M, De Angelis R, Crociata F, Sebastiani N, Pulliero A, Au W, Izzotti A. Occupational Exposures and Environmental Health Hazards of Military Personnel. Int J Environ Res Public Health. 2021 May 18;18(10):5395. doi: 10.3390/ijerph18105395. PMID: 34070145; PMCID: PMC8158372.

59: Harmon KK, Stout JR, Fukuda DH, Pabian PS, Rawson ES, Stock MS. The Application of Creatine Supplementation in Medical Rehabilitation. Nutrients. 2021 May 27;13(6):1825. doi: 10.3390/nu13061825. PMID: 34071875; PMCID: PMC8230227.

60: Ponticelli C, Favi E, Ferraresso M. New-Onset Diabetes after Kidney Transplantation. Medicina (Kaunas). 2021 Mar 8;57(3):250. doi: 10.3390/medicina57030250. PMID: 33800138; PMCID: PMC7998982.

61: Ageel HK, Harrad S, Abdallah MA. Occurrence, human exposure, and risk of microplastics in the indoor environment. Environ Sci Process Impacts. 2022 Jan 26;24(1):17-31. doi: 10.1039/d1em00301a. PMID: 34842877.

62: Schultz NM, Bhardwaj S, Barclay C, Gaspar L, Schwartz J. Global Burden of Dry Age-Related Macular Degeneration: A Targeted Literature Review. Clin Ther. 2021 Oct;43(10):1792-1818. doi: 10.1016/j.clinthera.2021.08.011. Epub 2021 Sep 20. PMID: 34548176.

63: de Araújo TP, de Moraes MM, Magalhães V, Afonso C, Santos C, Rodrigues SSP. Ultra-Processed Food Availability and Noncommunicable Diseases: A Systematic Review. Int J Environ Res Public Health. 2021 Jul 10;18(14):7382. doi: 10.3390/ijerph18147382. PMID: 34299832; PMCID: PMC8306957.

64: Mehmood A, Zhao L, Wang Y, Pan F, Hao S, Zhang H, Iftikhar A, Usman M. Dietary anthocyanins as potential natural modulators for the prevention and treatment of non-alcoholic fatty liver disease: A comprehensive review. Food Res Int. 2021 Apr;142:110180. doi: 10.1016/j.foodres.2021.110180. Epub 2021 Feb 2. PMID: 33773656.

65: Marzullo P, Bettini S, Menafra D, Aprano S, Muscogiuri G, Barrea L, Savastano S, Colao A; Obesity Programs of nutrition, Education, Research and Assessment (OPERA) group. Spot-light on microbiota in obesity and cancer. Int J Obes (Lond). 2021 Nov;45(11):2291-2299. doi: 10.1038/s41366-021-00866-7. Epub 2021 Aug 6. PMID: 34363002.

66: Duarte-Hospital C, Tête A, Brial F, Benoit L, Koual M, Tomkiewicz C, Kim MJ, Blanc EB, Coumoul X, Bortoli S. Mitochondrial Dysfunction as a Hallmark of Environmental Injury. Cells. 2021 Dec 30;11(1):110. doi: 10.3390/cells11010110. PMID: 35011671; PMCID: PMC8750015.

67: Bortone I, Sardone R, Lampignano L, Castellana F, Zupo R, Lozupone M, Moretti B, Giannelli G, Panza F. How gait influences frailty models and health-related outcomes in clinical-based and population-based studies: a systematic review. J Cachexia Sarcopenia Muscle. 2021 Apr;12(2):274-297. doi: 10.1002/jcsm.12667. Epub 2021 Feb 16. PMID: 33590975; PMCID: PMC8061366.

68: O'Donovan S, Palermo C, Ryan L. Competency-based assessment in nutrition

education: A systematic literature review. J Hum Nutr Diet. 2022 Feb;35(1):102-111. doi: 10.1111/jhn.12946. Epub 2021 Sep 19. PMID: 34541713.

69: Noakes TD. Hiding unhealthy heart outcomes in a low-fat diet trial: the Women's Health Initiative Randomized Controlled Dietary Modification Trial finds that postmenopausal women with established coronary heart disease were at increased risk of an adverse outcome if they consumed a low-fat 'heart-healthy' diet. Open Heart. 2021 Jul;8(2):e001680. doi: 10.1136/openhrt-2021-001680. PMID: 34290045; PMCID: PMC8296783.

70: Castro Henríquez E. Estudio metabólico en nefrolitiasis: una herramienta subutilizada y cómo implementarla en la práctica clínica [Metabolic Stone assessment: Underused tool and how to implement it on daily practice.]. Arch Esp Urol. 2021 Nov;74(9):823-834. Spanish. PMID: 34726618.

71: Capra ME, Pederiva C, Viggiano C, De Santis R, Banderali G, Biasucci G. Nutritional Approach to Prevention and Treatment of Cardiovascular Disease in Childhood. Nutrients. 2021 Jul 10;13(7):2359. doi: 10.3390/nu13072359. PMID: 34371871; PMCID: PMC8308497.

72: Zielinski CE. Regulation of T Cell Responses by Ionic Salt Signals. Cells. 2021 Sep 9;10(9):2365. doi: 10.3390/cells10092365. PMID: 34572015; PMCID: PMC8471541.

73: Juarez D, Fruman DA. Targeting the Mevalonate Pathway in Cancer. Trends Cancer. 2021 Jun;7(6):525-540. doi: 10.1016/j.trecan.2020.11.008. Epub 2021 Jan 6. PMID: 33358111; PMCID: PMC8137523.

74: Kirschner SK, Deutz NEP, Engelen MPKJ. Intestinal dysfunction in chronic disease. Curr Opin Clin Nutr Metab Care. 2021 Sep 1;24(5):464-472. doi: 10.1097/MCO.0000000000000780. PMID: 34138768.

75: Scarallo L, Lionetti P. Dietary Management in Pediatric Patients with Crohn's Disease. Nutrients. 2021 May 11;13(5):1611. doi: 10.3390/nu13051611.

PMID: 34064976; PMCID: PMC8150738.

76: Segovia-Zafra A, Di Zeo-Sánchez DE, López-Gómez C, Pérez-Valdés Z, García-Fuentes E, Andrade RJ, Lucena MI, Villanueva-Paz M. Preclinical models of idiosyncratic drug-induced liver injury (iDILI): Moving towards prediction. Acta Pharm Sin B. 2021 Dec;11(12):3685-3726. doi: 10.1016/j.apsb.2021.11.013. Epub 2021 Nov 18. PMID: 35024301; PMCID: PMC8727925.

77: Schuppelius B, Peters B, Ottawa A, Pivovarova-Ramich O. Time Restricted Eating: A Dietary Strategy to Prevent and Treat Metabolic Disturbances. Front Endocrinol (Lausanne). 2021 Aug 12;12:683140. doi: 10.3389/fendo.2021.683140. PMID: 34456861; PMCID: PMC8387818.

78: Sangild PT, Vonderohe C, Melendez Hebib V, Burrin DG. Potential Benefits of Bovine Colostrum in Pediatric Nutrition and Health. Nutrients. 2021 Jul 26;13(8):2551. doi: 10.3390/nu13082551. PMID: 34444709; PMCID: PMC8402036.

79: Drake D, Hayden AM, Delkoski S. Love the Food That Loves You Back: A Planetary Health and Women's Heart Health Partnership. Creat Nurs. 2021 Nov 1;27(4):262-266. doi: 10.1891/cn-2021-0016. PMID: 34903630.

80: Münzel T, Sørensen M, Lelieveld J, Hahad O, Al-Kindi S, Nieuwenhuijsen M, Giles-Corti B, Daiber A, Rajagopalan S. Heart healthy cities: genetics loads the gun but the environment pulls the trigger. Eur Heart J. 2021 Jul 1;42(25):2422-2438. doi: 10.1093/eurheartj/ehab235. PMID: 34005032; PMCID: PMC8248996.

81: Brennan L, Hu FB, Sun Q. Metabolomics Meets Nutritional Epidemiology: Harnessing the Potential in Metabolomics Data. Metabolites. 2021 Oct 19;11(10):709. doi: 10.3390/metabo11100709. PMID: 34677424; PMCID: PMC8537466.

82: Shah NA, Levy CJ. Emerging technologies for the management of type 2 diabetes mellitus. J Diabetes. 2021 Sep;13(9):713-724. doi: 10.1111/1753-0407.13188. Epub 2021 May 11. PMID: 33909352.

83: D'Amico E, Grosso G, Nieves JW, Zanghì A, Factor-Litvak P, Mitsumoto H. Metabolic Abnormalities, Dietary Risk Factors and Nutritional Management in Amyotrophic Lateral Sclerosis. Nutrients. 2021 Jun 30;13(7):2273. doi: 10.3390/nu13072273. PMID: 34209133; PMCID: PMC8308334.

84: Ostojic SM. Safety of Dietary Guanidinoacetic Acid: A Villain of a Good Guy? Nutrients. 2021 Dec 24;14(1):75. doi: 10.3390/nu14010075. PMID: 35010949; PMCID: PMC8746922.

85: Javaid N, Iqbal AZ, Hameeda M. NUTRITIONAL MANAGEMENT OF LIVER CIRRHOSIS AND ITS COMPLICATIONS IN HOSPITALIZED PATIENTS. Arq Gastroenterol. 2021 Apr-Jun;58(2):246-252. doi: 10.1590/S0004-2803.202100000-43. PMID: 34287534.

86: Crivelli JJ, Maalouf NM, Paiste HJ, Wood KD, Hughes AE, Oates GR, Assimos DG. Disparities in Kidney Stone Disease: A Scoping Review. J Urol. 2021 Sep;206(3):517-525. doi: 10.1097/JU.0000000000001846. Epub 2021 Apr 27. PMID: 33904797; PMCID: PMC8355087.

87: Montanari C, Parolisi S, Borghi E, Putignani L, Bassanini G, Zuvadelli J, Bonfanti C, Tummolo A, Dionisi Vici C, Biasucci G, Burlina A, Carbone MT, Verduci E. Dysbiosis, Host Metabolism, and Non-communicable Diseases: Trialogue in the Inborn Errors of Metabolism. Front Physiol. 2021 Sep 6;12:716520. doi: 10.3389/fphys.2021.716520. PMID: 34588993; PMCID: PMC8475650.

88: Ghimire K, Mishra SR, Satheesh G, Neupane D, Sharma A, Panda R, Kallestrup P, Mclachlan CS. Salt intake and salt-reduction strategies in South Asia: From evidence to action. J Clin Hypertens (Greenwich). 2021 Oct;23(10):1815-1829. doi: 10.1111/jch.14365. Epub 2021 Sep 9. PMID: 34498797; PMCID: PMC8678780.

89: Ng SW, Colchero MA, White M. How should we evaluate sweetened beverage tax policies? A review of worldwide experience. BMC Public Health. 2021 Oct 26;21(1):1941. doi: 10.1186/s12889-021-11984-2. PMID: 34702248; PMCID: PMC8546197.

90: Cromer SJ, Yu EW. Challenges and Opportunities for Osteoporosis Care During the COVID-19 Pandemic. J Clin Endocrinol Metab. 2021 Nov 19;106(12):e4795-e4808. doi: 10.1210/clinem/dgab570. PMID: 34343287; PMCID: PMC8385842.

91: Cheng TJ, More SL, Maddaloni MA, Fung ES. Evaluation of potential gastrointestinal carcinogenicity associated with the ingestion of asbestos. Rev Environ Health. 2020 Sep 23;36(1):15-26. doi: 10.1515/reveh-2020-0061. PMID: 32966235.

92: Wedekind LE, Mitchell CM, Andersen CC, Knowler WC, Hanson RL. Epidemiology of Type 2 Diabetes in Indigenous Communities in the United States. Curr Diab Rep. 2021 Nov 22;21(11):47. doi: 10.1007/s11892-021-01406-3. PMID: 34807308; PMCID: PMC8665733.

93: Al-Dashti YA, Holt RR, Keen CL, Hackman RM. Date Palm Fruit (<i>Phoenix dactylifera</i>): Effects on Vascular Health and Future Research Directions. Int J Mol Sci. 2021 Apr 28;22(9):4665. doi: 10.3390/ijms22094665. PMID: 33925062; PMCID: PMC8125345.

94: Piccioni A, de Cunzo T, Valletta F, Covino M, Rinninella E, Raoul P, Zanza C, Mele MC, Franceschi F. Gut Microbiota and Environment in Coronary Artery Disease. Int J Environ Res Public Health. 2021 Apr 16;18(8):4242. doi: 10.3390/ijerph18084242. PMID: 33923612; PMCID: PMC8073779.

95: Zhang S, Miller DD, Li W. Non-Musculoskeletal Benefits of Vitamin D beyond the Musculoskeletal System. Int J Mol Sci. 2021 Feb 21;22(4):2128. doi: 10.3390/ijms22042128. PMID: 33669918; PMCID: PMC7924658.

96: Duplantier SC, Gardner CD. A Critical Review of the Study of Neuroprotective Diets to Reduce Cognitive Decline. Nutrients. 2021 Jun 30;13(7):2264. doi: 10.3390/nu13072264. PMID: 34208980; PMCID: PMC8308213.

97: Jiang Y, Ogunade IM, Vyas D, Adesogan AT. Aflatoxin in Dairy Cows: Toxicity,

Occurrence in Feedstuffs and Milk and Dietary Mitigation Strategies. Toxins (Basel). 2021 Apr 17;13(4):283. doi: 10.3390/toxins13040283. PMID: 33920591; PMCID: PMC8074160.

98: Holmes CJ, Racette SB. The Utility of Body Composition Assessment in Nutrition and Clinical Practice: An Overview of Current Methodology. Nutrients. 2021 Jul 22;13(8):2493. doi: 10.3390/nu13082493. PMID: 34444653; PMCID: PMC8399582.

99: Poulsen KO, Sundekilde UK. The Metabolomic Analysis of Human Milk Offers Unique Insights into Potential Child Health Benefits. Curr Nutr Rep. 2021 Mar;10(1):12-29. doi: 10.1007/s13668-020-00345-x. Epub 2021 Feb 8. PMID: 33555534.

100: Shah JM, Ramsbotham J, Seib C, Muir R, Bonner A. A scoping review of the role of health literacy in chronic kidney disease self-management. J Ren Care. 2021 Dec;47(4):221-233. doi: 10.1111/jorc.12364. Epub 2021 Feb 2. PMID: 33533199.

101: Hossenbaccus L, Linton S, Ramchandani R, Gallant MJ, Ellis AK. Insights into allergic risk factors from birth cohort studies. Ann Allergy Asthma Immunol. 2021 Sep;127(3):312-317. doi: 10.1016/j.anai.2021.04.025. Epub 2021 May 7. PMID: 33971362.

102: Fernandez-Carbonell C, Charvet LE, Krupp LB. Enhancing Mood, Cognition, and Quality of Life in Pediatric Multiple Sclerosis. Paediatr Drugs. 2021 Jul;23(4):317-329. doi: 10.1007/s40272-021-00451-5. Epub 2021 May 17. PMID: 33997945; PMCID: PMC8275506.

103: Wuni R, Kuhnle GGC, Wynn-Jones AA, Vimaleswaran KS. A Nutrigenetic Update on CETP Gene-Diet Interactions on Lipid-Related Outcomes. Curr Atheroscler Rep. 2022 Jan 31. doi: 10.1007/s11883-022-00987-y. Epub ahead of print. PMID: 35098451.

104: Duncanson K, Burns G, Pryor J, Keely S, Talley NJ. Mechanisms of Food-Induced Symptom Induction and Dietary Management in Functional Dyspepsia. Nutrients. 2021 Mar 28;13(4):1109. doi: 10.3390/nu13041109. PMID: 33800668; PMCID: PMC8066021.

105: Cheshire WP. Salt: The paradoxical philosopher's stone of autonomic medicine. Auton Neurosci. 2021 Dec;236:102895. doi: 10.1016/j.autneu.2021.102895. Epub 2021 Oct 12. PMID: 34655930.

106: Ramadas A, Tham SM, Lalani SA, Shyam S. Diet Quality of Malaysians across Lifespan: A Scoping Review of Evidence in a Multi-Ethnic Population. Nutrients. 2021 Apr 20;13(4):1380. doi: 10.3390/nu13041380. PMID: 33924050; PMCID: PMC8074191.

107: Russell C, Grimes C, Baker P, Sievert K, Lawrence MA. The drivers, trends and dietary impacts of non-nutritive sweeteners in the food supply: a narrative review. Nutr Res Rev. 2021 Dec;34(2):185-208. doi: 10.1017/S0954422420000268. Epub 2020 Nov 5. PMID: 33148371.

108: Tessitore M, Sorrentino E, Schiano Di Cola G, Colucci A, Vajro P, Mandato C. Malnutrition in Pediatric Chronic Cholestatic Disease: An Up-to-Date Overview. Nutrients. 2021 Aug 13;13(8):2785. doi: 10.3390/nu13082785. PMID: 34444944; PMCID: PMC8400766.

109: Parveen S. Impact of calorie restriction and intermittent fasting on periodontal health. Periodontol 2000. 2021 Oct;87(1):315-324. doi: 10.1111/prd.12400. PMID: 34463980.

110: Ashaolu TJ, Ashaolu JO, Adeyeye SAO. Fermentation of prebiotics by human colonic microbiota in vitro and short-chain fatty acids production: a critical review. J Appl Microbiol. 2021 Mar;130(3):677-687. doi: 10.1111/jam.14843. Epub 2020 Sep 20. PMID: 32892434.

111: Mitra S, Rauf A, Tareq AM, Jahan S, Emran TB, Shahriar TG, Dhama K,

Alhumaydhi FA, Aljohani ASM, Rebezov M, Uddin MS, Jeandet P, Shah ZA, Shariati MA, Rengasamy KR. Potential health benefits of carotenoid lutein: An updated review. Food Chem Toxicol. 2021 Aug;154:112328. doi: 10.1016/j.fct.2021.112328. Epub 2021 Jun 8. PMID: 34111488.

112: Norman K, Haß U, Pirlich M. Malnutrition in Older Adults-Recent Advances and Remaining Challenges. Nutrients. 2021 Aug 12;13(8):2764. doi: 10.3390/nu13082764. PMID: 34444924; PMCID: PMC8399049.

113: Ng C, Major G, Smyth AR. Timing of pancreatic enzyme replacement therapy (PERT) in cystic fibrosis. Cochrane Database Syst Rev. 2021 Aug 2;8(8):CD013488. doi: 10.1002/14651858.CD013488.pub2. PMID: 34339047; PMCID: PMC8406465.

114: DiVito B, Talavlikar R, Seifu S. Common Hematologic, Nutritional, Asthma/Allergic Conditions and Lead Screening/Management. Prim Care. 2021 Mar;48(1):67-81. doi: 10.1016/j.pop.2020.10.002. Epub 2020 Nov 27. PMID: 33516425.

115: Zhang LX, Li CX, Kakar MU, Khan MS, Wu PF, Amir RM, Dai DF, Naveed M, Li QY, Saeed M, Shen JQ, Rajput SA, Li JH. Resveratrol (RV): A pharmacological review and call for further research. Biomed Pharmacother. 2021 Nov;143:112164. doi: 10.1016/j.biopha.2021.112164. Epub 2021 Oct 2. PMID: 34649335.

116: Hill Gallant KM, Vorland CJ. Intestinal phosphorus absorption: recent findings in translational and clinical research. Curr Opin Nephrol Hypertens. 2021 Jul 1;30(4):404-410. doi: 10.1097/MNH.0000000000000719. PMID: 34027902; PMCID: PMC8153371.

117: Kim IS, Hwang CW, Yang WS, Kim CH. Current Perspectives on the Physiological Activities of Fermented Soybean-Derived Cheonggukjang. Int J Mol Sci. 2021 May 27;22(11):5746. doi: 10.3390/ijms22115746. PMID: 34072216; PMCID: PMC8198423.

118: Salminen AV, Silvani A, Allen RP, Clemens S, Garcia-Borreguero D, Ghorayeb

I, Ferré S, Li Y, Ondo W, Picchietti DL, Rye D, Siegel JM, Winkelman JW, Manconi M; International Restless Legs Syndrome Study Group (IRLSSG). Consensus Guidelines on Rodent Models of Restless Legs Syndrome. Mov Disord. 2021 Mar;36(3):558-569. doi: 10.1002/mds.28401. Epub 2020 Dec 31. PMID: 33382140; PMCID: PMC8313425.

119: Kemp BJ, Thompson DR, Watson CJ, McGuigan K, Woodside JV, Ski CF. Effectiveness of family-based eHealth interventions in cardiovascular disease risk reduction: A systematic review. Prev Med. 2021 Aug;149:106608. doi: 10.1016/j.ypmed.2021.106608. Epub 2021 May 11. PMID: 33984372.

120: Anderson AS, Martin RM, Renehan AG, Cade J, Copson ER, Cross AJ, Grimmett C, Keaver L, King A, Riboli E, Shaw C, Saxton JM; UK NIHR Cancer and Nutrition Collaboration (Population Health Stream). Cancer survivorship, excess body fatness and weight-loss intervention-where are we in 2020? Br J Cancer. 2021 Mar;124(6):1057-1065. doi: 10.1038/s41416-020-01155-2. Epub 2020 Nov 25. PMID: 33235316; PMCID: PMC7961062.

121: Kumah E, Afriyie EK, Abuosi AA, Ankomah SE, Fusheini A, Otchere G. Influence of the Model of Care on the Outcomes of Diabetes Self-Management Education Program: A Scoping Review. J Diabetes Res. 2021 Feb 19;2021:2969243. doi: 10.1155/2021/2969243. PMID: 33688505; PMCID: PMC7914106.

122: Zarkani AA, Schikora A. Mechanisms adopted by Salmonella to colonize plant hosts. Food Microbiol. 2021 Oct;99:103833. doi: 10.1016/j.fm.2021.103833. Epub 2021 May 20. PMID: 34119117.

123: Hadrová S, Sedláková K, Křížová L, Malyugina S. Alternative and Unconventional Feeds in Dairy Diets and Their Effect on Fatty Acid Profile and Health Properties of Milk Fat. Animals (Basel). 2021 Jun 18;11(6):1817. doi: 10.3390/ani11061817. PMID: 34207160; PMCID: PMC8234496.

124: Osokpo O, Riegel B. Cultural factors influencing self-care by persons with cardiovascular disease: An integrative review. Int J Nurs Stud. 2021

Apr;116:103383. doi: 10.1016/j.ijnurstu.2019.06.014. Epub 2019 Jul 9. PMID: 31353026.

125: El Sabry MI, Stino FKR, El-Ghany WAA. Copper: benefits and risks for poultry, livestock, and fish production. Trop Anim Health Prod. 2021 Sep 29;53(5):487. doi: 10.1007/s11250-021-02915-9. PMID: 34590182.

126: Ko GJ, Kalantar-Zadeh K. How important is dietary management in chronic kidney disease progression? A role for low protein diets. Korean J Intern Med. 2021 Jul;36(4):795-806. doi: 10.3904/kjim.2021.197. Epub 2021 Jun 22. PMID: 34153180; PMCID: PMC8273814.

127: Sestini S, Paneghetti L, Lampe C, Betti G, Bond S, Bellettato CM, Maurizio S. Social and medical needs of rare metabolic patients: results from a MetabERN survey. Orphanet J Rare Dis. 2021 Aug 3;16(1):336. doi: 10.1186/s13023-021-01948-5. PMID: 34344397; PMCID: PMC8329639.

128: Manoogian ENC, Wei-Shatzel J, Panda S. Assessing temporal eating pattern in free living humans through the myCircadianClock app. Int J Obes (Lond). 2022 Jan 8. doi: 10.1038/s41366-021-01038-3. Epub ahead of print. PMID: 34997205.

129: Munjral S, Ahluwalia P, Jamthikar AD, Puvvula A, Saba L, Faa G, Singh IM, Chadha PS, Turk M, Johri AM, Khanna NN, Viskovic K, Mavrogeni S, Laird JR, Pareek G, Miner M, Sobel DW, Balestrieri A, Sfikakis PP, Tsoulfas G, Protogerou A, Misra P, Agarwal V, Kitas GD, Kolluri R, Teji J, Al-Maini M, Dhanjil SK, Sockalingam M, Saxena A, Sharma A, Rathore V, Fatemi M, Alizad A, Viswanathan V, Krishnan PK, Omerzu T, Naidu S, Nicolaides A, Suri JS. Nutrition, atherosclerosis, arterial imaging, cardiovascular risk stratification, and manifestations in COVID-19 framework: a narrative review. Front Biosci (Landmark Ed). 2021 Nov 30;26(11):1312-1339. doi: 10.52586/5026. PMID: 34856770.

130: Bye ZL, Keshavarz P, Lane GL, Vatanparast H. What Role Do Plant-Based Diets Play in Supporting the Optimal Health and Well-being of Canadians? A Scoping Review. Adv Nutr. 2021 Dec 1;12(6):2132-2146. doi: 10.1093/advances/nmab061.

PMID: 34049398; PMCID: PMC8634516.

131: Mirzaee F, Ahmadi A. Overview of the Effect of Complementary Medicine on Treating or Mitigating the Risk of Endometriosis. Rev Bras Ginecol Obstet. 2021 Dec;43(12):919-925. English. doi: 10.1055/s-0041-1735156. Epub 2021 Dec 21. PMID: 34933385.

132: Nur HA, Atoloye AT, Wengreen H, Archuleta M, Savoie-Roskos MR, Wille C, Jewkes M. A Scoping Review and Assessing the Evidence for Nutrition Education Delivery Strategies for Refugees in High-Income Countries. Adv Nutr. 2021 Dec 1;12(6):2508-2524. doi: 10.1093/advances/nmab080. PMID: 34245153; PMCID: PMC8634542.

133: Kebbe M, Sparks JR, Flanagan EW, Redman LM. Beyond weight loss: current perspectives on the impact of calorie restriction on healthspan and lifespan. Expert Rev Endocrinol Metab. 2021 May;16(3):95-108. doi: 10.1080/17446651.2021.1922077. Epub 2021 May 7. PMID: 33957841.

134: Christidis R, Lock M, Walker T, Egan M, Browne J. Concerns and priorities of Aboriginal and Torres Strait Islander peoples regarding food and nutrition: a systematic review of qualitative evidence. Int J Equity Health. 2021 Oct 7;20(1):220. doi: 10.1186/s12939-021-01551-x. PMID: 34620180; PMCID: PMC8499519.

135: Fiolet T, Mahamat-Saleh Y, Frenoy P, Kvaskoff M, Romana Mancini F. Background exposure to polychlorinated biphenyls and all-cause, cancer-specific, and cardiovascular-specific mortality: A systematic review and meta-analysis. Environ Int. 2021 Sep;154:106663. doi: 10.1016/j.envint.2021.106663. Epub 2021 May 31. PMID: 34082240.

136: Sinopoulou V, Gordon M, Akobeng AK, Gasparetto M, Sammaan M, Vasiliou J, Dovey TM. Interventions for the management of abdominal pain in Crohn's disease and inflammatory bowel disease. Cochrane Database Syst Rev. 2021 Nov 29;11(11):CD013531. doi: 10.1002/14651858.CD013531.pub2. PMID: 34844288; PMCID: PMC8629648.

137: Wehedy E, Shatat IF, Al Khodor S. The Human Microbiome in Chronic Kidney Disease: A Double-Edged Sword. Front Med (Lausanne). 2022 Jan 17;8:790783. doi: 10.3389/fmed.2021.790783. PMID: 35111779; PMCID: PMC8801809.

138: López-Moreno A, Acuña I, Torres-Sánchez A, Ruiz-Moreno Á, Cerk K, Rivas A, Suárez A, Monteoliva-Sánchez M, Aguilera M. Next Generation Probiotics for Neutralizing Obesogenic Effects: Taxa Culturing Searching Strategies. Nutrients. 2021 May 12;13(5):1617. doi: 10.3390/nu13051617. PMID: 34065873; PMCID: PMC8151043.

139: Desai D, Kandasamy S, Limbachia J, Zulyniak MA, Ritvo P, Sherifali D, Wahi G, Anand SS, de Souza RJ. Studies to Improve Perinatal Health through Diet and Lifestyle among South Asian Women Living in Canada: A Brief History and Future Research Directions. Nutrients. 2021 Aug 24;13(9):2932. doi: 10.3390/nu13092932. PMID: 34578810; PMCID: PMC8465246.

140: Zhao X, Xu X, Li X, He X, Yang Y, Zhu S. Emerging trends of technology-based dietary assessment: a perspective study. Eur J Clin Nutr. 2021 Apr;75(4):582-587. doi: 10.1038/s41430-020-00779-0. Epub 2020 Oct 20. PMID: 33082535.

141: Capozzi F, Magkos F, Fava F, Milani GP, Agostoni C, Astrup A, Saguy IS. A Multidisciplinary Perspective of Ultra-Processed Foods and Associated Food Processing Technologies: A View of the Sustainable Road Ahead. Nutrients. 2021 Nov 5;13(11):3948. doi: 10.3390/nu13113948. PMID: 34836203; PMCID: PMC8619086.

142: Padhani ZA, Moazzam Z, Ashraf A, Bilal H, Salam RA, Das JK, Bhutta ZA. Vitamin C supplementation for prevention and treatment of pneumonia. Cochrane Database Syst Rev. 2021 Nov 18;11(11):CD013134. doi: 10.1002/14651858.CD013134.pub3. PMID: 34791642; PMCID: PMC8599445.

143: Penhaligan J, Poppitt SD, Miles-Chan JL. The Role of Bovine and Non-Bovine Milk in Cardiometabolic Health: Should We Raise the "Baa"? Nutrients. 2022 Jan

11;14(2):290. doi: 10.3390/nu14020290. PMID: 35057470; PMCID: PMC8780791.

144: Venthodika A, Chhikara N, Mann S, Garg MK, Sofi SA, Panghal A. Bioactive compounds of Aegle marmelos L., medicinal values and its food applications: A critical review. Phytother Res. 2021 Apr;35(4):1887-1907. doi: 10.1002/ptr.6934. Epub 2020 Nov 6. PMID: 33159390.

145: Kris-Etherton PM, Petersen KS, Després JP, Braun L, de Ferranti SD, Furie KL, Lear SA, Lobelo F, Morris PB, Sacks FM. Special Considerations for Healthy Lifestyle Promotion Across the Life Span in Clinical Settings: A Science Advisory From the American Heart Association. Circulation. 2021 Dec 14;144(24):e515-e532. doi: 10.1161/CIR.0000000000001014. Epub 2021 Oct 25. PMID: 34689570.

146: Mozaffari H, Hosseini Z, Lafrenière J, Conklin AI. The role of dietary diversity in preventing metabolic-related outcomes: Findings from a systematic review. Obes Rev. 2021 Jun;22(6):e13174. doi: 10.1111/obr.13174. Epub 2021 Feb 21. PMID: 33615679.

147: Calandra-Buonaura G, Alfonsi E, Vignatelli L, Benarroch EE, Giannini G, Iranzo A, Low PA, Martinelli P, Provini F, Quinn N, Tolosa E, Wenning GK, Abbruzzese G, Bower P, Antonini A, Bhatia KP, Bonavita J, Pellecchia MT, Pizzorni N, Tison F, Ghorayeb I, Meissner WG, Ozawa T, Pacchetti C, Pozzi NG, Vicini C, Schindler A, Cortelli P, Kaufmann H. Dysphagia in multiple system atrophy consensus statement on diagnosis, prognosis and treatment. Parkinsonism Relat Disord. 2021 May;86:124-132. doi: 10.1016/j.parkreldis.2021.03.027. Epub 2021 Mar 30. PMID: 33839029.

148: Khazeei Tabari MA, Iranpanah A, Bahramsoltani R, Rahimi R. Flavonoids as Promising Antiviral Agents against SARS-CoV-2 Infection: A Mechanistic Review. Molecules. 2021 Jun 25;26(13):3900. doi: 10.3390/molecules26133900. PMID: 34202374; PMCID: PMC8271800.

149: Guz M, Jeleniewicz W, Malm A, Korona-Glowniak I. A Crosstalk between Diet,

Microbiome and microRNA in Epigenetic Regulation of Colorectal Cancer.
Nutrients. 2021 Jul 15;13(7):2428. doi: 10.3390/nu13072428. PMID: 34371938;
PMCID: PMC8308570.

150: Beulens JWJ, Pinho MGM, Abreu TC, den Braver NR, Lam TM, Huss A, Vlaanderen
J, Sonnenschein T, Siddiqui NZ, Yuan Z, Kerckhoffs J, Zhernakova A, Brandao Gois
MF, Vermeulen RCH. Environmental risk factors of type 2 diabetes-an exposome
approach. Diabetologia. 2022 Feb;65(2):263-274. doi: 10.1007/s00125-021-05618-w.
Epub 2021 Nov 18. PMID: 34792619.

151: Emara MH, Soliman HH, Elnadry M, Mohamed Said E, Abd-Elsalam S, Elbatae HE,
Zaher TI, Ezzeldin S Bazeed S, Abdel-Razik A, Youssef Mohamed S, Elfert A;
"Egyptian Ramadan Fasting, Liver Diseases Interest Group". Ramadan fasting and
liver diseases: A review with practice advices and recommendations. Liver Int.
2021 Mar;41(3):436-448. doi: 10.1111/liv.14775. Epub 2021 Jan 9. PMID: 33369880.

152: Lazar RM, Howard VJ, Kernan WN, Aparicio HJ, Levine DA, Viera AJ, Jordan
LC, Nyenhuis DL, Possin KL, Sorond FA, White CL; American Heart Association
Stroke Council. A Primary Care Agenda for Brain Health: A Scientific Statement
From the American Heart Association. Stroke. 2021 Jun;52(6):e295-e308. doi:
10.1161/STR.0000000000000367. Epub 2021 Mar 15. PMID: 33719523.

153: González N, Marquès M, Domingo JL. Respiratory viruses in foods and their
potential transmission through the diet: A review of the literature. Environ
Res. 2021 Apr;195:110826. doi: 10.1016/j.envres.2021.110826. Epub 2021 Jan 30.
PMID: 33529649; PMCID: PMC7963685.

154: Wiedeman AM, Panagiotopoulos C, Devlin AM. Treatment-related weight gain
and metabolic complications in children with mental health disorders: potential
role for lifestyle interventions. Appl Physiol Nutr Metab. 2021
Mar;46(3):193-204. doi: 10.1139/apnm-2020-0259. Epub 2020 Nov 23. PMID:
33226841.

155: Wood JA, Halmos EP, Taylor KM, Gibson PR. The Role of Epidemiological

Evidence from Prospective Population Studies in Shaping Dietary Approaches to Therapy in Crohn's Disease. Mol Nutr Food Res. 2021 Mar;65(5):e2000294. doi: 10.1002/mnfr.202000294. Epub 2020 Oct 12. PMID: 33006435.

156: Niknam Z, Jafari A, Golchin A, Danesh Pouya F, Nemati M, Rezaei-Tavirani M, Rasmi Y. Potential therapeutic options for COVID-19: an update on current evidence. Eur J Med Res. 2022 Jan 13;27(1):6. doi: 10.1186/s40001-021-00626-3. PMID: 35027080; PMCID: PMC8755901.

157: Schomburg L. Selenium Deficiency Due to Diet, Pregnancy, Severe Illness, or COVID-19-A Preventable Trigger for Autoimmune Disease. Int J Mol Sci. 2021 Aug 8;22(16):8532. doi: 10.3390/ijms22168532. PMID: 34445238; PMCID: PMC8395178.

158: Tsiogkas SG, Grammatikopoulou MG, Gkiouras K, Zafiriou E, Papadopoulos I, Liaskos C, Dardiotis E, Sakkas LI, Bogdanos DP. Effect of <i>Crocus sativus</i> (Saffron) Intake on Top of Standard Treatment, on Disease Outcomes and Comorbidities in Patients with Rheumatic Diseases: Synthesis without Meta-Analysis (SWiM) and Level of Adherence to the CONSORT Statement for Randomized Controlled Trials Delivering Herbal Medicine Interventions. Nutrients. 2021 Nov 27;13(12):4274. doi: 10.3390/nu13124274. PMID: 34959826; PMCID: PMC8706139.

159: Bjelakovic M, Nikolova D, Bjelakovic G, Gluud C. Vitamin D supplementation for chronic liver diseases in adults. Cochrane Database Syst Rev. 2021 Aug 25;8(8):CD011564. doi: 10.1002/14651858.CD011564.pub3. PMID: 34431511; PMCID: PMC8407054.

160: Akbarpour E, Sadjadi A, Derakhshan MH, Roshandel G, Alimohammadian M. Gastric Cancer in Iran: An Overview of Risk Factors and Preventive Measures. Arch Iran Med. 2021 Jul 1;24(7):556-567. doi: 10.34172/aim.2021.79. PMID: 34488321.

161: Cuzzubbo S, Mangsbo S, Nagarajan D, Habra K, Pockley AG, McArdle SEB. Cancer Vaccines: Adjuvant Potency, Importance of Age, Lifestyle, and Treatments. Front Immunol. 2021 Feb 17;11:615240. doi: 10.3389/fimmu.2020.615240. PMID:

33679703; PMCID: PMC7927599.

162: Schwingshackl L, Schünemann HJ, Meerpohl JJ. Improving the trustworthiness of findings from nutrition evidence syntheses: assessing risk of bias and rating the certainty of evidence. Eur J Nutr. 2021 Sep;60(6):2893-2903. doi: 10.1007/s00394-020-02464-1. Epub 2020 Dec 30. PMID: 33377996; PMCID: PMC8354882.

163: Händel MN, Rohde JF, Jacobsen R, Heitmann BL. Processed Meat Consumption and the Risk of Cancer: A Critical Evaluation of the Constraints of Current Evidence from Epidemiological Studies. Nutrients. 2021 Oct 14;13(10):3601. doi: 10.3390/nu13103601. PMID: 34684602; PMCID: PMC8537381.

164: Billich N, Maugeri I, Calligaro L, Truby H, Davidson ZE. Weight management interventions that include dietary components for young people with chronic health care needs: A systematic review. Nutr Diet. 2021 Aug 8. doi: 10.1111/1747-0080.12698. Epub ahead of print. PMID: 34369055.

165: McParland V, Wilck N. Ernährung und Hypertonie : Worauf man neben der medikamentösen Therapie achten sollte [Nutrition and hypertension : What one should pay attention to in addition to the pharmaceutical treatment]. Internist (Berl). 2021 Mar;62(3):269-276. German. doi: 10.1007/s00108-021-00988-0. Epub 2021 Feb 16. PMID: 33590293.

166: Song S, Stern Y, Gu Y. Modifiable lifestyle factors and cognitive reserve: A systematic review of current evidence. Ageing Res Rev. 2022 Feb;74:101551. doi: 10.1016/j.arr.2021.101551. Epub 2021 Dec 21. PMID: 34952208; PMCID: PMC8794051.

167: Flythe JE, Karlsson N, Sundgren A, Cordero P, Grandinetti A, Cremisi H, Rydén A. Development of a preliminary conceptual model of the patient experience of chronic kidney disease: a targeted literature review and analysis. BMC Nephrol. 2021 Jun 23;22(1):233. doi: 10.1186/s12882-021-02440-9. PMID: 34162354; PMCID: PMC8220773.

168: Włodarczyk M, Śliżewska K. Efficiency of Resistant Starch and Dextrins as Prebiotics: A Review of the Existing Evidence and Clinical Trials. Nutrients. 2021 Oct 26;13(11):3808. doi: 10.3390/nu13113808. PMID: 34836063; PMCID: PMC8621223.

169: Babashahi M, Omidvar N, Yazdizadeh B, Heidari-Beni M, Joulaei H, Narmcheshm S, Zargaraan A, Kelishadi R. Systematic review and meta-analysis of the most common processed foods consumed by Iranian children. East Mediterr Health J. 2021 Sep 21;27(9):918-930. doi: 10.26719/emhj.21.032. PMID: 34569048.

170: Baid D, Hayles E, Finkelstein EA. Return on Investment of Workplace Wellness Programs for Chronic Disease Prevention: A Systematic Review. Am J Prev Med. 2021 Aug;61(2):256-266. doi: 10.1016/j.amepre.2021.02.002. Epub 2021 May 5. PMID: 33965267.

171: Xu B, Fu J, Qiao Y, Cao J, Deehan EC, Li Z, Jin M, Wang X, Wang Y. Higher intake of microbiota-accessible carbohydrates and improved cardiometabolic risk factors: a meta-analysis and umbrella review of dietary management in patients with type 2 diabetes. Am J Clin Nutr. 2021 Jun 1;113(6):1515-1530. doi: 10.1093/ajcn/nqaa435. PMID: 33693499.

172: Adom T, De Villiers A, Puoane T, Kengne AP. A Scoping Review of Policies Related to the Prevention and Control of Overweight and Obesity in Africa. Nutrients. 2021 Nov 11;13(11):4028. doi: 10.3390/nu13114028. PMID: 34836281; PMCID: PMC8625107.

173: Guerrant RL, Bolick DT, Swann JR. Modeling Enteropathy or Diarrhea with the Top Bacterial and Protozoal Pathogens: Differential Determinants of Outcomes. ACS Infect Dis. 2021 May 14;7(5):1020-1031. doi: 10.1021/acsinfecdis.0c00831. Epub 2021 Apr 26. PMID: 33901398; PMCID: PMC8154416.

174: Wieser H, Ruiz-Carnicer Á, Segura V, Comino I, Sousa C. Challenges of Monitoring the Gluten-Free Diet Adherence in the Management and Follow-Up of Patients with Celiac Disease. Nutrients. 2021 Jun 30;13(7):2274. doi:

10.3390/nu13072274. PMID: 34209138; PMCID: PMC8308436.

175: Adriaans DJ, Dierick-van Daele AT, van Bakel MJHM, Nieuwenhuijzen GA, Teijink JA, Heesakkers FF, van Laarhoven HW. Digital Self-Management Support Tools in the Care Plan of Patients With Cancer: Review of Randomized Controlled Trials. J Med Internet Res. 2021 Jun 29;23(6):e20861. doi: 10.2196/20861. PMID: 34184997; PMCID: PMC8278296.

176: Mazzucca CB, Raineri D, Cappellano G, Chiocchetti A. How to Tackle the Relationship between Autoimmune Diseases and Diet: Well Begun Is Half-Done. Nutrients. 2021 Nov 5;13(11):3956. doi: 10.3390/nu13113956. PMID: 34836210; PMCID: PMC8620243.

177: Cormick G, Ciapponi A, Cafferata ML, Cormick MS, Belizán JM. Calcium supplementation for prevention of primary hypertension. Cochrane Database Syst Rev. 2022 Jan 11;1(1):CD010037. doi: 10.1002/14651858.CD010037.pub4. PMID: 35014026; PMCID: PMC8748265.

178: Crane MM, Halloway S, Walts ZL, Gavin KL, Moss A, Westrick JC, Appelhans BM. Behavioural interventions for CVD risk reduction for blue-collar workers: a systematic review. J Epidemiol Community Health. 2021 Dec;75(12):1236-1243. doi: 10.1136/jech-2021-216515. Epub 2021 Jul 28. PMID: 34321281; PMCID: PMC8595631.

179: Grootveld M. Evidence-Based Challenges to the Continued Recommendation and Use of Peroxidatively-Susceptible Polyunsaturated Fatty Acid-Rich Culinary Oils for High-Temperature Frying Practises: Experimental Revelations Focused on Toxic Aldehydic Lipid Oxidation Products. Front Nutr. 2022 Jan 5;8:711640. doi: 10.3389/fnut.2021.711640. PMID: 35071288; PMCID: PMC8769064.

180: Kim JA, Jang JH, Lee SY. An Updated Comprehensive Review on Vitamin A and Carotenoids in Breast Cancer: Mechanisms, Genetics, Assessment, Current Evidence, and Future Clinical Implications. Nutrients. 2021 Sep 10;13(9):3162. doi: 10.3390/nu13093162. PMID: 34579037; PMCID: PMC8465379.

181: Fraiz GM, da Conceição AR, de Souza Vilela DL, Rocha DMUP, Bressan J, Hermsdorff HHM. Can resveratrol modulate sirtuins in obesity and related diseases? A systematic review of randomized controlled trials. Eur J Nutr. 2021 Sep;60(6):2961-2977. doi: 10.1007/s00394-021-02623-y. Epub 2021 Jul 12. PMID: 34251517.

182: Baldwin C, de van der Schueren MA, Kruizenga HM, Weekes CE. Dietary advice with or without oral nutritional supplements for disease-related malnutrition in adults. Cochrane Database Syst Rev. 2021 Dec 21;12(12):CD002008. doi: 10.1002/14651858.CD002008.pub5. PMID: 34931696; PMCID: PMC8691169.

183: Smith C, Goss HR, Issartel J, Belton S. Health Literacy in Schools? A Systematic Review of Health-Related Interventions Aimed at Disadvantaged Adolescents. Children (Basel). 2021 Feb 25;8(3):176. doi: 10.3390/children8030176. PMID: 33668861; PMCID: PMC7996245.

184: Maksoud R, Balinas C, Holden S, Cabanas H, Staines D, Marshall-Gradisnik S. A systematic review of nutraceutical interventions for mitochondrial dysfunctions in myalgic encephalomyelitis/chronic fatigue syndrome. J Transl Med. 2021 Feb 17;19(1):81. doi: 10.1186/s12967-021-02742-4. PMID: 33596913; PMCID: PMC7890871.

185: Turner G, Green R, Alae-Carew C, Dangour AD. The association of dimensions of fruit and vegetable access in the retail food environment with consumption; a systematic review. Glob Food Sec. 2021 Jun;29:100528. doi: 10.1016/j.gfs.2021.100528. PMID: 34164256; PMCID: PMC8202327.

186: Witkowska AM, Waśkiewicz A, Zujko ME, Mirończuk-Chodakowska I, Cicha-Mikołajczyk A, Drygas W. Assessment of Plant Sterols in the Diet of Adult Polish Population with the Use of a Newly Developed Database. Nutrients. 2021 Aug 7;13(8):2722. doi: 10.3390/nu13082722. PMID: 34444882; PMCID: PMC8398305.

187: Kaushik A, Peralta-Alvarez F, Gupta P, Bazo-Alvarez JC, Ofori S, Bobrow K, Monyeki D, Guinto RR, Baumgartner J, Mohan S. Assessing the Policy Landscape for

Salt Reduction in South-East Asian and Latin American Countries - An Initiative Towards Developing an Easily Accessible, Integrated, Searchable Online Repository. Glob Heart. 2021 Jul 15;16(1):49. doi: 10.5334/gh.929. PMID: 34381671; PMCID: PMC8284507.

188: Ahuja V, Aronen P, Pramodkumar TA, Looker H, Chetrit A, Bloigu AH, Juutilainen A, Bianchi C, La Sala L, Anjana RM, Pradeepa R, Venkatesan U, Jebarani S, Baskar V, Fiorentino TV, Timpel P, DeFronzo RA, Ceriello A, Del Prato S, Abdul-Ghani M, Keinänen-Kiukaanniemi S, Dankner R, Bennett PH, Knowler WC, Schwarz P, Sesti G, Oka R, Mohan V, Groop L, Tuomilehto J, Ripatti S, Bergman M, Tuomi T. Accuracy of 1-Hour Plasma Glucose During the Oral Glucose Tolerance Test in Diagnosis of Type 2 Diabetes in Adults: A Meta-analysis. Diabetes Care. 2021 Apr;44(4):1062-1069. doi: 10.2337/dc20-1688. Erratum in: Diabetes Care. 2021 Apr 30;: PMID: 33741697; PMCID: PMC8578930.

189: Bondyra-Wiśniewska B, Myszkowska-Ryciak J, Harton A. Impact of Lifestyle Intervention Programs for Children and Adolescents with Overweight or Obesity on Body Weight and Selected Cardiometabolic Factors-A Systematic Review. Int J Environ Res Public Health. 2021 Feb 20;18(4):2061. doi: 10.3390/ijerph18042061. PMID: 33672502; PMCID: PMC7923753.

190: Moradi M, Sohrabi G, Golbidi M, Yarmohammadi S, Hemati N, Campbell MS, Moradi S, Kermani MAH, Farzaei MH. Effects of artichoke on blood pressure: A systematic review and meta-analysis. Complement Ther Med. 2021 Mar;57:102668. doi: 10.1016/j.ctim.2021.102668. Epub 2021 Jan 16. PMID: 33465383.

191: Nishi SK, Viguiliouk E, Blanco Mejia S, Kendall CWC, Bazinet RP, Hanley AJ, Comelli EM, Salas Salvadó J, Jenkins DJA, Sievenpiper JL. Are fatty nuts a weighty concern? A systematic review and meta-analysis and dose-response meta-regression of prospective cohorts and randomized controlled trials. Obes Rev. 2021 Nov;22(11):e13330. doi: 10.1111/obr.13330. Epub 2021 Sep 8. PMID: 34494363.

192: Patti AM, Giglio RV, Papanas N, Serban D, Stoian AP, Pafili K, Al Rasadi K, Rajagopalan K, Rizvi AA, Ciaccio M, Rizzo M. Experimental and Emerging Free

Fatty Acid Receptor Agonists for the Treatment of Type 2 Diabetes. Medicina (Kaunas). 2022 Jan 11;58(1):109. doi: 10.3390/medicina58010109. PMID: 35056417; PMCID: PMC8779029.

193: Ruys CA, van de Lagemaat M, Rotteveel J, Finken MJJ, Lafeber HN. Improving long-term health outcomes of preterm infants: how to implement the findings of nutritional intervention studies into daily clinical practice. Eur J Pediatr. 2021 Jun;180(6):1665-1673. doi: 10.1007/s00431-021-03950-2. Epub 2021 Jan 30. PMID: 33517483; PMCID: PMC8105221.

194: Sacks G, Kwon J, Vandevijvere S, Swinburn B. Benchmarking as a Public Health Strategy for Creating Healthy Food Environments: An Evaluation of the INFORMAS Initiative (2012-2020). Annu Rev Public Health. 2021 Apr 1;42:345-362. doi: 10.1146/annurev-publhealth-100919-114442. Epub 2021 Dec 22. PMID: 33351647.

195: de Winter J, Ezendam NPM, Bours MJL, Winkels RM, Weijenberg MP, Kampman E, Vissers PAJ, Mols F, Beijer S. Is sleep associated with BMI, waist circumference, and diet among long-term colorectal cancer survivors? Results from the population-based PROFILES registry. Support Care Cancer. 2021 Dec;29(12):7225-7235. doi: 10.1007/s00520-021-06393-5. Epub 2021 Jul 6. PMID: 34228174.

196: Händel MN, Jacobsen R, Thorsteinsdottir F, Keller AC, Stougaard M, Jensen CB, Moos C, Duus KS, Jensen A, Schiøler Kesmodel U, Abrahamsen B, Heitmann BL. Assessing Health Consequences of Vitamin D Fortification Utilizing a Societal Experiment Design: Methodological Lessons Learned from the D-Tect Project. Int J Environ Res Public Health. 2021 Jul 31;18(15):8136. doi: 10.3390/ijerph18158136. PMID: 34360427; PMCID: PMC8345774.

197: Lu Q, Guo P, Liu A, Ares I, Martínez-Larrañaga MR, Wang X, Anadón A, Martínez MA. The role of long noncoding RNA in lipid, cholesterol, and glucose metabolism and treatment of obesity syndrome. Med Res Rev. 2021 May;41(3):1751-1774. doi: 10.1002/med.21775. Epub 2020 Dec 24. PMID: 33368430.

198: Varsamis NA, Christou GA, Kiortsis DN. A critical review of the effects of vitamin K on glucose and lipid homeostasis: its potential role in the prevention and management of type 2 diabetes. Hormones (Athens). 2021 Sep;20(3):415-422. doi: 10.1007/s42000-020-00268-w. Epub 2021 Jan 16. PMID: 33454929.

199: Simakova AV, Chitnis N, Babkina IB, Fedorova OS, Fedotova MM, Babkin AM, Khodkevich NE. Abundance of <i>Opisthorchis felineus</i> Metacercariae in cyprinid fish in the middle Ob River basin (Tomsk region, Russia). Food Waterborne Parasitol. 2021 Feb 5;22:e00113. doi: 10.1016/j.fawpar.2021.e00113. PMID: 33681491; PMCID: PMC7930129.

200: Coelho-Júnior HJ, Trichopoulou A, Panza F. Cross-sectional and longitudinal associations between adherence to Mediterranean diet with physical performance and cognitive function in older adults: A systematic review and meta-analysis. Ageing Res Rev. 2021 Sep;70:101395. doi: 10.1016/j.arr.2021.101395. Epub 2021 Jun 19. PMID: 34153553.

201: Lafuente M, Rodríguez González-Herrero ME, Romeo Villadóniga S, Domingo JC. Antioxidant Activity and Neuroprotective Role of Docosahexaenoic Acid (DHA) Supplementation in Eye Diseases That Can Lead to Blindness: A Narrative Review. Antioxidants (Basel). 2021 Mar 5;10(3):386. doi: 10.3390/antiox10030386. PMID: 33807538; PMCID: PMC8000043.

202: Pengpid S, Peltzer K. Multiple behavioural risk factors of non-communicable diseases among adolescents in four Caribbean countries: prevalence and correlates. Int J Adolesc Med Health. 2021 Jun 18;33(6):305-312. doi: 10.1515/ijamh-2021-0021. PMID: 34142510.

203: Katz L, Tata A, Woolman M, Zarrine-Afsar A. Lipid Profiling in Cancer Diagnosis with Hand-Held Ambient Mass Spectrometry Probes: Addressing the Late-Stage Performance Concerns. Metabolites. 2021 Sep 28;11(10):660. doi: 10.3390/metabo11100660. PMID: 34677375; PMCID: PMC8537725.

204: Virgens IPA, Santana NM, Lima SCVC, Fayh APT. Can COVID-19 be a risk for

cachexia for patients during intensive care? Narrative review and nutritional recommendations. Br J Nutr. 2021 Aug 28;126(4):552-560. doi: 10.1017/S0007114520004420. Epub 2020 Nov 5. PMID: 33261670; PMCID: PMC7711335.

205: Henry Osokpo O, James R, Riegel B. Maintaining cultural identity: A systematic mixed studies review of cultural influences on the self-care of African immigrants living with non-communicable disease. J Adv Nurs. 2021 Sep;77(9):3600-3617. doi: 10.1111/jan.14804. Epub 2021 Feb 22. PMID: 33619819.

206: Schade DS, Gonzales K, Kaminsky N, Adolphe A, Shey L, Eaton RP. Resolving the Egg and Cholesterol Intake Controversy: New Clinical Insights Into Cholesterol Regulation by the Liver and Intestine. Endocr Pract. 2022 Jan;28(1):102-109. doi: 10.1016/j.eprac.2021.09.004. Epub 2021 Sep 20. PMID: 34547473.

207: Tadipatri R, Lyon K, Azadi A, Fonkem E. A view of the epidemiologic landscape: how population-based studies can lend novel insights regarding the pathophysiology of glioblastoma. Chin Clin Oncol. 2021 Aug;10(4):35. doi: 10.21037/cco.2020.02.07. Epub 2020 Apr 8. PMID: 32279523.

208: Llanaj E, Dejanovic GM, Valido E, Bano A, Gamba M, Kastrati L, Minder B, Stojic S, Voortman T, Marques-Vidal P, Stoyanov J, Metzger B, Glisic M, Kern H, Muka T. Effect of oat supplementation interventions on cardiovascular disease risk markers: a systematic review and meta-analysis of randomized controlled trials. Eur J Nutr. 2022 Jan 3. doi: 10.1007/s00394-021-02763-1. Epub ahead of print. PMID: 34977959.

209: Amiri M, Karabegović I, van Westing AC, Verkaar AJCF, Beigrezaei S, Lara M, Bramer WM, Voortman T. Whole-diet interventions and cardiovascular risk factors in postmenopausal women: A systematic review of controlled clinical trials. Maturitas. 2022 Jan;155:40-53. doi: 10.1016/j.maturitas.2021.10.001. Epub 2021 Oct 9. PMID: 34876248.

210: Rabelo ACS, Borghesi J, Noratto GD. The role of dietary polyphenols in

osteosarcoma: A possible clue about the molecular mechanisms involved in a process that is just in its infancy. J Food Biochem. 2022 Jan;46(1):e14026. doi: 10.1111/jfbc.14026. Epub 2021 Dec 7. PMID: 34873724.

211: Bergwall S, Johansson A, Sonestedt E, Acosta S. High versus low-added sugar consumption for the primary prevention of cardiovascular disease. Cochrane Database Syst Rev. 2022 Jan 5;1(1):CD013320. doi: 10.1002/14651858.CD013320.pub2. PMID: 34986271; PMCID: PMC8730703.

212: Tran DL, Gibson H, Maiorana AJ, Verrall CE, Baker DW, Clode M, Lubans DR, Zannino D, Bullock A, Ferrie S, Briody J, Simm P, Wijesekera V, D'Almeida M, Gosbell SE, Davis GM, Weintraub R, Keech AC, Puranik R, Ugander M, Justo R, Zentner D, Majumdar A, Grigg L, Coombes JS, d'Udekem Y, Morris NR, Ayer J, Celermajer DS, Cordina R. Exercise Intolerance, Benefits, and Prescription for People Living With a Fontan Circulation: The Fontan Fitness Intervention Trial (F-FIT)-Rationale and Design. Front Pediatr. 2022 Jan 6;9:799125. doi: 10.3389/fped.2021.799125. PMID: 35071139; PMCID: PMC8771702.

213: Wang Y, Gallegos JL, Haskell-Ramsay C, Lodge JK. Effects of chronic consumption of specific fruit (berries, citrus and cherries) on CVD risk factors: a systematic review and meta-analysis of randomised controlled trials. Eur J Nutr. 2021 Mar;60(2):615-639. doi: 10.1007/s00394-020-02299-w. Epub 2020 Jun 13. Erratum in: Eur J Nutr. 2021 Jan 23;: PMID: 32535781; PMCID: PMC7900084.

214: Djalalinia S, Hasani M, Asayesh H, Ejtahed HS, Malmir H, Kasaeian A, Zarei M, Baygi F, Rastad H, Mahdavi Gorabi A, Qorbani M. The effects of dietary selenium supplementation on inflammatory markers among patients with metabolic diseases: a systematic review and meta-analysis of randomized controlled trials. J Diabetes Metab Disord. 2021 Jun 9;20(1):1051-1062. doi: 10.1007/s40200-021-00821-3. PMID: 34222098; PMCID: PMC8212246.

215: Bae S, Kamynina E, Guetterman HM, Farinola AF, Caudill MA, Berry RJ, Cassano PA, Stover PJ. Provision of folic acid for reducing arsenic toxicity in arsenic-exposed children and adults. Cochrane Database Syst Rev. 2021 Oct

18;10(10):CD012649. doi: 10.1002/14651858.CD012649.pub2. PMID: 34661903; PMCID: PMC8522704.

216: Naude CE, Brand A, Schoonees A, Nguyen KA, Chaplin M, Volmink J. Low-carbohydrate versus balanced-carbohydrate diets for reducing weight and cardiovascular risk. Cochrane Database Syst Rev. 2022 Jan 28;1(1):CD013334. doi: 10.1002/14651858.CD013334.pub2. PMID: 35088407; PMCID: PMC8795871.

217: Karimi E, Bitarafan S, Mousavi SM, Zargarzadeh N, Mokhtari P, Hawkins J, Meysamie A, Koohdani F. The effect of vitamin D supplementation on fibroblast growth factor-23 in patients with chronic kidney disease: A systematic review and meta-analysis. Phytother Res. 2021 Oct;35(10):5339-5351. doi: 10.1002/ptr.7139. Epub 2021 Apr 30. PMID: 33928687.

218: Faghfouri AH, Zarezadeh M, Aghapour B, Izadi A, Rostamkhani H, Majnouni A, Abu-Zaid A, Kord Varkaneh H, Ghoreishi Z, Ostadrahimi A. Clinical efficacy of zinc supplementation in improving antioxidant defense system: A comprehensive systematic review and time-response meta-analysis of controlled clinical trials. Eur J Pharmacol. 2021 Sep 15;907:174243. doi: 10.1016/j.ejphar.2021.174243. Epub 2021 Jun 6. PMID: 34102185.

219: Yazdanpanah Z, Beigrezaei S, Mohseni-Takalloo S, Soltani S, Rajaie SH, Zohrabi T, Kaviani M, Forbes SC, Baker JS, Salehi-Abargouei A. Does exercise affect bone mineral density and content when added to a calorie-restricted diet? A systematic review and meta-analysis of controlled clinical trials. Osteoporos Int. 2022 Feb;33(2):339-354. doi: 10.1007/s00198-021-06187-9. Epub 2021 Oct 13. PMID: 34643754.

220: Moodi V, Abedi S, Esmaeilpour M, Asbaghi O, Izadi F, Shirinbakhshmasoleh M, Behrouzian M, Shahriari A, Ghaedi E, Miraghajani M. The effect of grapes/grape products on glycemic response: A systematic review and meta-analysis of randomized controlled trials. Phytother Res. 2021 Sep;35(9):5053-5067. doi: 10.1002/ptr.7135. Epub 2021 Apr 24. PMID: 33893683.

221: Hadrup N, Frederiksen M, Wedebye EB, Nikolov NG, Carøe TK, Sørli JB, Frydendall KB, Liguori B, Sejbaek CS, Wolkoff P, Flachs EM, Schlünssen V, Meyer HW, Clausen PA, Hougaard KS. Asthma-inducing potential of 28 substances in spray cleaning products-Assessed by quantitative structure activity relationship (QSAR) testing and literature review. J Appl Toxicol. 2022 Jan;42(1):130-153. doi: 10.1002/jat.4215. Epub 2021 Jul 11. PMID: 34247391.

222: Asbaghi O, Moradi S, Nezamoleslami S, Moosavian SP, Hojjati Kermani MA, Lazaridi AV, Miraghajani M. The Effects of Magnesium Supplementation on Lipid Profile Among Type 2 Diabetes Patients: a Systematic Review and Meta-analysis of Randomized Controlled Trials. Biol Trace Elem Res. 2021 Mar;199(3):861-873. doi: 10.1007/s12011-020-02209-5. Epub 2020 May 28. PMID: 32468224.
