# Supplementary material for: Perspectives of Dietary Assessment in Human Health and Disease
Source: Nutrients. 2022 Feb 16;14(4):830. doi: 10.3390/nu14040830 (PMC8877528; doi:10.3390/nu14040830)
Supplement: Supplementary file 1 [file nutrients-14-00830-s001.zip › Table S3.pdf]

Table S3 - PubMed search keywords "dietary assessment human health disease", filters "1 year" and "clinical trials"

starting date 08/02/2022

Type of article: Clinical trials

n = 200

1: Barone Gibbs B, Kline CE, Huber KA, Paley JL, Perera S. Covid-19 shelter-at-home and work, lifestyle and well-being in desk workers. Occup Med (Lond). 2021 Apr 9;71(2):86-94. doi: 10.1093/occmed/kqab011. PMID: 33598681; PMCID: PMC7928687.

2: Pasricha SR, Hasan MI, Braat S, Larson LM, Tipu SMM, Hossain SJ, Shiraji S, Baldi A, Bhuiyan MSA, Tofail F, Fisher J, Grantham-McGregor S, Simpson JA, Hamadani JD, Biggs BA. Benefits and Risks of Iron Interventions in Infants in Rural Bangladesh. N Engl J Med. 2021 Sep 9;385(11):982-995. doi: 10.1056/NEJMoa2034187. PMID: 34496174.

3: Li C, Xing C, Zhang J, Zhao H, Shi W, He B. Eight-hour time-restricted feeding improves endocrine and metabolic profiles in women with anovulatory polycystic ovary syndrome. J Transl Med. 2021 Apr 13;19(1):148. doi: 10.1186/s12967-021-02817-2. PMID: 33849562; PMCID: PMC8045367.

4: Fortier M, Castellano CA, St-Pierre V, Myette-Côté É, Langlois F, Roy M, Morin MC, Bocti C, Fulop T, Godin JP, Delannoy C, Cuenoud B, Cunnane SC. A ketogenic drink improves cognition in mild cognitive impairment: Results of a 6-month RCT. Alzheimers Dement. 2021 Mar;17(3):543-552. doi: 10.1002/alz.12206. Epub 2020 Oct 26. PMID: 33103819; PMCID: PMC8048678.

5: Keser I, Cvijetić S, Ilić A, Colić Barić I, Boschiero D, Ilich JZ. Assessment of Body Composition and Dietary Intake in Nursing-Home Residents: Could Lessons Learned from the COVID-19 Pandemic Be Used to Prevent Future Casualties in Older Individuals? Nutrients. 2021 Apr 29;13(5):1510. doi: 10.3390/nu13051510. PMID: 33947099; PMCID: PMC8146998.

6: Jimenez-Torres J, Alcalá-Diaz JF, Torres-Peña JD, Gutierrez-Mariscal FM,

Leon-Acuña A, Gómez-Luna P, Fernández-Gandara C, Quintana-Navarro GM, Fernandez-Garcia JC, Perez-Martinez P, Ordovas JM, Delgado-Lista J, Yubero-Serrano EM, Lopez-Miranda J. Mediterranean Diet Reduces Atherosclerosis Progression in Coronary Heart Disease: An Analysis of the CORDIOPREV Randomized Controlled Trial. Stroke. 2021 Nov;52(11):3440-3449. doi: 10.1161/STROKEAHA.120.033214. Epub 2021 Aug 10. Erratum in: Stroke. 2021 Nov;52(11):e754. PMID: 34372670.

7: Park S, Lee S, Kim Y, Lee Y, Kang MW, Kim K, Kim YC, Han SS, Lee H, Lee JP, Joo KW, Lim CS, Kim YS, Kim DK. Causal effects of relative fat, protein, and carbohydrate intake on chronic kidney disease: a Mendelian randomization study. Am J Clin Nutr. 2021 Apr 6;113(4):1023-1031. doi: 10.1093/ajcn/nqaa379. PMID: 33564816.

8: Amini L, Chekini R, Nateghi MR, Haghani H, Jamialahmadi T, Sathyapalan T, Sahebkar A. The Effect of Combined Vitamin C and Vitamin E Supplementation on Oxidative Stress Markers in Women with Endometriosis: A Randomized, Triple-Blind Placebo-Controlled Clinical Trial. Pain Res Manag. 2021 May 26;2021:5529741. doi: 10.1155/2021/5529741. PMID: 34122682; PMCID: PMC8172324.

9: Gotfredsen JL, Hoppe C, Andersen R, Andersen EW, Landberg R, Overvad K, Tetens I. Effects of substitution dietary guidelines targeted at prevention of IHD on dietary intake and risk factors in middle-aged Danish adults: the Diet and Prevention of Ischemic Heart Disease: a Translational Approach (DIPI) randomised controlled trial. Br J Nutr. 2021 Oct 28;126(8):1179-1193. doi: 10.1017/S0007114520005164. Epub 2020 Dec 28. PMID: 33357247.

10: Wauters L, Slaets H, De Paepe K, Ceulemans M, Wetzels S, Geboers K, Toth J, Thys W, Dybajlo R, Walgraeve D, Biessen E, Verbeke K, Tack J, Van de Wiele T, Hellings N, Vanuytsel T. Efficacy and safety of spore-forming probiotics in the treatment of functional dyspepsia: a pilot randomised, double-blind, placebo-controlled trial. Lancet Gastroenterol Hepatol. 2021 Oct;6(10):784-792. doi: 10.1016/S2468-1253(21)00226-0. Epub 2021 Aug 3. Erratum in: Lancet Gastroenterol Hepatol. 2021 Aug 27;: PMID: 34358486.

11: Li C, Luo F, Liu C, Xiong N, Xu Z, Zhang W, Yang M, Wang Y, Liu D, Yu C, Zeng J, Zhang L, Li D, Liu Y, Feng M, Liu R, Mei J, Deng S, Zeng Z, He Y, Liu H, Shi Z, Duan M, Kang D, Liao J, Li W, Liu L. Effect of a genetically engineered interferon-alpha versus traditional interferon-alpha in the treatment of moderate-to-severe COVID-19: a randomised clinical trial. Ann Med. 2021 Dec;53(1):391-401. doi: 10.1080/07853890.2021.1890329. PMID: 33620016; PMCID: PMC7906612.

12: Liu X, Morris MC, Dhana K, Ventrelle J, Johnson K, Bishop L, Hollings CS, Boulin A, Laranjo N, Stubbs BJ, Reilly X, Carey VJ, Wang Y, Furtado JD, Marcovina SM, Tangney C, Aggarwal NT, Arfanakis K, Sacks FM, Barnes LL. Mediterranean-DASH Intervention for Neurodegenerative Delay (MIND) study: Rationale, design and baseline characteristics of a randomized control trial of the MIND diet on cognitive decline. Contemp Clin Trials. 2021 Mar;102:106270. doi: 10.1016/j.cct.2021.106270. Epub 2021 Jan 9. PMID: 33434704; PMCID: PMC8042655.

13: Neeland IJ, Marso SP, Ayers CR, Lewis B, Oslica R, Francis W, Rodder S, Pandey A, Joshi PH. Effects of liraglutide on visceral and ectopic fat in adults with overweight and obesity at high cardiovascular risk: a randomised, double-blind, placebo-controlled, clinical trial. Lancet Diabetes Endocrinol. 2021 Sep;9(9):595-605. doi: 10.1016/S2213-8587(21)00179-0. Epub 2021 Aug 3. PMID: 34358471.

14: Goraya N, Munoz-Maldonado Y, Simoni J, Wesson DE. Treatment of Chronic Kidney Disease-Related Metabolic Acidosis With Fruits and Vegetables Compared to NaHCO₃ Yields More and Better Overall Health Outcomes and at Comparable Five-Year Cost. J Ren Nutr. 2021 May;31(3):239-247. doi: 10.1053/j.jrn.2020.08.001. Epub 2020 Sep 18. PMID: 32952009.

15: Weiman DI, Mahmud FH, Clarke ABM, Assor E, McDonald C, Saibil F, Lochnan HA, Punthakee Z, Marcon MA; CD-DIET Study Group. Impact of a Gluten-Free Diet on Quality of Life and Health Perception in Patients With Type 1 Diabetes and Asymptomatic Celiac Disease. J Clin Endocrinol Metab. 2021 Apr

23;106(5):e1984-e1992. doi: 10.1210/clinem/dgaa977. PMID: 33524131.

16: Xing Y, Zhao B, Yin L, Guo M, Shi H, Zhu Z, Zhang L, He J, Ling Y, Gao M, Lu H, Mao E, Zhang L. Vitamin C supplementation is necessary for patients with coronavirus disease: An ultra-high-performance liquid chromatography-tandem mass spectrometry finding. J Pharm Biomed Anal. 2021 Mar 20;196:113927. doi: 10.1016/j.jpba.2021.113927. Epub 2021 Jan 27. PMID: 33549875; PMCID: PMC7839397.

17: Kragsnaes MS, Kjeldsen J, Horn HC, Munk HL, Pedersen JK, Just SA, Ahlquist P, Pedersen FM, de Wit M, Möller S, Andersen V, Kristiansen K, Kinggaard Holm D, Holt HM, Christensen R, Ellingsen T. Safety and efficacy of faecal microbiota transplantation for active peripheral psoriatic arthritis: an exploratory randomised placebo-controlled trial. Ann Rheum Dis. 2021 Sep;80(9):1158-1167. doi: 10.1136/annrheumdis-2020-219511. Epub 2021 Apr 29. PMID: 33926922.

18: Altun E, Walther C, Borof K, Petersen E, Lieske B, Kasapoudis D, Jalilvand N, Beikler T, Jagemann B, Zyriax BC, Aarabi G. Association between Dietary Pattern and Periodontitis-A Cross-Sectional Study. Nutrients. 2021 Nov 21;13(11):4167. doi: 10.3390/nu13114167. PMID: 34836422; PMCID: PMC8621734.

19: Hochmayr C, Ndayisaba JP, Gande N, Staudt A, Bernar B, Stock K, Geiger R, Knoflach M, Kiechl-Kohlendorfer U; Early Vascular Ageing (EVA) Study Group. Prevalence and differences of ideal cardiovascular health in urban and rural adolescents in the Region of Tyrol: results from the EVA Tyrol study. BMC Cardiovasc Disord. 2021 Jul 13;21(1):338. doi: 10.1186/s12872-021-02156-6. PMID: 34256716; PMCID: PMC8276470.

20: Wright KD, Klatt MD, Adams IR, Nguyen CM, Mion LC, Tan A, Monroe TB, Rose KM, Scharre DW. Mindfulness in Motion and Dietary Approaches to Stop Hypertension (DASH) in Hypertensive African Americans. J Am Geriatr Soc. 2021 Mar;69(3):773-778. doi: 10.1111/jgs.16947. Epub 2020 Nov 23. PMID: 33227157; PMCID: PMC8329944.

21: Ko J, Wang J, Du Y, Jiwani R, Li C. Personalized Behavioral Nutrition Among

Older Asian Americans: Study Protocol. Nurs Res. 2021 Jul-Aug 01;70(4):317-322. doi: 10.1097/NNR.0000000000000514. PMID: 34160184; PMCID: PMC8231758.

22: Wu L, Lo ECM, McGrath C, Wong MCM, Ho SMY, Gao X. Motivational interviewing for caries prevention in adolescents: a randomized controlled trial. Clin Oral Investig. 2022 Jan;26(1):585-594. doi: 10.1007/s00784-021-04037-w. Epub 2021 Jul 13. PMID: 34254214.

23: Kavyani M, Saleh-Ghadimi S, Dehghan P, Abbasalizad Farhangi M, Khoshbaten M. Co-supplementation of camelina oil and a prebiotic is more effective for in improving cardiometabolic risk factors and mental health in patients with NAFLD: a randomized clinical trial. Food Funct. 2021 Sep 20;12(18):8594-8604. doi: 10.1039/d1fo00448d. PMID: 34338703.

24: Guagnano MT, D'Angelo C, Caniglia D, Di Giovanni P, Celletti E, Sabatini E, Speranza L, Bucci M, Cipollone F, Paganelli R. Improvement of Inflammation and Pain after Three Months' Exclusion Diet in Rheumatoid Arthritis Patients. Nutrients. 2021 Oct 9;13(10):3535. doi: 10.3390/nu13103535. PMID: 34684536; PMCID: PMC8539601.

25: Nishi SK, Kendall CWC, Bazinet RP, Hanley AJ, Comelli EM, Jenkins DJA, Sievenpiper JL. Almond Bioaccessibility in a Randomized Crossover Trial: Is a Calorie a Calorie? Mayo Clin Proc. 2021 Sep;96(9):2386-2397. doi: 10.1016/j.mayocp.2021.01.026. Epub 2021 Apr 11. PMID: 33853731.

26: Pham VT, Calatayud M, Rotsaert C, Seifert N, Richard N, Van den Abbeele P, Marzorati M, Steinert RE. Antioxidant Vitamins and Prebiotic FOS and XOS Differentially Shift Microbiota Composition and Function and Improve Intestinal Epithelial Barrier In Vitro. Nutrients. 2021 Mar 29;13(4):1125. doi: 10.3390/nu13041125. PMID: 33805552; PMCID: PMC8066074.

27: Majeed M, Majeed S, Nagabhushanam K. An Open-Label Pilot Study on Macumax Supplementation for Dry-Type Age-Related Macular Degeneration. J Med Food. 2021 May;24(5):551-557. doi: 10.1089/jmf.2020.0097. Epub 2020 Aug 27. PMID: 33180005;

PMCID: PMC8140349.

28: Mølmen KS, Hammarström D, Pedersen K, Lian Lie AC, Steile RB, Nygaard H, Khan Y, Hamarsland H, Koll L, Hanestadhaugen M, Eriksen AL, Grindaker E, Whist JE, Buck D, Ahmad R, Strand TA, Rønnestad BR, Ellefsen S. Vitamin D₃ supplementation does not enhance the effects of resistance training in older adults. J Cachexia Sarcopenia Muscle. 2021 Jun;12(3):599-628. doi: 10.1002/jcsm.12688. Epub 2021 Mar 31. PMID: 33788419; PMCID: PMC8200443.

29: Guazzelli Williamson V, Lee AM, Miller D, Huo T, Maner JK, Cardel M. Psychological Resilience, Experimentally Manipulated Social Status, and Dietary Intake among Adolescents. Nutrients. 2021 Mar 1;13(3):806. doi: 10.3390/nu13030806. PMID: 33804409; PMCID: PMC7998543.

30: Porper K, Shpatz Y, Plotkin L, Pechthold RG, Talianski A, Champ CE, Furman O, Shimoni-Sebag A, Symon Z, Amit U, Hemi R, Kanety H, Mardor Y, Cohen ZR, Jan E, Genssin H, Anikster Y, Zach L, Lawrence YR. A Phase I clinical trial of dose-escalated metabolic therapy combined with concomitant radiation therapy in high-grade glioma. J Neurooncol. 2021 Jul;153(3):487-496. doi: 10.1007/s11060-021-03786-8. Epub 2021 Jun 21. PMID: 34152528.

31: Basaqr R, Skleres M, Jayswal R, Thomas DT. The effect of dietary nitrate and vitamin C on endothelial function, oxidative stress and blood lipids in untreated hypercholesterolemic subjects: A randomized double-blind crossover study. Clin Nutr. 2021 Apr;40(4):1851-1860. doi: 10.1016/j.clnu.2020.10.012. Epub 2020 Oct 14. PMID: 33115598.

32: Calvo-Lerma J, Boon M, Hulst J, Colombo C, Asseiceira I, Garriga M, Masip E, Claes I, Bulfamante A, Janssens HM, Roca M, Vicente S, Fornés V, Zazzeron L, van Schijndel B, Woodcock S, Pereira L, de Boeck K, Ribes-Koninckx C. Change in Nutrient and Dietary Intake in European Children with Cystic Fibrosis after a 6-Month Intervention with a Self-Management mHealth Tool. Nutrients. 2021 May 26;13(6):1801. doi: 10.3390/nu13061801. PMID: 34073260; PMCID: PMC8229611.

33: Krapf J, Schuhbeck A, Wendel T, Fritz J, Scholl-Bürgi S, Bösmüller C, Oberhuber R, Margreiter C, Maglione M, Stättner S, Messner F, Berchtold V, Braunwarth E, Primavesi F, Cardini B, Resch T, Karall D, Öfner D, Margreiter R, Schneeberger S. Assessment of the Clinical Impact of a Liver-Specific, BCAA-Enriched Diet in Major Liver Surgery. Transplant Proc. 2021 Mar;53(2):624-629. doi: 10.1016/j.transproceed.2020.09.013. Epub 2020 Nov 1. PMID: 33139038.

34: Genton L, Teta D, Pruijm M, Stoermann C, Marangon N, Mareschal J, Bassi I, Wurzner-Ghajarzadeh A, Lazarevic V, Cynober L, Cani PD, Herrmann FR, Schrenzel J. Glycine increases fat-free mass in malnourished haemodialysis patients: a randomized double-blind crossover trial. J Cachexia Sarcopenia Muscle. 2021 Dec;12(6):1540-1552. doi: 10.1002/jcsm.12780. Epub 2021 Sep 14. PMID: 34519439; PMCID: PMC8718019.

35: Yiallourou SR, Carrington MJ. Improved sleep efficiency is associated with reduced cardio-metabolic risk: Findings from the MODERN trial. J Sleep Res. 2021 Dec;30(6):e13389. doi: 10.1111/jsr.13389. Epub 2021 Jun 2. PMID: 34080247.

36: Muscat DM, Morris GM, Bell K, Cvejic E, Smith J, Jansen J, Thomas R, Bonner C, Doust J, McCaffery K. Benefits and Harms of Hypertension and High-Normal Labels: A Randomized Experiment. Circ Cardiovasc Qual Outcomes. 2021 Apr;14(4):e007160. doi: 10.1161/CIRCOUTCOMES.120.007160. Epub 2021 Apr 5. PMID: 33813855.

37: Amerikanou C, Kanoni S, Kaliora AC, Barone A, Bjelan M, D'Auria G, Gioxari A, Gosalbes MJ, Mouchti S, Stathopoulou MG, Soriano B, Stojanoski S, Banerjee R, Halabalaki M, Mikropoulou EV, Kannt A, Lamont J, Llorens C, Marascio F, Marascio M, Roig FJ, Smyrnioudis I, Varlamis I, Visvikis-Siest S, Vukic M, Milic N, Medic-Stojanoska M, Cesarini L, Campolo J, Gastaldelli A, Deloukas P, Trivella MG, Francino MP, Dedoussis GV; MAST4HEALTH consortium. Effect of Mastiha supplementation on NAFLD: The MAST4HEALTH Randomised, Controlled Trial. Mol Nutr Food Res. 2021 May;65(10):e2001178. doi: 10.1002/mnfr.202001178. Epub 2021 Apr 16. PMID: 33629536.

38: Harris JR, Hammerback K, Brown M, Ryan DE, Coe NB, Pike KJ, Santiago PM, Hannon PA. Local Health Jurisdiction Staff Deliver Health Promotion to Small Worksites, Washington. J Public Health Manag Pract. 2021 Mar-Apr 01;27(2):117-124. doi: 10.1097/PHH.0000000000001105. PMID: 31738191; PMCID: PMC7220816.

39: Oliai Araghi S, Kiefte-de Jong JC, van Dijk SC, Swart KMA, Ploegmakers KJ, Zillikens MC, van Schoor NM, de Groot LCPGM, Lips P, Stricker BH, Uitterlinden AG, van der Velde N. Long-term effects of folic acid and vitamin-B12 supplementation on fracture risk and cardiovascular disease: Extended follow-up of the B-PROOF trial. Clin Nutr. 2021 Mar;40(3):1199-1206. doi: 10.1016/j.clnu.2020.07.033. Epub 2020 Aug 5. PMID: 32800386.

40: Schvey NA, Shank LM, Tanofsky-Kraff M, Ramirez S, Altman DR, Swanson T, Rubin AG, Kelly NR, LeMay-Russell S, Byrne ME, Parker MN, Broadney MM, Brady SM, Yanovski SZ, Yanovski JA. Weight-based teasing in youth: Associations with metabolic and inflammatory markers. Pediatr Obes. 2021 Mar;16(3):e12729. doi: 10.1111/ijpo.12729. Epub 2020 Oct 15. PMID: 33059389; PMCID: PMC8209784.

41: Miao J, Bachmann KN, Huang S, Su YR, Dusek J, Newton-Cheh C, Arora P, Wang TJ. Effects of Vitamin D Supplementation on Cardiovascular and Glycemic Biomarkers. J Am Heart Assoc. 2021 May 18;10(10):e017727. doi: 10.1161/JAHA.120.017727. Epub 2021 May 7. PMID: 33960201; PMCID: PMC8200713.

42: Baxter JB, Wasan Y, Hussain A, Soofi SB, Ahmed I, Bhutta ZA. Characterizing Micronutrient Status and Risk Factors among Late Adolescent and Young Women in Rural Pakistan: A Cross-Sectional Assessment of the MaPPS Trial. Nutrients. 2021 Apr 9;13(4):1237. doi: 10.3390/nu13041237. PMID: 33918630; PMCID: PMC8069550.

43: Sondo P, Tahita MC, Rouamba T, Derra K, Kaboré B, Compaoré CS, Ouédraogo F, Rouamba E, Ilboudo H, Bambara EA, Nana M, Sawadogo EY, Sorgho H, Somé AM, Valéa I, Dahal P, Traoré/Coulibaly M, Tinto H. Assessment of a combined strategy of seasonal malaria chemoprevention and supplementation with vitamin A, zinc and Plumpy'Doz™ to prevent malaria and malnutrition in children under 5 years old in

Burkina Faso: a randomized open-label trial (SMC-NUT). Trials. 2021 May 24;22(1):360. doi: 10.1186/s13063-021-05320-7. PMID: 34030705; PMCID: PMC8142067.

44: Bhargava A, Bhargava M, Velayutham B, Thiruvengadam K, Watson B, Kulkarni B, Singh M, Dayal R, Pathak RR, Mitra A, Rade K, Sachdeva KS. The RATIONS (Reducing Activation of Tuberculosis by Improvement of Nutritional Status) study: a cluster randomised trial of nutritional support (food rations) to reduce TB incidence in household contacts of patients with microbiologically confirmed pulmonary tuberculosis in communities with a high prevalence of undernutrition, Jharkhand, India. BMJ Open. 2021 May 20;11(5):e047210. doi: 10.1136/bmjopen-2020-047210. PMID: 34016663; PMCID: PMC8141431.

45: Boedt T, Matthys C, Lie Fong S, De Neubourg D, Vereeck S, Seghers J, Van der Gucht K, Weyn B, Geerts D, Spiessens C, Dancet EAF. Systematic development of a mobile preconception lifestyle programme for couples undergoing IVF: the PreLiFe-programme. Hum Reprod. 2021 Aug 18;36(9):2493-2505. doi: 10.1093/humrep/deab166. PMID: 34379119.

46: Chao SM, Yen M, Lin HS, Sung JM, Hung SY, Natashia D. Effects of helping relationships on health-promoting lifestyles among patients with chronic kidney disease: A randomized controlled trial. Int J Nurs Stud. 2022 Feb;126:104137. doi: 10.1016/j.ijnurstu.2021.104137. Epub 2021 Nov 22. PMID: 34890837.

47: Sandebring-Matton A, Goikolea J, Björkhem I, Paternain L, Kemppainen N, Laatikainen T, Ngandu T, Rinne J, Soininen H, Cedazo-Minguez A, Solomon A, Kivipelto M. 27-Hydroxycholesterol, cognition, and brain imaging markers in the FINGER randomized controlled trial. Alzheimers Res Ther. 2021 Mar 6;13(1):56. doi: 10.1186/s13195-021-00790-y. PMID: 33676572; PMCID: PMC7937194.

48: Miazgowski T, Kaczmarkiewicz A, Miazgowski B, Kopeć J. Cardiometabolic health, visceral fat and circulating irisin levels: results from a real-world weight loss study. J Endocrinol Invest. 2021 Jun;44(6):1243-1252. doi: 10.1007/s40618-020-01415-1. Epub 2020 Sep 6. PMID: 32892317; PMCID: PMC8124056.

49: Camacho-Barcia L, Munguía L, Lucas I, de la Torre R, Salas-Salvadó J, Pintó X, Corella D, Granero R, Jiménez-Murcia S, González-Monje I, Esteve-Luque V, Cuenca-Royo A, Gómez-Martínez C, Paz-Graniel I, Forcano L, Fernández-Aranda F. Metabolic, Affective and Neurocognitive Characterization of Metabolic Syndrome Patients with and without Food Addiction. Implications for Weight Progression. Nutrients. 2021 Aug 13;13(8):2779. doi: 10.3390/nu13082779. PMID: 34444940; PMCID: PMC8398101.

50: Turesson Wadell A, Bärebring L, Hulander E, Gjertsson I, Hagberg L, Lindqvist HM, Winkvist A. Effects on health-related quality of life in the randomized, controlled crossover trial ADIRA (Anti-inflammatory Diet In Rheumatoid Arthritis). PLoS One. 2021 Oct 14;16(10):e0258716. doi: 10.1371/journal.pone.0258716. PMID: 34648598; PMCID: PMC8516209.

51: Brauer P, Royall D, Rodrigues A. Use of the Healthy Eating Index in Intervention Studies for Cardiometabolic Risk Conditions: A Systematic Review. Adv Nutr. 2021 Jul 30;12(4):1317-1331. doi: 10.1093/advances/nmaa167. PMID: 33460430; PMCID: PMC8321868.

52: Sasso FC, Pafundi PC, Simeon V, De Nicola L, Chiodini P, Galiero R, Rinaldi L, Nevola R, Salvatore T, Sardu C, Marfella R, Adinolfi LE, Minutolo R; NID-2 Study Group Investigators. Efficacy and durability of multifactorial intervention on mortality and MACEs: a randomized clinical trial in type-2 diabetic kidney disease. Cardiovasc Diabetol. 2021 Jul 16;20(1):145. doi: 10.1186/s12933-021-01343-1. PMID: 34271948; PMCID: PMC8285851.

53: Hand S, Dunstan F, Jones K, Doull I. The effect of diet in infancy on asthma in young adults: the Merthyr Allergy Prevention Study. Thorax. 2021 Nov;76(11):1072-1077. doi: 10.1136/thoraxjnl-2020-215040. Epub 2021 May 7. PMID: 33963089.

54: Beigmohammadi MT, Bitarafan S, Hoseindokht A, Abdollahi A, Amoozadeh L, Soltani D. The effect of supplementation with vitamins A, B, C, D, and E on

disease severity and inflammatory responses in patients with COVID-19: a randomized clinical trial. Trials. 2021 Nov 14;22(1):802. doi: 10.1186/s13063-021-05795-4. PMID: 34776002; PMCID: PMC8590866.

55: Ribó-Coll M, Lassale C, Sacanella E, Ros E, Toledo E, Sorlí JV, Babio N, Lapetra J, Gómez-Gracia E, Alonso-Gómez ÁM, Fiol M, Serra-Majem L, Pinto X, Castañer O, Díez-Espino J, González JI, Becerra-Tomás N, Cofán M, Díaz-López A, Estruch R, Hernáez Á. Mediterranean diet and antihypertensive drug use: a randomized controlled trial. J Hypertens. 2021 Jun 1;39(6):1230-1237. doi: 10.1097/HJH.0000000000002765. PMID: 33496530.

56: Payne Riches S, Piernas C, Aveyard P, Sheppard JP, Rayner M, Albury C, Jebb SA. A Mobile Health Salt Reduction Intervention for People With Hypertension: Results of a Feasibility Randomized Controlled Trial. JMIR Mhealth Uhealth. 2021 Oct 21;9(10):e26233. doi: 10.2196/26233. PMID: 34673535; PMCID: PMC8569539.

57: Kanoni S, Kumar S, Amerikanou C, Kurth MJ, Stathopoulou MG, Bourgeois S, Masson C, Kannt A, Cesarini L, Kontoe MS, Milanović M, Roig FJ, Beribaka M, Campolo J, Jiménez-Hernández N, Milošević N, Llorens C, Smyrnioudis I, Francino MP, Milić N, Kaliora AC, Trivella MG, Ruddock MW, Medić-Stojanoska M, Gastaldelli A, Lamont J, Deloukas P, Dedoussis GV, Visvikis-Siest S. Nutrigenetic Interactions Might Modulate the Antioxidant and Anti-Inflammatory Status in Mastiha-Supplemented Patients With NAFLD. Front Immunol. 2021 May 7;12:683028. doi: 10.3389/fimmu.2021.683028. PMID: 34025683; PMCID: PMC8138178.

58: Kirkham AA, King K, Joy AA, Pelletier AB, Mackey JR, Young K, Zhu X, Meza-Junco J, Basi SK, Hiller JP, Brkin T, Michalowski B, Pituskin E, Paterson DI, Courneya KS, Thompson RB, Prado CM. Rationale and design of the Diet Restriction and Exercise-induced Adaptations in Metastatic breast cancer (DREAM) study: a 2-arm, parallel-group, phase II, randomized control trial of a short-term, calorie-restricted, and ketogenic diet plus exercise during intravenous chemotherapy versus usual care. BMC Cancer. 2021 Oct 10;21(1):1093. doi: 10.1186/s12885-021-08808-2. PMID: 34629067; PMCID: PMC8504029.

59: Rennekamp R, Brandl B, Giesbertz P, Skurk T, Hauner H. Metabolic and satiating effects and consumer acceptance of a fibre-enriched Leberkas meal: a randomized cross-over trial. Eur J Nutr. 2021 Sep;60(6):3203-3210. doi: 10.1007/s00394-020-02472-1. Epub 2021 Feb 8. PMID: 33555374.

60: Caballero FF, Struijk EA, Lana A, Buño A, Rodríguez-Artalejo F, Lopez-Garcia E. Plasma acylcarnitines and risk of lower-extremity functional impairment in older adults: a nested case-control study. Sci Rep. 2021 Feb 8;11(1):3350. doi: 10.1038/s41598-021-82912-y. PMID: 33558555; PMCID: PMC7870673.

61: Byrne J, Murphy C, Keogh JB, Clifton PM. The Effect of Magnesium Supplementation on Endothelial Function: A Randomised Cross-Over Pilot Study. Int J Environ Res Public Health. 2021 Aug 2;18(15):8169. doi: 10.3390/ijerph18158169. PMID: 34360460; PMCID: PMC8346147.

62: Dorling JL, Ravussin E, Redman LM, Bhapkar M, Huffman KM, Racette SB, Das SK, Apolzan JW, Kraus WE, Höchsmann C, Martin CK; CALERIE Phase 2 Study Group. Effect of 2 years of calorie restriction on liver biomarkers: results from the CALERIE phase 2 randomized controlled trial. Eur J Nutr. 2021 Apr;60(3):1633-1643. doi: 10.1007/s00394-020-02361-7. Epub 2020 Aug 14. PMID: 32803412; PMCID: PMC7882001.

63: Foulkes S, Kukuljan S, Nowson CA, Sanders KM, Daly RM. Effects of a multi-modal resistance exercise program and calcium-vitamin D₃ fortified milk on blood pressure and blood lipids in middle-aged and older men: secondary analysis of an 18-month factorial design randomised controlled trial. Eur J Nutr. 2021 Apr;60(3):1289-1299. doi: 10.1007/s00394-020-02325-x. Epub 2020 Jul 14. PMID: 32666313.

64: Edbrooke L, Khaw P, Freimund A, Carpenter D, McNally O, Joubert L, Loeliger J, Traill A, Gough K, Mileshkin L, Denehy L. ENhAncing Lifestyle Behaviors in EndometriaL CancEr (ENABLE): A Pilot Randomized Controlled Trial. Integr Cancer Ther. 2022 Jan-Dec;21:15347354211069885. doi: 10.1177/15347354211069885. PMID: 35045735; PMCID: PMC8785429.

65: Zhong GC, Hu TY, Yang PF, Peng Y, Wu JJ, Sun WP, Cheng L, Wang CR. Chocolate consumption and all-cause and cause-specific mortality in a US population: a post hoc analysis of the PLCO cancer screening trial. Aging (Albany NY). 2021 Jul 29;13(14):18564-18585. doi: 10.18632/aging.203302. Epub 2021 Jul 29. PMID: 34329196; PMCID: PMC8351724.

66: Robbins SR, Melo LRS, Urban H, Deveza LA, Asher R, Johnson VL, Hunter DJ. Effectiveness of Stepped-Care Intervention in Overweight and Obese Patients With Medial Tibiofemoral Osteoarthritis: A Randomized Controlled Trial. Arthritis Care Res (Hoboken). 2021 Apr;73(4):520-530. doi: 10.1002/acr.24148. PMID: 31961489.

67: Matheson EM, Nelson JL, Baggs GE, Luo M, Deutz NE. Specialized oral nutritional supplement (ONS) improves handgrip strength in hospitalized, malnourished older patients with cardiovascular and pulmonary disease: A randomized clinical trial. Clin Nutr. 2021 Mar;40(3):844-849. doi: 10.1016/j.clnu.2020.08.035. Epub 2020 Sep 5. PMID: 32943241.

68: Eisenhauer CM, Brito F, Kupzyk K, Yoder A, Almeida F, Beller RJ, Miller J, Hageman PA. Mobile health assisted self-monitoring is acceptable for supporting weight loss in rural men: a pragmatic randomized controlled feasibility trial. BMC Public Health. 2021 Aug 18;21(1):1568. doi: 10.1186/s12889-021-11618-7. PMID: 34407782; PMCID: PMC8375071.

69: Calvo-Malvar M, Benítez-Estévez AJ, Sánchez-Castro J, Leis R, Gude F. Effects of a Community-Based Behavioral Intervention with a Traditional Atlantic Diet on Cardiometabolic Risk Markers: A Cluster Randomized Controlled Trial ("The GALIAT Study"). Nutrients. 2021 Apr 7;13(4):1211. doi: 10.3390/nu13041211. PMID: 33916940; PMCID: PMC8067574.

70: Laurens C, Grundler F, Damiot A, Chery I, Le Maho AL, Zahariev A, Le Maho Y, Bergouignan A, Gauquelin-Koch G, Simon C, Blanc S, Wilhelmi de Toledo F. Is muscle and protein loss relevant in long-term fasting in healthy men? A

prospective trial on physiological adaptations. J Cachexia Sarcopenia Muscle. 2021 Dec;12(6):1690-1703. doi: 10.1002/jcsm.12766. Epub 2021 Oct 20. PMID: 34668663; PMCID: PMC8718030.

71: Paratthakonkun C, Vimuttipong V, Nana A, Chaijenkij K, Soonthornworasiri N, Arthan D. The Effects of Crocodile Blood Supplementation on Delayed-Onset Muscle Soreness. Nutrients. 2021 Jul 5;13(7):2312. doi: 10.3390/nu13072312. PMID: 34371824; PMCID: PMC8308554.

72: Rist PM, Buring JE, Cook NR, Manson JE, Rexrode KM. Effect of vitamin D and/or omega-3 fatty acid supplementation on stroke outcomes: A randomized trial. Eur J Neurol. 2021 Mar;28(3):809-815. doi: 10.1111/ene.14623. Epub 2020 Nov 24. PMID: 33131164; PMCID: PMC7952033.

73: Dalrymple KV, Tydeman FAS, Taylor PD, Flynn AC, O'Keeffe M, Briley AL, Santosh P, Hayes L, Robson SC, Nelson SM, Sattar N, Whitworth MK, Mills HL, Singh C, Seed CStat PT, White SL, Lawlor DA, Godfrey KM, Poston L; UPBEAT consortium. Adiposity and cardiovascular outcomes in three-year-old children of participants in UPBEAT, an RCT of a complex intervention in pregnant women with obesity. Pediatr Obes. 2021 Mar;16(3):e12725. doi: 10.1111/ijpo.12725. Epub 2020 Sep 11. PMID: 32914569; PMCID: PMC7116719.

74: Howard LSGE, He J, Watson GMJ, Huang L, Wharton J, Luo Q, Kiely DG, Condliffe R, Pepke-Zaba J, Morrell NW, Sheares KK, Ulrich A, Quan R, Zhao Z, Jing X, An C, Liu Z, Xiong C, Robbins PA, Dawes T, de Marvao A, Rhodes CJ, Richter MJ, Gall H, Ghofrani HA, Zhao L, Huson L, Wilkins MR. Supplementation with Iron in Pulmonary Arterial Hypertension. Two Randomized Crossover Trials. Ann Am Thorac Soc. 2021 Jun;18(6):981-988. doi: 10.1513/AnnalsATS.202009-1131OC. PMID: 33735594; PMCID: PMC8456720.

75: Walsh JJ, Neudorf H, Little JP. 14-Day Ketone Supplementation Lowers Glucose and Improves Vascular Function in Obesity: A Randomized Crossover Trial. J Clin Endocrinol Metab. 2021 Mar 25;106(4):e1738-e1754. doi: 10.1210/clinem/dgaa925. PMID: 33367782; PMCID: PMC7993591.

76: Alustiza E, Perales A, Mateo-Abad M, Ozcoidi I, Aizpuru G, Albaina O, Vergara I; en representación del Grupo PRE-STARt Euskadi. Tackling risk factors for type 2 diabetes in adolescents: PRE-STARt study in Euskadi. An Pediatr (Engl Ed). 2021 Sep;95(3):186-196. doi: 10.1016/j.anpede.2020.11.005. Epub 2021 Aug 9. PMID: 34384737.

77: Tang Z, Ming Y, Wu M, Jing J, Xu S, Li H, Zhu Y. Effects of Caloric Restriction and Rope-Skipping Exercise on Cardiometabolic Health: A Pilot Randomized Controlled Trial in Young Adults. Nutrients. 2021 Sep 16;13(9):3222. doi: 10.3390/nu13093222. PMID: 34579097; PMCID: PMC8467906.

78: Bonten TN, Verkleij SM, van der Kleij RM, Busch K, van den Hout WB, Chavannes NH, Numans ME. Selective prevention of cardiovascular disease using integrated lifestyle intervention in primary care: protocol of the Healthy Heart stepped-wedge trial. BMJ Open. 2021 Jul 9;11(7):e043829. doi: 10.1136/bmjopen-2020-043829. PMID: 34244248; PMCID: PMC8273466.

79: Malta MB, Gomes CB, Barros AJD, Baraldi LG, Takito MY, Benício MHD, Carvalhaes MABL. Effectiveness of an intervention focusing on diet and walking during pregnancy in the primary health care service. Cad Saude Publica. 2021 May 24;37(5):e00010320. doi: 10.1590/0102-311X00010320. PMID: 34037070.

80: Toh DWK, Xia X, Sutanto CN, Low JHM, Poh KK, Wang JW, Foo RS, Kim JE. Enhancing the cardiovascular protective effects of a healthy dietary pattern with wolfberry (Lycium barbarum): A randomized controlled trial. Am J Clin Nutr. 2021 Jul 1;114(1):80-89. doi: 10.1093/ajcn/nqab062. Erratum in: Am J Clin Nutr. 2021 Jul 1;114(1):397. PMID: 33964853.

81: Wei J, Liu S, Cheng Y, Yang W, Zhu Z, Zeng L. Association of Infant Physical Development and Rapid Growth With Pubertal Onset Among Girls in Rural China. JAMA Netw Open. 2021 May 3;4(5):e216831. doi: 10.1001/jamanetworkopen.2021.6831. PMID: 33938939; PMCID: PMC8094009.

82: Pitchik HO, Tofail F, Rahman M, Akter F, Sultana J, Shoab AK, Huda TMN, Jahir T, Amin MR, Hossain MK, Das JB, Chung EO, Byrd KA, Yeasmin F, Kwong LH, Forsyth JE, Mridha MK, Winch PJ, Luby SP, Fernald LC. A holistic approach to promoting early child development: a cluster randomised trial of a group-based, multicomponent intervention in rural Bangladesh. BMJ Glob Health. 2021 Mar;6(3):e004307. doi: 10.1136/bmjgh-2020-004307. PMID: 33727278; PMCID: PMC7970287.

83: Hassen HY, Aerts N, Demarest S, Manzar MD, Abrams S, Bastiaens H. Validation of the Dutch-Flemish translated ABCD questionnaire to measure cardiovascular diseases knowledge and risk perception among adults. Sci Rep. 2021 Apr 26;11(1):8952. doi: 10.1038/s41598-021-88456-5. PMID: 33903718; PMCID: PMC8076268.

84: Wu Z, Broad J, Sluyter J, Waayer D, Camargo CA Jr, Scragg R. Effect of monthly vitamin D on diverticular disease hospitalization: Post-hoc analysis of a randomized controlled trial. Clin Nutr. 2021 Mar;40(3):839-843. doi: 10.1016/j.clnu.2020.08.030. Epub 2020 Aug 31. PMID: 32919816.

85: Neumeier WH, Guerra N, Hsieh K, Thirumalai M, Ervin D, Rimmer JH. POWERSforID: Personalized online weight and exercise response system for individuals with intellectual disability: A randomized controlled trial. Disabil Health J. 2021 Oct;14(4):101111. doi: 10.1016/j.dhjo.2021.101111. Epub 2021 Apr 28. PMID: 33965364; PMCID: PMC8448903.

86: Soldevila-Domenech N, Forcano L, Vintró-Alcaraz C, Cuenca-Royo A, Pintó X, Jiménez-Murcia S, García-Gavilán JF, Nishi SK, Babio N, Gomis-González M, Corella D, Sorlí JV, Fernandez-Carrión R, Martínez-González MÁ, Marti A, Salas-Salvadó J, Castañer O, Fernández-Aranda F, Torre R. Interplay between cognition and weight reduction in individuals following a Mediterranean Diet: Three-year follow-up of the PREDIMED-Plus trial. Clin Nutr. 2021 Sep;40(9):5221-5237. doi: 10.1016/j.clnu.2021.07.020. Epub 2021 Aug 5. PMID: 34474192.

87: Walsh JJ, Caldwell HG, Neudorf H, Ainslie PN, Little JP. Short-term ketone

monoester supplementation improves cerebral blood flow and cognition in obesity: A randomized cross-over trial. J Physiol. 2021 Nov;599(21):4763-4778. doi: 10.1113/JP281988. Epub 2021 Oct 4. PMID: 34605026.

88: Marin-Alejandre BA, Cantero I, Perez-Diaz-Del-Campo N, Monreal JI, Elorz M, Herrero JI, Benito-Boillos A, Quiroga J, Martinez-Echeverria A, Uriz-Otano JI, Huarte-Muniesa MP, Tur JA, Martinez JA, Abete I, Zulet MA. Effects of two personalized dietary strategies during a 2-year intervention in subjects with nonalcoholic fatty liver disease: A randomized trial. Liver Int. 2021 Jul;41(7):1532-1544. doi: 10.1111/liv.14818. Epub 2021 Mar 1. PMID: 33550706.

89: Rijnaarts I, de Roos NM, Wang T, Zoetendal EG, Top J, Timmer M, Bouwman EP, Hogenelst K, Witteman B, de Wit N. Increasing dietary fibre intake in healthy adults using personalised dietary advice compared with general advice: a single-blind randomised controlled trial. Public Health Nutr. 2021 Apr;24(5):1117-1128. doi: 10.1017/S1368980020002980. Epub 2020 Sep 18. PMID: 32943128; PMCID: PMC8025104.

90: Reeves MM, Terranova CO, Winkler EAH, McCarthy N, Hickman IJ, Ware RS, Lawler SP, Eakin EG, Demark-Wahnefried W. Effect of a Remotely Delivered Weight Loss Intervention in Early-Stage Breast Cancer: Randomized Controlled Trial. Nutrients. 2021 Nov 15;13(11):4091. doi: 10.3390/nu13114091. PMID: 34836345; PMCID: PMC8622393.

91: Haufe S, Hupa-Breier KL, Bayerle P, Boeck HT, Rolff S, Sundermeier T, Kerling A, Eigendorf J, Kück M, Hanke AA, Ensslen R, Nachbar L, Lauenstein D, Böthig D, Hilfiker-Kleiner D, Stiesch M, Terkamp C, Wedemeyer H, Haverich A, Tegtbur U. Telemonitoring-Supported Exercise Training in Employees With Metabolic Syndrome Improves Liver Inflammation and Fibrosis. Clin Transl Gastroenterol. 2021 Jun 18;12(6):e00371. doi: 10.14309/ctg.0000000000000371. PMID: 34140456; PMCID: PMC8216678.

92: Oncina-Cánovas A, Vioque J, González-Palacios S, Martínez-González MÁ, Salas-Salvadó J, Corella D, Zomeño D, Martínez JA, Alonso-Gómez ÁM, Wärnberg J,

Romaguera D, López-Miranda J, Estruch R, Bernal-Lopez RM, Lapetra J, Serra-Majem JL, Bueno-Cavanillas A, Tur JA, Martín-Sánchez V, Pintó X, Delgado-Rodríguez M, Matía-Martín P, Vidal J, Vázquez C, Daimiel L, Ros E, Toledo E, Babio N, Sorli JV, Schröder H, Zulet MA, Sorto-Sánchez C, Barón-López FJ, Compañ-Gabucio L, Morey M, García-Ríos A, Casas R, Gómez-Pérez AM, Santos-Lozano JM, Vázquez-Ruiz Z, Nishi SK, Asensio EM, Soldevila N, Abete I, Goicolea-Güemez L, Buil-Cosiales P, García-Gavilán JF, Canals E, Torres-Collado L, García-de-la-Hera M. Pro-vegetarian food patterns and cardiometabolic risk in the PREDIMED-Plus study: a cross-sectional baseline analysis. Eur J Nutr. 2022 Feb;61(1):357-372. doi: 10.1007/s00394-021-02647-4. Epub 2021 Aug 9. PMID: 34368892; PMCID: PMC8783853.

93: Barnes C, Yoong SL, Nathan N, Wolfenden L, Wedesweiler T, Kerr J, Ward DS, Grady A. Feasibility of a Web-Based Implementation Intervention to Improve Child Dietary Intake in Early Childhood Education and Care: Pilot Randomized Controlled Trial. J Med Internet Res. 2021 Dec 15;23(12):e25902. doi: 10.2196/25902. PMID: 34914617; PMCID: PMC8717135.

94: García Pérez de Sevilla G, Barceló Guido O, De la Cruz MP, Blanco Fernández A, Alejo LB, Montero Martínez M, Pérez-Ruiz M. Adherence to a Lifestyle Exercise and Nutrition Intervention in University Employees during the COVID-19 Pandemic: A Randomized Controlled Trial. Int J Environ Res Public Health. 2021 Jul 14;18(14):7510. doi: 10.3390/ijerph18147510. PMID: 34299960; PMCID: PMC8305330.

95: Santiago-Torres M, Contento I, Koch P, Tsai WY, Brickman AM, Gaffney AO, Thomson CA, Crane TE, Dominguez N, Sepulveda J, Marín-Chollom AM, Paul R, Shi Z, Ulanday KT, Hale C, Hershman D, Greenlee H. ¡Mi Vida Saludable! A randomized, controlled, 2 × 2 factorial trial of a diet and physical activity intervention among Latina breast cancer survivors: Study design and methods. Contemp Clin Trials. 2021 Nov;110:106524. doi: 10.1016/j.cct.2021.106524. Epub 2021 Aug 6. PMID: 34365016; PMCID: PMC8595705.

96: Calvo-Malvar M, Benítez-Estévez AJ, Leis R, Sánchez-Castro J, Gude F. Changes in Dietary Patterns through a Nutritional Intervention with a Traditional Atlantic Diet: The Galiat Randomized Controlled Trial. Nutrients.

2021 Nov 25;13(12):4233. doi: 10.3390/nu13124233. PMID: 34959784; PMCID: PMC8704078.

97: Pérez-Castrillón JL, Dueñas-Laita A, Brandi ML, Jódar E, Del Pino-Montes J, Quesada-Gómez JM, Cereto Castro F, Gómez-Alonso C, Gallego López L, Olmos Martínez JM, Alhambra Expósito MR, Galarraga B, González-Macías J, Bouillon R, Hernández-Herrero G, Fernández-Hernando N, Arranz-Gutiérrez P, Chinchilla SP. Calcifediol is superior to cholecalciferol in improving vitamin D status in postmenopausal women: a randomized trial. J Bone Miner Res. 2021 Oct;36(10):1967-1978. doi: 10.1002/jbmr.4387. Epub 2021 Jun 24. PMID: 34101900; PMCID: PMC8597097.

98: Ugartemendia L, Bravo R, Reuter M, Castaño MY, Plieger T, Zamoscik V, Kirsch P, Rodríguez AB. SLC6A4 polymorphisms modulate the efficacy of a tryptophan-enriched diet on age-related depression and social cognition. Clin Nutr. 2021 Apr;40(4):1487-1494. doi: 10.1016/j.clnu.2021.02.023. Epub 2021 Feb 22. PMID: 33743283.

99: Glenn AJ, Boucher BA, Kavcic CC, Khan TA, Paquette M, Kendall CWC, Hanley AJ, Jenkins DJA, Sievenpiper JL. Development of a Portfolio Diet Score and Its Concurrent and Predictive Validity Assessed by a Food Frequency Questionnaire. Nutrients. 2021 Aug 19;13(8):2850. doi: 10.3390/nu13082850. PMID: 34445009; PMCID: PMC8398786.

100: Zanganeh M, Adab P, Li B, Pallan M, Liu WJ, Hemming K, Lin R, Liu W, Martin J, Cheng KK, Frew E. Cost-Effectiveness of a School-and Family-Based Childhood Obesity Prevention Programme in China: The "CHIRPY DRAGON" Cluster-Randomised Controlled Trial. Int J Public Health. 2021 Aug 25;66:1604025. doi: 10.3389/ijph.2021.1604025. PMID: 34531712; PMCID: PMC8439195.

101: Kołodziejczyk-Nowotarska A, Bokiniec R, Seliga-Siwecka J. Monitored Supplementation of Vitamin D in Preterm Infants: A Randomized Controlled Trial. Nutrients. 2021 Sep 28;13(10):3442. doi: 10.3390/nu13103442. PMID: 34684442; PMCID: PMC8537871.

102: Newnham ED, Clayton-Chubb D, Nagarethinam M, Hosking P, Gibson PR. Randomised clinical trial: adjunctive induction therapy with oral effervescent budesonide in newly diagnosed coeliac disease. Aliment Pharmacol Ther. 2021 Aug;54(4):419-428. doi: 10.1111/apt.16446. Epub 2021 Jun 28. PMID: 34181750.

103: Dorling JL, Belsky DW, Racette SB, Das SK, Ravussin E, Redman LM, Höchsmann C, Huffman KM, Kraus WE, Kobor MS, MacIsaac JL, Lin DTS, Corcoran DL, Martin CK. Association between the FTO rs9939609 single nucleotide polymorphism and dietary adherence during a 2-year caloric restriction intervention: Exploratory analyses from CALERIE™ phase 2. Exp Gerontol. 2021 Nov;155:111555. doi: 10.1016/j.exger.2021.111555. Epub 2021 Sep 20. PMID: 34543722; PMCID: PMC8720865.

104: Mohammed A, He S. A Double-Blind, Randomized, Placebo-Controlled Trial to Evaluate the Efficacy of a Hydrolyzed Chicken Collagen Type II Supplement in Alleviating Joint Discomfort. Nutrients. 2021 Jul 18;13(7):2454. doi: 10.3390/nu13072454. PMID: 34371963; PMCID: PMC8308696.

105: do Rosario VA, Fitzgerald Z, Broyd S, Paterson A, Roodenrys S, Thomas S, Bliokas V, Potter J, Walton K, Weston-Green K, Yousefi M, Williams D, Wright IMR, Charlton K. Food anthocyanins decrease concentrations of TNF-α in older adults with mild cognitive impairment: A randomized, controlled, double blind clinical trial. Nutr Metab Cardiovasc Dis. 2021 Mar 10;31(3):950-960. doi: 10.1016/j.numecd.2020.11.024. Epub 2020 Dec 5. PMID: 33546942.

106: Nadinskaia M, Maevskaya M, Ivashkin V, Kodzoeva K, Pirogova I, Chesnokov E, Nersesov A, Kaibullayeva J, Konysbekova A, Raissova A, Khamrabaeva F, Zueva E. Ursodeoxycholic acid as a means of preventing atherosclerosis, steatosis and liver fibrosis in patients with nonalcoholic fatty liver disease. World J Gastroenterol. 2021 Mar 14;27(10):959-975. doi: 10.3748/wjg.v27.i10.959. PMID: 33776366; PMCID: PMC7968130.

107: Manzoor S, Hisam A, Aziz S, Mashhadi SF, Haq ZU. Effectiveness of Mobile

Health Augmented Cardiac Rehabilitation on Behavioural Outcomes among Post-acute Coronary Syndrome Patients: A Randomised Controlled Trial. J Coll Physicians Surg Pak. 2021 Oct;31(10):1148-1153. doi: 10.29271/jcpsp.2021.10.1148. PMID: 34601832.

108: Ferguson GM, Meeks Gardner JM, Nelson MR, Giray C, Sundaram H, Fiese BH, Koester B, Tran SP, Powell R. Food-Focused Media Literacy for Remotely Acculturating Adolescents and Mothers: A Randomized Controlled Trial of the "JUS Media? Programme". J Adolesc Health. 2021 Dec;69(6):1013-1023. doi: 10.1016/j.jadohealth.2021.06.006. Epub 2021 Jul 17. PMID: 34281754; PMCID: PMC8628116.

109: Stephen R, Ngandu T, Liu Y, Peltonen M, Antikainen R, Kemppainen N, Laatikainen T, Lötjönen J, Rinne J, Strandberg T, Tuomilehto J, Vanninen R, Soininen H, Kivipelto M, Solomon A; FINGER Study Group. Change in CAIDE Dementia Risk Score and Neuroimaging Biomarkers During a 2-Year Multidomain Lifestyle Randomized Controlled Trial: Results of a Post-Hoc Subgroup Analysis. J Gerontol A Biol Sci Med Sci. 2021 Jul 13;76(8):1407-1414. doi: 10.1093/gerona/glab130. PMID: 33970268; PMCID: PMC8277089.

110: Chow EJ, Doody DR, Di C, Armenian SH, Baker KS, Bricker JB, Gopal AK, Hagen AM, Ketterl TG, Lee SJ, Reding KW, Schenk JM, Syrjala KL, Taylor SA, Wang G, Neuhouser ML, Mendoza JA. Feasibility of a behavioral intervention using mobile health applications to reduce cardiovascular risk factors in cancer survivors: a pilot randomized controlled trial. J Cancer Surviv. 2021 Aug;15(4):554-563. doi: 10.1007/s11764-020-00949-w. Epub 2020 Oct 10. PMID: 33037989; PMCID: PMC8035343.

111: Tasevska N, Sagi-Kiss V, Palma-Duran SA, Barrett B, Chaloux M, Commins J, O'Brien DM, Johnston CS, Midthune D, Kipnis V, Freedman LS. Investigating the performance of 24-h urinary sucrose and fructose as a biomarker of total sugars intake in US participants - a controlled feeding study. Am J Clin Nutr. 2021 Aug 2;114(2):721-730. doi: 10.1093/ajcn/nqab158. Erratum in: Am J Clin Nutr. 2021 Aug 2;114(2):827. PMID: 34036321; PMCID: PMC8326031.

112: Alwosais EZM, Al-Ozairi E, Zafar TA, Alkandari S. Chia seed (<i>Salvia hispanica</i> L.) supplementation to the diet of adults with type 2 diabetes improved systolic blood pressure: A randomized controlled trial. Nutr Health. 2021 Jun;27(2):181-189. doi: 10.1177/0260106020981819. Epub 2021 Feb 2. PMID: 33530854.

113: Pastor-Ibáñez R, Blanco-Heredia J, Etcheverry F, Sánchez-Palomino S, Díez-Fuertes F, Casas R, Navarrete-Muñoz MÁ, Castro-Barquero S, Lucero C, Fernández I, Leal L, Benito JM, Noguera-Julian M, Paredes R, Rallón N, Estruch R, Torrents D, García F. Adherence to a Supplemented Mediterranean Diet Drives Changes in the Gut Microbiota of HIV-1-Infected Individuals. Nutrients. 2021 Mar 30;13(4):1141. doi: 10.3390/nu13041141. PMID: 33808476; PMCID: PMC8067262.

114: Rashid M, Verhoeven AJM, Mulder MT, Timman R, Ozcan B, van Beek-Nieuwland Y, Chow LM, van de Laar RJJM, Dik WA, Sijbrands EJG, Berk KA. The effect of monomeric and oligomeric FLAVAnols in patients with type 2 diabetes and microalbuminuria (FLAVA-trial): A double-blind randomized controlled trial. Clin Nutr. 2021 Nov;40(11):5587-5594. doi: 10.1016/j.clnu.2021.09.038. Epub 2021 Sep 24. PMID: 34656955.

115: Rajendiran E, Lamarche B, She Y, Ramprasath V, Eck P, Brassard D, Gigleux I, Levy E, Tremblay A, Couture P, House JD, Jones PJH, Desmarchelier C. A combination of single nucleotide polymorphisms is associated with the interindividual variability in the blood lipid response to dietary fatty acid consumption in a randomized clinical trial. Am J Clin Nutr. 2021 Aug 2;114(2):564-577. doi: 10.1093/ajcn/nqab064. PMID: 33871574.

116: Domínguez-López I, Marhuenda-Muñoz M, Tresserra-Rimbau A, Hernáez Á, Moreno JJ, Martínez-González MÁ, Salas-Salvadó J, Corella D, Fitó M, Martínez JA, Alonso-Gómez ÁM, Wärnberg J, Vioque J, Romaguera D, López-Miranda J, Bernal-Lopez MR, Lapetra J, Serra-Majem JL, Bueno-Cavanillas A, Tur JA, Martín-Sánchez V, Pintó X, Delgado-Rodríguez M, Matía-Martín P, Vidal J, Vázquez C, Daimiel L, Serra-Mir M, Vázquez-Ruiz Z, Nishi SK, Sorli JV, Castañer O, Abete I, Luna JV, Carabaño-Moral R, Asencio A, Prohens L, Garcia-Rios A, Casas R, Gomez-Perez AM,

Santos-Lozano JM, Razquin C, Martínez MÁ, Saiz C, Robledo-Pastor V, Zulet MA, Salaverria I, Eguaras S, Babio N, Malcampo M, Ros E, Estruch R, López-Sabater MC, Lamuela-Raventós RM. Fruit and Vegetable Consumption is Inversely Associated with Plasma Saturated Fatty Acids at Baseline in Predimed Plus Trial. Mol Nutr Food Res. 2021 Sep;65(17):e2100363. doi: 10.1002/mnfr.202100363. Epub 2021 Jul 28. PMID: 34273124.

117: McCue A, Munten S, Herzig KH, Gagnon DD. Metabolic flexibility is unimpaired during exercise in the cold following acute glucose ingestion in young healthy adults. J Therm Biol. 2021 May;98:102912. doi: 10.1016/j.jtherbio.2021.102912. Epub 2021 Mar 17. PMID: 34016339.

118: Ptomey LT, Willis EA, Reitmeier K, Dreyer Gillette ML, Sherman JR, Sullivan DK. Comparison of energy intake assessed by image-assisted food records to doubly labelled water in adolescents with intellectual and developmental disabilities: a feasibility study. J Intellect Disabil Res. 2021 Apr;65(4):340-347. doi: 10.1111/jir.12816. Epub 2021 Jan 14. PMID: 33443319; PMCID: PMC8499687.

119: Bernal-Jiménez MÁ, Calle-Pérez G, Gutiérrez-Barrios A, Gheorghe L, Solano-Mulero AM, Rodríguez-Martín A, Tur JA, Vázquez-García R, Santi-Cano MJ. Lifestyle and Treatment Adherence Intervention after a Coronary Event Based on an Interactive Web Application (EVITE): Randomized Controlled Clinical Trial Protocol. Nutrients. 2021 May 27;13(6):1818. doi: 10.3390/nu13061818. PMID: 34071782; PMCID: PMC8226528.

120: Sowerby LJ, Patel KB, Schmerk C, Rotenberg BW, Rocha T, Sommer DD. Effect of low salicylate diet on clinical and inflammatory markers in patients with aspirin exacerbated respiratory disease - a randomized crossover trial. J Otolaryngol Head Neck Surg. 2021 Apr 23;50(1):27. doi: 10.1186/s40463-021-00502-4. PMID: 33892819; PMCID: PMC8063291.

121: Feehan J, Degabrielle E, Tripodi N, Al Saedi A, Vogrin S, Duque G. The effect of vitamin D supplementation on circulating osteoprogenitor cells: A

pilot randomized controlled trial. Exp Gerontol. 2021 Jul 15;150:111399. doi: 10.1016/j.exger.2021.111399. Epub 2021 May 7. PMID: 33971278.

122: de Araújo AR, Sampaio GR, da Silva LR, Portal VL, Markoski MM, de Quadros AS, Rogero MM, da Silva Torres EAF, Marcadenti A. Effects of extra virgin olive oil and pecans on plasma fatty acids in patients with stable coronary artery disease. Nutrition. 2021 Nov-Dec;91-92:111411. doi: 10.1016/j.nut.2021.111411. Epub 2021 Jul 15. PMID: 34425320.

123: Dover S, Stephens S, Schneiderman JE, Pullenayegum E, Wells GD, Levy DM, Marcuz JA, Whitney K, Schulze A, Tein I, Feldman BM. The Effect of Creatine Supplementation on Muscle Function in Childhood Myositis: A Randomized, Double-blind, Placebo-controlled Feasibility Study. J Rheumatol. 2021 Mar;48(3):434-441. doi: 10.3899/jrheum.191375. Epub 2020 Aug 1. PMID: 32739897.

124: Binder EF, Christensen JC, Stevens-Lapsley J, Bartley J, Berry SD, Dobs AS, Fortinsky RH, Hildreth KL, Kiel DP, Kuchel GA, Marcus RL, McDonough CM, Orwig D, Sinacore DR, Schwartz RS, Volpi E, Magaziner J, Schechtman KB. A multi-center trial of exercise and testosterone therapy in women after hip fracture: Design, methods and impact of the COVID-19 pandemic. Contemp Clin Trials. 2021 May;104:106356. doi: 10.1016/j.cct.2021.106356. Epub 2021 Mar 11. PMID: 33716173.

125: Khani Jeihooni A, Jormand H, Saadat N, Hatami M, Abdul Manaf R, Afzali Harsini P. The application of the theory of planned behavior to nutritional behaviors related to cardiovascular disease among the women. BMC Cardiovasc Disord. 2021 Dec 7;21(1):589. doi: 10.1186/s12872-021-02399-3. PMID: 34876014; PMCID: PMC8650365.

126: Kang JH, Vyas CM, Okereke OI, Ogata S, Albert M, Lee IM, D'Agostino D, Buring JE, Cook NR, Grodstein F, Manson JE. Effect of vitamin D on cognitive decline: results from two ancillary studies of the VITAL randomized trial. Sci Rep. 2021 Dec 1;11(1):23253. doi: 10.1038/s41598-021-02485-8. PMID: 34853363; PMCID: PMC8636504.

127: Abbate M, Mascaró CM, Montemayor S, Barbería-Latasa M, Casares M, Gómez C, Angullo-Martinez E, Tejada S, Abete I, Zulet MA, Sureda A, Martínez JA, Tur JA. Energy Expenditure Improved Risk Factors Associated with Renal Function Loss in NAFLD and MetS Patients. Nutrients. 2021 Feb 15;13(2):629. doi: 10.3390/nu13020629. PMID: 33672073; PMCID: PMC7919687.

128: Dagne S, Menber Y, Petrucka P, Wassihun Y. Prevalence and associated factors of abdominal obesity among the adult population in Woldia town, Northeast Ethiopia, 2020: Community-based cross-sectional study. PLoS One. 2021 Mar 8;16(3):e0247960. doi: 10.1371/journal.pone.0247960. PMID: 33684157; PMCID: PMC7939297.

129: Chou SH, Murata EM, Yu C, Danik J, Kotler G, Cook NR, Bubes V, Mora S, Chandler PD, Tobias DK, Copeland T, Buring JE, Manson JE, LeBoff MS. Effects of Vitamin D3 Supplementation on Body Composition in the VITamin D and OmegA-3 TriaL (VITAL). J Clin Endocrinol Metab. 2021 Apr 23;106(5):1377-1388. doi: 10.1210/clinem/dgaa981. PMID: 33513226; PMCID: PMC8063236.

130: Dobson P, Burney R, Hales D, Vaughn A, Tovar A, Østbye T, Ward D. Self-Efficacy for Healthy Eating Moderates the Impact of Stress on Diet Quality Among Family Child Care Home Providers. J Nutr Educ Behav. 2021 Apr;53(4):309-315. doi: 10.1016/j.jneb.2021.01.005. PMID: 33838763.

131: Liddle DM , Lin X , Ward EM , Cox LC , Wright AJ , Robinson LE . Apple consumption reduces markers of postprandial inflammation following a high fat meal in overweight and obese adults: A randomized, crossover trial. Food Funct. 2021 Jul 21;12(14):6348-6362. doi: 10.1039/d1fo00392e. Epub 2021 Jun 8. PMID: 34105575.

132: Salmani M, Alipoor E, Navid H, Farahbakhsh P, Yaseri M, Imani H. Effect of l-arginine on cardiac reverse remodeling and quality of life in patients with heart failure. Clin Nutr. 2021 May;40(5):3037-3044. doi: 10.1016/j.clnu.2021.01.044. Epub 2021 Feb 5. PMID: 33610421.

133: D'Alessandro MCO, Gomes AD, Morais JF, Mizubuti YGG, Silva TAD, Fernandes SM, Mendes LL, Correia MITD, Generoso SV. SHORT-TERM EFFECT OF WHEY PROTEIN SUPPLEMENTATION ON THE QUALITY OF LIFE OF PATIENTS WAITING FOR LIVER TRANSPLANTATION: A DOUBLE BLINDED RANDOMIZED CLINICAL TRIAL. Arq Bras Cir Dig. 2021 Oct 15;34(2):e1596. doi: 10.1590/0102-672020210002e1596. PMID: 34669886; PMCID: PMC8521869.

134: van Keulen HM, van Breukelen G, de Vries H, Brug J, Mesters I. A randomized controlled trial comparing community lifestyle interventions to improve adherence to diet and physical activity recommendations: the VitalUM study. Eur J Epidemiol. 2021 Mar;36(3):345-360. doi: 10.1007/s10654-020-00708-2. Epub 2020 Dec 30. PMID: 33377998; PMCID: PMC8032577.

135: Leung GKW, Davis R, Huggins CE, Ware RS, Bonham MP. Does rearranging meal times at night improve cardiovascular risk factors? An Australian pilot randomised trial in night shift workers. Nutr Metab Cardiovasc Dis. 2021 Jun 7;31(6):1890-1902. doi: 10.1016/j.numecd.2021.03.008. Epub 2021 Mar 19. PMID: 33994064.

136: Huang L, Xiao D, Zhang X, Sandhu AK, Chandra P, Kay C, Edirisinghe I, Burton-Freeman B. Strawberry Consumption, Cardiometabolic Risk Factors, and Vascular Function: A Randomized Controlled Trial in Adults with Moderate Hypercholesterolemia. J Nutr. 2021 Jun 1;151(6):1517-1526. doi: 10.1093/jn/nxab034. PMID: 33758944.

137: Saneian H, Khalilian L, Heidari-Beni M, Khademian M, Famouri F, Nasri P, Hassanzadeh A, Kelishadi R. Effect of l-carnitine supplementation on children and adolescents with nonalcoholic fatty liver disease (NAFLD): a randomized, triple-blind, placebo-controlled clinical trial. J Pediatr Endocrinol Metab. 2021 May 4;34(7):897-904. doi: 10.1515/jpem-2020-0642. PMID: 33939897.

138: Smith J, Ayre J, Jansen J, Cvejic E, McCaffery KJ, Doust J, Copp T. Impact of diagnostic labels and causal explanations for weight gain on diet intentions,

cognitions and emotions: An experimental online study. Appetite. 2021 Dec 1;167:105612. doi: 10.1016/j.appet.2021.105612. Epub 2021 Jul 26. PMID: 34324910.

139: Tagliamonte S, Laiola M, Ferracane R, Vitale M, Gallo MA, Meslier V, Pons N, Ercolini D, Vitaglione P. Mediterranean diet consumption affects the endocannabinoid system in overweight and obese subjects: possible links with gut microbiome, insulin resistance and inflammation. Eur J Nutr. 2021 Oct;60(7):3703-3716. doi: 10.1007/s00394-021-02538-8. Epub 2021 Mar 24. PMID: 33763720; PMCID: PMC8437855.

140: Isanaka S, Garba S, Plikaytis B, Malone McNeal M, Guindo O, Langendorf C, Adehossi E, Ciglenecki I, Grais RF. Immunogenicity of an oral rotavirus vaccine administered with prenatal nutritional support in Niger: A cluster randomized clinical trial. PLoS Med. 2021 Aug 10;18(8):e1003720. doi: 10.1371/journal.pmed.1003720. Erratum in: PLoS Med. 2021 Oct 15;18(10):e1003776. PMID: 34375336; PMCID: PMC8354620.

141: Yang B, Yue Y, Chen Y, Ding M, Li B, Wang L, Wang Q, Stanton C, Ross RP, Zhao J, Zhang H, Chen W. <i>Lactobacillus plantarum</i> CCFM1143 Alleviates Chronic Diarrhea <i>via</i> Inflammation Regulation and Gut Microbiota Modulation: A Double-Blind, Randomized, Placebo-Controlled Study. Front Immunol. 2021 Oct 15;12:746585. doi: 10.3389/fimmu.2021.746585. PMID: 34721416; PMCID: PMC8555466.

142: Sadeghi F, Javid AZ, Nazarinasab M, Haghighi-Zadeh MH. Effects of PMS50 supplementation on psychological symptoms of students with premenstrual syndrome. Int J Gynaecol Obstet. 2022 Feb;156(2):247-255. doi: 10.1002/ijgo.13703. Epub 2021 May 29. PMID: 33837571.

143: Taylor JL, Keating SE, Holland DJ, Finlayson G, King NA, Gomersall SR, Rowlands AV, Coombes JS, Leveritt MD. High intensity interval training does not result in short- or long-term dietary compensation in cardiac rehabilitation: Results from the FITR heart study. Appetite. 2021 Mar 1;158:105021. doi:

10.1016/j.appet.2020.105021. Epub 2020 Nov 6. PMID: 33161045.

144: Alzahrani AH, Skytte MJ, Samkani A, Thomsen MN, Astrup A, Ritz C, Frystyk J, Holst JJ, Madsbad S, Haugaard SB, Krarup T, Larsen TM, Magkos F. Effects of a Self-Prepared Carbohydrate-Reduced High-Protein Diet on Cardiovascular Disease Risk Markers in Patients with Type 2 Diabetes. Nutrients. 2021 May 17;13(5):1694. doi: 10.3390/nu13051694. PMID: 34067585; PMCID: PMC8157073.

145: Lacerda JF, Lagos AC, Carolino E, Silva-Herdade AS, Silva M, Sousa Guerreiro C. Functional Food Components, Intestinal Permeability and Inflammatory Markers in Patients with Inflammatory Bowel Disease. Nutrients. 2021 Feb 16;13(2):642. doi: 10.3390/nu13020642. PMID: 33669400; PMCID: PMC7920414.

146: Ismael S, Silvestre MP, Vasques M, Araújo JR, Morais J, Duarte MI, Pestana D, Faria A, Pereira-Leal JB, Vaz J, Ribeiro P, Teixeira D, Marques C, Calhau C. A Pilot Study on the Metabolic Impact of Mediterranean Diet in Type 2 Diabetes: Is Gut Microbiota the Key? Nutrients. 2021 Apr 8;13(4):1228. doi: 10.3390/nu13041228. PMID: 33917736; PMCID: PMC8068165.

147: Fryer S, Stone K, Paterson C, Brown M, Faulkner J, Lambrick D, Credeur D, Zieff G, Martínez Aguirre-Betolaza A, Stoner L. Central and peripheral arterial stiffness responses to uninterrupted prolonged sitting combined with a high-fat meal: a randomized controlled crossover trial. Hypertens Res. 2021 Oct;44(10):1332-1340. doi: 10.1038/s41440-021-00708-z. Epub 2021 Aug 2. PMID: 34334790; PMCID: PMC8490151.

148: Pacheco LS, Bradley RD, Denenberg JO, Anderson CAM, Allison MA. Effects of Different Allotments of Avocados on the Nutritional Status of Families: A Cluster Randomized Controlled Trial. Nutrients. 2021 Nov 11;13(11):4021. doi: 10.3390/nu13114021. PMID: 34836276; PMCID: PMC8623192.

149: Maghsoumi-Norouzabad L, Zare Javid A, Mansoori A, Dadfar M, Serajian A. The effects of Vitamin D3 supplementation on Spermatogram and endocrine factors in

asthenozoospermia infertile men: a randomized, triple blind, placebo-controlled clinical trial. Reprod Biol Endocrinol. 2021 Jul 5;19(1):102. doi: 10.1186/s12958-021-00789-y. PMID: 34225767; PMCID: PMC8256550.

150: Akins JD, Curtis BM, Patik JC, Olvera G, Nasirian A, Campbell JC, Shiva S, Brothers RM. Blunted hyperemic response to mental stress in young, non-Hispanic black men is not impacted by acute dietary nitrate supplementation. J Appl Physiol (1985). 2021 May 1;130(5):1510-1521. doi: 10.1152/japplphysiol.00453.2020. Epub 2021 Mar 25. PMID: 33764167.

151: Stevens Y, Winkens B, Jonkers D, Masclee A. The effect of olive leaf extract on cardiovascular health markers: a randomized placebo-controlled clinical trial. Eur J Nutr. 2021 Jun;60(4):2111-2120. doi: 10.1007/s00394-020-02397-9. Epub 2020 Oct 9. PMID: 33034707; PMCID: PMC8137474.

152: Tettamanzi F, Bagnardi V, Louca P, Nogal A, Monti GS, Mambrini SP, Lucchetti E, Maestrini S, Mazza S, Rodriguez-Mateos A, Scacchi M, Valdes AM, Invitti C, Menni C. A High Protein Diet Is More Effective in Improving Insulin Resistance and Glycemic Variability Compared to a Mediterranean Diet-A Cross-Over Controlled Inpatient Dietary Study. Nutrients. 2021 Dec 7;13(12):4380. doi: 10.3390/nu13124380. PMID: 34959931; PMCID: PMC8707429.

153: Bąk-Sosnowska M, Gruszczyńska M, Skrypnik D, Grzegorczyn S, Karolkiewicz J, Ratajczak M, Mądry E, Walkowiak J, Bogdański P. Type of Physical Training and Selected Aspects of Psychological Functioning of Women with Obesity: A Randomised Trial. Nutrients. 2021 Jul 26;13(8):2555. doi: 10.3390/nu13082555. PMID: 34444714; PMCID: PMC8400574.

154: Zhou M, Zhang N, Zhang Y, Yan X, Li M, Guo W, Guo X, He H, Guo K, Ma G. Effect of Mobile-Based Lifestyle Intervention on Weight Loss among the Overweight and Obese Elderly Population in China: A Randomized Controlled Trial. Int J Environ Res Public Health. 2021 Aug 21;18(16):8825. doi: 10.3390/ijerph18168825. PMID: 34444573; PMCID: PMC8393964.

155: Simpson CA, Zhang JH, Vanderschueren D, Fu L, Pennestri TC, Bouillon R, Cole DEC, Carpenter TO. 25-OHD response to vitamin D supplementation in children: effect of dose but not GC haplotype. Eur J Endocrinol. 2021 Jul 7;185(2):333-342. doi: 10.1530/EJE-21-0349. PMID: 34128826; PMCID: PMC8284876.

156: Zhu R, Fogelholm M, Poppitt SD, Silvestre MP, Møller G, Huttunen-Lenz M, Stratton G, Sundvall J, Råman L, Jalo E, Taylor MA, Macdonald IA, Handjiev S, Handjieva-Darlenska T, Martinez JA, Muirhead R, Brand-Miller J, Raben A. Adherence to a Plant-Based Diet and Consumption of Specific Plant Foods-Associations with 3-Year Weight-Loss Maintenance and Cardiometabolic Risk Factors: A Secondary Analysis of the PREVIEW Intervention Study. Nutrients. 2021 Nov 1;13(11):3916. doi: 10.3390/nu13113916. PMID: 34836170; PMCID: PMC8618731.

157: Vaughn AE, Hennink-Kaminski H, Moore R, Burney R, Chittams JL, Parker P, Luecking CT, Hales D, Ward DS. Evaluating a child care-based social marketing approach for improving children's diet and physical activity: results from the Healthy Me, Healthy We cluster-randomized controlled trial. Transl Behav Med. 2021 Apr 7;11(3):775-784. doi: 10.1093/tbm/ibaa113. PMID: 33231679; PMCID: PMC8033596.

158: Sharma AK, Baig VN, Ahuja J, Sharma S, Panwar RB, Katoch VM, Gupta R. Efficacy of IVRS-based mHealth intervention in reducing cardiovascular risk in metabolic syndrome: A cluster randomized trial. Diabetes Metab Syndr. 2021 Sep-Oct;15(5):102182. doi: 10.1016/j.dsx.2021.06.019. Epub 2021 Jun 21. PMID: 34330073.

159: Schmidt FP, Herzog J, Schnorbus B, Ostad MA, Lasetzki L, Hahad O, Schäfers G, Gori T, Sørensen M, Daiber A, Münzel T. The impact of aircraft noise on vascular and cardiac function in relation to noise event number: a randomized trial. Cardiovasc Res. 2021 Apr 23;117(5):1382-1390. doi: 10.1093/cvr/cvaa204. PMID: 32914847; PMCID: PMC8064430.

160: Rondanelli M, Riva A, Petrangolini G, Allegrini P, Giacosa A, Fazia T, Bernardinelli L, Gasparri C, Peroni G, Perna S. Berberine Phospholipid Is an

Effective Insulin Sensitizer and Improves Metabolic and Hormonal Disorders in Women with Polycystic Ovary Syndrome: A One-Group Pretest-Post-Test Explanatory Study. Nutrients. 2021 Oct 19;13(10):3665. doi: 10.3390/nu13103665. PMID: 34684666; PMCID: PMC8538182.

161: Rubinfeld G, Driggin E, Woolf K, Slater J, Newman JD, Heffron S, Shah B. Factors associated with participation in a short-term dietary intervention study among patients with established coronary artery disease: insights from the EVADE CAD trial. Coron Artery Dis. 2021 May 1;32(3):258-260. doi: 10.1097/MCA.0000000000000925. PMID: 32639244; PMCID: PMC7779744.

162: Rabbani E, Golgiri F, Janani L, Moradi N, Fallah S, Abiri B, Vafa M. Randomized Study of the Effects of Zinc, Vitamin A, and Magnesium Co-supplementation on Thyroid Function, Oxidative Stress, and hs-CRP in Patients with Hypothyroidism. Biol Trace Elem Res. 2021 Nov;199(11):4074-4083. doi: 10.1007/s12011-020-02548-3. Epub 2021 Jan 7. PMID: 33409923.

163: Bartstra JW, Draaisma F, Zwakenberg SR, Lessmann N, Wolterink JM, van der Schouw YT, de Jong PA, Beulens JWJ. Six months vitamin K treatment does not affect systemic arterial calcification or bone mineral density in diabetes mellitus 2. Eur J Nutr. 2021 Apr;60(3):1691-1699. doi: 10.1007/s00394-020-02412-z. Epub 2020 Oct 17. PMID: 33068157; PMCID: PMC7987615.

164: Teo CH, Chin YS, Lim PY, Masrom SAH, Shariff ZM. Impacts of a School-Based Intervention That Incorporates Nutrition Education and a Supportive Healthy School Canteen Environment among Primary School Children in Malaysia. Nutrients. 2021 May 18;13(5):1712. doi: 10.3390/nu13051712. PMID: 34070053; PMCID: PMC8158127.

165: Janyajirawong R, Vilaichone RK, Sethasine S. Efficacy of Zinc Supplement in Minimal hepatic Encephalopathy: A prospective, Randomized Controlled Study (Zinc-MHE Trial). Asian Pac J Cancer Prev. 2021 Sep 1;22(9):2879-2887. doi: 10.31557/APJCP.2021.22.9.2879. PMID: 34582657.

166: Moghtaderi F, Amiri M, Zimorovat A, Raeisi-Dehkordi H, Rahmanian M, Hosseinzadeh M, Fallahzadeh H, Salehi-Abargouei A. The effect of canola, sesame and sesame-canola oils on body fat and composition in adults: a triple-blind, three-way randomised cross-over clinical trial. Int J Food Sci Nutr. 2021 Mar;72(2):226-235. doi: 10.1080/09637486.2020.1786024. Epub 2020 Jul 20. PMID: 32684099.

167: Adamson D, Blazeby J, Porter C, Hurt C, Griffiths G, Nelson A, Sewell B, Jones M, Svobodova M, Fitzsimmons D, Nixon L, Fitzgibbon J, Thomas S, Millin A, Crosby T, Staffurth J, Byrne A. Palliative radiotherapy combined with stent insertion to reduce recurrent dysphagia in oesophageal cancer patients: the ROCS RCT. Health Technol Assess. 2021 May;25(31):1-144. doi: 10.3310/hta25310. PMID: 34042566; PMCID: PMC8182443.

168: Pourabbas M, Bagheri R, Hooshmand Moghadam B, Willoughby DS, Candow DG, Elliott BT, Forbes SC, Ashtary-Larky D, Eskandari M, Wong A, Dutheil F. Strategic Ingestion of High-Protein Dairy Milk during a Resistance Training Program Increases Lean Mass, Strength, and Power in Trained Young Males. Nutrients. 2021 Mar 15;13(3):948. doi: 10.3390/nu13030948. PMID: 33804259; PMCID: PMC7999866.

169: Mousavi-Shirazi-Fard Z, Mazloom Z, Izadi S, Fararouei M. The effects of modified anti-inflammatory diet on fatigue, quality of life, and inflammatory biomarkers in relapsing-remitting multiple sclerosis patients: a randomized clinical trial. Int J Neurosci. 2021 Jul;131(7):657-665. doi: 10.1080/00207454.2020.1750398. Epub 2020 Apr 16. PMID: 32249637.

170: Salamanca-González P, Valls-Zamora RM, Pedret-Figuerola A, Sorlí-Aguilar M, Santigosa-Ayala A, Catalin RE, Pallejà-Millán M, Solà-Alberich R, Martin-Lujan F, The Cenit Research Group Investigators. Effectiveness of a Motivational Nutritional Intervention through Social Networks 2.0 to Increase Adherence to the Mediterranean Diet and Improve Lung Function in Active Smokers: The DIET Study, a Randomized, Controlled and Parallel Clinical Trial in Primary Care. Nutrients. 2021 Oct 14;13(10):3597. doi: 10.3390/nu13103597. PMID: 34684600;

PMCID: PMC8538243.

171: Chan SW, Chu TTW, Choi SW, Benzie IFF, Tomlinson B. Impact of short-term bilberry supplementation on glycemic control, cardiovascular disease risk factors, and antioxidant status in Chinese patients with type 2 diabetes. Phytother Res. 2021 Jun;35(6):3236-3245. doi: 10.1002/ptr.7038. Epub 2021 Feb 18. PMID: 33599340.

172: Mohajeri M, Horriatkhah E, Mohajery R. The effect of glutamine supplementation on serum levels of some inflammatory factors, oxidative stress, and appetite in COVID-19 patients: a case-control study. Inflammopharmacology. 2021 Dec;29(6):1769-1776. doi: 10.1007/s10787-021-00881-0. Epub 2021 Oct 28. Erratum in: Inflammopharmacology. 2021 Dec 20;: PMID: 34709541; PMCID: PMC8552429.

173: Siljander H, Jason E, Ruohtula T, Selvenius J, Koivusaari K, Salonen M, Ahonen S, Honkanen J, Ilonen J, Vaarala O, Virtanen SM, Lähdeaho ML, Knip M. Effect of Early Feeding on Intestinal Permeability and Inflammation Markers in Infants with Genetic Susceptibility to Type 1 Diabetes: A Randomized Clinical Trial. J Pediatr. 2021 Nov;238:305-311.e3. doi: 10.1016/j.jpeds.2021.07.042. Epub 2021 Jul 20. PMID: 34293372.

174: Namkhah Z, Naeini F, Mahdi Rezayat S, Mehdi Yaseri, Mansouri S, Javad Hosseinzadeh-Attar M. Does naringenin supplementation improve lipid profile, severity of hepatic steatosis and probability of liver fibrosis in overweight/obese patients with NAFLD? A randomised, double-blind, placebo-controlled, clinical trial. Int J Clin Pract. 2021 Nov;75(11):e14852. doi: 10.1111/ijcp.14852. Epub 2021 Sep 18. PMID: 34516703.

175: He J, Huang JF, Li C, Chen J, Lu X, Chen JC, He H, Li JX, Cao J, Chen CS, Bazzano LA, Hu D, Kelly TN, Gu DF. Sodium Sensitivity, Sodium Resistance, and Incidence of Hypertension: A Longitudinal Follow-Up Study of Dietary Sodium Intervention. Hypertension. 2021 Jul;78(1):155-164. doi: 10.1161/HYPERTENSIONAHA.120.16758. Epub 2021 Apr 26. PMID: 33896191; PMCID:

PMC8192427.

176: Baghban F, Hosseinzadeh M, Mozaffari-Khosravi H, Dehghan A, Fallahzadeh H. The effect of L-Carnitine supplementation on clinical symptoms, C-reactive protein and malondialdehyde in obese women with knee osteoarthritis: a double blind randomized controlled trial. BMC Musculoskelet Disord. 2021 Feb 17;22(1):195. doi: 10.1186/s12891-021-04059-1. PMID: 33596883; PMCID: PMC7891026.

177: Nagieb CS, Harhash TA, Fayed HL, Ali S. Evaluation of diode laser versus topical corticosteroid in management of Behcet's disease-associated oral ulcers: a randomized clinical trial. Clin Oral Investig. 2022 Jan;26(1):697-704. doi: 10.1007/s00784-021-04047-8. Epub 2021 Jul 2. PMID: 34212234.

178: Stupin A, Drenjančević I, Šušnjara P, Debeljak Ž, Kolobarić N, Jukić I, Mihaljević Z, Martinović G, Selthofer-Relatić K. Is There Association between Altered Adrenergic System Activity and Microvascular Endothelial Dysfunction Induced by a 7-Day High Salt Intake in Young Healthy Individuals. Nutrients. 2021 May 20;13(5):1731. doi: 10.3390/nu13051731. PMID: 34065261; PMCID: PMC8161165.

179: Mitchell CM, Davy BM, Ponder MA, McMillan RP, Hughes MD, Hulver MW, Neilson AP, Davy KP. Prebiotic Inulin Supplementation and Peripheral Insulin Sensitivity in adults at Elevated Risk for Type 2 Diabetes: A Pilot Randomized Controlled Trial. Nutrients. 2021 Sep 17;13(9):3235. doi: 10.3390/nu13093235. PMID: 34579112; PMCID: PMC8471706.

180: Attwells S, Setiawan E, Rusjan PM, Xu C, Kish SJ, Vasdev N, Houle S, Santhirakumar A, Meyer JH. A double-blind placebo-controlled trial of minocycline on translocator protein distribution volume in treatment-resistant major depressive disorder. Transl Psychiatry. 2021 May 29;11(1):334. doi: 10.1038/s41398-021-01450-3. PMID: 34052828; PMCID: PMC8164633.

181: Moludi J, Kafil HS, Qaisar SA, Gholizadeh P, Alizadeh M, Vayghyan HJ.

Effect of probiotic supplementation along with calorie restriction on metabolic endotoxemia, and inflammation markers in coronary artery disease patients: a double blind placebo controlled randomized clinical trial. Nutr J. 2021 Jun 1;20(1):47. doi: 10.1186/s12937-021-00703-7. PMID: 34074289; PMCID: PMC8170788.

182: Rondanelli M, Peroni G, Riva A, Petrangolini G, Allegrini P, Fazia T, Bernardinelli L, Naso M, Faliva MA, Tartara A, Gasparri C, Infantino V, Perna S. Bergamot phytosome improved visceral fat and plasma lipid profiles in overweight and obese class I subject with mild hypercholesterolemia: A randomized placebo controlled trial. Phytother Res. 2021 Apr;35(4):2045-2056. doi: 10.1002/ptr.6950. Epub 2020 Nov 13. PMID: 33188552; PMCID: PMC8246838.

183: Schmidt KA, Cromer G, Burhans MS, Kuzma JN, Hagman DK, Fernando I, Murray M, Utzschneider KM, Holte S, Kraft J, Kratz M. The impact of diets rich in low-fat or full-fat dairy on glucose tolerance and its determinants: a randomized controlled trial. Am J Clin Nutr. 2021 Mar 11;113(3):534-547. doi: 10.1093/ajcn/nqaa301. PMID: 33184632; PMCID: PMC7948850.

184: Victoria-Montesinos D, Sánchez-Macarro M, Gabaldón-Hernández JA, Abellán-Ruiz MS, Querol-Calderón M, Luque-Rubia AJ, Bernal-Morell E, Ávila-Gandía V, López-Román FJ. Effect of Dietary Supplementation with a Natural Extract of <i>Sclerocarya birrea</i> on Glycemic Metabolism in Subjects with Prediabetes: A Randomized Double-Blind Placebo-Controlled Study. Nutrients. 2021 Jun 6;13(6):1948. doi: 10.3390/nu13061948. PMID: 34204042; PMCID: PMC8229573.

185: Madjd A, Taylor MA, Delavari A, Malekzadeh R, Macdonald IA, Farshchi HR. Effects of consuming later evening meal <i>v</i>. earlier evening meal on weight loss during a weight loss diet: a randomised clinical trial. Br J Nutr. 2021 Aug 28;126(4):632-640. doi: 10.1017/S0007114520004456. Epub 2020 Nov 11. PMID: 33172509.

186: Azhar G, Wei JY, Schutzler SE, Coker K, Gibson RV, Kirby MF, Ferrando AA, Wolfe RR. Daily Consumption of a Specially Formulated Essential Amino Acid-Based Dietary Supplement Improves Physical Performance in Older Adults With Low

Physical Functioning. J Gerontol A Biol Sci Med Sci. 2021 Jun 14;76(7):1184-1191. doi: 10.1093/gerona/glab019. PMID: 33475727; PMCID: PMC8202157.

187: Hernáez Á, Lassale C, Castro-Barquero S, Ros E, Tresserra-Rimbau A, Castañer O, Pintó X, Vázquez-Ruiz Z, Sorlí JV, Salas-Salvadó J, Lapetra J, Gómez-Gracia E, Alonso-Gómez ÁM, Fiol M, Serra-Majem L, Sacanella E, Razquin C, Corella D, Guasch-Ferré M, Cofán M, Estruch R. Mediterranean Diet Maintained Platelet Count within a Healthy Range and Decreased Thrombocytopenia-Related Mortality Risk: A Randomized Controlled Trial. Nutrients. 2021 Feb 8;13(2):559. doi: 10.3390/nu13020559. PMID: 33567733; PMCID: PMC7915168.

188: Hosseini R, Montazerifar F, Shahraki E, Karajibani M, Mokhtari AM, Dashipour AR, Ferns GA, Jalali M. The Effects of Zinc Sulfate Supplementation on Serum Copeptin, C-Reactive Protein and Metabolic Markers in Zinc-Deficient Diabetic Patients on Hemodialysis: A Randomized, Double-Blind, Placebo-Controlled Trial. Biol Trace Elem Res. 2022 Jan;200(1):76-83. doi: 10.1007/s12011-021-02649-7. Epub 2021 Mar 3. PMID: 33655432.

189: Crabtree CD, Kackley ML, Buga A, Fell B, LaFountain RA, Hyde PN, Sapper TN, Kraemer WJ, Scandling D, Simonetti OP, Volek JS. Comparison of Ketogenic Diets with and without Ketone Salts versus a Low-Fat Diet: Liver Fat Responses in Overweight Adults. Nutrients. 2021 Mar 17;13(3):966. doi: 10.3390/nu13030966. PMID: 33802651; PMCID: PMC8002465.

190: Munk T, Svendsen JA, Knudsen AW, Østergaard TB, Thomsen T, Olesen SS, Rasmussen HH, Beck AM. A multimodal nutritional intervention after discharge improves quality of life and physical function in older patients - a randomized controlled trial. Clin Nutr. 2021 Nov;40(11):5500-5510. doi: 10.1016/j.clnu.2021.09.029. Epub 2021 Sep 24. PMID: 34656032.

191: Razmpoosh E, Safi S, Nadjarzadeh A, Fallahzadeh H, Abdollahi N, Mazaheri M, Nazari M, Salehi-Abargouei A. The effect of Nigella sativa supplementation on cardiovascular risk factors in obese and overweight women: a crossover, double-

blind, placebo-controlled randomized clinical trial. Eur J Nutr. 2021 Jun;60(4):1863-1874. doi: 10.1007/s00394-020-02374-2. Epub 2020 Sep 2. PMID: 32876804.

192: Markey O, Vasilopoulou D, Kliem KE, Fagan CC, Grandison AS, Sutton R, Humphries DJ, Todd S, Jackson KG, Givens DI, Lovegrove JA. Postprandial Fatty Acid Profile, but Not Cardiometabolic Risk Markers, Is Modulated by Dairy Fat Manipulation in Adults with Moderate Cardiovascular Disease Risk: The Randomized Controlled REplacement of SaturatEd fat in dairy on Total cholesterol (RESET) Study. J Nutr. 2021 Jul 1;151(7):1755-1768. doi: 10.1093/jn/nxab050. PMID: 33758921; PMCID: PMC8327197.

193: Moghadam BH, Bagheri R, Roozbeh B, Ashtary-Larky D, Gaeini AA, Dutheil F, Wong A. Impact of saffron (Crocus Sativus Linn) supplementation and resistance training on markers implicated in depression and happiness levels in untrained young males. Physiol Behav. 2021 May 1;233:113352. doi: 10.1016/j.physbeh.2021.113352. Epub 2021 Feb 6. PMID: 33556410.

194: Sangouni AA, Alizadeh M, Jamalzehi A, Parastouei K. Effects of garlic powder supplementation on metabolic syndrome components, insulin resistance, fatty liver index, and appetite in subjects with metabolic syndrome: A randomized clinical trial. Phytother Res. 2021 Aug;35(8):4433-4441. doi: 10.1002/ptr.7146. Epub 2021 May 11. PMID: 33974725.

195: Toupchian O, Abdollahi S, Salehi-Abargouei A, Heshmati J, Clark CCT, Sheikhha MH, Fallahzadeh H, Mozaffari-Khosravi H. The effects of resveratrol supplementation on PPARα, p16, p53, p21 gene expressions, and sCD163/sTWEAK ratio in patients with type 2 diabetes mellitus: A double-blind controlled randomized trial. Phytother Res. 2021 Jun;35(6):3205-3213. doi: 10.1002/ptr.7031. Epub 2021 Feb 13. PMID: 33580595.

196: Nogay NH, Walton J, Roberts KM, Nahikian-Nelms M, Witwer AN. The Effect of the Low FODMAP Diet on Gastrointestinal Symptoms, Behavioral Problems and Nutrient Intake in Children with Autism Spectrum Disorder: A Randomized

Controlled Pilot Trial. J Autism Dev Disord. 2021 Aug;51(8):2800-2811. doi: 10.1007/s10803-020-04717-8. PMID: 33057858.

197: Huang G, Pencina K, Li Z, Apovian CM, Travison TG, Storer TW, Gagliano-Jucá T, Basaria S, Bhasin S. Effect of Protein Intake on Visceral Abdominal Fat and Metabolic Biomarkers in Older Men With Functional Limitations: Results From a Randomized Clinical Trial. J Gerontol A Biol Sci Med Sci. 2021 May 22;76(6):1084-1089. doi: 10.1093/gerona/glab007. PMID: 33417663; PMCID: PMC8140050.

198: Jørgensen HS, Eide IA, Jenssen T, Åsberg A, Bollerslev J, Godang K, Hartmann A, Schmidt EB, Svensson M. Marine n-3 Polyunsaturated Fatty Acids and Bone Mineral Density in Kidney Transplant Recipients: A Randomized, Placebo-Controlled Trial. Nutrients. 2021 Jul 10;13(7):2361. doi: 10.3390/nu13072361. PMID: 34371870; PMCID: PMC8308635.

199: He S, Le NA, Ramìrez-Zea M, Martorell R, Narayan KMV, Stein AD. Postprandial glycemic response differed by early life nutritional exposure in a longitudinal cohort: a single- and multi-biomarker approach. Eur J Nutr. 2021 Jun;60(4):1973-1984. doi: 10.1007/s00394-020-02389-9. Epub 2020 Sep 24. PMID: 32970235; PMCID: PMC7987862.

200: Morshedzadeh N, Rahimlou M, Shahrokh S, Karimi S, Mirmiran P, Zali MR. The effects of flaxseed supplementation on metabolic syndrome parameters, insulin resistance and inflammation in ulcerative colitis patients: An open-labeled randomized controlled trial. Phytother Res. 2021 Jul;35(7):3781-3791. doi: 10.1002/ptr.7081. Epub 2021 Apr 15. PMID: 33856729.
