# Supplementary material for: Perspectives of Dietary Assessment in Human Health and Disease
Source: Nutrients. 2022 Feb 16;14(4):830. doi: 10.3390/nu14040830 (PMC8877528; doi:10.3390/nu14040830)
Supplement: Supplementary file 1 [file nutrients-14-00830-s001.zip › Table S5.pdf]

Table S5 - PubMed search keywords "dietary assessment human health disease", filters "1 year" and "systematic reviews"

starting date 08/02/2022

Type of article: Systematic reviews

n = 171

1: Bassatne A, Basbous M, Chakhtoura M, El Zein O, Rahme M, El-Hajj Fuleihan G. The link between COVID-19 and VItamin D (VIVID): A systematic review and meta-analysis. Metabolism. 2021 Jun;119:154753. doi: 10.1016/j.metabol.2021.154753. Epub 2021 Mar 24. PMID: 33774074; PMCID: PMC7989070.

2: English LK, Ard JD, Bailey RL, Bates M, Bazzano LA, Boushey CJ, Brown C, Butera G, Callahan EH, de Jesus J, Mattes RD, Mayer-Davis EJ, Novotny R, Obbagy JE, Rahavi EB, Sabate J, Snetselaar LG, Stoody EE, Van Horn LV, Venkatramanan S, Heymsfield SB. Evaluation of Dietary Patterns and All-Cause Mortality: A Systematic Review. JAMA Netw Open. 2021 Aug 2;4(8):e2122277. doi: 10.1001/jamanetworkopen.2021.22277. PMID: 34463743; PMCID: PMC8408672.

3: Quintela BCSF, Carioca AAF, de Oliveira JGR, Fraser SDS, da Silva Junior GB. Dietary patterns and chronic kidney disease outcomes: A systematic review. Nephrology (Carlton). 2021 Jul;26(7):603-612. doi: 10.1111/nep.13883. Epub 2021 Apr 28. PMID: 33864650.

4: Chiavaroli L, Lee D, Ahmed A, Cheung A, Khan TA, Blanco S, Mejia, Mirrahimi A, Jenkins DJA, Livesey G, Wolever TMS, Rahelić D, Kahleová H, Salas-Salvadó J, Kendall CWC, Sievenpiper JL. Effect of low glycaemic index or load dietary patterns on glycaemic control and cardiometabolic risk factors in diabetes: systematic review and meta-analysis of randomised controlled trials. BMJ. 2021 Aug 4;374:n1651. doi: 10.1136/bmj.n1651. Erratum in: BMJ. 2021 Aug 26;374:n2114. PMID: 34348965; PMCID: PMC8336013.

5: Baygi F, Mohammadi-Nasrabadi F, Zyriax BC, Jensen OC, Bygvraa DA, Oldenburg M, Nielsen JB. Global overview of dietary outcomes and dietary intake assessment methods in maritime settings: a systematic review. BMC Public Health. 2021 Aug

21;21(1):1579. doi: 10.1186/s12889-021-11593-z. PMID: 34419000; PMCID: PMC8379789.

6: Dahl IK, Dalgård C. Sami dietary habits and the risk of cardiometabolic disease: a systematic review. Int J Circumpolar Health. 2021 Dec;80(1):1873621. doi: 10.1080/22423982.2021.1873621. PMID: 33463398; PMCID: PMC7832988.

7: Martini D, Godos J, Marventano S, Tieri M, Ghelfi F, Titta L, Lafranconi A, Trigueiro H, Gambera A, Alonzo E, Sciacca S, Buscemi S, Ray S, Galvano F, Del Rio D, Grosso G. Nut and legume consumption and human health: an umbrella review of observational studies. Int J Food Sci Nutr. 2021 Nov;72(7):871-878. doi: 10.1080/09637486.2021.1880554. Epub 2021 Feb 4. PMID: 33541169.

8: Naghshi S, Aune D, Beyene J, Mobarak S, Asadi M, Sadeghi O. Dietary intake and biomarkers of alpha linolenic acid and risk of all cause, cardiovascular, and cancer mortality: systematic review and dose-response meta-analysis of cohort studies. BMJ. 2021 Oct 13;375:n2213. doi: 10.1136/bmj.n2213. PMID: 34645650; PMCID: PMC8513503.

9: Little M, Hagar H, Zivot C, Dodd W, Skinner K, Kenny TA, Caughey A, Gaupholm J, Lemire M. Drivers and health implications of the dietary transition among Inuit in the Canadian Arctic: a scoping review. Public Health Nutr. 2021 Jun;24(9):2650-2668. doi: 10.1017/S1368980020002402. Epub 2020 Sep 11. PMID: 32914743.

10: Buja A, Grotto G, Montecchio L, De Battisti E, Sperotto M, Bertoncello C, Cocchio S, Baldovin T, Baldo V. Association between health literacy and dietary intake of sugar, fat and salt: a systematic review. Public Health Nutr. 2021 Jun;24(8):2085-2097. doi: 10.1017/S1368980020002311. Epub 2020 Aug 3. PMID: 32744216; PMCID: PMC8145460.

11: Verger EO, Le Port A, Borderon A, Bourbon G, Moursi M, Savy M, Mariotti F, Martin-Prevel Y. Dietary Diversity Indicators and Their Associations with Dietary Adequacy and Health Outcomes: A Systematic Scoping Review. Adv Nutr.

2021 Oct 1;12(5):1659-1672. doi: 10.1093/advances/nmab009. PMID: 33684194; PMCID: PMC8483968.

12: Deane KHO, Jimoh OF, Biswas P, O'Brien A, Hanson S, Abdelhamid AS, Fox C, Hooper L. Omega-3 and polyunsaturated fat for prevention of depression and anxiety symptoms: systematic review and meta-analysis of randomised trials. Br J Psychiatry. 2021 Mar;218(3):135-142. doi: 10.1192/bjp.2019.234. PMID: 31647041.

13: Fenton S, Burrows TL, Skinner JA, Duncan MJ. The influence of sleep health on dietary intake: a systematic review and meta-analysis of intervention studies. J Hum Nutr Diet. 2021 Apr;34(2):273-285. doi: 10.1111/jhn.12813. Epub 2020 Oct 1. PMID: 33001515.

14: Hassani Zadeh S, Mansoori A, Hosseinzadeh M. Relationship between dietary patterns and non-alcoholic fatty liver disease: A systematic review and meta-analysis. J Gastroenterol Hepatol. 2021 Jun;36(6):1470-1478. doi: 10.1111/jgh.15363. Epub 2020 Dec 14. PMID: 33269500.

15: Liu FH, Liu C, Gong TT, Gao S, Sun H, Jiang YT, Zhang JY, Zhang M, Gao C, Li XY, Zhao YH, Wu QJ. Dietary Inflammatory Index and Health Outcomes: An Umbrella Review of Systematic Review and Meta-Analyses of Observational Studies. Front Nutr. 2021 May 19;8:647122. doi: 10.3389/fnut.2021.647122. PMID: 34095187; PMCID: PMC8169973.

16: Liu YS, Wu QJ, Lv JL, Jiang YT, Sun H, Xia Y, Chang Q, Zhao YH. Dietary Carbohydrate and Diverse Health Outcomes: Umbrella Review of 30 Systematic Reviews and Meta-Analyses of 281 Observational Studies. Front Nutr. 2021 Apr 29;8:670411. doi: 10.3389/fnut.2021.670411. PMID: 33996880; PMCID: PMC8116488.

17: Ajabnoor SM, Thorpe G, Abdelhamid A, Hooper L. Long-term effects of increasing omega-3, omega-6 and total polyunsaturated fats on inflammatory bowel disease and markers of inflammation: a systematic review and meta-analysis of randomized controlled trials. Eur J Nutr. 2021 Aug;60(5):2293-2316. doi: 10.1007/s00394-020-02413-y. Epub 2020 Oct 21. PMID: 33084958.

18: Rees K, Al-Khudairy L, Takeda A, Stranges S. Vegan dietary pattern for the primary and secondary prevention of cardiovascular diseases. Cochrane Database Syst Rev. 2021 Feb 25;2(2):CD013501. doi: 10.1002/14651858.CD013501.pub2. PMID: 33629376; PMCID: PMC8092640.

19: Whittaker J, Wu K. Low-fat diets and testosterone in men: Systematic review and meta-analysis of intervention studies. J Steroid Biochem Mol Biol. 2021 Jun;210:105878. doi: 10.1016/j.jsbmb.2021.105878. Epub 2021 Mar 16. PMID: 33741447.

20: Windus JL, Burrows TL, Duncanson K, Collins CE, Rollo ME. Scoping review of nutrition intervention and dietary assessment studies in Khmer populations living in Cambodia. J Hum Nutr Diet. 2021 Dec;34(6):953-968. doi: 10.1111/jhn.12932. Epub 2021 Aug 16. PMID: 34231266.

21: Kaiser J, van Daalen KR, Thayyil A, Cocco MTARR, Caputo D, Oliver-Williams C. A Systematic Review of the Association Between Vegan Diets and Risk of Cardiovascular Disease. J Nutr. 2021 Jun 1;151(6):1539-1552. doi: 10.1093/jn/nxab037. PMID: 33831953; PMCID: PMC8169813.

22: Corrao S, Mallaci Bocchio R, Lo Monaco M, Natoli G, Cavezzi A, Troiani E, Argano C. Does Evidence Exist to Blunt Inflammatory Response by Nutraceutical Supplementation during COVID-19 Pandemic? An Overview of Systematic Reviews of Vitamin D, Vitamin C, Melatonin, and Zinc. Nutrients. 2021 Apr 12;13(4):1261. doi: 10.3390/nu13041261. PMID: 33921297; PMCID: PMC8069903.

23: Fatemeh G, Sajjad M, Niloufar R, Neda S, Leila S, Khadijeh M. Effect of melatonin supplementation on sleep quality: a systematic review and meta-analysis of randomized controlled trials. J Neurol. 2022 Jan;269(1):205-216. doi: 10.1007/s00415-020-10381-w. Epub 2021 Jan 8. PMID: 33417003.

24: Schwab U, Reynolds AN, Sallinen T, Rivellese AA, Risérus U. Dietary fat intakes and cardiovascular disease risk in adults with type 2 diabetes: a

systematic review and meta-analysis. Eur J Nutr. 2021 Sep;60(6):3355-3363. doi: 10.1007/s00394-021-02507-1. Epub 2021 Feb 21. PMID: 33611616.

25: Kim Y, Je Y, Giovannucci EL. Association between dietary fat intake and mortality from all-causes, cardiovascular disease, and cancer: A systematic review and meta-analysis of prospective cohort studies. Clin Nutr. 2021 Mar;40(3):1060-1070. doi: 10.1016/j.clnu.2020.07.007. Epub 2020 Jul 14. PMID: 32723506.

26: Low JHM, Toh DWK, Ng MTT, Fam J, Kua EH, Kim JE. A Systematic Review and Meta-Analysis of the Impact of Different Intensity of Dietary Counselling on Cardiometabolic Health in Middle-Aged and Older Adults. Nutrients. 2021 Aug 25;13(9):2936. doi: 10.3390/nu13092936. PMID: 34578814; PMCID: PMC8469488.

27: Peñalvo JL, Sagastume D, Mertens E, Uzhova I, Smith J, Wu JHY, Bishop E, Onopa J, Shi P, Micha R, Mozaffarian D. Effectiveness of workplace wellness programmes for dietary habits, overweight, and cardiometabolic health: a systematic review and meta-analysis. Lancet Public Health. 2021 Sep;6(9):e648-e660. doi: 10.1016/S2468-2667(21)00140-7. PMID: 34454642; PMCID: PMC8627548.

28: Lukomskyj N, Allman-Farinelli M, Shi Y, Rangan A. Dietary exposures in childhood and adulthood and cardiometabolic outcomes: a systematic scoping review. J Hum Nutr Diet. 2021 Jun;34(3):511-523. doi: 10.1111/jhn.12841. Epub 2021 Jan 6. PMID: 33406314.

29: Boelens Keun JT, Arnoldussen IA, Vriend C, van de Rest O. Dietary Approaches to Improve Efficacy and Control Side Effects of Levodopa Therapy in Parkinson's Disease: A Systematic Review. Adv Nutr. 2021 Dec 1;12(6):2265-2287. doi: 10.1093/advances/nmab060. PMID: 34113965; PMCID: PMC8634393.

30: Kocanda L, Brain K, Frawley J, Schumacher TL, May J, Rollo ME, Brown LJ. The Effectiveness of Randomized Controlled Trials to Improve Dietary Intake in the Context of Cardiovascular Disease Prevention and Management in Rural

Communities: A Systematic Review. J Acad Nutr Diet. 2021 Oct;121(10):2046-2070.e1. doi: 10.1016/j.jand.2021.05.025. Epub 2021 Jul 8. PMID: 34247977.

31: McKenzie BL, Coyle DH, Santos JA, Burrows T, Rosewarne E, Peters SAE, Carcel C, Jaacks LM, Norton R, Collins CE, Woodward M, Webster J. Investigating sex differences in the accuracy of dietary assessment methods to measure energy intake in adults: a systematic review and meta-analysis. Am J Clin Nutr. 2021 May 8;113(5):1241-1255. doi: 10.1093/ajcn/nqaa370. PMID: 33564834; PMCID: PMC8106762.

32: Garcêz LS, Avelar CR, Fonseca NSS, Costa PRF, Lyra AC, Cunha CM, Jesus RP, Oliveira LPM. Effect of dietary carbohydrate and lipid modification on clinical and anthropometric parameters in nonalcoholic fatty liver disease: a systematic review and meta-analysis. Nutr Rev. 2021 Nov 10;79(12):1321-1337. doi: 10.1093/nutrit/nuaa146. PMID: 33515021.

33: Ooi JY, Wolfenden L, Sutherland R, Nathan N, Oldmeadow C, Mclaughlin M, Barnes C, Hall A, Vanderlee L, Yoong SL. A Systematic Review of the Recent Consumption Levels of Sugar-Sweetened Beverages in Children and Adolescents From the World Health Organization Regions With High Dietary-Related Burden of Disease. Asia Pac J Public Health. 2022 Jan;34(1):11-24. doi: 10.1177/10105395211014642. Epub 2021 May 20. PMID: 34013784.

34: Sellem L, Flourakis M, Jackson KG, Joris PJ, Lumley J, Lohner S, Mensink RP, Soedamah-Muthu SS, Lovegrove JA. Impact of Individual Dietary Saturated Fatty Acid Replacement on Circulating Lipids and Other Biomarkers of Cardiometabolic Health: A Systematic Review and Meta-analysis of RCTs in Humans. Adv Nutr. 2021 Nov 25:nmab143. doi: 10.1093/advances/nmab143. Epub ahead of print. PMID: 34849532.

35: de Araújo TP, de Moraes MM, Magalhães V, Afonso C, Santos C, Rodrigues SSP. Ultra-Processed Food Availability and Noncommunicable Diseases: A Systematic Review. Int J Environ Res Public Health. 2021 Jul 10;18(14):7382. doi:

10.3390/ijerph18147382. PMID: 34299832; PMCID: PMC8306957.

36: Bortone I, Sardone R, Lampignano L, Castellana F, Zupo R, Lozupone M, Moretti B, Giannelli G, Panza F. How gait influences frailty models and health-related outcomes in clinical-based and population-based studies: a systematic review. J Cachexia Sarcopenia Muscle. 2021 Apr;12(2):274-297. doi: 10.1002/jcsm.12667. Epub 2021 Feb 16. PMID: 33590975; PMCID: PMC8061366.

37: O'Donovan S, Palermo C, Ryan L. Competency-based assessment in nutrition education: A systematic literature review. J Hum Nutr Diet. 2022 Feb;35(1):102-111. doi: 10.1111/jhn.12946. Epub 2021 Sep 19. PMID: 34541713.

38: Jolliffe DA, Camargo CA Jr, Sluyter JD, Aglipay M, Aloia JF, Ganmaa D, Bergman P, Bischoff-Ferrari HA, Borzutzky A, Damsgaard CT, Dubnov-Raz G, Esposito S, Gilham C, Ginde AA, Golan-Tripto I, Goodall EC, Grant CC, Griffiths CJ, Hibbs AM, Janssens W, Khadilkar AV, Laaksi I, Lee MT, Loeb M, Maguire JL, Majak P, Mauger DT, Manaseki-Holland S, Murdoch DR, Nakashima A, Neale RE, Pham H, Rake C, Rees JR, Rosendahl J, Scragg R, Shah D, Shimizu Y, Simpson-Yap S, Trilok-Kumar G, Urashima M, Martineau AR. Vitamin D supplementation to prevent acute respiratory infections: a systematic review and meta-analysis of aggregate data from randomised controlled trials. Lancet Diabetes Endocrinol. 2021 May;9(5):276-292. doi: 10.1016/S2213-8587(21)00051-6. Epub 2021 Mar 30. PMID: 33798465.

39: Li N, Wu X, Zhuang W, Xia L, Chen Y, Wang Y, Wu C, Rao Z, Du L, Zhao R, Yi M, Wan Q, Zhou Y. Green leafy vegetable and lutein intake and multiple health outcomes. Food Chem. 2021 Oct 30;360:130145. doi: 10.1016/j.foodchem.2021.130145. Epub 2021 May 18. PMID: 34034049.

40: Aleksandrova K, Koelman L, Rodrigues CE. Dietary patterns and biomarkers of oxidative stress and inflammation: A systematic review of observational and intervention studies. Redox Biol. 2021 Jun;42:101869. doi: 10.1016/j.redox.2021.101869. Epub 2021 Jan 22. PMID: 33541846; PMCID: PMC8113044.

41: Bryant L, Rangan A, Grafenauer S. Lupins and Health Outcomes: A Systematic Literature Review. Nutrients. 2022 Jan 13;14(2):327. doi: 10.3390/nu14020327. PMID: 35057507; PMCID: PMC8777979.

42: Ng C, Major G, Smyth AR. Timing of pancreatic enzyme replacement therapy (PERT) in cystic fibrosis. Cochrane Database Syst Rev. 2021 Aug 2;8(8):CD013488. doi: 10.1002/14651858.CD013488.pub2. PMID: 34339047; PMCID: PMC8406465.

43: Lee SD, Kellow NJ, Choi TST, Huggins CE. Assessment of Dietary Acculturation in East Asian Populations: A Scoping Review. Adv Nutr. 2021 Jun 1;12(3):865-886. doi: 10.1093/advances/nmaa127. PMID: 33119743; PMCID: PMC8166541.

44: Kemp BJ, Thompson DR, Watson CJ, McGuigan K, Woodside JV, Ski CF. Effectiveness of family-based eHealth interventions in cardiovascular disease risk reduction: A systematic review. Prev Med. 2021 Aug;149:106608. doi: 10.1016/j.ypmed.2021.106608. Epub 2021 May 11. PMID: 33984372.

45: Jiang YW, Sun ZH, Tong WW, Yang K, Guo KQ, Liu G, Pan A. Dietary Intake and Circulating Concentrations of Carotenoids and Risk of Type 2 Diabetes: A Dose-Response Meta-Analysis of Prospective Observational Studies. Adv Nutr. 2021 Oct 1;12(5):1723-1733. doi: 10.1093/advances/nmab048. PMID: 33979433; PMCID: PMC8483954.

46: Yeh KL, Kautz A, Lohse B, Groth SW. Associations between Dietary Patterns and Inflammatory Markers during Pregnancy: A Systematic Review. Nutrients. 2021 Mar 4;13(3):834. doi: 10.3390/nu13030834. PMID: 33806342; PMCID: PMC8000934.

47: Norde MM, Collese TS, Giovannucci E, Rogero MM. A posteriori dietary patterns and their association with systemic low-grade inflammation in adults: a systematic review and meta-analysis. Nutr Rev. 2021 Feb 11;79(3):331-350. doi: 10.1093/nutrit/nuaa010. PMID: 32417914.

48: Hart MJ, Torres SJ, McNaughton SA, Milte CM. Dietary patterns and

associations with biomarkers of inflammation in adults: a systematic review of observational studies. Nutr J. 2021 Mar 12;20(1):24. doi: 10.1186/s12937-021-00674-9. PMID: 33712009; PMCID: PMC7955619.

49: Klonizakis M, Bugg A, Hunt B, Theodoridis X, Bogdanos DP, Grammatikopoulou MG. Assessing the Physiological Effects of Traditional Regional Diets Targeting the Prevention of Cardiovascular Disease: A Systematic Review of Randomized Controlled Trials Implementing Mediterranean, New Nordic, Japanese, Atlantic, Persian and Mexican Dietary Interventions. Nutrients. 2021 Aug 30;13(9):3034. doi: 10.3390/nu13093034. PMID: 34578911; PMCID: PMC8466163.

50: Christidis R, Lock M, Walker T, Egan M, Browne J. Concerns and priorities of Aboriginal and Torres Strait Islander peoples regarding food and nutrition: a systematic review of qualitative evidence. Int J Equity Health. 2021 Oct 7;20(1):220. doi: 10.1186/s12939-021-01551-x. PMID: 34620180; PMCID: PMC8499519.

51: Fiolet T, Mahamat-Saleh Y, Frenoy P, Kvaskoff M, Romana Mancini F. Background exposure to polychlorinated biphenyls and all-cause, cancer-specific, and cardiovascular-specific mortality: A systematic review and meta-analysis. Environ Int. 2021 Sep;154:106663. doi: 10.1016/j.envint.2021.106663. Epub 2021 May 31. PMID: 34082240.

52: Sinopoulou V, Gordon M, Akobeng AK, Gasparetto M, Sammaan M, Vasiliou J, Dovey TM. Interventions for the management of abdominal pain in Crohn's disease and inflammatory bowel disease. Cochrane Database Syst Rev. 2021 Nov 29;11(11):CD013531. doi: 10.1002/14651858.CD013531.pub2. PMID: 34844288; PMCID: PMC8629648.

53: Padhani ZA, Moazzam Z, Ashraf A, Bilal H, Salam RA, Das JK, Bhutta ZA. Vitamin C supplementation for prevention and treatment of pneumonia. Cochrane Database Syst Rev. 2021 Nov 18;11(11):CD013134. doi: 10.1002/14651858.CD013134.pub3. PMID: 34791642; PMCID: PMC8599445.

54: Lari A, Sohouli MH, Fatahi S, Cerqueira HS, Santos HO, Pourrajab B, Rezaei

M, Saneie S, Rahideh ST. The effects of the Dietary Approaches to Stop Hypertension (DASH) diet on metabolic risk factors in patients with chronic disease: A systematic review and meta-analysis of randomized controlled trials. Nutr Metab Cardiovasc Dis. 2021 Sep 22;31(10):2766-2778. doi: 10.1016/j.numecd.2021.05.030. Epub 2021 Jun 10. PMID: 34353704.

55: Mozaffari H, Hosseini Z, Lafrenière J, Conklin AI. The role of dietary diversity in preventing metabolic-related outcomes: Findings from a systematic review. Obes Rev. 2021 Jun;22(6):e13174. doi: 10.1111/obr.13174. Epub 2021 Feb 21. PMID: 33615679.

56: Silva-Santos T, Moreira P, Rodrigues M, Padrão P, Pinho O, Norton P, Ndrio A, Gonçalves C. Interventions That Successfully Reduced Adults Salt Intake-A Systematic Review. Nutrients. 2021 Dec 21;14(1):6. doi: 10.3390/nu14010006. PMID: 35010883; PMCID: PMC8746410.

57: Bjelakovic M, Nikolova D, Bjelakovic G, Gluud C. Vitamin D supplementation for chronic liver diseases in adults. Cochrane Database Syst Rev. 2021 Aug 25;8(8):CD011564. doi: 10.1002/14651858.CD011564.pub3. PMID: 34431511; PMCID: PMC8407054.

58: Joufi AI, Claiborne DM, Shuman D. Oral Health Education and Promotion Activities by Early Head Start Programs in the United States: A systematic review. J Dent Hyg. 2021 Oct;95(5):14-21. PMID: 34654711.

59: Billich N, Maugeri I, Calligaro L, Truby H, Davidson ZE. Weight management interventions that include dietary components for young people with chronic health care needs: A systematic review. Nutr Diet. 2021 Aug 8. doi: 10.1111/1747-0080.12698. Epub ahead of print. PMID: 34369055.

60: Quan J, Panaccione N, Jeong J, Underwood FE, Coward S, Windsor JW, Ronksley PE, Gidrewicz D, deBruyn J, Turner JM, Lebwohl B, Kaplan GG, King JA. Association Between Celiac Disease and Autism Spectrum Disorder: A Systematic Review. J Pediatr Gastroenterol Nutr. 2021 May 1;72(5):704-711. doi:

10.1097/MPG.0000000000003051. PMID: 33847288.

61: Głąbska D, Kołota A, Lachowicz K, Skolmowska D, Stachoń M, Guzek D. Vitamin D Supplementation and Mental Health in Inflammatory Bowel Diseases and Irritable Bowel Syndrome Patients: A Systematic Review. Nutrients. 2021 Oct 19;13(10):3662. doi: 10.3390/nu13103662. PMID: 34684663; PMCID: PMC8540769.

62: Song S, Stern Y, Gu Y. Modifiable lifestyle factors and cognitive reserve: A systematic review of current evidence. Ageing Res Rev. 2022 Feb;74:101551. doi: 10.1016/j.arr.2021.101551. Epub 2021 Dec 21. PMID: 34952208; PMCID: PMC8794051.

63: Schüz B, Meyerhof H, Hilz LK, Mata J. Equity Effects of Dietary Nudging Field Experiments: Systematic Review. Front Public Health. 2021 Jul 23;9:668998. doi: 10.3389/fpubh.2021.668998. PMID: 34368049; PMCID: PMC8342848.

64: Shoesmith A, Hall A, Wolfenden L, Shelton RC, Powell BJ, Brown H, McCrabb S, Sutherland R, Yoong S, Lane C, Booth D, Nathan N. Barriers and facilitators influencing the sustainment of health behaviour interventions in schools and childcare services: a systematic review. Implement Sci. 2021 Jun 12;16(1):62. doi: 10.1186/s13012-021-01134-y. PMID: 34118955; PMCID: PMC8199827.

65: Babashahi M, Omidvar N, Yazdizadeh B, Heidari-Beni M, Joulaei H, Narmcheshm S, Zargaraan A, Kelishadi R. Systematic review and meta-analysis of the most common processed foods consumed by Iranian children. East Mediterr Health J. 2021 Sep 21;27(9):918-930. doi: 10.26719/emhj.21.032. PMID: 34569048.

66: Baid D, Hayles E, Finkelstein EA. Return on Investment of Workplace Wellness Programs for Chronic Disease Prevention: A Systematic Review. Am J Prev Med. 2021 Aug;61(2):256-266. doi: 10.1016/j.amepre.2021.02.002. Epub 2021 May 5. PMID: 33965267.

67: Jayawardena R, Swarnamali H, Ranasinghe P, Misra A. Health effects of coconut oil: Summary of evidence from systematic reviews and meta-analysis of interventional studies. Diabetes Metab Syndr. 2021 Mar-Apr;15(2):549-555. doi:

10.1016/j.dsx.2021.02.032. Epub 2021 Mar 3. PMID: 33689936.

68: Cormick G, Ciapponi A, Cafferata ML, Cormick MS, Belizán JM. Calcium supplementation for prevention of primary hypertension. Cochrane Database Syst Rev. 2022 Jan 11;1(1):CD010037. doi: 10.1002/14651858.CD010037.pub4. PMID: 35014026; PMCID: PMC8748265.

69: Hanners A, Melnyk B, Volek J, Kelley MM. Ketogenic diet, African American women, and cardiovascular health: A systematic review. Worldviews Evid Based Nurs. 2022 Jan 19. doi: 10.1111/wvn.12561. Epub ahead of print. PMID: 35044076.

70: Nevins JEH, Donovan SM, Snetselaar L, Dewey KG, Novotny R, Stang J, Taveras EM, Kleinman RE, Bailey RL, Raghavan R, Scinto-Madonich SR, Venkatramanan S, Butera G, Terry N, Altman J, Adler M, Obbagy JE, Stoody EE, de Jesus J. Omega-3 Fatty Acid Dietary Supplements Consumed During Pregnancy and Lactation and Child Neurodevelopment: A Systematic Review. J Nutr. 2021 Nov 2;151(11):3483-3494. doi: 10.1093/jn/nxab238. PMID: 34383914; PMCID: PMC8764572.

71: Crane MM, Halloway S, Walts ZL, Gavin KL, Moss A, Westrick JC, Appelhans BM. Behavioural interventions for CVD risk reduction for blue-collar workers: a systematic review. J Epidemiol Community Health. 2021 Dec;75(12):1236-1243. doi: 10.1136/jech-2021-216515. Epub 2021 Jul 28. PMID: 34321281; PMCID: PMC8595631.

72: Bosetti R, Tabatabai L, Naufal G, Menser T, Kash B. Comprehensive cost-effectiveness of diabetes management for the underserved in the United States: A systematic review. PLoS One. 2021 Nov 18;16(11):e0260139. doi: 10.1371/journal.pone.0260139. PMID: 34793562; PMCID: PMC8601459.

73: Andraos S, Beck KL, Jones MB, Han TL, Conlon CA, de Seymour JV. Characterizing patterns of dietary exposure using metabolomic profiles of human biospecimens: a systematic review. Nutr Rev. 2022 Jan 12:nuab103. doi: 10.1093/nutrit/nuab103. Epub ahead of print. PMID: 35024860.

74: Fraiz GM, da Conceição AR, de Souza Vilela DL, Rocha DMUP, Bressan J,

Hermsdorff HHM. Can resveratrol modulate sirtuins in obesity and related diseases? A systematic review of randomized controlled trials. Eur J Nutr. 2021 Sep;60(6):2961-2977. doi: 10.1007/s00394-021-02623-y. Epub 2021 Jul 12. PMID: 34251517.

75: Baldwin C, de van der Schueren MA, Kruizenga HM, Weekes CE. Dietary advice with or without oral nutritional supplements for disease-related malnutrition in adults. Cochrane Database Syst Rev. 2021 Dec 21;12(12):CD002008. doi: 10.1002/14651858.CD002008.pub5. PMID: 34931696; PMCID: PMC8691169.

76: Dewey KG, Güngör D, Donovan SM, Madan EM, Venkatramanan S, Davis TA, Kleinman RE, Taveras EM, Bailey RL, Novotny R, Terry N, Butera G, Obbagy J, de Jesus J, Stoody E. Breastfeeding and risk of overweight in childhood and beyond: a systematic review with emphasis on sibling-pair and intervention studies. Am J Clin Nutr. 2021 Nov 8;114(5):1774-1790. doi: 10.1093/ajcn/nqab206. PMID: 34224561.

77: Smith C, Goss HR, Issartel J, Belton S. Health Literacy in Schools? A Systematic Review of Health-Related Interventions Aimed at Disadvantaged Adolescents. Children (Basel). 2021 Feb 25;8(3):176. doi: 10.3390/children8030176. PMID: 33668861; PMCID: PMC7996245.

78: Maksoud R, Balinas C, Holden S, Cabanas H, Staines D, Marshall-Gradisnik S. A systematic review of nutraceutical interventions for mitochondrial dysfunctions in myalgic encephalomyelitis/chronic fatigue syndrome. J Transl Med. 2021 Feb 17;19(1):81. doi: 10.1186/s12967-021-02742-4. PMID: 33596913; PMCID: PMC7890871.

79: Turner G, Green R, Alae-Carew C, Dangour AD. The association of dimensions of fruit and vegetable access in the retail food environment with consumption; a systematic review. Glob Food Sec. 2021 Jun;29:100528. doi: 10.1016/j.gfs.2021.100528. PMID: 34164256; PMCID: PMC8202327.

80: Vilela DLS, Fonseca PG, Pinto SL, Bressan J. Influence of dietary patterns

on the metabolically healthy obesity phenotype: A systematic review. Nutr Metab Cardiovasc Dis. 2021 Sep 22;31(10):2779-2791. doi: 10.1016/j.numecd.2021.05.007. Epub 2021 May 26. PMID: 34340900.

81: Glynn H, Möller SP, Wilding H, Apputhurai P, Moore G, Knowles SR. Prevalence and Impact of Post-traumatic Stress Disorder in Gastrointestinal Conditions: A Systematic Review. Dig Dis Sci. 2021 Dec;66(12):4109-4119. doi: 10.1007/s10620-020-06798-y. Epub 2021 Jan 12. PMID: 33433790.

82: Bondyra-Wiśniewska B, Myszkowska-Ryciak J, Harton A. Impact of Lifestyle Intervention Programs for Children and Adolescents with Overweight or Obesity on Body Weight and Selected Cardiometabolic Factors-A Systematic Review. Int J Environ Res Public Health. 2021 Feb 20;18(4):2061. doi: 10.3390/ijerph18042061. PMID: 33672502; PMCID: PMC7923753.

83: Matthews JJ, Dolan E, Swinton PA, Santos L, Artioli GG, Turner MD, Elliott-Sale KJ, Sale C. Effect of Carnosine or β-Alanine Supplementation on Markers of Glycemic Control and Insulin Resistance in Humans and Animals: A Systematic Review and Meta-analysis. Adv Nutr. 2021 Dec 1;12(6):2216-2231. doi: 10.1093/advances/nmab087. PMID: 34333586; PMCID: PMC8634390.

84: Moradi M, Sohrabi G, Golbidi M, Yarmohammadi S, Hemati N, Campbell MS, Moradi S, Kermani MAH, Farzaei MH. Effects of artichoke on blood pressure: A systematic review and meta-analysis. Complement Ther Med. 2021 Mar;57:102668. doi: 10.1016/j.ctim.2021.102668. Epub 2021 Jan 16. PMID: 33465383.

85: Chung M, Ruan M, Cara KC, Yao Q, Penkert LP, Chen J. Vitamin D and Calcium in Children 0-36 Months: A Scoping Review of Health Outcomes. J Am Coll Nutr. 2021 May-Jun;40(4):367-396. doi: 10.1080/07315724.2020.1774822. Epub 2020 Jul 14. PMID: 32662755.

86: Nishi SK, Viguiliouk E, Blanco Mejia S, Kendall CWC, Bazinet RP, Hanley AJ, Comelli EM, Salas Salvadó J, Jenkins DJA, Sievenpiper JL. Are fatty nuts a weighty concern? A systematic review and meta-analysis and dose-response meta-

regression of prospective cohorts and randomized controlled trials. Obes Rev. 2021 Nov;22(11):e13330. doi: 10.1111/obr.13330. Epub 2021 Sep 8. PMID: 34494363.

87: Emmert-Fees KMF, Karl FM, von Philipsborn P, Rehfuess EA, Laxy M. Simulation Modeling for the Economic Evaluation of Population-Based Dietary Policies: A Systematic Scoping Review. Adv Nutr. 2021 Oct 1;12(5):1957-1995. doi: 10.1093/advances/nmab028. PMID: 33873201; PMCID: PMC8483966.

88: Galekop MMJ, Uyl-de Groot CA, Ken Redekop W. A Systematic Review of Cost-Effectiveness Studies of Interventions With a Personalized Nutrition Component in Adults. Value Health. 2021 Mar;24(3):325-335. doi: 10.1016/j.jval.2020.12.006. Epub 2021 Feb 10. PMID: 33641765.

89: Ahmed S, Newton PD, Ojo O, Dibley L. Experiences of ethnic minority patients who are living with a primary chronic bowel condition: a systematic scoping review with narrative synthesis. BMC Gastroenterol. 2021 Aug 18;21(1):322. doi: 10.1186/s12876-021-01857-8. PMID: 34407752; PMCID: PMC8371833.

90: Wang MX, Gwee SXW, Pang J. Micronutrients Deficiency, Supplementation and Novel Coronavirus Infections-A Systematic Review and Meta-Analysis. Nutrients. 2021 May 10;13(5):1589. doi: 10.3390/nu13051589. PMID: 34068656; PMCID: PMC8151981.

91: Della Valle PG, Mosconi G, Nucci D, Vigezzi GP, Gentile L, Gianfredi V, Bonaccio M, Gianfagna F, Signorelli C, Iacoviello L, Odone A. Adherence to the Mediterranean Diet during the COVID-19 national lockdowns: a systematic review of observational studies. Acta Biomed. 2021 Oct 19;92(S6):e2021440. doi: 10.23750/abm.v92iS6.12233. PMID: 34739464.

92: Yin X, Liu H, Webster J, Trieu K, Huffman MD, Miranda JJ, Marklund M, Wu JHY, Cobb LK, Li KC, Pearson SA, Neal B, Tian M. Availability, Formulation, Labeling, and Price of Low-sodium Salt Worldwide: Environmental Scan. JMIR Public Health Surveill. 2021 Jul 14;7(7):e27423. doi: 10.2196/27423. PMID: 33985938; PMCID: PMC8319774.

93: Bakhtiary M, Morvaridzadeh M, Agah S, Rahimlou M, Christopher E, Zadro JR, Heshmati J. Effect of Probiotic, Prebiotic, and Synbiotic Supplementation on Cardiometabolic and Oxidative Stress Parameters in Patients With Chronic Kidney Disease: A Systematic Review and Meta-analysis. Clin Ther. 2021 Mar;43(3):e71-e96. doi: 10.1016/j.clinthera.2020.12.021. Epub 2021 Jan 30. PMID: 33526314.

94: Coelho-Júnior HJ, Trichopoulou A, Panza F. Cross-sectional and longitudinal associations between adherence to Mediterranean diet with physical performance and cognitive function in older adults: A systematic review and meta-analysis. Ageing Res Rev. 2021 Sep;70:101395. doi: 10.1016/j.arr.2021.101395. Epub 2021 Jun 19. PMID: 34153553.

95: Nartey EB, Spector J, Adu-Afarwuah S, Jones CL, Jackson A, Ohemeng A, Shah R, Koryo-Dabrah A, Kuma AB, Hyacinth HI, Steiner-Asiedu M. Nutritional perspectives on sickle cell disease in Africa: a systematic review. BMC Nutr. 2021 Mar 18;7(1):9. doi: 10.1186/s40795-021-00410-w. PMID: 33731225; PMCID: PMC7972183.

96: Hartono FA, Martin-Arrowsmith PW, Peeters WM, Churchward-Venne TA. The Effects of Dietary Protein Supplementation on Acute Changes in Muscle Protein Synthesis and Longer-Term Changes in Muscle Mass, Strength, and Aerobic Capacity in Response to Concurrent Resistance and Endurance Exercise in Healthy Adults: A Systematic Review. Sports Med. 2022 Feb 3. doi: 10.1007/s40279-021-01620-9. Epub ahead of print. PMID: 35113389.

97: McMahon EJ, Campbell KL, Bauer JD, Mudge DW, Kelly JT. Altered dietary salt intake for people with chronic kidney disease. Cochrane Database Syst Rev. 2021 Jun 24;6(6):CD010070. doi: 10.1002/14651858.CD010070.pub3. PMID: 34164803; PMCID: PMC8222708.

98: Brown TJ, Williams H, Mafrici B, Jackson HS, Johansson L, Willingham F, McIntosh A, MacLaughlin HL. Dietary interventions with dietitian involvement in

adults with chronic kidney disease: A systematic review. J Hum Nutr Diet. 2021 Aug;34(4):747-757. doi: 10.1111/jhn.12870. Epub 2021 Mar 8. PMID: 33682964.

99: Mihaescu A, Masood E, Zafran M, Khokhar HT, Augustine AM, Filippo A, Van Biesen W, Farrigton K, Carrero JJ, Covic A, Nistor I. Nutritional status improvement in elderly CKD patients: a systematic review. Int Urol Nephrol. 2021 Aug;53(8):1603-1621. doi: 10.1007/s11255-020-02775-6. Epub 2021 Jan 18. PMID: 33459956.

100: Trieu K, Bhat S, Dai Z, Leander K, Gigante B, Qian F, Korat AVA, Sun Q, Pan XF, Laguzzi F, Cederholm T, de Faire U, Hellénius ML, Wu JHY, Risérus U, Marklund M. Biomarkers of dairy fat intake, incident cardiovascular disease, and all-cause mortality: A cohort study, systematic review, and meta-analysis. PLoS Med. 2021 Sep 21;18(9):e1003763. doi: 10.1371/journal.pmed.1003763. PMID: 34547017; PMCID: PMC8454979.

101: Fang Y, Zhu J, Fan J, Sun L, Cai S, Fan C, Zhong Y, Li Y. Dietary Inflammatory Index in relation to bone mineral density, osteoporosis risk and fracture risk: a systematic review and meta-analysis. Osteoporos Int. 2021 Apr;32(4):633-643. doi: 10.1007/s00198-020-05578-8. Epub 2020 Aug 1. PMID: 32740669.

102: Henry Osokpo O, James R, Riegel B. Maintaining cultural identity: A systematic mixed studies review of cultural influences on the self-care of African immigrants living with non-communicable disease. J Adv Nurs. 2021 Sep;77(9):3600-3617. doi: 10.1111/jan.14804. Epub 2021 Feb 22. PMID: 33619819.

103: Duan Y, Shang B, Liang W, Du G, Yang M, Rhodes RE. Effects of eHealth-Based Multiple Health Behavior Change Interventions on Physical Activity, Healthy Diet, and Weight in People With Noncommunicable Diseases: Systematic Review and Meta-analysis. J Med Internet Res. 2021 Feb 22;23(2):e23786. doi: 10.2196/23786. PMID: 33616534; PMCID: PMC8074786.

104: Allanach JR, Farrell JW 3rd, Mésidor M, Karimi-Abdolrezaee S. Current

status of neuroprotective and neuroregenerative strategies in multiple sclerosis: A systematic review. Mult Scler. 2022 Jan;28(1):29-48. doi: 10.1177/13524585211008760. Epub 2021 Apr 19. PMID: 33870797; PMCID: PMC8688986.

105: Babu AF, Csader S, Lok J, Gómez-Gallego C, Hanhineva K, El-Nezami H, Schwab U. Positive Effects of Exercise Intervention without Weight Loss and Dietary Changes in NAFLD-Related Clinical Parameters: A Systematic Review and Meta-Analysis. Nutrients. 2021 Sep 8;13(9):3135. doi: 10.3390/nu13093135. PMID: 34579012; PMCID: PMC8466505.

106: Llanaj E, Dejanovic GM, Valido E, Bano A, Gamba M, Kastrati L, Minder B, Stojic S, Voortman T, Marques-Vidal P, Stoyanov J, Metzger B, Glisic M, Kern H, Muka T. Effect of oat supplementation interventions on cardiovascular disease risk markers: a systematic review and meta-analysis of randomized controlled trials. Eur J Nutr. 2022 Jan 3. doi: 10.1007/s00394-021-02763-1. Epub ahead of print. PMID: 34977959.

107: Becerra-Tomás N, Paz-Graniel I, Hernández-Alonso P, Jenkins DJA, Kendall CWC, Sievenpiper JL, Salas-Salvadó J. Nut consumption and type 2 diabetes risk: a systematic review and meta-analysis of observational studies. Am J Clin Nutr. 2021 Apr 6;113(4):960-971. doi: 10.1093/ajcn/nqaa358. PMID: 33471083.

108: Amiri M, Karabegović I, van Westing AC, Verkaar AJCF, Beigrezaei S, Lara M, Bramer WM, Voortman T. Whole-diet interventions and cardiovascular risk factors in postmenopausal women: A systematic review of controlled clinical trials. Maturitas. 2022 Jan;155:40-53. doi: 10.1016/j.maturitas.2021.10.001. Epub 2021 Oct 9. PMID: 34876248.

109: Ashtary-Larky D, Bagheri R, Tinsley GM, Asbaghi O, Salehpour S, Kashkooli S, Kooti W, Wong A. Betaine supplementation fails to improve body composition: a systematic review and meta-analysis. Br J Nutr. 2021 Oct 7:1-14. doi: 10.1017/S0007114521004062. Epub ahead of print. PMID: 34743773.

110: Brauer P, Royall D, Rodrigues A. Use of the Healthy Eating Index in

Intervention Studies for Cardiometabolic Risk Conditions: A Systematic Review. Adv Nutr. 2021 Jul 30;12(4):1317-1331. doi: 10.1093/advances/nmaa167. PMID: 33460430; PMCID: PMC8321868.

111: Bergwall S, Johansson A, Sonestedt E, Acosta S. High versus low-added sugar consumption for the primary prevention of cardiovascular disease. Cochrane Database Syst Rev. 2022 Jan 5;1(1):CD013320. doi: 10.1002/14651858.CD013320.pub2. PMID: 34986271; PMCID: PMC8730703.

112: Lin M, Heizati M, Wang L, Nurula M, Yang Z, Wang Z, Abudoyreyimu R, Wu Z, Li N. A systematic review and meta-analysis of effects of spironolactone on blood pressure, glucose, lipids, renal function, fibrosis and inflammation in patients with hypertension and diabetes. Blood Press. 2021 Jun;30(3):145-153. doi: 10.1080/08037051.2021.1880881. Epub 2021 Mar 8. PMID: 33682538.

113: Robinson K, Rozga M, Braakhuis A, Ellis A, Monnard CR, Sinley R, Wanner A, Vargas AJ. Effect of Incorporating Genetic Testing Results into Nutrition Counseling and Care on Dietary Intake: An Evidence Analysis Center Systematic Review-Part I. J Acad Nutr Diet. 2021 Mar;121(3):553-581.e3. doi: 10.1016/j.jand.2020.04.001. Epub 2020 Jul 3. PMID: 32624394.

114: Wang Y, Gallegos JL, Haskell-Ramsay C, Lodge JK. Effects of chronic consumption of specific fruit (berries, citrus and cherries) on CVD risk factors: a systematic review and meta-analysis of randomised controlled trials. Eur J Nutr. 2021 Mar;60(2):615-639. doi: 10.1007/s00394-020-02299-w. Epub 2020 Jun 13. Erratum in: Eur J Nutr. 2021 Jan 23;: PMID: 32535781; PMCID: PMC7900084.

115: Sinopoulou V, Gordon M, Dovey TM, Akobeng AK. Interventions for the management of abdominal pain in ulcerative colitis. Cochrane Database Syst Rev. 2021 Jul 22;7(7):CD013589. doi: 10.1002/14651858.CD013589.pub2. PMID: 34291816; PMCID: PMC8407332.

116: Heydenreich J, Schweter A, Lührmann P. Impact of physical activity, anthropometric, body composition, and dietary factors on bone stiffness in

German university students. J Sports Med Phys Fitness. 2021 Apr;61(4):571-581. doi: 10.23736/S0022-4707.20.11281-7. Epub 2020 Jul 30. PMID: 32744046.

117: Kim S, Park M, Song R. Effects of self-management programs on behavioral modification among individuals with chronic disease: A systematic review and meta-analysis of randomized trials. PLoS One. 2021 Jul 23;16(7):e0254995. doi: 10.1371/journal.pone.0254995. PMID: 34297741; PMCID: PMC8301623.

118: Shahinfar H, Jayedi A, Khan TA, Shab-Bidar S. Coffee consumption and cardiovascular diseases and mortality in patients with type 2 diabetes: A systematic review and dose-response meta-analysis of cohort studies. Nutr Metab Cardiovasc Dis. 2021 Aug 26;31(9):2526-2538. doi: 10.1016/j.numecd.2021.05.014. Epub 2021 May 24. PMID: 34112583.

119: Ribeiro FM, Lopes G, da Cunha Nascimento D, Pires L, Mulder AP, Franco OL, Petriz B. An overview of the level of dietary support in the gut microbiota at different stages of life: A systematic review. Clin Nutr ESPEN. 2021 Apr;42:41-52. doi: 10.1016/j.clnesp.2021.01.024. Epub 2021 Feb 9. PMID: 33745615.

120: Macey R, Walsh T, Riley P, Glenny AM, Worthington HV, Clarkson JE, Ricketts D. Electrical conductance for the detection of dental caries. Cochrane Database Syst Rev. 2021 Mar 16;3(3):CD014547. doi: 10.1002/14651858.CD014547. PMID: 33724442; PMCID: PMC8406820.

121: Djalalinia S, Hasani M, Asayesh H, Ejtahed HS, Malmir H, Kasaeian A, Zarei M, Baygi F, Rastad H, Mahdavi Gorabi A, Qorbani M. The effects of dietary selenium supplementation on inflammatory markers among patients with metabolic diseases: a systematic review and meta-analysis of randomized controlled trials. J Diabetes Metab Disord. 2021 Jun 9;20(1):1051-1062. doi: 10.1007/s40200-021-00821-3. PMID: 34222098; PMCID: PMC8212246.

122: Semlitsch T, Krenn C, Jeitler K, Berghold A, Horvath K, Siebenhofer A. Long-term effects of weight-reducing diets in people with hypertension. Cochrane

Database Syst Rev. 2021 Feb 8;2(2):CD008274. doi:
10.1002/14651858.CD008274.pub4. PMID: 33555049; PMCID: PMC8093137.

123: Bae S, Kamynina E, Guetterman HM, Farinola AF, Caudill MA, Berry RJ, Cassano PA, Stover PJ. Provision of folic acid for reducing arsenic toxicity in arsenic-exposed children and adults. Cochrane Database Syst Rev. 2021 Oct 18;10(10):CD012649. doi: 10.1002/14651858.CD012649.pub2. PMID: 34661903; PMCID: PMC8522704.

124: Naude CE, Brand A, Schoonees A, Nguyen KA, Chaplin M, Volmink J. Low-carbohydrate versus balanced-carbohydrate diets for reducing weight and cardiovascular risk. Cochrane Database Syst Rev. 2022 Jan 28;1(1):CD013334. doi: 10.1002/14651858.CD013334.pub2. PMID: 35088407; PMCID: PMC8795871.

125: Buzzetti E, Linden A, Best LM, Madden AM, Roberts D, Chase TJG, Freeman SC, Cooper NJ, Sutton AJ, Fritche D, Milne EJ, Wright K, Pavlov CS, Davidson BR, Tsochatzis E, Gurusamy KS. Lifestyle modifications for nonalcohol-related fatty liver disease: a network meta-analysis. Cochrane Database Syst Rev. 2021 Jun 11;6(6):CD013156. doi: 10.1002/14651858.CD013156.pub2. PMID: 34114650; PMCID: PMC8193812.

126: Karimi E, Bitarafan S, Mousavi SM, Zargarzadeh N, Mokhtari P, Hawkins J, Meysamie A, Koohdani F. The effect of vitamin D supplementation on fibroblast growth factor-23 in patients with chronic kidney disease: A systematic review and meta-analysis. Phytother Res. 2021 Oct;35(10):5339-5351. doi: 10.1002/ptr.7139. Epub 2021 Apr 30. PMID: 33928687.

127: Yao N, Yan S, Guo Y, Wang H, Li X, Wang L, Hu W, Li B, Cui W. The association between carotenoids and subjects with overweight or obesity: a systematic review and meta-analysis. Food Funct. 2021 Jun 8;12(11):4768-4782. doi: 10.1039/d1fo00004g. PMID: 33977977.

128: Pu Y, Zhu G, Xu Y, Zheng S, Tang B, Huang H, Wu IXY, Huang D, Liu Y, Zhang X. Association Between Vitamin D Exposure and Head and Neck Cancer: A Systematic

Review With Meta-Analysis. Front Immunol. 2021 Feb 23;12:627226. doi: 10.3389/fimmu.2021.627226. PMID: 33732250; PMCID: PMC7959800.

129: Vlassopoulou M, Yannakoulia M, Pletsa V, Zervakis GI, Kyriacou A. Effects of fungal beta-glucans on health - a systematic review of randomized controlled trials. Food Funct. 2021 Apr 26;12(8):3366-3380. doi: 10.1039/d1fo00122a. PMID: 33876798.

130: Dorelli B, Gallè F, De Vito C, Duranti G, Iachini M, Zaccarin M, Preziosi Standoli J, Ceci R, Romano F, Liguori G, Romano Spica V, Sabatini S, Valeriani F, Cattaruzza MS. Can Physical Activity Influence Human Gut Microbiota Composition Independently of Diet? A Systematic Review. Nutrients. 2021 May 31;13(6):1890. doi: 10.3390/nu13061890. PMID: 34072834; PMCID: PMC8228232.

131: Dewsbury LS, Lim CK, Steiner GZ. The Efficacy of Ketogenic Therapies in the Clinical Management of People with Neurodegenerative Disease: A Systematic Review. Adv Nutr. 2021 Jul 30;12(4):1571-1593. doi: 10.1093/advances/nmaa180. PMID: 33621313; PMCID: PMC8321843.

132: Hutton GB, Brugulat-Panés A, Bhagtani D, Mba Maadjhou C, Birch JM, Shih H, Okop K, Muti M, Wadende P, Tatah L, Mogo E, Guariguata L, Unwin N. A Systematic Scoping Review of the Impacts of Community Food Production Initiatives in Kenya, Cameroon, and South Africa. J Glob Health Rep. 2021 Mar 24;5:e2021010. doi: 10.29392/001c.19468. PMID: 33829114; PMCID: PMC7610539.

133: Wagnild JM, Pollard TM. How is television time linked to cardiometabolic health in adults? A critical systematic review of the evidence for an effect of watching television on eating, movement, affect and sleep. BMJ Open. 2021 May 5;11(5):e040739. doi: 10.1136/bmjopen-2020-040739. PMID: 33952532; PMCID: PMC8103379.

134: Yazdanpanah Z, Beigrezaei S, Mohseni-Takalloo S, Soltani S, Rajaie SH, Zohrabi T, Kaviani M, Forbes SC, Baker JS, Salehi-Abargouei A. Does exercise affect bone mineral density and content when added to a calorie-restricted diet?

A systematic review and meta-analysis of controlled clinical trials. Osteoporos Int. 2022 Feb;33(2):339-354. doi: 10.1007/s00198-021-06187-9. Epub 2021 Oct 13. PMID: 34643754.

135: Kazmierski KFM, Gillespie ML, Kuo S, Zurita T, Felix D, Rao U. Stress-Induced Eating Among Racial/Ethnic Groups in the United States: a Systematic Review. J Racial Ethn Health Disparities. 2021 Aug;8(4):912-926. doi: 10.1007/s40615-020-00849-w. Epub 2020 Aug 24. PMID: 32839895; PMCID: PMC7902740.

136: Kazemi M, Kim JY, Parry SA, Azziz R, Lujan ME. Disparities in cardio metabolic risk between Black and White women with polycystic ovary syndrome: a systematic review and meta-analysis. Am J Obstet Gynecol. 2021 May;224(5):428-444.e8. doi: 10.1016/j.ajog.2020.12.019. Epub 2020 Dec 13. PMID: 33316275.

137: Vanoni FO, Milani GP, Agostoni C, Treglia G, Faré PB, Camozzi P, Lava SAG, Bianchetti MG, Janett S. Magnesium Metabolism in Chronic Alcohol-Use Disorder: Meta-Analysis and Systematic Review. Nutrients. 2021 Jun 7;13(6):1959. doi: 10.3390/nu13061959. PMID: 34200366; PMCID: PMC8229336.

138: Komolafe O, Buzzetti E, Linden A, Best LM, Madden AM, Roberts D, Chase TJ, Fritche D, Freeman SC, Cooper NJ, Sutton AJ, Milne EJ, Wright K, Pavlov CS, Davidson BR, Tsochatzis E, Gurusamy KS. Nutritional supplementation for nonalcohol-related fatty liver disease: a network meta-analysis. Cochrane Database Syst Rev. 2021 Jul 19;7(7):CD013157. doi: 10.1002/14651858.CD013157.pub2. PMID: 34280304; PMCID: PMC8406904.

139: Cicero AFG, Kennedy C, Knežević T, Bove M, Georges CMG, Šatrauskienė A, Toth PP, Fogacci F. Efficacy and Safety of Armolipid Plus®: An Updated PRISMA Compliant Systematic Review and Meta-Analysis of Randomized Controlled Clinical Trials. Nutrients. 2021 Feb 16;13(2):638. doi: 10.3390/nu13020638. PMID: 33669333; PMCID: PMC7920267.

140: Grammatikopoulou MG, Gkiouras K, Polychronidou G, Kaparounaki C, Gkouskou

KK, Magkos F, Donini LM, Eliopoulos AG, Goulis DG. Obsessed with Healthy Eating: A Systematic Review of Observational Studies Assessing Orthorexia Nervosa in Patients with Diabetes Mellitus. Nutrients. 2021 Oct 27;13(11):3823. doi: 10.3390/nu13113823. PMID: 34836080; PMCID: PMC8622186.

141: Gold N, Yau A, Rigby B, Dyke C, Remfry EA, Chadborn T. Effectiveness of Digital Interventions for Reducing Behavioral Risks of Cardiovascular Disease in Nonclinical Adult Populations: Systematic Review of Reviews. J Med Internet Res. 2021 May 14;23(5):e19688. doi: 10.2196/19688. PMID: 33988126; PMCID: PMC8164125.

142: Moodi V, Abedi S, Esmaeilpour M, Asbaghi O, Izadi F, Shirinbakhshmasoleh M, Behrouzian M, Shahriari A, Ghaedi E, Miraghajani M. The effect of grapes/grape products on glycemic response: A systematic review and meta-analysis of randomized controlled trials. Phytother Res. 2021 Sep;35(9):5053-5067. doi: 10.1002/ptr.7135. Epub 2021 Apr 24. PMID: 33893683.

143: Cabalín C, Iturriaga C, Pérez-Mateluna G, Echeverría D, Camargo CA Jr, Borzutzky A. Vitamin D status and supplementation in Antarctica: a systematic review and meta- analysis. Int J Circumpolar Health. 2021 Dec;80(1):1926133. doi: 10.1080/22423982.2021.1926133. PMID: 33983101; PMCID: PMC8128169.

144: Williams GM, Tapsell LC, O'Brien CL, Tosh SM, Barrett EM, Beck EJ. Gut microbiome responses to dietary intake of grain-based fibers with the potential to modulate markers of metabolic disease: a systematic literature review. Nutr Rev. 2021 Oct 11;79(11):1274-1292. doi: 10.1093/nutrit/nuaa128. PMID: 33369654.

145: Hasani M, Mansour A, Asayesh H, Djalalinia S, Mahdavi Gorabi A, Ochi F, Qorbani M. Effect of glutamine supplementation on cardiometabolic risk factors and inflammatory markers: a systematic review and meta-analysis. BMC Cardiovasc Disord. 2021 Apr 17;21(1):190. doi: 10.1186/s12872-021-01986-8. PMID: 33865313; PMCID: PMC8053267.

146: Wiebe N, Ye F, Crumley ET, Bello A, Stenvinkel P, Tonelli M. Temporal Associations Among Body Mass Index, Fasting Insulin, and Systemic Inflammation:

A Systematic Review and Meta-analysis. JAMA Netw Open. 2021 Mar 1;4(3):e211263. doi: 10.1001/jamanetworkopen.2021.1263. PMID: 33710289; PMCID: PMC7955272.

147: Whitley A, Yahia N. Efficacy of Clinic-Based Telehealth vs. Face-to-Face Interventions for Obesity Treatment in Children and Adolescents in the United States and Canada: A Systematic Review. Child Obes. 2021 Jul;17(5):299-310. doi: 10.1089/chi.2020.0347. Epub 2021 Apr 29. PMID: 33926238.

148: Akhavan Zanjani M, Rahmani S, Mehranfar S, Zarrin M, Bazyar H, Moradi Poodeh B, Zare Javid A, Hosseini SA, Sadeghian M. Soy Foods and the Risk of Fracture: A Systematic Review of Prospective Cohort Studies. Complement Med Res. 2021 Sep 21:1-10. English. doi: 10.1159/000519036. Epub ahead of print. PMID: 34547749.

149: Asbaghi O, Moradi S, Nezamoleslami S, Moosavian SP, Hojjati Kermani MA, Lazaridi AV, Miraghajani M. The Effects of Magnesium Supplementation on Lipid Profile Among Type 2 Diabetes Patients: a Systematic Review and Meta-analysis of Randomized Controlled Trials. Biol Trace Elem Res. 2021 Mar;199(3):861-873. doi: 10.1007/s12011-020-02209-5. Epub 2020 May 28. PMID: 32468224.

150: Perna S, Ilyas Z, Giacosa A, Gasparri C, Peroni G, Faliva MA, Rigon C, Naso M, Riva A, Petrangolini G, A Redha A, Rondanelli M. Is Probiotic Supplementation Useful for the Management of Body Weight and Other Anthropometric Measures in Adults Affected by Overweight and Obesity with Metabolic Related Diseases? A Systematic Review and Meta-Analysis. Nutrients. 2021 Feb 19;13(2):666. doi: 10.3390/nu13020666. PMID: 33669580; PMCID: PMC7922558.

151: Pereira RO, Correia LA, Farah D, Komoni G, Farah V, Fiorino P. Wistar rat as an animal model to study high-fat induced kidney damage: a systematic review. Arch Physiol Biochem. 2021 Dec 16:1-10. doi: 10.1080/13813455.2021.2017462. Epub ahead of print. PMID: 34915796.

152: Beaulieu K, Blundell JE, van Baak MA, Battista F, Busetto L, Carraça EV, Dicker D, Encantado J, Ermolao A, Farpour-Lambert N, Pramono A, Woodward E,

Bellicha A, Oppert JM. Effect of exercise training interventions on energy intake and appetite control in adults with overweight or obesity: A systematic review and meta-analysis. Obes Rev. 2021 Jul;22 Suppl 4(Suppl 4):e13251. doi: 10.1111/obr.13251. Epub 2021 May 5. PMID: 33949089; PMCID: PMC8365695.

153: He J, Kong D, Yang Z, Guo R, Amponsah AE, Feng B, Zhang X, Zhang W, Liu A, Ma J, O'Brien T, Cui H. Clinical efficacy on glycemic control and safety of mesenchymal stem cells in patients with diabetes mellitus: Systematic review and meta-analysis of RCT data. PLoS One. 2021 Mar 11;16(3):e0247662. doi: 10.1371/journal.pone.0247662. PMID: 33705413; PMCID: PMC7951834.

154: Nkambule SJ, Moodley I, Kuupiel D, Mashamba-Thompson TP. Association between food insecurity and key metabolic risk factors for diet-sensitive non-communicable diseases in sub-Saharan Africa: a systematic review and meta-analysis. Sci Rep. 2021 Mar 4;11(1):5178. doi: 10.1038/s41598-021-84344-0. PMID: 33664339; PMCID: PMC7933340.

155: Vahedian-Azimi A, Abbasifard M, Rahimi-Bashar F, Guest PC, Majeed M, Mohammadi A, Banach M, Jamialahmadi T, Sahebkar A. Effectiveness of Curcumin on Outcomes of Hospitalized COVID-19 Patients: A Systematic Review of Clinical Trials. Nutrients. 2022 Jan 7;14(2):256. doi: 10.3390/nu14020256. PMID: 35057437; PMCID: PMC8779570.

156: Iyer P, Beck EJ, Walton KL. A systematic review of the effect of dietary interventions on cardiovascular disease risk in adults with spinal cord injury. J Spinal Cord Med. 2021 Mar;44(2):184-203. doi: 10.1080/10790268.2019.1592926. Epub 2019 Apr 4. PMID: 30945998; PMCID: PMC7952075.

157: Andueza N, Navas-Carretero S, Cuervo M. Effectiveness of Nutritional Strategies on Improving the Quality of Diet of Children from 6 to 12 Years Old: A Systematic Review. Nutrients. 2022 Jan 15;14(2):372. doi: 10.3390/nu14020372. PMID: 35057552; PMCID: PMC8781853.

158: Conley MM, McFarlane CM, Johnson DW, Kelly JT, Campbell KL, MacLaughlin HL.

Interventions for weight loss in people with chronic kidney disease who are overweight or obese. Cochrane Database Syst Rev. 2021 Mar 30;3(3):CD013119. doi: 10.1002/14651858.CD013119.pub2. PMID: 33782940; PMCID: PMC8094234.

159: Kirkham AA, Beka V, Prado CM. The effect of caloric restriction on blood pressure and cardiovascular function: A systematic review and meta-analysis of randomized controlled trials. Clin Nutr. 2021 Mar;40(3):728-739. doi: 10.1016/j.clnu.2020.06.029. Epub 2020 Jul 1. PMID: 32675017.

160: Schönenberger KA, Schüpfer AC, Gloy VL, Hasler P, Stanga Z, Kaegi-Braun N, Reber E. Effect of Anti-Inflammatory Diets on Pain in Rheumatoid Arthritis: A Systematic Review and Meta-Analysis. Nutrients. 2021 Nov 24;13(12):4221. doi: 10.3390/nu13124221. PMID: 34959772; PMCID: PMC8706441.

161: Santos JA, Tekle D, Rosewarne E, Flexner N, Cobb L, Al-Jawaldeh A, Kim WJ, Breda J, Whiting S, Campbell N, Neal B, Webster J, Trieu K. A Systematic Review of Salt Reduction Initiatives Around the World: A Midterm Evaluation of Progress Towards the 2025 Global Non-Communicable Diseases Salt Reduction Target. Adv Nutr. 2021 Oct 1;12(5):1768-1780. doi: 10.1093/advances/nmab008. PMID: 33693460; PMCID: PMC8483946.

162: Asbaghi O, Ashtary-Larky D, Bagheri R, Moosavian SP, Olyaei HP, Nazarian B, Rezaei Kelishadi M, Wong A, Candow DG, Dutheil F, Suzuki K, Alavi Naeini A. Folic Acid Supplementation Improves Glycemic Control for Diabetes Prevention and Management: A Systematic Review and Dose-Response Meta-Analysis of Randomized Controlled Trials. Nutrients. 2021 Jul 9;13(7):2355. doi: 10.3390/nu13072355. PMID: 34371867; PMCID: PMC8308657.

163: Ebrahimzadeh A, Abbasi F, Ebrahimzadeh A, Jibril AT, Milajerdi A. Effects of curcumin supplementation on inflammatory biomarkers in patients with Rheumatoid Arthritis and Ulcerative colitis: A systematic review and meta-analysis. Complement Ther Med. 2021 Sep;61:102773. doi: 10.1016/j.ctim.2021.102773. Epub 2021 Aug 31. PMID: 34478838.

164: Lam CN, Watt AE, Isenring EA, de van der Schueren MAE, van der Meij BS. The effect of oral omega-3 polyunsaturated fatty acid supplementation on muscle maintenance and quality of life in patients with cancer: A systematic review and meta-analysis. Clin Nutr. 2021 Jun;40(6):3815-3826. doi: 10.1016/j.clnu.2021.04.031. Epub 2021 Apr 27. PMID: 34130028.

165: Mishu MP, Uphoff E, Aslam F, Philip S, Wright J, Tirbhowan N, Ajjan RA, Al Azdi Z, Stubbs B, Churchill R, Siddiqi N. Interventions for preventing type 2 diabetes in adults with mental disorders in low- and middle-income countries. Cochrane Database Syst Rev. 2021 Feb 16;2(2):CD013281. doi: 10.1002/14651858.CD013281.pub2. PMID: 33591592; PMCID: PMC8092639.

166: Mohammad A, Falahi E, Mohd Yusof BN, Hanipah ZN, Sabran MR, Mohamad Yusof L, Gheitasvand M. The effects of the ginger supplements on inflammatory parameters in type 2 diabetes patients: A systematic review and meta-analysis of randomised controlled trials. Clin Nutr ESPEN. 2021 Dec;46:66-72. doi: 10.1016/j.clnesp.2021.10.013. Epub 2021 Oct 22. PMID: 34857250.

167: Jahrami HA, Faris ME, I Janahi A, I Janahi M, Abdelrahim DN, Madkour MI, Sater MS, Hassan AB, Bahammam AS. Does four-week consecutive, dawn-to-sunset intermittent fasting during Ramadan affect cardiometabolic risk factors in healthy adults? A systematic review, meta-analysis, and meta-regression. Nutr Metab Cardiovasc Dis. 2021 Jul 22;31(8):2273-2301. doi: 10.1016/j.numecd.2021.05.002. Epub 2021 May 25. PMID: 34167865.

168: Ahmed S, Innes JK, Calder PC. Influence of different intravenous lipid emulsions on fatty acid status and laboratory and clinical outcomes in adult patients receiving home parenteral nutrition: A systematic review. Clin Nutr. 2021 Mar;40(3):1115-1122. doi: 10.1016/j.clnu.2020.07.014. Epub 2020 Jul 23. PMID: 32758383.

169: Rodrigues C, Pinto A, Faria A, Teixeira D, van Wegberg AMJ, Ahring K, Feillet F, Calhau C, MacDonald A, Moreira-Rosário A, Rocha JC. Is the Phenylalanine-Restricted Diet a Risk Factor for Overweight or Obesity in

Patients with Phenylketonuria (PKU)? A Systematic Review and Meta-Analysis. Nutrients. 2021 Sep 28;13(10):3443. doi: 10.3390/nu13103443. PMID: 34684443; PMCID: PMC8538431.

170: Asbaghi O, Ashtary-Larky D, Bagheri R, Moosavian SP, Nazarian B, Afrisham R, Kelishadi MR, Wong A, Dutheil F, Suzuki K, Alavi Naeini A. Effects of Folic Acid Supplementation on Inflammatory Markers: A Grade-Assessed Systematic Review and Dose-Response Meta-Analysis of Randomized Controlled Trials. Nutrients. 2021 Jul 6;13(7):2327. doi: 10.3390/nu13072327. PMID: 34371837; PMCID: PMC8308638.

171: Zhang Y, Lu Y, Wang S, Yang L, Xia H, Sun G. Excessive Vitamin A Supplementation Increased the Incidence of Acute Respiratory Tract Infections: A Systematic Review and Meta-Analysis. Nutrients. 2021 Nov 26;13(12):4251. doi: 10.3390/nu13124251. PMID: 34959803; PMCID: PMC8706818.
