# Supplementary material for: Perspectives of Dietary Assessment in Human Health and Disease
Source: Nutrients. 2022 Feb 16;14(4):830. doi: 10.3390/nu14040830 (PMC8877528; doi:10.3390/nu14040830)
Supplement: Supplementary file 1 [file nutrients-14-00830-s001.zip › Table S6.pdf]

Table S6 - PubMed search keywords "dietary assessment human health disease", filters "1 year" and "meta-analysis"

starting date 08/02/2022

Type of article: Meta-analysis

n = 92

1: Bolte LA, Vich Vila A, Imhann F, Collij V, Gacesa R, Peters V, Wijmenga C, Kurilshikov A, Campmans-Kuijpers MJE, Fu J, Dijkstra G, Zhernakova A, Weersma RK. Long-term dietary patterns are associated with pro-inflammatory and anti-inflammatory features of the gut microbiome. Gut. 2021 Jul;70(7):1287-1298. doi: 10.1136/gutjnl-2020-322670. Epub 2021 Apr 2. PMID: 33811041; PMCID: PMC8223641.

2: Bassatne A, Basbous M, Chakhtoura M, El Zein O, Rahme M, El-Hajj Fuleihan G. The link between COVID-19 and VItamin D (VIVID): A systematic review and meta-analysis. Metabolism. 2021 Jun;119:154753. doi: 10.1016/j.metabol.2021.154753. Epub 2021 Mar 24. PMID: 33774074; PMCID: PMC7989070.

3: Wang DD, Li Y, Bhupathiraju SN, Rosner BA, Sun Q, Giovannucci EL, Rimm EB, Manson JE, Willett WC, Stampfer MJ, Hu FB. Fruit and Vegetable Intake and Mortality: Results From 2 Prospective Cohort Studies of US Men and Women and a Meta-Analysis of 26 Cohort Studies. Circulation. 2021 Apr 27;143(17):1642-1654. doi: 10.1161/CIRCULATIONAHA.120.048996. Epub 2021 Mar 1. PMID: 33641343; PMCID: PMC8084888.

4: Chiavaroli L, Lee D, Ahmed A, Cheung A, Khan TA, Blanco S, Mejia, Mirrahimi A, Jenkins DJA, Livesey G, Wolever TMS, Rahelić D, Kahleová H, Salas-Salvadó J, Kendall CWC, Sievenpiper JL. Effect of low glycaemic index or load dietary patterns on glycaemic control and cardiometabolic risk factors in diabetes: systematic review and meta-analysis of randomised controlled trials. BMJ. 2021 Aug 4;374:n1651. doi: 10.1136/bmj.n1651. Erratum in: BMJ. 2021 Aug 26;374:n2114. PMID: 34348965; PMCID: PMC8336013.

5: Naghshi S, Aune D, Beyene J, Mobarak S, Asadi M, Sadeghi O. Dietary intake and biomarkers of alpha linolenic acid and risk of all cause, cardiovascular, and cancer mortality: systematic review and dose-response meta-analysis of

cohort studies. BMJ. 2021 Oct 13;375:n2213. doi: 10.1136/bmj.n2213. PMID: 34645650; PMCID: PMC8513503.

6: Godos J, Micek A, Brzostek T, Toledo E, Iacoviello L, Astrup A, Franco OH, Galvano F, Martinez-Gonzalez MA, Grosso G. Egg consumption and cardiovascular risk: a dose-response meta-analysis of prospective cohort studies. Eur J Nutr. 2021 Jun;60(4):1833-1862. doi: 10.1007/s00394-020-02345-7. Epub 2020 Aug 31. PMID: 32865658; PMCID: PMC8137614.

7: Deane KHO, Jimoh OF, Biswas P, O'Brien A, Hanson S, Abdelhamid AS, Fox C, Hooper L. Omega-3 and polyunsaturated fat for prevention of depression and anxiety symptoms: systematic review and meta-analysis of randomised trials. Br J Psychiatry. 2021 Mar;218(3):135-142. doi: 10.1192/bjp.2019.234. PMID: 31647041.

8: Fenton S, Burrows TL, Skinner JA, Duncan MJ. The influence of sleep health on dietary intake: a systematic review and meta-analysis of intervention studies. J Hum Nutr Diet. 2021 Apr;34(2):273-285. doi: 10.1111/jhn.12813. Epub 2020 Oct 1. PMID: 33001515.

9: Hassani Zadeh S, Mansoori A, Hosseinzadeh M. Relationship between dietary patterns and non-alcoholic fatty liver disease: A systematic review and meta-analysis. J Gastroenterol Hepatol. 2021 Jun;36(6):1470-1478. doi: 10.1111/jgh.15363. Epub 2020 Dec 14. PMID: 33269500.

10: Ajabnoor SM, Thorpe G, Abdelhamid A, Hooper L. Long-term effects of increasing omega-3, omega-6 and total polyunsaturated fats on inflammatory bowel disease and markers of inflammation: a systematic review and meta-analysis of randomized controlled trials. Eur J Nutr. 2021 Aug;60(5):2293-2316. doi: 10.1007/s00394-020-02413-y. Epub 2020 Oct 21. PMID: 33084958.

11: Rees K, Al-Khudairy L, Takeda A, Stranges S. Vegan dietary pattern for the primary and secondary prevention of cardiovascular diseases. Cochrane Database Syst Rev. 2021 Feb 25;2(2):CD013501. doi: 10.1002/14651858.CD013501.pub2. PMID: 33629376; PMCID: PMC8092640.

12: Whittaker J, Wu K. Low-fat diets and testosterone in men: Systematic review and meta-analysis of intervention studies. J Steroid Biochem Mol Biol. 2021 Jun;210:105878. doi: 10.1016/j.jsbmb.2021.105878. Epub 2021 Mar 16. PMID: 33741447.

13: Fatemeh G, Sajjad M, Niloufar R, Neda S, Leila S, Khadijeh M. Effect of melatonin supplementation on sleep quality: a systematic review and meta-analysis of randomized controlled trials. J Neurol. 2022 Jan;269(1):205-216. doi: 10.1007/s00415-020-10381-w. Epub 2021 Jan 8. PMID: 33417003.

14: Schwab U, Reynolds AN, Sallinen T, Rivellese AA, Risérus U. Dietary fat intakes and cardiovascular disease risk in adults with type 2 diabetes: a systematic review and meta-analysis. Eur J Nutr. 2021 Sep;60(6):3355-3363. doi: 10.1007/s00394-021-02507-1. Epub 2021 Feb 21. PMID: 33611616.

15: Kim Y, Je Y, Giovannucci EL. Association between dietary fat intake and mortality from all-causes, cardiovascular disease, and cancer: A systematic review and meta-analysis of prospective cohort studies. Clin Nutr. 2021 Mar;40(3):1060-1070. doi: 10.1016/j.clnu.2020.07.007. Epub 2020 Jul 14. PMID: 32723506.

16: Low JHM, Toh DWK, Ng MTT, Fam J, Kua EH, Kim JE. A Systematic Review and Meta-Analysis of the Impact of Different Intensity of Dietary Counselling on Cardiometabolic Health in Middle-Aged and Older Adults. Nutrients. 2021 Aug 25;13(9):2936. doi: 10.3390/nu13092936. PMID: 34578814; PMCID: PMC8469488.

17: Peñalvo JL, Sagastume D, Mertens E, Uzhova I, Smith J, Wu JHY, Bishop E, Onopa J, Shi P, Micha R, Mozaffarian D. Effectiveness of workplace wellness programmes for dietary habits, overweight, and cardiometabolic health: a systematic review and meta-analysis. Lancet Public Health. 2021 Sep;6(9):e648-e660. doi: 10.1016/S2468-2667(21)00140-7. PMID: 34454642; PMCID: PMC8627548.

18: McKenzie BL, Coyle DH, Santos JA, Burrows T, Rosewarne E, Peters SAE, Carcel C, Jaacks LM, Norton R, Collins CE, Woodward M, Webster J. Investigating sex differences in the accuracy of dietary assessment methods to measure energy intake in adults: a systematic review and meta-analysis. Am J Clin Nutr. 2021 May 8;113(5):1241-1255. doi: 10.1093/ajcn/nqaa370. PMID: 33564834; PMCID: PMC8106762.

19: Garcêz LS, Avelar CR, Fonseca NSS, Costa PRF, Lyra AC, Cunha CM, Jesus RP, Oliveira LPM. Effect of dietary carbohydrate and lipid modification on clinical and anthropometric parameters in nonalcoholic fatty liver disease: a systematic review and meta-analysis. Nutr Rev. 2021 Nov 10;79(12):1321-1337. doi: 10.1093/nutrit/nuaa146. PMID: 33515021.

20: Ooi JY, Wolfenden L, Sutherland R, Nathan N, Oldmeadow C, Mclaughlin M, Barnes C, Hall A, Vanderlee L, Yoong SL. A Systematic Review of the Recent Consumption Levels of Sugar-Sweetened Beverages in Children and Adolescents From the World Health Organization Regions With High Dietary-Related Burden of Disease. Asia Pac J Public Health. 2022 Jan;34(1):11-24. doi: 10.1177/10105395211014642. Epub 2021 May 20. PMID: 34013784.

21: Ba DM, Gao X, Al-Shaar L, Muscat J, Chinchilli VM, Ssentongo P, Zhang X, Liu G, Beelman RB, Richie JP Jr. Prospective study of dietary mushroom intake and risk of mortality: results from continuous National Health and Nutrition Examination Survey (NHANES) 2003-2014 and a meta-analysis. Nutr J. 2021 Sep 21;20(1):80. doi: 10.1186/s12937-021-00738-w. PMID: 34548082; PMCID: PMC8454070.

22: Jolliffe DA, Camargo CA Jr, Sluyter JD, Aglipay M, Aloia JF, Ganmaa D, Bergman P, Bischoff-Ferrari HA, Borzutzky A, Damsgaard CT, Dubnov-Raz G, Esposito S, Gilham C, Ginde AA, Golan-Tripto I, Goodall EC, Grant CC, Griffiths CJ, Hibbs AM, Janssens W, Khadilkar AV, Laaksi I, Lee MT, Loeb M, Maguire JL, Majak P, Mauger DT, Manaseki-Holland S, Murdoch DR, Nakashima A, Neale RE, Pham H, Rake C, Rees JR, Rosendahl J, Scragg R, Shah D, Shimizu Y, Simpson-Yap S, Trilok-Kumar G, Urashima M, Martineau AR. Vitamin D supplementation to prevent acute respiratory infections: a systematic review and meta-analysis of aggregate

data from randomised controlled trials. Lancet Diabetes Endocrinol. 2021 May;9(5):276-292. doi: 10.1016/S2213-8587(21)00051-6. Epub 2021 Mar 30. PMID: 33798465.

23: Li N, Wu X, Zhuang W, Xia L, Chen Y, Wang Y, Wu C, Rao Z, Du L, Zhao R, Yi M, Wan Q, Zhou Y. Green leafy vegetable and lutein intake and multiple health outcomes. Food Chem. 2021 Oct 30;360:130145. doi: 10.1016/j.foodchem.2021.130145. Epub 2021 May 18. PMID: 34034049.

24: Jiang YW, Sun ZH, Tong WW, Yang K, Guo KQ, Liu G, Pan A. Dietary Intake and Circulating Concentrations of Carotenoids and Risk of Type 2 Diabetes: A Dose-Response Meta-Analysis of Prospective Observational Studies. Adv Nutr. 2021 Oct 1;12(5):1723-1733. doi: 10.1093/advances/nmab048. PMID: 33979433; PMCID: PMC8483954.

25: Norde MM, Collese TS, Giovannucci E, Rogero MM. A posteriori dietary patterns and their association with systemic low-grade inflammation in adults: a systematic review and meta-analysis. Nutr Rev. 2021 Feb 11;79(3):331-350. doi: 10.1093/nutrit/nuaa010. PMID: 32417914.

26: Fiolet T, Mahamat-Saleh Y, Frenoy P, Kvaskoff M, Romana Mancini F. Background exposure to polychlorinated biphenyls and all-cause, cancer-specific, and cardiovascular-specific mortality: A systematic review and meta-analysis. Environ Int. 2021 Sep;154:106663. doi: 10.1016/j.envint.2021.106663. Epub 2021 May 31. PMID: 34082240.

27: Lari A, Sohouli MH, Fatahi S, Cerqueira HS, Santos HO, Pourrajab B, Rezaei M, Saneie S, Rahideh ST. The effects of the Dietary Approaches to Stop Hypertension (DASH) diet on metabolic risk factors in patients with chronic disease: A systematic review and meta-analysis of randomized controlled trials. Nutr Metab Cardiovasc Dis. 2021 Sep 22;31(10):2766-2778. doi: 10.1016/j.numecd.2021.05.030. Epub 2021 Jun 10. PMID: 34353704.

28: Guo F, Zhang Q, Jiang H, He Y, Li M, Ran J, Lin J, Tian L, Ma L. Dietary

potato intake and risks of type 2 diabetes and gestational diabetes mellitus. Clin Nutr. 2021 Jun;40(6):3754-3764. doi: 10.1016/j.clnu.2021.04.039. Epub 2021 May 1. PMID: 34130021.

29: Wang J, You D, Wang H, Yang Y, Zhang D, Lv J, Luo S, Liao R, Ma L. Association between homocysteine and obesity: A meta-analysis. J Evid Based Med. 2021 Sep;14(3):208-217. doi: 10.1111/jebm.12412. Epub 2020 Nov 3. PMID: 33145936.

30: Babashahi M, Omidvar N, Yazdizadeh B, Heidari-Beni M, Joulaei H, Narmcheshm S, Zargaraan A, Kelishadi R. Systematic review and meta-analysis of the most common processed foods consumed by Iranian children. East Mediterr Health J. 2021 Sep 21;27(9):918-930. doi: 10.26719/emhj.21.032. PMID: 34569048.

31: Xu B, Fu J, Qiao Y, Cao J, Deehan EC, Li Z, Jin M, Wang X, Wang Y. Higher intake of microbiota-accessible carbohydrates and improved cardiometabolic risk factors: a meta-analysis and umbrella review of dietary management in patients with type 2 diabetes. Am J Clin Nutr. 2021 Jun 1;113(6):1515-1530. doi: 10.1093/ajcn/nqaa435. PMID: 33693499.

32: Cormick G, Ciapponi A, Cafferata ML, Cormick MS, Belizán JM. Calcium supplementation for prevention of primary hypertension. Cochrane Database Syst Rev. 2022 Jan 11;1(1):CD010037. doi: 10.1002/14651858.CD010037.pub4. PMID: 35014026; PMCID: PMC8748265.

33: Jiang YW, Zhang YB, Pan A. [Consumption of sugar-sweetened beverages and artificially sweetened beverages and risk of cardiovascular disease: a meta-analysis]. Zhonghua Yu Fang Yi Xue Za Zhi. 2021 Sep 6;55(9):1159-1167. Chinese. doi: 10.3760/cma.j.cn112150-20210729-00726. PMID: 34619938.

34: Yu J, Cao G, Yuan S, Luo C, Yu J, Cai M. Probiotic supplements and bone health in postmenopausal women: a meta-analysis of randomised controlled trials. BMJ Open. 2021 Mar 2;11(3):e041393. doi: 10.1136/bmjopen-2020-041393. PMID: 33653743; PMCID: PMC7929795.

35: Ahuja V, Aronen P, Pramodkumar TA, Looker H, Chetrit A, Bloigu AH, Juutilainen A, Bianchi C, La Sala L, Anjana RM, Pradeepa R, Venkatesan U, Jebarani S, Baskar V, Fiorentino TV, Timpel P, DeFronzo RA, Ceriello A, Del Prato S, Abdul-Ghani M, Keinänen-Kiukaanniemi S, Dankner R, Bennett PH, Knowler WC, Schwarz P, Sesti G, Oka R, Mohan V, Groop L, Tuomilehto J, Ripatti S, Bergman M, Tuomi T. Accuracy of 1-Hour Plasma Glucose During the Oral Glucose Tolerance Test in Diagnosis of Type 2 Diabetes in Adults: A Meta-analysis. Diabetes Care. 2021 Apr;44(4):1062-1069. doi: 10.2337/dc20-1688. Erratum in: Diabetes Care. 2021 Apr 30;: PMID: 33741697; PMCID: PMC8578930.

36: Matthews JJ, Dolan E, Swinton PA, Santos L, Artioli GG, Turner MD, Elliott-Sale KJ, Sale C. Effect of Carnosine or β-Alanine Supplementation on Markers of Glycemic Control and Insulin Resistance in Humans and Animals: A Systematic Review and Meta-analysis. Adv Nutr. 2021 Dec 1;12(6):2216-2231. doi: 10.1093/advances/nmab087. PMID: 34333586; PMCID: PMC8634390.

37: Moradi M, Sohrabi G, Golbidi M, Yarmohammadi S, Hemati N, Campbell MS, Moradi S, Kermani MAH, Farzaei MH. Effects of artichoke on blood pressure: A systematic review and meta-analysis. Complement Ther Med. 2021 Mar;57:102668. doi: 10.1016/j.ctim.2021.102668. Epub 2021 Jan 16. PMID: 33465383.

38: Nishi SK, Viguiliouk E, Blanco Mejia S, Kendall CWC, Bazinet RP, Hanley AJ, Comelli EM, Salas Salvadó J, Jenkins DJA, Sievenpiper JL. Are fatty nuts a weighty concern? A systematic review and meta-analysis and dose-response meta-regression of prospective cohorts and randomized controlled trials. Obes Rev. 2021 Nov;22(11):e13330. doi: 10.1111/obr.13330. Epub 2021 Sep 8. PMID: 34494363.

39: Wang MX, Gwee SXW, Pang J. Micronutrients Deficiency, Supplementation and Novel Coronavirus Infections-A Systematic Review and Meta-Analysis. Nutrients. 2021 May 10;13(5):1589. doi: 10.3390/nu13051589. PMID: 34068656; PMCID: PMC8151981.

40: Bakhtiary M, Morvaridzadeh M, Agah S, Rahimlou M, Christopher E, Zadro JR,

Heshmati J. Effect of Probiotic, Prebiotic, and Synbiotic Supplementation on Cardiometabolic and Oxidative Stress Parameters in Patients With Chronic Kidney Disease: A Systematic Review and Meta-analysis. Clin Ther. 2021 Mar;43(3):e71-e96. doi: 10.1016/j.clinthera.2020.12.021. Epub 2021 Jan 30. PMID: 33526314.

41: Coelho-Júnior HJ, Trichopoulou A, Panza F. Cross-sectional and longitudinal associations between adherence to Mediterranean diet with physical performance and cognitive function in older adults: A systematic review and meta-analysis. Ageing Res Rev. 2021 Sep;70:101395. doi: 10.1016/j.arr.2021.101395. Epub 2021 Jun 19. PMID: 34153553.

42: McMahon EJ, Campbell KL, Bauer JD, Mudge DW, Kelly JT. Altered dietary salt intake for people with chronic kidney disease. Cochrane Database Syst Rev. 2021 Jun 24;6(6):CD010070. doi: 10.1002/14651858.CD010070.pub3. PMID: 34164803; PMCID: PMC8222708.

43: Trieu K, Bhat S, Dai Z, Leander K, Gigante B, Qian F, Korat AVA, Sun Q, Pan XF, Laguzzi F, Cederholm T, de Faire U, Hellénius ML, Wu JHY, Risérus U, Marklund M. Biomarkers of dairy fat intake, incident cardiovascular disease, and all-cause mortality: A cohort study, systematic review, and meta-analysis. PLoS Med. 2021 Sep 21;18(9):e1003763. doi: 10.1371/journal.pmed.1003763. PMID: 34547017; PMCID: PMC8454979.

44: Do WL, Whitsel EA, Costeira R, Masachs OM, Le Roy CI, Bell JT, Staimez LR, Stein AD, Smith AK, Horvath S, Assimes TL, Liu S, Manson JE, Shadyab AH, Li Y, Hou L, Bhatti P, Jordahl K, Narayan KMV, Conneely KN. Epigenome-wide association study of diet quality in the Women's Health Initiative and TwinsUK cohort. Int J Epidemiol. 2021 May 17;50(2):675-684. doi: 10.1093/ije/dyaa215. PMID: 33354722; PMCID: PMC8128469.

45: Fang Y, Zhu J, Fan J, Sun L, Cai S, Fan C, Zhong Y, Li Y. Dietary Inflammatory Index in relation to bone mineral density, osteoporosis risk and fracture risk: a systematic review and meta-analysis. Osteoporos Int. 2021

Apr;32(4):633-643. doi: 10.1007/s00198-020-05578-8. Epub 2020 Aug 1. PMID: 32740669.

46: Duan Y, Shang B, Liang W, Du G, Yang M, Rhodes RE. Effects of eHealth-Based Multiple Health Behavior Change Interventions on Physical Activity, Healthy Diet, and Weight in People With Noncommunicable Diseases: Systematic Review and Meta-analysis. J Med Internet Res. 2021 Feb 22;23(2):e23786. doi: 10.2196/23786. PMID: 33616534; PMCID: PMC8074786.

47: Babu AF, Csader S, Lok J, Gómez-Gallego C, Hanhineva K, El-Nezami H, Schwab U. Positive Effects of Exercise Intervention without Weight Loss and Dietary Changes in NAFLD-Related Clinical Parameters: A Systematic Review and Meta-Analysis. Nutrients. 2021 Sep 8;13(9):3135. doi: 10.3390/nu13093135. PMID: 34579012; PMCID: PMC8466505.

48: Becerra-Tomás N, Paz-Graniel I, Hernández-Alonso P, Jenkins DJA, Kendall CWC, Sievenpiper JL, Salas-Salvadó J. Nut consumption and type 2 diabetes risk: a systematic review and meta-analysis of observational studies. Am J Clin Nutr. 2021 Apr 6;113(4):960-971. doi: 10.1093/ajcn/nqaa358. PMID: 33471083.

49: van der Meer TP, Chung MK, van Faassen M, Makris KC, van Beek AP, Kema IP, Wolffenbuttel BHR, van Vliet-Ostaptchouk JV, Patel CJ. Temporal exposure and consistency of endocrine disrupting chemicals in a longitudinal study of individuals with impaired fasting glucose. Environ Res. 2021 Jun;197:110901. doi: 10.1016/j.envres.2021.110901. Epub 2021 Feb 20. PMID: 33617867.

50: Sahebkar A, Katsiki N, Ward N, Reiner Ž. Flaxseed Supplementation Reduces Plasma Lipoprotein(a) Levels: A Meta-Analysis. Altern Ther Health Med. 2021 May;27(3):50-53. PMID: 31634874.

51: Lin M, Heizati M, Wang L, Nurula M, Yang Z, Wang Z, Abudoyreyimu R, Wu Z, Li N. A systematic review and meta-analysis of effects of spironolactone on blood pressure, glucose, lipids, renal function, fibrosis and inflammation in patients with hypertension and diabetes. Blood Press. 2021 Jun;30(3):145-153. doi:

10.1080/08037051.2021.1880881. Epub 2021 Mar 8. PMID: 33682538.

52: Wang Y, Gallegos JL, Haskell-Ramsay C, Lodge JK. Effects of chronic consumption of specific fruit (berries, citrus and cherries) on CVD risk factors: a systematic review and meta-analysis of randomised controlled trials. Eur J Nutr. 2021 Mar;60(2):615-639. doi: 10.1007/s00394-020-02299-w. Epub 2020 Jun 13. Erratum in: Eur J Nutr. 2021 Jan 23;: PMID: 32535781; PMCID: PMC7900084.

53: Kim S, Park M, Song R. Effects of self-management programs on behavioral modification among individuals with chronic disease: A systematic review and meta-analysis of randomized trials. PLoS One. 2021 Jul 23;16(7):e0254995. doi: 10.1371/journal.pone.0254995. PMID: 34297741; PMCID: PMC8301623.

54: Shahinfar H, Jayedi A, Khan TA, Shab-Bidar S. Coffee consumption and cardiovascular diseases and mortality in patients with type 2 diabetes: A systematic review and dose-response meta-analysis of cohort studies. Nutr Metab Cardiovasc Dis. 2021 Aug 26;31(9):2526-2538. doi: 10.1016/j.numecd.2021.05.014. Epub 2021 May 24. PMID: 34112583.

55: Macey R, Walsh T, Riley P, Glenny AM, Worthington HV, Clarkson JE, Ricketts D. Electrical conductance for the detection of dental caries. Cochrane Database Syst Rev. 2021 Mar 16;3(3):CD014547. doi: 10.1002/14651858.CD014547. PMID: 33724442; PMCID: PMC8406820.

56: Semlitsch T, Krenn C, Jeitler K, Berghold A, Horvath K, Siebenhofer A. Long-term effects of weight-reducing diets in people with hypertension. Cochrane Database Syst Rev. 2021 Feb 8;2(2):CD008274. doi: 10.1002/14651858.CD008274.pub4. PMID: 33555049; PMCID: PMC8093137.

57: Buzzetti E, Linden A, Best LM, Madden AM, Roberts D, Chase TJG, Freeman SC, Cooper NJ, Sutton AJ, Fritche D, Milne EJ, Wright K, Pavlov CS, Davidson BR, Tsochatzis E, Gurusamy KS. Lifestyle modifications for nonalcohol-related fatty liver disease: a network meta-analysis. Cochrane Database Syst Rev. 2021 Jun 11;6(6):CD013156. doi: 10.1002/14651858.CD013156.pub2. PMID: 34114650; PMCID:

PMC8193812.

58: Karimi E, Bitarafan S, Mousavi SM, Zargarzadeh N, Mokhtari P, Hawkins J, Meysamie A, Koohdani F. The effect of vitamin D supplementation on fibroblast growth factor-23 in patients with chronic kidney disease: A systematic review and meta-analysis. Phytother Res. 2021 Oct;35(10):5339-5351. doi: 10.1002/ptr.7139. Epub 2021 Apr 30. PMID: 33928687.

59: Yao N, Yan S, Guo Y, Wang H, Li X, Wang L, Hu W, Li B, Cui W. The association between carotenoids and subjects with overweight or obesity: a systematic review and meta-analysis. Food Funct. 2021 Jun 8;12(11):4768-4782. doi: 10.1039/d1fo00004g. PMID: 33977977.

60: Pu Y, Zhu G, Xu Y, Zheng S, Tang B, Huang H, Wu IXY, Huang D, Liu Y, Zhang X. Association Between Vitamin D Exposure and Head and Neck Cancer: A Systematic Review With Meta-Analysis. Front Immunol. 2021 Feb 23;12:627226. doi: 10.3389/fimmu.2021.627226. PMID: 33732250; PMCID: PMC7959800.

61: Nonterah EA, Crowther NJ, Oduro A, Agongo G, Micklesfield LK, Boua PR, Choma SSR, Mohamed SF, Sorgho H, Tollman SM, Norris SA, Raal FJ, Grobbee DE, Ramsay M, Bots ML, Klipstein-Grobusch K; as part of the H3Africa AWI-Gen study. Poor cardiovascular health is associated with subclinical atherosclerosis in apparently healthy sub-Saharan African populations: an H3Africa AWI-Gen study. BMC Med. 2021 Feb 10;19(1):30. doi: 10.1186/s12916-021-01909-6. PMID: 33563289; PMCID: PMC7874493.

62: Yazdanpanah Z, Beigrezaei S, Mohseni-Takalloo S, Soltani S, Rajaie SH, Zohrabi T, Kaviani M, Forbes SC, Baker JS, Salehi-Abargouei A. Does exercise affect bone mineral density and content when added to a calorie-restricted diet? A systematic review and meta-analysis of controlled clinical trials. Osteoporos Int. 2022 Feb;33(2):339-354. doi: 10.1007/s00198-021-06187-9. Epub 2021 Oct 13. PMID: 34643754.

63: Vanoni FO, Milani GP, Agostoni C, Treglia G, Faré PB, Camozzi P, Lava SAG,

Bianchetti MG, Janett S. Magnesium Metabolism in Chronic Alcohol-Use Disorder: Meta-Analysis and Systematic Review. Nutrients. 2021 Jun 7;13(6):1959. doi: 10.3390/nu13061959. PMID: 34200366; PMCID: PMC8229336.

64: Komolafe O, Buzzetti E, Linden A, Best LM, Madden AM, Roberts D, Chase TJ, Fritche D, Freeman SC, Cooper NJ, Sutton AJ, Milne EJ, Wright K, Pavlov CS, Davidson BR, Tsochatzis E, Gurusamy KS. Nutritional supplementation for nonalcohol-related fatty liver disease: a network meta-analysis. Cochrane Database Syst Rev. 2021 Jul 19;7(7):CD013157. doi: 10.1002/14651858.CD013157.pub2. PMID: 34280304; PMCID: PMC8406904.

65: Cicero AFG, Kennedy C, Knežević T, Bove M, Georges CMG, Šatrauskienė A, Toth PP, Fogacci F. Efficacy and Safety of Armolipid Plus®: An Updated PRISMA Compliant Systematic Review and Meta-Analysis of Randomized Controlled Clinical Trials. Nutrients. 2021 Feb 16;13(2):638. doi: 10.3390/nu13020638. PMID: 33669333; PMCID: PMC7920267.

66: Gold N, Yau A, Rigby B, Dyke C, Remfry EA, Chadborn T. Effectiveness of Digital Interventions for Reducing Behavioral Risks of Cardiovascular Disease in Nonclinical Adult Populations: Systematic Review of Reviews. J Med Internet Res. 2021 May 14;23(5):e19688. doi: 10.2196/19688. PMID: 33988126; PMCID: PMC8164125.

67: Moodi V, Abedi S, Esmaeilpour M, Asbaghi O, Izadi F, Shirinbakhshmasoleh M, Behrouzian M, Shahriari A, Ghaedi E, Miraghajani M. The effect of grapes/grape products on glycemic response: A systematic review and meta-analysis of randomized controlled trials. Phytother Res. 2021 Sep;35(9):5053-5067. doi: 10.1002/ptr.7135. Epub 2021 Apr 24. PMID: 33893683.

68: Cabalín C, Iturriaga C, Pérez-Mateluna G, Echeverría D, Camargo CA Jr, Borzutzky A. Vitamin D status and supplementation in Antarctica: a systematic review and meta- analysis. Int J Circumpolar Health. 2021 Dec;80(1):1926133. doi: 10.1080/22423982.2021.1926133. PMID: 33983101; PMCID: PMC8128169.

69: Hasani M, Mansour A, Asayesh H, Djalalinia S, Mahdavi Gorabi A, Ochi F,

Qorbani M. Effect of glutamine supplementation on cardiometabolic risk factors and inflammatory markers: a systematic review and meta-analysis. BMC Cardiovasc Disord. 2021 Apr 17;21(1):190. doi: 10.1186/s12872-021-01986-8. PMID: 33865313; PMCID: PMC8053267.

70: Wiebe N, Ye F, Crumley ET, Bello A, Stenvinkel P, Tonelli M. Temporal Associations Among Body Mass Index, Fasting Insulin, and Systemic Inflammation: A Systematic Review and Meta-analysis. JAMA Netw Open. 2021 Mar 1;4(3):e211263. doi: 10.1001/jamanetworkopen.2021.1263. PMID: 33710289; PMCID: PMC7955272.

71: Asbaghi O, Moradi S, Nezamoleslami S, Moosavian SP, Hojjati Kermani MA, Lazaridi AV, Miraghajani M. The Effects of Magnesium Supplementation on Lipid Profile Among Type 2 Diabetes Patients: a Systematic Review and Meta-analysis of Randomized Controlled Trials. Biol Trace Elem Res. 2021 Mar;199(3):861-873. doi: 10.1007/s12011-020-02209-5. Epub 2020 May 28. PMID: 32468224.

72: Perna S, Ilyas Z, Giacosa A, Gasparri C, Peroni G, Faliva MA, Rigon C, Naso M, Riva A, Petrangolini G, A Redha A, Rondanelli M. Is Probiotic Supplementation Useful for the Management of Body Weight and Other Anthropometric Measures in Adults Affected by Overweight and Obesity with Metabolic Related Diseases? A Systematic Review and Meta-Analysis. Nutrients. 2021 Feb 19;13(2):666. doi: 10.3390/nu13020666. PMID: 33669580; PMCID: PMC7922558.

73: Beaulieu K, Blundell JE, van Baak MA, Battista F, Busetto L, Carraça EV, Dicker D, Encantado J, Ermolao A, Farpour-Lambert N, Pramono A, Woodward E, Bellicha A, Oppert JM. Effect of exercise training interventions on energy intake and appetite control in adults with overweight or obesity: A systematic review and meta-analysis. Obes Rev. 2021 Jul;22 Suppl 4(Suppl 4):e13251. doi: 10.1111/obr.13251. Epub 2021 May 5. PMID: 33949089; PMCID: PMC8365695.

74: He J, Kong D, Yang Z, Guo R, Amponsah AE, Feng B, Zhang X, Zhang W, Liu A, Ma J, O'Brien T, Cui H. Clinical efficacy on glycemic control and safety of mesenchymal stem cells in patients with diabetes mellitus: Systematic review and meta-analysis of RCT data. PLoS One. 2021 Mar 11;16(3):e0247662. doi:

10.1371/journal.pone.0247662. PMID: 33705413; PMCID: PMC7951834.

75: Nkambule SJ, Moodley I, Kuupiel D, Mashamba-Thompson TP. Association between food insecurity and key metabolic risk factors for diet-sensitive non-communicable diseases in sub-Saharan Africa: a systematic review and meta-analysis. Sci Rep. 2021 Mar 4;11(1):5178. doi: 10.1038/s41598-021-84344-0. PMID: 33664339; PMCID: PMC7933340.

76: Kachroo N, Lange D, Penniston KL, Stern J, Tasian G, Bajic P, Wolfe AJ, Suryavanshi M, Ticinesi A, Meschi T, Monga M, Miller AW. Meta-analysis of Clinical Microbiome Studies in Urolithiasis Reveal Age, Stone Composition, and Study Location as the Predominant Factors in Urolithiasis-Associated Microbiome Composition. mBio. 2021 Aug 31;12(4):e0200721. doi: 10.1128/mBio.02007-21. Epub 2021 Aug 10. PMID: 34372696; PMCID: PMC8406293.

77: Vahedian-Azimi A, Abbasifard M, Rahimi-Bashar F, Guest PC, Majeed M, Mohammadi A, Banach M, Jamialahmadi T, Sahebkar A. Effectiveness of Curcumin on Outcomes of Hospitalized COVID-19 Patients: A Systematic Review of Clinical Trials. Nutrients. 2022 Jan 7;14(2):256. doi: 10.3390/nu14020256. PMID: 35057437; PMCID: PMC8779570.

78: Conley MM, McFarlane CM, Johnson DW, Kelly JT, Campbell KL, MacLaughlin HL. Interventions for weight loss in people with chronic kidney disease who are overweight or obese. Cochrane Database Syst Rev. 2021 Mar 30;3(3):CD013119. doi: 10.1002/14651858.CD013119.pub2. PMID: 33782940; PMCID: PMC8094234.

79: Kirkham AA, Beka V, Prado CM. The effect of caloric restriction on blood pressure and cardiovascular function: A systematic review and meta-analysis of randomized controlled trials. Clin Nutr. 2021 Mar;40(3):728-739. doi: 10.1016/j.clnu.2020.06.029. Epub 2020 Jul 1. PMID: 32675017.

80: Schönenberger KA, Schüpfer AC, Gloy VL, Hasler P, Stanga Z, Kaegi-Braun N, Reber E. Effect of Anti-Inflammatory Diets on Pain in Rheumatoid Arthritis: A Systematic Review and Meta-Analysis. Nutrients. 2021 Nov 24;13(12):4221. doi:

10.3390/nu13124221. PMID: 34959772; PMCID: PMC8706441.

81: Asbaghi O, Ashtary-Larky D, Bagheri R, Moosavian SP, Olyaei HP, Nazarian B, Rezaei Kelishadi M, Wong A, Candow DG, Dutheil F, Suzuki K, Alavi Naeini A. Folic Acid Supplementation Improves Glycemic Control for Diabetes Prevention and Management: A Systematic Review and Dose-Response Meta-Analysis of Randomized Controlled Trials. Nutrients. 2021 Jul 9;13(7):2355. doi: 10.3390/nu13072355. PMID: 34371867; PMCID: PMC8308657.

82: Lim SY, Wang R, Tan DJH, Ng CH, Lim WH, Quek J, Syn N, Nah BKY, Wong ET, Huang DQ, Vathsala A, Siddiqui MS, Fung J, Muthiah MD, Tan EX. A meta-analysis of the cumulative incidence, risk factors, and clinical outcomes associated with chronic kidney disease after liver transplantation. Transpl Int. 2021 Dec;34(12):2524-2533. doi: 10.1111/tri.14149. Epub 2021 Nov 15. PMID: 34714569.

83: Ebrahimzadeh A, Abbasi F, Ebrahimzadeh A, Jibril AT, Milajerdi A. Effects of curcumin supplementation on inflammatory biomarkers in patients with Rheumatoid Arthritis and Ulcerative colitis: A systematic review and meta-analysis. Complement Ther Med. 2021 Sep;61:102773. doi: 10.1016/j.ctim.2021.102773. Epub 2021 Aug 31. PMID: 34478838.

84: Lam CN, Watt AE, Isenring EA, de van der Schueren MAE, van der Meij BS. The effect of oral omega-3 polyunsaturated fatty acid supplementation on muscle maintenance and quality of life in patients with cancer: A systematic review and meta-analysis. Clin Nutr. 2021 Jun;40(6):3815-3826. doi: 10.1016/j.clnu.2021.04.031. Epub 2021 Apr 27. PMID: 34130028.

85: Mishu MP, Uphoff E, Aslam F, Philip S, Wright J, Tirbhowan N, Ajjan RA, Al Azdi Z, Stubbs B, Churchill R, Siddiqi N. Interventions for preventing type 2 diabetes in adults with mental disorders in low- and middle-income countries. Cochrane Database Syst Rev. 2021 Feb 16;2(2):CD013281. doi: 10.1002/14651858.CD013281.pub2. PMID: 33591592; PMCID: PMC8092639.

86: Lari A, Fatahi S, Sohouli MH, Shidfar F. The Impact of Chromium

Supplementation on Blood Pressure: A Systematic Review and Dose-Response Meta-Analysis of Randomized-Controlled Trials. High Blood Press Cardiovasc Prev. 2021 Jul;28(4):333-342. doi: 10.1007/s40292-021-00456-8. Epub 2021 Jun 3. PMID: 34081296.

87: Mohammad A, Falahi E, Mohd Yusof BN, Hanipah ZN, Sabran MR, Mohamad Yusof L, Gheitasvand M. The effects of the ginger supplements on inflammatory parameters in type 2 diabetes patients: A systematic review and meta-analysis of randomised controlled trials. Clin Nutr ESPEN. 2021 Dec;46:66-72. doi: 10.1016/j.clnesp.2021.10.013. Epub 2021 Oct 22. PMID: 34857250.

88: Jahrami HA, Faris ME, I Janahi A, I Janahi M, Abdelrahim DN, Madkour MI, Sater MS, Hassan AB, Bahammam AS. Does four-week consecutive, dawn-to-sunset intermittent fasting during Ramadan affect cardiometabolic risk factors in healthy adults? A systematic review, meta-analysis, and meta-regression. Nutr Metab Cardiovasc Dis. 2021 Jul 22;31(8):2273-2301. doi: 10.1016/j.numecd.2021.05.002. Epub 2021 May 25. PMID: 34167865.

89: Rodrigues C, Pinto A, Faria A, Teixeira D, van Wegberg AMJ, Ahring K, Feillet F, Calhau C, MacDonald A, Moreira-Rosário A, Rocha JC. Is the Phenylalanine-Restricted Diet a Risk Factor for Overweight or Obesity in Patients with Phenylketonuria (PKU)? A Systematic Review and Meta-Analysis. Nutrients. 2021 Sep 28;13(10):3443. doi: 10.3390/nu13103443. PMID: 34684443; PMCID: PMC8538431.

90: Asbaghi O, Ashtary-Larky D, Bagheri R, Moosavian SP, Nazarian B, Afrisham R, Kelishadi MR, Wong A, Dutheil F, Suzuki K, Alavi Naeini A. Effects of Folic Acid Supplementation on Inflammatory Markers: A Grade-Assessed Systematic Review and Dose-Response Meta-Analysis of Randomized Controlled Trials. Nutrients. 2021 Jul 6;13(7):2327. doi: 10.3390/nu13072327. PMID: 34371837; PMCID: PMC8308638.

91: Kodama S, Horikawa C, Fujihara K, Hatta M, Takeda Y, Nedachi R, Kato K, Watanabe K, Sone H. Meta-analytic research of the dose-response relationship between salt intake and risk of heart failure. Hypertens Res. 2021

Jul;44(7):885-887. doi: 10.1038/s41440-021-00632-2. Epub 2021 Mar 2. Erratum in: Hypertens Res. 2021 Mar 25;: PMID: 33654246.

92: Zhang Y, Lu Y, Wang S, Yang L, Xia H, Sun G. Excessive Vitamin A Supplementation Increased the Incidence of Acute Respiratory Tract Infections: A Systematic Review and Meta-Analysis. Nutrients. 2021 Nov 26;13(12):4251. doi: 10.3390/nu13124251. PMID: 34959803; PMCID: PMC8706818.
