# Supplementary material for: Perspectives of Dietary Assessment in Human Health and Disease
Source: Nutrients. 2022 Feb 16;14(4):830. doi: 10.3390/nu14040830 (PMC8877528; doi:10.3390/nu14040830)
Supplement: Supplementary file 1 [file nutrients-14-00830-s001.zip › Table S7.pdf]

Table S7 - PubMed search keywords "dietary assessment human health disease", filters "1 year" and "books and documents"

starting date 08/02/2022

Type of article: Books and documents

n = 2

1: Moleyar-Narayana P, Ranganathan S. Cancer Screening. 2021 Oct 9. In: StatPearls [Internet]. Treasure Island (FL): StatPearls Publishing; 2022 Jan–. PMID: 33085285.

2: Adamson D, Blazeby J, Porter C, Hurt C, Griffiths G, Nelson A, Sewell B, Jones M, Svobodova M, Fitzsimmons D, Nixon L, Fitzgibbon J, Thomas S, Millin A, Crosby T, Staffurth J, Byrne A. Palliative radiotherapy combined with stent insertion to reduce recurrent dysphagia in oesophageal cancer patients: the ROCS RCT. Health Technol Assess. 2021 May;25(31):1-144. doi: 10.3310/hta25310. PMID: 34042566; PMCID: PMC8182443.
